# In Silico Pharmacokinetic Profiling of the Identified Bioactive Metabolites of *Pergularia tomentosa* L. Latex Extract and In Vitro Cytotoxic Activity via the Induction of Caspase-Dependent Apoptosis with S-Phase Arrest

**DOI:** 10.3390/ph15091132

**Published:** 2022-09-09

**Authors:** Amr S. Abouzied, Marwa M. Abd-Rabo, Bader Huwaimel, Suliman A. Almahmoud, Afnan Abdulkareem Almarshdi, Fai Mutaz Alharbi, Sulafa Salem Alenzi, Bayan Naef Albsher, Ahmed Alafnan

**Affiliations:** 1 Department of Pharmaceutical Chemistry, College of Pharmacy, University of Hail, Hail 81442, Saudi Arabia; 2 Department of Pharmaceutical Chemistry, National Organization for Drug Control and Research (NODCAR), Giza 12553, Egypt; 3 Department of Hormone Evaluation, National Organization for Drug Control and Research (NODCAR), Giza 12553, Egypt; 4 Department of Medicinal Chemistry and Pharmacognosy, College of Pharmacy, Qassim University, Buraidah 51452, Saudi Arabia; 5 College of Pharmacy, University of Hail, Hail 81411, Saudi Arabia; 6 Department of Pharmacology and Toxicology, College of Pharmacy, University of Hail, Hail 81411, Saudi Arabia

**Keywords:** *Pergularia tomentosa* L. latex, cytotoxicity, apoptosis, pharmacokinetic, docking

## Abstract

The in vitro cytotoxic efficacy of plant latex from *Pergularia tomentosa* L. was studied using five human cancer cell lines: HeLa cells (cervical carcinoma cells), A-549 (lung carcinoma), Panc-1 (pancreatic carcinoma cells), MDA-MB-231 (metastatic mammary adenocarcinoma), and MRC-5 (lung fibroblast cell line) cells. The phytonutrient content of plant latex was identified using the liquid chromatography/mass spectra-quadrupole time of flight (LC/MS-QTOF) technique. In silico studies of polyphenols were carried out to clarify the potential mode of action of the plant latex’s constituents. The treatment of different tumor cell lines with different concentrations of plant latex revealed a potent efficacy on the human lung carcinoma cell line (A-549) (IC_50_ = 3.89 µg/mL) compared with that with vinblastine as a positive control (IC_50_ = 7.12 µg/mL). The effect of the potent concentration of plant latex on the A-549 cell line induced cell arrest, upregulated the expression of pre-apoptotic markers, and downregulated the expression of antiapoptotic markers. Seven identified polyphenols were selected for the in silico study. A docking assessment using the epidermal growth factor receptor kinase (EGFRk) and eltronib as a positive control showed a higher affinity for the enzyme receptor of the selected polyphenols, except for methyl orsellinate and ginkgotoxin. The ADMET assessment demonstrated the inhibitory effect of the polyphenols on CYP450, except for ouabagenin and xanthyletine. The selected polyphenols obey Lipinski’s drug-likeness with no significant toxicity effect. In conclusion, the plant latex of *P. tomentosa* L. showed cytotoxic activity on the A-549 cell line, and the selected polyphenols showed a promising prodrug agent with a low profile of toxicity in the study.

## 1. Introduction

Cancer is the leading cause of death in 112 of 118 nations [1]. The traditional treatment for cancer chemotherapy induces various kinds of toxicities such as myelotoxicity, cardiotoxicity, renal toxicity, and pulmonary toxicity [2]. Furthermore, surgery and radiotherapy lead to patient stress associated with a debility in all bodily functions [3,4]. African and Asian regions are enriched with medicinal plants that are used for various [5,6,7,8].

Natural products are the alternative way to develop new antitumor agents with safer margins than traditional routine cancer treatments [2,9]; secondary metabolite plant products such as flavonoids, alkaloids, terpenoids, saponins, and others are documented as anticancer agents through several mechanisms including apoptosis induction, immune system modulation, angiogenesis inhibition, and the inhibition of several stages of tumorigenesis and associated inflammatory processes [3,4,5].

*Pergularia tomentosa* L. is a climbing weed plant and a perennial shrub. It belongs to the family *Asclepiadaceous* and is distributed in North African and Asian countries, such as Tunisia, Egypt, Saudia Arabia, etc. [10,11,12,13]. *P. tomentosa* L. is enriched with polyphenols such as phenolic acids, alkaloids, cardiac glycosides, and flavonoids [14,15]. In traditional medicine, *P. tomentosa* L. is used in the treatment of skin infections, constipation, abortions, and tuberculosis [13,14]. Furthermore, the extracts of different parts of *P. tomentosa* L. revealed its antifungal, antioxidant, antiproliferative, and cytotoxic effects [11,12,14,16]. Previous study has documented that *Pergularia tomentosa* L. latex extract exhibited moderate cytotoxic activities against several cancer cell lines, colon carcinoma (HCT-116), hepatocellular carcinoma (HepG2), and breast carcinoma (MCF-7) [11].

This study is designed to evaluate the in vitro cytotoxic efficacy of *P. tomentosa* L. latex on other cell lines different from that previously used and investigate a potential plant-latex activities as apoptotic inducer on the most affected cell line and furthermore to identify polyphenol compounds in plant latex using the LC/MS-QTOF technique, to characterize the polyphenol compounds using docking, and to employ ADMET studies to project new natural pharmaceuticals in the field of cancer therapy.

## 2. Results

### 2.1. In Vitro Cytotoxicity Study

**Figure 1:**
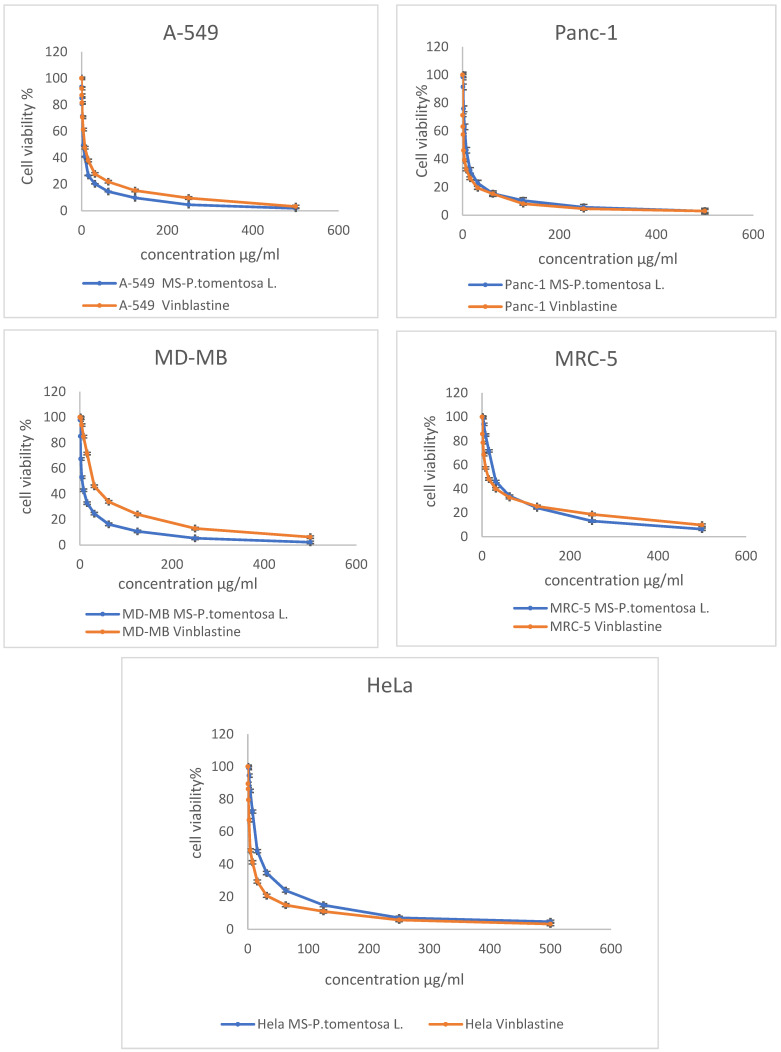
Demonstrated dose response profile of the plant latex from *P. tomentosa* L. on the cell viability of A-495, Panc-1, MDA-MB-231, MRC-5, and HeLa cell lines following incubation for 24 h. All data are represented as the mean ± SD, *n* = 3.

The MTT assay results reveal that the cancer cell line (A-549) was the most affected cell line following treatment with different concentrations of plant latex. This conclusion is based on the statistical difference between the concentration of the tested samples and vinblastine (the positive control) to induce a 50% inhibition (IC_50_) on the viability of the treated cell line. The effective IC_50_ (3.89 µg/mL, *p* < 0.01) of the plant latex was lower than the IC_50_ (7.12 µg/mL) of vinblastine against the viability of the A-549 cell line. The IC_50_ of the plant latex (5.11 µg/mL, *p* < 0.01) was higher than the IC_50_ (2.3 µg/mL) of vinblastine against the viability of the MDA-MB-231 cell line. The IC_50_ of plant latex (6.93 µg/mL, *p* < 0.01) was higher than the IC_50_ (1.65 µg/mL) of vinblastine against the viability of the Panc-1 cell line. The IC_50_ of the plant latex (15.02 µg/mL, *p* < 0.01) was higher than the IC_50_ (3.8 µg/mL) of vinblastine against the viability of the HeLa cell line. The IC_50_ of plant latex (28.85 µg/mL, *p* < 0.01) was higher than the IC_50_ (14.06 µg/mL) of vinblastine against the viability of MRC-5 (Figure 1). Collectively, the A-549 cell line was selected for the further assessment of the potential role of plant latex as a cytotoxic agent.

### 2.2. Cell Cycle Analysis

**Figure 2:**
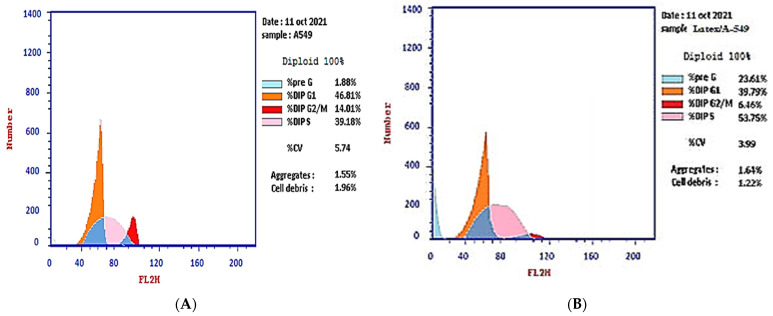
Photograph of the flow cytometry analysis shows the effect of 3.8 µg/mL of the plant latex of *P. tomentosa* L. compared to the negative control on the phases of the cell cycle of the lung cancer cell line (A-549). (**A**) negative control and (**B**) plant latex of *P. tomentosa* L.

The effect of the IC_50_ of plant latex on the checkpoints of the A-549 cell cycle provided an estimate of the efficacy in inducing cell arrest. The treated A-549 cell line subpopulation increased in the pre-G2 phase (23.61%) compared with the subpopulation of the untreated A-549 cell line (1.88%). Concomitantly, the subpopulation of treated A-549 decreased (39.79%) in the G1 phase compared with the subpopulation of untreated A-549 (46.81%). The treated A-549 subpopulation increased (53.75%) in the S phase compared with the subpopulation of untreated A-549 (39.18%). The treated A-549 subpopulation decreased (6.46%) in the SG2/M phase, compared with the subpopulation of untreated A-549 (14.01%). These results project the mutation of DNA in the S phase that could affect cell division in the M phase. Collectively, the IC_50_ of the plant latex treatment provoked the inhibition of tumor cell growth by inducing A-549 cell arrest (Table 1, Figure 2).

### 2.3. Annexin V-FITC Assay

**Figure 3:**
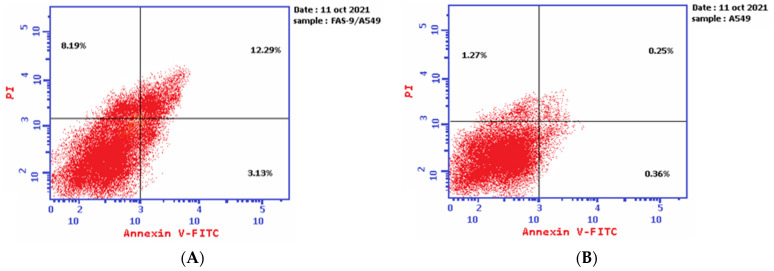
Demonstrated effect of *P. tomentosa* L. latex (IC_50_ = 3.8 ug/mL) (**A**) on the percentage of Annexin V-FITC-positive staining monolayer A-549 cells versus the control (**B**) using flow cytometry.

The results of the Annexin V-FITC assay reveal the ability of plant latex to induce A-549 cell line apoptosis. The IC_50_ of plant latex increased the Annexin V-FITC-positive apoptotic cell population in the early phase compared with the population of untreated cells (3.13% and 0.36%, respectively). In addition, the treated A-549 showed a late apoptotic cell population higher than the untreated cell line (12.29% and 0.25%, respectively), along with an 8.19% increase in the necrotic cells of the treated A-549 cell line relative to the untreated A-549 line (1.27%). Collectively, the Annexin V-FITC assay revealed the efficacy of plant latex to induce programmed cell death in the treated human lung carcinoma A-549 cell line (Table 2, Figure 3).

### 2.4. Apoptotic Markers of Cytotoxicity

**Figure 4:**
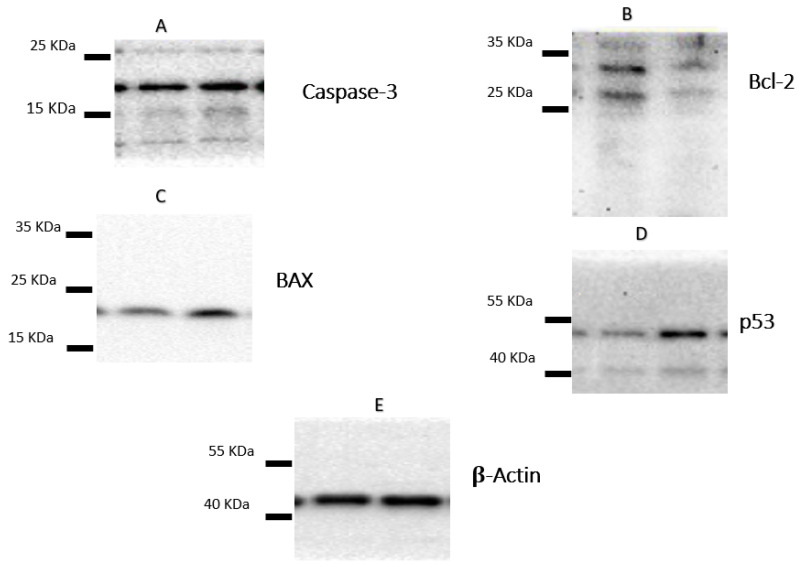
The figure showed the protein expression of (**A**) caspase-3, (**B**) Bcl-2, (**C**) Bax, and (**D**) p53, relative to (**E**) β-actin-mediated apoptosis in lung cancer cell lines (A-549) following treatments with *P. tomentosa* L. latex (IC_50_ = 3.8 µg/mL).

Following the treatment of A-549 with the IC_50_ of plant latex for 24 h, the proapoptotic protein markers BAX and caspase-3, as well as tumor suppressor p53 protein expression, were upregulated. Moreover, there was a downregulation of the expression of the antiapoptosis protein Bcl-2. These findings are consistent with previous findings concerning Annexin V staining, which demonstrate that the cell death pattern was related to apoptosis rather than necrosis (Table 3, Figure 4).

### 2.5. Screening of Secondary Metabolites

**Figure 5:**
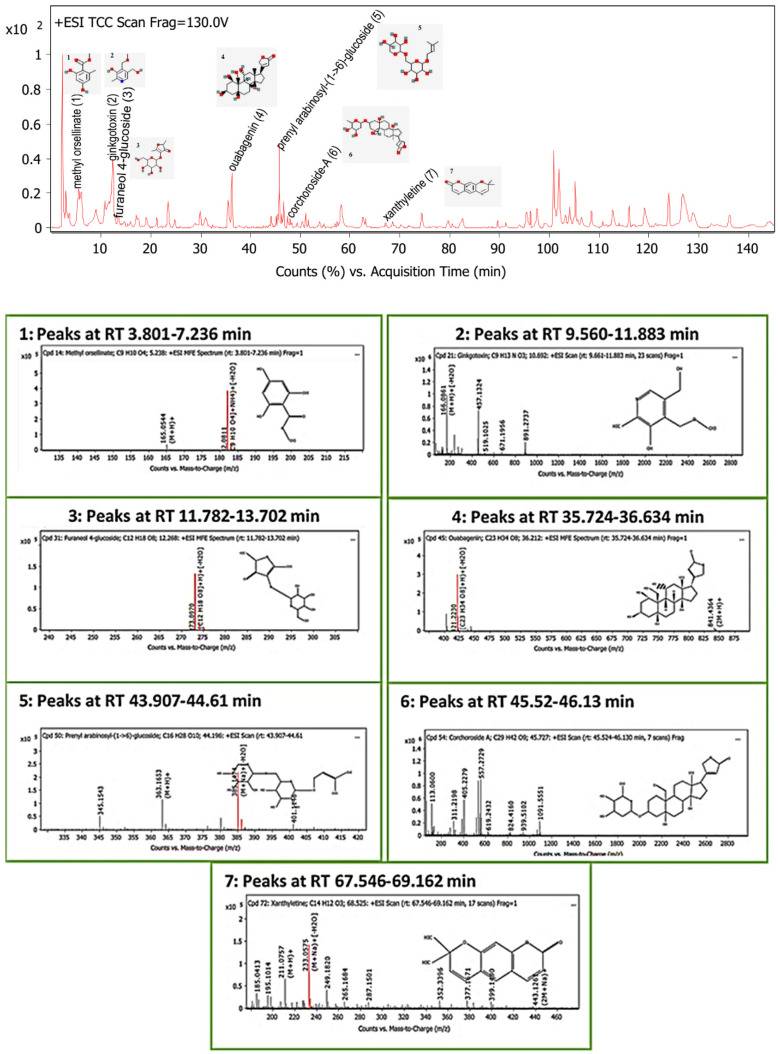
Base peak chromatogram of the identified bioactive metabolites in *P. tomentosa* L. latex: methyl orsellinate (**1**), ginkgotoxin (**2**), furaneol 4-glucoside (**3**), ouabagenin (**4**), prenyl arabinosyl-(1->6)-glucoside (**5**), corchoroside-A (**6**), and xanthyletine (**7**).

The LC-MS screening revealed that plant latex is enriched with polyphenols. In the present study, seven compounds with concentrations exceeding 99% were selected for in silico studies. The methyl orsellinate, a phenolic acid compound, peaked at a retention time (RT) of 5.32 min, with *m*/*z* 182.0811. The molecular formula was [C_9_H_10_O_4_] with a fragmented product of *m*/*z* 165.0544 in a positive ion mode (M+H)^+^, and the molecular formula was [C_9_H_8_O_3_]. The ginkgotoxin compound peaked at an RT of 10.692 with *m*/*z* 166.0862, and the molecular formula was [C_9_H_13_NO_3_] with a fragmented product of *m*/*z* 452.1768 in a negative ion mode, and the molecular formula was [C_18_H_23_N_9_O_4_]. Furaneol 4-glucoside, a glycosyl compound, peaked at an RT of 12.268 min with *m*/*z* 273.097, and the molecular formula was [C_12_H_18_O_8_]. Ouabagenin, a cardiotonic steroid compound, peaked at an RT of 36.212 with *m*/*z* 421.223, and the molecular formula was [C_23_H_34_O_8_]. Prenyl arabinosyl-(1->6)-glucoside, a glycoside compound, peaked at an RT of 44.2 with *m*/*z* 385.15, and the molecular formula was C_16_H_28_O_10_. Corchoroside A, a cardenolide glycoside compound, peaked at an RT of 45.73 with *m*/*z* 534.28, and the molecular formula was [C_29_H_42_O_9_]. Xanthyletine, a coumarin derivative compound, peaked at an RT of 68.53 with *m*/*z* 228.1, and the molecular formula was [C_14_H_12_O_3_]. LC chromatograms with retention times as well as all mass spectra are represented in Figure 5.

### 2.6. In Silico Study

#### 2.6.1. Molecular Docking of Screened Metabolites

**Figure 6:**
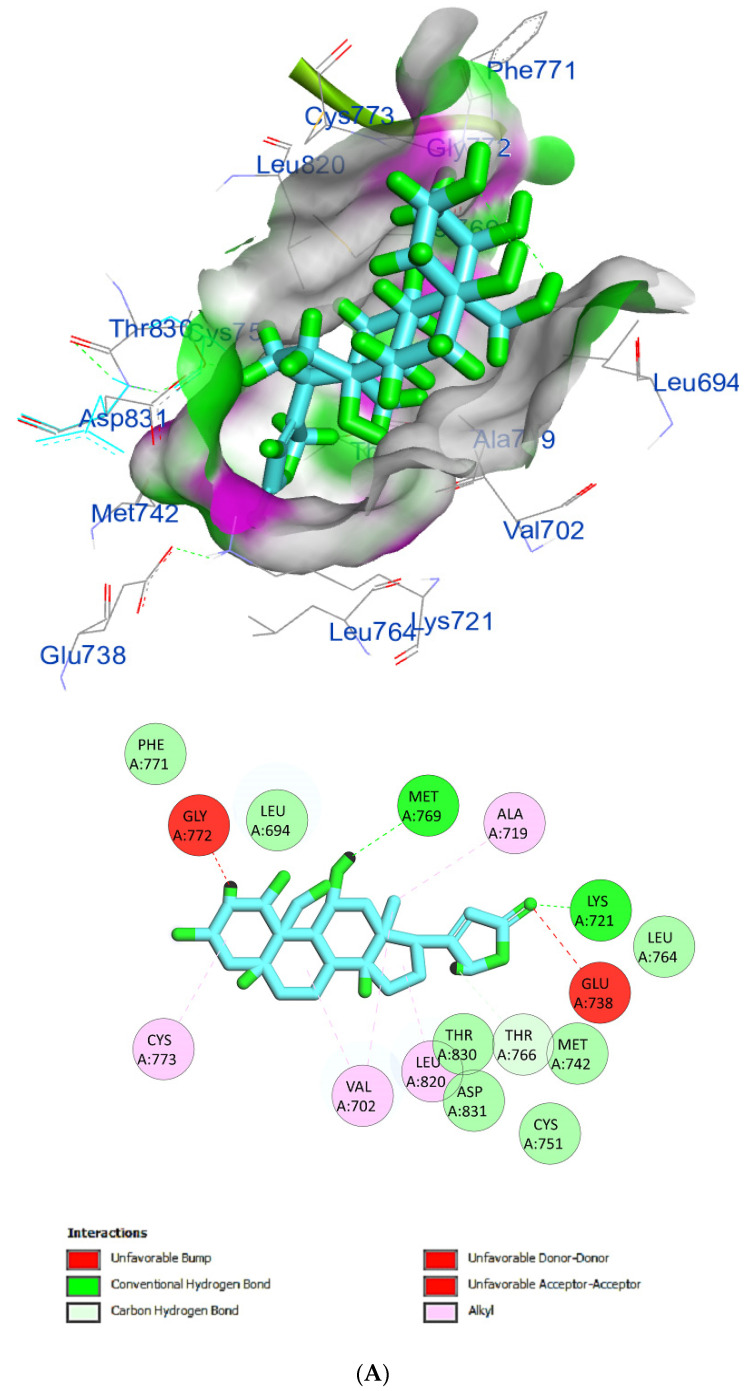

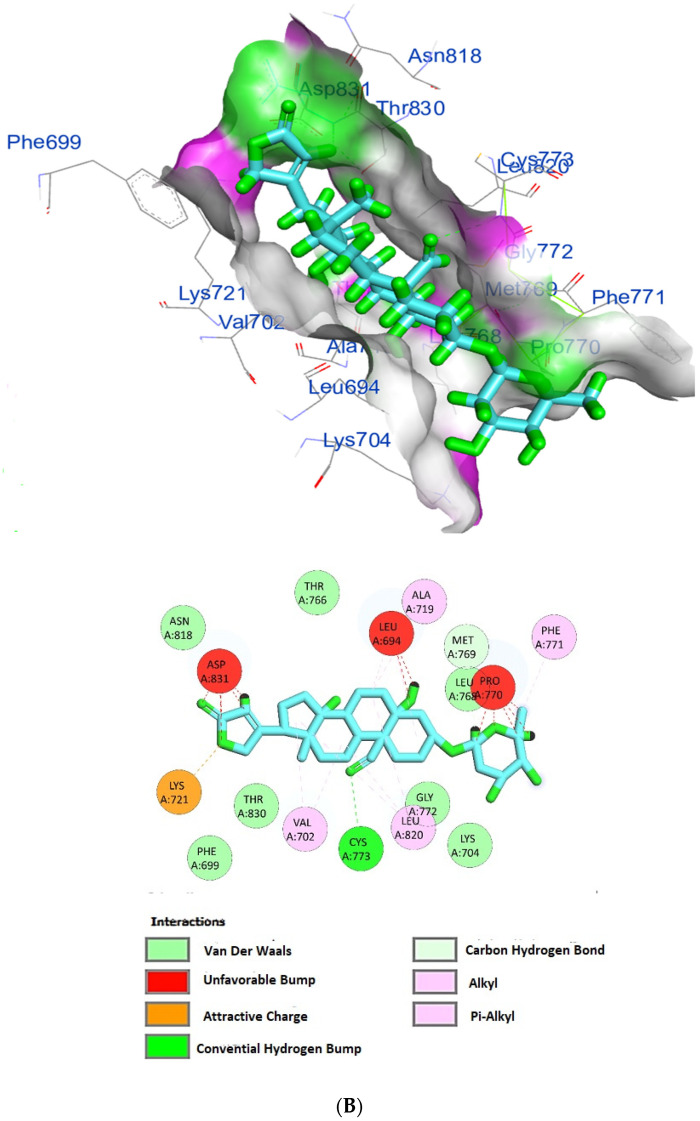

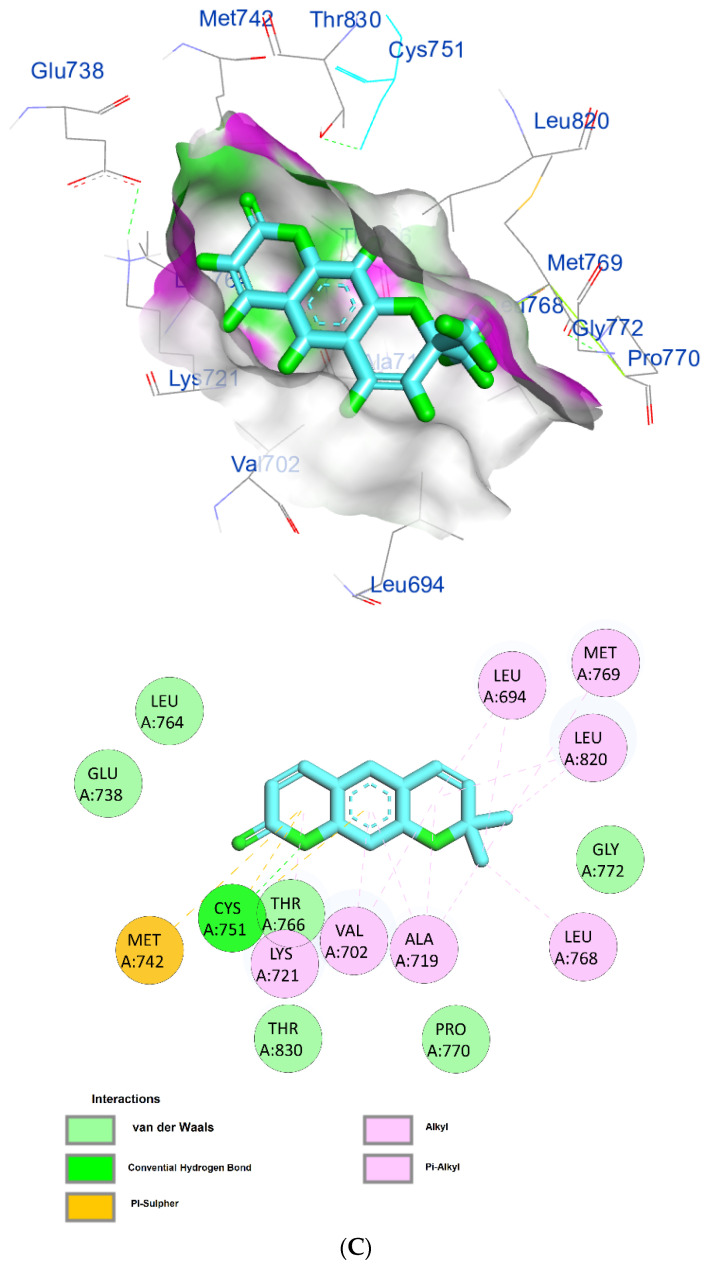

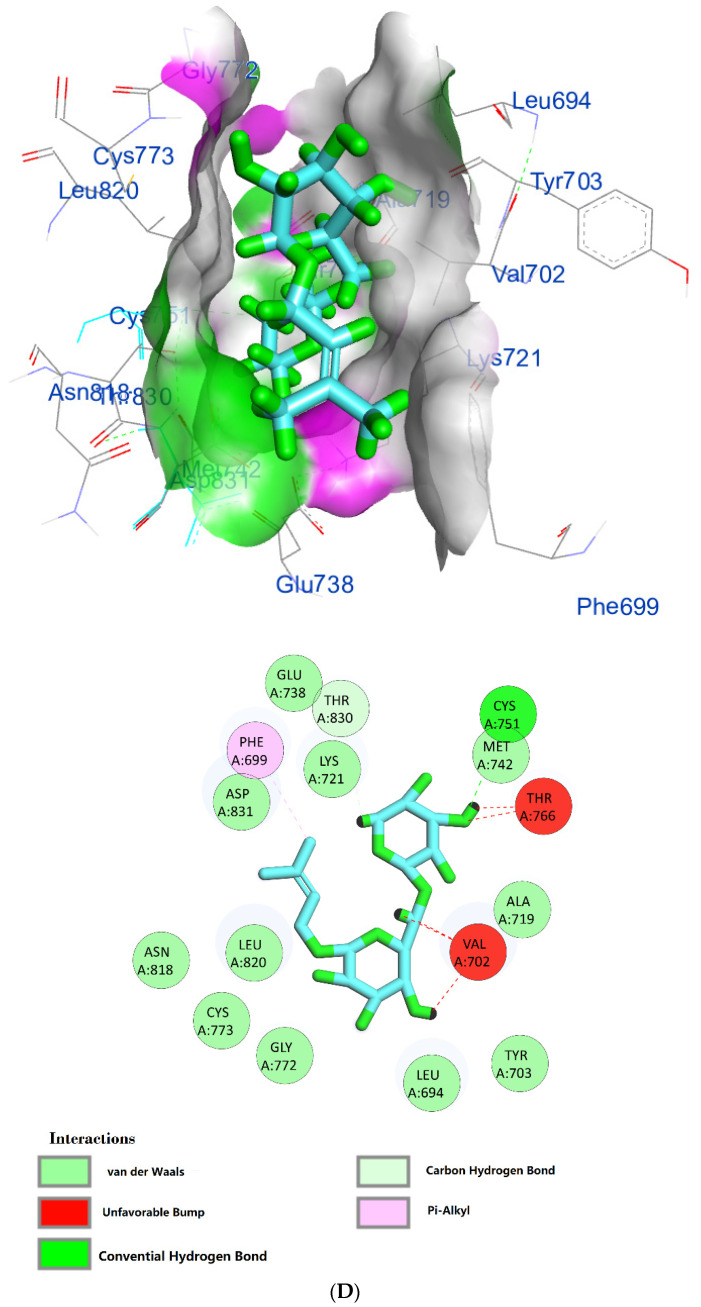

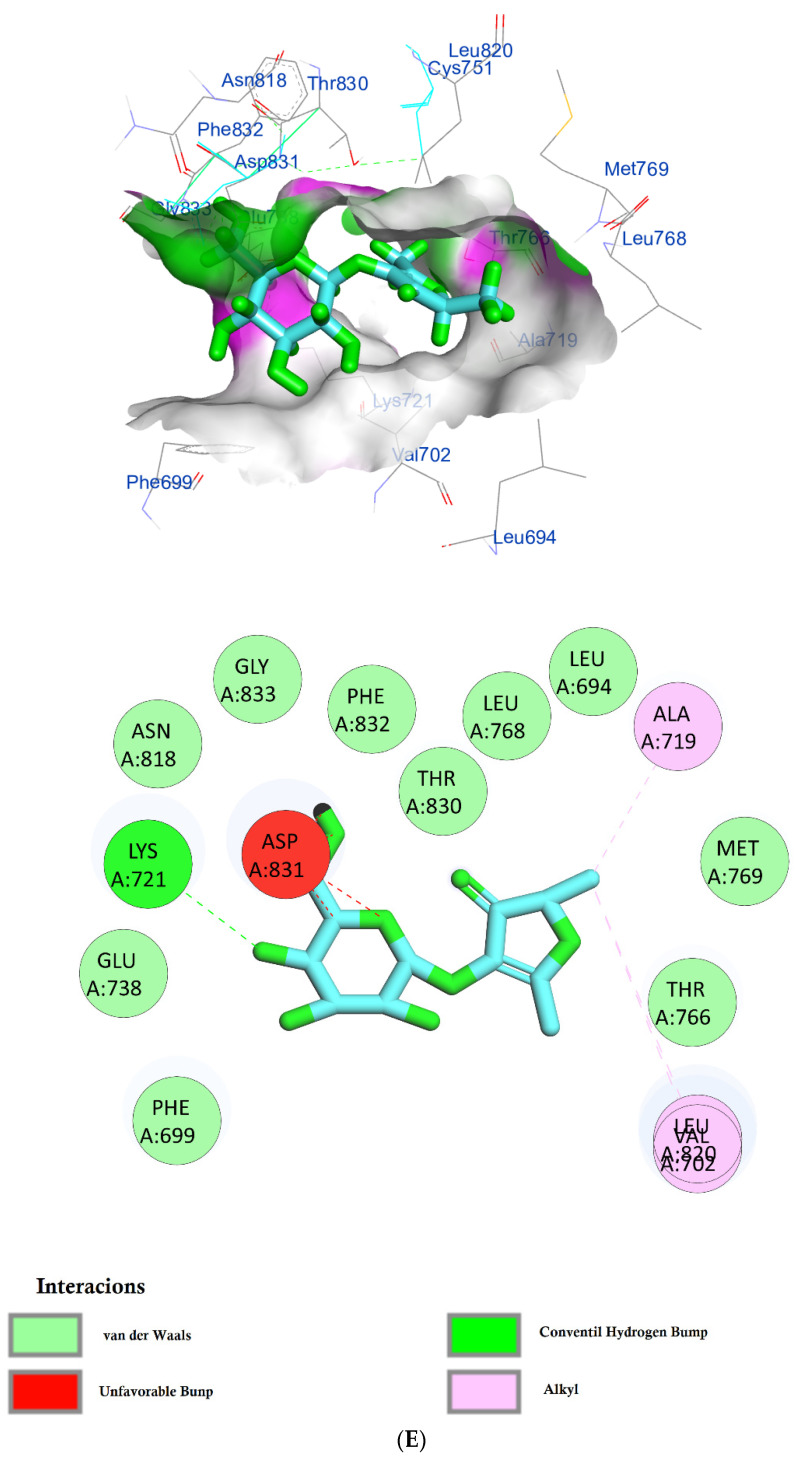

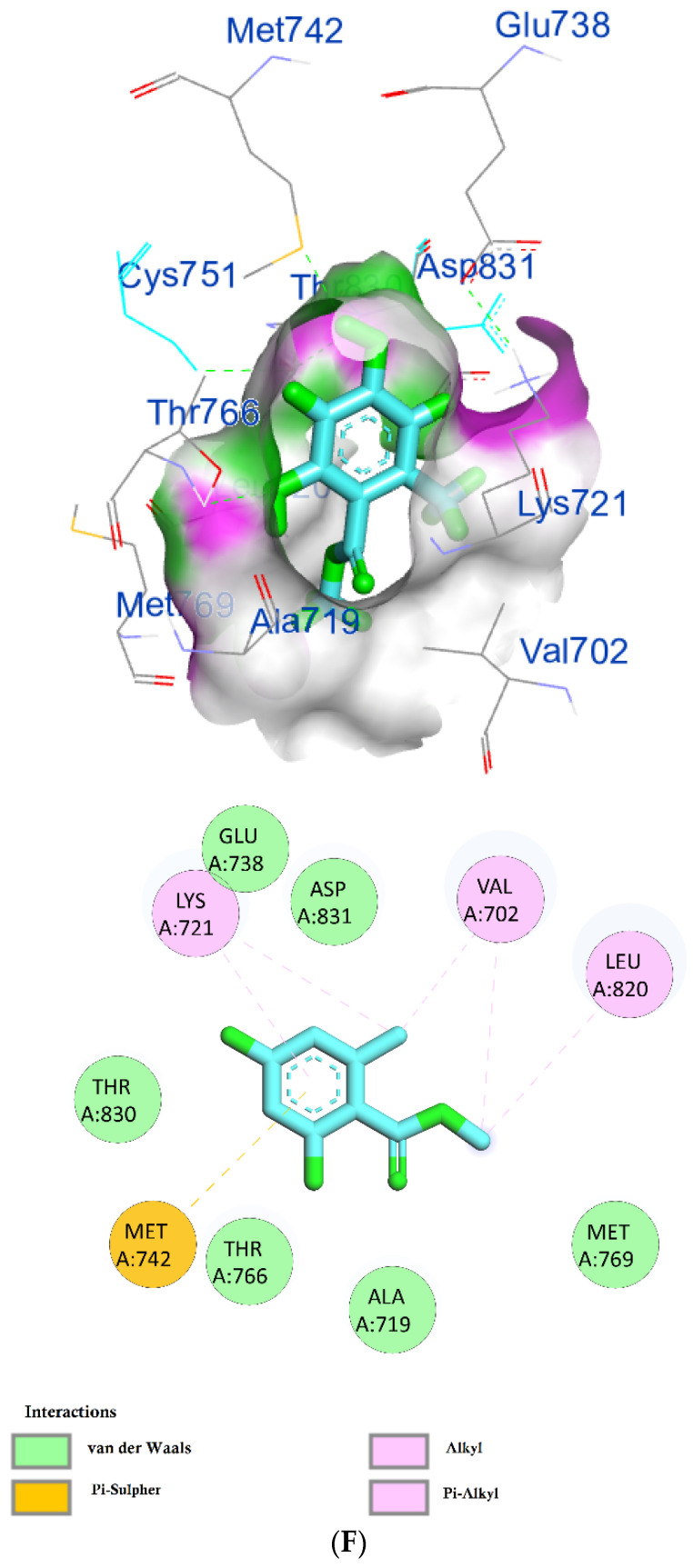

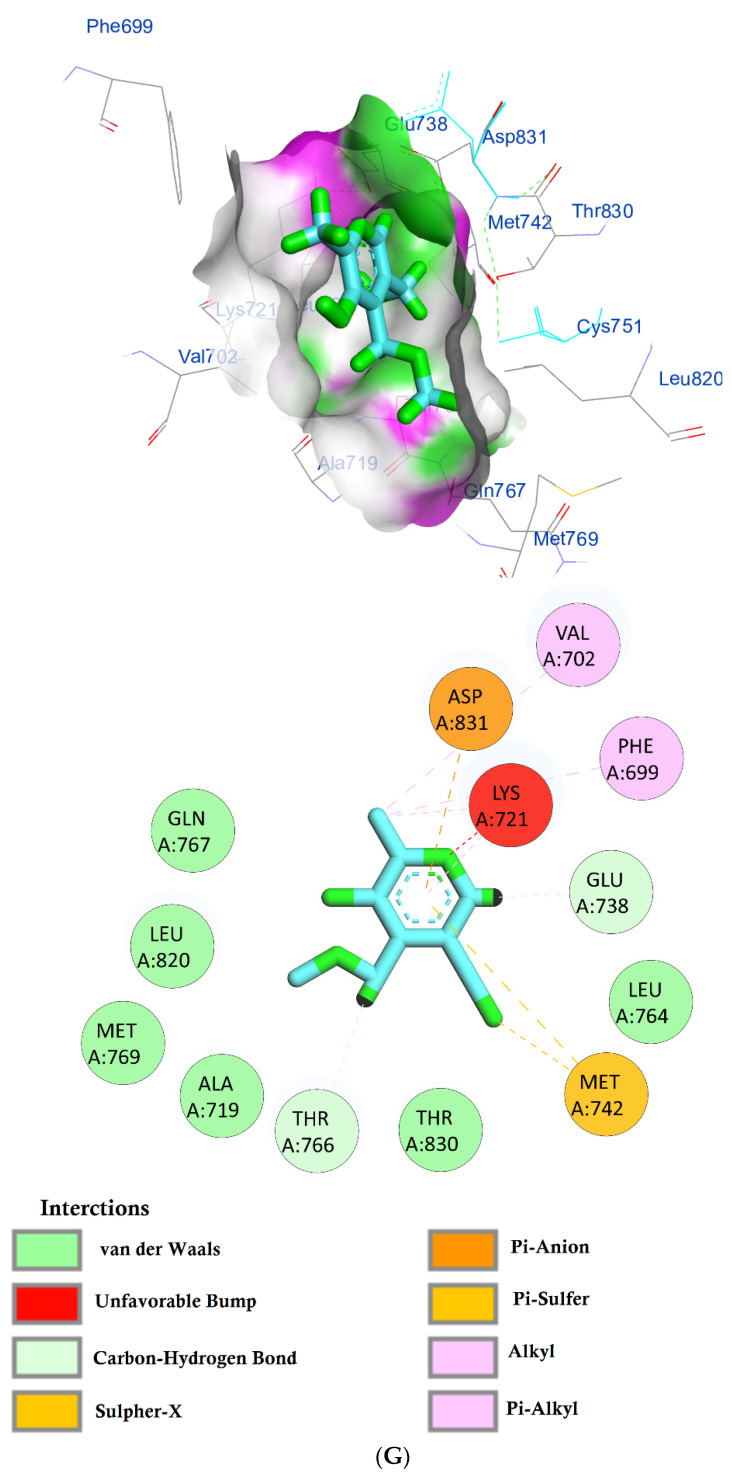
(**A**) Protein-ligand interactions of identified bioactive metabolites of *P. tomentosa* L. latex with the active site of EGFRK. Diagrams representing 3D and 2D protein–ligand interactions of ouabagenin with the active site of EGFRK. (**B**) Protein–ligand interactions of identified bioactive metabolites of *P. tomentosa* L. latex with the active site of EGFRK. Diagrams representing 3D and 2D protein–ligand interactions of corchoroside A. with the active site of EGFRK. (**C**) Protein–ligand interactions of identified bioactive metabolites of *P. tomentosa* L. latex with the active site of EGFRK. Diagrams representing 3D and 2D protein–ligand interactions of xanthyletine with the active site of EGFRK. (**D**) Protein–ligand interactions of identified bioactive metabolites of *P. tomentosa* L. latex with the active site of EGFRK. Diagrams representing 3D and 2D protein–ligand interactions of prenyl arabinosyl-(1->6)-glucoside with the active site of EGFRK. (**E**) Protein–ligand interactions of identified bioactive metabolites of *P. tomentosa* L. latex with the active site of EGFRK. Diagrams representing 3D and 2D protein–ligand interactions of furaneol 4-glucoside with the active site of EGFRK. (**F**) Proteinx–ligand interactions of identified bioactive metabolites of *P. tomentosa* L. latex with the active site of EGFRK. Diagrams representing 3D and 2D protein–ligand interactions of methyl orsellinate with the active site of EGFRK. (**G**) Protein–ligand interactions of identified bioactive metabolites of *P. tomentosa* L. latex with the active site of EGFRK. Diagrams representing 3D and 2D protein–ligand interactions of ginkgotoxin with the active site of EGFRK.

The protein epidermal growth factor receptor tyrosine kinase domain (EGFRK) was selected for this study. The ability to inhibit this receptor ultimately leads to the blockade of the growth pathway, thus serving as a promising anticancer agent [17]. The lower binding energy resulting from the association of the compound with the targeted protein is an indication of a higher binding efficiency. Erlotinib was used in this study as an EGFRK inhibitor. By comparing the binding affinity of different screened metabolites with erlotinib (ΔG of −6.8), it was found that ouabagenin showed the best binding affinity with a ΔG of −8.6 and displayed a better interaction profile than erlotinib. Erlotinib is completely trapped with EGFRK by strong hydrogen bonding generated by CYS773, THR766, MET769, and LYS721 with a distance between 1.46 and 2.76 A as well as a hydrophobic interaction with GLU736, GLU772, ALA719, PHE771 and LEU820 with distances between 2.63 and 4.13 A. Corchoroside A, with a ΔG of −8.1, is connected with EGFRK’s active sites at CYS773, LYS704, GLY772, PRO770, LEU694, MET769, and ASP831 with a strong hydrogen bond with distances varying from 2.24 to 3.9 A. Xanthyletine had a ΔG of −7.8, and it was connected with EGFRK’s active sites at the hydrophobic bonds at LEU694, MET769, LEU820, LEU768, ALA719, VAL702, LYS721, and MET742 with distances varying between 3.76 and 5.42. Prenyl arabinosyl-(1->6)-glucoside had a ΔG of −7.3, and the compounds showed a possible interaction with EGFRK’s active sites at CYS751 and THR830 with a strong hydrogen bond and connected with VAL702, THR766, and PHE699 with a hydrophobic interaction bond. Furaneol 4-glucoside had a ΔG of −7.1, and the compound showed a better interaction than erlotinib and was connected with LYS721 and ASP831 residues with a strong hydrogen bond and with ALA719 and VAL702 with hydrophobic bonds. Methyl orsellinate and ginkgotoxin showed a moderate binding activity, with a ΔG of −5.9 and 5.4 at EGFRK active sites, respectively. These compounds are connected with less bonding affinity with pi and sulfur bonds to EGFRK (Table 4, Figure 6).

#### 2.6.2. Pharmacokinetics of the Screened Secondary Metabolites

**Figure 7:**
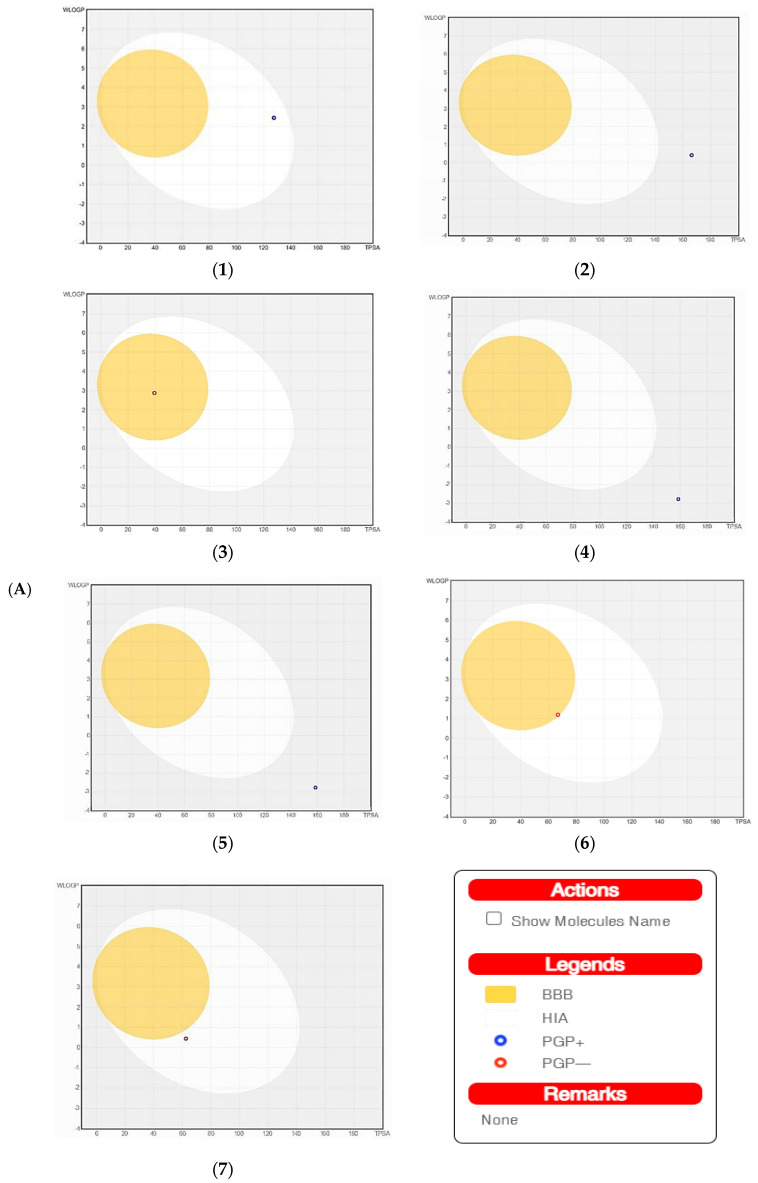

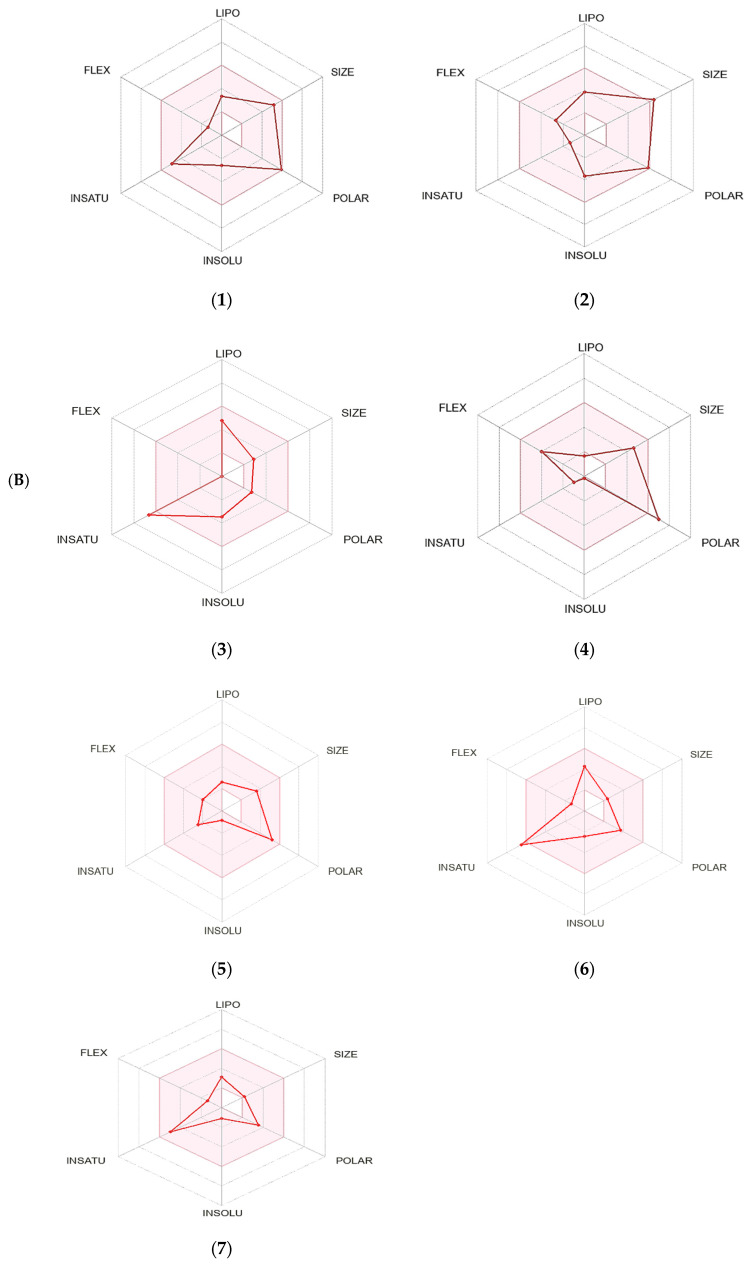
A boiled egg diagram (**A**) and bioavailability radar (**B**) for compounds 1: ouabagenin; 2: corchoroside A; 3: xanthyletine; 4: prenyl arabinosyl-(1->6)-glucoside; 5: furaneol 4-glucoside; 6: methyl orsellinate; and 7: ginkgotoxin. A boiled egg diagram represents the passive gastrointestinal absorption (HIA) and brain penetration (BBB) in the function of the position of the molecules in the WLOGP-versus-TPSA referential. The white region is for a high probability of passive absorption by the gastrointestinal tract, and the yellow region (yolk) is for a high probability of brain penetration. Yolk and white areas are not mutually exclusive. In addition, the points are colored in blue if they are predicted as actively effluxed by P-gp (PGP+) and in red if they are predicted as a non-substrate of P-gp (PGP−). The bioavailability radar represents the drug-likeness of a molecule, with the pink area representing the optimal range for each property (lipophilicity: XLOGP3 between −0.7 and +5.0; size: MW between 150 and 500 g/mol; polarity: TPSA between 20 and 130 Å2; solubility: log S not higher than 6; saturation: fraction of carbons in the sp3 hybridization that is no less than 0.25; and flexibility: no more than nine rotatable bonds for compounds).

The SwissADME software was used to estimate the absorption behavior of the tested compounds [18]. The results revealed that ouabagenin, corchoroside A, xanthyletine, furaneol 4-glucoside, methyl orsellinate, and ginkgotoxin showed high gastrointestinal absorption, while prenyl arabinosyl-(1->6)-glucoside showed low absorption. All the screened compounds cannot penetrate the blood–brain barrier (BBB) except for xanthyletine and methyl orsellinate (Figure 7A). These data are confirmed by the description of the P-glycoprotein (P-gp) substrate, where xanthyletine and methyl orsellinate are positive substrates for P-glycoprotein, that could inhibit the efflux transporters, which could explain their ability to pass the blood–brain barrier. On the other hand, the other compounds are negative P-gp, which could not inhibit the efflux transporter, which could be attributed to the inability of these compounds to pass the BBB. This behavior is reversed with ginkgotoxin, which is a negative P-gp substrate and did not pass the BBB. Except for ouabagenin and xanthyletine, none of the other compounds are CYP450 inhibitors, and they could not illicit drug–drug interactions or induce drug toxicity through the metabolism. Ouabagenin is described as a CYP2C9 inhibitor and xanthyletine is an inhibitor of CYP1A2 and CYP2C19. The results of the permeability test of the screened compound were determined through skin permeation, indicating that these compounds are not permeable through the skin (Table 5, Figure 7A).

The SwissADME software used drug-likeness rules to guarantee that selected compounds are a new drug; these rules are Lipinski (Pfizer^®^), Ghose (Amgen^®^), Veber (GSK^®^), Egan (Pharmacia^®^), and Muegge (Bayer^®^). These rules are discovered a new drug according to analysis of some physiochemical key. Results revealed that ouabagenin and xanthyletine met the complete criteria of all the above-mentioned rules. Corchoroside A and furaneol 4-glucoside obey all drug-likeness rules except Ghose’s (Amgen^®^) rule, whereas methyl orsellinate and ginkgotoxin obey all drug-likeness rules except for Muegge’s (Bayer^®^) rule. Prenyl arabinosyl-(1->6)-glucoside met the requirement of Lipinski’s (Pfizer^®^) rule. All compounds showed a good bioavailability score of 0.55, which was demonstrated in the bioavailability radar (Figure 7B). Furthermore, the medicinal chemistry data showed that all compounds except for methyl orsellinate have no pan assay interference, whereas all compounds showed a problematic fragmentation at the metabolism stage except for furaneol 4-glucoside and ginkgotoxin. For the possibility of synthesizing the screened metabolites, the SwissADME software gives a score ranging from 1 to 10; a score of 1 denotes ease of synthesis, and a score of 10 denotes difficulty of synthesis. From the scores recorded in Table 5, the compounds can be ordered from the easiest to the most difficult as follows: methyl orsellinate, ginkgotoxin, xanthyletine, furaneol 4-glucoside, ouabagenin, prenyl arabinosyl-(1->6)-glucoside, and, finally, corchoroside A (Table 5).

#### 2.6.3. In Silico Toxicity Data

Table 6 depicts the toxicity data of the screened metabolites. The ADMETlab 2.0 software used the AMES toxicity test that described the potential carcinogenic effect of the compound [19], and according to the human ether-a-go-go-related gene (hERG) toxicity test, the blockage of the potassium channel of that gene led to cardiac toxicity [20]]. Furthermore, the software estimated the maximum tolerated dose (human) and the oral toxicity (LD50) for rats as well as the oral rat chronic toxicity (LOAEL), hepatotoxicity, and skin sensitization.

In addition to the toxicity model, toxicophoric rules were also estimated for identifying the ability of the compound to induce cancer by mutation (genotoxic carcinogenicity rule) or by any other mechanism rather than mutation (non-genotoxic carcinogenicity rule) [21]. The results revealed that none of the tested compounds showed a toxicity impact in any toxic model. In addition, the compounds have no genotoxic carcinogenicity effect, where the results of xanthyletine and prenyl arabinosyl-(1->6)-glucoside revealed that they could induce cancer via another mechanism other than mutation (Table 6).

## 3. Discussion

As reported previously, plant latex from *P. tomentosa* L. is used in folk medicine to treat many diseases and in modern therapy. It shows multiple biological impacts such as antimicrobial, antioxidant, and cytotoxic effects. In this study, the plant latex secretion from *P. tomentosa* L. was collected to study the potential cytotoxic effect on various human cancer cell lines, and the study was extended to identify the polyphenols in plant latex to reveal their potential role in the cytotoxic impact.

An in vitro cytotoxicity study was carried out on five different human cancer cell lines, and the tested compound showed potent cytotoxic activity against A-549 (human lung carcinoma) with IC_50_ = 3.8 μg/mL compared with using vinblastine as the standard, with IC_50_ = 7.12 μg/ml. Accordingly, the A-549 cell line was selected to study the potential mode of action of latex as a cytotoxic agent [22,23].

Cancer patients are characterized by the dysregulation of the normal cell cycle. The potential mechanism of cancer cells could be due to the activation of oncogenes (RAS and MYC protein) that lead to the overexpression of growth factors that stimulate the expression of cyclin-dependent kinase/cyclin D protein. This cascade of protein synthesis elicits the overgrowth of cells that bypass the checkpoint cell cycle, leading to cancer [22,23].

Tumor suppressor p53 is responsible for checking the integrity of DNA to maintain the normal growth of the cell or initiate apoptosis. An activation of tumor suppressor p53 transcription stimulates the expression of the p21 gene (a potent inhibitor of CDK/cyclin D), which ends with cell cycle arrest [24,25]. In normal cells, p53 regulates the activity of EGFR kinase, where the suppression of p53 is associated with EGFR inhibitor resistance. It leads to cytotoxic drug inactivation [26]. In the severe damage of DNA, the p53 gene stimulates the synthesis of apoptotic marker proteins and initiates programmed cell death. Conversely, in the cancer cell, p53 is suppressed and leads to the overgrowth of cells by passing the checkpoint, leading to cancer, and may lead to the resistance of the activity of chemotherapy through the induction of EGFR inhibition resistance. Considering the potential mechanism of cancer at the level of the cell cycle controller, this study reveals that plant latex at an IC_50_ of 3.8 μg/mL induced cell cycle arrest and increased the apoptotic cell % more than the necrotic cell % using a morphological Annexin V-FITC assay. These data are confirmed by a biochemical study using the Western blot technique, where current results revealed that plant latex upregulates the expression of the p53 suppressor gene, which could be attributed to the upregulation of apoptotic protein.

Programmed cell death is regulated through extrinsic and intrinsic factors. The extrinsic factors started from the activation of cysteine protease (caspase 9) as a result of p53 stimulation through cytochrome c [27]. Consequently, the cleavage of caspase 9 initiated the activation of caspase 3 to promote apoptosis. An intrinsic factor is the Bcl-2 protein, which modulates mitochondrial cell permeability under the external stimuli of apoptotic factors via the activation of Bax proapoptotic proteins [28,29]. The overexpression of the Bcl-2 oncogene with the inhibition of the Bax proapoptotic factor is associated with a good prognosis in many types of cancer [24,30,31]. In agreement with present results, it was demonstrated that plant latex at an IC_50_ of 3.8 μg/mL induced the overexpression of the tumor suppression oncogene p53 associated with the stimulation of the caspase 3 apoptotic protein that promotes apoptosis by increasing the permeability of the mitochondrial membrane for apoptotic factors via the suppression of Bcl-2 and the activation of the Bax protein.

The results obtained from the LC/MS-QTOF technique identified seven major compounds in plant latex: methyl orsellinate, ginkgotoxin, furaneol 4-glucoside, ouabagenin, prenyl arabinosyl-(1->6)-glucoside, corchoroside A and xanthyletine. Most of these compounds were identified as bioactive components from different plant extracts. Methyl orsellinate has antioxidant, antiproliferative, and antidiabetic activities [32,33,34]. Furaneol 4-glucoside is abundant in fruits with characteristic aroma and antimicrobial activity [35]. Ouabagenin is an aglycon precursor of ouabain, and it was first described as having no biological activity [36]; recently, ouabagenin was found to have antihypertensive activity and anticancer activity [37,38]. Corchoroside A is a cardiac glycoside that shows digoxin and digitoxin-like action which stimulate cardiac muscles [39]. Xanthyletine, extracted from the stem bark of Citrus reticulata, was found to have cytotoxic effects against different tumor cell lines [40].

The in silico study demonstrated that the identified compounds have a great affinity for EGFRK compared with erlotinib, which is a potent EGFRK inhibitor [41,42,43]. This could reveal a different array of the cytotoxic mechanism of plant latex by the inhibition of the epidermal growth factor (EGF) and initiate apoptosis. The pharmacokinetic study reveals the lipophilicity of the screened metabolite, which enhances its permeability through a cell to induce its cytotoxic effect. Furthermore, the screened compounds showed a good bioavailability score and were the easiest to synthesize.

Drug-likeness used a virtual analysis to discover a new drug according to several filter rules [43,44] based on the analysis of their physiochemical properties. FDA was approved a number of drugs that failed to obey some of these rules and recommended to use more than one rule to discover a new drug [45]. All screened compounds obeyed most of filter rules that could guarantee to be a new drug, where corchoroside A and furaneol 4-glucoside obeying all drug-likeness rules except Ghose’s (Amgen^®^) rule. Methyl orsellinate and ginkgotoxin obeyed all drug-likeness rules except Muegge’s (Bayer^®^) rule, and prenyl arabinosyl-(1->6)-glucoside met the requirement of Lipinski’s (Pfizer^®^) rule. The identified compounds of plant latex are safe, as shown from the in silico toxicity profile. Collectively, latex polyphenol compounds from *P. tomentosa* L. identified by LC/MS showed a promising antitumor, prodrug agent.

Due to the small quantity of plant extract, this study is limited to evaluate the potential efficacy of plant latex as apoptotic inducer or mediated via inducing cell cycle arrest on the most affected cell line lung cancer cell lines (A-549).

## 4. Materials and Methods

### 4.1. Chemicals

All the solvents in this study were obtained from Sigma-Aldrich, Fisher Scientific, Scharlau (Madrid, Spain) and VWR BDH Prolabor chemical (analytical grade). Roswell Park Memorial Institute 1640 Medium (RPMI-1640), Dulbecco’s modified Eagle’s medium, 4-(2-hydroxyethyl)-1-piperazineethanesulfonic acid buffer solution (HEPES), L-glutamine, gentamycin, and 0.25% trypsin-EDTA were purchased from Lonza (Bornem, Belgium).

### 4.2. Plant Material and Extraction

The fresh latex of the plant *P. tomentosa* L. was collected during spring seasons from the Hail region, with coordinates (27°15′41.6″ N, 41°13′43.3″ E), (27°3′30.4″ N, 42°9′15.9″ E), (27°58′43.8″ N, 42°15′42″ E), (27°55′9″ N, 42°15′48.8″ E), Saudi Arabia. The plant was identified, and the specimen was deposited in the Herbarium of the Department of Biology, UoH 18211, University of Hail. The crude, thick, white liquid was collected by striping and cutting the plant stem, and the secretion was collected in amber glass tubes and stored at +4 °C until used in biological assays.

A portion of stored plant latex (approximate 25.0 mL) used for polyphenol identification, it was dissolved in methanol followed by evaporation under reduced pressure by using a rotary evaporator vacuum at 40 °C, (model: W2-100 SENCO, rpm of 100; Shanghai SENCO Technology Co., Ltd., Shanghai, China).

### 4.3. In Vitro Biological Activity

#### 4.3.1. Cytotoxic Activity

The study used five human cancer cell lines to evaluate the cytotoxic activity of the plant latex. Cancer cell lines are HeLa cells (cervical carcinoma cells), A-549 (lung carcinoma), Panc-1 (pancreatic carcinoma cells), MDA-MB-231 (metastatic mammary adenocarcinoma), and MRC-5 (lung fibroblast cell line) cells. Cell lines were obtained from the American Type Culture Collection (ATCC, Rockville, MD, USA).

##### Cell Line Propagation

A-549 and MDA-MB-231 cells were cultivated in Dulbecco’s modified Eagle’s medium supplemented with 10% heat-inactivated fetal bovine serum. Panc-1, HeLa, and MCR-5 cells were cultivated in RPMI 1640 medium supplemented with 10% FBS. Cells were maintained in a 5% CO_2_ incubator at 37 °C and passaged one to two times per week [46,47,48,49,50].

##### Evaluation of the MTT Assay for Cytotoxic Activity

Corning^®^ 96-well tissue culture plates were seeded at a density of 5 × 10^4^ cells/well, and the cells attached for 24 h at 37 °C. A triplicate serial dilution of plant latex residue and 0.25–500 μg/100 µL cell culture media were transferred to the cell culture plates containing the cells. After 72 h of incubation, the media were removed and 100 µL fresh culture of the RPMI 1640 medium containing 10 µL of the 12 mM MTT was added to each well. The 96-well plate was incubated at 37 °C and 5% CO_2_ for 4 h. An 85 µL aliquot of the media was removed from the wells, and 50 µL of DMSO was added to each well, mixed, and incubated at 37 °C for 10 min. The optical density was measured at 590 nm with the microplate reader (Sunrise, Tecan, Inc., Männedorf, Switzerland) to determine the number of viable cells [51]. The percentage of viability was calculated as follows:

[(ODt/ODc)] × 100%

where ODt is the optical density of the test samples, ODc is the optical density of the control.

The most affected cell line with its respective half-maximal inhibitory concentration (IC_50_) of the plant latex was selected to elucidate the possible mode of action of the plant latex.

#### 4.3.2. Cell Cycle Analysis by Flow Cytometry

The CycleTEST™ PLUS DNA Reagent Kit (Becton Dickinson BD Pharmingen^TM^, Heidelberg, Germany) was used to determine the effect of the plant latex treatment on the most affected cell line from the MTT assay. The treated or non-treated cell lines were stained with propodium iodide stain according to the manufacturer’s instructions. The samples were acquired automatically using the Loader with acquisition criteria of 10,000 events for each tube. A cytometer was used for reading the test experiment. Cell-cycle distribution was calculated using Cell Quest software (Becton Dickinson Immunocytometry Systems, San Jose, CA, USA) [52].

#### 4.3.3. Apoptosis Analysis (Annexin V-FITC Assay)

Apoptotic cells were analyzed by an Annexin V-FITC assay. Briefly, selected cell lines from MTT assay were cultured to a confluent monolayer then treated with its respective IC_50_ of plant latex. After treatment for 72 h, the cell lines were then harvested and rinsed twice in PBS (20 min) followed by a binding buffer. Moreover, cells were re-suspended in 100 µL of the kit binding buffer with the addition of 1 µL of Annexin V-FITC (Becton Dickinson BD Pharmingen^TM^, Heidelberg, Germany), followed by incubation for 40 min. at 4 °C. Cells were then washed and re-suspended in 150 µL of binding buffer with the addition of 1 µL of DAPI (1 µg/mL in PBS) (Invitrogen, Life Technologies, Darmstadt, Germany). The samples were acquired automatically using the Loader with acquisition criteria of 10,000 events for each tube. Then, the cells were analyzed using the flow cytometer BD FACS Calibur (BD Biosciences, San Jose, CA, USA) [53].

#### 4.3.4. Evaluation of the Apoptotic Markers of Cytotoxicity

The immunoblot analysis of proteins (Western blot) technique was used to determine the apoptotic activity of the IC_50_ of plant latex against the cell line. Briefly, the total protein with volume of 20 µg was collected from treated and untreated cell lines for denaturation and electrophoretic separation, and the separated protein was transferred to a polyvinylidene fluoride (PVDF) membrane. The primary antibodies against Bax, Bcl-2, p53, and caspase-3 (The primary antibodies against BAX (1:1000, Cell Signaling Technology, Danvers, MA, USA), Bcl-2 (1:1500, Cell Signaling Technology), cleaved caspase-3 (1:500, Cell Signaling Technology), p53 (1:1000, Abcam, Cambridge, England), and β-actin (1:5000, Sigma, Madrid, Spain) were loaded to the gel membrane and incubated to form a protein–antibody complex at 4 °C. The membrane was washed and reintubated with a secondary antibody which bounded with enzyme to produce luminescence. The amount of luminescence is directly proportional to the amount of the protein that reacted with the antibody. The amount of luminescence was captured by a BioRad Imager [54,55].

### 4.4. Screening of the Secondary Metabolite by LC-Plant Latex

An LC/MS 6530 Q-TOF (Agilent Technologies, Santa Clara, CA, USA) equipped with an autosampler (G7129A), a quaternary pump (G7104C), and a column compartment (G7116A) was used for the chromatographic separation of polyphenols in the plant latex. The residue was dissolved in methanol, and the injection volume was 6 μL. The analytes were separated on a Zorbax RP-18 column (dimensions: 150 mm; I.D: 3 mm; dp: 2.7 μm), and the flow rate was 0.3 ml/min. Mass spectra were acquired using ESI in (+) ionization modes with a capillary voltage of 4500 V. The mass spectra were recorded in the *m*/*z* range of 50 to 3000 *m*/*z*. The gas temperature and drying gas flow were 200 °C and 8 L/min, respectively. The skimmer and fragmentator voltages were set at 65 and 130 V, respectively, and the collision energy was 10 V. The nebulization pressure was 58 psi. The separation was conducted via gradient elution, using mobile phase A (water, 0.1% formic acid) and Solvent B (acetonitrile, 0.1% formic acid). The elution profile is described in Table 7 [56]. MassHunter Workstation Software with Bioconfirm Software, Version B.07.01, integrated with LC/MS personal compound database libraries (PCDLs) were used for extraction and identification of polyphenols (see Supplementary Materials).

### 4.5. In Silico Study

#### 4.5.1. Molecular Docking

Molecular docking assessment were performed by the molecular docking software Autodock 4.2.6. [57,58,59]. The 3D structures of identified compounds such as methyl orsellinate, ginkgotoxin, furaneol 4-glucoside, ouabagenin, prenyl arabinosyl-(1->6)-glucoside, corchoroside A, and xanthyletine were obtained in SDF format from organic structure databases such as the ZINC database [60] and the PubChem database (https://pubchem.ncbi.nlm.nih.gov, accessed on 2 February 2022). These molecules were converted into PDB format by the Open Babel program [61]. The crystal structure from the epidermal growth factor receptor tyrosine kinase domain (EGFRK) was downloaded from RCSB PDB (PDB: 1M17.pdb; resolution: 2.60 Å) to find the potential hit molecules for further drug discovery experiments. AutoDock uses a Lamarckian genetic algorithm (LGA) and is based on a semi-empirical free energy force field. An auto-docking grid was used for the preparation of the grid map using a grid box, with the size set to (X: 40; Y: 40; Z: 58), the grid spacing set to 0.511 Å, and the grid center set to (X: 28.557; Y: 5.994; Z: 57.099). Grid spacing was kept at 0.58 Å for all the receptors. The validation step confirmed the suitability of the used protocol for this docking study as demonstrated by the root mean square deviation (RMSD). The generated docked conformation was ranked by predicting the docked conformation with the top-most binding energy compared with the binding affinity of erlotinib (a potent EGFRK inhibitor). The binding energy was analyzed using UCSF Chimera for the intermolecular hydrogen bonding of amino acid residues at active sites from the receptors with docked ligands [57,62].

#### 4.5.2. In Silico ADME Assessment

The physicochemical and pharmacokinetic properties of the identified compounds were estimated using the SwissADME server [18].

#### 4.5.3. In Silico Toxicity Assessment

The in silico toxicity study of the identified compounds was estimated by ADMETlab 2.0 software [63].

### 4.6. Statistical Analysis

In vitro data were represented as the mean ± standard deviation (SD), and all the data were replicated three times (*n* = 3); the results of the half-maximal inhibitory concentration (IC_50_) were estimated from the dose–cell viability response curve using non-linear regression (curve fit) followed by the inhibitor vs. response–variable slope (four parameters) test. The statistical difference between groups was analyzed using one-way ANOVA, with a post hoc Tukey test. Differences were considered statistically significant at *p* < 0.05. The data analyses were conducted using GraphPad Prism 7 (GraphPad Software, San Diego CA, USA).

## 5. Conclusions

Plant latex collected from *P. tomentosa* L. constitutes valuable polyphenols such as methyl orsellinate, ginkgotoxin, furaneol 4-glucoside, ouabagenin, prenyl arabinosyl-(1->6)-glucoside, corchoroside A, and xanthyletine. The in vitro cytotoxic study revealed the efficacy of plant latex in inhibiting tumor cell growth and inducing apoptosis in the A-549 cell line. The results of the in silico study demonstrated an elucidated mechanism whereby the identified metabolites were cytotoxic agents. A total of five identified metabolites from the seven selected metabolites showed a great binding affinity to EGFRK compared with the positive control. All selected metabolites (methyl orsellinate, ginkgotoxin, furaneol 4-glucoside, ouabagenin, prenyl arabinosyl-(1->6)-glucoside, corchoroside A, and xanthyletine) obeyed Lipinski’s rule, with no adverse toxic impact. The current study elucidated the possible mechanism of latex from *P. tomentosa* L. as a cytotoxic agent and projected identified compounds from a new natural source as promising antitumor pharmaceutical agents. Preclinical study will be submitted on an identified isolated compound form of *P. tomentosa* L.

## Figures and Tables

**Table 1:** Effect of the plant latex of *P. tomentosa* L. compared to the control treatments on the phases of the cell cycle of the lung cancer cell line (A-549) ^a^.

Sample Code	Concentration (µg/mL)	%Pre G1	%G1	%S	%G2/M
Plant Latex *P. tomentosa* L. (Treated cells)	3.89	23.61 ^b,^***	39.79 ^b,^*	53.75 ^b,^**	6.46 ^b,^***
A-549 cells (control)	0	1.88	46.81	39.18	14.01

^a^ Data are presented as the mean ± SD. *** *p* < 0.0001; **p < 0.001; * *p* < 0.05. ^b^ Versus control.

**Table 2:** The effect of the IC_50_ of plant latex of *P. tomentosa* L. on the percentages of apoptotic and necrotic cells of the A-549 cell line ^a^.

Sample	Tested Conc. (µg/mL)	Early Apoptosis	Late Apoptosis	Necrosis
Plant- Latex *P. tomentosa* L. (Treated cells)	3.89	3.13 ^b,^**	12.29 ^b,^***	8.19 ^b,^**
A-549 cells (control)	0	0.36	0.25	1.27

^a^ Data are presented as mean ± SD. *** *p* < 0.0001; ** *p* < 0.001. ^b^ Versus control.

**Table 3:** The effect of the IC_50_ of the plant latex of *P. tomentosa* L. on the expression of the apoptotic protein expression of Bcl-2, Bax, caspase-3, and p53-mediated apoptosis in lung cancer cell lines (A-549) ^a^.

Samples	Protein Expression (Normalized to β-Actin) *				
	BAX	Bcl-2	BAX/Bcl-2 Ratio	Caspases-3	p53
Control (A-549 cells, nontreated)	1.00	1.00	1.00	1.00	1.00
Plant latex of *P. tomentosa* L. (3.89 µg/mL)	2.13 ^b,^*	0.41 ^b,^**	5.19 ^b,^***	1.69 ^b,^*	3.94 ^b,^**

^a^ Data are presented as mean ± SD. *** *p* < 0.0001; ** *p* < 0.001. ^b^ Versus control. * All the data are normalized to β-actin, and the values are given as fold changes from the control, which is set to ‘1’.

**Table 4:** Glide docking score and interacting residues in the molecular docking analysis of identified bioactive metabolites with EGFRK protein.

Phytochemical Compounds	Glide Docking Score ΔG Kcal/mol	Receptor–Ligand Interaction	Distance Å
Ouabagenin	−8.6	(CYS773)—(Ligand) C–H interaction	2.76
		(VAL702)—(Ligand) Alkyl interaction	3.88
		(LEU820)—(Ligand)Alkyl interaction	2.51
		(THR766)—(Ligand) C–H interaction	2.01
		(GLU736)—(Ligand) Hydrophobic interaction	2.63
		(GLU772)—(Ligand) Hydrophobic interaction	1.46
		(ALA719)—(Ligand) Hydrophobic interaction	3.27
		(MET769)—(Ligand) H-bond interaction	2.48
		(LYS721)—(Ligand) H-bond interaction and carboxylate salt bridge interactions	2.60 4.13
Corchoroside A	−8.1	(CYS773)—(Ligand) H-bond interaction	2.44
		(LYS704)—(Ligand) H-bond interaction	2.09
		(GLY772)—(Ligand) H-bond interaction	3.51
		(PRO770)—(Ligand) H-bond interaction	3.06
		(LEU694)—(Ligand) H-bond interaction	3.78
		(ASP831)—(Ligand) H-bond interaction	3.90
		(ALA719)—(Ligand) Alkyl interaction and pi–alkyl hydrophobic interaction	3.55
		(PHE771)—(Ligand) Alkyl interaction and pi–alkyl hydrophobic interaction	4.20
		(LEU820)—(Ligand) Alkyl interaction and pi–alkyl hydrophobic interaction	3.42
		(VAL702)—(Ligand) Alkyl interaction and pi–alkyl hydrophobic interaction	3.85
		(MET769)—(Ligand) C–H bond interaction	2.77
		(LYS721)—(Ligand) Carboxylate salt bridge interaction	3.45
Xanthyletine	−7.8	(LEU694)—(Ligand) Alkyl and pi–alkyl hydrophobic interaction	5.42 5.37
		(MET769)—(Ligand) Alkyl and pi–alkyl hydrophobic interaction	5.36
		(LEU820)—(Ligand) Alkyl and pi–alkyl hydrophobic interaction	3.89 5.28
		(LEU768)—(Ligand) Alkyl and pi–alkyl hydrophobic interaction	4.19
		(ALA719)—(Ligand) Alkyl and pi–alkyl hydrophobic interaction	3.95 3.76 4.04
		(VAL702)—(Ligand) Alkyl and pi–alkyl hydrophobic interaction	4.54 4.61
		(LYS721)—(Ligand) Alkyl and pi–alkyl hydrophobic interaction	4.31
		(MET742)—(Ligand) Alkyl and pi–alkyl hydrophobic interaction	5.40
Prenyl arabinosyl-(1->6)-glucoside	−7.3	(CYS751)—(Ligand) H-bond interaction	3.17
		(VAL702)—(Ligand) Hydrophobic interaction	1.42 1.62
		(THR766)—(Ligand) Hydrophobic interaction	2.07 2.15
		(PHE699)—(Ligand) Pi–alkyl hydrophobic interaction	3.02
		(THR830)—(Ligand) C–H bond interaction	3.03
Furaneol 4-glucoside	−7.1	(LYS721)—(Ligand) Conventional hydrogen bond and carboxylate salt bridge interactions	2.36 4.55
		(ASP831)—(Ligand) 4 H-bonds and hydrophobic interactions	3.10, 1.90, 1.75, 2.57 and 2.17
		(ALA719)—(Ligand) Hydrophobic interaction	3.12
		(VAL702)—(Ligand) Alkyl hydrophobic interaction	5.30
Erlotinib	−6.8	(GLU767) & (THR830)—(Ligand) Carbon hydrogen bond interaction	3.15 4.07
		(MET769)—(Ligand) Conventional hydrogen bond interaction	
		(ALA719)—(Ligand) Alkyl and pi–alkyl hydrophobic interaction	2.70
		(LEU764)—(Ligand) Alkyl and pi–alkyl hydrophobic interaction	3.74 5.35
		(LYS721)—(Ligand) Alkyl and pi–alkyl hydrophobic interaction	4.21
		(LEU694)—(Ligand) Pi–sigma interaction	4.14 4.81
		(LEU920)—(Ligand) Pi–sigma interaction	3.68
		(MET742)—(Ligand) Pi–sulfur interaction	3.86 5.31
Methyl orsellinate	−5.9	(LYS721)—(Ligand) Alkyl and pi–alkyl interactions	5.50
		(VAL702)—(Ligand) Alkyl and pi–alkyl interactions	4.60 4.41
		(LEU820)—(Ligand) Pi–alkyl interaction	4.10 4.87
		(ASP831)—(Ligand) Pi–anion interaction	4.57
Ginkgotoxin	−5.4	(MET742)—(Ligand) Pi–sulfur and pi–anion interaction	4.02
		(GLU738)—(Ligand) Pi–sulfur interaction	6.00 3.25
		(PHE699)—(Ligand) Pi–alkyl interaction	1.97
		(VAL702)—(Ligand) Alkyl interaction	4.90
		(LYS721)—(Ligand) Unfavorable bump and pi–alkyl and alkyl interactions	4.84
		(THR766)—(Ligand) Carbon–hydrogen bond interaction	2.15 1.13 4.11

**Table 5:** Pharmacokinetics, drug-likeness and medicinal chemistry of latex from *P. tomentosa* L.’s screened secondary metabolites according to SwissADME software.

Pharmacokinetics	Ouabagenin	Corchoroside A	Xanthyletine	Prenyl Arabinosyl-(1->6)-Glucoside	Furaneol 4-Glucoside	Methyl Orsellinate	Ginkgotoxin
GI absorption	High	High	High	Low	High	High	High
BBB permeant	No	No	Yes	No	No	Yes	No
P-gp substrate	Yes	Yes	No	Yes	Yes	No	No
CYP1A2 inhibitor	No	No	Yes	No	No	No	No
CYP2C19 inhibitor	No	No	Yes	No	No	No	No
CYP2C9 inhibitor	Yes	No	No	No	No	No	No
CYP2D6 inhibitor	No	No	No	No	No	No	No
CYP3A4 inhibitor	No	No	No	No	No	No	No
Log Kp (cm/s)	−8.62	−8.64	−5.68	−10.49	−8.72	−6.4	−7.35
Drug-likeness							
Lipinski	yes	yes	yes	Yes	yes	yes	yes
Ghose	yes	No	yes	No	No	Yes	Yes
Veber	yes	Yes	yes	No	Yes	Yes	Yes
Egan	yes	Yes	yes	No	Yes	Yes	Yes
Muegge	yes	Yes	yes	No	Yes	No	No
Bioavailability score	0.55	0.55	0.55	0.55	0.55	0.55	0.55
Medicinal chemistry							
PAINS	0	0	0	0	0	1	0
Brenk	1	2	1	1	0	1	0
Lead-likeness	No	No	No	No	Yes	No	No
Synthetic accessibility	5.64	7.24	3.24	5.77	5.23	1.55	1.78

**Table 6:** Toxicity data for the screened metabolites using ADMETlab 2.0 software.

Toxicity Model	Ouabagenin	Corchoroside A	Xanthyletine	Prenyl arabinosyl-(1->6)-Glucoside	Furaneol 4-Glucoside	Methyl Orsellinate	Ginkgotoxin
AMES toxicity	No	No	No	No	No	Yes	No
Max. tolerated dose (human) (log mg/kg/day)	0.276	−1.229	−0.001	1.32	1.414	0.983	1.059
hERG I inhibitor	No	No	No	No	No	No	No
hERG II inhibitor	No	No	No	No	No	No	No
Hepatotoxicity	No	No	No	No	No	No	No
Skin sensitization	No	No	No	No	No	No	No
T. Pyriformis toxicity (log ug/L)	0.285	0.285	0.871	0.29	0.285	0.585	−0.258
Minnow toxicity (log mM)	4.323	3.312	0.997	7.58	5.153	2.16	2.65
Oral rat acute toxicity (LD50) (mol/kg)	2.802	2.854	2.307	1.95	1.983	1.731	1.963
Oral rat chronic toxicity (LOAEL) (log mg/kg_bw/day)	2.928	2.251	1.866	3.16	3.518	2.137	2.148
Toxicophore rules							
Genotoxic carcinogenicity rule	NO	NO	NO	NO	NO	NO	NO
Non-genotoxic carcinogenicity rule	NO	NO	yes	yes	NO	NO	NO

**Table 7:** Elution profile used in polyphenols identification.

Time	Solvent A (Water 0.1% Formic Acid)	Solvent B (Acetonitrile 0.1% Formic Acid)
0	98	2
2	98	2
15	90	10
35	80	20
60	50	50
80	30	70
100	0	100

## Data Availability

Data sharing not applicable.

## Supplementary Materials

The following supporting information can be downloaded at: https://www.mdpi.com/article/10.3390/ph15091132/s1.
