# Supplementary material for: In Silico Pharmacokinetic Profiling of the Identified Bioactive Metabolites of Pergularia tomentosa L. Latex Extract and In Vitro Cytotoxic Activity via the Induction of Caspase-Dependent Apoptosis with S-Phase Arrest
Source: Pharmaceuticals (Basel). 2022 Sep 9;15(9):1132. doi: 10.3390/ph15091132 (PMC9501251; doi:10.3390/ph15091132)
Supplement: Supplementary file 1 [file pharmaceuticals-15-01132-s001.zip › FAS-9).01_CompoundReportWithIdentificationHits.pdf]

Qualitative Compound Identification Report

**Data File**	FAS-9).01.d	**Sample Name**	FAS-9
**Sample Type**	Sample	**Position**	81
**Instrument Name**	LC-MS-MS-QTOF	**User Name**	
**Acq Method**	dr-fatin.m	**Acquired Time**	1/11/2022 2:19:57 PM (UTC+02:00)
**IRM Calibration Status**	Success	**DA Method**	khalid lc-ms.m
**Comment**	FAS-9-Dr-Amr		
**Method part to run:**	Acquisition Only	**Sample Group**	
**Info.**		**Stream Name**	LC 1
**Acquisition Time (Local)**	1/11/2022 2:19:57 PM (UTC+02:00)	**Acquisition SW Version**	6200 series TOF/6500 series Q-TOF B.09.00 (B9044.0)
**QTOF Driver Version**	8.00.00	**QTOF Firmware Version**	26.723
**DDE Mode**	1	**Tune Mass Range Max.**	3200

Fragmentor Voltage 130 **Collision Energy** 0 **Ionization Mode** ESI

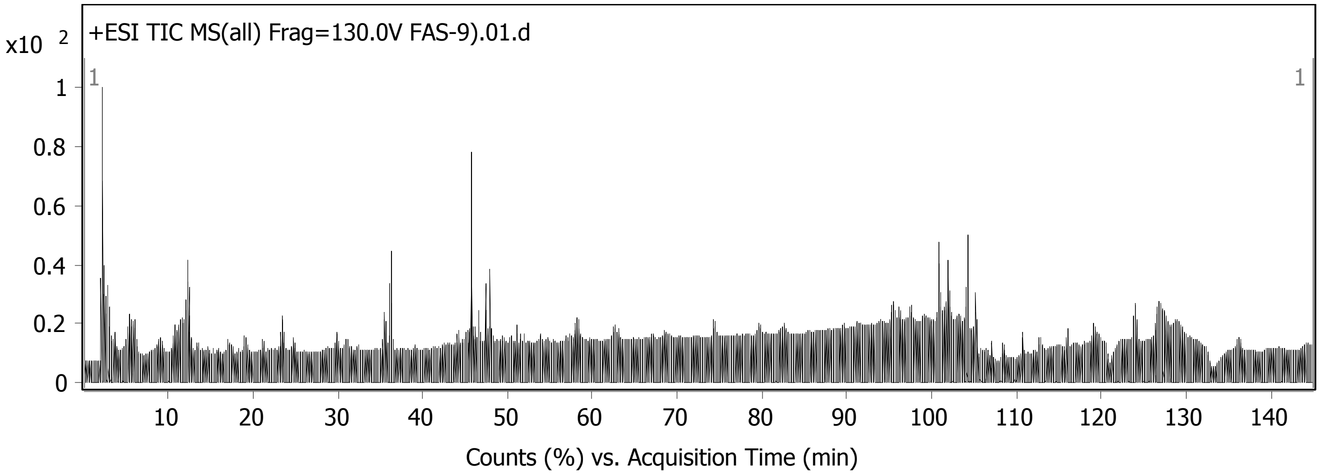

Fragmentor Voltage 130 **Collision Energy** 0 **Ionization Mode** ESI

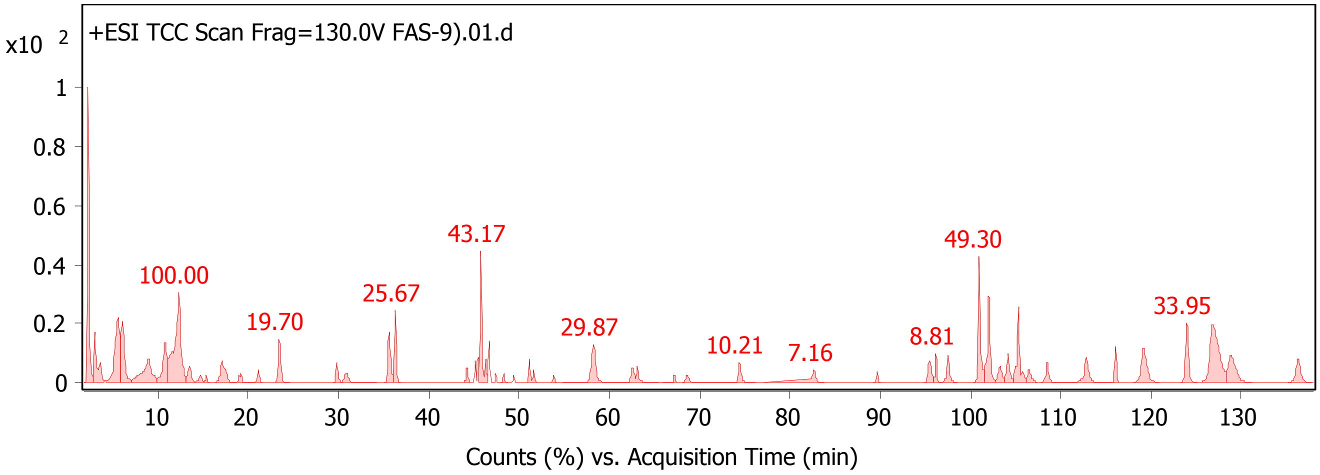

User Chromatogram Peak List

Peak	RT	Height	Normalize	Height	Area	Area	Area Sum	Base Peak	Symmet	Widt

Qualitative Compound Identification Report

#			d Height	%		%	%	m/z	ry	h
82	2.185	10186156.48	99.98	100	133308563.25	89.16	7.85	110.0061	1.39	0.885
82	2.892	1776163.98	17.43	17.44	44897555	30.03	2.64	118.0873	2.66	1.212
82	5.519	2236742.48	21.96	21.96	82555690.16	55.21	4.86	132.0998	0.38	1.616
82	6.024	2107014.98	20.68	20.69	65465724.75	43.78	3.85	132.0998	1.68	1.313
82	8.953	820203.92	8.05	8.05	58426980.32	39.08	3.44	177.098	0.48	2.829
82	10.772	1396382.23	13.71	13.71	49182974.48	32.89	2.89	166.0838	0.3	1.212
82	12.287	3097771.73	30.41	30.41	149518694.95	100	8.8	457.1268	0.45	2.02
82	13.398	560381.73	5.5	5.5	15027399.69	10.05	0.88	122.0953	1.21	1.01
82	14.712	268525.73	2.64	2.64	8846581.38	5.92	0.52	279.1333	1.34	1.49
82	17.035	739210.29	7.26	7.26	28800495.43	19.26	1.69	205.0986	1.99	2.169
82	19.157	306838.26	3.01	3.01	8394224.11	5.61	0.49	309.6505	0.93	2.216
82	21.076	467041.79	4.58	4.59	6660060.61	4.45	0.39	366.1723	1.45	0.769
82	23.4	1517554.1	14.9	14.9	29457082.17	19.7	1.73	506.1957	1.84	1.589
82	29.764	711604.35	6.98	6.99	12059999.07	8.07	0.71	373.1824	2.04	0.84
82	30.875	335419.7	3.29	3.29	11457029.5	7.66	0.67	345.1557	0.97	3.911
82	35.522	1714506.35	16.83	16.83	36645127.61	24.51	2.16	520.2092	1.52	0.909
82	36.229	2515611.98	24.69	24.7	38380555.87	25.67	2.26	551.2823	0.53	0.898
82	44.109	486813.1	4.78	4.78	8051437.18	5.38	0.47	310.2082	1.5	0.731
82	45.726	4572448.48	44.88	44.89	64551467.51	43.17	3.8	535.2799	1.13	0.909
82	48.251	302579.7	2.97	2.97	3237050.71	2.16	0.19	561.2534	0.95	0.693
82	51.08	807955.67	7.93	7.93	14489296.49	9.69	0.85	497.2807	1.7	1.018
82	53.807	247169.9	2.43	2.43	2838143.84	1.9	0.17	64.0159	1.17	0.586
82	58.252	1327493.73	13.03	13.03	44656745.65	29.87	2.63	228.1926	0.93	7.146
82	62.596	508594.1	4.99	4.99	22930655.79	15.34	1.35	227.1063	2.45	3.43
82	67.142	270943.07	2.66	2.66	3545915.2	2.37	0.21	64.0159	0.99	1.785
82	68.556	266829.07	2.62	2.62	8197829.09	5.48	0.48	64.0159	1.21	5.892
82	74.314	677837.48	6.65	6.65	15271787.02	10.21	0.9	64.0159	1.86	2.184
82	82.598	435940.1	4.28	4.28	10698782.28	7.16	0.63	64.0159	0.13	6.638
82	89.67	402306.04	3.95	3.95	4910572.13	3.28	0.29	64.0159	0.9	0.687
82	95.428	774007.6	7.6	7.6	21673318.78	14.5	1.28	64.0159	2	1.073
82	96.135	1003375.98	9.85	9.85	13174491.22	8.81	0.78	64.0159	0.67	0.753
82	97.55	908758.17	8.92	8.92	20810572.43	13.92	1.22	64.0159	0.7	1.756
82	100.883	4344463.48	42.64	42.65	73709645.42	49.3	4.34	628.1859	2.07	1.111
82	101.894	2988870.23	29.34	29.34	71087859.93	47.54	4.18	628.1859	0.68	1.313
82	103.207	590713.23	5.8	5.8	17280047.51	11.56	1.02	326.3398	0.83	0.909
82	104.116	986849.73	9.69	9.69	24121074.9	16.13	1.42	803.5371	1	1.01
82	105.227	2625657.73	25.77	25.78	58551517.09	39.16	3.45	702.2113	0.59	1.414
82	106.44	448678.73	4.4	4.4	13901253.6	9.3	0.82	312.3219	1.43	1.209
82	108.359	701568.73	6.89	6.89	16111734.43	10.78	0.95	776.2291	1.39	1.515
82	112.703	898318.92	8.82	8.82	32997976.07	22.07	1.94	850.256	1.08	5.354
82	116.037	1233442.48	12.11	12.11	19008776.97	12.71	1.12	924.2712	0.83	1.717
82	119.168	1210229.35	11.88	11.88	52780570.77	35.3	3.11	924.2712	1.14	2.826
82	123.916	2086825.73	20.48	20.49	50767275.33	33.95	2.99	998.2788	1.34	1.818
82	126.745	2024997.6	19.88	19.88	142897461.65	95.57	8.41	680.4796	2.72	2.526
82	128.867	938836.54	9.22	9.22	57397209.23	38.39	3.38	998.2788	1.57	2.866
82	136.241	801850.79	7.87	7.87	30504848.41	20.4	1.8	1073.3052	0.99	2.682

Compound Table

Compound Label	RT	Mass	Name	MFG Formula	MFG Diff (ppm)	DB Formula	DB Diff (ppm)	Hits (DB)	Score (Lib)	Library
Cpd 1: 2,4-Dichlorotoluene; C7 H6 Cl2; 2.181	2.181	159.9863	2,4-Dichlorotoluene			C7 H6 Cl2	-10.47	3		
Cpd 2: 4-Thiocyanatophenol; C7 H5 N O S; 2.182	2.182	151.0095	4-Thiocyanatophenol			C7 H5 N O S	-2.16	2		

Qualitative Compound Identification Report

Cpd 3: 2.183	2.183	113.9807		<none>				0		
Cpd 4: S-Propyl thiosulfate; C3 H8 O3 S2; 2.184	2.184	155.9911	S-Propyl thiosulfate			C3 H8 O3 S2	2.43	2		
Cpd 5: 2.185	2.185	85.9863		<none>				0		
Cpd 6: S-Methyl methanesulfinothioate; C2 H6 O S2; 2.187	2.187	109.9861	S-Methyl methanesulfinothioate			C2 H6 O S2	-0.42	1	0	
Cpd 7: 1-Isothiocyanato-2-(methylthio)ethane; C4 H7 N S2; 2.188	2.188	133.0018	1-Isothiocyanato-2-(methylthio)ethane			C4 H7 N S2	1.71	1		
Cpd 8: 4-Guanidino-1-butanol; C5 H13 N3 O; 2.286	2.286	131.1061	4-Guanidino-1-butanol			C5 H13 N3 O	-2.07	4		
Cpd 9: Amino acid(Arg-); C6 H14 N4 O2; 2.405	2.405	174.1117	Amino acid(Arg-)			C6 H14 N4 O2	0.11	3		
Cpd 10: Ethyl lactate; C5 H10 O3; 2.912	2.912	118.0634	Ethyl lactate			C5 H10 O3	-3.13	35	0	
Cpd 11: Dimethylethanolamine; C4 H11 N O; 2.916	2.916	89.085	Dimethylethanolamine			C4 H11 N O	-10.84	11	0	
Cpd 12: L-Valine; C5 H11 N O2; 3.502	3.502	117.0791	L-Valine			C5 H11 N O2	-1.37	41	99.87	D:\MassHunter\PCDL\METLIN PCDL B.08.00\Metlin_AM_PCDL.cdb
Cpd 13: Dimethylethanolamine; C4 H11 N O; 3.544	3.544	89.0849	Dimethylethanolamine			C4 H11 N O	-9.67	11	0	
Cpd 14: Methyl orsellinate; C9 H10 O4; 5.238	5.238	182.0578	Methyl orsellinate			C9 H10 O4	0.63	27		
Cpd 15: L-Isoleucine; C6 H13 N O2; 5.456	5.456	131.0949	L-Isoleucine			C6 H13 N O2	-2.31	14	95.82	D:\MassHunter\PCDL\METLIN PCDL B.08.00\Metlin_AM_PCDL.cdb
Cpd 16: 2-Amino-3-methyl-1-butanol; C5 H13 N O; 5.458	5.458	103.1003	2-Amino-3-methyl-1-butanol			C5 H13 N O	-6.13	20	0	
Cpd 17: L-Isoleucine; C6 H13 N O2; 5.990	5.99	131.0948	L-Isoleucine			C6 H13 N O2	-1.33	14	95.73	D:\MassHunter\PCDL\METLIN PCDL B.08.00\Metlin_AM_PCDL.cdb
Cpd 18: 2-Amino-3-methyl-1-butanol; C5 H13 N O; 5.996	5.996	103.1003	2-Amino-3-methyl-1-butanol			C5 H13 N O	-5.8	20	0	
Cpd 19: MDAI; C10 H11 N O2; 8.766	8.766	177.0788	MDAI			C10 H11 N O2	0.97	10		
Cpd 20: 5-Pentyloxazole; C8 H13 N O; 9.486	9.486	139.0997	5-Pentyloxazole			C8 H13 N O	0.09	22		
Cpd 21: Ginkgotoxin; C9 H13 N O3; 10.692	10.692	183.0895	Ginkgotoxin			C9 H13 N O3	0.22	22		
Cpd 22: His His His; C18 H23 N9 O4; 11.336	11.336	429.1878	His His His			C18 H23 N9 O4	-1.1	64		
Cpd 23: Wilfordine; C43 H49 N O19; 11.437	11.437	883.287	Wilfordine			C43 H49 N O19	3.22	4		
Cpd 24: His Met Phe; C20 H27 N5 O4 S; 11.976	11.976	433.1791	His Met Phe			C20 H27 N5 O4 S	-1.67	14		
Cpd 25: Glycerol 1-propanoate diacetate; C10 H16 O6; 12.154	12.154	232.0948	Glycerol 1-propanoate diacetate			C10 H16 O6	-0.37	15		
Cpd 26: His His His; C18 H23 N9 O4; 12.220	12.22	429.1877	His His His			C18 H23 N9 O4	-0.98	64		
Cpd 27: Wilfordine; C43 H49 N O19; 12.256	12.256	883.2875	Wilfordine			C43 H49 N O19	2.69	4		
Cpd 28: 3,4-Dihydroxyphenylpropanoate; C9 H10 O4; 12.258	12.258	182.0579	3,4-Dihydroxyphenylpropanoate			C9 H10 O4	-0.04	27		
Cpd 29: H-8; C12 H15 N3 O2 S; 12.263	12.263	265.09	H-8			C12 H15 N3 O2 S	-5.66	2		
Cpd 30: Met Gly Asn; C11 H20 N4 O5 S; 12.268	12.268	320.1112	Met Gly Asn			C11 H20 N4 O5 S	13.38	26		
Cpd 31: Furaneol 4-glucoside; C12 H18 O8; 12.268	12.268	290.1003	Furaneol 4-glucoside			C12 H18 O8	-0.29	27		
Cpd 32: 3,4-Dimethylphenol; C8 H10 O; 13.446	13.446	122.0733	3,4-Dimethylphenol			C8 H10 O	-0.71	20		
Cpd 33: Tyrosyl-Proline; C14 H18 N2 O4; 14.673	14.673	278.1267	Tyrosyl-Proline			C14 H18 N2 O4	-0.19	16		
Cpd 34: Tetrahydropteroyltri-L-glutamate; C24 H34 N8 O12; 15.311	15.311	626.2327	Tetrahydropteroyltri-L-glutamate			C24 H34 N8 O12	-4.91	1		
Cpd 35: Methyl 1-methoxy-1H-indole-3-carboxylate; C11 H11 N O3; 17.013	17.013	205.0738	Methyl 1-methoxy-1H-indole-3-carboxylate			C11 H11 N O3	0.3	11		
Cpd 36: L-isoleucyl-L-proline; C11 H20 N2 O3; 17.368	17.368	228.1474	L-isoleucyl-L-proline			C11 H20 N2 O3	0.03	6		
Cpd 37: Quercetin 7-[xylosyl-(1->2)-rhamnosyl-(1->2)-rhamnosyl]-(1->6)-glucoside; C38 H48 O24; 18.971	18.971	888.2509	Quercetin 7-[xylosyl-(1->2)-rhamnosyl-(1->2)-rhamnosyl]-(1->6)-glucoside			C38 H48 O24	3.03	6		
Cpd 38: Asn Trp Trp Leu; C32 H39 N7 O6; 19.178	19.178	617.2966	Asn Trp Trp Leu			C32 H39 N7 O6	-0.66	26	0	
Cpd 39: C38 H46 N6 O9; 21.102	21.102	730.3332		C38 H46 N6 O9	-0.83	C38 H46 N6 O9		200	0	
Cpd 40: C27 H27 N3 O7; 23.441	23.441	505.1852		C27 H27 N3 O7	-0.63	C27 H27 N3 O7		256		
Cpd 41: C41 H50 N3 O10; 29.806	29.806	744.3494		C41 H50 N3 O10	0.24	C41 H50 N3 O10		208	0	
Cpd 42: Butyl 3-O-beta-D-	30.862	322.1632	Butyl 3-O-beta-D-			C14 H26 O8	-1.2	28		

Agilent Technologies

glucopyranosyl-butanoate; C14 H26 O8; 30.862			glucopyranosyl-butanoate							
Cpd 43: C28 H29 N3 O7; 35.517	35.517	519.2009		C28 H29 N3 O7	-0.71	C28 H29 N3 O7		269		
Cpd 44: 36.212	36.212	569.2792						0		
Cpd 45: Ouabagenin; C23 H34 O8; 36.212	36.212	438.2262	Ouabagenin			C23 H34 O8	-1.87	28		
Cpd 46: Tyr Tyr Leu Lys; C30 H43 N5 O7; 36.213	36.213	585.3155	Tyr Tyr Leu Lys			C30 H43 N5 O7	1.21	25		
Cpd 47: 11-alpha-O-beta-D-Glucopyranosyl-16alpha-O-methylneoquassin; C29 H44 O11; 36.215	36.215	568.2893	11-alpha-O-beta-D-Glucopyranosyl-16alpha-O-methylneoquassin			C29 H44 O11	-1.73	32		
Cpd 48: Methyl levulinate; C6 H10 O3; 36.216	36.216	130.0634	Methyl levulinate			C6 H10 O3	-2.76	36		
Cpd 49: Ginsenoside Rh8; C36 H60 O9; 44.110	44.11	636.4204	Ginsenoside Rh8			C36 H60 O9	5.2	6	0	
Cpd 50: Prenyl arabinosyl-(1->6)-glucoside; C16 H28 O10; 44.196	44.196	380.1687	Prenyl arabinosyl-(1->6)-glucoside			C16 H28 O10	-1.27	39		
Cpd 51: Ginsenoside Rh8; C36 H60 O9; 45.110	45.11	636.4284	Ginsenoside Rh8			C36 H60 O9	-7.25	6	0	
Cpd 52: Sanchinoside B1; C36 H62 O9; 45.358	45.358	638.4363	Sanchinoside B1			C36 H62 O9	4.82	17	0	
Cpd 53: Desglucocheirotoxol; C29 H44 O10; 45.727	45.727	552.2943	Desglucocheirotoxol			C29 H44 O10	-1.55	34	0	
Cpd 54: Corchoroside A; C29 H42 O9; 45.727	45.727	534.2835	Corchoroside A			C29 H42 O9	-1.13	36	0	
Cpd 55: Picrasin C; C23 H34 O7; 45.727	45.727	422.2313	Picrasin C			C23 H34 O7	-1.89	41		
Cpd 56: C29 H35 N12; 45.728	45.728	551.3093		C29 H35 N12	2.62	C29 H35 N12		176		
Cpd 57: Glu Trp Leu Gln; C27 H38 N6 O8; 45.728	45.728	574.2763	Glu Trp Leu Gln			C27 H38 N6 O8	-2.03	28	0	
Cpd 58: Sanchinoside B1; C36 H62 O9; 46.198	46.198	638.436	Sanchinoside B1			C36 H62 O9	5.28	17	0	
Cpd 59: Pro Ile Lys Arg; C23 H44 N8 O5; 46.250	46.25	512.344	Pro Ile Lys Arg			C23 H44 N8 O5	-1.11	42		
Cpd 60: Phe Thr Thr Thr; C21 H32 N4 O8; 46.603	46.603	468.2216	Phe Thr Thr Thr			C21 H32 N4 O8	0.91	43	0	
Cpd 61: Pro Ile Lys Arg; C23 H44 N8 O5; 46.644	46.644	512.3443	Pro Ile Lys Arg			C23 H44 N8 O5	-1.66	43		
Cpd 62: Mulberrofuran T; C44 H44 O9; 47.356	47.356	716.3021	Mulberrofuran T			C44 H44 O9	-4.99	7	0	
Cpd 63: Trp Ser Lys His; C26 H36 N8 O6; 48.243	48.243	556.274	Trp Ser Lys His			C26 H36 N8 O6	3.21	26		
Cpd 64: 14-O-(alpha-L-rhamnopyranosyl)-7S,14R-dihydroxy-7,9,13,17-tetramethyl-2E,4E,8E,10E,12E,16E-octadecahexaenoic acid; C28 H42 O8; 49.363	49.363	506.2884	14-O-(alpha-L-rhamnopyranosyl)-7S,14R-dihydroxy-7,9,13,17-tetramethyl-2E,4E,8E,10E,12E,16E-octadecahexaenoic acid			C28 H42 O8	-0.92	29		
Cpd 65: C22 H36 N9 O3; 51.097	51.097	474.294		C22 H36 N9 O3	0.26	C22 H36 N9 O3		60	0	
Cpd 66: C22 H36 N9 O3; 51.581	51.581	474.2942		C22 H36 N9 O3	-0.29	C22 H36 N9 O3		60	0	
Cpd 67: QX-314; C16 H26 N2 O; 53.807	53.807	262.2017	QX-314			C16 H26 N2 O	10.56	1		
Cpd 68: C13 H25 N O2; 58.225	58.225	227.1888		C13 H25 N O2	-1.17	C13 H25 N O2		3		
Cpd 69: Isoamyl p-anisate; C13 H18 O3; 62.574	62.574	222.1258	Isoamyl p-anisate			C13 H18 O3	-0.75	19	0	
Cpd 70: 4-methyltridecan-7-ol; C14 H30 O; 63.107	63.107	214.2299	4-methyltridecan-7-ol			C14 H30 O	-1.23	10		
Cpd 71: Phytosphingosine; C18 H39 N O3; 67.109	67.109	317.2936	Phytosphingosine			C18 H39 N O3	-1.94	1		
Cpd 72: Xanthyletine; C14 H12 O3; 68.525	68.525	228.0788	Xanthyletine			C14 H12 O3	-0.84	18		
Cpd 73: Ser Ser Ser Ser; C12 H22 N4 O9; 74.364	74.364	366.1393	Ser Ser Ser Ser			C12 H22 N4 O9	-1.61	39		
Cpd 74: 4-Phenyl-2-butenal; C10 H10 O; 74.365	74.365	146.0732	4-Phenyl-2-butenal			C10 H10 O	0.02	26		
Cpd 75: 3,8-Dihydroxy-6-methoxy-7(11)-eremophilen-12,8-olide; C16 H24 O5; 82.623	82.623	296.1627	3,8-Dihydroxy-6-methoxy-7(11)-eremophilen-12,8-olide			C16 H24 O5	-1.14	12		
Cpd 76: Pyranodelphinin B; C24 H23 O12; 89.650	89.65	503.1218	Pyranodelphinin B			C24 H23 O12	-5.58	1		
Cpd 77: MG(16:0/0:0/0:0)[rac]; C19 H38 O4; 95.382	95.382	330.2778	MG(16:0/0:0/0:0)[rac]			C19 H38 O4	-2.3	4	0	
Cpd 78: Oleoyl sarcosine; C21 H39 N O3; 95.541	95.541	353.2939	Oleoyl sarcosine			C21 H39 N O3	-2.43	5	0	
Cpd 79: R207910; C32 H31 Br N2 O2; 96.169	96.169	554.1528	R207910			C32 H31 Br N2 O2	7.33	1		
Cpd 80: R207910; C32 H31 Br N2 O2; 97.512	97.512	554.1525	R207910			C32 H31 Br N2 O2	8.01	1		
Cpd 81: Isorhamnetin 3-O-[b-D-xylopyranosyl-(1->6)-b-D-glucopyranoside]; C27 H30 O16; 100.855	100.855	610.1585	Isorhamnetin 3-O-[b-D-xylopyranosyl-(1->6)-b-D-glucopyranoside]			C27 H30 O16	-8.33	48		
Cpd 82: Stearamide; C18 H37 N O; 100.937	100.937	283.2883	Stearamide			C18 H37 N O	-2.89	4	59.81	D:\MassHunter\PCDL\METLIN PCDL B.08.00\Metlin_AM_PCDL.cdb
Cpd 83: Isorhamnetin 3-O-[b-D-xylopyranosyl-(1->6)-b-D-glucopyranoside]; C27 H30 O16; 101.949	101.949	610.1589	Isorhamnetin 3-O-[b-D-xylopyranosyl-(1->6)-b-D-glucopyranoside]			C27 H30 O16	-9.07	48	0	
Cpd 84: D-erythro-	103.03	327.314	D-erythro-Sphingosine C-20			C20 H41 N O2	-0.92	7	0	

Qualitative Compound Identification Report

Sphingosine C-20; C20 H41 N O2; 103.030										
Cpd 85: Isopropyl stearate; C21 H42 O2; 103.249	103.249	326.3188	Isopropyl stearate			C21 H42 O2	-0.89	26	0	
Cpd 86: Eicosanoyl-EA; C22 H45 N O2; 103.898	103.898	355.345	Eicosanoyl-EA			C22 H45 N O2	0.04	4	0	
Cpd 87: 3α,11β,12α-Trihydroxy-5β-cholan-24-oic Acid; C24 H40 O5; 104.112	104.112	408.2878	3α,11β,12α-Trihydroxy-5β-cholan-24-oic Acid			C24 H40 O5	-0.66	50		
Cpd 88: Dihydrostreptomycin 3'α-phosphate; C21 H42 N7 O15 P; 105.212	105.212	663.2488	Dihydrostreptomycin 3'α-phosphate			C21 H42 N7 O15 P	-1.79	6	0	
Cpd 89: 2'',3''-Di-O-p-coumaroylafzelin; C39 H32 O14; 105.221	105.221	724.1717	2'',3''-Di-O-p-coumaroylafzelin			C39 H32 O14	10.31	9	0	
Cpd 90: Eicosanoyl-EA; C22 H45 N O2; 105.680	105.68	355.3453	Eicosanoyl-EA			C22 H45 N O2	-0.79	27		
Cpd 91: N,N-dimethyl-Safingol; C20 H43 N O2; 106.427	106.427	329.3295	N,N-dimethyl-Safingol			C20 H43 N O2	-0.29	2	0	
Cpd 92: Hyaluronan; C28 H44 N2 O23; 108.421	108.421	776.2295	Hyaluronan			C28 H44 N2 O23	5.07	5	0	
Cpd 93: Quercetin 3-(6'''-sinapylglucosyl)(1->2)-galactoside; C38 H40 O21; 112.775	112.775	832.2132	Quercetin 3-(6'''-sinapylglucosyl)(1->2)-galactoside			C38 H40 O21	-8.4	1	0	
Cpd 94: 116.022	116.022	923.2633		<none>				0		
Cpd 95: 119.170	119.17	923.2633		<none>				0		
Cpd 96: (S)-3-Hydroxytetradecanoyl-CoA; C35 H62 N7 O18 P3 S; 123.955	123.955	993.3061	(S)-3-Hydroxytetradecanoyl-CoA			C35 H62 N7 O18 P3 S	2.36	6	0	
Cpd 97: PG(14:0/15:0); C35 H69 O10 P; 126.955	126.955	680.4576	PG(14:0/15:0)			C35 H69 O10 P	7.74	8	0	
Cpd 98: Loroxanthin ester/ Loroxanthin dodecenoate; C52 H76 O4; 126.956	126.956	764.5743	Loroxanthin ester/ Loroxanthin dodecenoate			C52 H76 O4	0.1	26	0	
Cpd 99: (S)-3-Hydroxytetradecanoyl-CoA; C35 H62 N7 O18 P3 S; 128.936	128.936	993.3061	(S)-3-Hydroxytetradecanoyl-CoA			C35 H62 N7 O18 P3 S	2.37	6	0	
Cpd 100: Retinoyl CoA; C41 H62 N7 O17 P3 S; 136.205	136.205	1049.3137	Retinoyl CoA			C41 H62 N7 O17 P3 S	-0.13	1		

Compound Label	Name	*m/z*	RT	Algorithm	Mass
Cpd 1: 2,4-Dichlorotoluene; C7 H6 Cl2; 2.181	**2,4-Dichlorotoluene**	160.9937	2.181	Find by Molecular Feature	159.9863

Compound Chromatograms

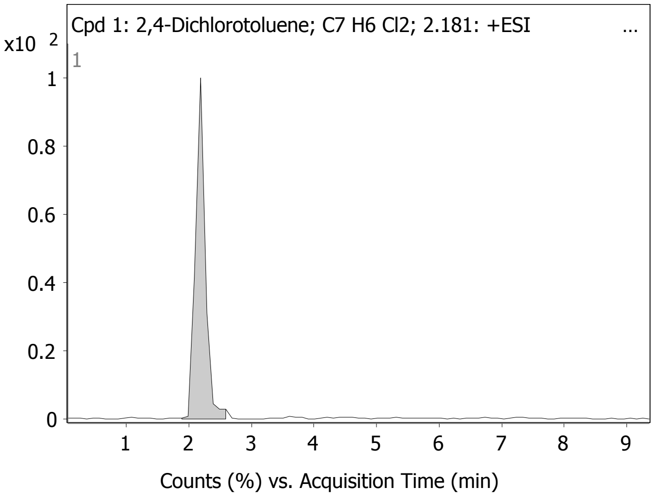

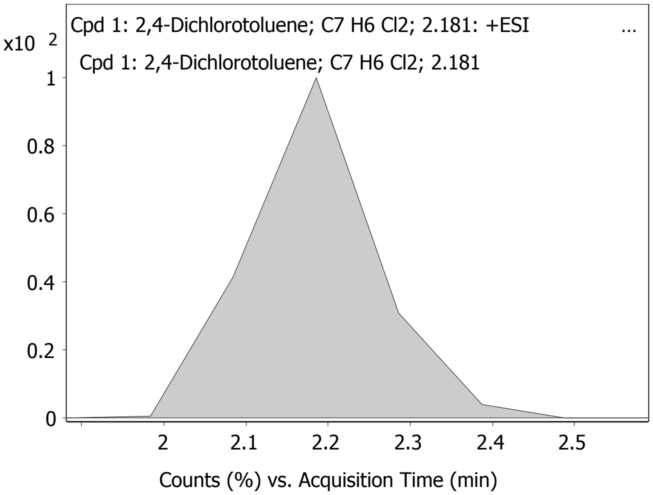

MFE MS Spectrum

Qualitative Compound Identification Report

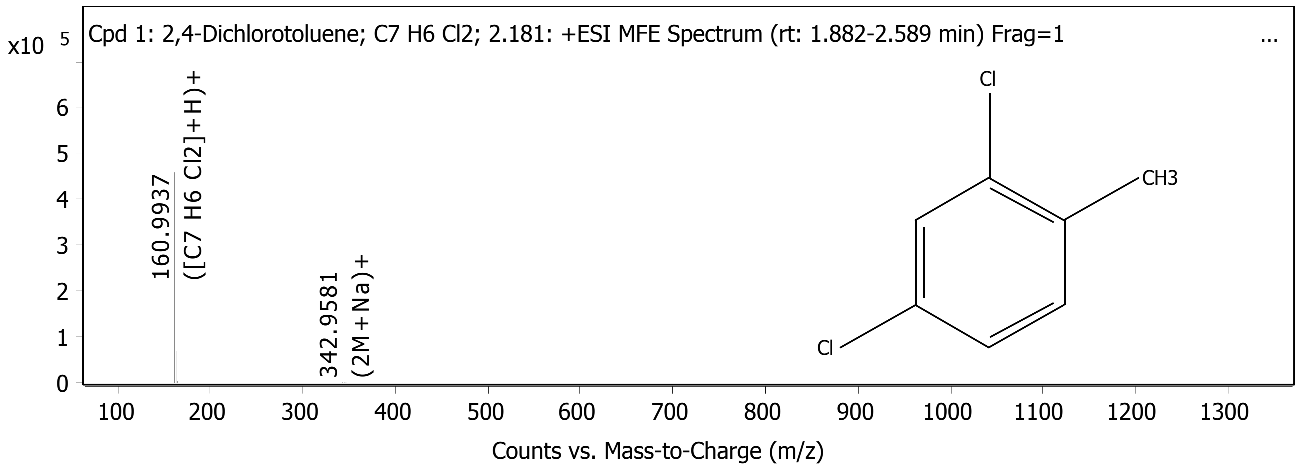

MFE MS Zoomed Spectrum

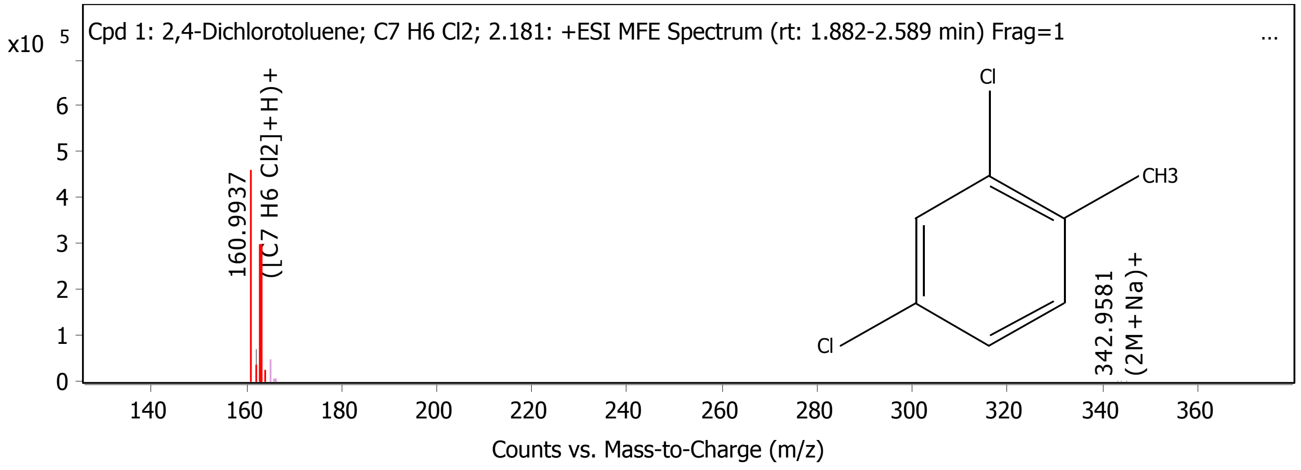

MS Spectrum

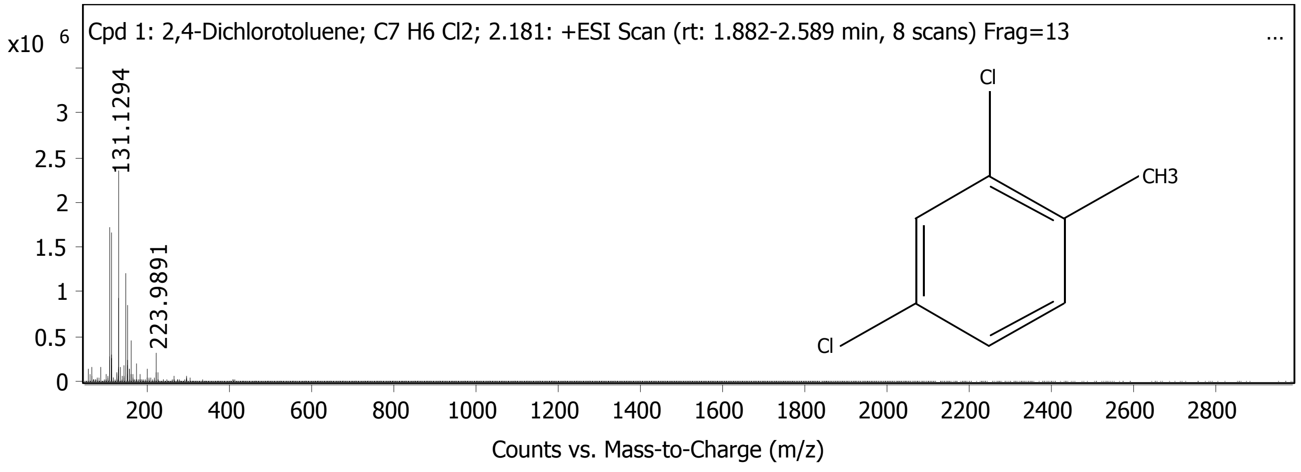

MS Zoomed Spectrum

Qualitative Compound Identification Report

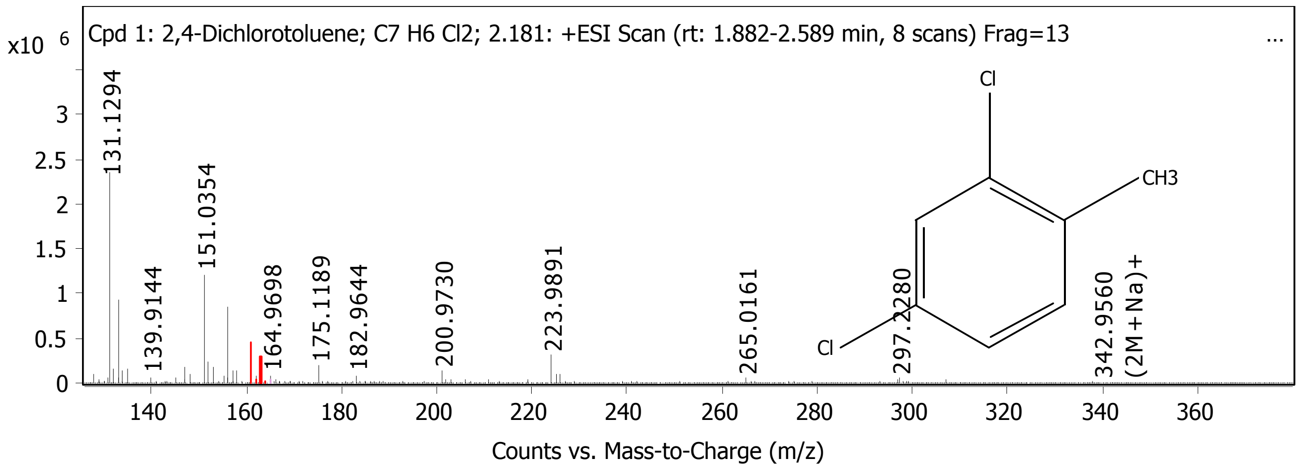

Identification Hit Table

Best Hit	Compound Name	RT	Formula	Notes	Match Score	Mass	Difference	Ion Species
✓	2,4-Dichlorotoluene	2.181	C7 H6 Cl2		50.93	159.9863	-1.68	(M+H)+
	Benzal chloride	2.181	C7 H6 Cl2		50.93	159.9863	-1.68	(M+H)+
	Arsenobetaine	2.181	C5 H12 As O2		45.76	179.0037	1.61	M+[-H2O]

Identification Hit Table

Best Hit	Compound Name	RT	Formula	Notes	Match Score	Mass	Difference	Ion Species

Identification Hit Table

Best Hit	Compound Name	RT	Formula	Notes	Match Score	Mass	Difference	Ion Species

Compound Label	Name	*m/z*	RT	Algorithm	Mass
Cpd 2: 4-Thiocyanatophenol; C7 H5 N O S; 2.182	**4-Thiocyanatophenol**	132.9984	2.182	Find by Molecular Feature	151.0095

Compound Chromatograms

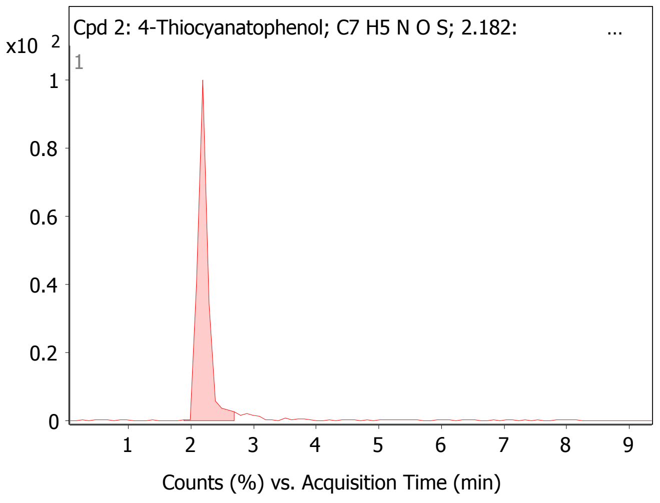

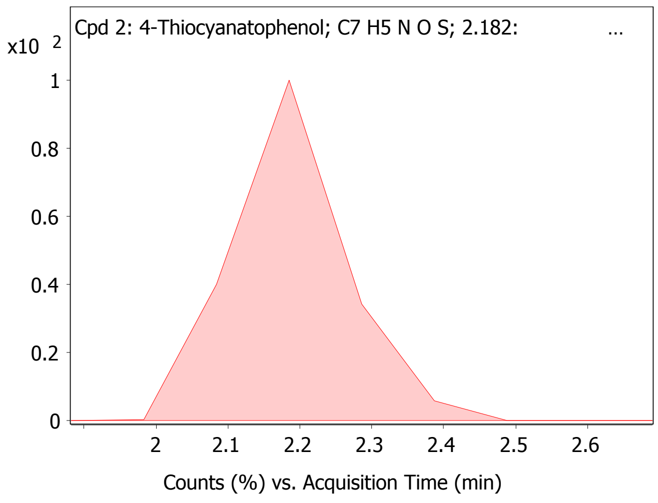

MFE MS Spectrum

Qualitative Compound Identification Report

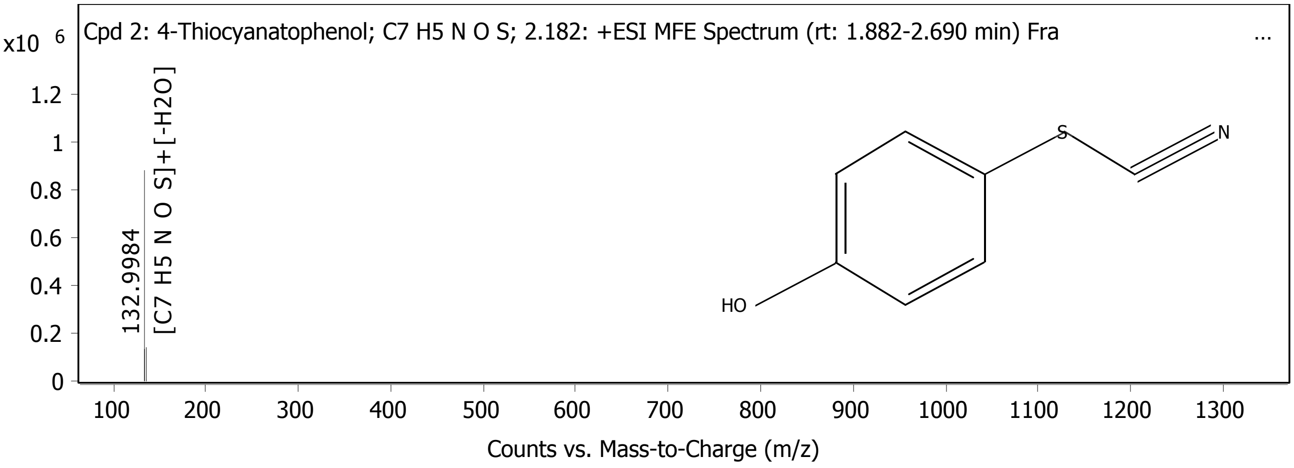

MFE MS Zoomed Spectrum

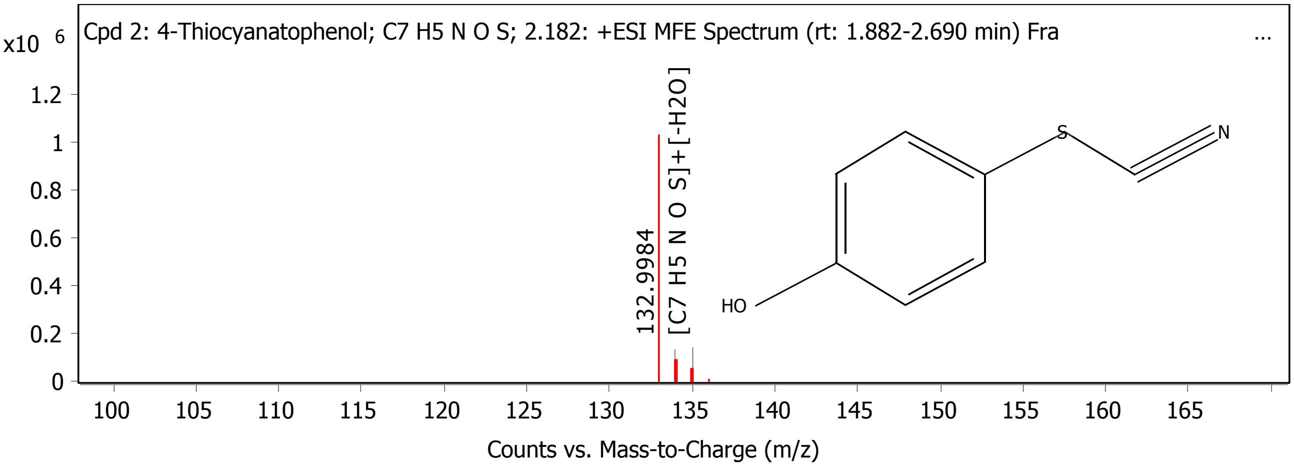

MS Spectrum

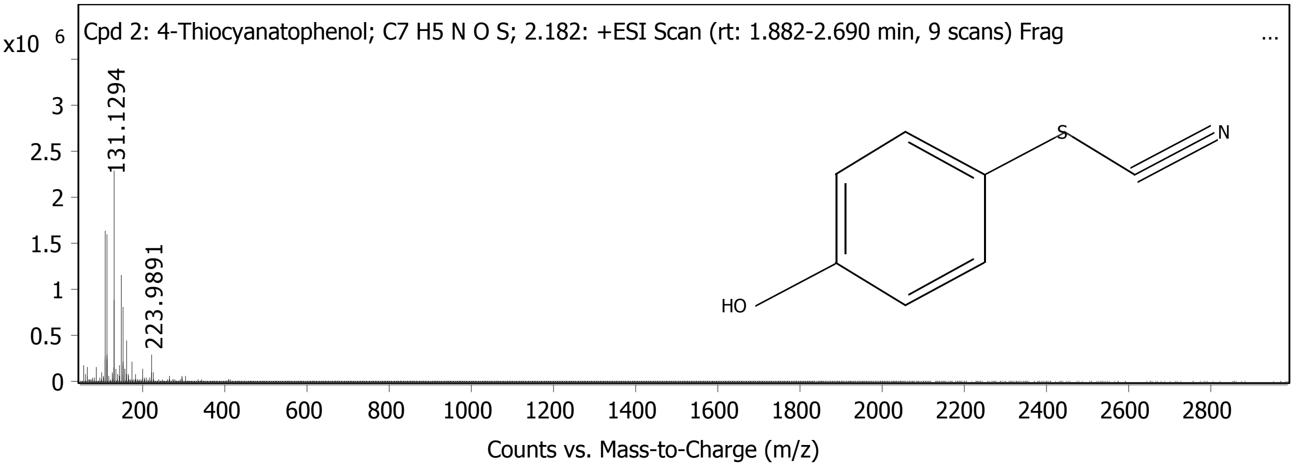

MS Zoomed Spectrum

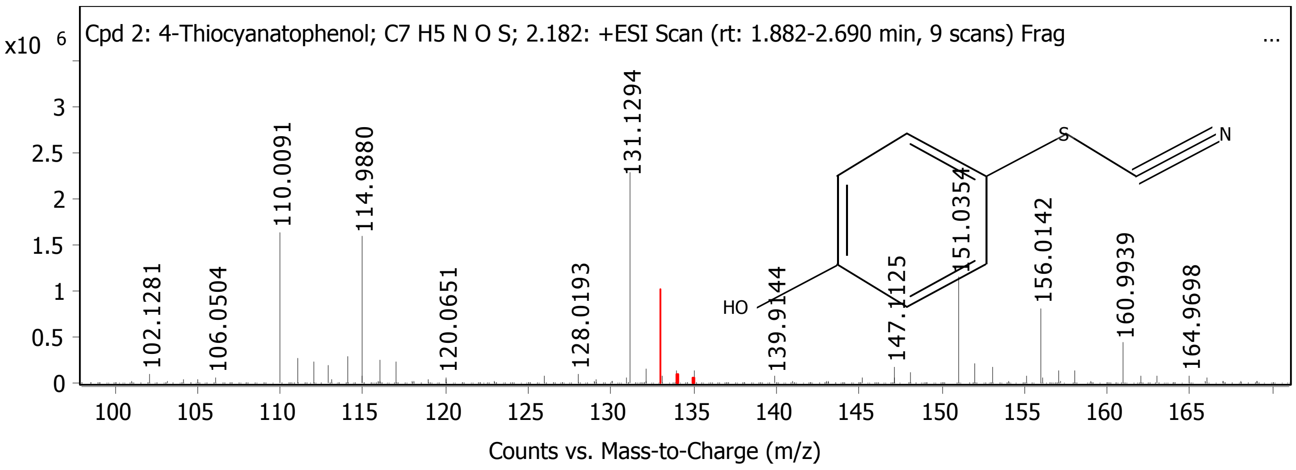

Identification Hit Table

Best Hit	Compound Name	RT	Formula	Notes	Match Score	Mass	Difference	Ion Species
✓	4-Thiocyanatophenol	2.182	C7 H5 N O S		70.37	151.0095	-0.33	M+[-H2O]
	1,2-Benzisothiazol-3(2H)-one	2.182	C7 H5 N O S		70.37	151.0095	-0.33	M+[-H2O]

Identification Hit Table

Best Hit	Compound Name	RT	Formula	Notes	Match Score	Mass	Difference	Ion Species

Identification Hit Table

Best Hit	Compound Name	RT	Formula	Notes	Match Score	Mass	Difference	Ion Species

Compound Label	*m/z*	RT	Algorithm	Mass
Cpd 3: 2.183	114.988	2.183	Find by Molecular Feature	113.9807

Compound Chromatograms

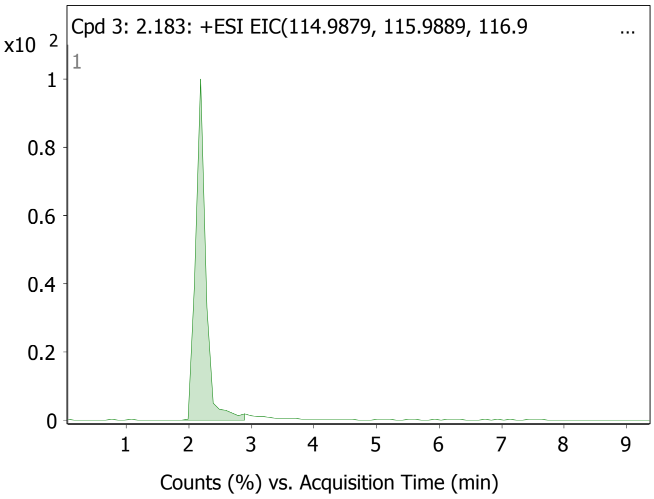

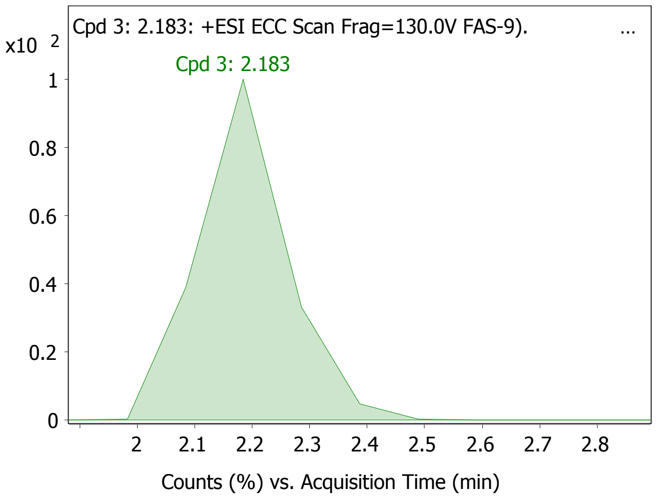

MFE MS Spectrum

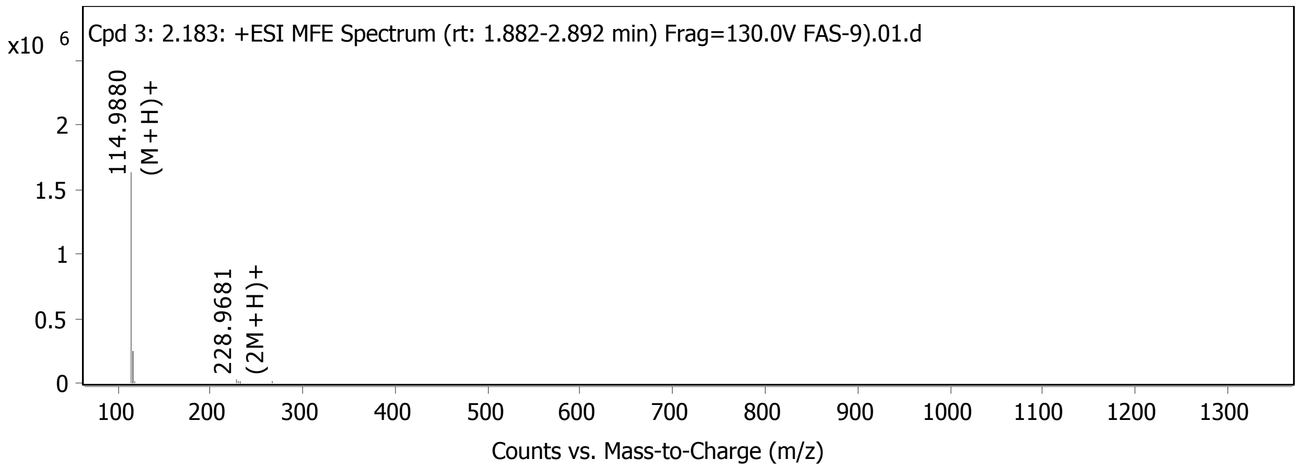

MFE MS Zoomed Spectrum

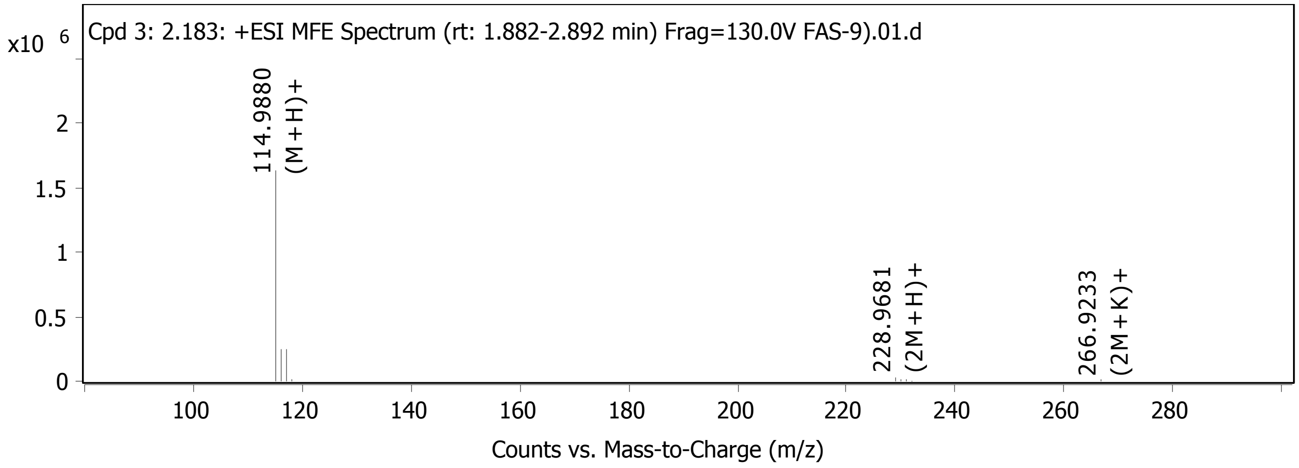

MS Spectrum

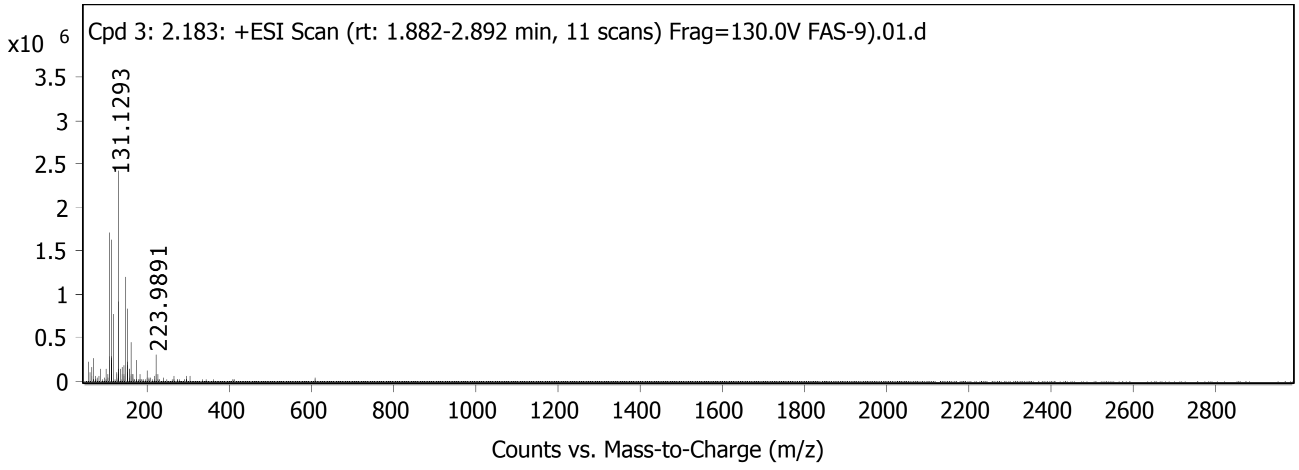

MS Zoomed Spectrum

Qualitative Compound Identification Report

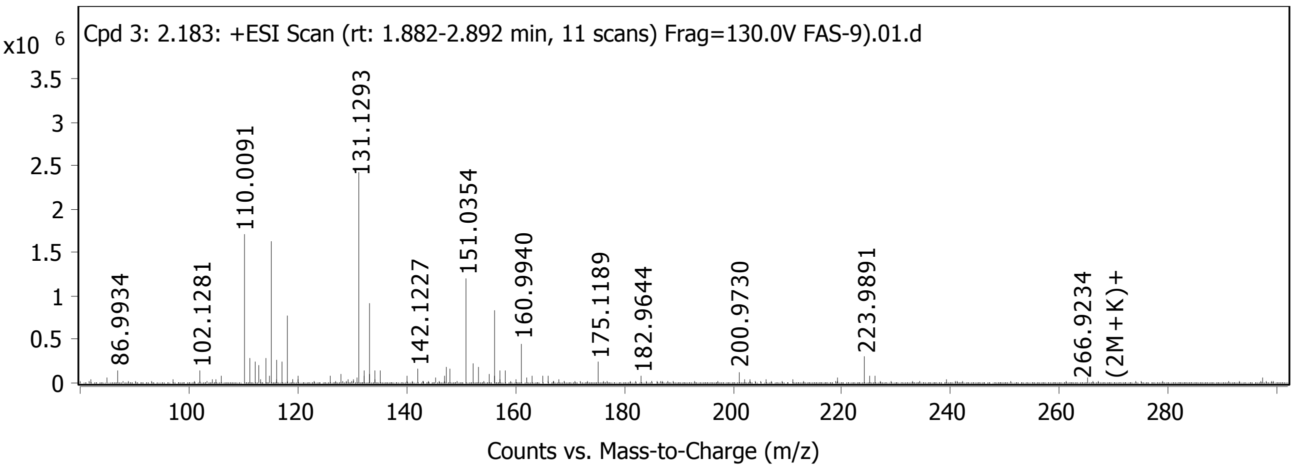

Compound Label	Name	*m/z*	RT	Algorithm	Mass
Cpd 4: S-Propyl thiosulfate; C3 H8 O3 S2; 2.184	**S-Propyl thiosulfate**	156.0143	2.184	Find by Molecular Feature	155.9911

Compound Chromatograms

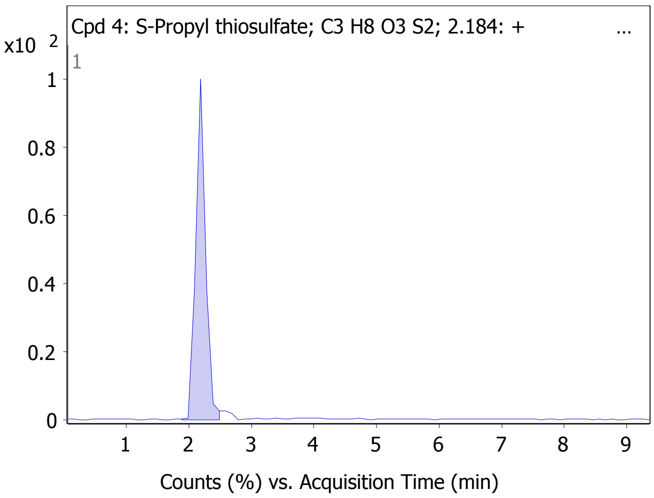

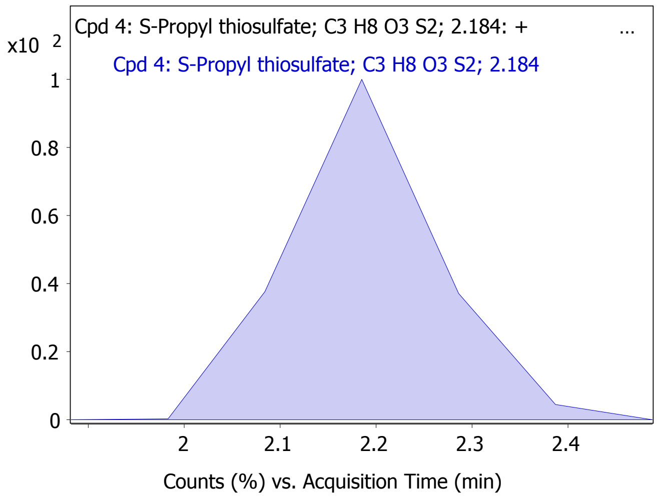

MFE MS Spectrum

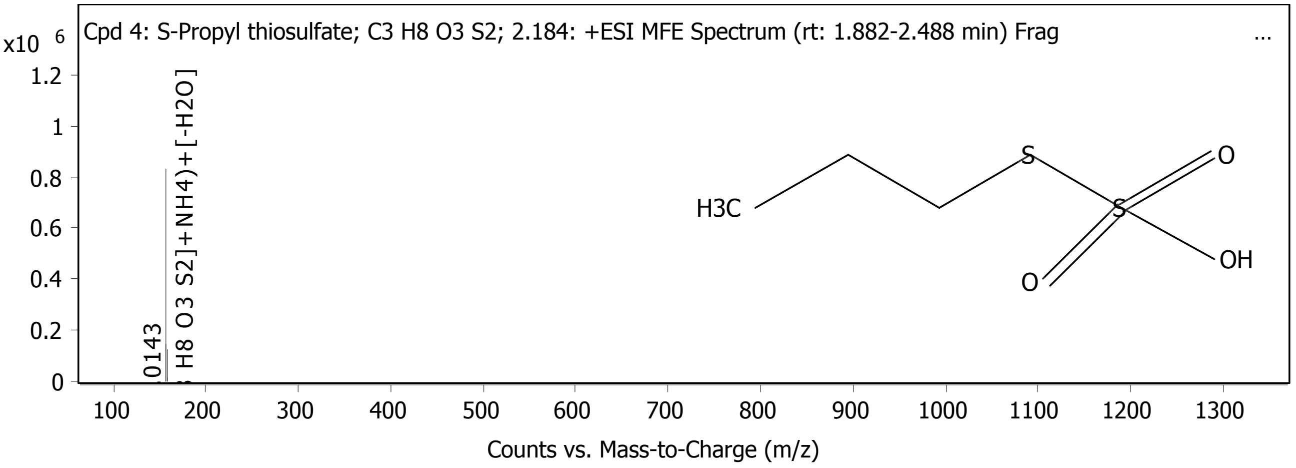

MFE MS Zoomed Spectrum

Qualitative Compound Identification Report

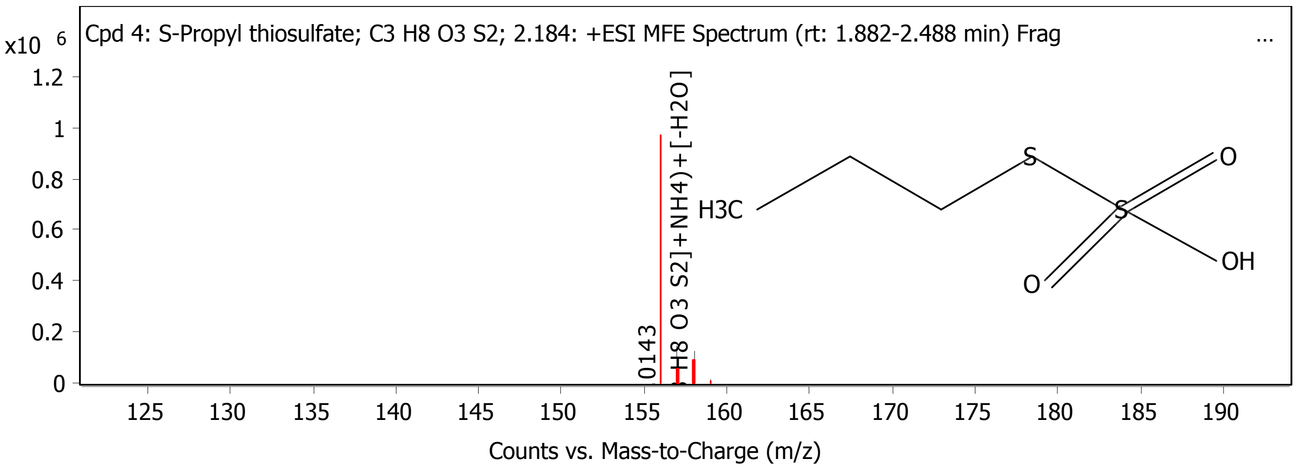

MS Spectrum

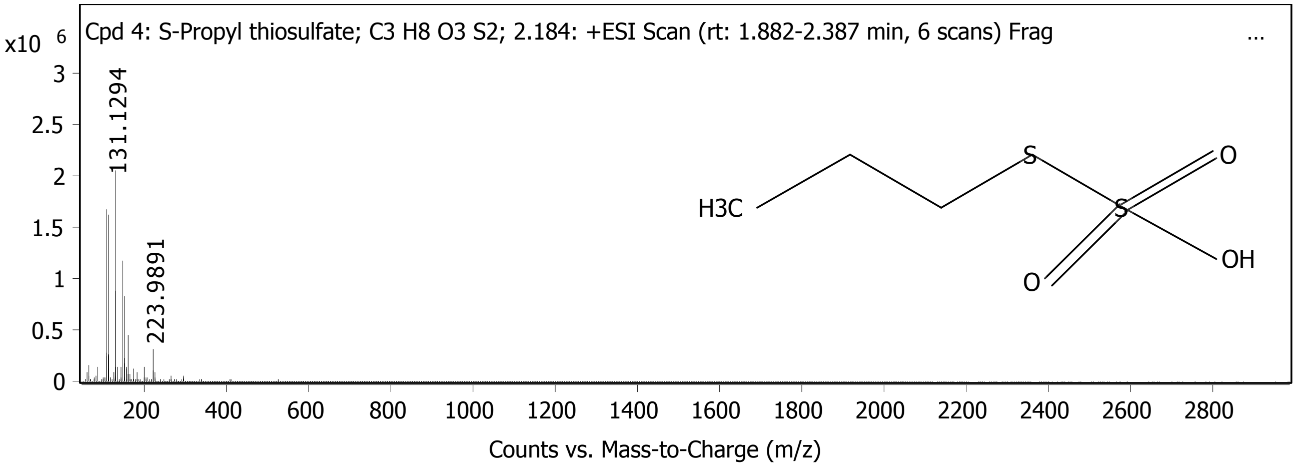

MS Zoomed Spectrum

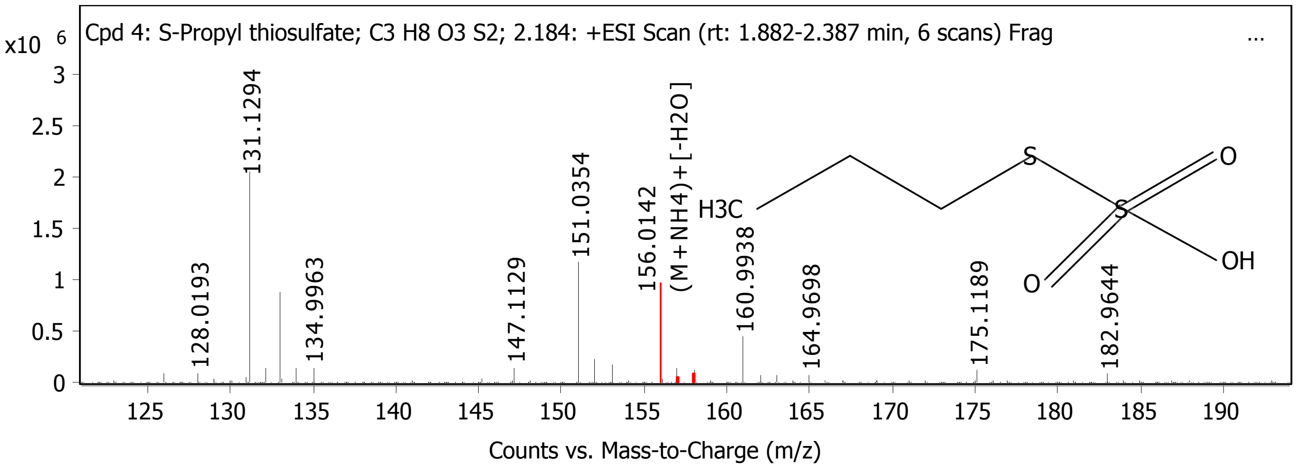

Identification Hit Table

Best Hit	Compound Name	RT	Formula	Notes	Match Score	Mass	Difference	Ion Species
✓	S-Propyl thiosulfate	2.184	C3 H8 O3 S2		74.16	155.9911	0.38	(M+NH4)+[-H2O]
	2-(Methylthio)ethanesulfonate	2.184	C3 H8 O3 S2		74.16	155.9911	0.38	(M+NH4)+[-H2O]

Identification Hit Table

Best Hit	Compound Name	RT	Formula	Notes	Match Score	Mass	Difference	Ion Species

Identification Hit Table

Best Hit	Compound Name	RT	Formula	Notes	Match Score	Mass	Difference	Ion Species

Qualitative Compound Identification Report

Compound Label	*m/z*	RT	Algorithm	Mass
Cpd 5: 2.185	68.9831	2.185	Find by Molecular Feature	85.9863

Compound Chromatograms

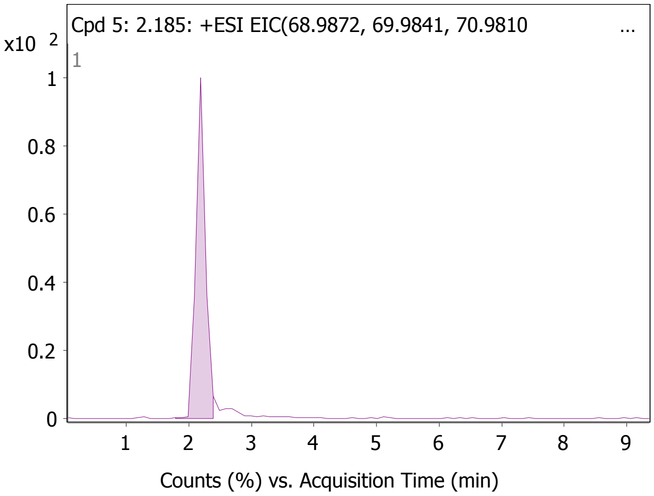

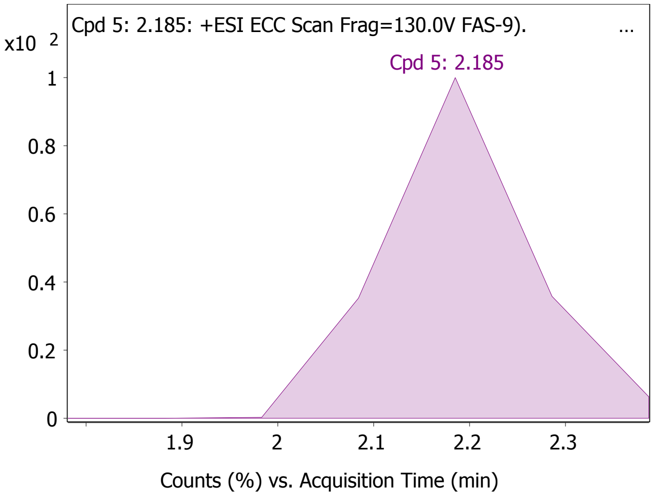

MFE MS Spectrum

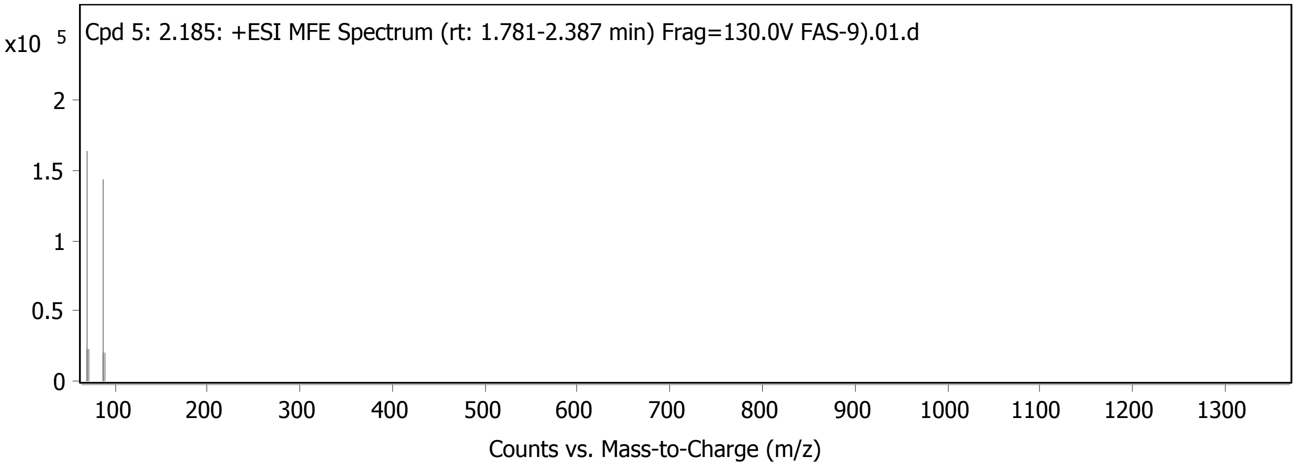

MFE MS Zoomed Spectrum

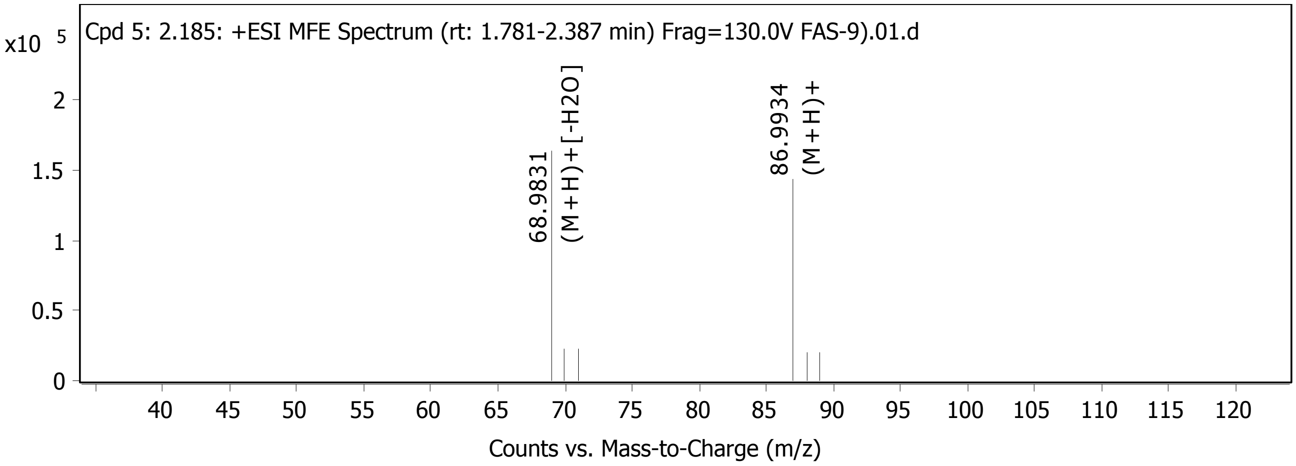

MS Spectrum

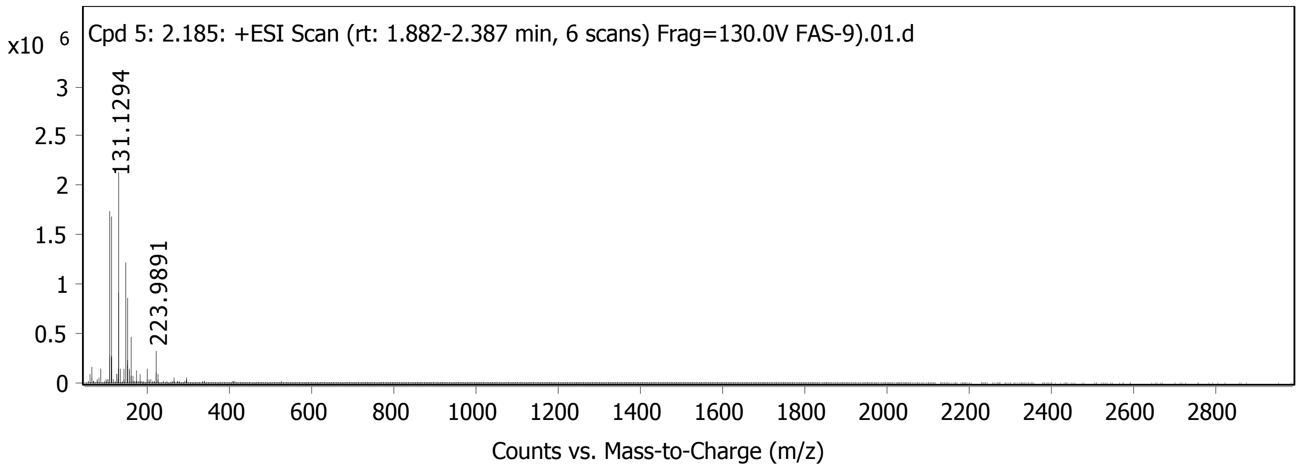

MS Zoomed Spectrum

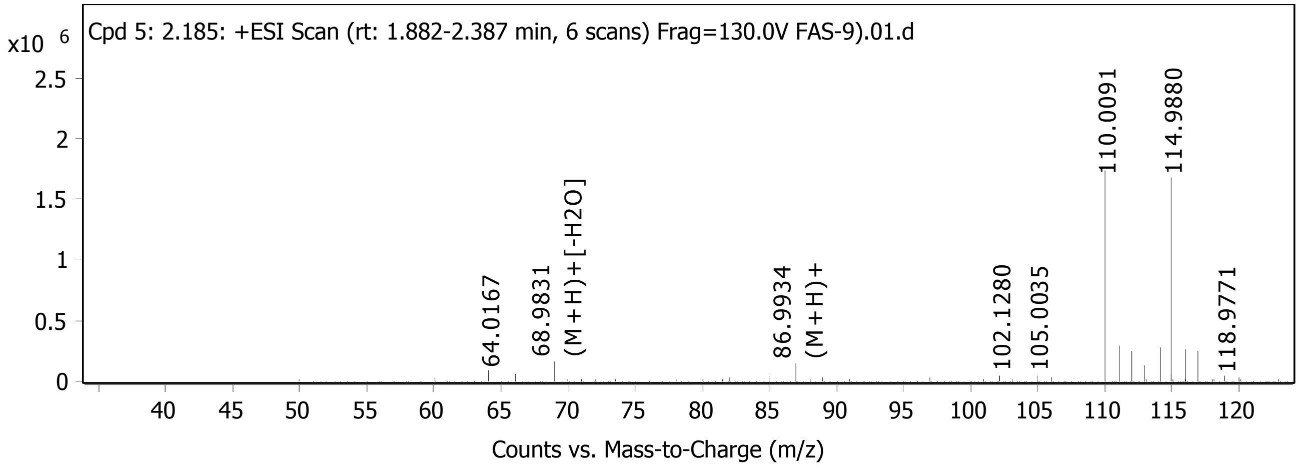

Compound Label	Name	*m/z*	RT	Algorithm	Mass
Cpd 6: S-Methyl methanesulfinothioate; C2 H6 O S2; 2.187	**S-Methyl methanesulfinothioate**	110.0091	2.187	Find by Molecular Feature	109.9861

Compound Chromatograms

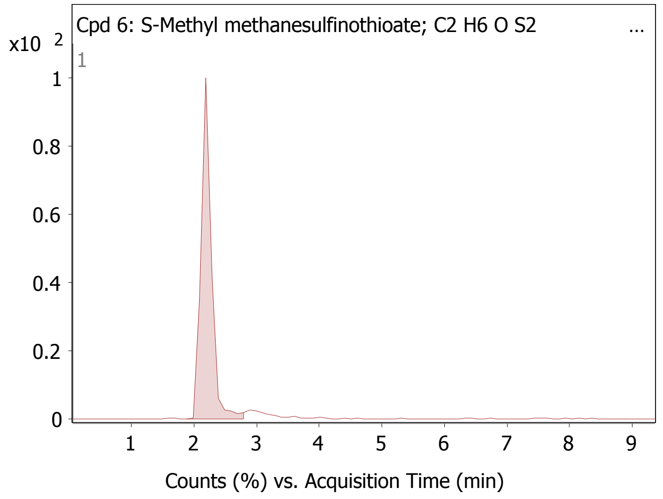

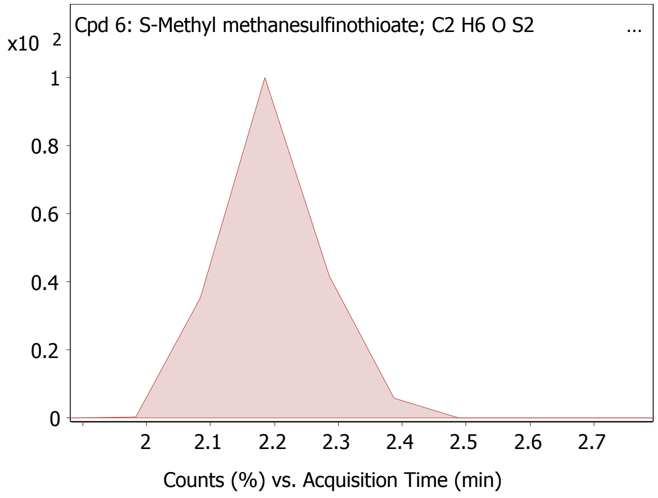

MFE MS Spectrum

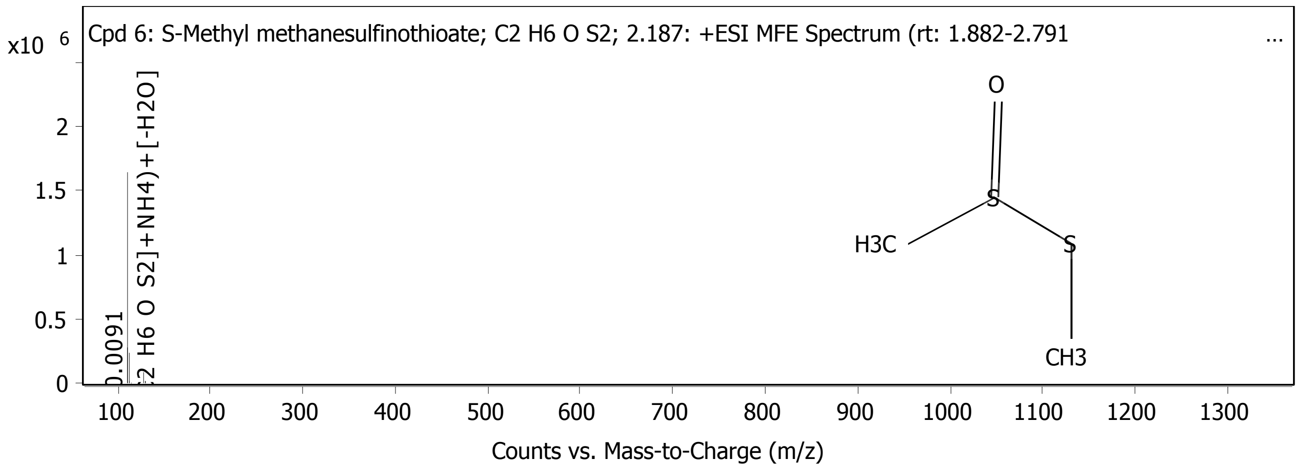

MFE MS Zoomed Spectrum

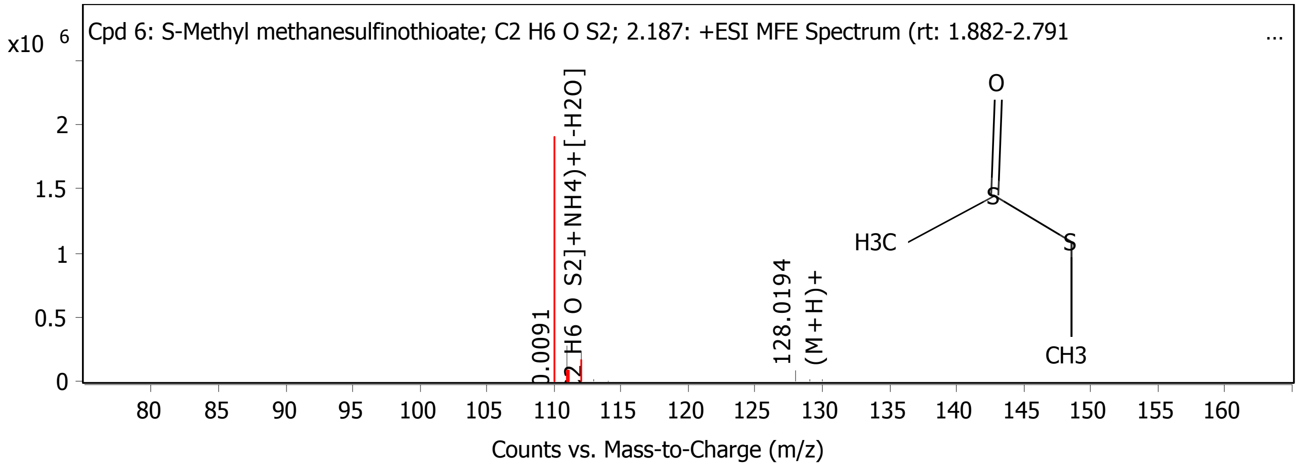

MS Spectrum

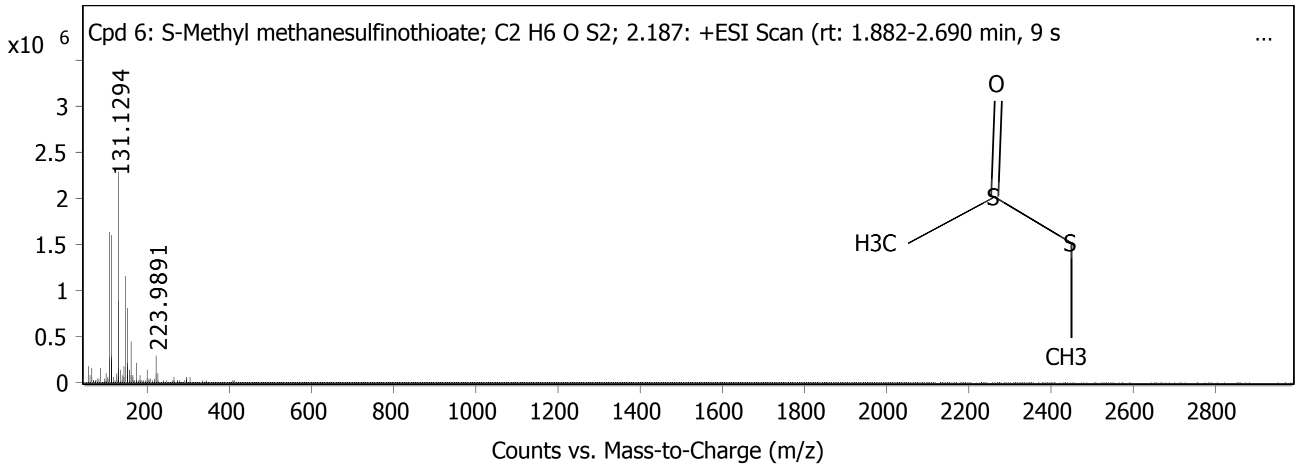

MS Zoomed Spectrum

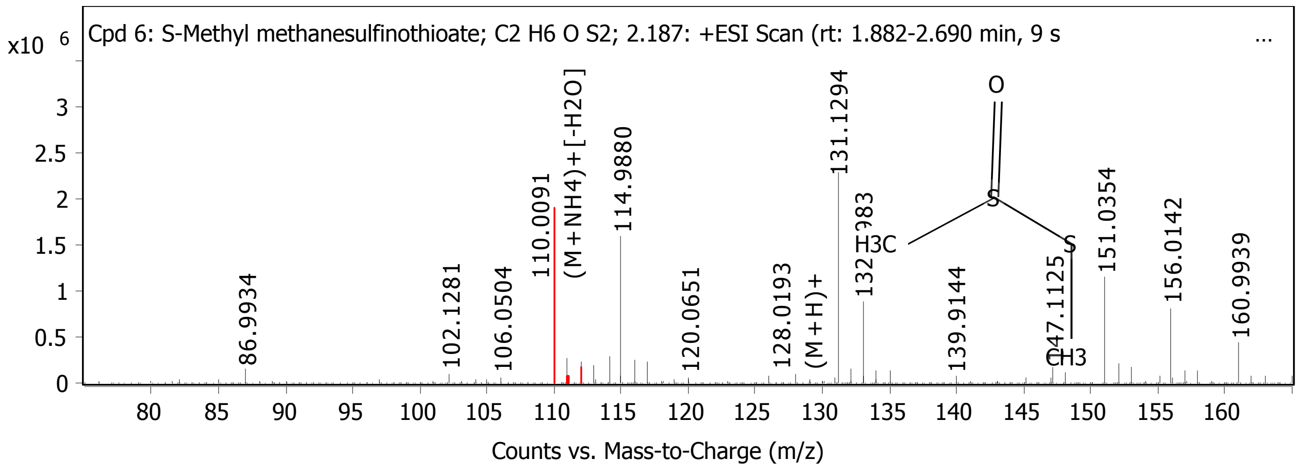

MSMS Spectrum

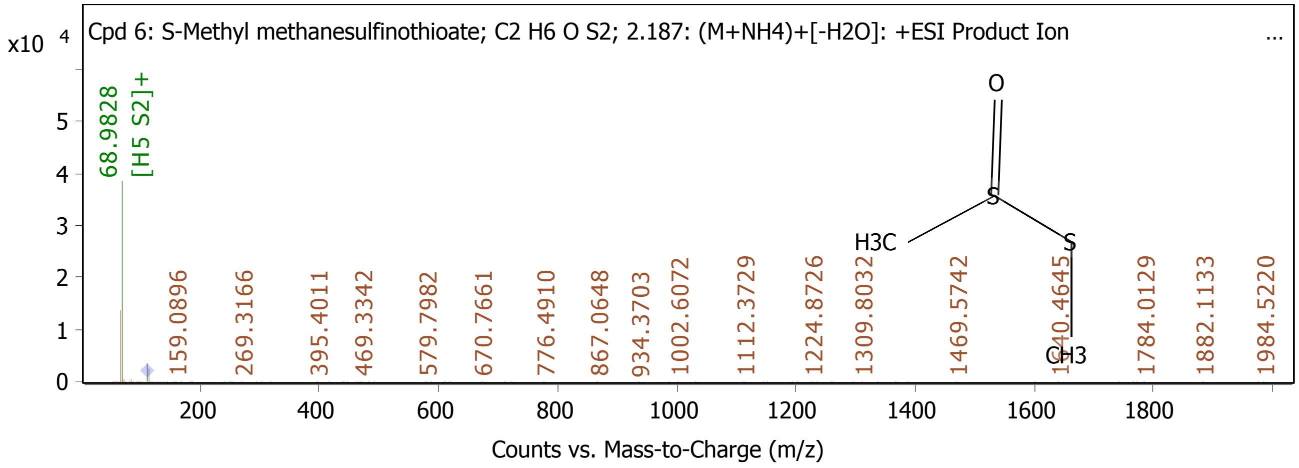

Identification Hit Table

Best Hit	Compound Name	RT	Formula	Notes	Match Score	Mass	Difference	Ion Species
✓	S-Methyl methanesulfinothioate	2.187	C2 H6 O S2		73.66	109.9861	-0.05	(M+NH4)+[-H2O]

Identification Hit Table

Best Hit	Compound Name	RT	Formula	Notes	Match Score	Mass	Difference	Ion Species

Identification Hit Table

Best Hit	Compound Name	RT	Formula	Notes	Match Score	Mass	Difference	Ion Species

Compound Label	Name	*m/z*	RT	Algorithm	Mass
Cpd 7: 1-Isothiocyanato-2-(methylthio)ethane; C4 H7 N S2; 2.188	**1-Isothiocyanato-2-(methylthio)ethane**	151.0354	2.188	Find by Molecular Feature	133.0018

Compound Chromatograms

Qualitative Compound Identification Report

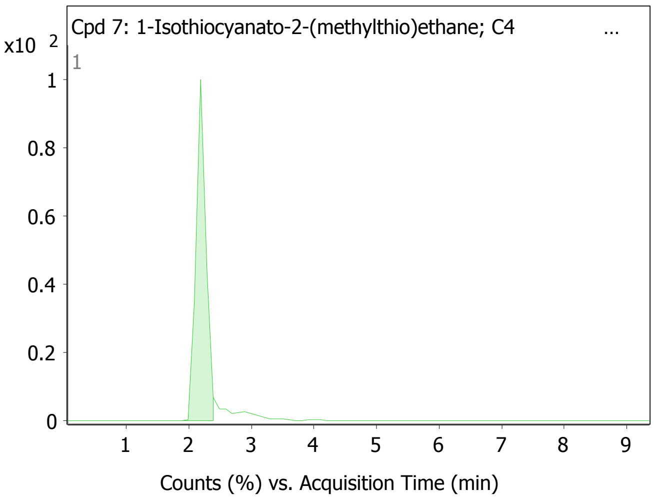

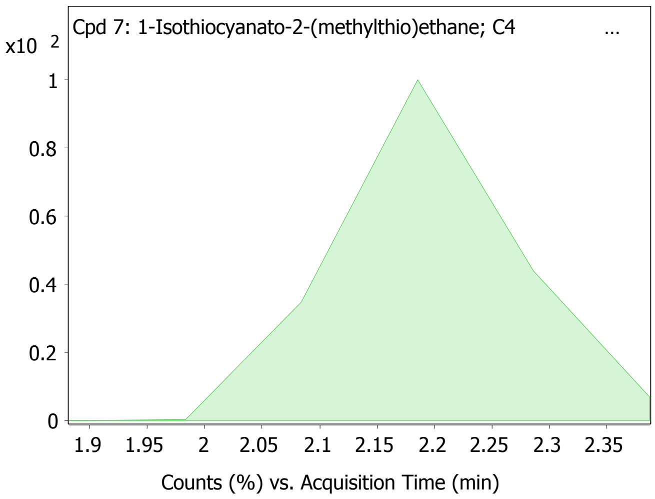

MFE MS Spectrum

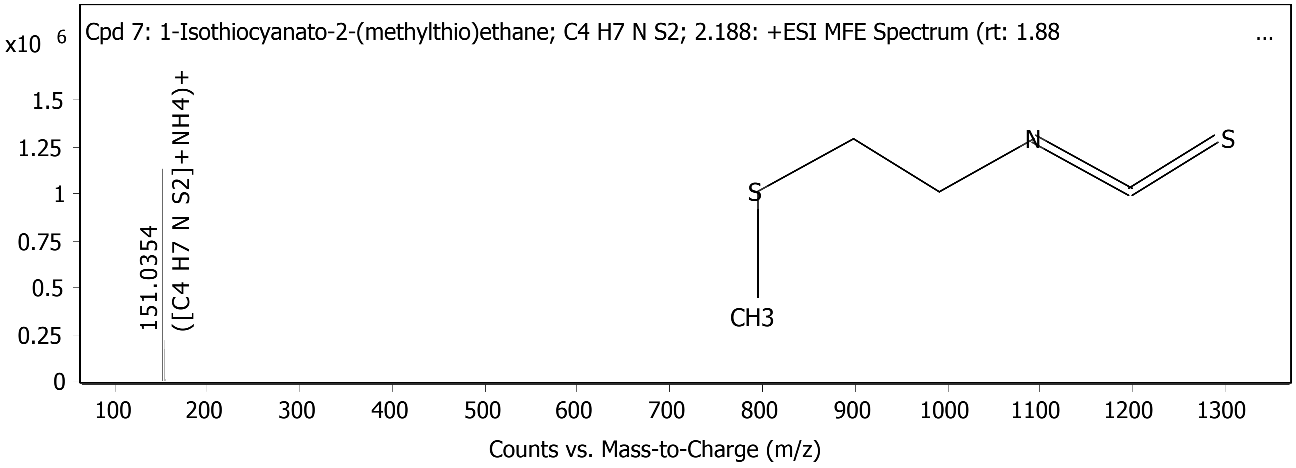

MFE MS Zoomed Spectrum

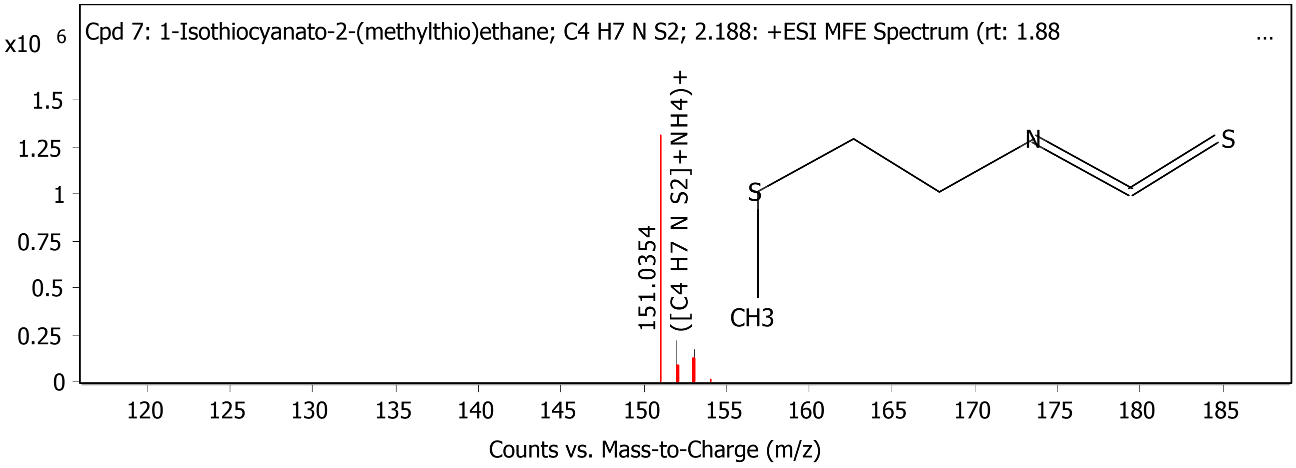

MS Spectrum

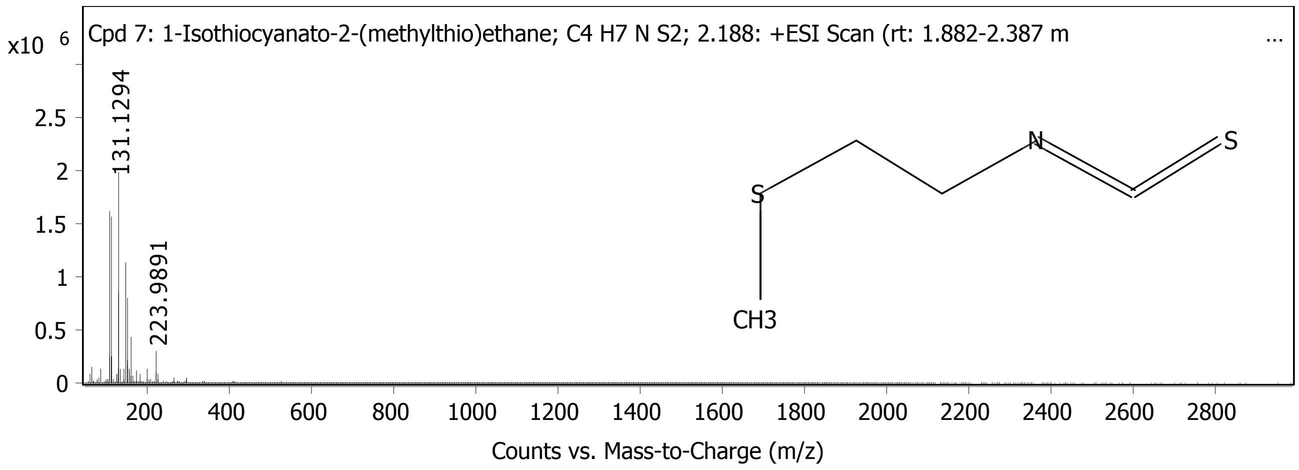

MS Zoomed Spectrum

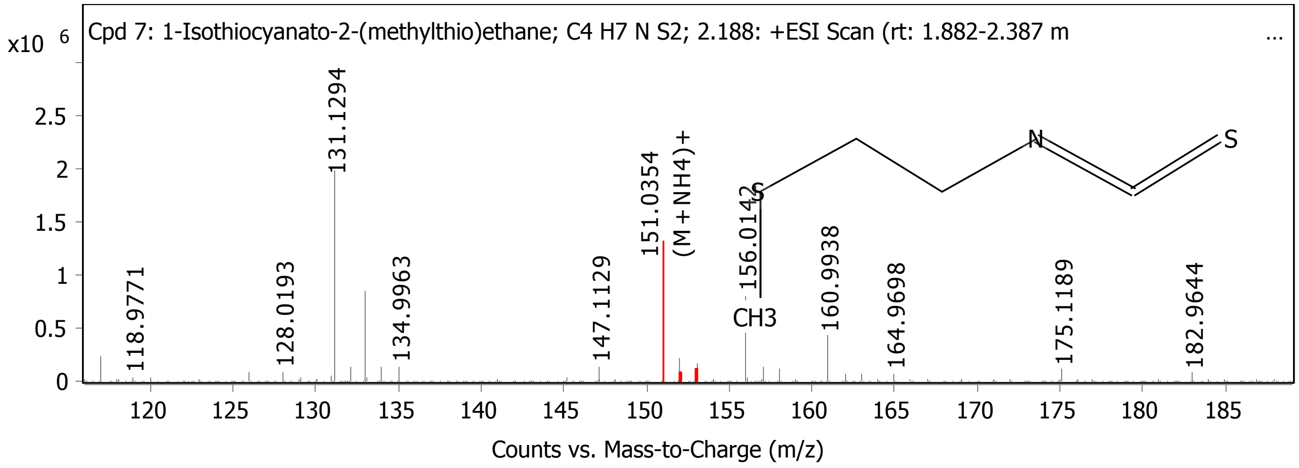

Identification Hit Table

Best Hit	Compound Name	RT	Formula	Notes	Match Score	Mass	Difference	Ion Species
✓	1-Isothiocyanato-2-(methylthio)ethane	2.188	C4 H7 N S2		74.1	133.0018	0.23	(M+NH4)+

Identification Hit Table

Best Hit	Compound Name	RT	Formula	Notes	Match Score	Mass	Difference	Ion Species

Identification Hit Table

Best Hit	Compound Name	RT	Formula	Notes	Match Score	Mass	Difference	Ion Species

Compound Label	Name	*m/z*	RT	Algorithm	Mass
Cpd 8: 4-Guanidino-1-butanol; C5 H13 N3 O; 2.286	**4-Guanidino-1-butanol**	131.1294	2.286	Find by Molecular Feature	131.1061

Compound Chromatograms

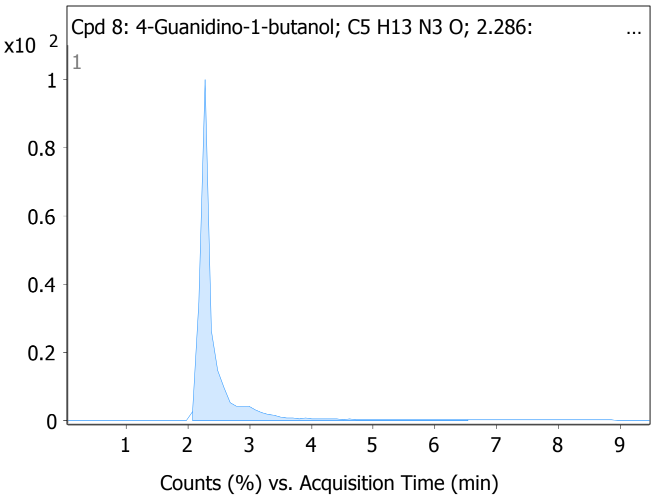

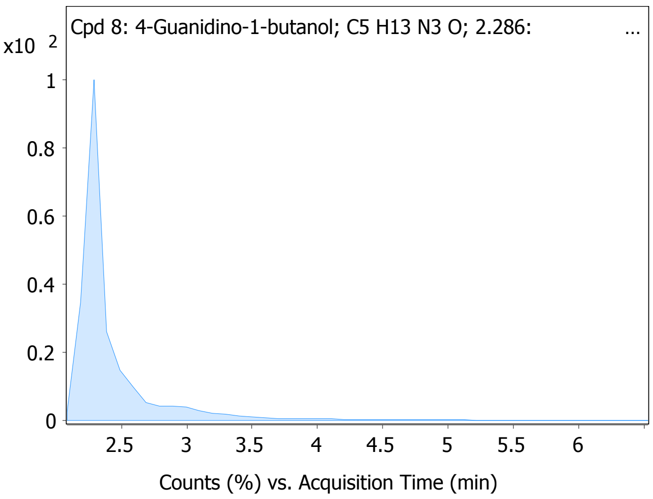

MFE MS Spectrum

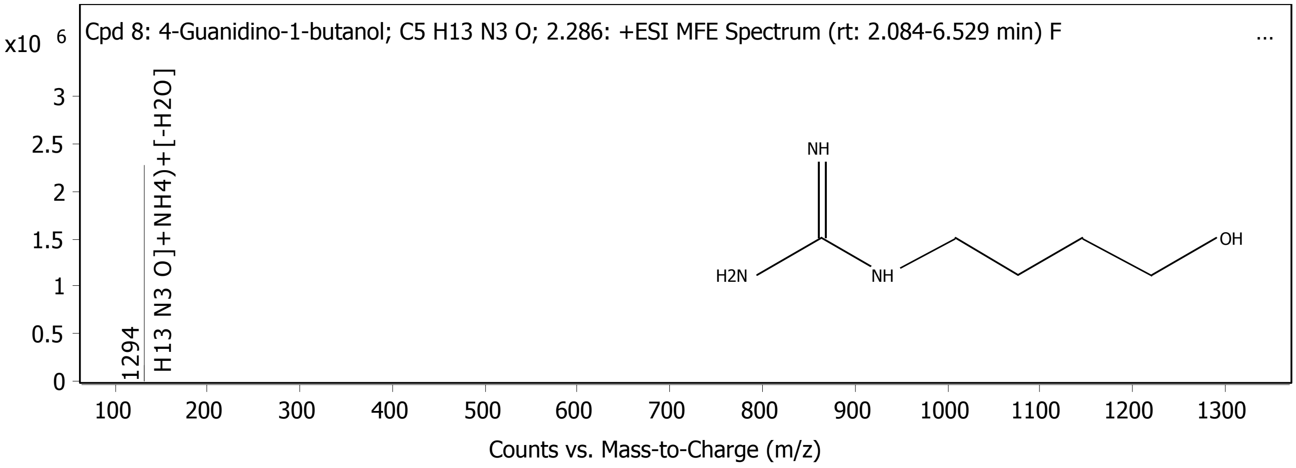

MFE MS Zoomed Spectrum

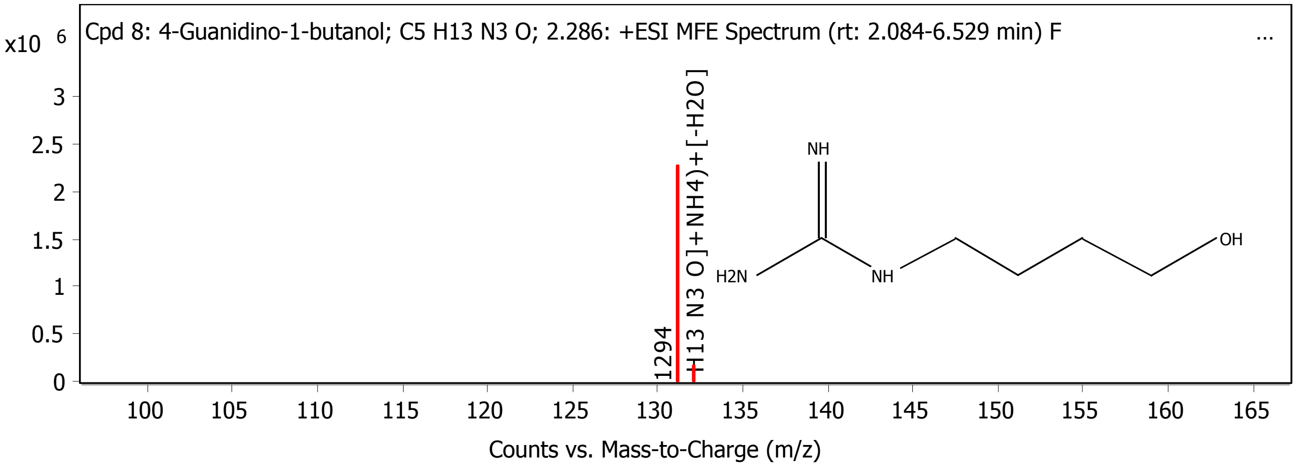

MS Spectrum

Qualitative Compound Identification Report

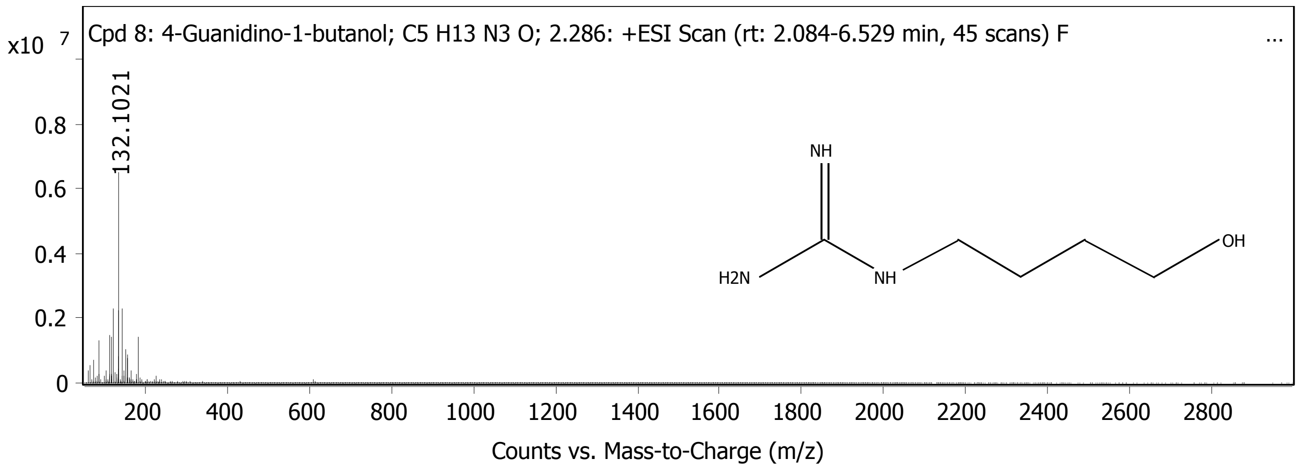

MS Zoomed Spectrum

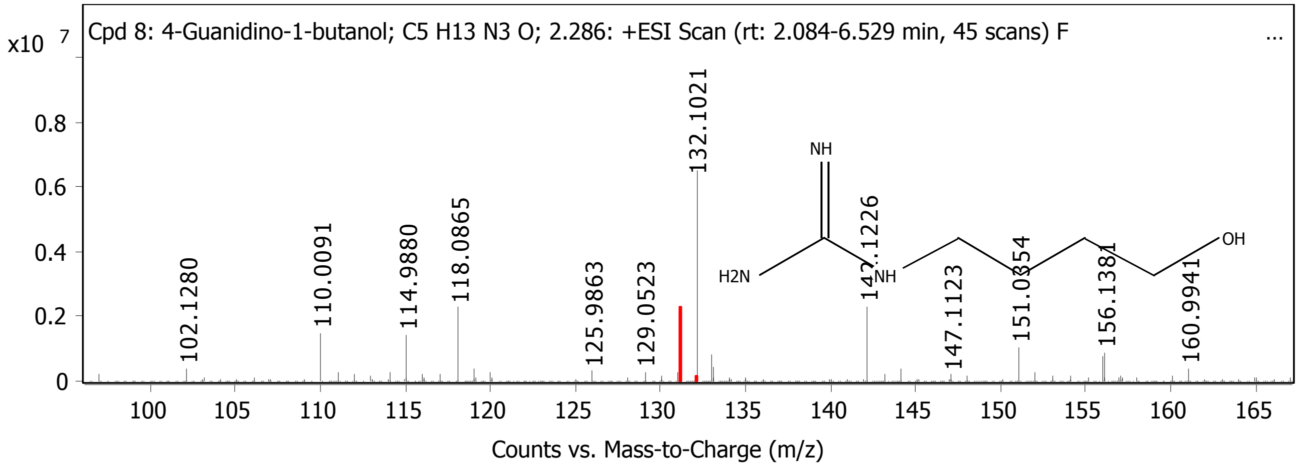

Identification Hit Table

Best Hit	Compound Name	RT	Formula	Notes	Match Score	Mass	Difference	Ion Species
✓	4-Guanidino-1-butanol	2.286	C5 H13 N3 O		87.59	131.1061	-0.27	(M+NH4)+[-H2O]
	N-HBG	2.286	C5 H13 N3 O		87.59	131.1061	-0.27	(M+NH4)+[-H2O]
	N-Carbamoylputrescine	2.286	C5 H13 N3 O		87.59	131.1061	-0.27	(M+NH4)+[-H2O]
	Agmatine	2.286	C5 H14 N4	Postive MS/MS	87.59	130.1221	-0.27	(M+H)+

Identification Hit Table

Best Hit	Compound Name	RT	Formula	Notes	Match Score	Mass	Difference	Ion Species

Identification Hit Table

Best Hit	Compound Name	RT	Formula	Notes	Match Score	Mass	Difference	Ion Species

Compound Label	Name	*m/z*	RT	Algorithm	Mass
Cpd 9: Amino acid(Arg-); C6 H14 N4 O2; 2.405	**Amino acid(Arg-)**	175.1189	2.405	Find by Molecular Feature	174.1117

Compound Chromatograms

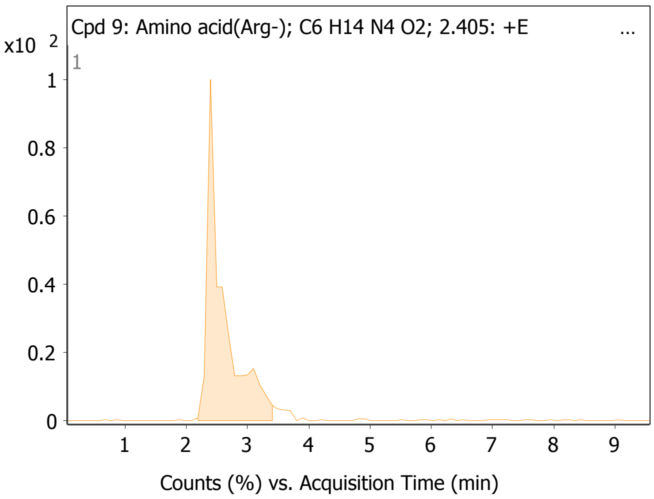

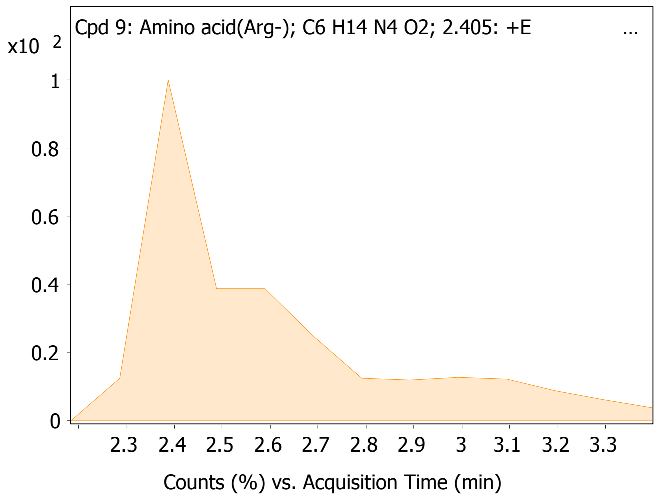

MFE MS Spectrum

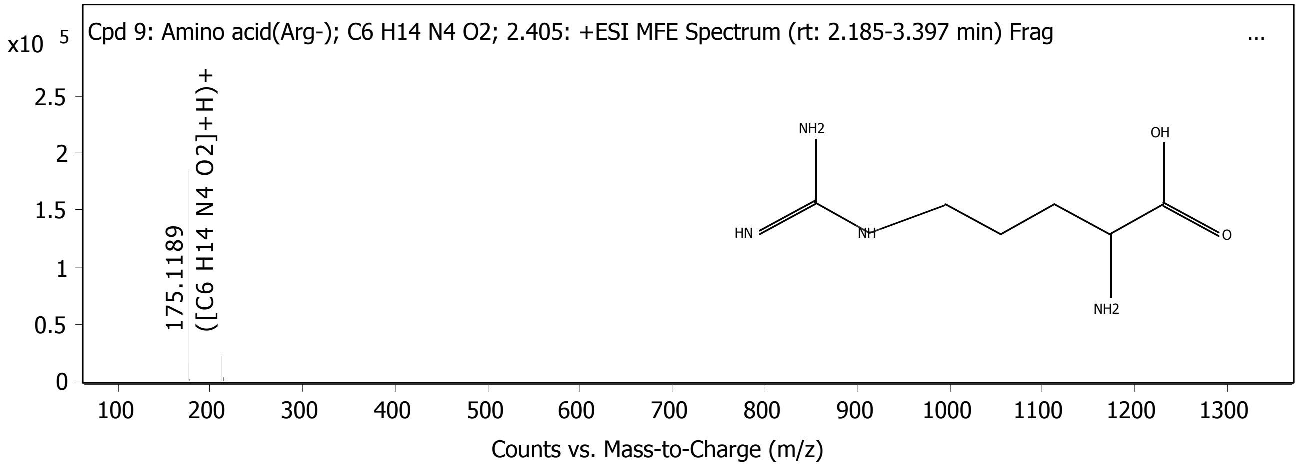

MFE MS Zoomed Spectrum

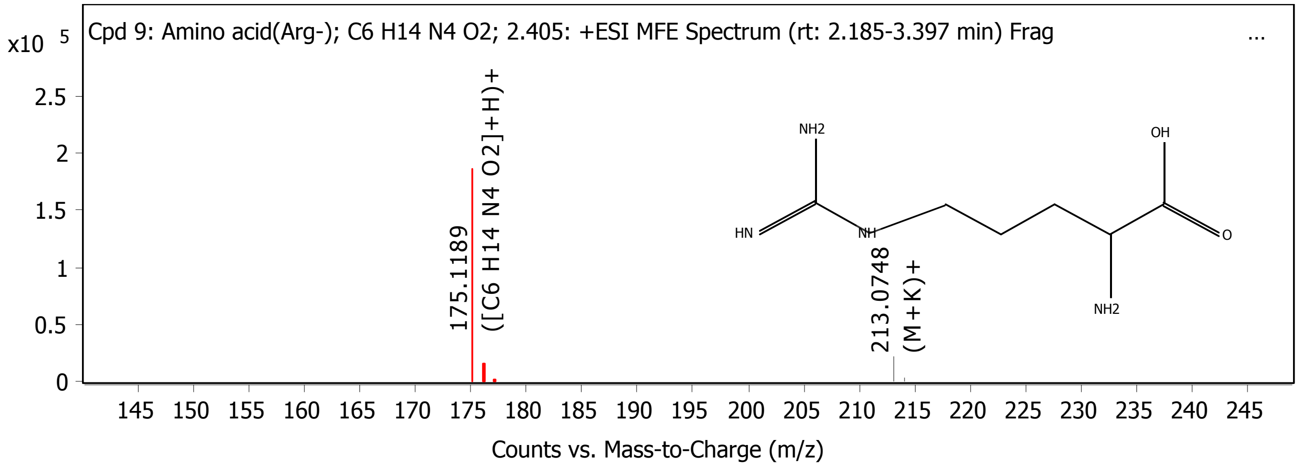

MS Spectrum

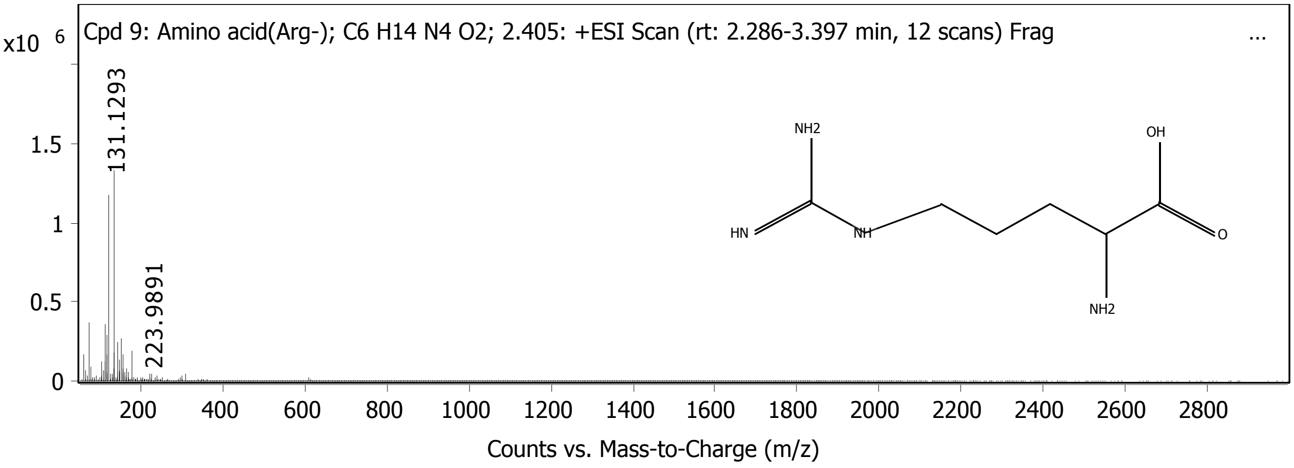

MS Zoomed Spectrum

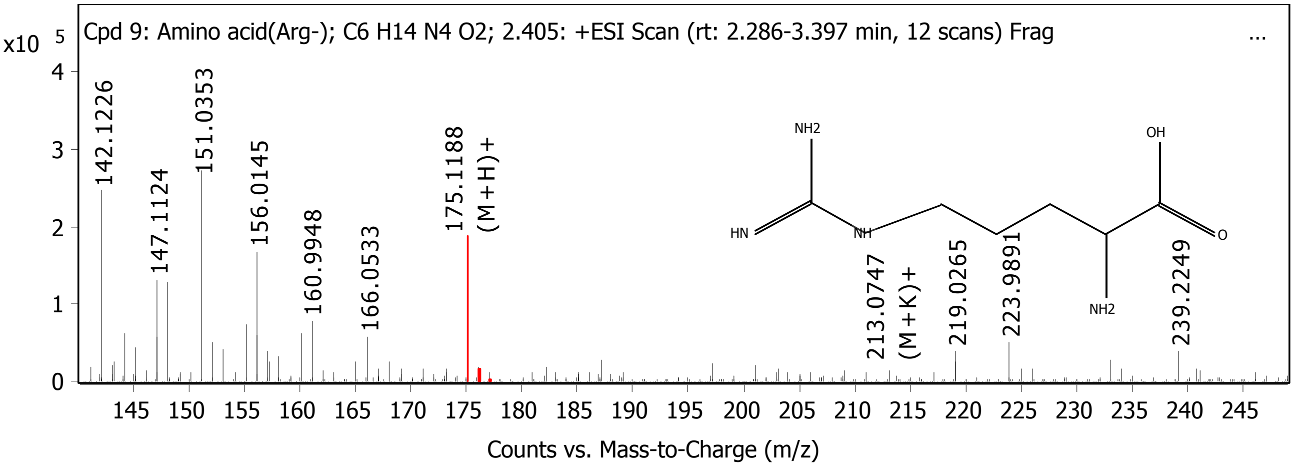

Identification Hit Table

Best Hit	Compound Name	RT	Formula	Notes	Match Score	Mass	Difference	Ion Species
✓	Amino acid(Arg-)	2.405	C6 H14 N4 O2		99.63	174.1117	0.02	(M+H)+
	D-Arginine	2.405	C6 H14 N4 O2		99.63	174.1117	0.02	(M+H)+
	L-Arginine	2.405	C6 H14 N4 O2	Postive MS/MS Endogenous Metabolite Geigy vol. 3 p. 92 Deleted CAS: 142-49-4; 7004-12-8; 154605-63-7; 154605-67-1; 667422-95-9; 1332377-47-5	99.63	174.1117	0.02	(M+H)+

Identification Hit Table

Best Hit	Compound Name	RT	Formula	Notes	Match Score	Mass	Difference	Ion Species

Identification Hit Table

Best Hit	Compound Name	RT	Formula	Notes	Match Score	Mass	Difference	Ion Species

Compound Label	Name	*m/z*	RT	Algorithm	Mass
Cpd 10: Ethyl lactate; C5 H10 O3; 2.912	**Ethyl lactate**	118.0866	2.912	Find by Molecular Feature	118.0634

Compound Chromatograms

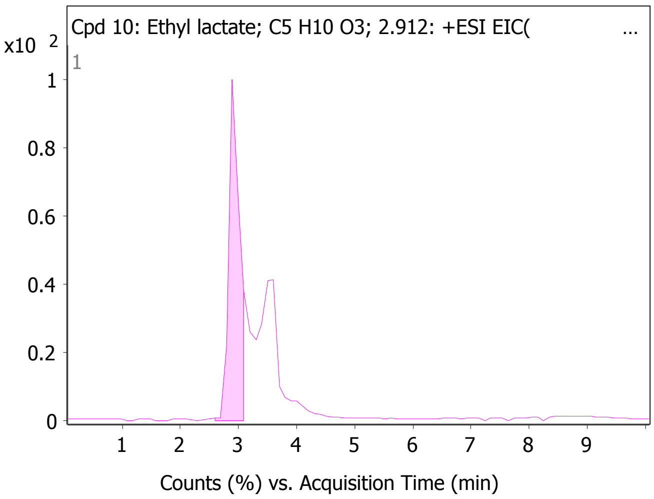

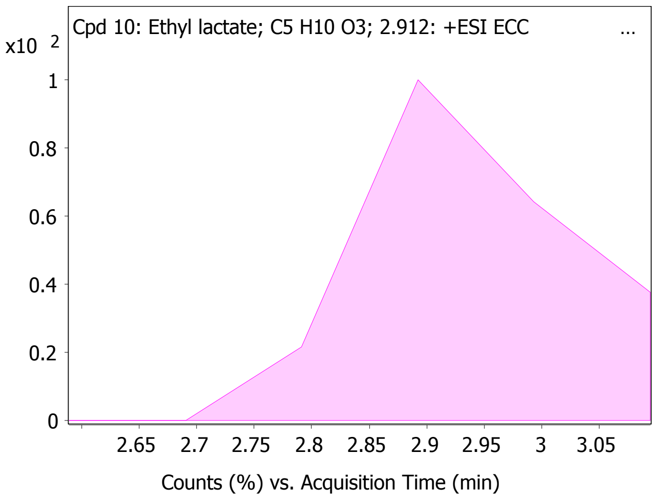

MFE MS Spectrum

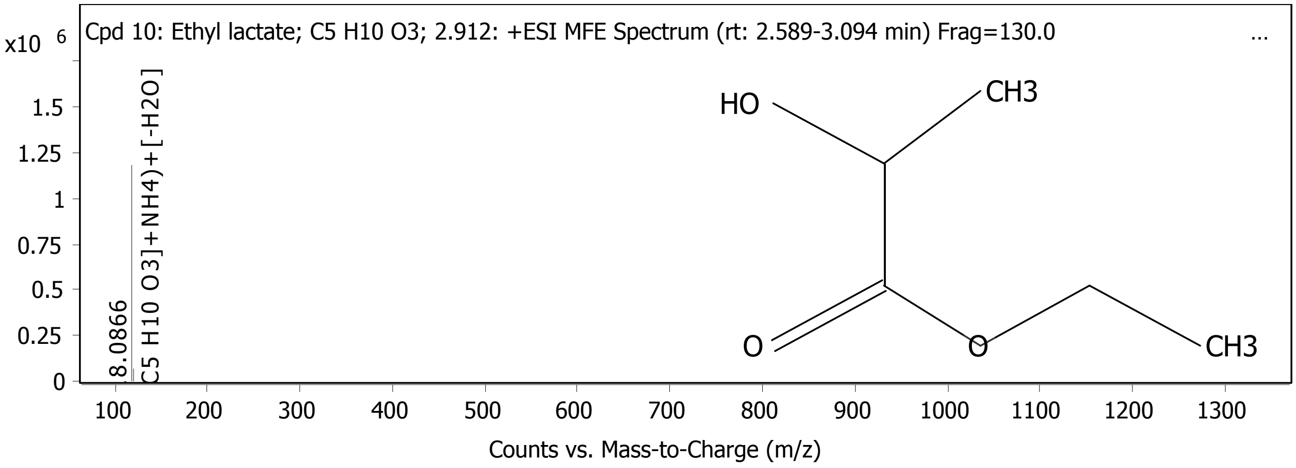

MFE MS Zoomed Spectrum

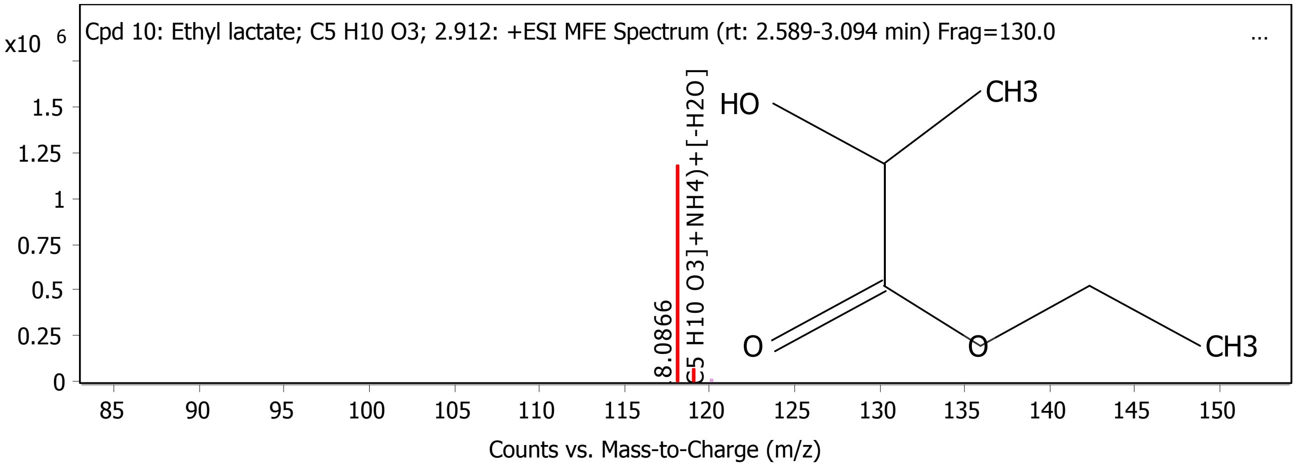

MS Spectrum

Qualitative Compound Identification Report

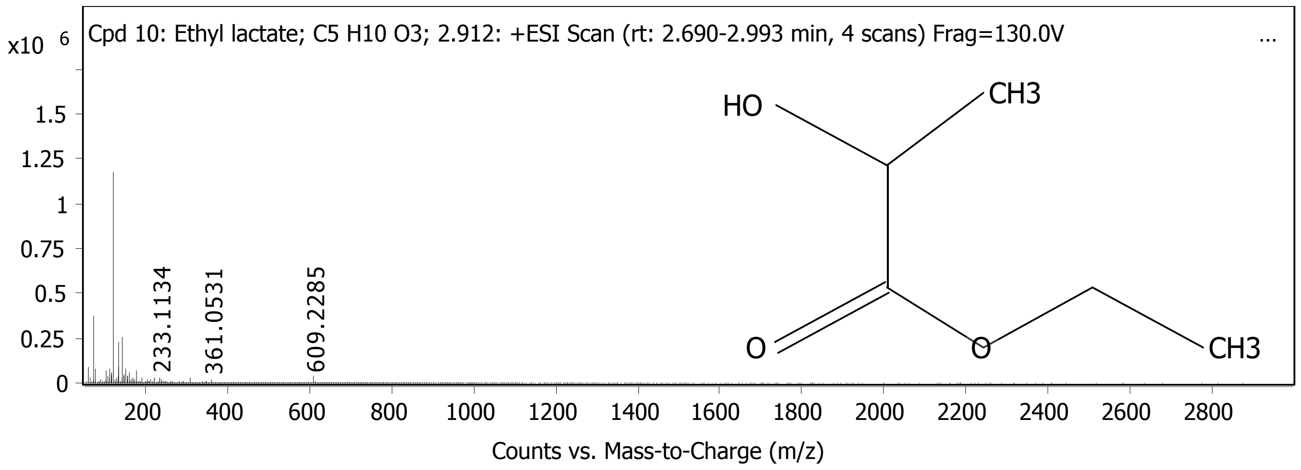

MS Zoomed Spectrum

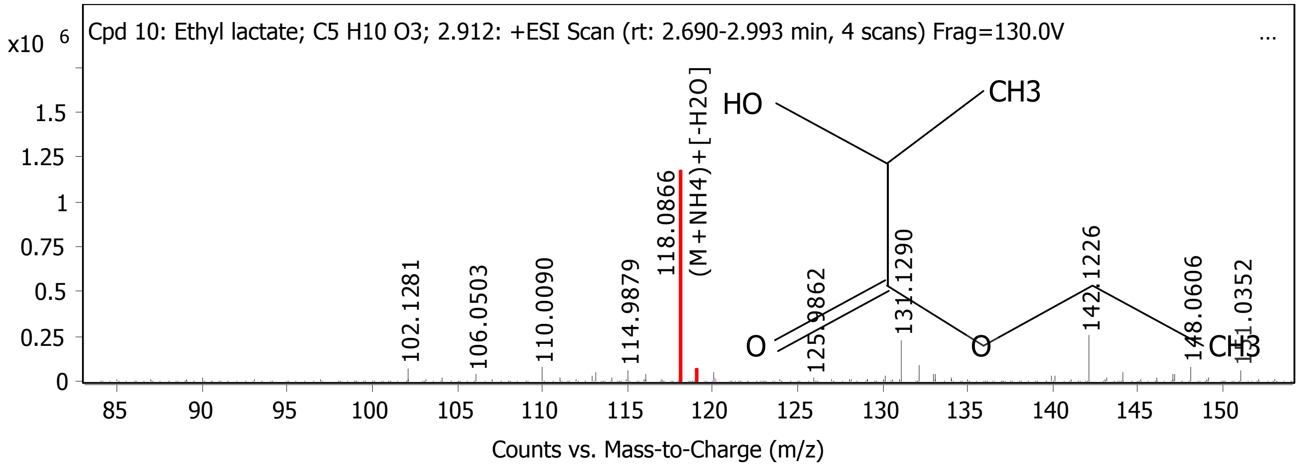

MSMS Spectrum

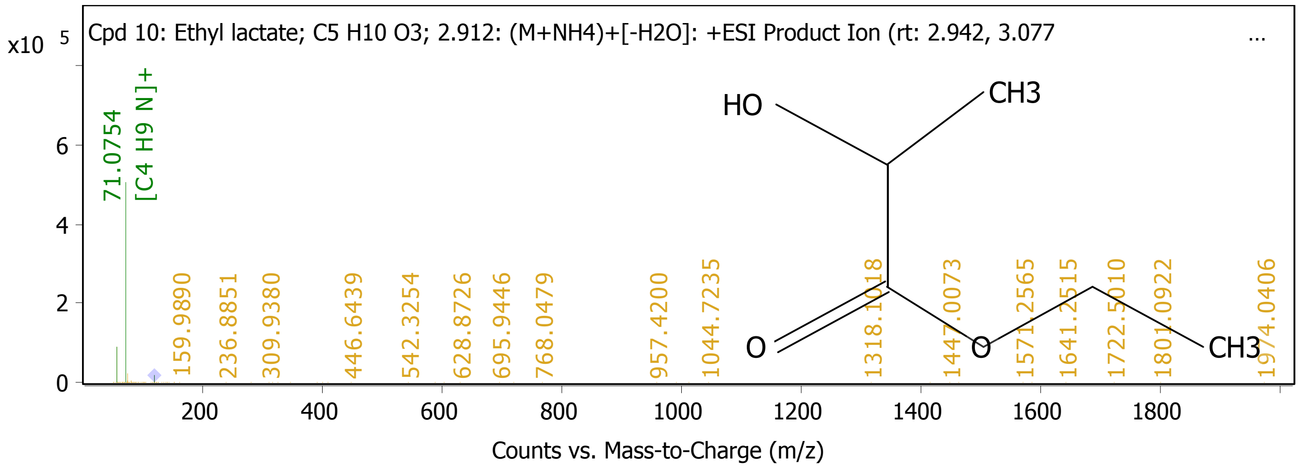

Identification Hit Table

Best Hit	Compound Name	RT	Formula	Notes	Match Score	Mass	Difference	Ion Species
✓	Ethyl lactate	2.912	C5 H10 O3		87.01	118.0634	-0.37	(M+NH4)+[-H2O]
	4-hydroxy-valeric acid	2.912	C5 H10 O3		87.01	118.0634	-0.37	(M+NH4)+[-H2O]
	(S)-2-Ethyl-3-hydroxypropionic acid	2.912	C5 H10 O3	2-Ethylhydracrylic acid PID: 766484	87.01	118.0634	-0.37	(M+NH4)+[-H2O]
	2-hydroxy-3-methyl-butyric acid	2.912	C5 H10 O3		87.01	118.0634	-0.37	(M+NH4)+[-H2O]
	(+/-)-alpha-Hydroxyisovaleric acid	2.912	C5 H10 O3	NMR Spectroscopy Of Biofluids. J.C. Lindon, J.K. Nicholson, J.E. Everett CSF, Urine	87.01	118.0634	-0.37	(M+NH4)+[-H2O]
	DL-a-Hydroxyvaleric acid	2.912	C5 H10 O3	Nugo Website Http://Nugo.org/metabolomics/13 496 Urine	87.01	118.0634	-0.37	(M+NH4)+[-H2O]
	2-Methyl-3-hydroxybutyric acid	2.912	C5 H10 O3	Nugo Website	87.01	118.0634	-0.37	(M+NH4)+[-H2O]

				Http://Nugo.org/metabolomics/13 496 Urine				
	β-Hydroxyisovaleric acid	2.912	C5 H10 O3	Nugo Website Http://Nugo.org/metabolomics/13 496 Urine	87.01	118.0634	-0.37	(M+NH4)+[-H2O]
	L-α-Hydroxyisovaleric acid	2.912	C5 H10 O3		87.01	118.0634	-0.37	(M+NH4)+[-H2O]
	3-Hydroxy-2-methyl-[R-(R,S)]-butanoic acid	2.912	C5 H10 O3		87.01	118.0634	-0.37	(M+NH4)+[-H2O]

Identification Hit Table

Best Hit	Compound Name	RT	Formula	Notes	Match Score	Mass	Difference	Ion Species
✓	DL-2-hydroxy valeric acid	2.912	C5 H10 O3		87.01	118.0634	-0.37	(M+NH4)+[-H2O]
	4-hydroxy-valeric acid	2.912	C5 H10 O3		87.01	118.0634	-0.37	(M+NH4)+[-H2O]
	5-hydroxy valeric acid	2.912	C5 H10 O3		87.01	118.0634	-0.37	(M+NH4)+[-H2O]
	2-ethyl-hydracrylic acid	2.912	C5 H10 O3		87.01	118.0634	-0.37	(M+NH4)+[-H2O]
	2-hydroxy-2-methyl-butyric acid	2.912	C5 H10 O3		87.01	118.0634	-0.37	(M+NH4)+[-H2O]
	2-hydroxy-3-methyl-butyric acid	2.912	C5 H10 O3		87.01	118.0634	-0.37	(M+NH4)+[-H2O]
	2-Methyl-3-hydroxybutyric acid	2.912	C5 H10 O3	Nugo Website Http://Nugo.org/metabolomics/13 496 Urine	87.01	118.0634	-0.37	(M+NH4)+[-H2O]
	3-Hydroxy-2-methyl-[S-(R,R)]-butanoic acid	2.912	C5 H10 O3		87.01	118.0634	-0.37	(M+NH4)+[-H2O]
	β-Hydroxyisovaleric acid	2.912	C5 H10 O3	Nugo Website Http://Nugo.org/metabolomics/13 496 Urine	87.01	118.0634	-0.37	(M+NH4)+[-H2O]
	4-hydroxy-isovaleric acid	2.912	C5 H10 O3		87.01	118.0634	-0.37	(M+NH4)+[-H2O]

Identification Hit Table

Best Hit	Compound Name	RT	Formula	Notes	Match Score	Mass	Difference	Ion Species

Compound Label	Name	*m/z*	RT	Algorithm	Mass
Cpd 11: Dimethylethanolamine; C4 H11 N O; 2.916	**Dimethylethanolamine**	72.0817	2.916	Find by Molecular Feature	89.085

Compound Chromatograms

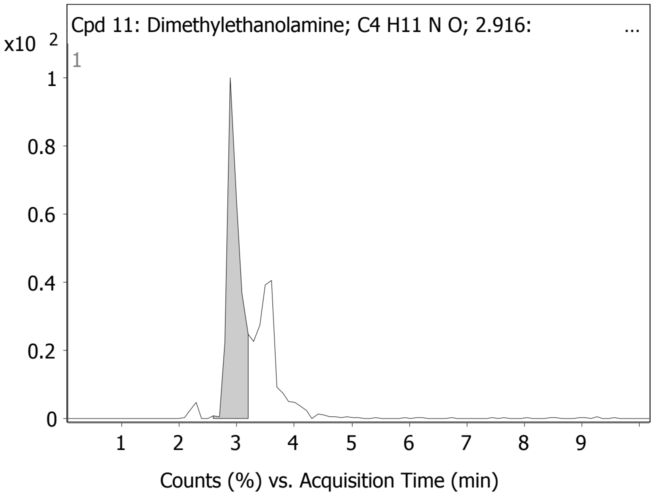

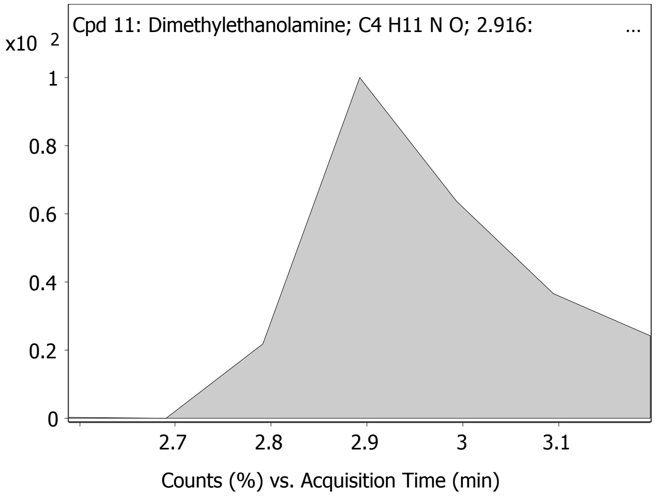

MFE MS Spectrum

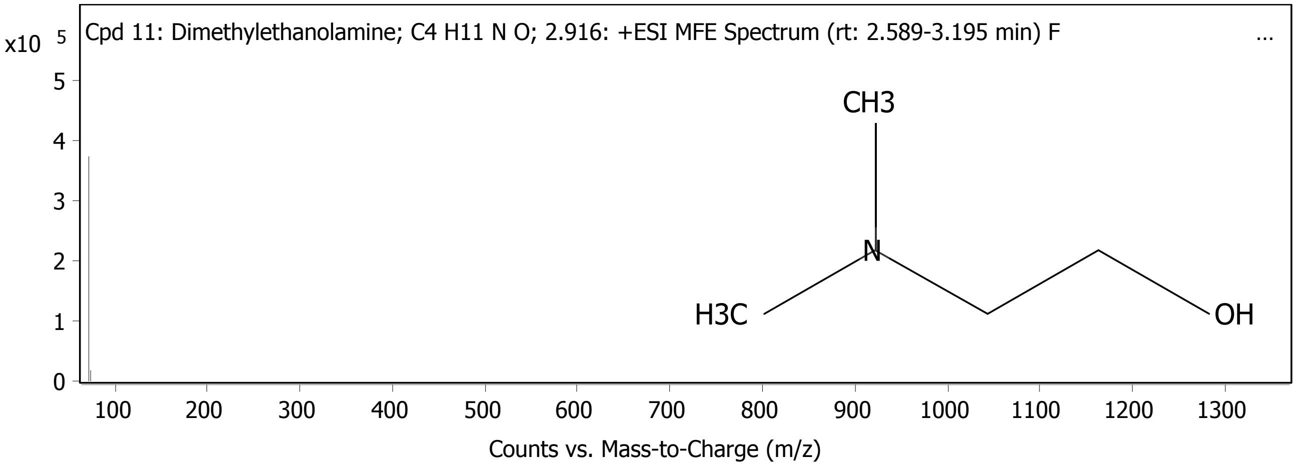

MFE MS Zoomed Spectrum

Qualitative Compound Identification Report

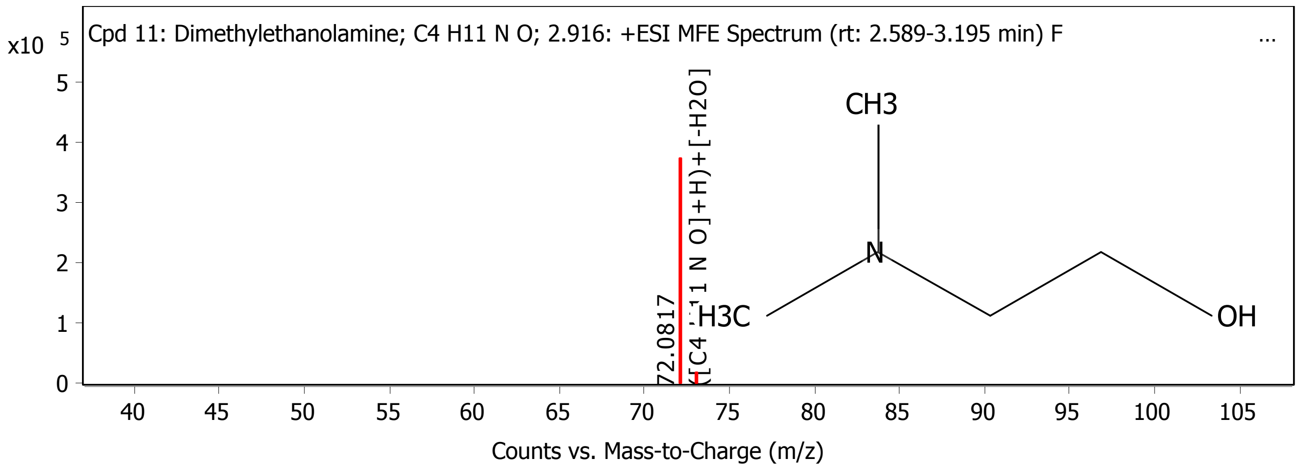

MS Spectrum

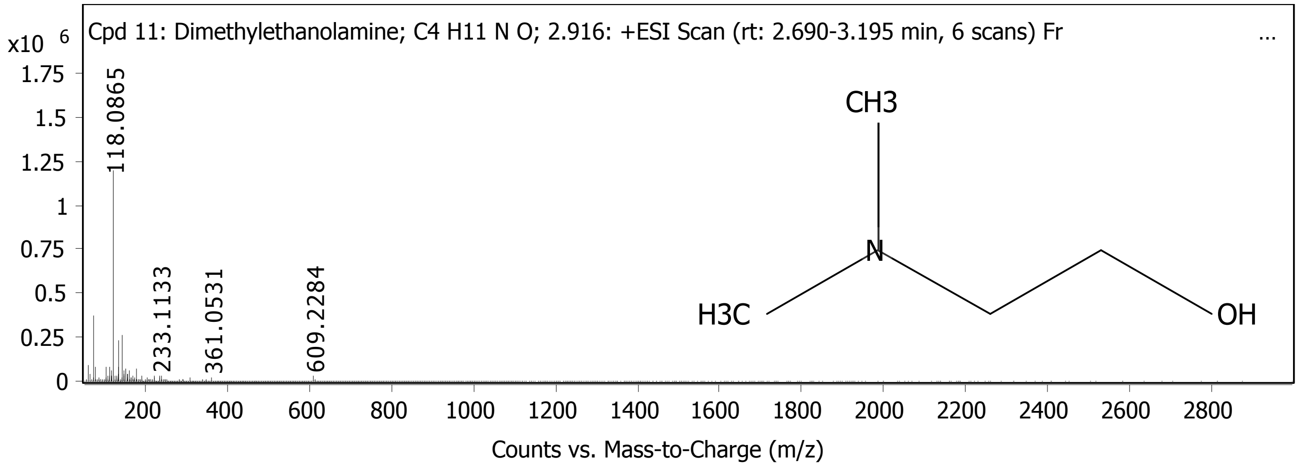

MS Zoomed Spectrum

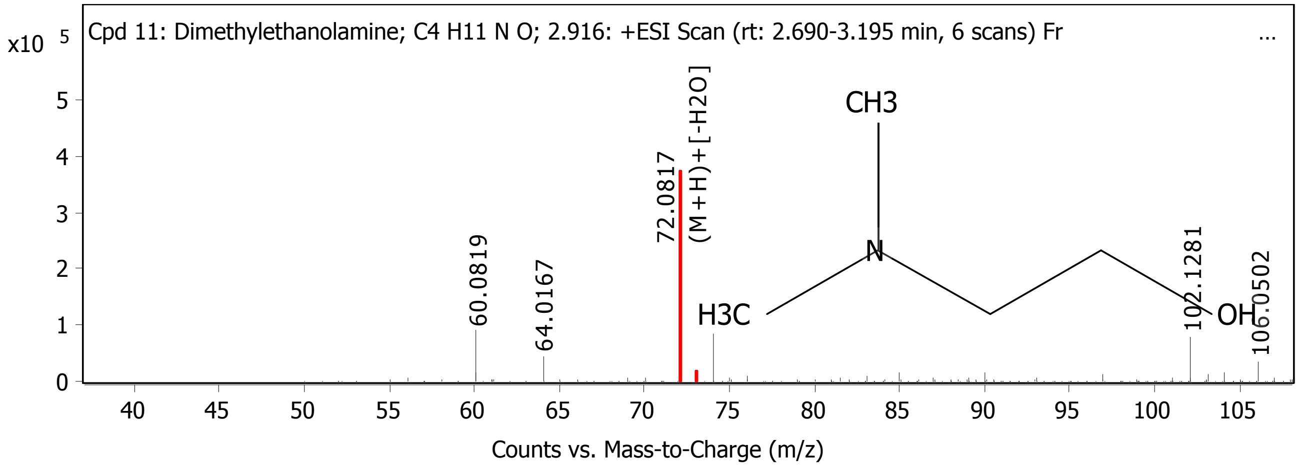

MSMS Spectrum

Qualitative Compound Identification Report

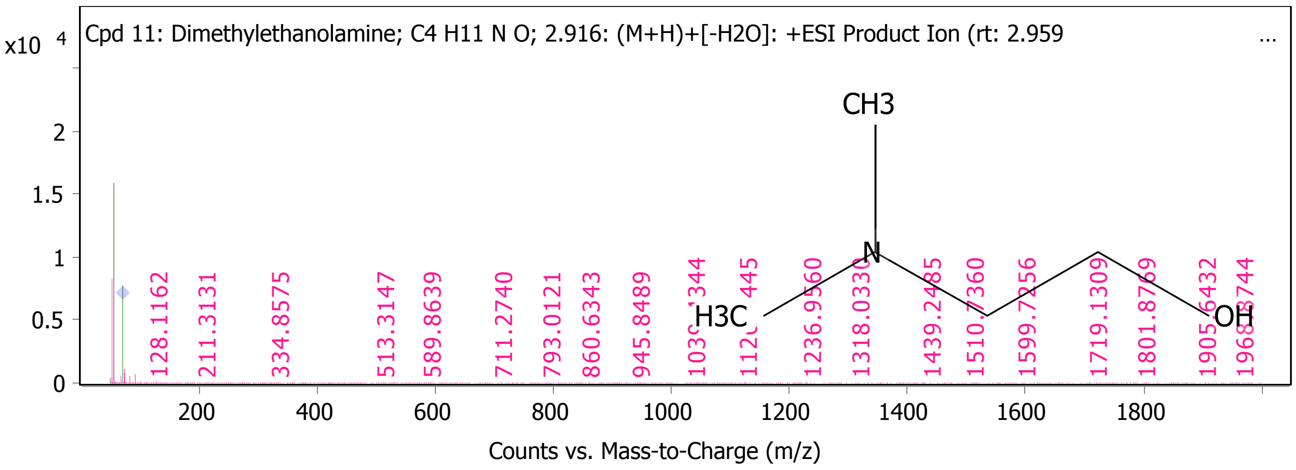

Identification Hit Table

Best Hit	Compound Name	RT	Formula	Notes	Match Score	Mass	Difference	Ion Species
✓	Dimethylethanolamine	2.916	C4 H11 N O		81.44	89.085	-0.97	(M+H)+[-H2O]
	2-Amino-2-methyl-1-propanol	2.916	C4 H11 N O	â-Aminoisobutyl alcohol, AMP	81.44	89.085	-0.97	(M+H)+[-H2O]
	2,2-Dimethyloxirane	2.916	C4 H8 O		80.95	72.0585	-0.97	(M+NH4)+[-H2O]
	2-Buten-1-ol	2.916	C4 H8 O		80.95	72.0585	-0.97	(M+NH4)+[-H2O]
	3-Buten-1-ol	2.916	C4 H8 O		80.95	72.0585	-0.97	(M+NH4)+[-H2O]
	1,2-Epoxybutane	2.916	C4 H8 O		80.95	72.0585	-0.97	(M+NH4)+[-H2O]
	Butanal	2.916	C4 H8 O		80.95	72.0585	-0.97	(M+NH4)+[-H2O]
	Tetrahydrofuran	2.916	C4 H8 O		80.95	72.0585	-0.97	(M+NH4)+[-H2O]
	Butanone	2.916	C4 H8 O		80.95	72.0585	-0.97	(M+NH4)+[-H2O]
	Pyrrolidine	2.916	C4 H9 N		80.91	71.0745	-0.97	(M+H)+

Identification Hit Table

Best Hit	Compound Name	RT	Formula	Notes	Match Score	Mass	Difference	Ion Species

Identification Hit Table

Best Hit	Compound Name	RT	Formula	Notes	Match Score	Mass	Difference	Ion Species

Compound Label	Name	*m/z*	RT	Algorithm	Mass
Cpd 12: L-Valine; C5 H11 N O2; 3.502	**L-Valine**	118.0864	3.502	Find by Molecular Feature	117.0791

Compound Chromatograms

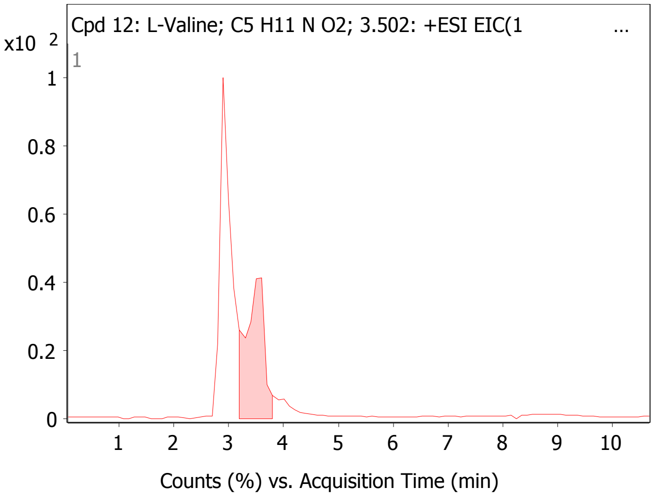

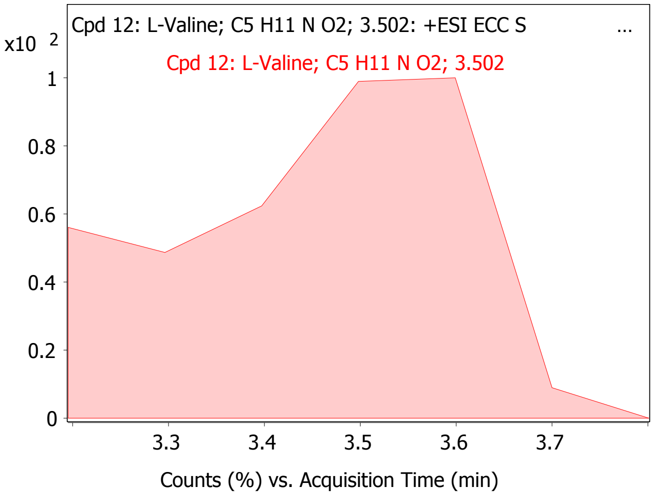

MFE MS Spectrum

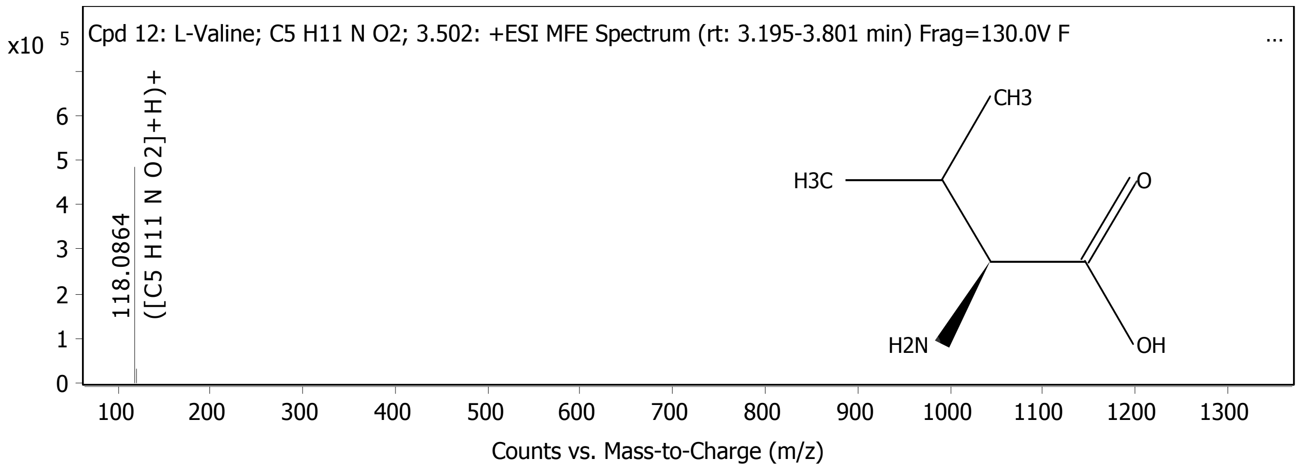

MFE MS Zoomed Spectrum

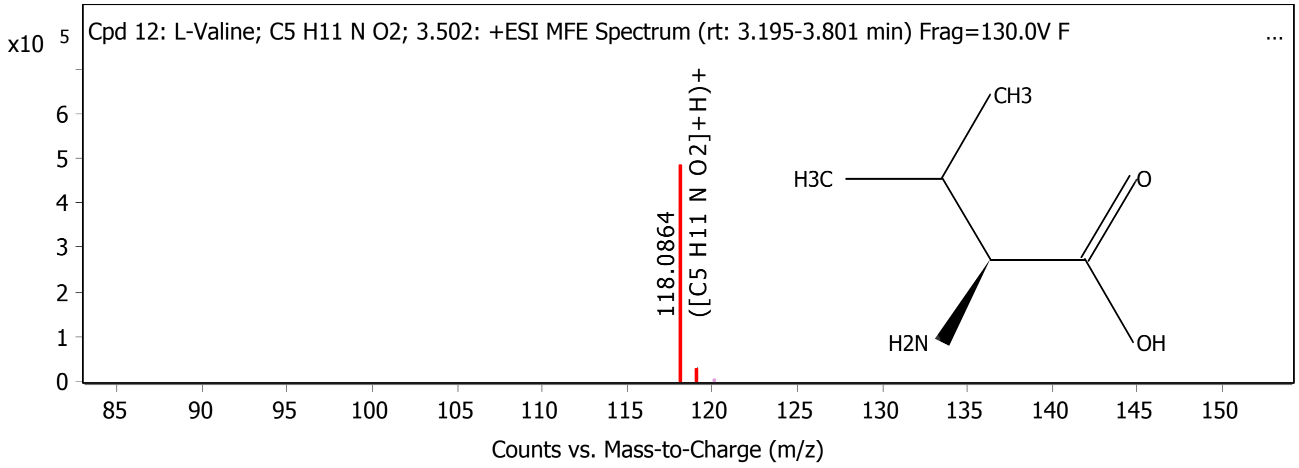

MS Spectrum

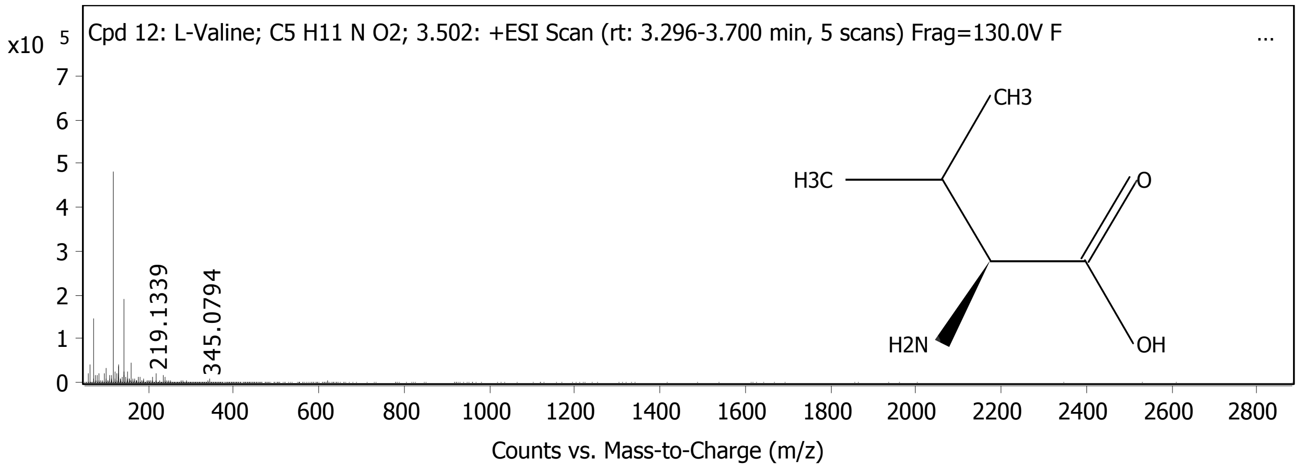

MS Zoomed Spectrum

Qualitative Compound Identification Report

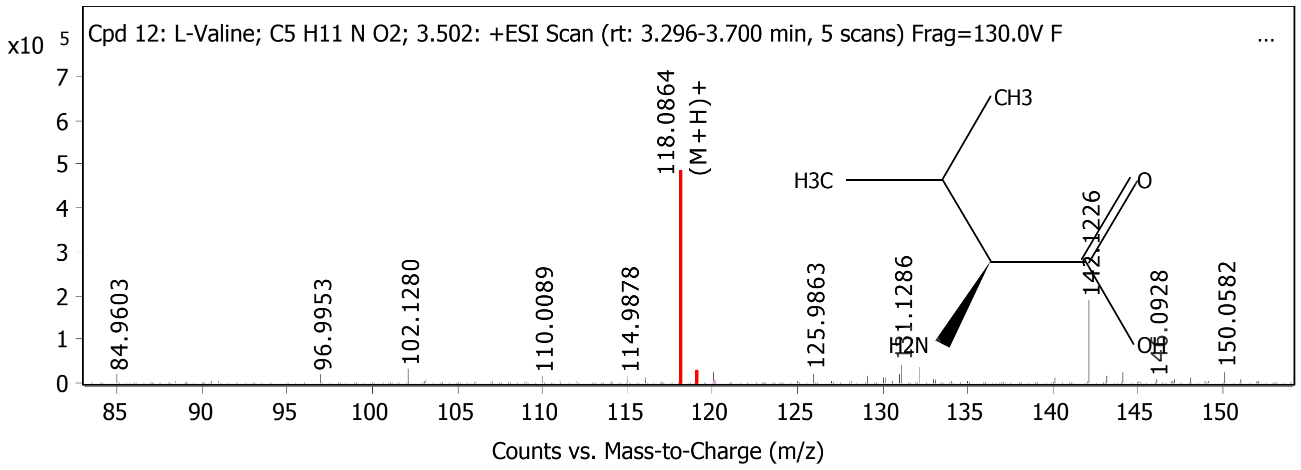

Library Spectrum

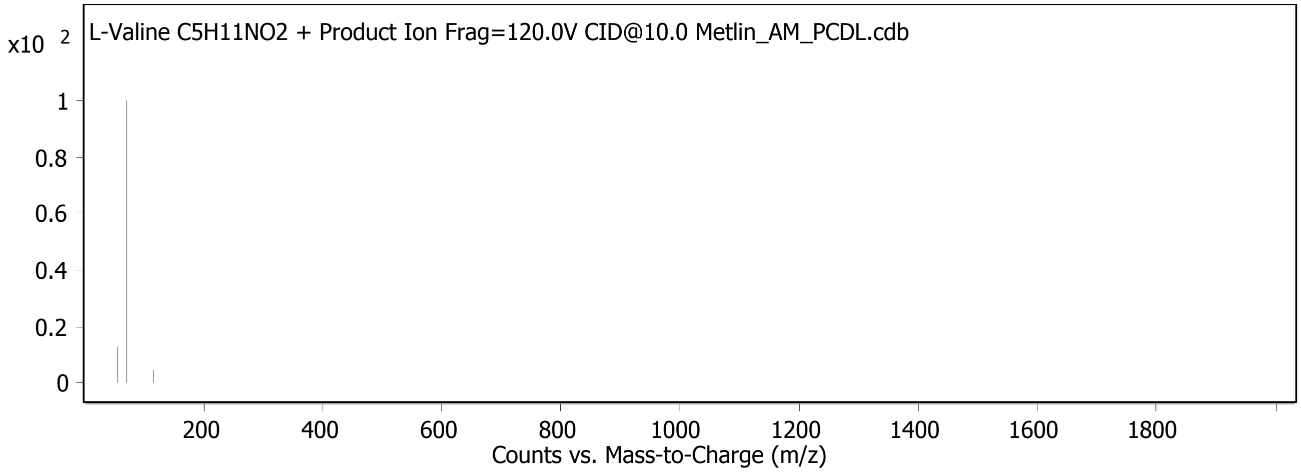

Difference Spectrum

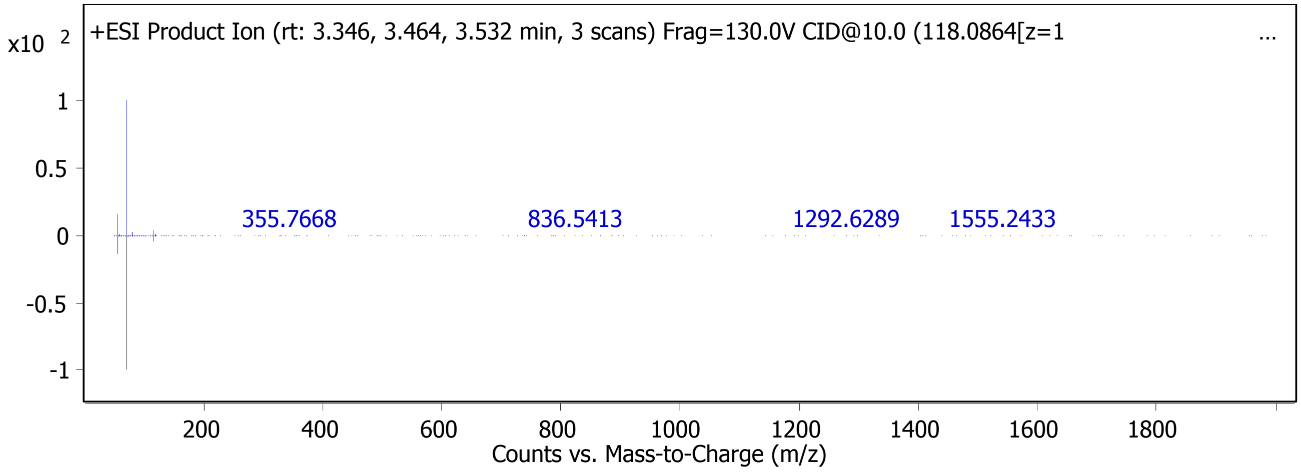

MSMS Spectrum

Qualitative Compound Identification Report

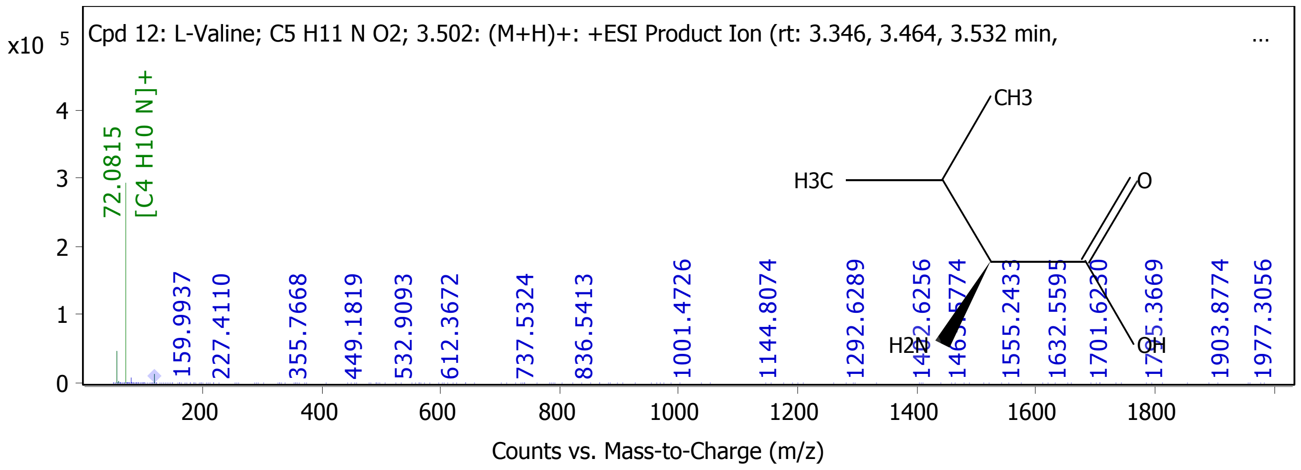

Identification Hit Table

Best Hit	Compound Name	RT	Formula	Notes	Match Score	Mass	Difference	Ion Species
✓	L-Valine	3.502	C5 H11 N O2	Postive MS/MS Endogenous Metabolite Geigy vol. 3 p. 92	87.7	117.0791	-0.16	(M+H)+
	4-amino-pentanoic acid	3.502	C5 H11 N O2		87.7	117.0791	-0.16	(M+H)+
	2-Amino-2-methylbutanoate	3.502	C5 H11 N O2		87.7	117.0791	-0.16	(M+H)+
	L-Norvaline	3.502	C5 H11 N O2		87.7	117.0791	-0.16	(M+H)+
	Norvaline	3.502	C5 H11 N O2		87.7	117.0791	-0.16	(M+H)+
	2-Amino-3-methylbutanoic acid	3.502	C5 H11 N O2		87.7	117.0791	-0.16	(M+H)+
	Pentanoic acid, 4-amino-, (1)-; Valeric acid, 4-amino-, (1)-; (1)-4-Aminopentanoic acid; (RS)-4-Aminopentanoic acid; (RS)-4-Methyl-gamma-aminobutyric acid	3.502	C5 H11 N O2		87.7	117.0791	-0.16	(M+H)+
	Pentanoic acid, 4-amino-, (S)-; (S)-4-Aminopentanoic acid; (S)-4-Aminovaleric acid	3.502	C5 H11 N O2		87.7	117.0791	-0.16	(M+H)+
	Pentanoic acid, 4-amino-, (R)-; Valeric acid, 4-amino-, (R)-(+)-; (R)-(+)-4-Aminovaleric acid; (R)-4-Aminopentanoic acid	3.502	C5 H11 N O2		87.7	117.0791	-0.16	(M+H)+
	Valine	3.502	C5 H11 N O2		87.7	117.0791	-0.16	(M+H)+

Identification Hit Table

Best Hit	Compound Name	RT	Formula	Notes	Match Score	Mass	Difference	Ion Species
✓	2-Amino-3-methylbutanoic acid	3.502	C5 H11 N O2		87.7	117.0791	-0.16	(M+H)+
	2S-amino-pentanoic acid	3.502	C5 H11 N O2		87.7	117.0791	-0.16	(M+H)+
	5-Aminopentanoic acid	3.502	C5 H11 N O2		87.7	117.0791	-0.16	(M+H)+
	Pentanoic acid, 4-amino-, (1)-; Valeric acid, 4-amino-, (1)-; (1)-4-Aminopentanoic acid; (RS)-4-Aminopentanoic acid; (RS)-4-Methyl-gamma-aminobutyric acid	3.502	C5 H11 N O2		87.7	117.0791	-0.16	(M+H)+
	Pentanoic acid, 4-amino-, (S)-; (S)-4-Aminopentanoic acid; (S)-4-Aminovaleric acid	3.502	C5 H11 N O2		87.7	117.0791	-0.16	(M+H)+
	Pentanoic acid, 4-amino-, (R)-; Valeric acid, 4-amino-, (R)-(+)-; (R)-(+)-4-Aminovaleric acid; (R)-4-Aminopentanoic acid	3.502	C5 H11 N O2		87.7	117.0791	-0.16	(M+H)+
	β-Hydroxyisovaleric acid	3.502	C5 H10 O3	Nugo Website Http://Nugo.org/metabolomics/13496 Urine	87.7	118.0632	-0.16	(M+NH4)+[-H2O]
	3-hydroxy valeric acid	3.502	C5 H10 O3		87.7	118.0632	-0.16	(M+NH4)+[-H2O]
	4-hydroxy-isovaleric acid	3.502	C5 H10 O3		87.7	118.0632	-0.16	(M+NH4)+[-H2O]
	DL-2-hydroxy valeric acid	3.502	C5 H10 O3		87.7	118.0632	-0.16	(M+NH4)+[-H2O]

Identification Hit Table

Best Hit	Compound Name	RT	Formula	Notes	Match Score	Mass	Difference	Ion Species

Compound Label	Name	*m/z*	RT	Algorithm	Mass
Cpd 13: Dimethylethanolamine; C4 H11 N O; 3.544	**Dimethylethanolamine**	72.0816	3.544	Find by Molecular Feature	89.0849

Compound Chromatograms

Qualitative Compound Identification Report

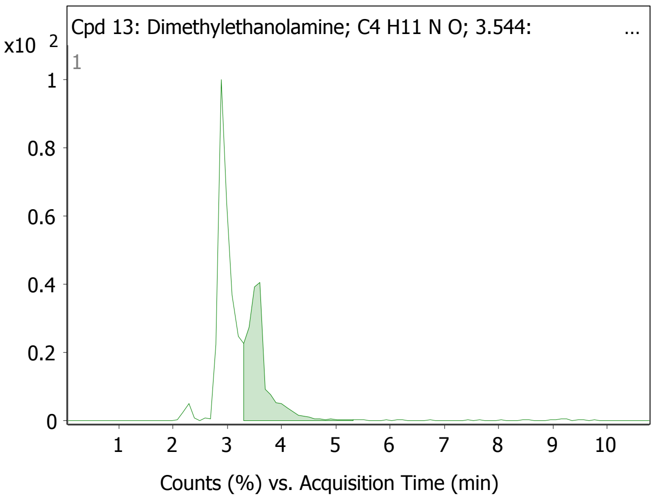

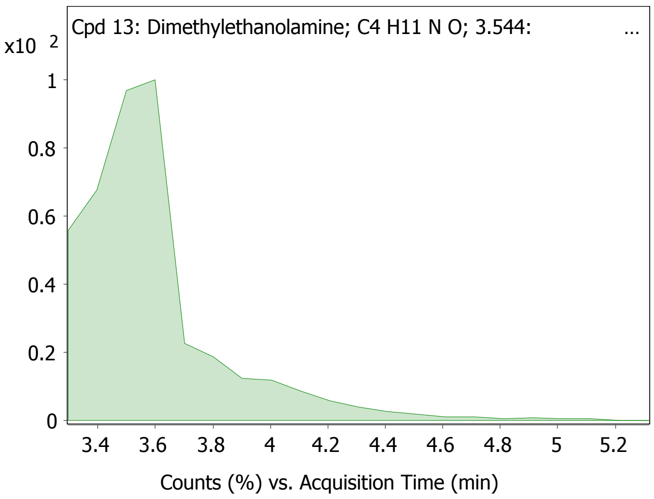

MFE MS Spectrum

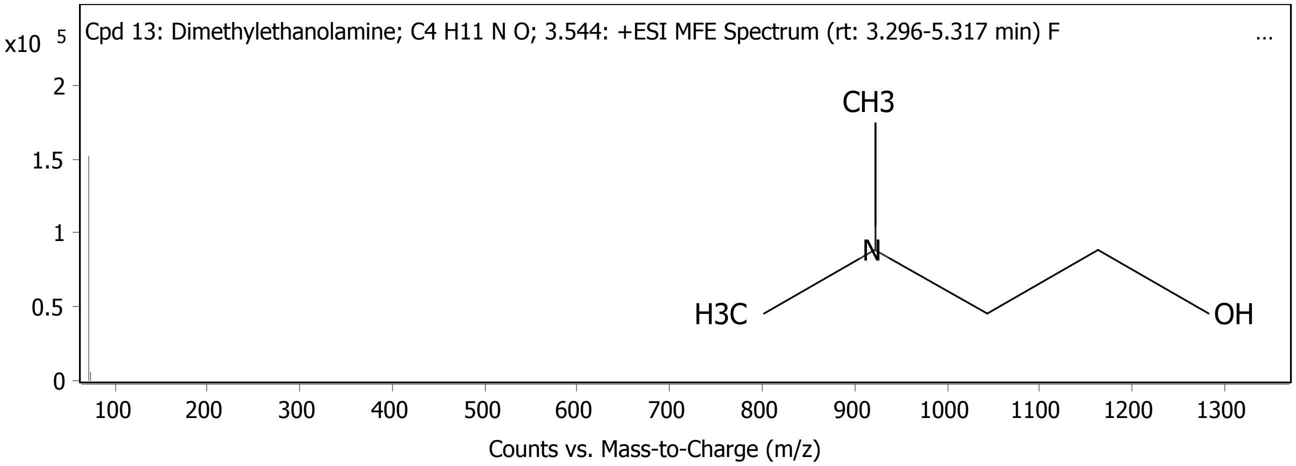

MFE MS Zoomed Spectrum

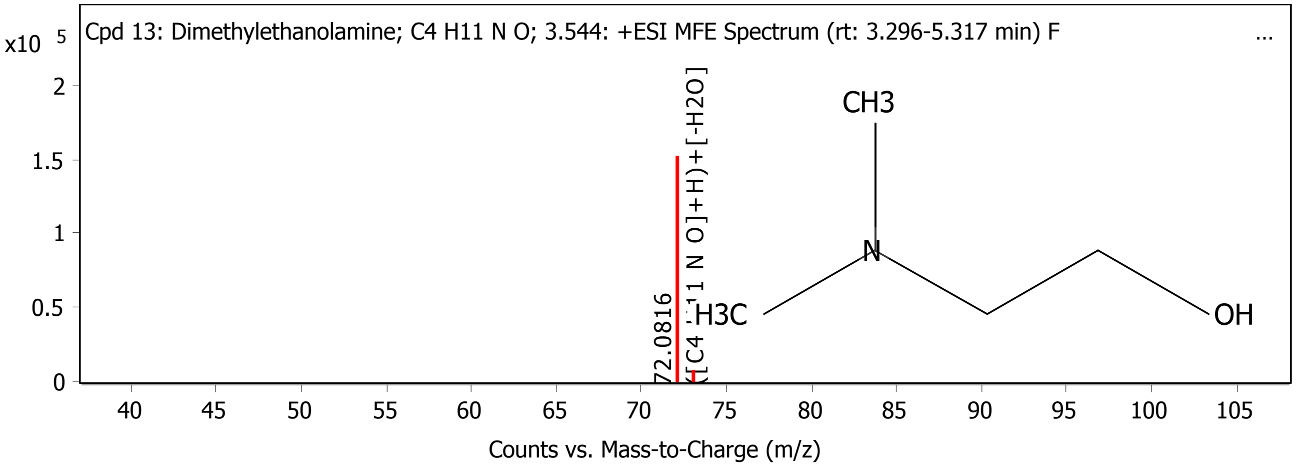

MS Spectrum

Qualitative Compound Identification Report

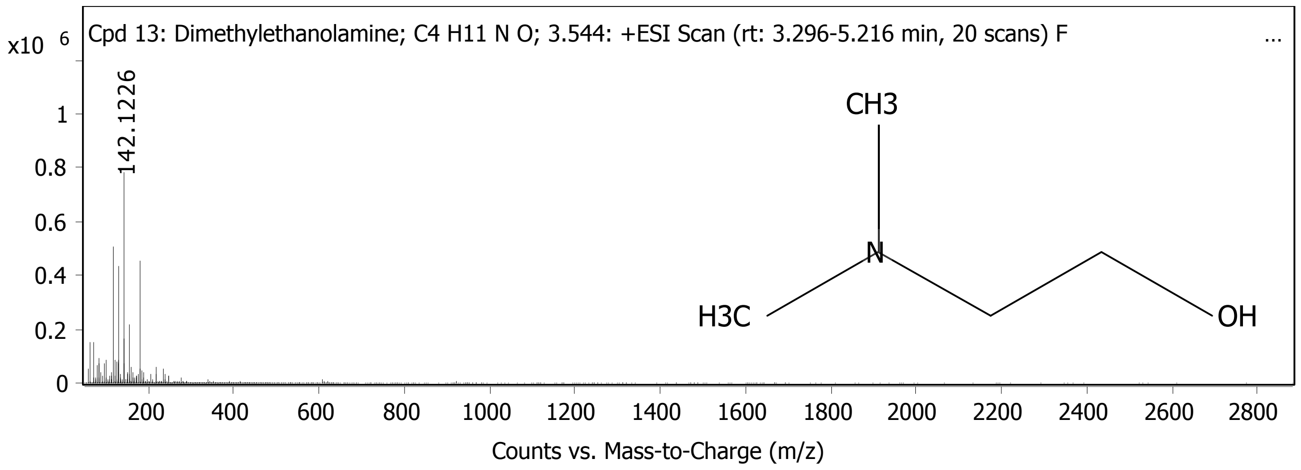

MS Zoomed Spectrum

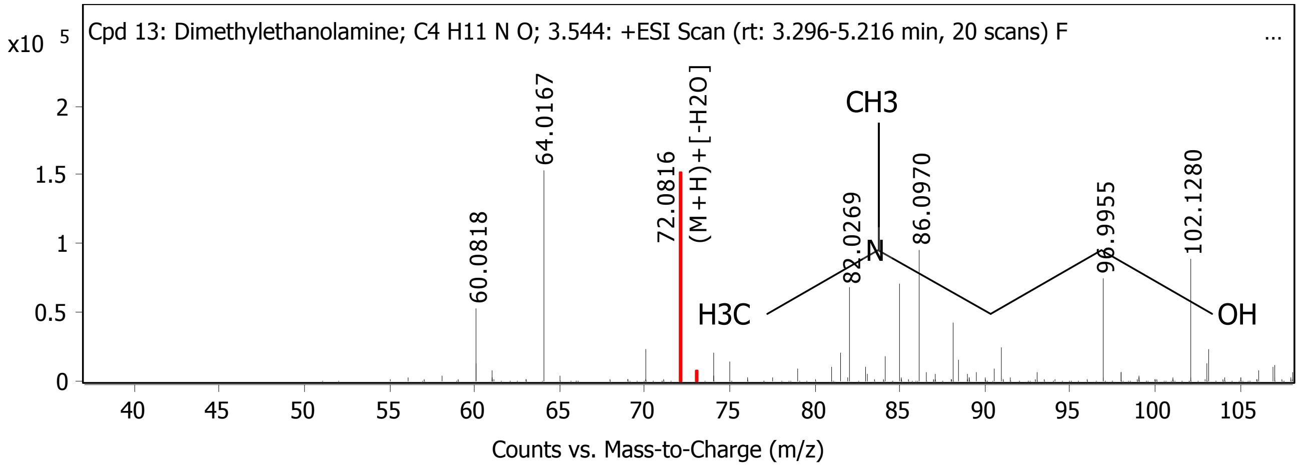

MSMS Spectrum

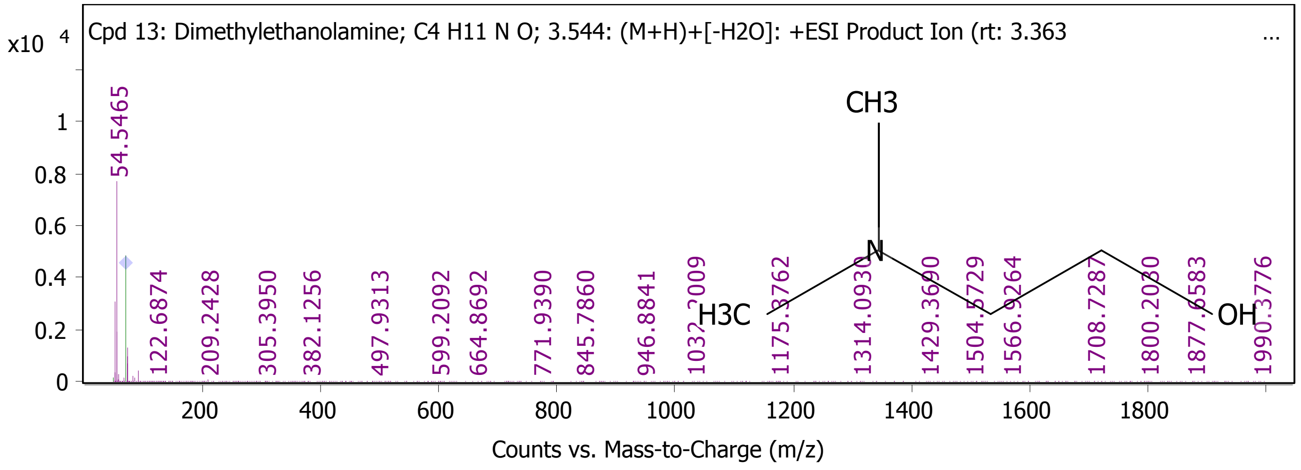

Identification Hit Table

Best Hit	Compound Name	RT	Formula	Notes	Match Score	Mass	Difference	Ion Species
✓	Dimethylethanolamine	3.544	C4 H11 N O		82.64	89.0849	-0.86	(M+H)+[-H2O]
	2-Amino-2-methyl-1-propanol	3.544	C4 H11 N O	â-Aminoisobutyl alcohol, AMP	82.64	89.0849	-0.86	(M+H)+[-H2O]
	2,2-Dimethyloxirane	3.544	C4 H8 O		82.23	72.0584	-0.86	(M+NH4)+[-H2O]
	2-Buten-1-ol	3.544	C4 H8 O		82.23	72.0584	-0.86	(M+NH4)+[-H2O]
	3-Buten-1-ol	3.544	C4 H8 O		82.23	72.0584	-0.86	(M+NH4)+[-H2O]
	1,2-Epoxybutane	3.544	C4 H8 O		82.23	72.0584	-0.86	(M+NH4)+[-H2O]
	Butanal	3.544	C4 H8 O		82.23	72.0584	-0.86	(M+NH4)+[-H2O]
	Tetrahydrofuran	3.544	C4 H8 O		82.23	72.0584	-0.86	(M+NH4)+[-H2O]
	Butanone	3.544	C4 H8 O		82.23	72.0584	-0.86	(M+NH4)+[-H2O]
	Pyrrolidine	3.544	C4 H9 N		82.2	71.0744	-0.86	(M+H)+

Qualitative Compound Identification Report

Identification Hit Table

Best Hit	Compound Name	RT	Formula	Notes	Match Score	Mass	Difference	Ion Species

Identification Hit Table

Best Hit	Compound Name	RT	Formula	Notes	Match Score	Mass	Difference	Ion Species

Compound Label	Name	*m/z*	RT	Algorithm	Mass
Cpd 14: Methyl orsellinate; C9 H10 O4; 5.238	**Methyl orsellinate**	182.0811	5.238	Find by Molecular Feature	182.0578

Compound Chromatograms

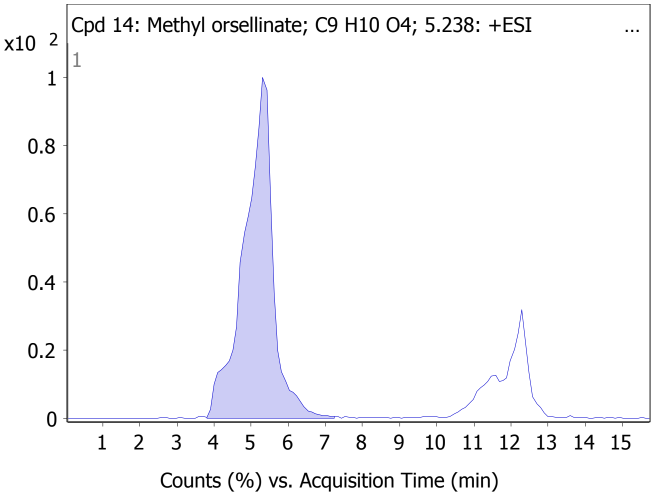

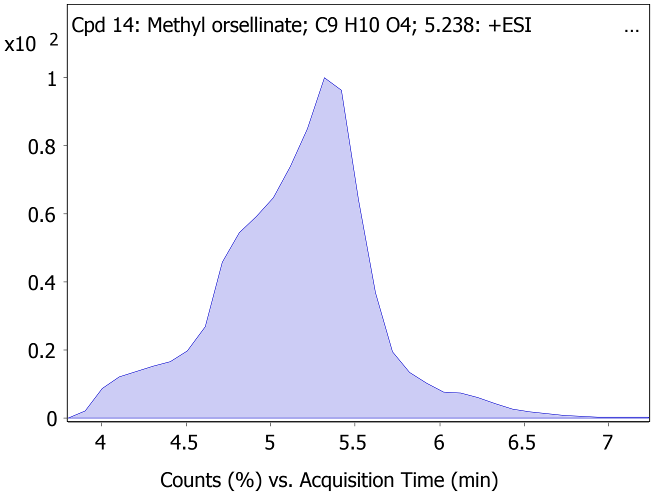

MFE MS Spectrum

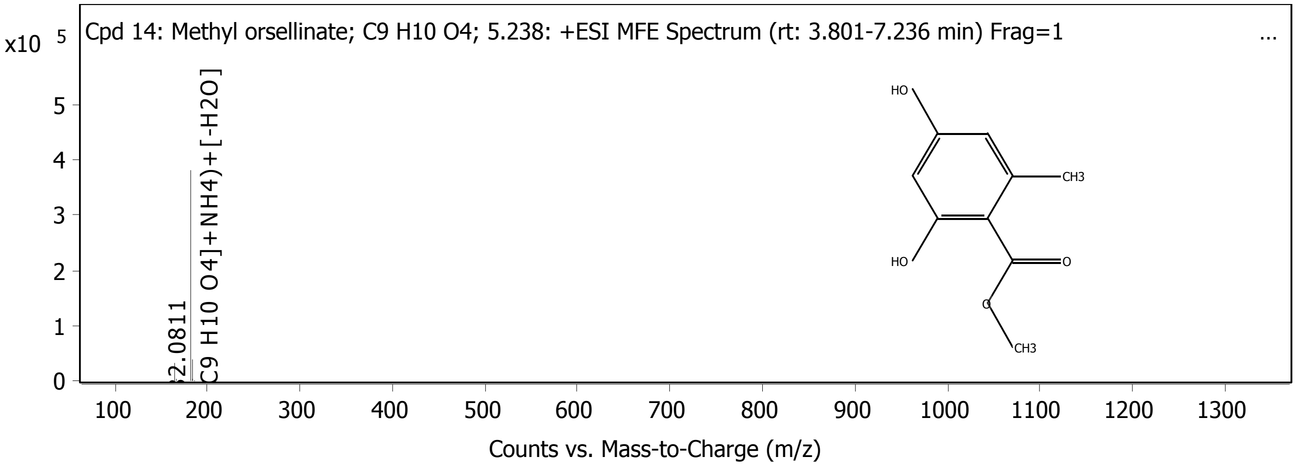

MFE MS Zoomed Spectrum

Qualitative Compound Identification Report

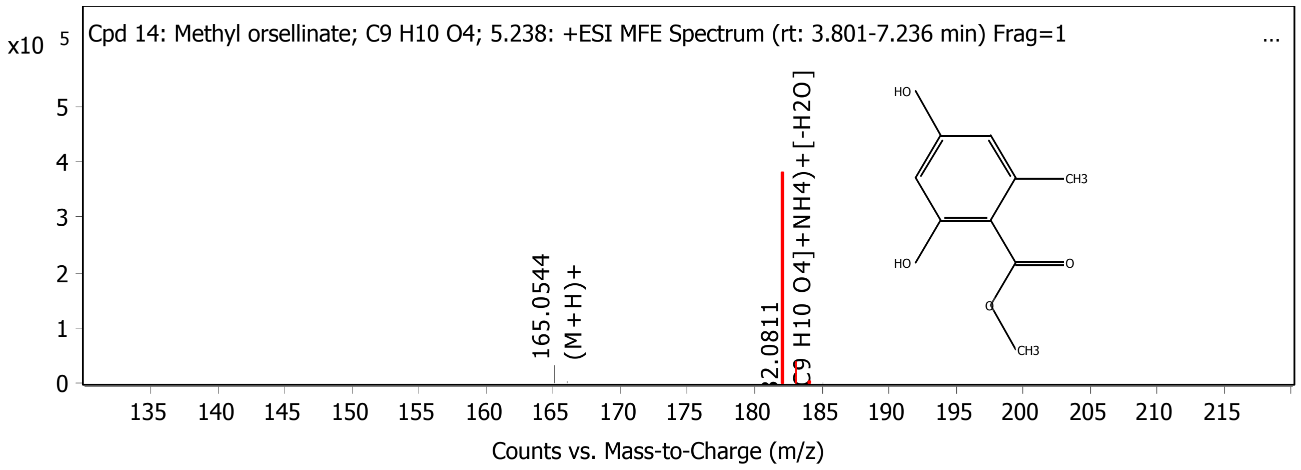

MS Spectrum

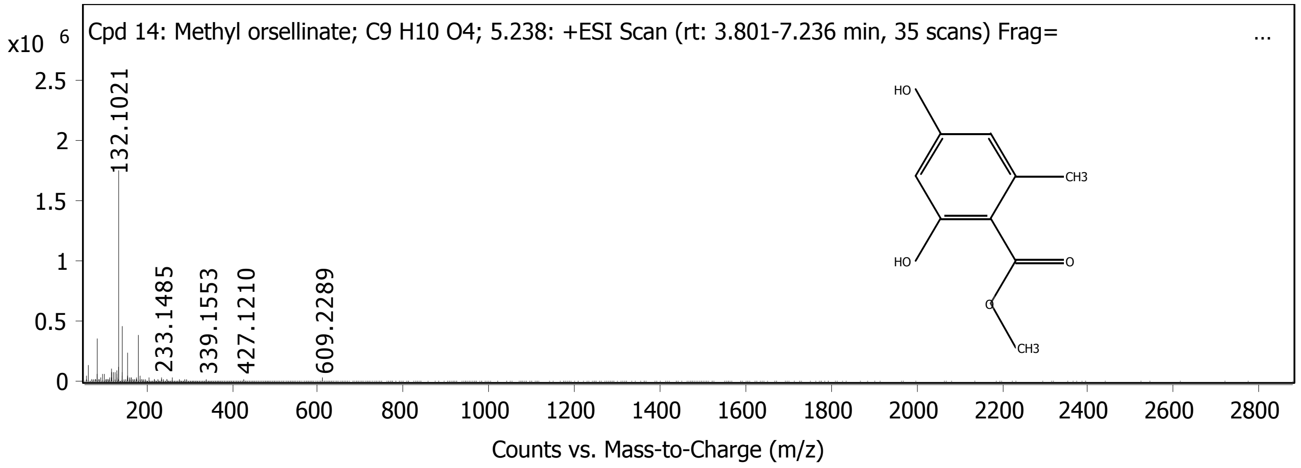

MS Zoomed Spectrum

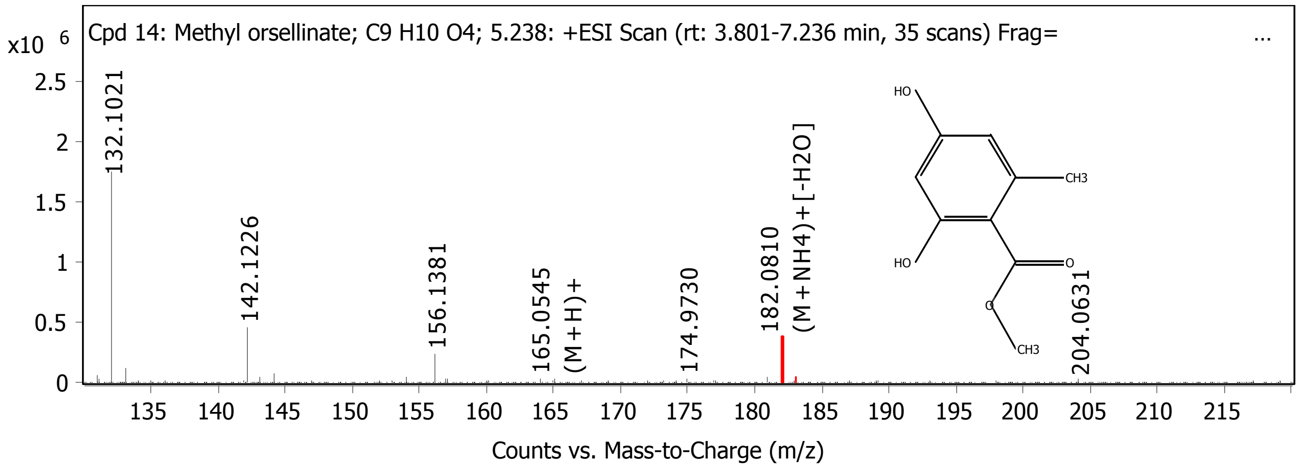

Identification Hit Table

Best Hit	Compound Name	RT	Formula	Notes	Match Score	Mass	Difference	Ion Species
✓	Methyl orsellinate	5.238	C9 H10 O4	Lichens and lichen acids	99.92	182.0578	0.12	(M+NH4)+[-H2O]
	3,4-Dihydroxyphenylpropanoate	5.238	C9 H10 O4	Nugo Website Http://Nugo.org/metabolomics/13496 Urine	99.92	182.0578	0.12	(M+NH4)+[-H2O]
	Methyl vanillate	5.238	C9 H10 O4		99.92	182.0578	0.12	(M+NH4)+[-H2O]
	3-Methoxy-4-hydroxyphenylglycolaldehyde	5.238	C9 H10 O4		99.92	182.0578	0.12	(M+NH4)+[-H2O]
	Homovanillic acid	5.238	C9 H10 O4	3-Methoxy-4-hydroxyphenylacetic acid, Homovanillate Treatment of Parkinsons, Segawa's and Restless legs syndrome Endogenous metabolite of L-DOPA Dollery, Colin Therapeutic	99.92	182.0578	0.12	(M+NH4)+[-H2O]

Qualitative Compound Identification Report

				Drugs, 2nd Ed. 1999 p. L39				
	Veratric acid	5.238	C9 H10 O4	Antispasmodic (Treatment of IBS and other abdominal complications) Metabolite of Mebeverine Dollery, Colin Therapeutic Drugs, 2nd Ed. 1999 p. M15	99.92	182.0578	0.12	(M+NH4)+[-H2O]
	4-Hydroxy-3,5-dimethoxybenzaldehyde	5.238	C9 H10 O4		99.92	182.0578	0.12	(M+NH4)+[-H2O]
	3-Hydroxy-4-methoxyphenylacetic acid	5.238	C9 H10 O4	Nugo Website Http://Nugo.org/metabolomics/13496 Urine	99.92	182.0578	0.12	(M+NH4)+[-H2O]
	Hydroxyphenyllactic acid	5.238	C9 H10 O4	Pubchem 9378	99.92	182.0578	0.12	(M+NH4)+[-H2O]
	3-Methylorsellinic acid	5.238	C9 H10 O4	Aspergillus terreus	99.92	182.0578	0.12	(M+NH4)+[-H2O]

Identification Hit Table

Best Hit	Compound Name	RT	Formula	Notes	Match Score	Mass	Difference	Ion Species
✓	9-hydroxy-7E-Nonene-3,5-diynoic acid	5.238	C9 H8 O3		99.92	164.0472	0.12	(M+NH4)+
	9-hydroxy-7Z-Nonene-3,5-diynoic acid	5.238	C9 H8 O3		99.92	164.0472	0.12	(M+NH4)+

Identification Hit Table

Best Hit	Compound Name	RT	Formula	Notes	Match Score	Mass	Difference	Ion Species

Compound Label	Name	*m/z*	RT	Algorithm	Mass
Cpd 15: L-Isoleucine; C6 H13 N O2; 5.456	**L-Isoleucine**	132.1022	5.456	Find by Molecular Feature	131.0949

Compound Chromatograms

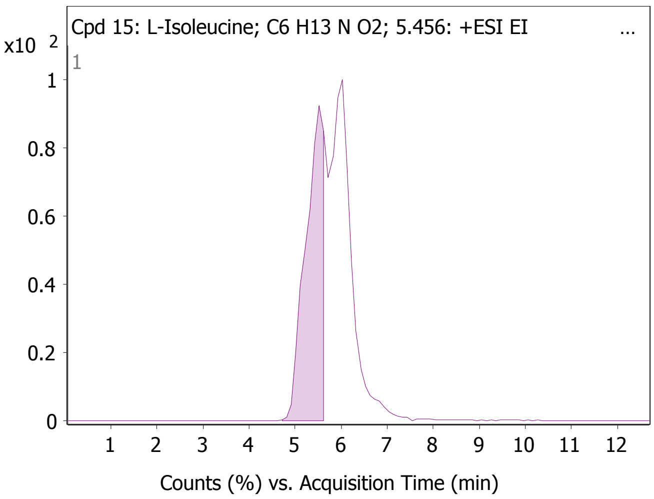

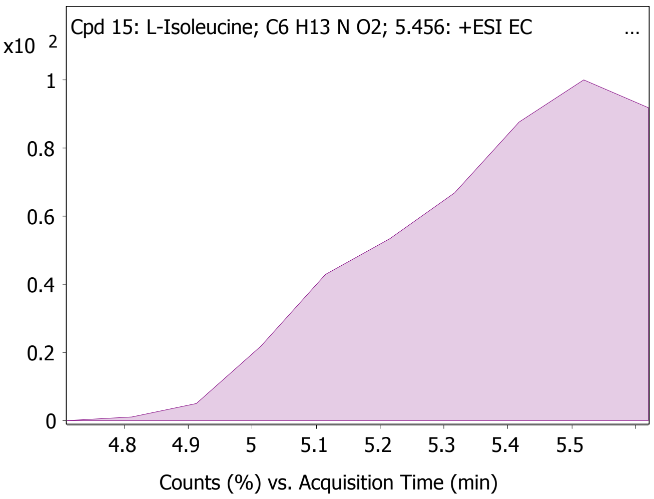

MFE MS Spectrum

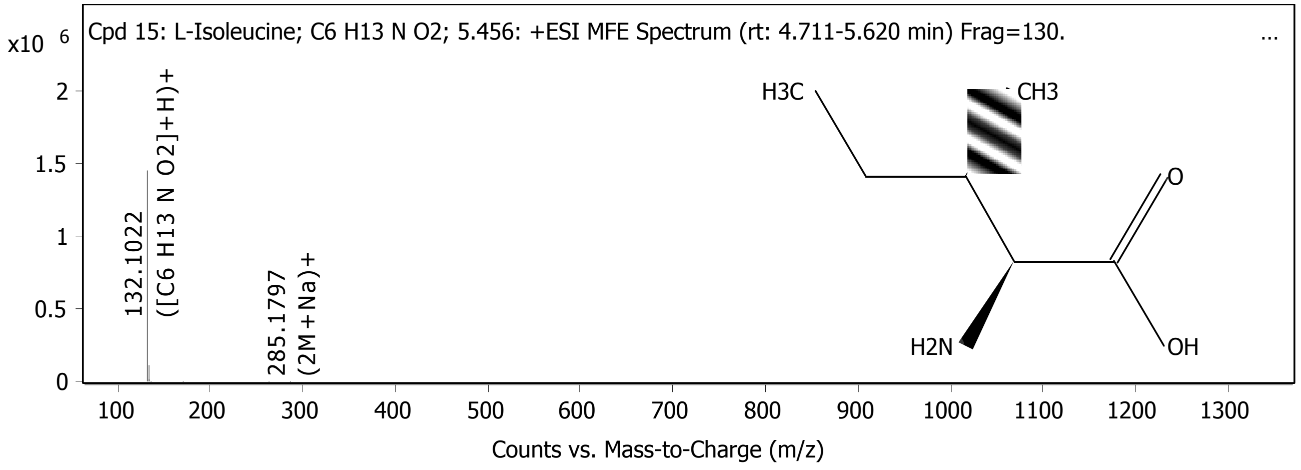

MFE MS Zoomed Spectrum

Qualitative Compound Identification Report

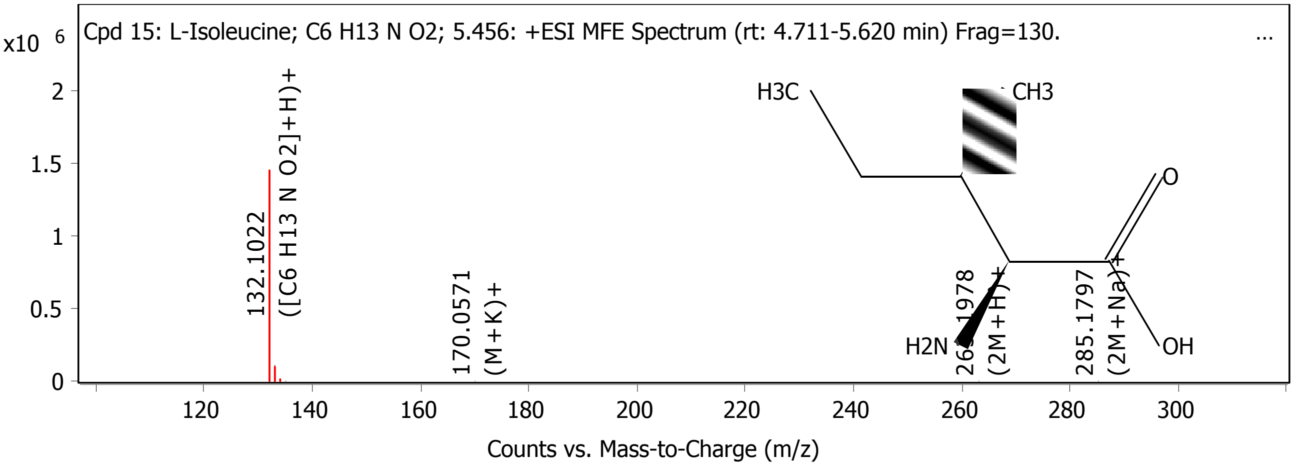

MS Spectrum

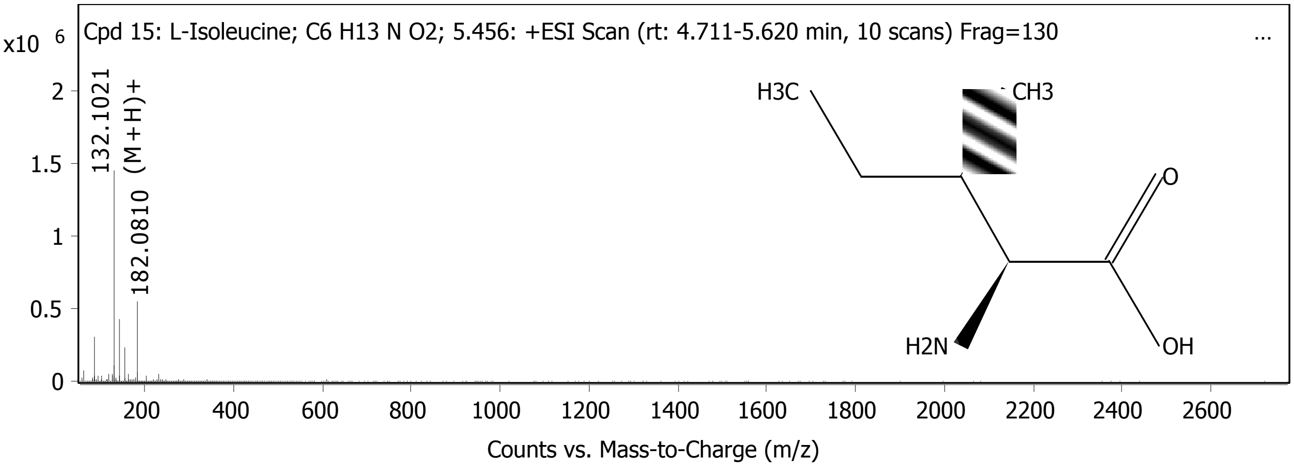

MS Zoomed Spectrum

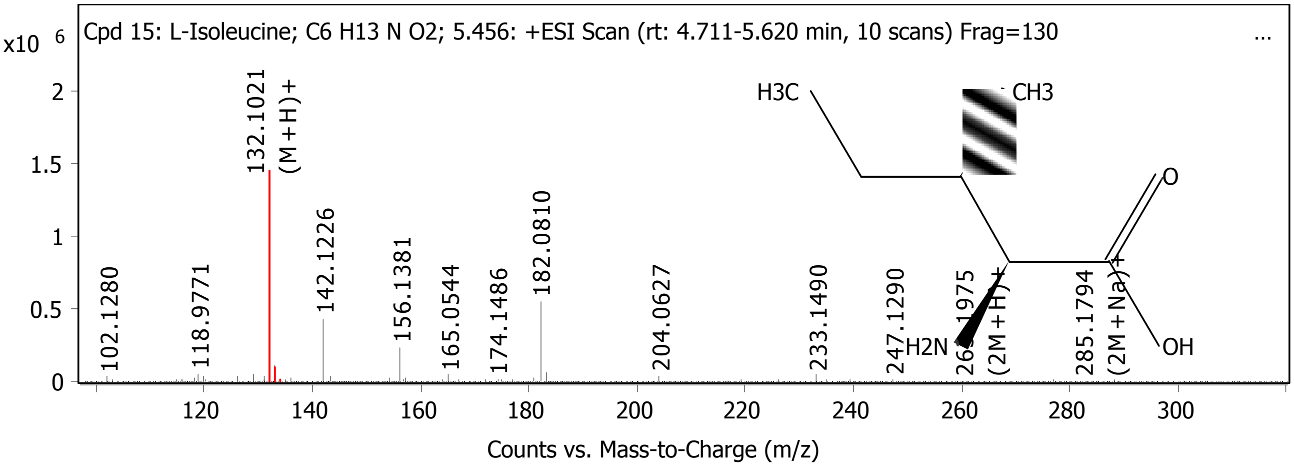

Library Spectrum

Qualitative Compound Identification Report

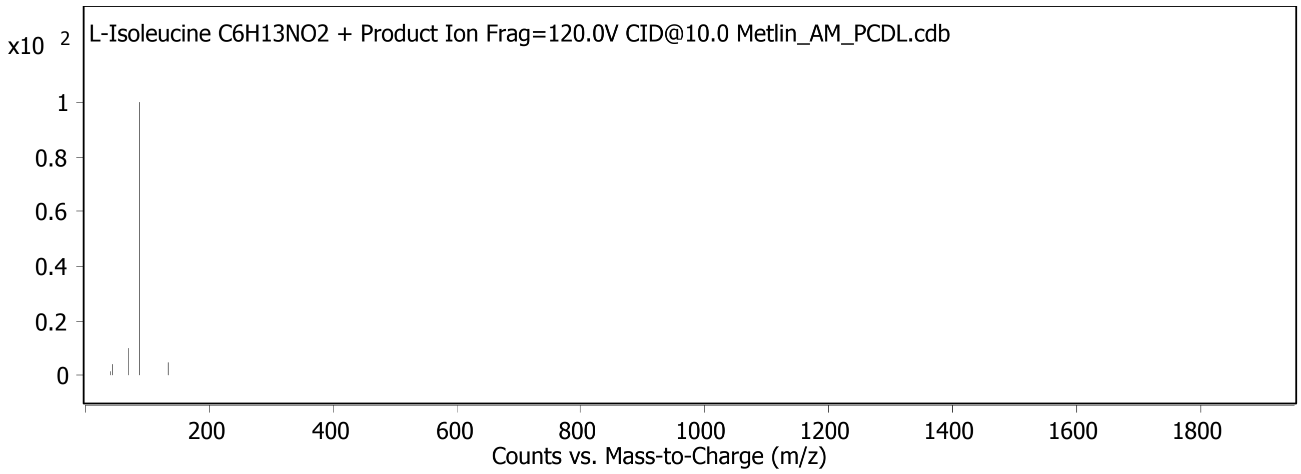

Difference Spectrum

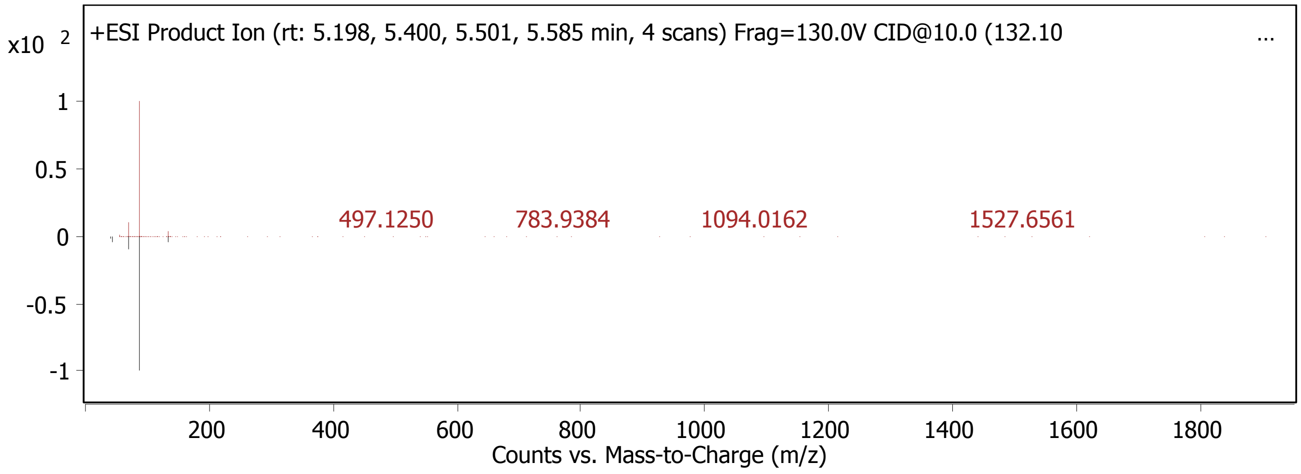

MSMS Spectrum

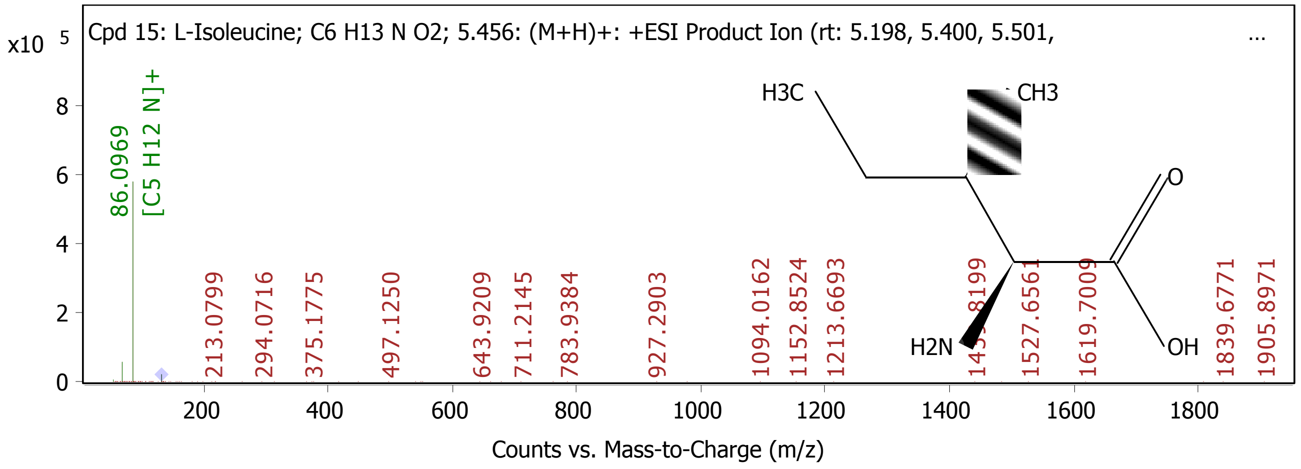

Identification Hit Table

Best Hit	Compound Name	RT	Formula	Notes	Match Score	Mass	Difference	Ion Species
✓	Trolamine	5.456	C6 H15 N O3	Pubchem 7618	99.39	149.1055	-0.3	(M+H)+[-H2O]
	6-Deoxyfagomine	5.456	C6 H13 N O2		99.35	131.0949	-0.3	(M+H)+
	3-Aminocaproic acid	5.456	C6 H13 N O2		99.35	131.0949	-0.3	(M+H)+
	1-Nitrohexane	5.456	C6 H13 N O2		99.35	131.0949	-0.3	(M+H)+
	N,N-Diethylglycine	5.456	C6 H13 N O2		99.35	131.0949	-0.3	(M+H)+
	Isoleucine	5.456	C6 H13 N O2		99.35	131.0949	-0.3	(M+H)+
	DL-β-Leucine	5.456	C6 H13 N O2		99.35	131.0949	-0.3	(M+H)+
	L-Alloisoleucine	5.456	C6 H13 N O2		99.35	131.0949	-0.3	(M+H)+
	2-Amino-4-methylpentanoic acid	5.456	C6 H13 N O2		99.35	131.0949	-0.3	(M+H)+
	L-Norleucine	5.456	C6 H13 N O2		99.35	131.0949	-0.3	(M+H)+

Qualitative Compound Identification Report

Identification Hit Table

Best Hit	Compound Name	RT	Formula	Notes	Match Score	Mass	Difference	Ion Species
✓	Aminocaproic acid	5.456	C6 H13 N O2	hexanoic acid, 6-aminohexanoic acid. The United States Pharmacopeia 29, The National Formulary 24, 2006	99.35	131.0949	-0.3	(M+H)+
	L-Norleucine	5.456	C6 H13 N O2		99.35	131.0949	-0.3	(M+H)+
	L-Isoleucine	5.456	C6 H13 N O2	Postive MS/MS Endogenous Metabolite Geigy vol. 3 p. 92 Deleted CAS: 7004-09-3; 410078-51-2; 501028-75-7; 807611-99-0; 959215-79-3; 1050482-26-2; 1131911-83-5; 1186126-44-2; 1431840-86-6; 2012595-46-7	99.35	131.0949	-0.3	(M+H)+
	L-Leucine	5.456	C6 H13 N O2	Postive MS/MS Endogenous Metabolite Geigy vol. 3 p. 92	99.35	131.0949	-0.3	(M+H)+
	2-Amino-4-methylpentanoic acid	5.456	C6 H13 N O2		99.35	131.0949	-0.3	(M+H)+

Identification Hit Table

Best Hit	Compound Name	RT	Formula	Notes	Match Score	Mass	Difference	Ion Species

Compound Label	Name	*m/z*	RT	Algorithm	Mass
Cpd 16: 2-Amino-3-methyl-1-butanol; C5 H13 N O; 5.458	**2-Amino-3-methyl-1-butanol**	86.0971	5.458	Find by Molecular Feature	103.1003

Compound Chromatograms

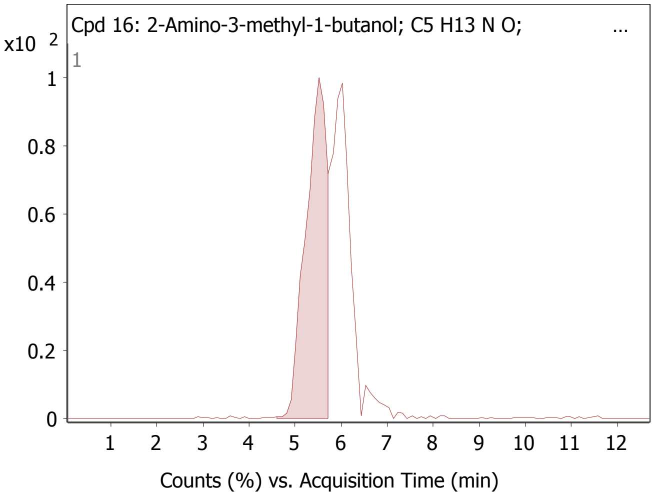

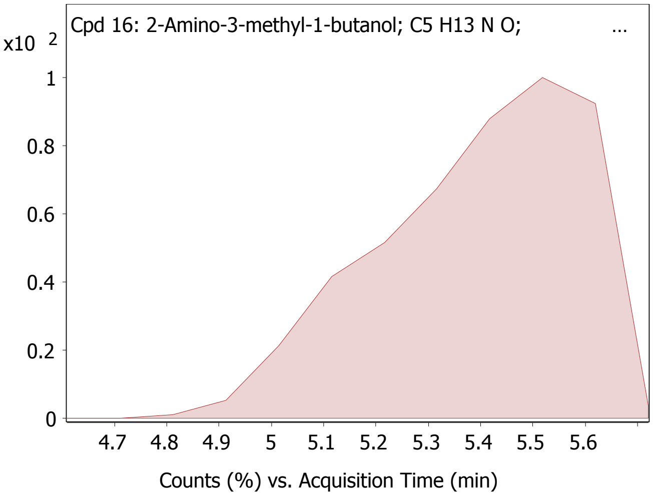

MFE MS Spectrum

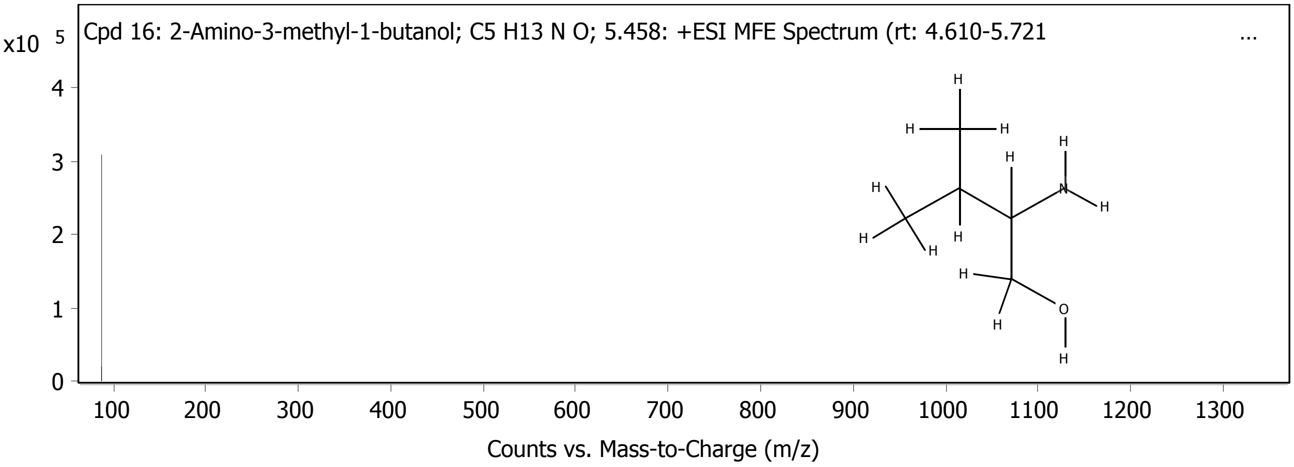

MFE MS Zoomed Spectrum

Qualitative Compound Identification Report

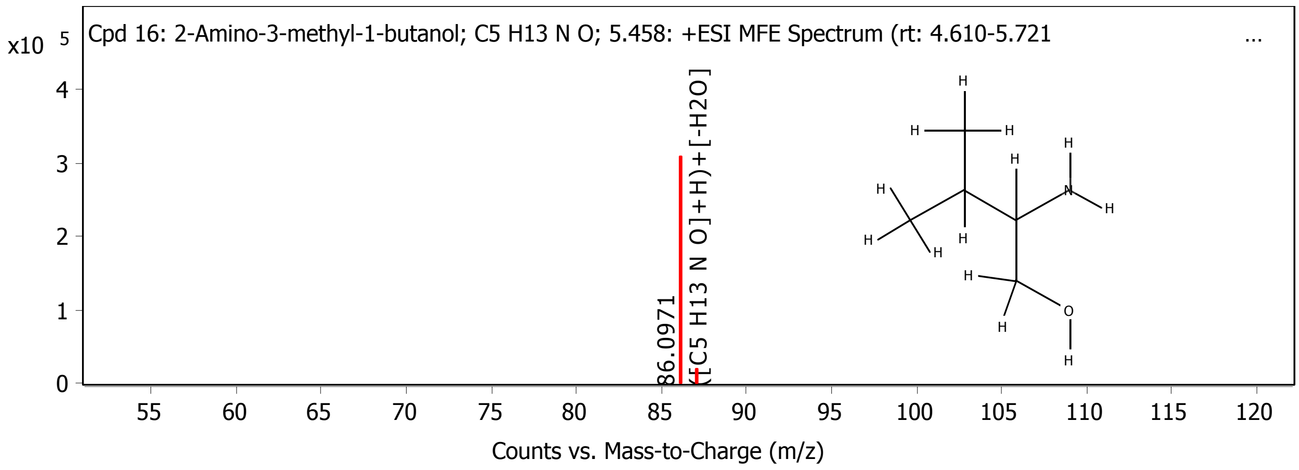

MS Spectrum

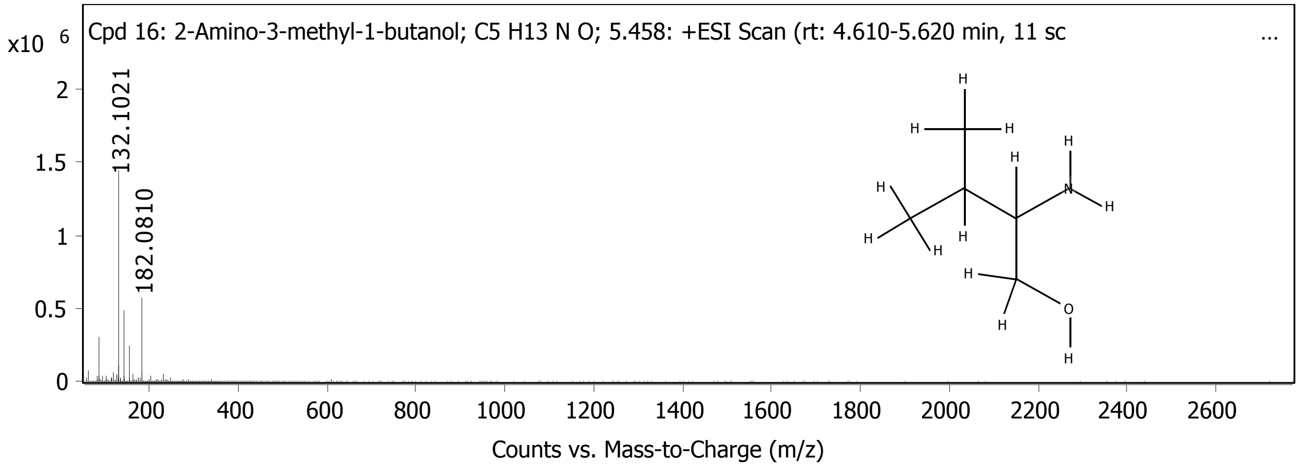

MS Zoomed Spectrum

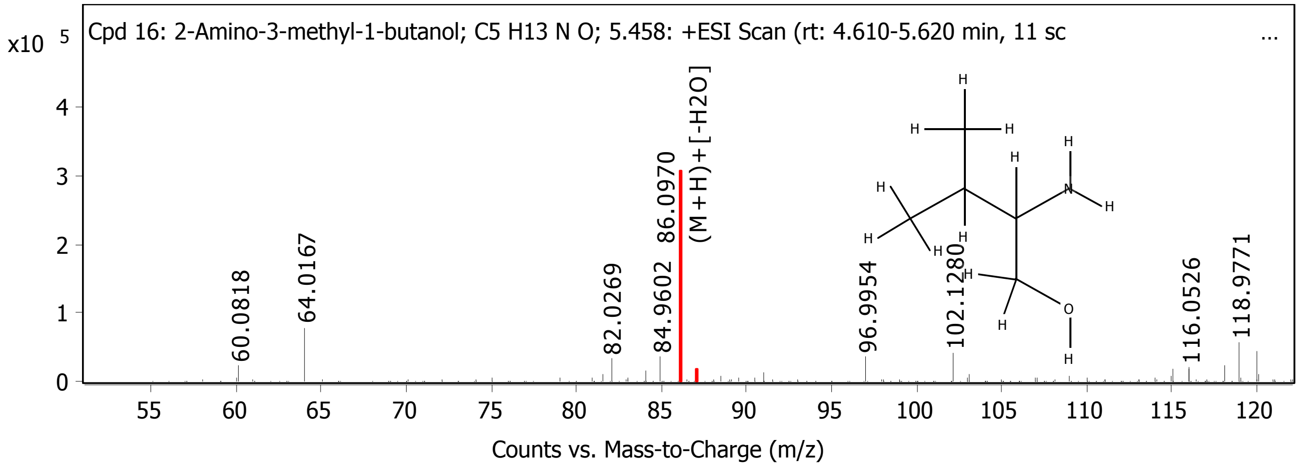

MSMS Spectrum

Qualitative Compound Identification Report

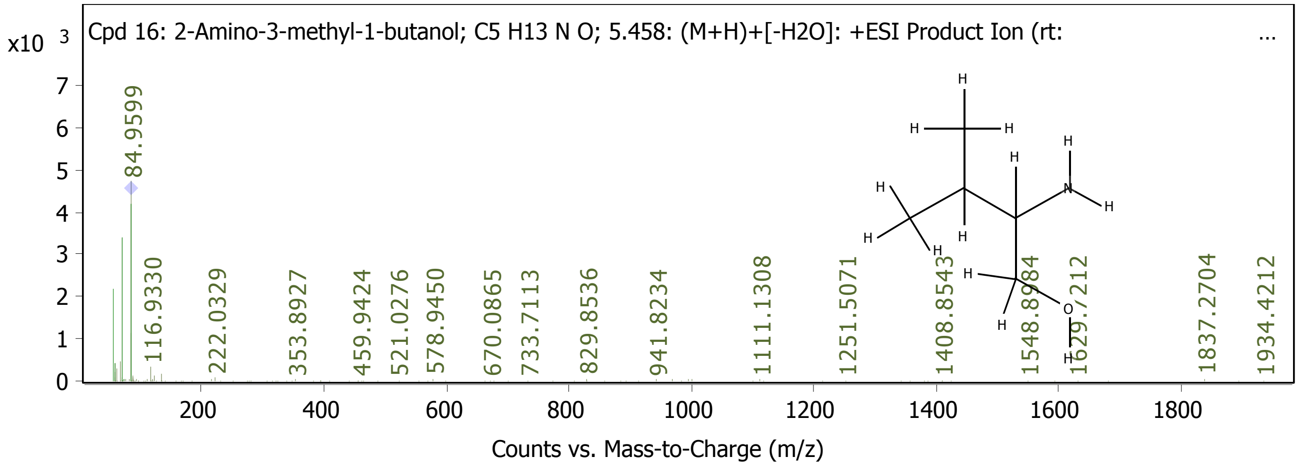

Identification Hit Table

Best Hit	Compound Name	RT	Formula	Notes	Match Score	Mass	Difference	Ion Species
✓	2-Amino-3-methyl-1-butanol	5.458	C5 H13 N O		85.3	103.1003	-0.63	(M+H)+[-H2O]
	3-Methylbutan-2-one	5.458	C5 H10 O		85.09	86.0738	-0.63	(M+NH4)+[-H2O]
	xi-3-Methyl-3-buten-2-ol	5.458	C5 H10 O		85.09	86.0738	-0.63	(M+NH4)+[-H2O]
	pentan-3-one	5.458	C5 H10 O		85.09	86.0738	-0.63	(M+NH4)+[-H2O]
	pentan-2-one	5.458	C5 H10 O		85.09	86.0738	-0.63	(M+NH4)+[-H2O]
	2-Methyl-3-buten-1-ol	5.458	C5 H10 O		85.09	86.0738	-0.63	(M+NH4)+[-H2O]
	2-Methylenebutan-1-ol	5.458	C5 H10 O		85.09	86.0738	-0.63	(M+NH4)+[-H2O]
	3-Methyl-2-buten-1-ol	5.458	C5 H10 O		85.09	86.0738	-0.63	(M+NH4)+[-H2O]
	3-Methyl-3-buten-1-ol	5.458	C5 H10 O		85.09	86.0738	-0.63	(M+NH4)+[-H2O]
	2Z-Penten-1-ol	5.458	C5 H10 O		85.09	86.0738	-0.63	(M+NH4)+[-H2O]

Identification Hit Table

Best Hit	Compound Name	RT	Formula	Notes	Match Score	Mass	Difference	Ion Species
✓	2-Methyl-3-buten-1-ol	5.458	C5 H10 O		85.09	86.0738	-0.63	(M+NH4)+[-H2O]
	2-Methylenebutan-1-ol	5.458	C5 H10 O		85.09	86.0738	-0.63	(M+NH4)+[-H2O]
	3-Methyl-2-buten-1-ol	5.458	C5 H10 O		85.09	86.0738	-0.63	(M+NH4)+[-H2O]
	3-Methyl-3-buten-1-ol	5.458	C5 H10 O		85.09	86.0738	-0.63	(M+NH4)+[-H2O]
	2Z-Penten-1-ol	5.458	C5 H10 O		85.09	86.0738	-0.63	(M+NH4)+[-H2O]
	Pentanal	5.458	C5 H10 O		85.09	86.0738	-0.63	(M+NH4)+[-H2O]
	pentan-3-one	5.458	C5 H10 O		85.09	86.0738	-0.63	(M+NH4)+[-H2O]
	pentan-2-one	5.458	C5 H10 O		85.09	86.0738	-0.63	(M+NH4)+[-H2O]
	3-Methylbutan-2-one	5.458	C5 H10 O		85.09	86.0738	-0.63	(M+NH4)+[-H2O]

Identification Hit Table

Best Hit	Compound Name	RT	Formula	Notes	Match Score	Mass	Difference	Ion Species

Compound Label	Name	*m/z*	RT	Algorithm	Mass
Cpd 17: L-Isoleucine; C6 H13 N O2; 5.990	**L-Isoleucine**	132.1021	5.99	Find by Molecular Feature	131.0948

Compound Chromatograms

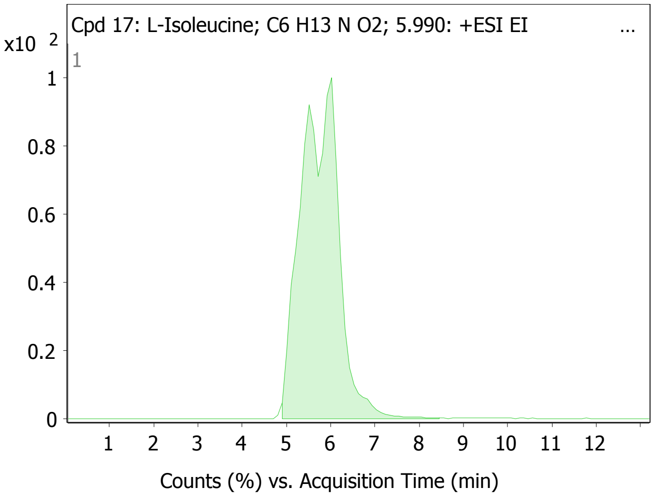

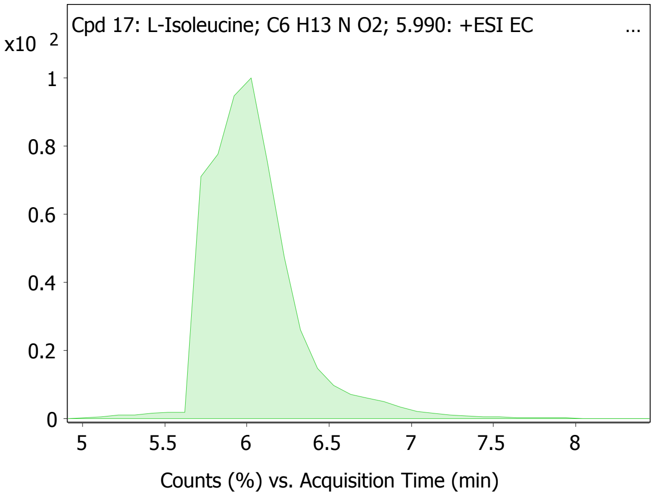

MFE MS Spectrum

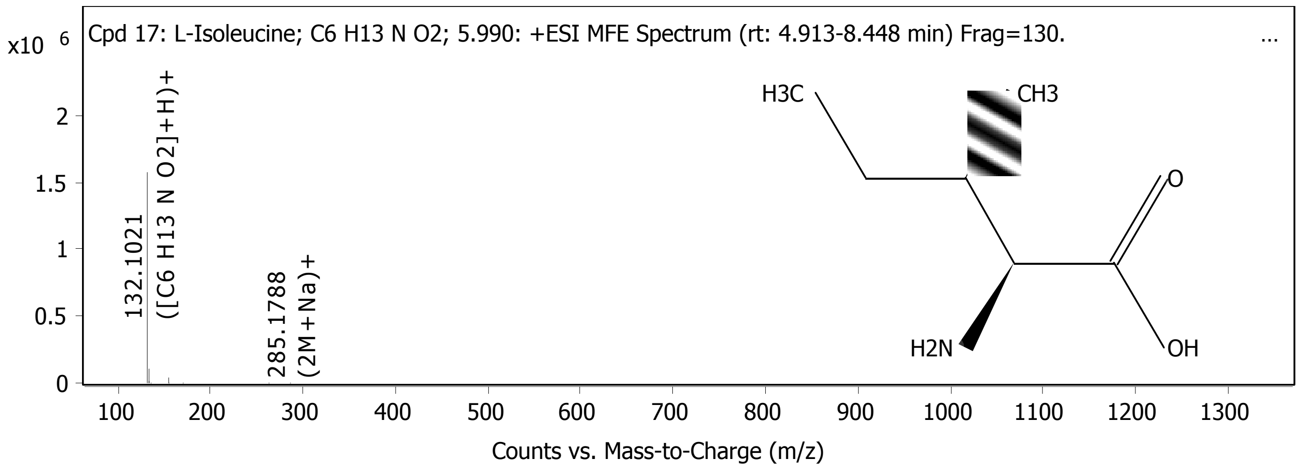

MFE MS Zoomed Spectrum

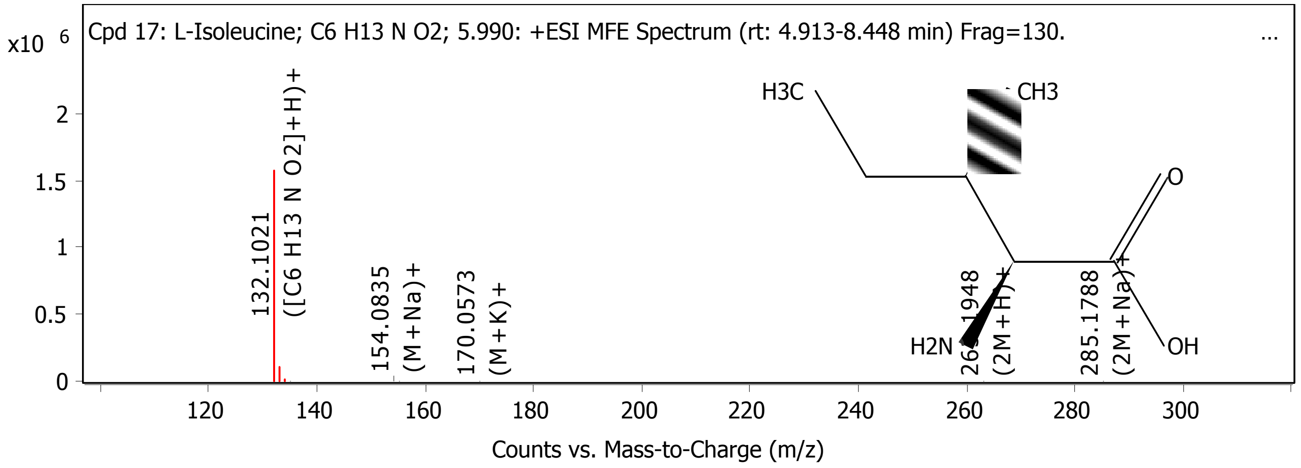

MS Spectrum

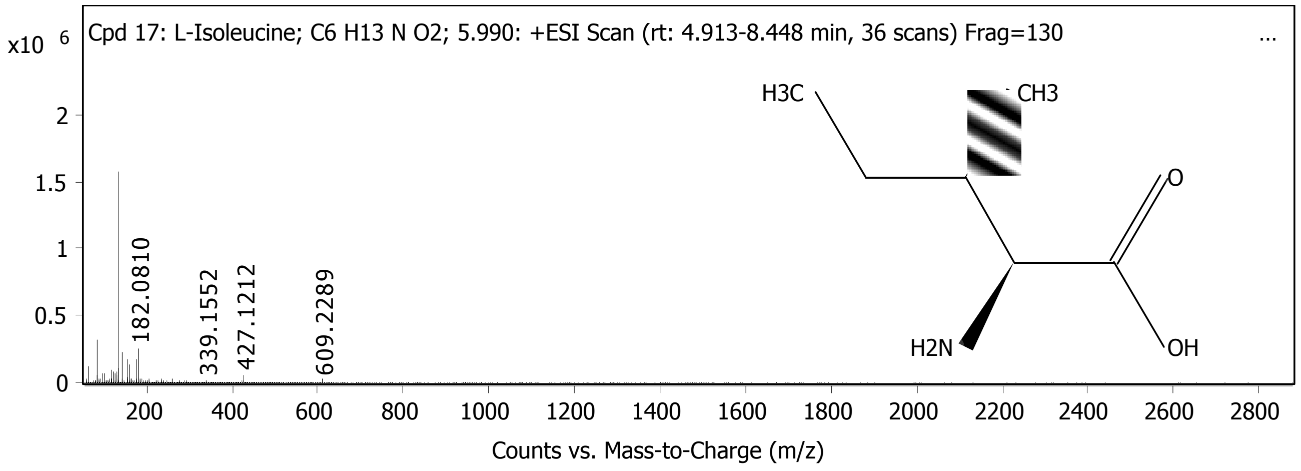

MS Zoomed Spectrum

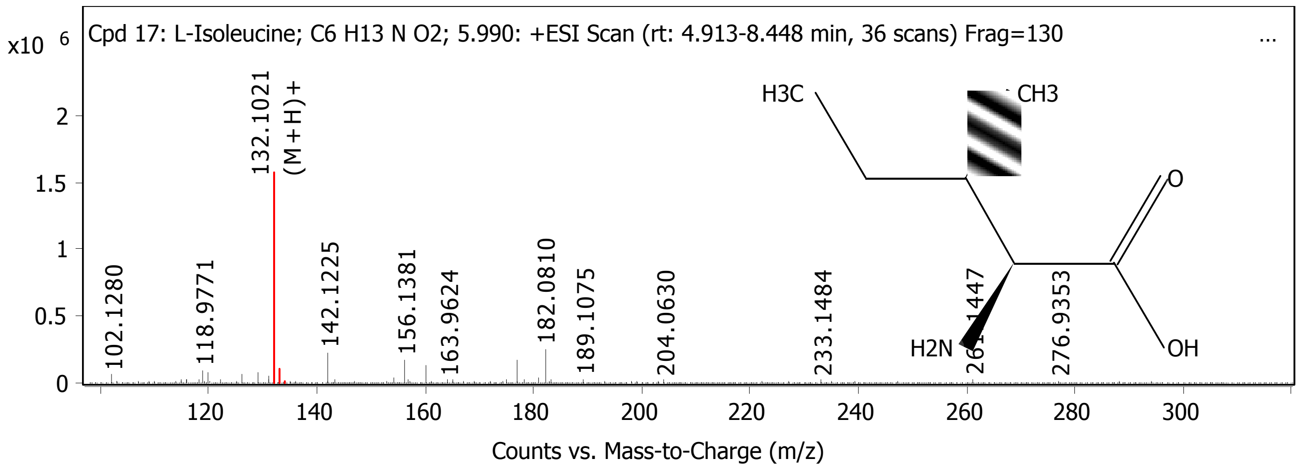

Library Spectrum

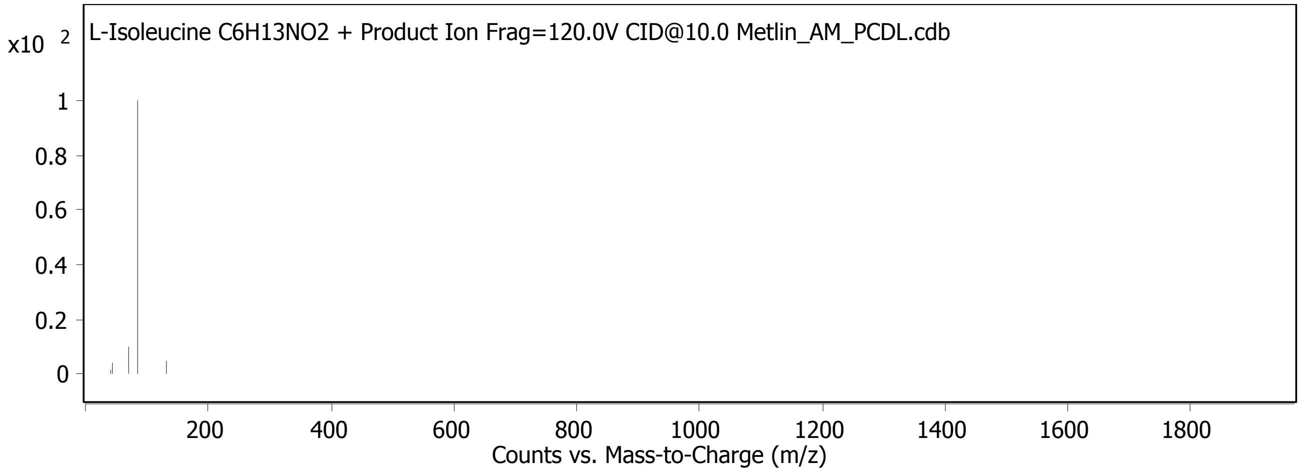

Difference Spectrum

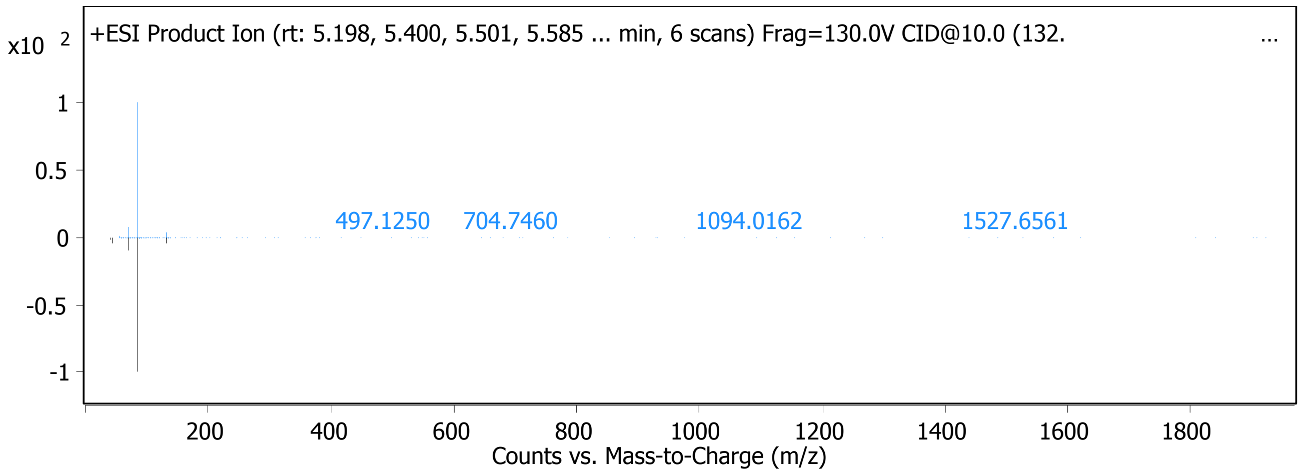

MSMS Spectrum

Qualitative Compound Identification Report

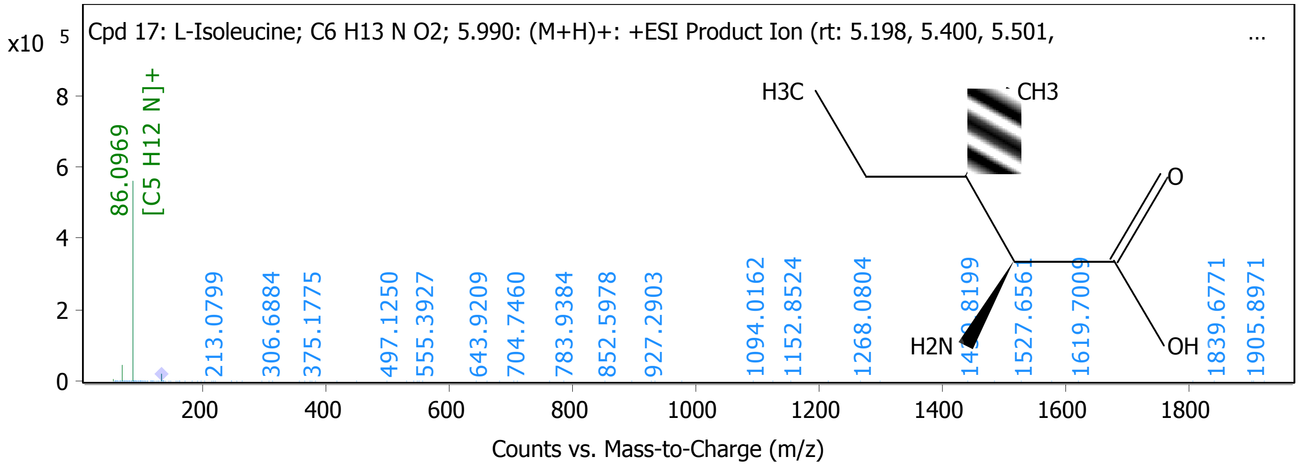

Identification Hit Table

Best Hit	Compound Name	RT	Formula	Notes	Match Score	Mass	Difference	Ion Species
✓	Trolamine	5.99	C6 H15 N O3	Pubchem 7618	99.79	149.1054	-0.17	(M+H)+[-H2O]
	6-Deoxyfagomine	5.99	C6 H13 N O2		99.77	131.0948	-0.17	(M+H)+
	3-Aminocaproic acid	5.99	C6 H13 N O2		99.77	131.0948	-0.17	(M+H)+
	1-Nitrohexane	5.99	C6 H13 N O2		99.77	131.0948	-0.17	(M+H)+
	N,N-Diethylglycine	5.99	C6 H13 N O2		99.77	131.0948	-0.17	(M+H)+
	Isoleucine	5.99	C6 H13 N O2		99.77	131.0948	-0.17	(M+H)+
	DL-β-Leucine	5.99	C6 H13 N O2		99.77	131.0948	-0.17	(M+H)+
	L-Alloisoleucine	5.99	C6 H13 N O2		99.77	131.0948	-0.17	(M+H)+
	2-Amino-4-methylpentanoic acid	5.99	C6 H13 N O2		99.77	131.0948	-0.17	(M+H)+
	L-Norleucine	5.99	C6 H13 N O2		99.77	131.0948	-0.17	(M+H)+

Identification Hit Table

Best Hit	Compound Name	RT	Formula	Notes	Match Score	Mass	Difference	Ion Species
✓	Aminocaproic acid	5.99	C6 H13 N O2	hexanoic acid, 6-aminohexanoic acid. The United States Pharmacopeia 29, The National Formulary 24, 2006	99.77	131.0948	-0.17	(M+H)+
	L-Norleucine	5.99	C6 H13 N O2		99.77	131.0948	-0.17	(M+H)+
	L-Isoleucine	5.99	C6 H13 N O2	Postive MS/MS Endogenous Metabolite Geigy vol. 3 p. 92 Deleted CAS: 7004-09-3; 410078-51-2; 501028-75-7; 807611-99-0; 959215-79-3; 1050482-26-2; 1131911-83-5; 1186126-44-2; 1431840-86-6; 2012595-46-7	99.77	131.0948	-0.17	(M+H)+
	L-Leucine	5.99	C6 H13 N O2	Postive MS/MS Endogenous Metabolite Geigy vol. 3 p. 92	99.77	131.0948	-0.17	(M+H)+
	2-Amino-4-methylpentanoic acid	5.99	C6 H13 N O2		99.77	131.0948	-0.17	(M+H)+

Identification Hit Table

Best Hit	Compound Name	RT	Formula	Notes	Match Score	Mass	Difference	Ion Species

Compound Label	Name	*m/z*	RT	Algorithm	Mass
Cpd 18: 2-Amino-3-methyl-1-butanol; C5 H13 N O; 5.996	**2-Amino-3-methyl-1-butanol**	86.097	5.996	Find by Molecular Feature	103.1003

Compound Chromatograms

Qualitative Compound Identification Report

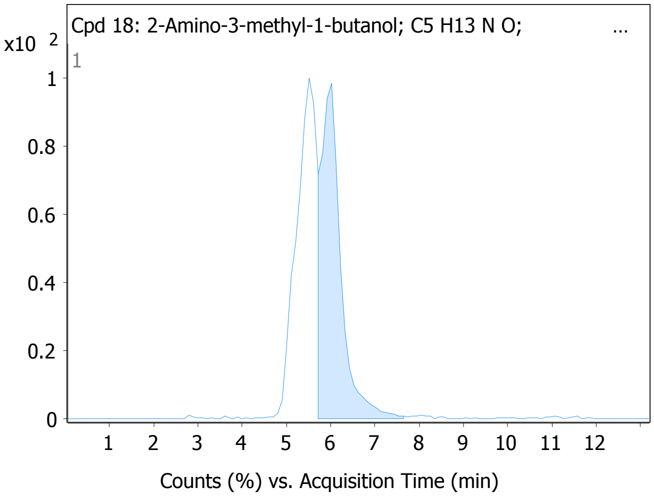

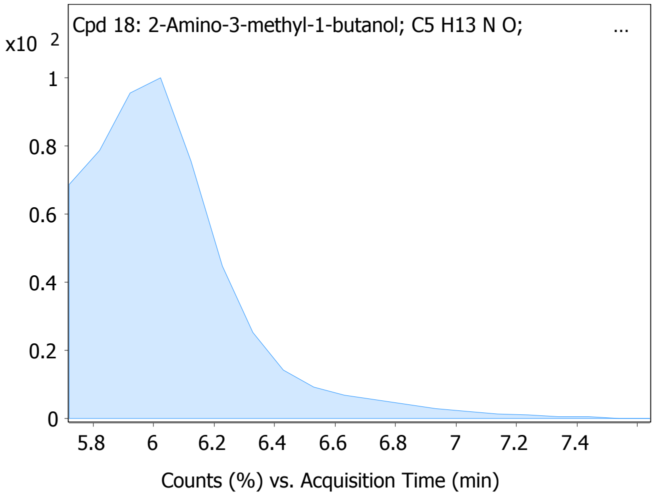

MFE MS Spectrum

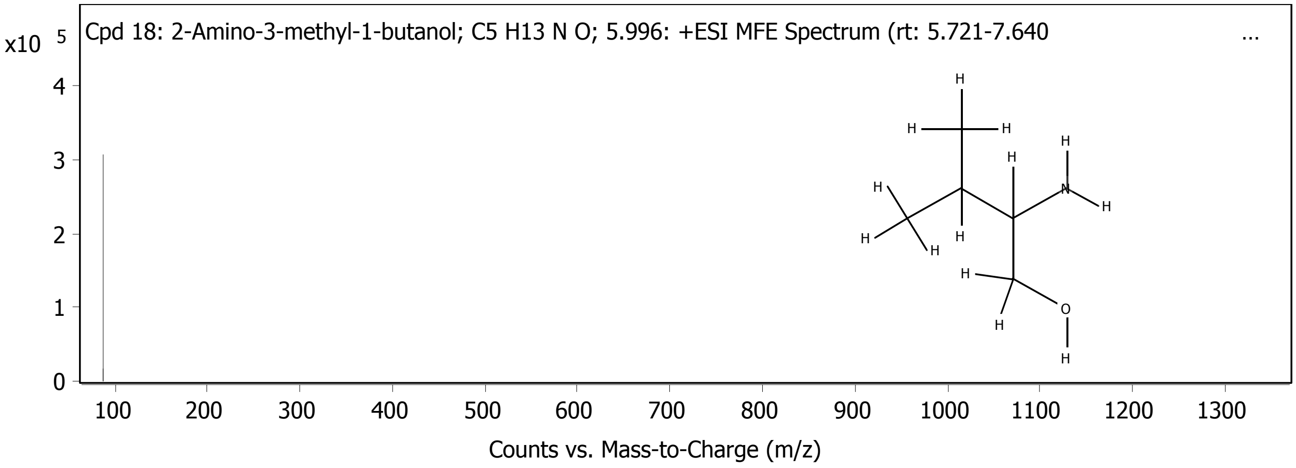

MFE MS Zoomed Spectrum

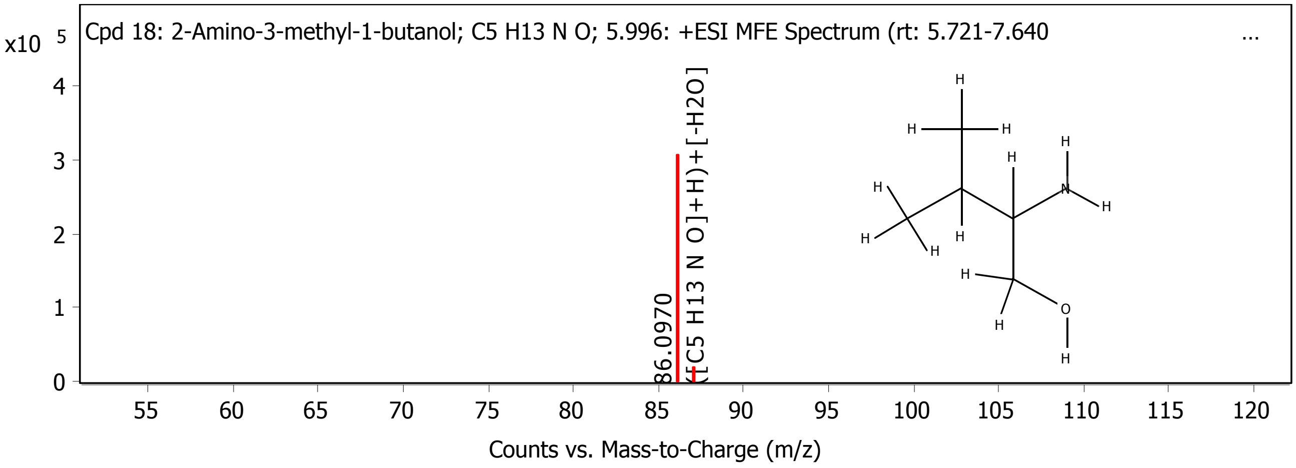

MS Spectrum

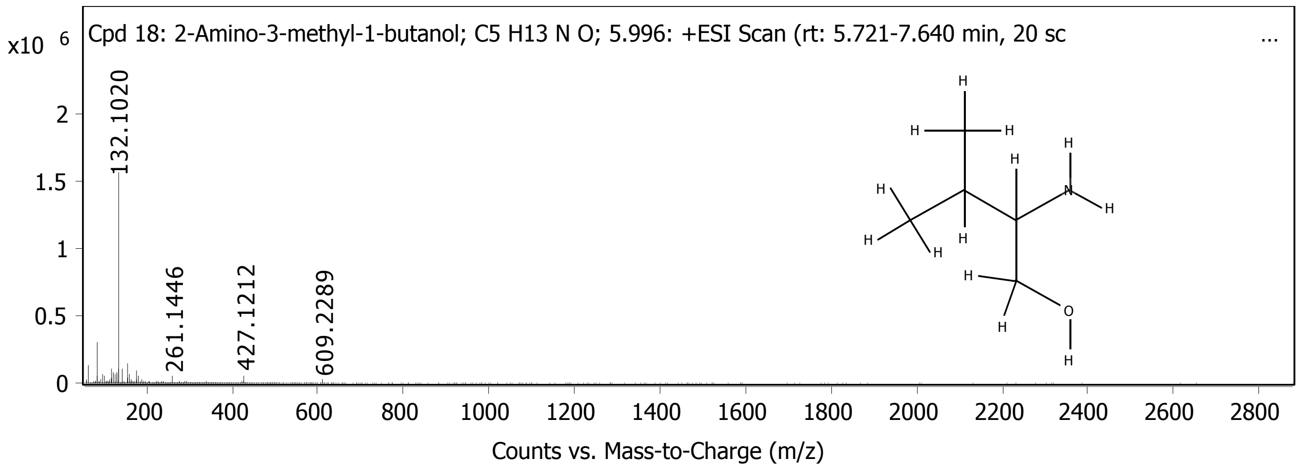

MS Zoomed Spectrum

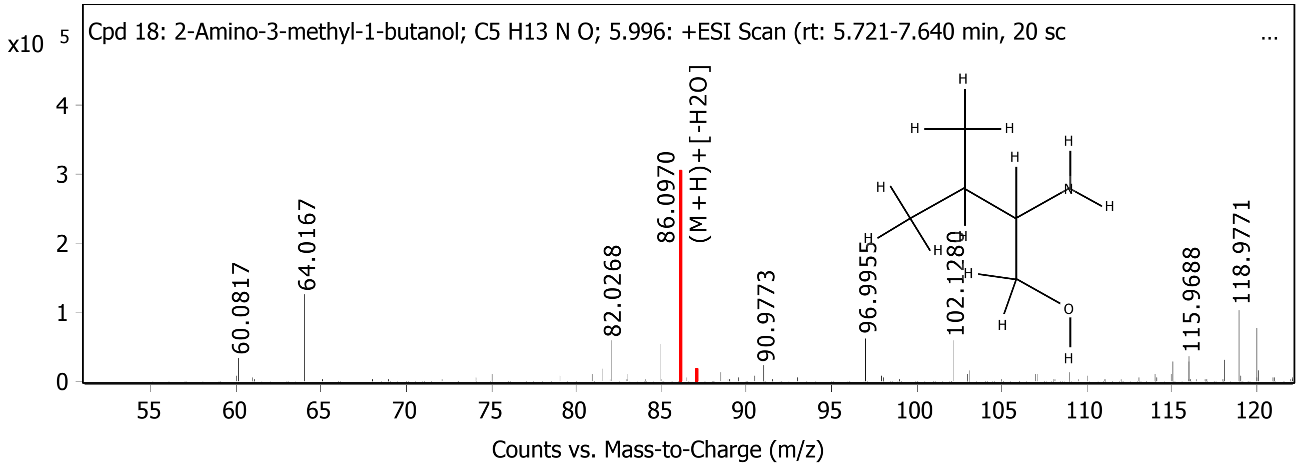

MSMS Spectrum

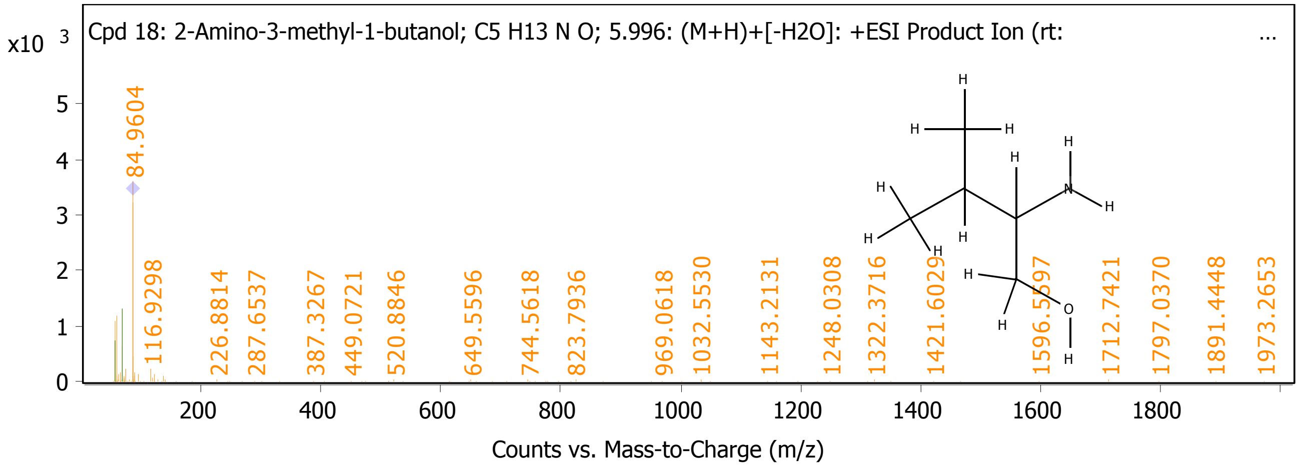

Identification Hit Table

Best Hit	Compound Name	RT	Formula	Notes	Match Score	Mass	Difference	Ion Species
✓	2-Amino-3-methyl-1-butanol	5.996	C5 H13 N O		85.46	103.1003	-0.6	(M+H)+[-H2O]
	3-Methylbutan-2-one	5.996	C5 H10 O		85.27	86.0738	-0.6	(M+NH4)+[-H2O]
	xi-3-Methyl-3-buten-2-ol	5.996	C5 H10 O		85.27	86.0738	-0.6	(M+NH4)+[-H2O]
	pentan-3-one	5.996	C5 H10 O		85.27	86.0738	-0.6	(M+NH4)+[-H2O]
	pentan-2-one	5.996	C5 H10 O		85.27	86.0738	-0.6	(M+NH4)+[-H2O]
	2-Methyl-3-buten-1-ol	5.996	C5 H10 O		85.27	86.0738	-0.6	(M+NH4)+[-H2O]
	2-Methylenebutan-1-ol	5.996	C5 H10 O		85.27	86.0738	-0.6	(M+NH4)+[-H2O]
	3-Methyl-2-buten-1-ol	5.996	C5 H10 O		85.27	86.0738	-0.6	(M+NH4)+[-H2O]
	3-Methyl-3-buten-1-ol	5.996	C5 H10 O		85.27	86.0738	-0.6	(M+NH4)+[-H2O]
	2Z-Penten-1-ol	5.996	C5 H10 O		85.27	86.0738	-0.6	(M+NH4)+[-H2O]

Qualitative Compound Identification Report

Identification Hit Table

Best Hit	Compound Name	RT	Formula	Notes	Match Score	Mass	Difference	Ion Species
✓	2-Methyl-3-buten-1-ol	5.996	C5 H10 O		85.27	86.0738	-0.6	(M+NH4)+[-H2O]
	2-Methylenebutan-1-ol	5.996	C5 H10 O		85.27	86.0738	-0.6	(M+NH4)+[-H2O]
	3-Methyl-2-buten-1-ol	5.996	C5 H10 O		85.27	86.0738	-0.6	(M+NH4)+[-H2O]
	3-Methyl-3-buten-1-ol	5.996	C5 H10 O		85.27	86.0738	-0.6	(M+NH4)+[-H2O]
	2Z-Penten-1-ol	5.996	C5 H10 O		85.27	86.0738	-0.6	(M+NH4)+[-H2O]
	Pentanal	5.996	C5 H10 O		85.27	86.0738	-0.6	(M+NH4)+[-H2O]
	pentan-3-one	5.996	C5 H10 O		85.27	86.0738	-0.6	(M+NH4)+[-H2O]
	pentan-2-one	5.996	C5 H10 O		85.27	86.0738	-0.6	(M+NH4)+[-H2O]
	3-Methylbutan-2-one	5.996	C5 H10 O		85.27	86.0738	-0.6	(M+NH4)+[-H2O]

Identification Hit Table

Best Hit	Compound Name	RT	Formula	Notes	Match Score	Mass	Difference	Ion Species

Compound Label	Name	*m/z*	RT	Algorithm	Mass
Cpd 19: MDAI; C10 H11 N O2; 8.766	**MDAI**	177.1021	8.766	Find by Molecular Feature	177.0788

Compound Chromatograms

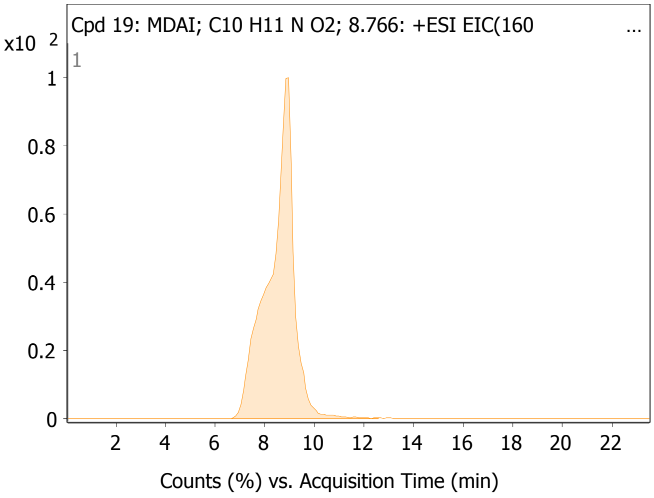

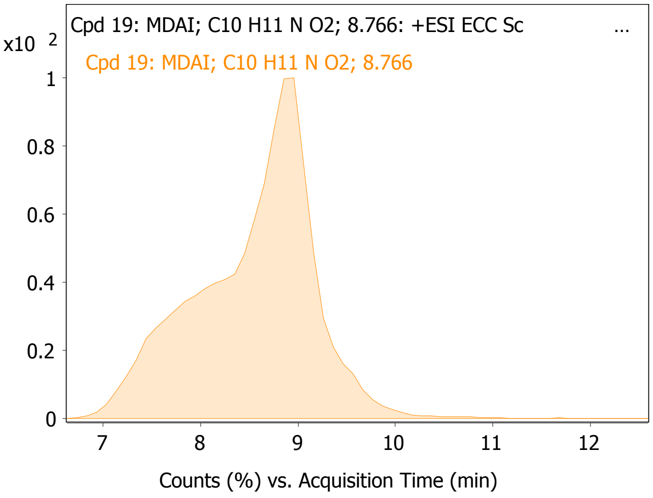

MFE MS Spectrum

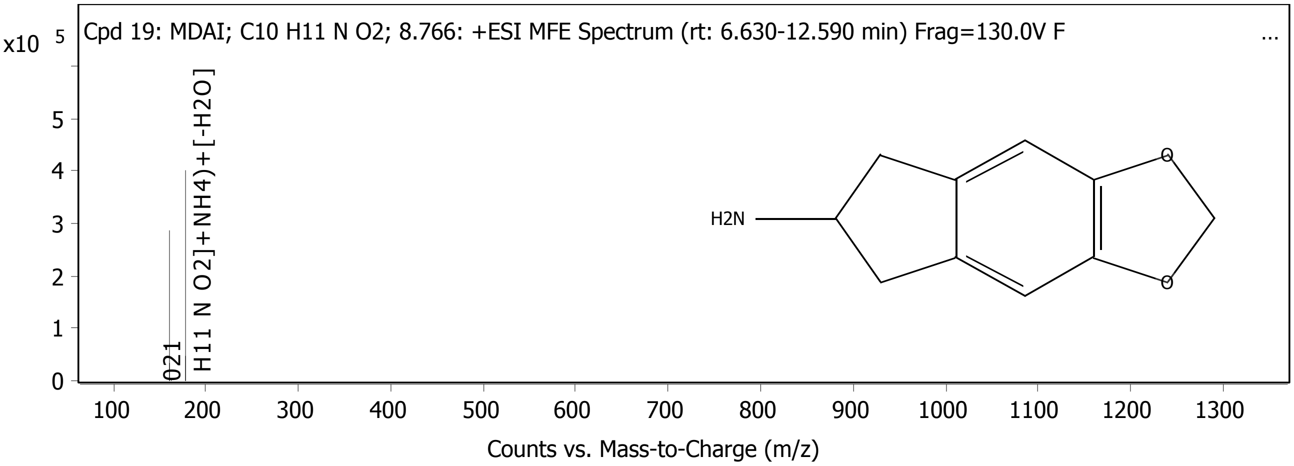

MFE MS Zoomed Spectrum

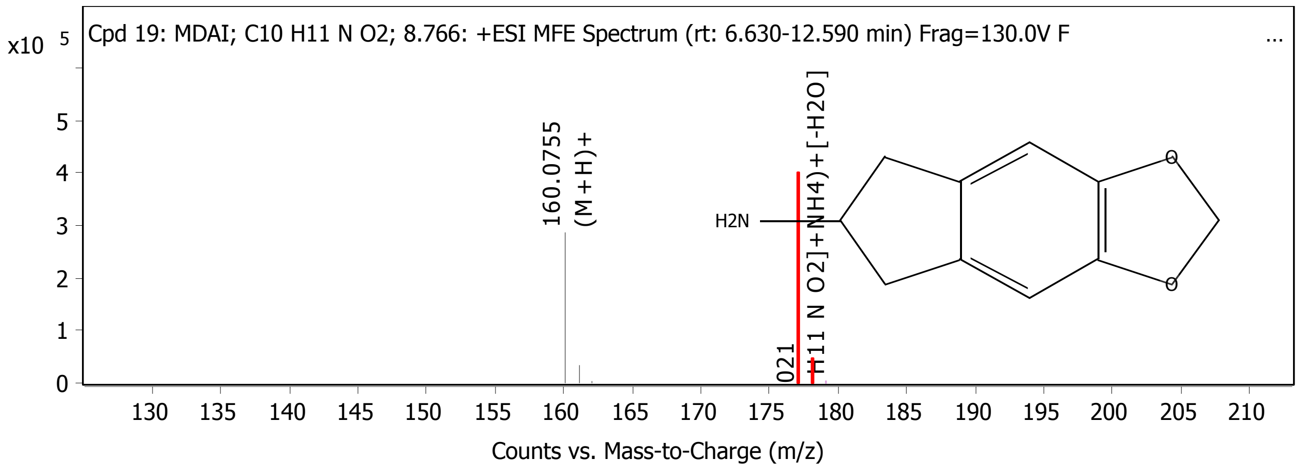

MS Spectrum

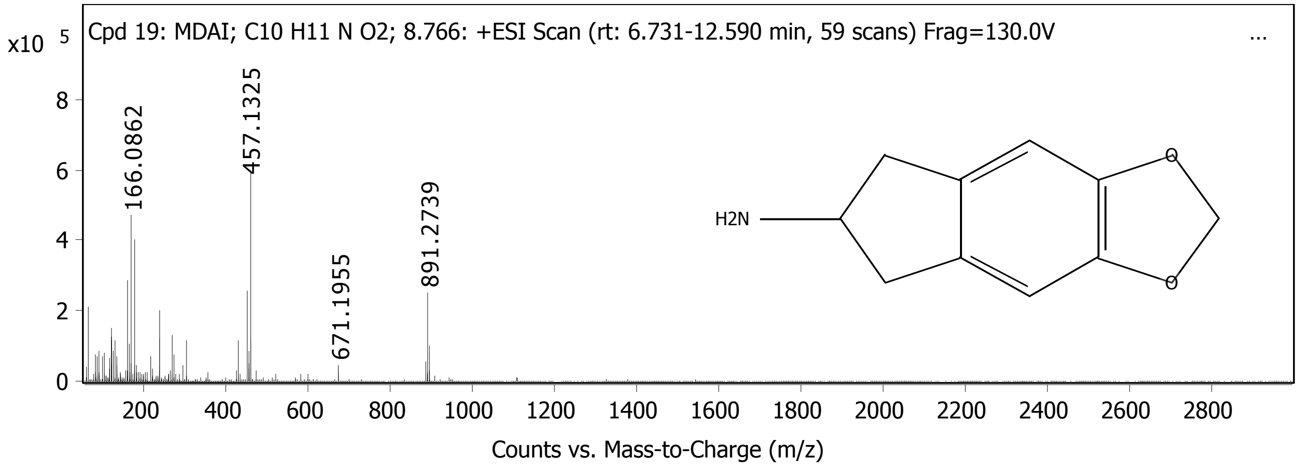

MS Zoomed Spectrum

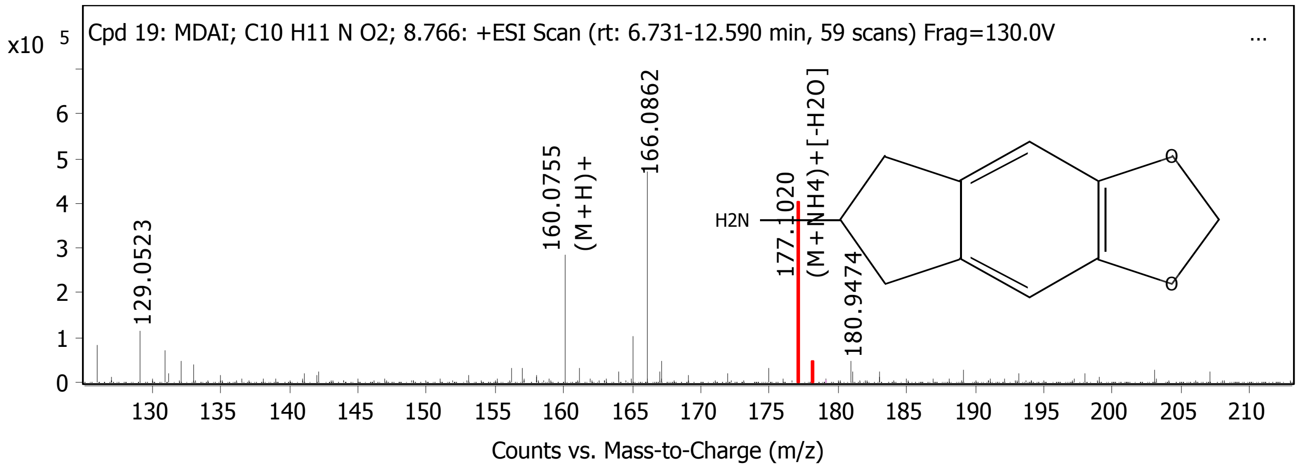

Identification Hit Table

Best Hit	Compound Name	RT	Formula	Notes	Match Score	Mass	Difference	Ion Species
✓	MDAI	8.766	C10 H11 N O2		87.6	177.0788	0.17	(M+NH4)+[-H2O]
	5-[2H-Pyrrol-4-(3H)-ylidenemethyl]-2-furanmethanol	8.766	C10 H11 N O2		87.6	177.0788	0.17	(M+NH4)+[-H2O]
	2-Propenyl 2-aminobenzoate	8.766	C10 H11 N O2		87.6	177.0788	0.17	(M+NH4)+[-H2O]
	Plantagonine	8.766	C10 H11 N O2		87.6	177.0788	0.17	(M+NH4)+[-H2O]
	N-Hydroxy-1-aminonaphthalene	8.766	C10 H9 N O		87.59	159.0682	0.17	(M+NH4)+
	Echinopsine	8.766	C10 H9 N O		87.59	159.0682	0.17	(M+NH4)+
	3-Methyl-quinolin-2-ol	8.766	C10 H9 N O		87.59	159.0682	0.17	(M+NH4)+
	6-Methoxyquinoline	8.766	C10 H9 N O	Pubmed 14860	87.59	159.0682	0.17	(M+NH4)+
	1-Acetylindole	8.766	C10 H9 N O	Pubchem 68470	87.59	159.0682	0.17	(M+NH4)+
	Indoleacetaldehyde	8.766	C10 H9 N O		87.59	159.0682	0.17	(M+NH4)+

Qualitative Compound Identification Report

Identification Hit Table

Best Hit	Compound Name	RT	Formula	Notes	Match Score	Mass	Difference	Ion Species

Identification Hit Table

Best Hit	Compound Name	RT	Formula	Notes	Match Score	Mass	Difference	Ion Species

Compound Label	Name	*m/z*	RT	Algorithm	Mass
Cpd 20: 5-Pentyloxazole; C8 H13 N O; 9.486	**5-Pentyloxazole**	122.0964	9.486	Find by Molecular Feature	139.0997

Compound Chromatograms

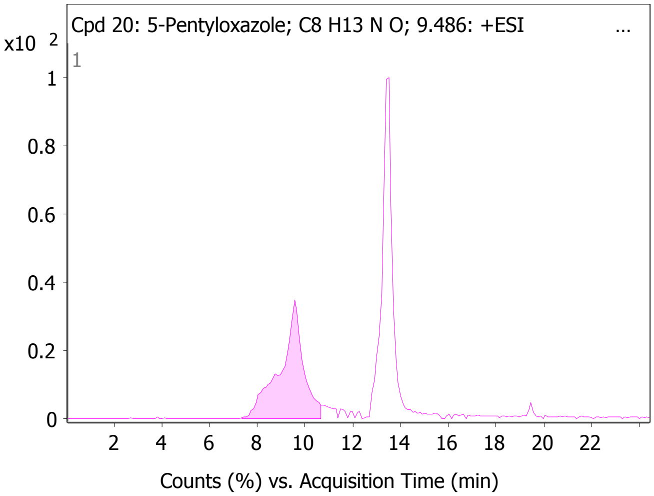

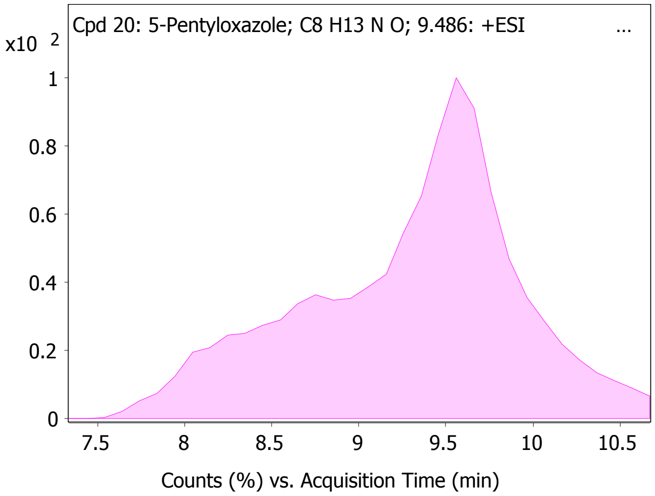

MFE MS Spectrum

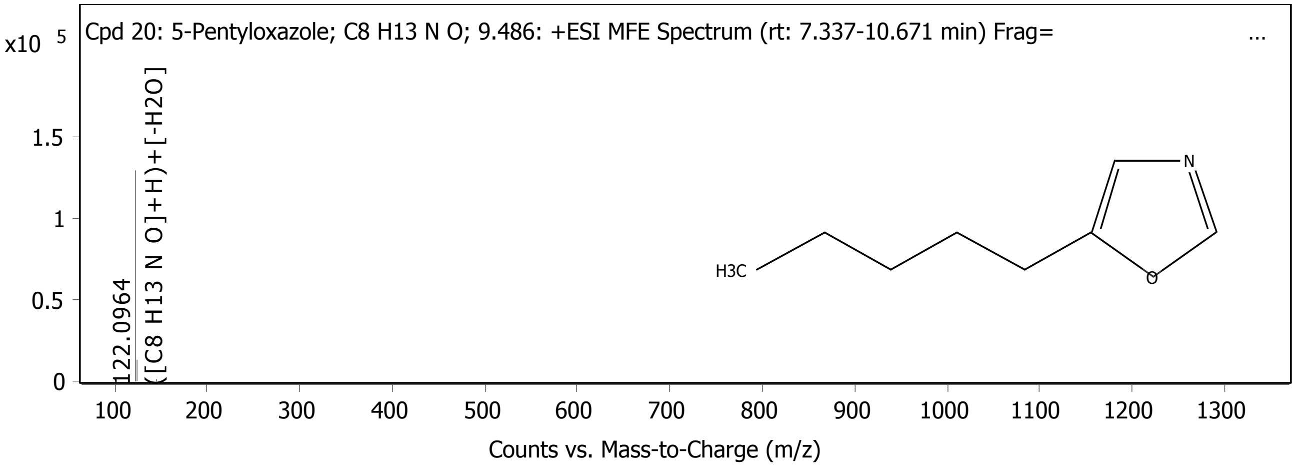

MFE MS Zoomed Spectrum

Qualitative Compound Identification Report

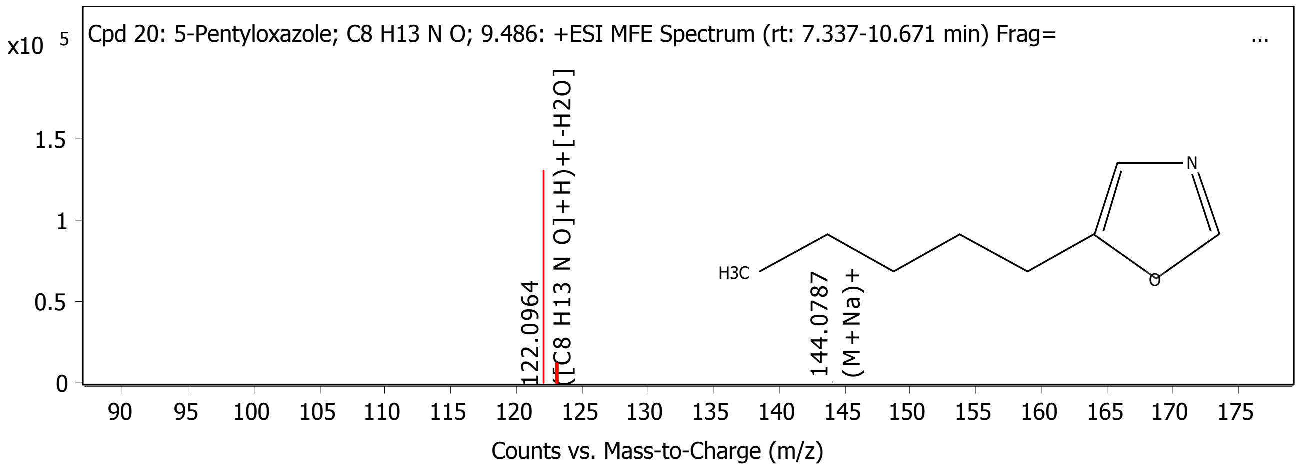

MS Spectrum

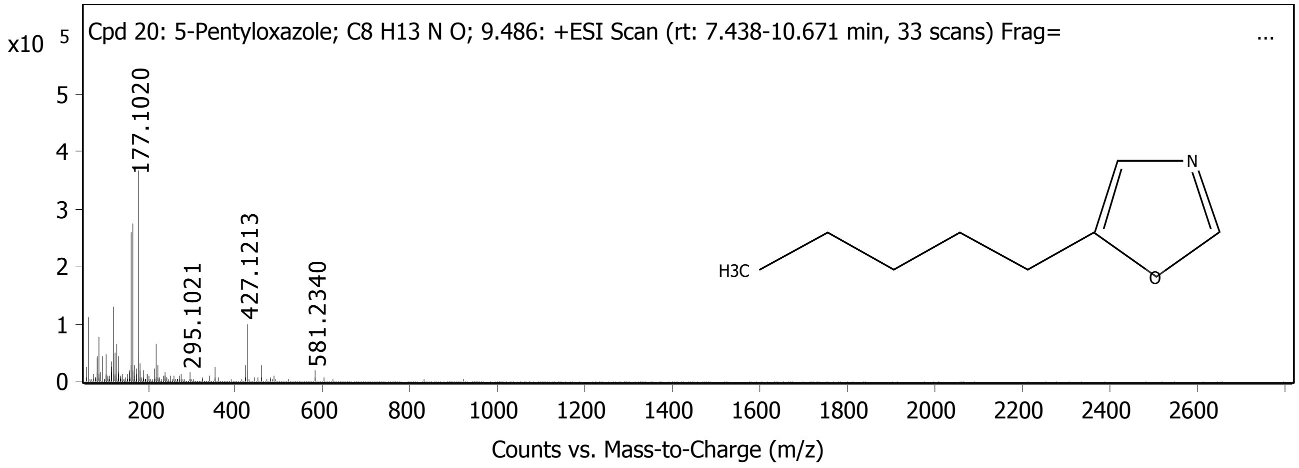

MS Zoomed Spectrum

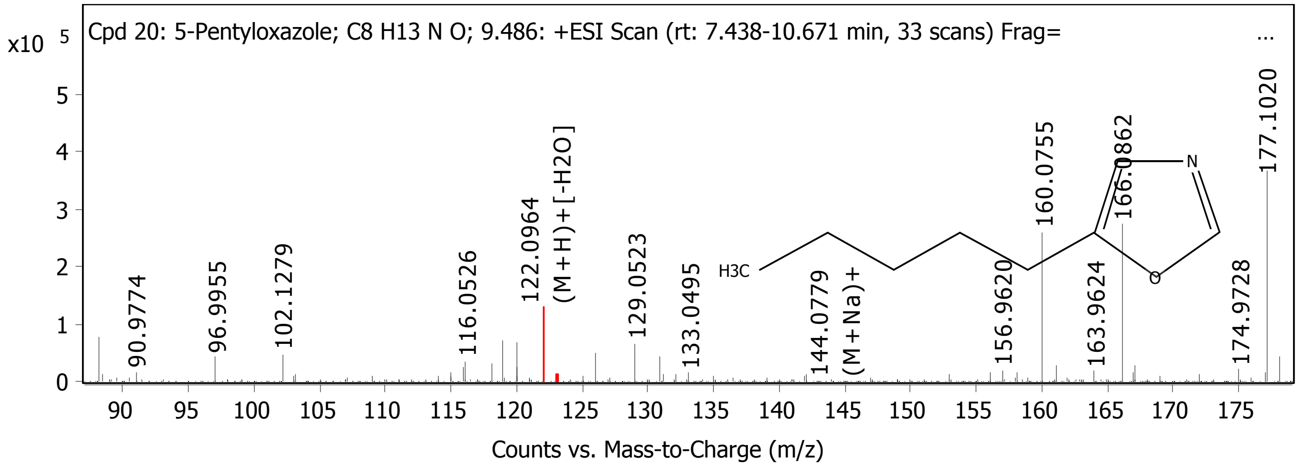

Identification Hit Table

Best Hit	Compound Name	RT	Formula	Notes	Match Score	Mass	Difference	Ion Species
✓	5-Pentyloxazole	9.486	C8 H13 N O		87.74	139.0997	0.01	(M+H)+[-H2O]
	1,2,3,4-Tetrahydro-6-propanoylpyridine	9.486	C8 H13 N O		87.74	139.0997	0.01	(M+H)+[-H2O]
	4,5-Dimethyl-2-(1-methylethyl)oxazole	9.486	C8 H13 N O		87.74	139.0997	0.01	(M+H)+[-H2O]
	1-Indolizidinone	9.486	C8 H13 N O		87.74	139.0997	0.01	(M+H)+[-H2O]
	Supinidine	9.486	C8 H13 N O		87.74	139.0997	0.01	(M+H)+[-H2O]
	9-Azabicyclo[3.3.1]nonan-3-one	9.486	C8 H13 N O		87.74	139.0997	0.01	(M+H)+[-H2O]
	Tropinone	9.486	C8 H13 N O		87.74	139.0997	0.01	(M+H)+[-H2O]
	2-Pentyloxazole	9.486	C8 H13 N O		87.74	139.0997	0.01	(M+H)+[-H2O]
	4-Butyl-2-methyloxazole	9.486	C8 H13 N O		87.74	139.0997	0.01	(M+H)+[-H2O]
	2,4-Dimethyl-5-propyloxazole	9.486	C8 H13 N O		87.74	139.0997	0.01	(M+H)+[-H2O]

Qualitative Compound Identification Report

Identification Hit Table

Best Hit	Compound Name	RT	Formula	Notes	Match Score	Mass	Difference	Ion Species

Identification Hit Table

Best Hit	Compound Name	RT	Formula	Notes	Match Score	Mass	Difference	Ion Species

Compound Label	Name	*m/z*	RT	Algorithm	Mass
Cpd 21: Ginkgotoxin; C9 H13 N O3; 10.692	**Ginkgotoxin**	166.0862	10.692	Find by Molecular Feature	183.0895

Compound Chromatograms

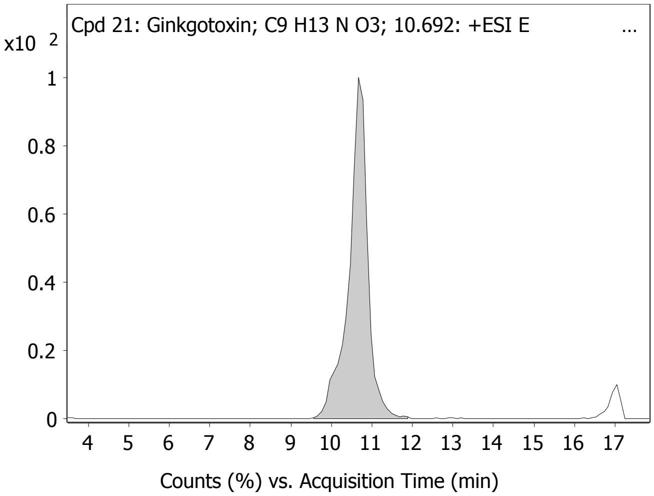

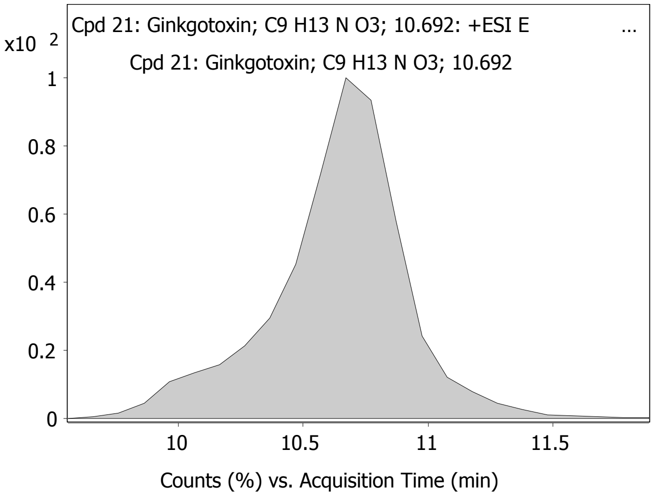

MFE MS Spectrum

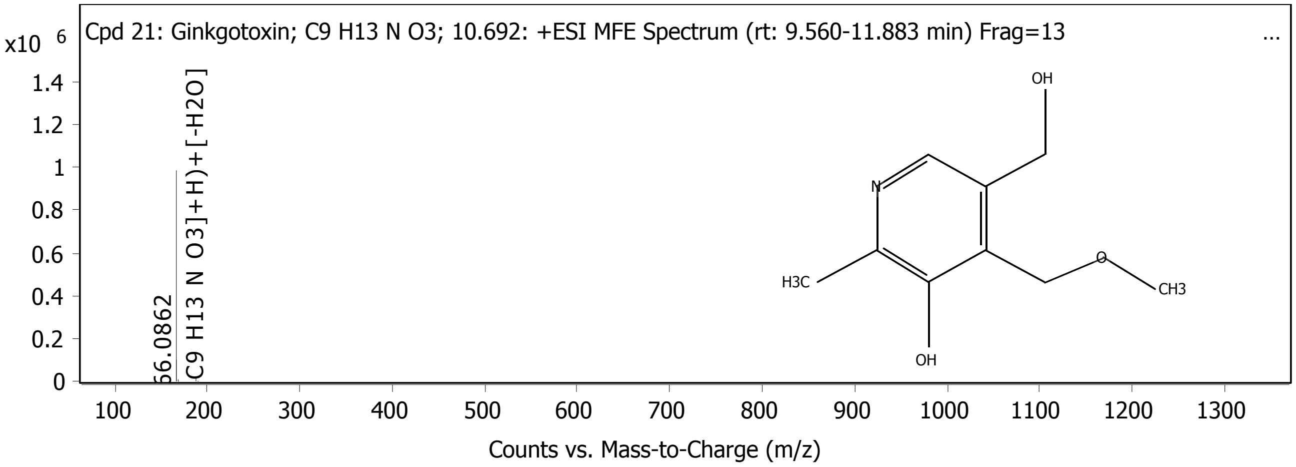

MFE MS Zoomed Spectrum

Qualitative Compound Identification Report

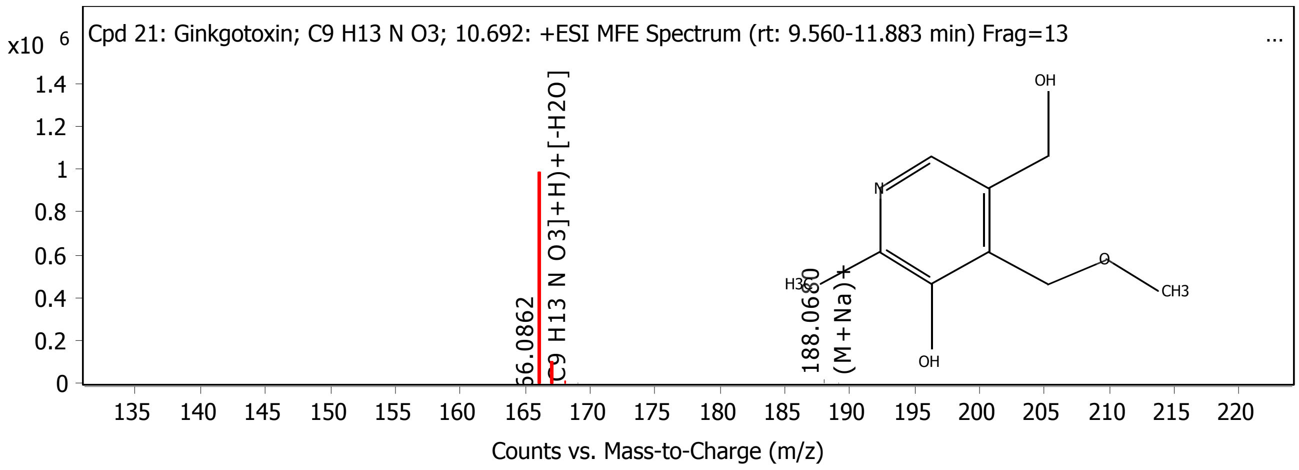

MS Spectrum

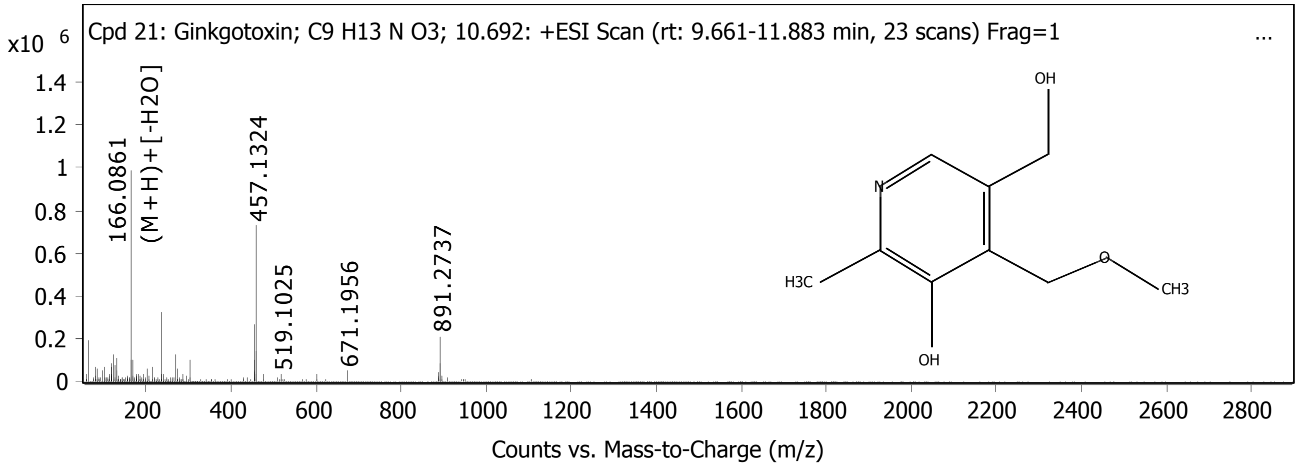

MS Zoomed Spectrum

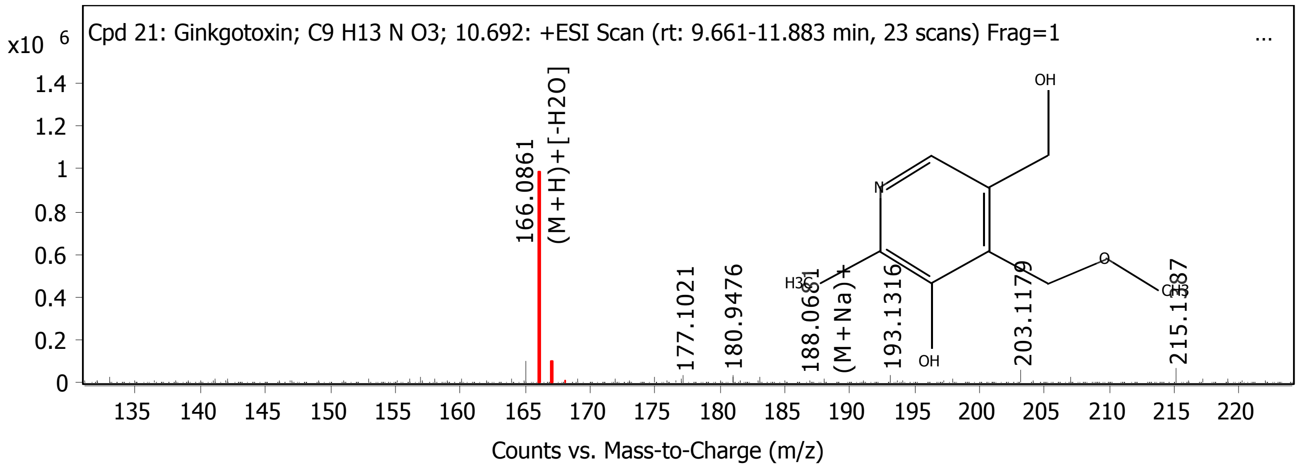

Identification Hit Table

Best Hit	Compound Name	RT	Formula	Notes	Match Score	Mass	Difference	Ion Species
✓	Ginkgotoxin	10.692	C9 H13 N O3		99.86	183.0895	0.04	(M+H)+[-H2O]
	Levonordefrin	10.692	C9 H13 N O3	Pubchem 164739	99.86	183.0895	0.04	(M+H)+[-H2O]
	Methylnoradrenaline	10.692	C9 H13 N O3	Antihypertension Metabolite of Methyldopa Dollery, Colin Therapeutic Drugs, 2nd Ed. 1999 p. M111	99.86	183.0895	0.04	(M+H)+[-H2O]
	Normetanephrine	10.692	C9 H13 N O3	Adjunctive use in local anesthesia Endogenous Metabolite of Norepinephrine Dollery, Colin Therapeutic Drugs, 2nd Ed. 1999 p. N129	99.86	183.0895	0.04	(M+H)+[-H2O]
	(-)-Epinephrine	10.692	C9 H13 N O3	Endogenous Metabolite Geigy vol. 3 p. 97	99.86	183.0895	0.04	(M+H)+[-H2O]

	Racepinephrine	10.692	C9 H13 N O3	Pubchem 838	99.86	183.0895	0.04	(M+H)+[-H2O]
	DL-Phenylalanine	10.692	C9 H11 N O2		99.85	165.0789	0.04	(M+H)+
	2-amino-4'-hydroxy-Propiophenone	10.692	C9 H11 N O2	anorectic drug, structurally related to, but with fewer sympathomimetic effects than, amphetamine. metabolite of Diethylpropion drug Dollery, Colin Therapeutic Drugs, 2nd Ed. 1999 p. D107	99.85	165.0789	0.04	(M+H)+
	2-Propylisonicotinic acid	10.692	C9 H11 N O2	Treatment of Tuberculosis and leprosy Metabolite of Protionamide Dollery, Colin Therapeutic Drugs, 2nd Ed. 1999 p. P270	99.85	165.0789	0.04	(M+H)+
	Benzocaine	10.692	C9 H11 N O2	The United States Pharmacopeia 29, The National Formulary 24, 2006	99.85	165.0789	0.04	(M+H)+

Identification Hit Table

Best Hit	Compound Name	RT	Formula	Notes	Match Score	Mass	Difference	Ion Species

Identification Hit Table

Best Hit	Compound Name	RT	Formula	Notes	Match Score	Mass	Difference	Ion Species

Compound Label	Name	*m/z*	RT	Algorithm	Mass
Cpd 22: His His His; C18 H23 N9 O4; 11.336	**His His His**	452.1768	11.336	Find by Molecular Feature	429.1878

Compound Chromatograms

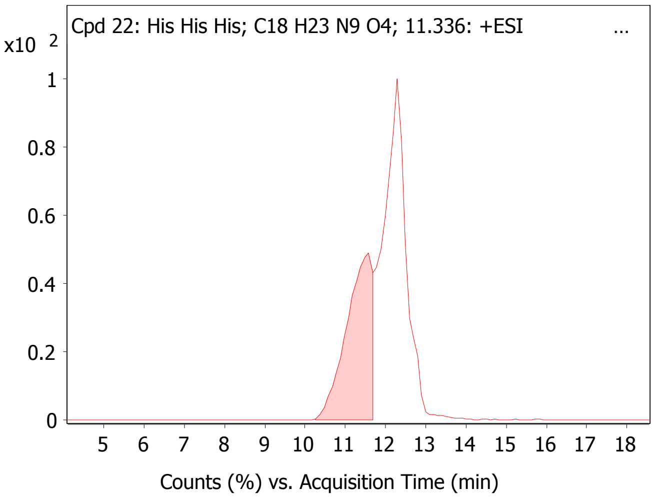

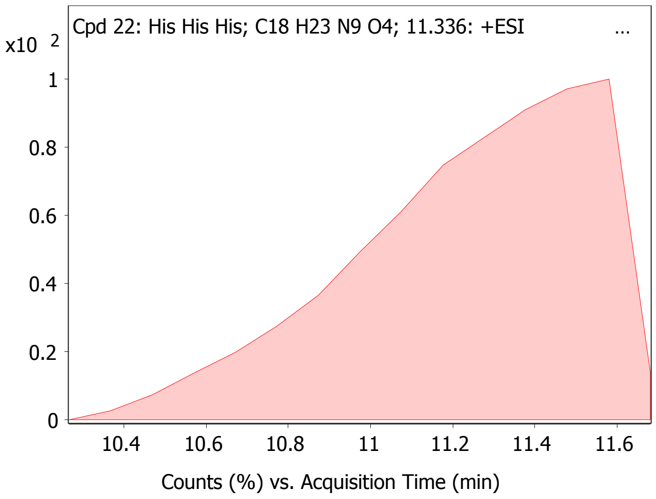

MFE MS Spectrum

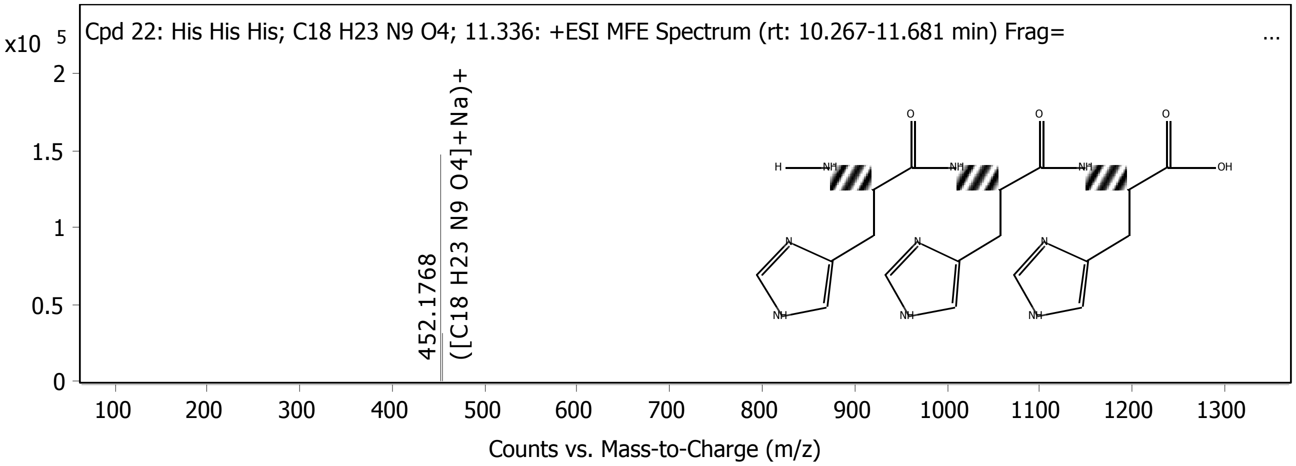

MFE MS Zoomed Spectrum

Qualitative Compound Identification Report

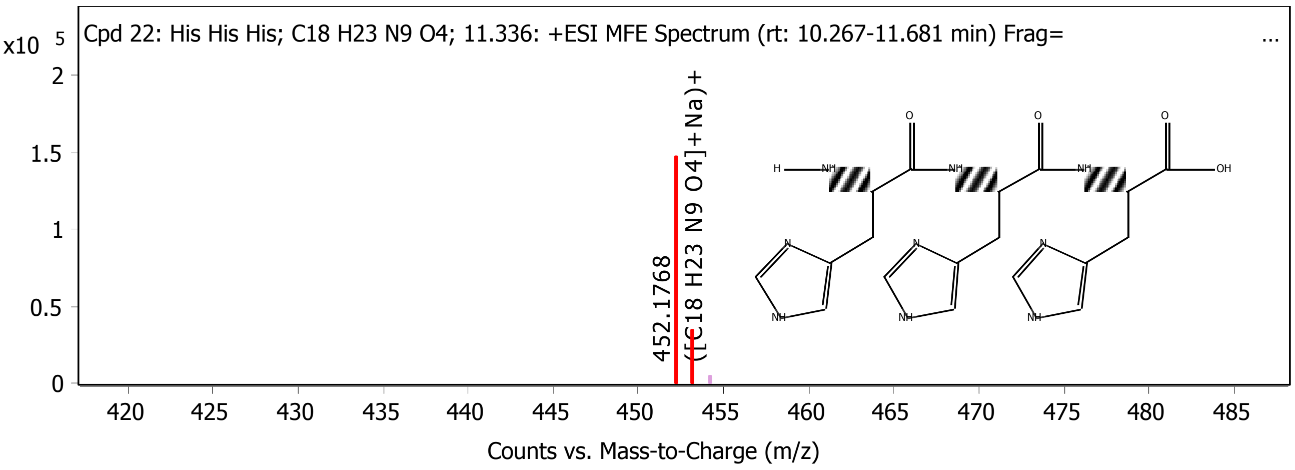

MS Spectrum

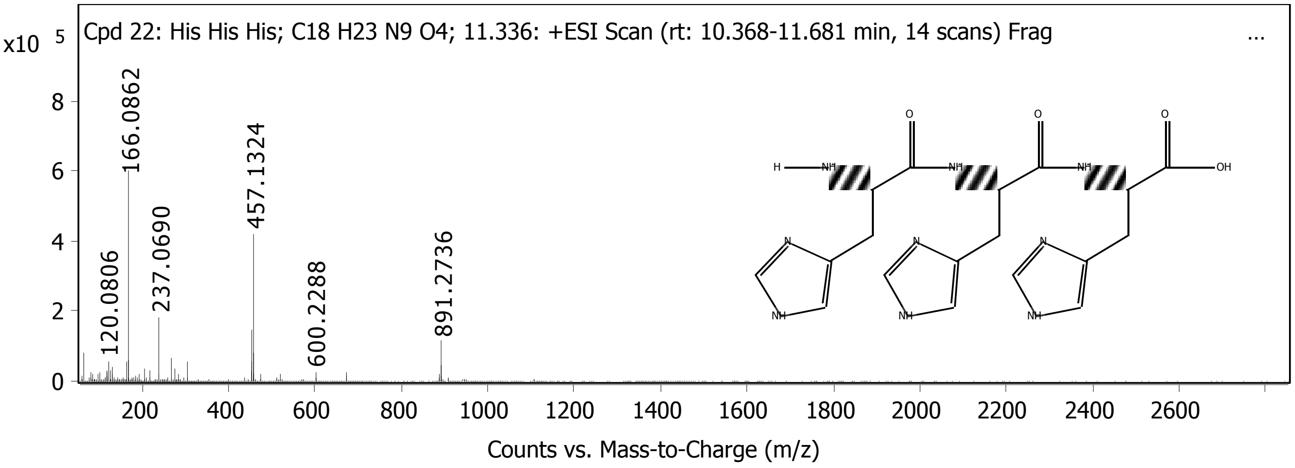

MS Zoomed Spectrum

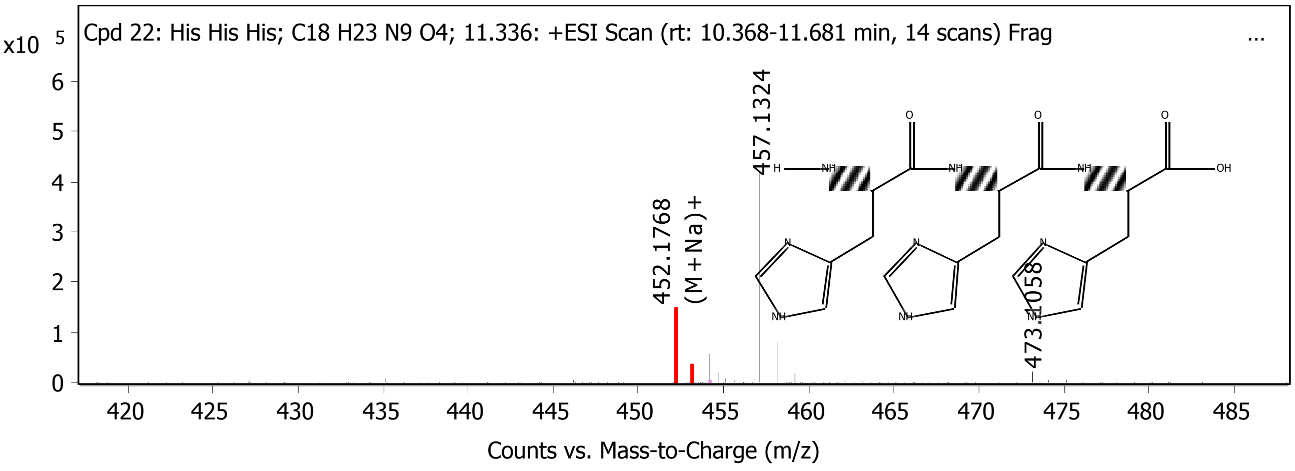

Identification Hit Table

Best Hit	Compound Name	RT	Formula	Notes	Match Score	Mass	Difference	Ion Species
✓	His His His	11.336	C18 H23 N9 O4		83.01	429.1878	-0.47	(M+Na)+
	Asp Asp Trp	11.336	C19 H22 N4 O8		81.62	434.1431	0.66	(M+NH4)+
	Asp Trp Asp	11.336	C19 H22 N4 O8		81.62	434.1431	0.66	(M+NH4)+
	Trp Asp Asp	11.336	C19 H22 N4 O8		81.62	434.1431	0.66	(M+NH4)+
	Galβ1-4GlcNAcβ-Sp	11.336	C16 H28 N4 O11	Negative MS/MS D10 from http://www.functionalglycomics.org/static/consortium/resources/resourcecored2.shtml#dissacharides	73.45	452.1775	-2.01	M+
	Galβ1-3GlcNAcβ-Sp	11.336	C16 H28 N4 O11	D8 from http://www.functionalglycomics.org/static/consortium/resources/resourcecored2.shtml#dissacharide	73.45	452.1775	-2.01	M+

Qualitative Compound Identification Report

				s				
	Tyr Thr Phe	11.336	C22 H27 N3 O6		69.31	429.1877	2.32	(M+Na)+
	Thr Tyr Phe	11.336	C22 H27 N3 O6		69.31	429.1877	2.32	(M+Na)+
	Thr Phe Tyr	11.336	C22 H27 N3 O6		69.31	429.1877	2.32	(M+Na)+
	Phe Thr Tyr	11.336	C22 H27 N3 O6		69.31	429.1877	2.32	(M+Na)+

Identification Hit Table

Best Hit	Compound Name	RT	Formula	Notes	Match Score	Mass	Difference	Ion Species
✓	16-hydroxy-18-bromo-8E,17E19Z-tricosatrien-4,6-diynoic acid	11.336	C23 H31 Br O3		45.39	434.143	2.63	(M+NH4)+
	Heteroartonin A	11.336	C26 H28 O7		35.65	452.1774	6.11	M+
	Artonin S	11.336	C26 H28 O7		35.65	452.1774	6.11	M+
	Dorsmanin D	11.336	C26 H28 O7		35.65	452.1774	6.11	M+
	Broussoflavonol B	11.336	C26 H28 O7		35.65	452.1774	6.11	M+
	5,7-Dihydroxy-8-methoxy-3,4'-diprenyloxyflavone	11.336	C26 H28 O7		35.65	452.1774	6.11	M+
	Derrichalcone	11.336	C26 H28 O7		35.65	452.1774	6.11	M+
	Derriflavanone	11.336	C26 H28 O7		35.65	452.1774	6.11	M+
	epi-Derriflavanone	11.336	C26 H28 O7		35.65	452.1774	6.11	M+
	3',4'-Dihydroxy-7-methoxy-8-(3-methylbut-2-enyl)-2'''-(1-hydroxy-1-methylethyl)-furano-(4'',5'':6,5)favanone	11.336	C26 H28 O7		35.65	452.1774	6.11	M+

Identification Hit Table

Best Hit	Compound Name	RT	Formula	Notes	Match Score	Mass	Difference	Ion Species
✓	His His His	11.336	C18 H23 N9 O4		83.01	429.1878	-0.47	(M+Na)+
	His Ser Asn Asn	11.336	C17 H26 N8 O8		82.18	470.1881	-0.74	M+[-H2O]
	Ser Asn Asn His	11.336	C17 H26 N8 O8		82.18	470.1881	-0.74	M+[-H2O]
	Asn His Asn Ser	11.336	C17 H26 N8 O8		82.18	470.1881	-0.74	M+[-H2O]
	Ser His Asn Asn	11.336	C17 H26 N8 O8		82.18	470.1881	-0.74	M+[-H2O]
	Asn Ser Asn His	11.336	C17 H26 N8 O8		82.18	470.1881	-0.74	M+[-H2O]
	Asn Ser His Asn	11.336	C17 H26 N8 O8		82.18	470.1881	-0.74	M+[-H2O]
	Ser Asn His Asn	11.336	C17 H26 N8 O8		82.18	470.1881	-0.74	M+[-H2O]
	Asn Asn His Ser	11.336	C17 H26 N8 O8		82.18	470.1881	-0.74	M+[-H2O]
	Asn His Ser Asn	11.336	C17 H26 N8 O8		82.18	470.1881	-0.74	M+[-H2O]

Compound Label	Name	*m/z*	RT	Algorithm	Mass
Cpd 23: Wilfordine; C43 H49 N O19; 11.437	**Wilfordine**	888.2734	11.437	Find by Molecular Feature	883.287

Compound Chromatograms

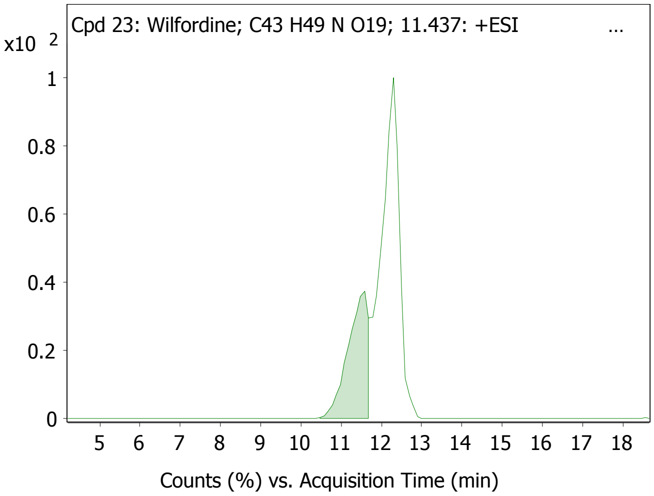

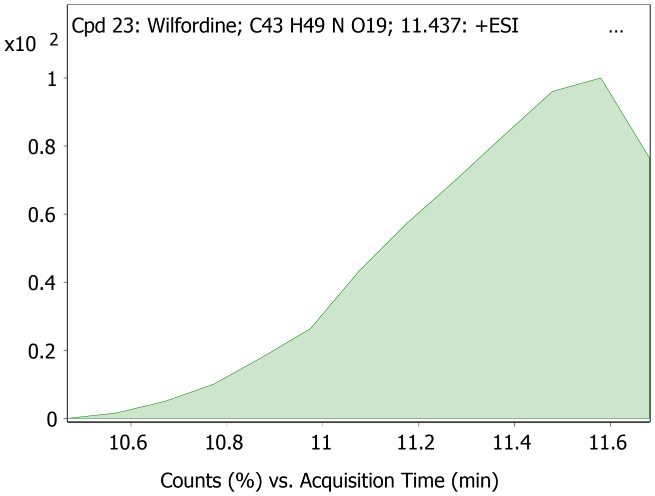

MFE MS Spectrum

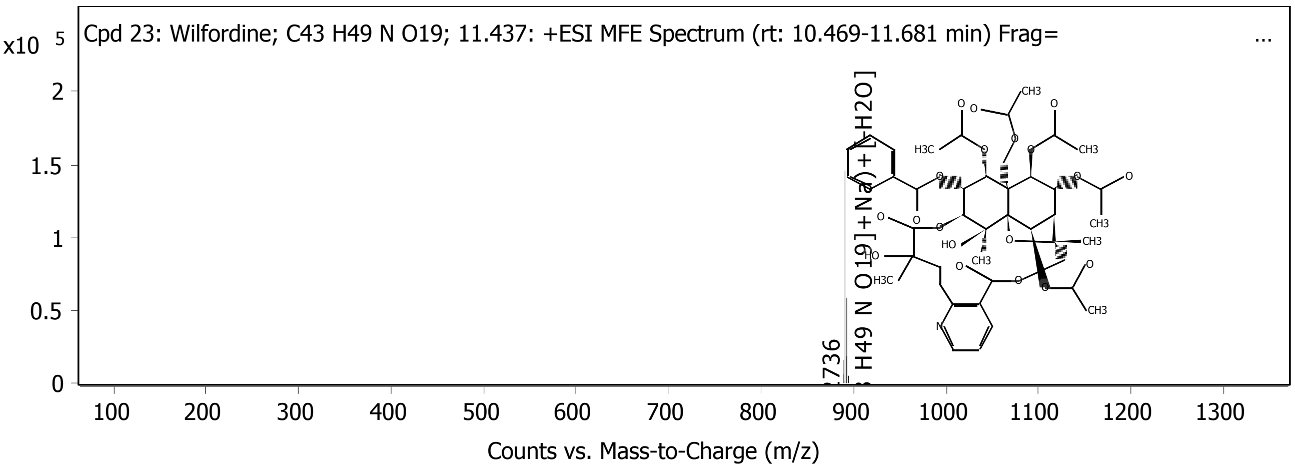

MFE MS Zoomed Spectrum

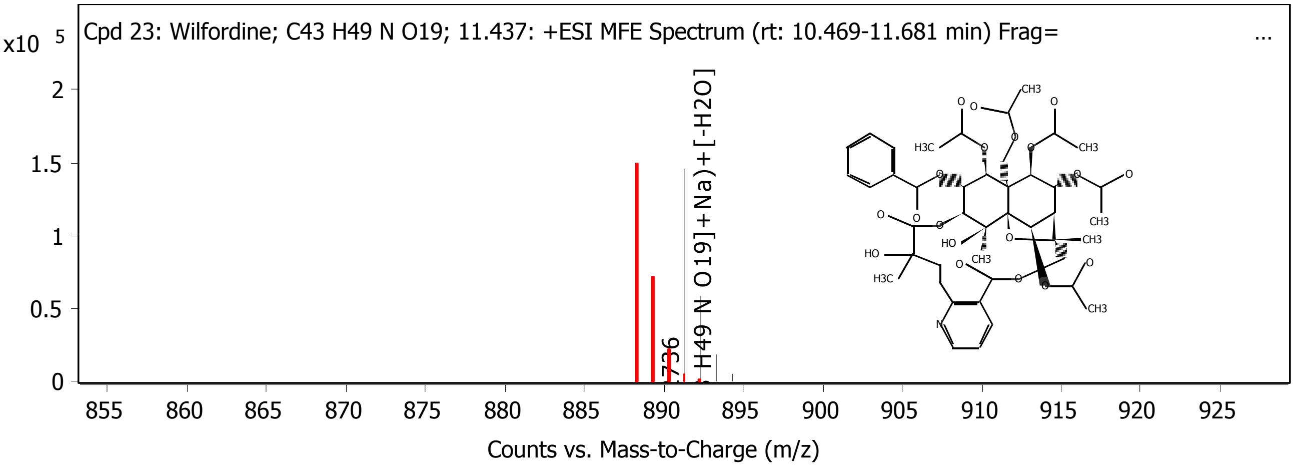

MS Spectrum

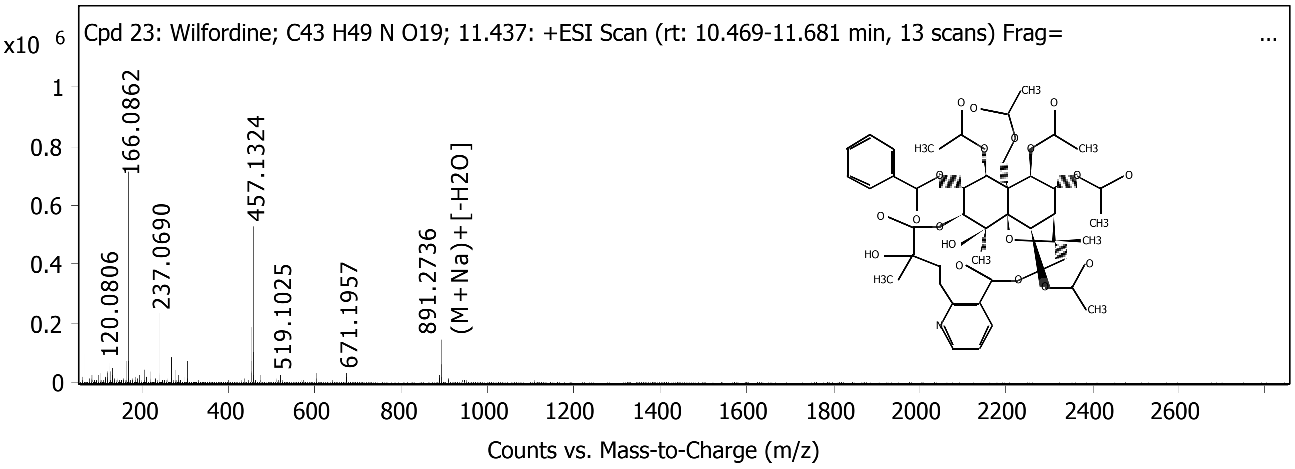

MS Zoomed Spectrum

Agilent Technologies

Qualitative Compound Identification Report

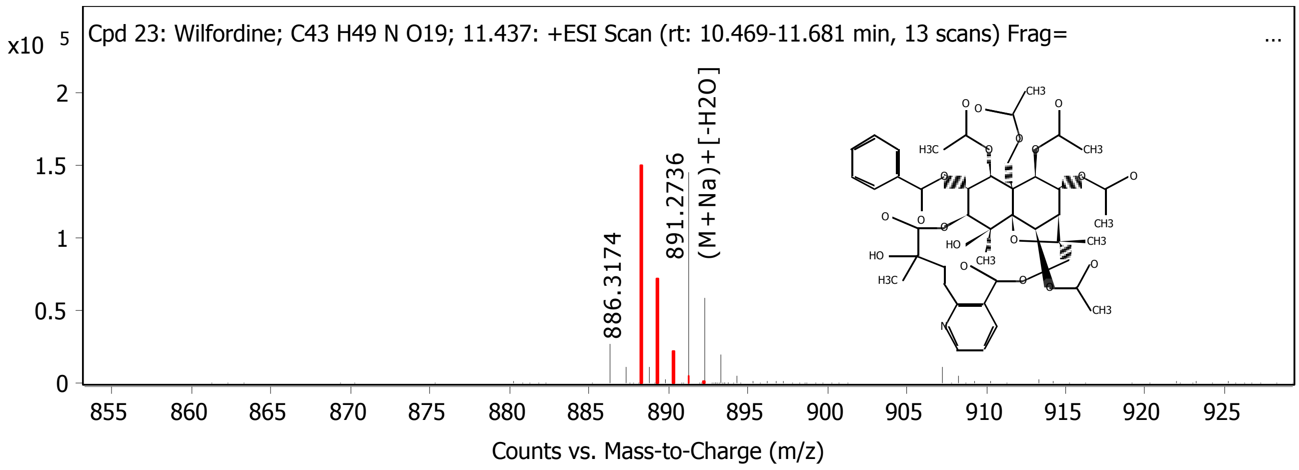

Identification Hit Table

Best Hit	Compound Name	RT	Formula	Notes	Match Score	Mass	Difference	Ion Species
✓	Wilfordine	11.437	C43 H49 N O19		56.79	883.287	2.84	(M+Na)+[-H2O]
	Coenzyme F430	11.437	C42 H51 N6 Ni O13		30.39	905.279	7.77	(M+H)+[-H2O]
	Quercetin 7-[xylosyl-(1->2)-rhamnosyl-(1->2)-rhamnosyl]-(1->6)-glucoside	11.437	C38 H48 O24		21.02	888.2427	10.82	(M+NH4)+[-H2O]
	Kaempferol 3-(2G-xylosylrutinoside)-7-glucoside	11.437	C38 H48 O24		21.02	888.2427	10.82	(M+NH4)+[-H2O]

Identification Hit Table

Best Hit	Compound Name	RT	Formula	Notes	Match Score	Mass	Difference	Ion Species
✓	Kaempferol 3-(2G-xylosylrutinoside)-7-glucoside	11.437	C38 H48 O24		21.02	888.2427	10.82	(M+NH4)+[-H2O]
	Quercetin 7-[xylosyl-(1->2)-rhamnosyl-(1->2)-rhamnosyl]-(1->6)-glucoside	11.437	C38 H48 O24		21.02	888.2427	10.82	(M+NH4)+[-H2O]

Identification Hit Table

Best Hit	Compound Name	RT	Formula	Notes	Match Score	Mass	Difference	Ion Species

Compound Label	Name	*m/z*	RT	Algorithm	Mass
Cpd 24: His Met Phe; C20 H27 N5 O4 S; 11.976	**His Met Phe**	454.1251	11.976	Find by Molecular Feature	433.1791

Compound Chromatograms

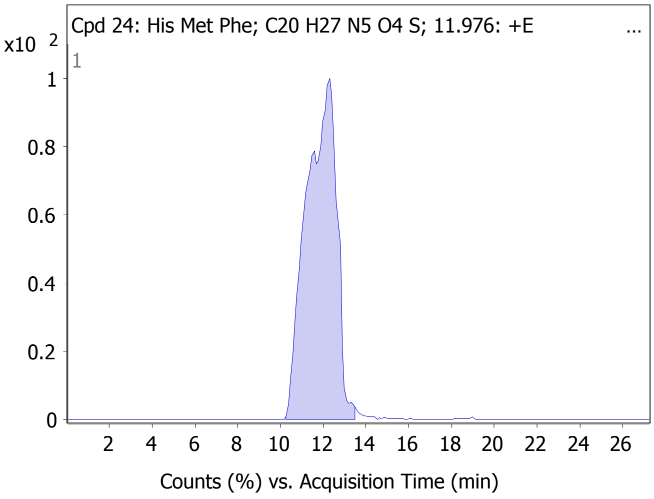

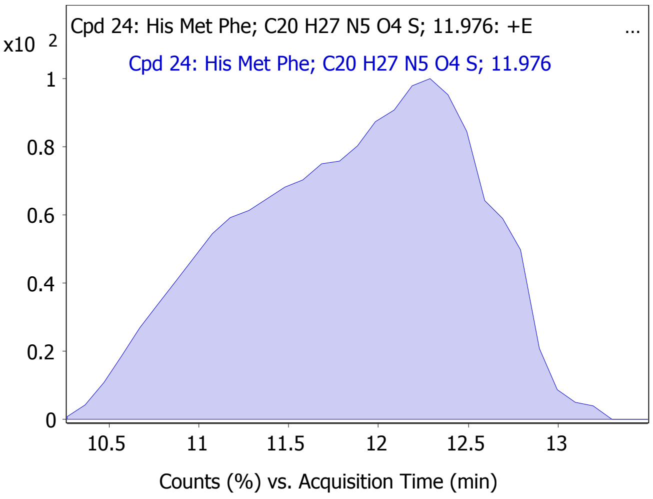

MFE MS Spectrum

Qualitative Compound Identification Report

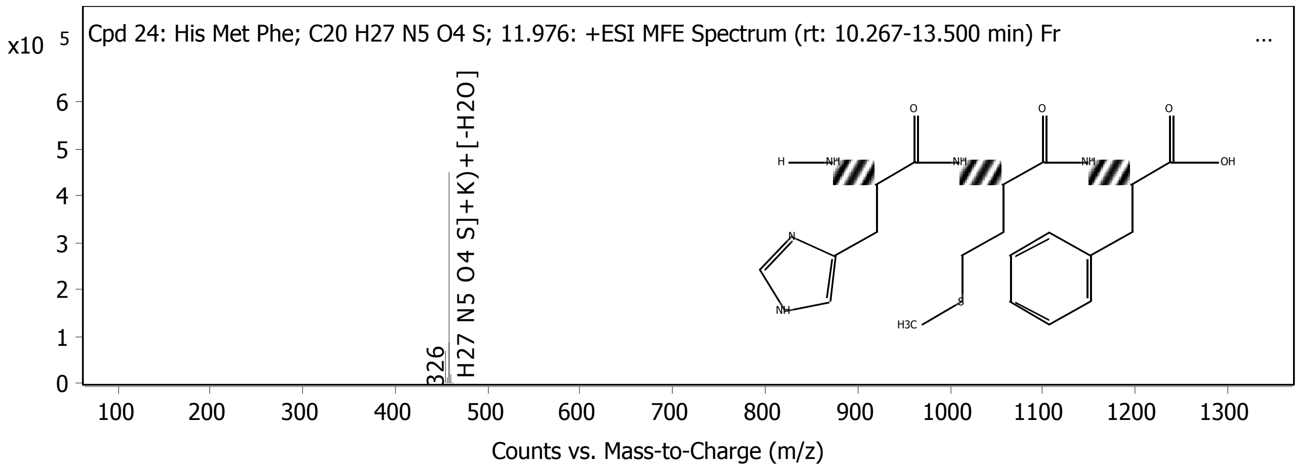

MFE MS Zoomed Spectrum

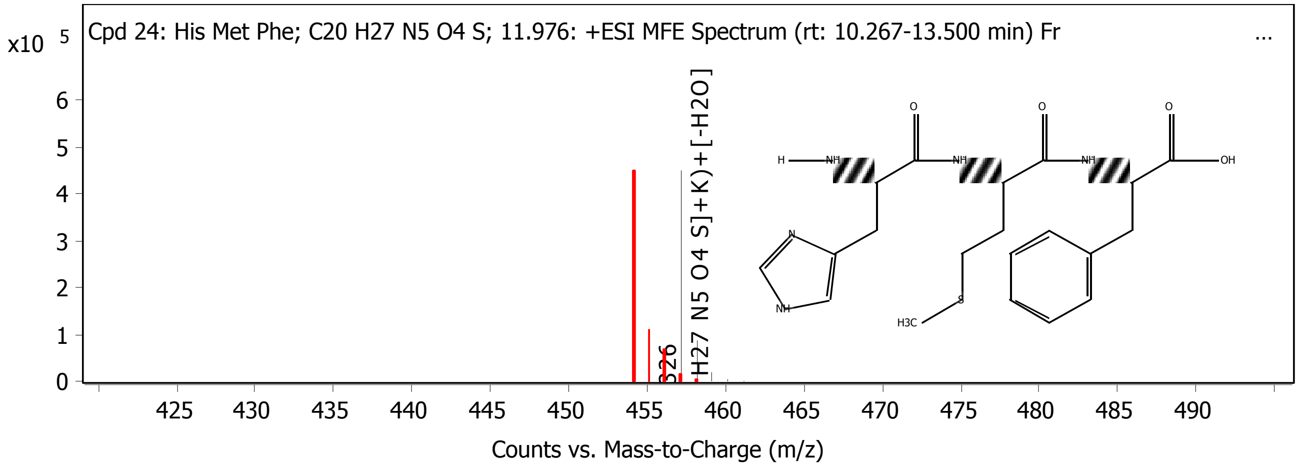

MS Spectrum

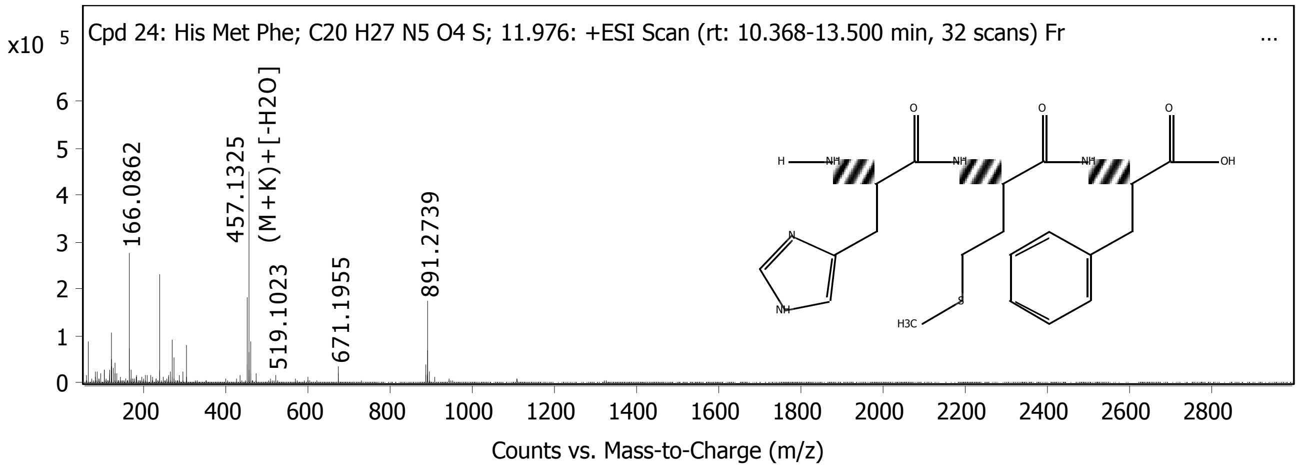

MS Zoomed Spectrum

Qualitative Compound Identification Report

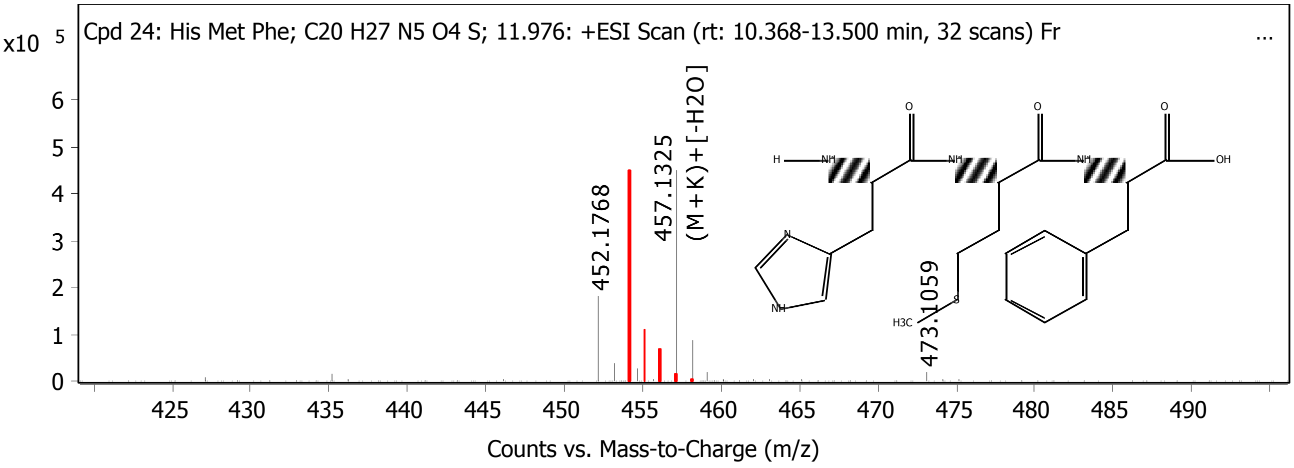

Identification Hit Table

Best Hit	Compound Name	RT	Formula	Notes	Match Score	Mass	Difference	Ion Species
✓	His Met Phe	11.976	C20 H27 N5 O4 S		69.97	433.1791	-0.72	(M+K)+[-H2O]
	Met Phe His	11.976	C20 H27 N5 O4 S		69.97	433.1791	-0.72	(M+K)+[-H2O]
	His Phe Met	11.976	C20 H27 N5 O4 S		69.97	433.1791	-0.72	(M+K)+[-H2O]
	Met His Phe	11.976	C20 H27 N5 O4 S		69.97	433.1791	-0.72	(M+K)+[-H2O]
	Phe Met His	11.976	C20 H27 N5 O4 S		69.97	433.1791	-0.72	(M+K)+[-H2O]
	Phe His Met	11.976	C20 H27 N5 O4 S		69.97	433.1791	-0.72	(M+K)+[-H2O]
	Kinamycin D	11.976	C22 H18 N2 O9		68.24	454.1014	-0.19	(M+NH4)+[-H2O]
	Formononetin 7-O-(6''-acetylglcoside)	11.976	C24 H24 O10		62.53	472.1354	1.6	M+[-H2O]
	Margrapine B	11.976	C22 H25 N O7		53.8	415.1659	-2.83	(M+K)+
	N-Desmethylnefopam glucuronide	11.976	C22 H25 N O7	Analgesic Metabolite of Nefopam Dollery, Colin Therapeutic Drugs, 2nd Ed. 1999 p. N46	53.8	415.1659	-2.83	(M+K)+

Identification Hit Table

Best Hit	Compound Name	RT	Formula	Notes	Match Score	Mass	Difference	Ion Species
✓	Formononetin 7-O-(6''-acetylglcoside)	11.976	C24 H24 O10		62.53	472.1354	1.6	M+[-H2O]

Identification Hit Table

Best Hit	Compound Name	RT	Formula	Notes	Match Score	Mass	Difference	Ion Species
✓	His Met Phe	11.976	C20 H27 N5 O4 S		69.97	433.1791	-0.72	(M+K)+[-H2O]
	Met Phe His	11.976	C20 H27 N5 O4 S		69.97	433.1791	-0.72	(M+K)+[-H2O]
	His Phe Met	11.976	C20 H27 N5 O4 S		69.97	433.1791	-0.72	(M+K)+[-H2O]
	Met His Phe	11.976	C20 H27 N5 O4 S		69.97	433.1791	-0.72	(M+K)+[-H2O]
	Phe Met His	11.976	C20 H27 N5 O4 S		69.97	433.1791	-0.72	(M+K)+[-H2O]
	Phe His Met	11.976	C20 H27 N5 O4 S		69.97	433.1791	-0.72	(M+K)+[-H2O]

Compound Label	Name	*m/z*	RT	Algorithm	Mass
Cpd 25: Glycerol 1-propanoate diacetate; C10 H16 O6; 12.154	**Glycerol 1-propanoate diacetate**	237.0732	12.154	Find by Molecular Feature	232.0948

Compound Chromatograms

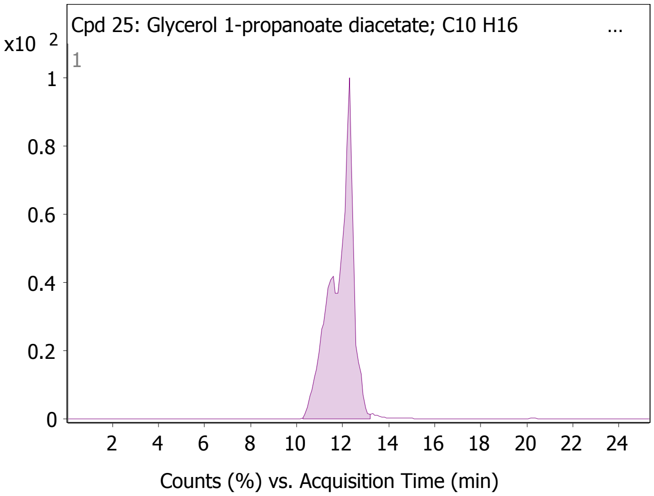

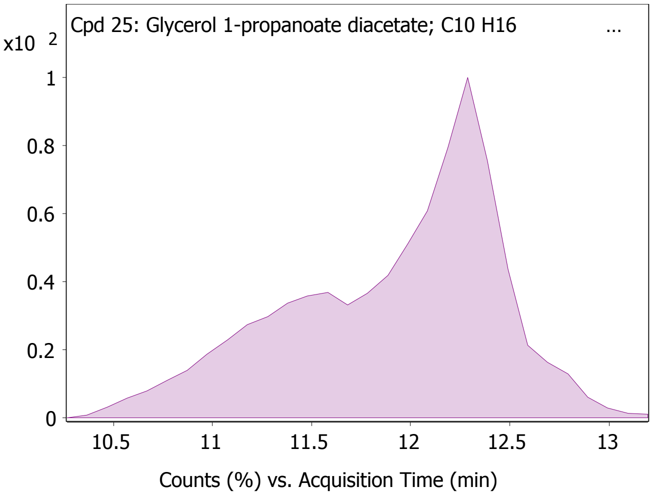

MFE MS Spectrum

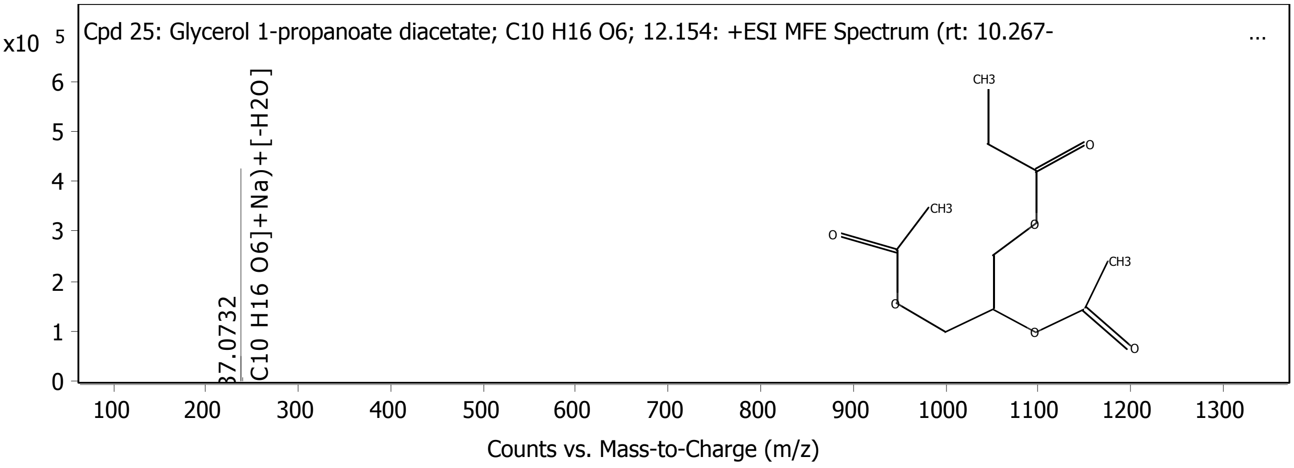

MFE MS Zoomed Spectrum

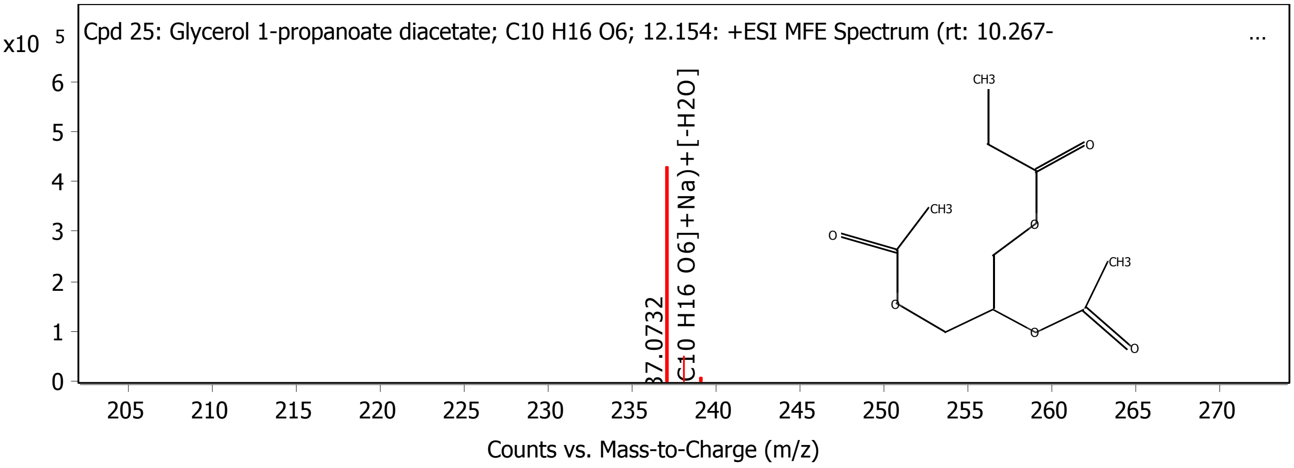

MS Spectrum

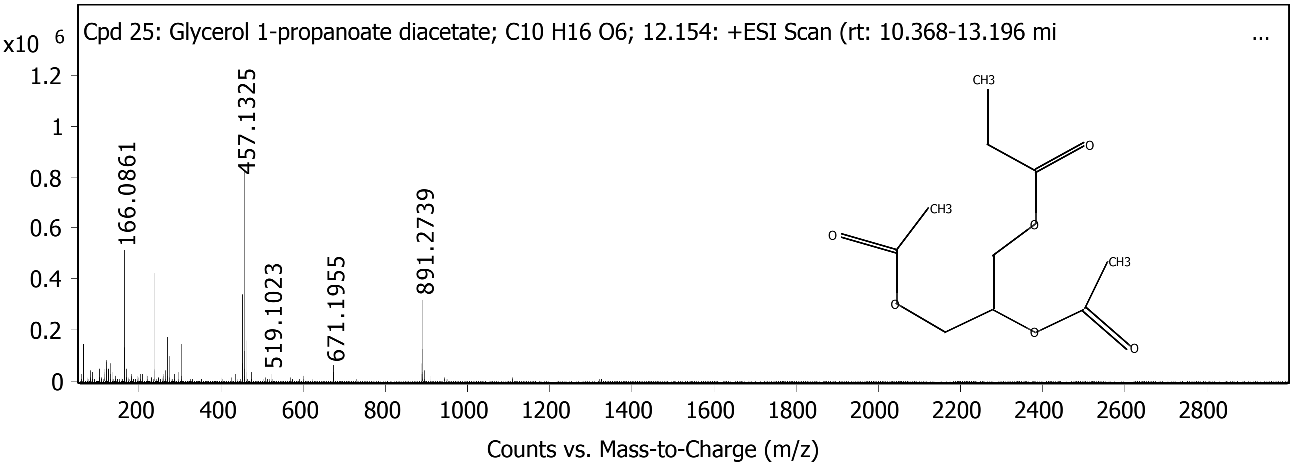

MS Zoomed Spectrum

Qualitative Compound Identification Report

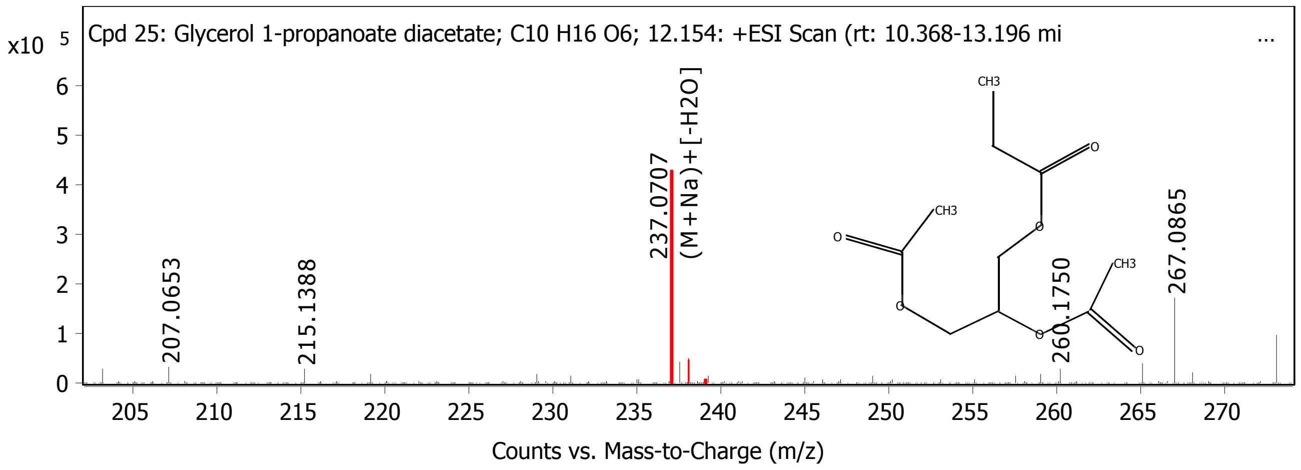

Identification Hit Table

Best Hit	Compound Name	RT	Formula	Notes	Match Score	Mass	Difference	Ion Species
✓	Glycerol 1-propanoate diacetate	12.154	C10 H16 O6		94.96	232.0948	-0.09	(M+Na)+[-H2O]
	Todralazine	12.154	C11 H12 N4 O2	Pubchem 5501 antihypertensive, peripheral vasodilator	86.62	232.0949	1.18	(M+Na)+[-H2O]
	L-Arginine phosphate	12.154	C6 H15 N4 O5 P		82.47	254.0768	1.16	(M+H)+[-H2O]
	4-Amino-2-methyl-5-phosphomethylpyrimidine	12.154	C6 H10 N3 O4 P	Endogenous Metabolite http://dbk.ch.umist.ac.uk/ExactMasses.htm	81.87	219.0397	1.16	(M+NH4)+
	(S)-2,3-Dihydro-7-hydroxy-2-methyl-4-oxo-4H-1-benzopyran-5-acetic acid	12.154	C12 H12 O5		76.46	236.0662	2.32	(M+H)+
	Apional	12.154	C12 H12 O5		76.46	236.0662	2.32	(M+H)+
	Austdiol	12.154	C12 H12 O5		76.46	236.0662	2.32	(M+H)+
	Dillapional	12.154	C12 H12 O5		76.46	236.0662	2.32	(M+H)+
	Radicinin	12.154	C12 H12 O5		76.46	236.0662	2.32	(M+H)+
	Orthosporin	12.154	C12 H12 O5		76.46	236.0662	2.32	(M+H)+

Identification Hit Table

Best Hit	Compound Name	RT	Formula	Notes	Match Score	Mass	Difference	Ion Species

Identification Hit Table

Best Hit	Compound Name	RT	Formula	Notes	Match Score	Mass	Difference	Ion Species

Compound Label	Name	*m/z*	RT	Algorithm	Mass
Cpd 26: His His His; C18 H23 N9 O4; 12.220	**His His His**	452.1769	12.22	Find by Molecular Feature	429.1877

Compound Chromatograms

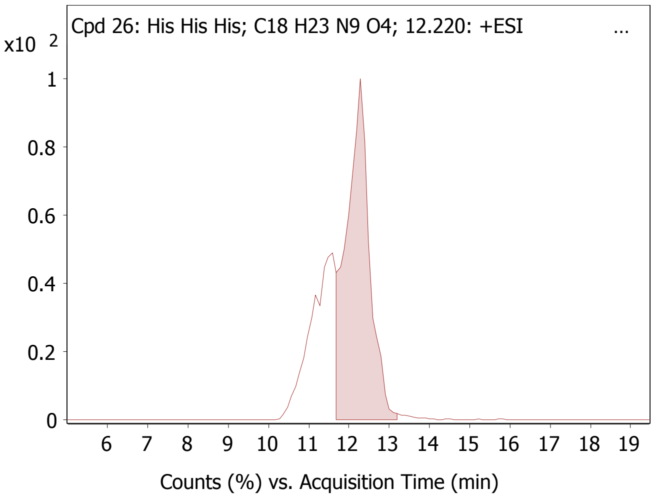

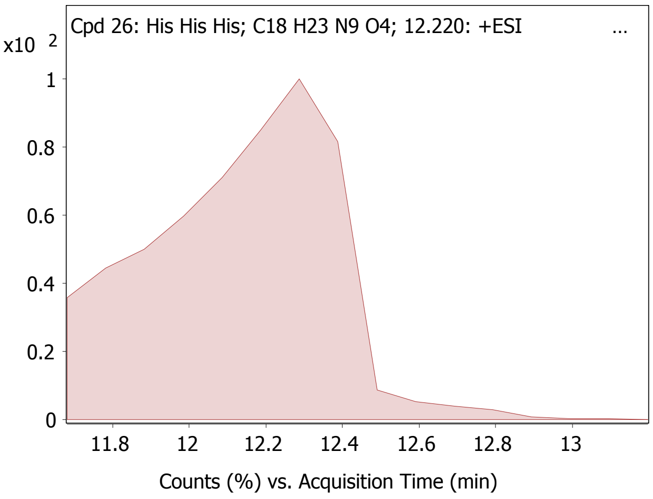

MFE MS Spectrum

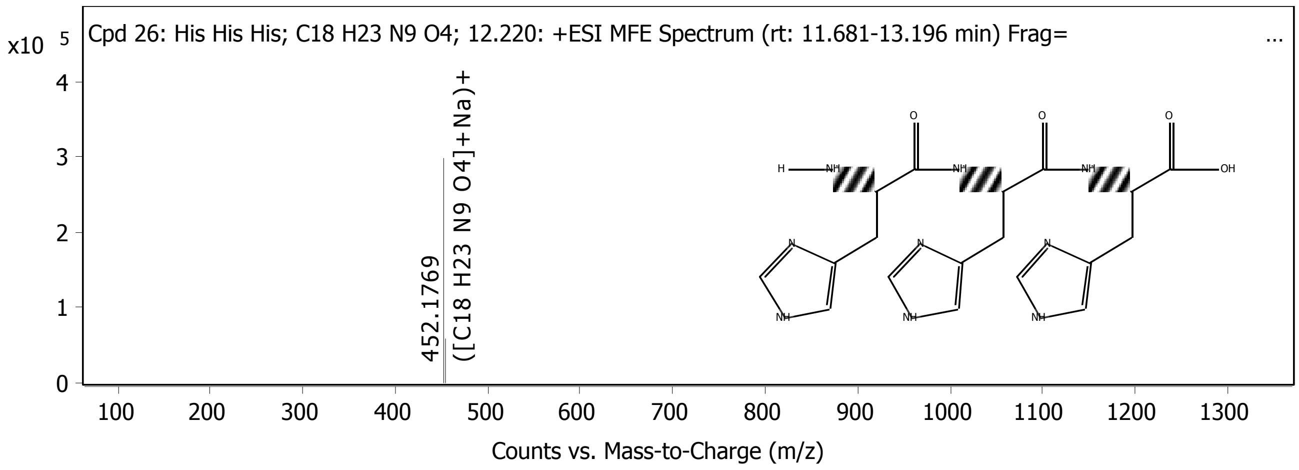

MFE MS Zoomed Spectrum

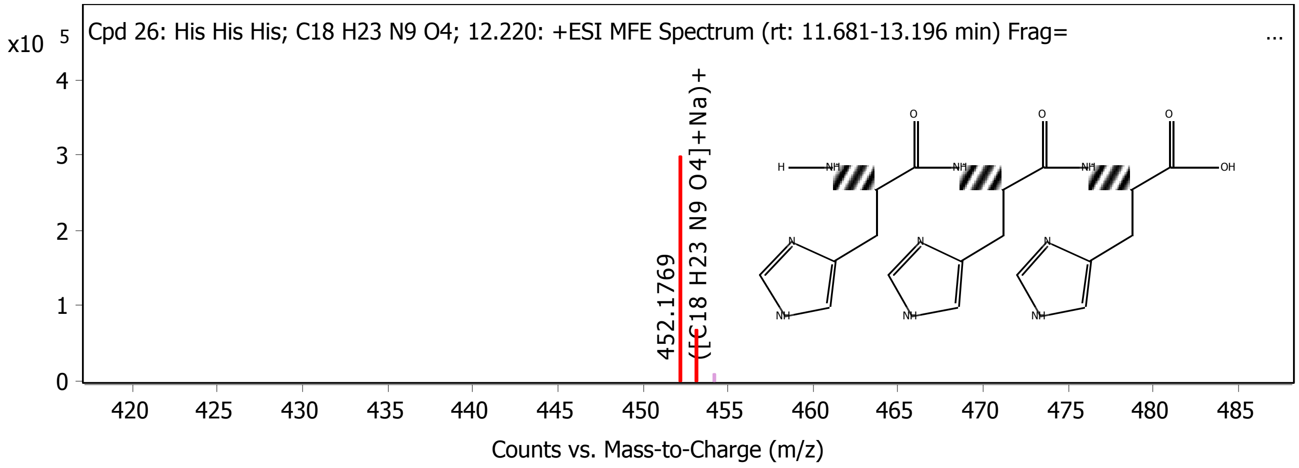

MS Spectrum

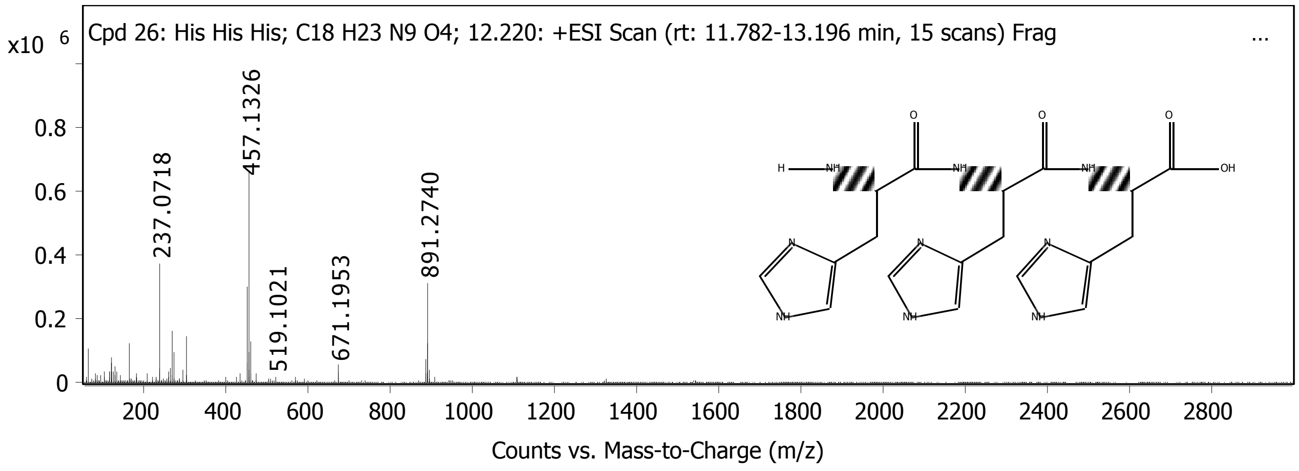

MS Zoomed Spectrum

Qualitative Compound Identification Report

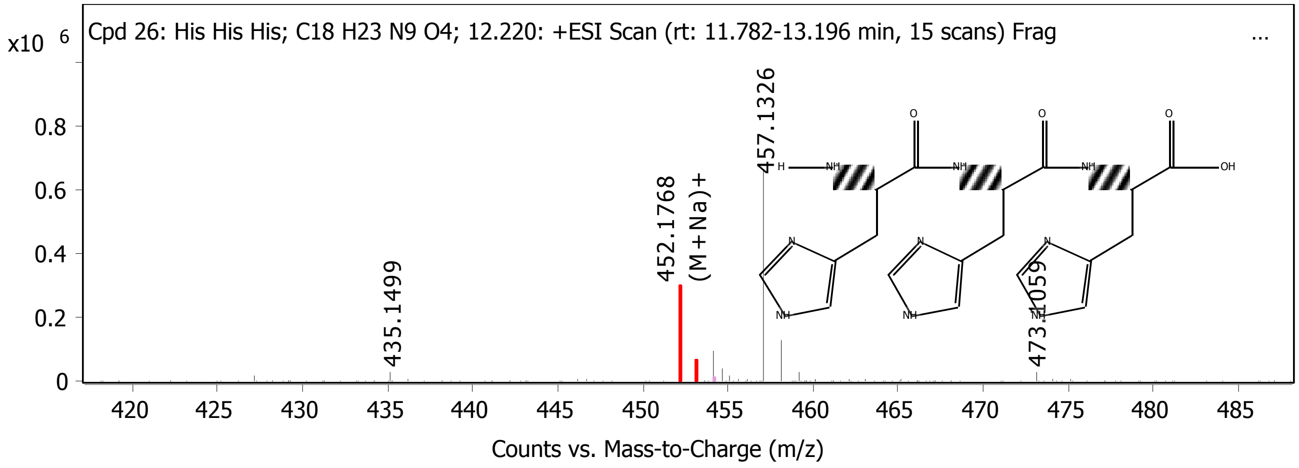

Identification Hit Table

Best Hit	Compound Name	RT	Formula	Notes	Match Score	Mass	Difference	Ion Species
✓	His His His	12.22	C18 H23 N9 O4		82.98	429.1877	-0.42	(M+Na)+
	Asp Asp Trp	12.22	C19 H22 N4 O8		81.11	434.143	0.72	(M+NH4)+
	Trp Asp Asp	12.22	C19 H22 N4 O8		81.11	434.143	0.72	(M+NH4)+
	Asp Trp Asp	12.22	C19 H22 N4 O8		81.11	434.143	0.72	(M+NH4)+
	Galβ1-4GlcNAcβ-Sp	12.22	C16 H28 N4 O11	Negative MS/MS D10 from http://www.functionalglycomics.org/static/consortium/resources/resourcecored2.shtml#dissacharides	74.59	452.1774	-1.96	M+
	Galβ1-3GlcNAcβ-Sp	12.22	C16 H28 N4 O11	D8 from http://www.functionalglycomics.org/static/consortium/resources/resourcecored2.shtml#dissacharides	74.59	452.1774	-1.96	M+
	Tyr Thr Phe	12.22	C22 H27 N3 O6		67.69	429.1876	2.37	(M+Na)+
	Phe Tyr Thr	12.22	C22 H27 N3 O6		67.69	429.1876	2.37	(M+Na)+
	Thr Tyr Phe	12.22	C22 H27 N3 O6		67.69	429.1876	2.37	(M+Na)+
	Thr Phe Tyr	12.22	C22 H27 N3 O6		67.69	429.1876	2.37	(M+Na)+

Identification Hit Table

Best Hit	Compound Name	RT	Formula	Notes	Match Score	Mass	Difference	Ion Species
✓	16-hydroxy-18-bromo-8E,17E19Z-tricosatrien-4,6-diynoic acid	12.22	C23 H31 Br O3		44.73	434.143	2.68	(M+NH4)+
	(9R,10S)-rel-(-)-9,10-bis(Acetyloxy)-9,10-dihydro-5-methoxy-8,8-dimethyl-2-phenyl-4H,8H-benzo[1,2-b:3,4-b']dipyran-4-one	12.22	C25 H24 O8		33.9	452.1535	-6.43	(M+NH4)+[-H2O]
	Epimedokoreanin A	12.22	C25 H24 O8		33.9	452.1535	-6.43	(M+NH4)+[-H2O]
	Heteroartonin A	12.22	C26 H28 O7		33.71	452.1773	6.16	M+
	Artonin S	12.22	C26 H28 O7		33.71	452.1773	6.16	M+
	Dorsmanin D	12.22	C26 H28 O7		33.71	452.1773	6.16	M+
	Broussoflavonol B	12.22	C26 H28 O7		33.71	452.1773	6.16	M+
	5,7-Dihydroxy-8-methoxy-3,4'-diprenyloxyflavone	12.22	C26 H28 O7		33.71	452.1773	6.16	M+
	Derrichalcone	12.22	C26 H28 O7		33.71	452.1773	6.16	M+
	Derriflavanone	12.22	C26 H28 O7		33.71	452.1773	6.16	M+

Identification Hit Table

Best Hit	Compound Name	RT	Formula	Notes	Match Score	Mass	Difference	Ion Species
✓	His His His	12.22	C18 H23 N9 O4		82.98	429.1877	-0.42	(M+Na)+
	His Asn Asn Ser	12.22	C17 H26 N8 O8		82.63	470.188	-0.68	M+[-H2O]
	His Asn Ser Asn	12.22	C17 H26 N8 O8		82.63	470.188	-0.68	M+[-H2O]
	Asn Asn His Ser	12.22	C17 H26 N8 O8		82.63	470.188	-0.68	M+[-H2O]
	Ser Asn Asn His	12.22	C17 H26 N8 O8		82.63	470.188	-0.68	M+[-H2O]
	Ser His Asn Asn	12.22	C17 H26 N8 O8		82.63	470.188	-0.68	M+[-H2O]
	Asn Ser Asn His	12.22	C17 H26 N8 O8		82.63	470.188	-0.68	M+[-H2O]
	Asn Ser His Asn	12.22	C17 H26 N8 O8		82.63	470.188	-0.68	M+[-H2O]
	Asn Asn Ser His	12.22	C17 H26 N8 O8		82.63	470.188	-0.68	M+[-H2O]
	Ser Asn His Asn	12.22	C17 H26 N8 O8		82.63	470.188	-0.68	M+[-H2O]

Compound Label	Name	*m/z*	RT	Algorithm	Mass
Cpd 27: Wilfordine; C43 H49 N O19; 12.256	**Wilfordine**	888.2748	12.256	Find by Molecular Feature	883.2875

Compound Chromatograms

Qualitative Compound Identification Report

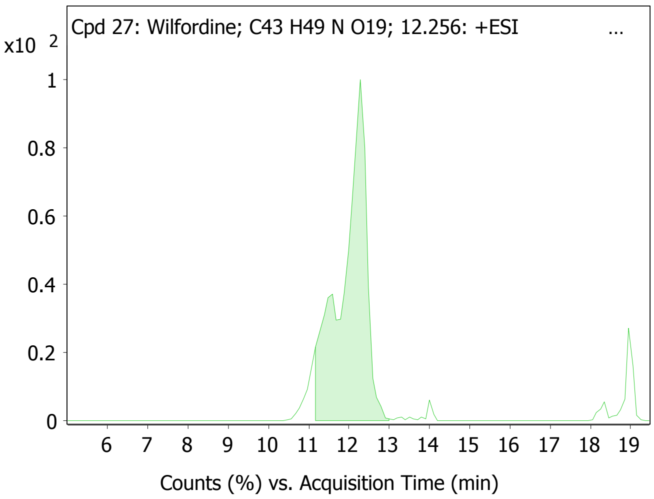

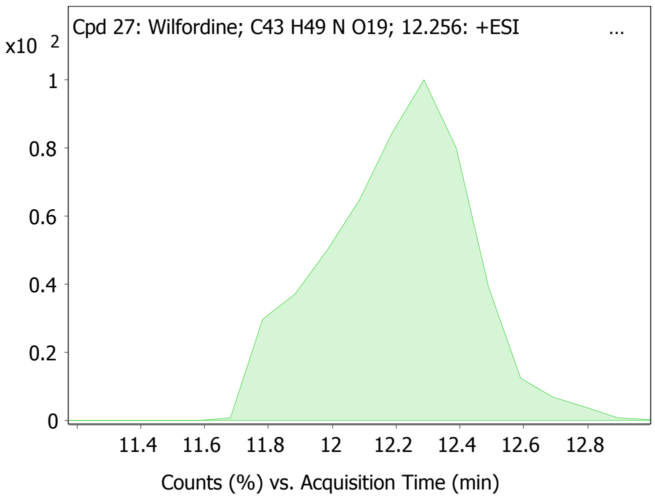

MFE MS Spectrum

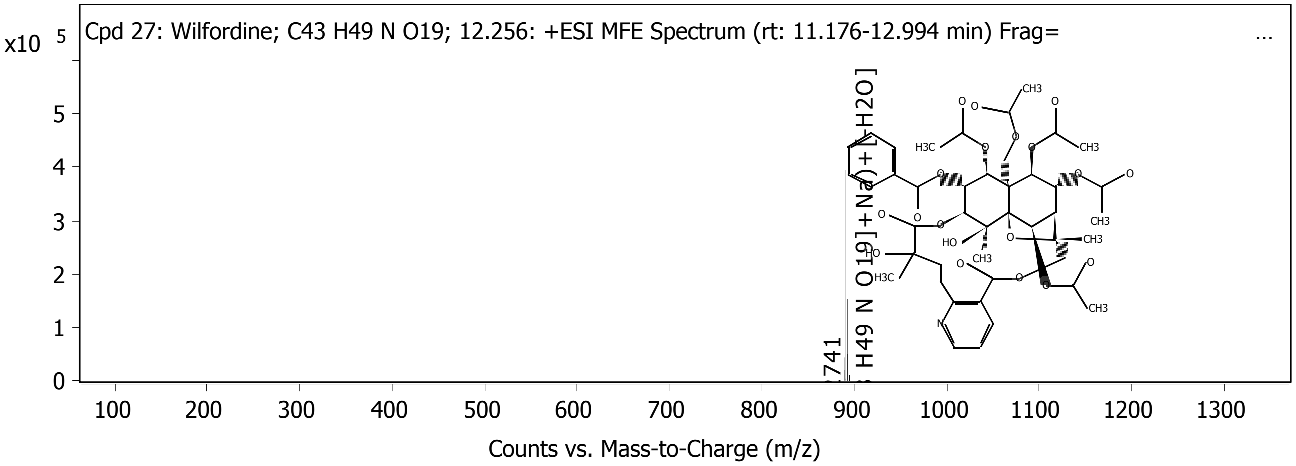

MFE MS Zoomed Spectrum

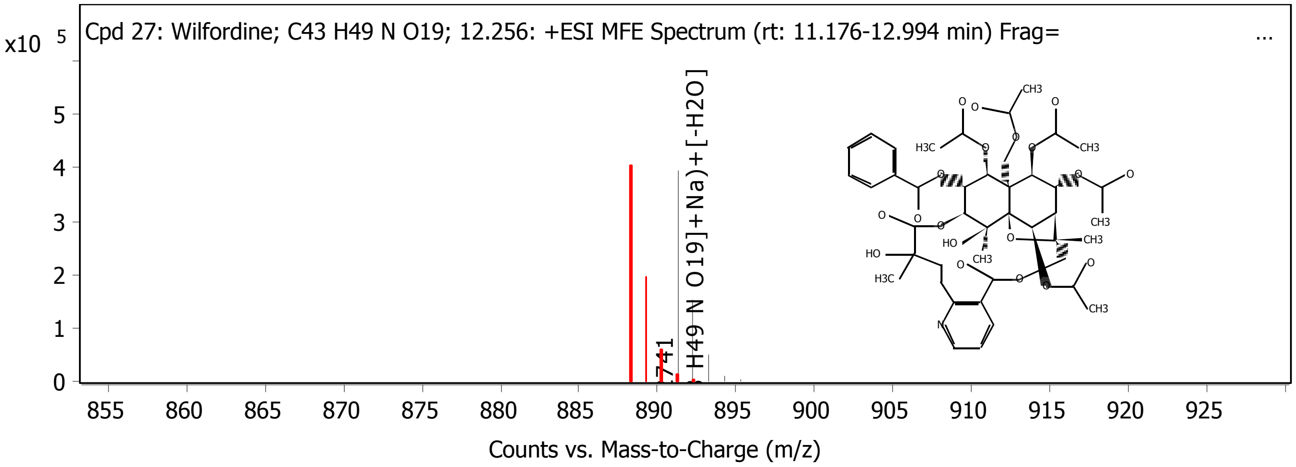

MS Spectrum

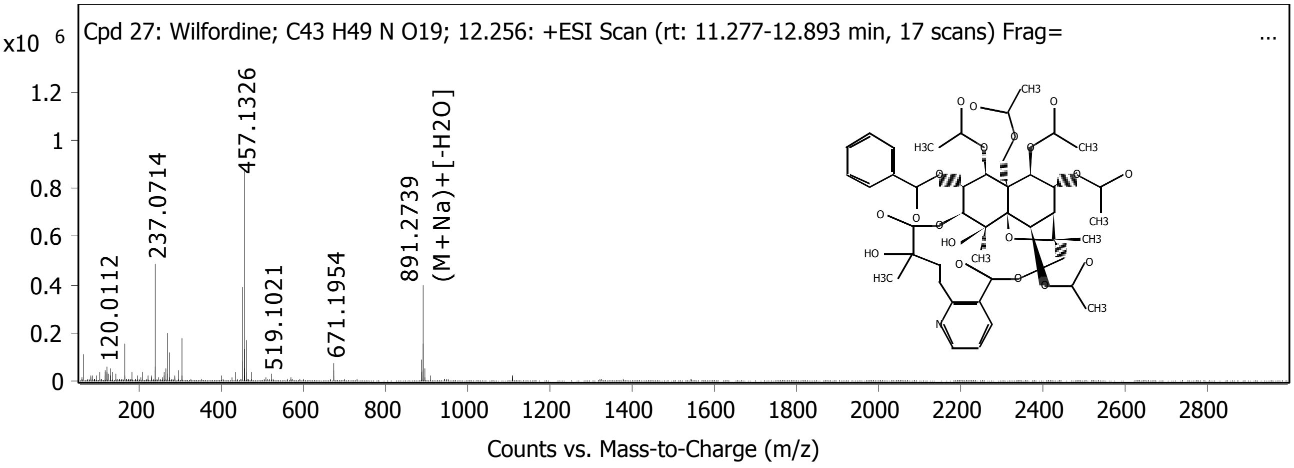

MS Zoomed Spectrum

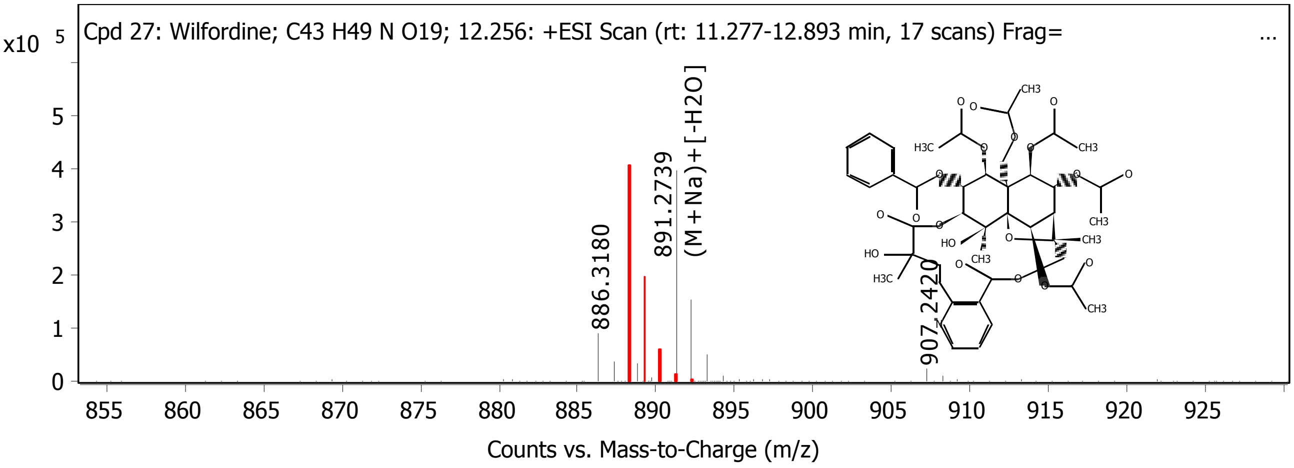

Identification Hit Table

Best Hit	Compound Name	RT	Formula	Notes	Match Score	Mass	Difference	Ion Species
✓	Wilfordine	12.256	C43 H49 N O19		52.38	883.2875	2.38	(M+Na)+[-H2O]
	Coenzyme F430	12.256	C42 H51 N6 Ni O13		33.42	905.2794	7.38	(M+H)+[-H2O]
	Quercetin 7-[xylosyl-(1->2)-rhamnosyl-(1->2)-rhamnosyl]-(1->6)-glucoside	12.256	C38 H48 O24		15.45	888.2432	10.36	(M+NH4)+[-H2O]
	Kaempferol 3-(2G-xylosylrutinoside)-7-glucoside	12.256	C38 H48 O24		15.45	888.2432	10.36	(M+NH4)+[-H2O]

Identification Hit Table

Best Hit	Compound Name	RT	Formula	Notes	Match Score	Mass	Difference	Ion Species
✓	Kaempferol 3-(2G-xylosylrutinoside)-7-glucoside	12.256	C38 H48 O24		15.45	888.2432	10.36	(M+NH4)+[-H2O]
	Quercetin 7-[xylosyl-(1->2)-rhamnosyl-(1->2)-rhamnosyl]-(1->6)-glucoside	12.256	C38 H48 O24		15.45	888.2432	10.36	(M+NH4)+[-H2O]

Identification Hit Table

Best Hit	Compound Name	RT	Formula	Notes	Match Score	Mass	Difference	Ion Species

Compound Label	Name	*m/z*	RT	Algorithm	Mass
Cpd 28: 3,4-Dihydroxyphenylpropanoate; C9 H10 O4; 12.258	**3,4-Dihydroxyphenylpropanoate**	165.0545	12.258	Find by Molecular Feature	182.0579

Compound Chromatograms

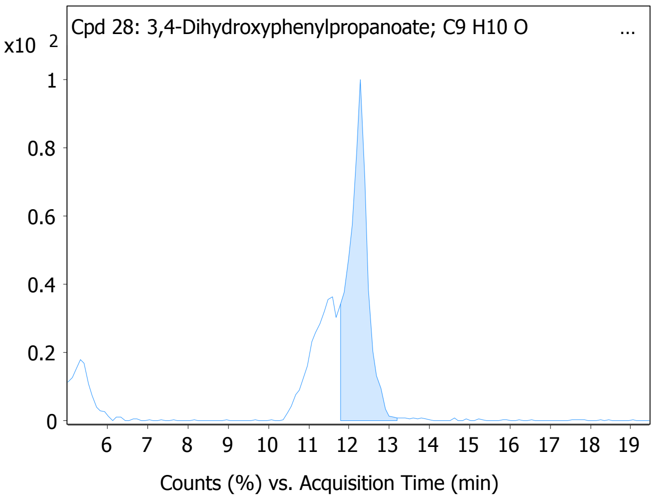

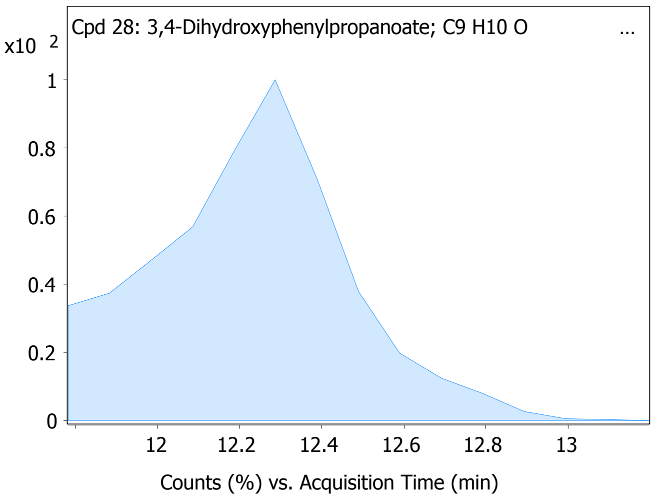

MFE MS Spectrum

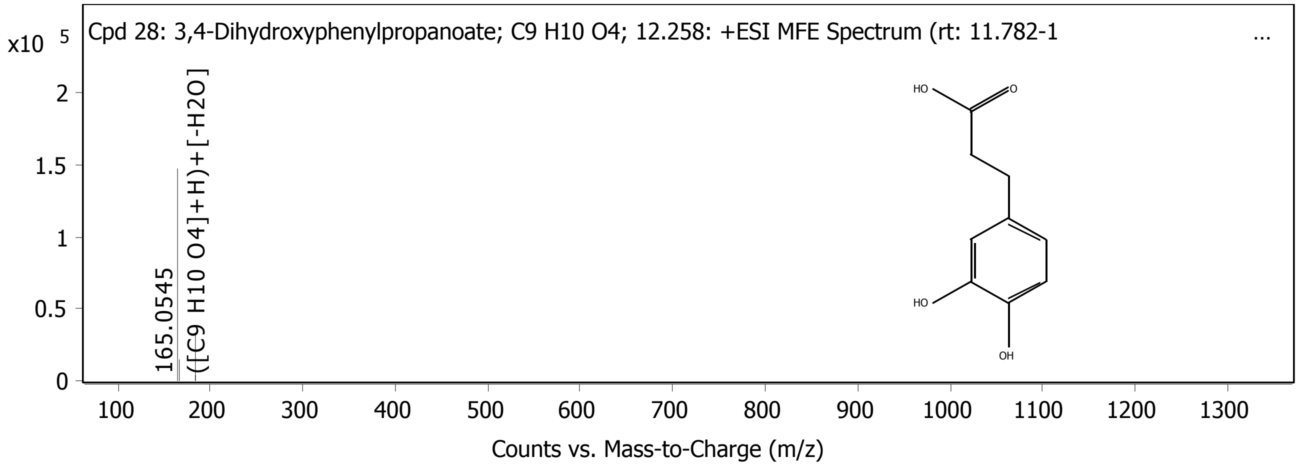

MFE MS Zoomed Spectrum

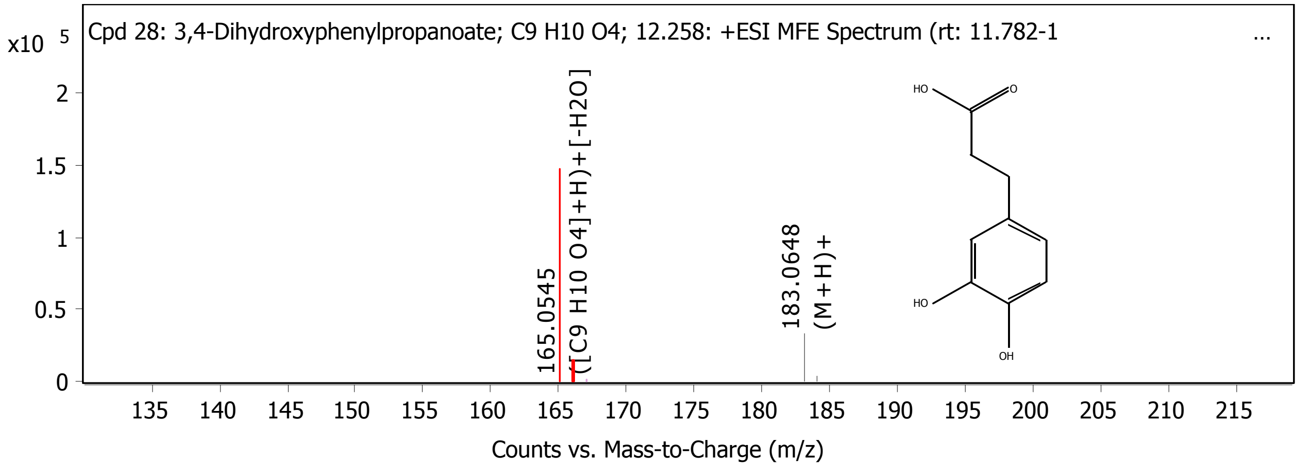

MS Spectrum

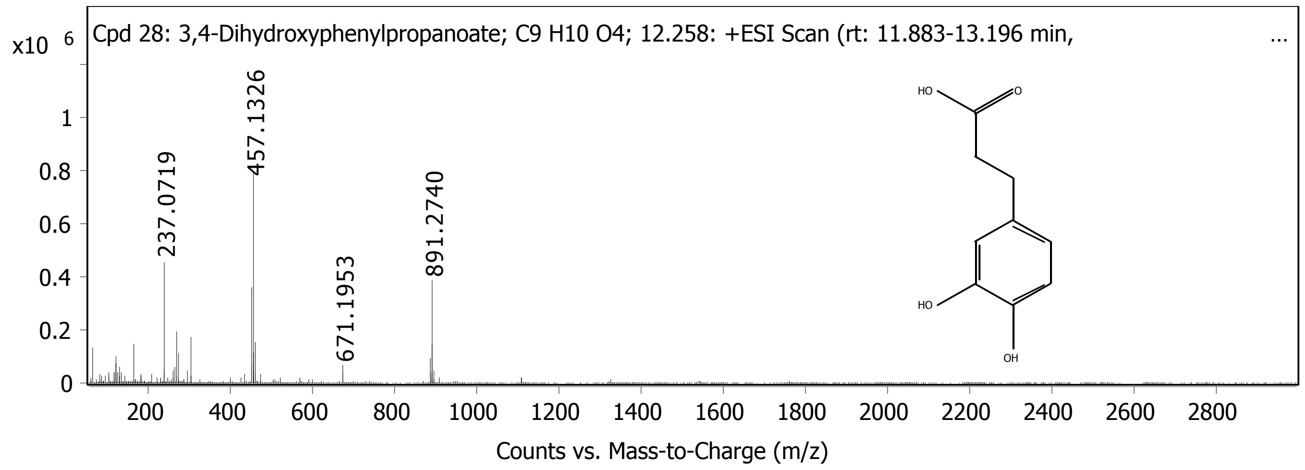

MS Zoomed Spectrum

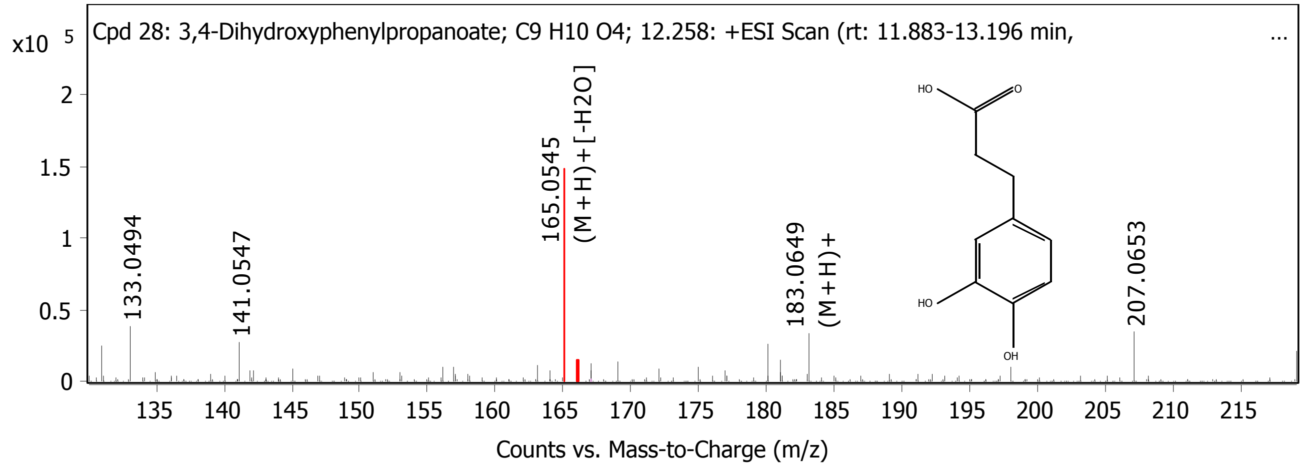

Identification Hit Table

Best Hit	Compound Name	RT	Formula	Notes	Match Score	Mass	Difference	Ion Species
✓	3,4-Dihydroxyphenylpropanoate	12.258	C9 H10 O4	Nugo Website Http://Nugo.org/metabolomics/13496 Urine	84.84	182.0579	-0.01	(M+H)+[-H2O]
	cis-3-(3-Carboxyethenyl)-3,5-cyclohexadiene-1,2-diol	12.258	C9 H10 O4		84.84	182.0579	-0.01	(M+H)+[-H2O]
	3-(2,3-Dihydroxyphenyl)propanoate	12.258	C9 H10 O4		84.84	182.0579	-0.01	(M+H)+[-H2O]
	Homovanillic acid	12.258	C9 H10 O4	3-Methoxy-4-hydroxyphenylacetic acid, Homovanillate Treatment of Parkinsons, Segawa's and Restless legs syndrome Endogenous metabolite of L-DOPA Dollery, Colin Therapeutic Drugs, 2nd Ed. 1999 p. L39	84.84	182.0579	-0.01	(M+H)+[-H2O]
	Veratric acid	12.258	C9 H10 O4	Antispasmodic (Treatment of IBS and other abdominal complications) Metabolite of Mebeverine Dollery, Colin Therapeutic Drugs, 2nd Ed. 1999 p. M15	84.84	182.0579	-0.01	(M+H)+[-H2O]
	Methyl vanillate	12.258	C9 H10 O4		84.84	182.0579	-0.01	(M+H)+[-H2O]
	Hydroxyphenyllactic acid	12.258	C9 H10 O4	Pubchem 9378	84.84	182.0579	-0.01	(M+H)+[-H2O]
	3-Methylorsellinic acid	12.258	C9 H10 O4	Aspergillus terreus	84.84	182.0579	-0.01	(M+H)+[-H2O]
	Everninic acid	12.258	C9 H10 O4	oak moss lichen	84.84	182.0579	-0.01	(M+H)+[-H2O]
	Flopropione	12.258	C9 H10 O4	synthetic	84.84	182.0579	-0.01	(M+H)+[-H2O]

Identification Hit Table

Best Hit	Compound Name	RT	Formula	Notes	Match Score	Mass	Difference	Ion Species
✓	9-hydroxy-7E-Nonene-3,5-diynoic acid	12.258	C9 H8 O3		84.84	164.0474	-0.01	(M+H)+
	9-hydroxy-7Z-Nonene-3,5-diynoic acid	12.258	C9 H8 O3		84.84	164.0474	-0.01	(M+H)+

Identification Hit Table

Best Hit	Compound Name	RT	Formula	Notes	Match Score	Mass	Difference	Ion Species

Compound Label	Name	*m/z*	RT	Algorithm	Mass
Cpd 29: H-8; C12 H15 N3 O2 S; 12.263	**H-8**	265.0919	12.263	Find by Molecular Feature	265.09

Qualitative Compound Identification Report

Compound Chromatograms

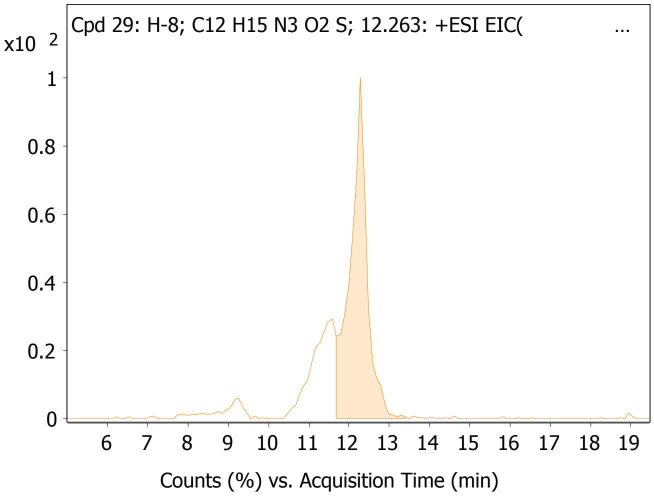

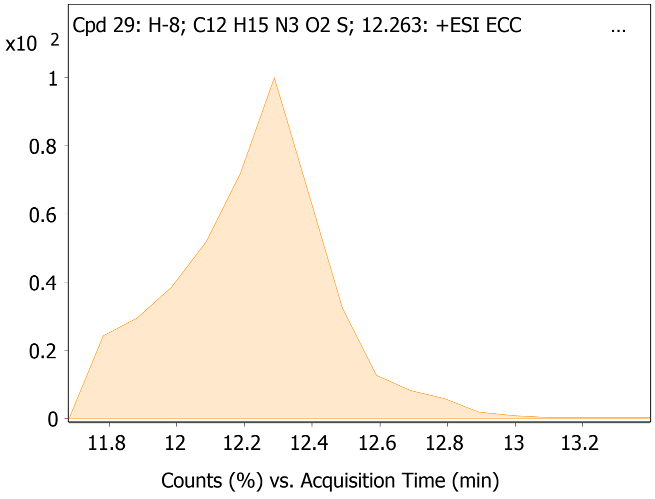

MFE MS Spectrum

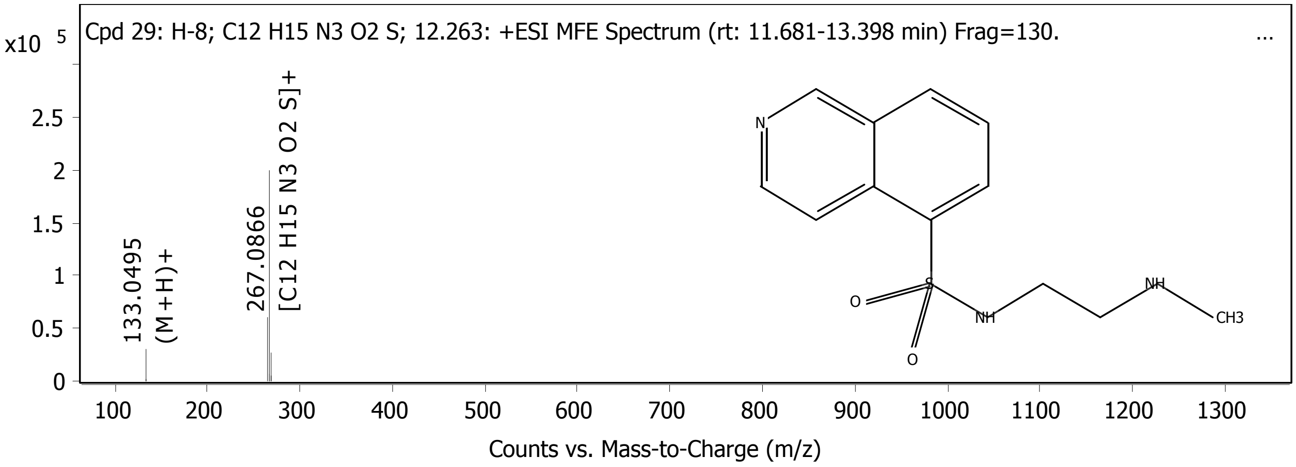

MFE MS Zoomed Spectrum

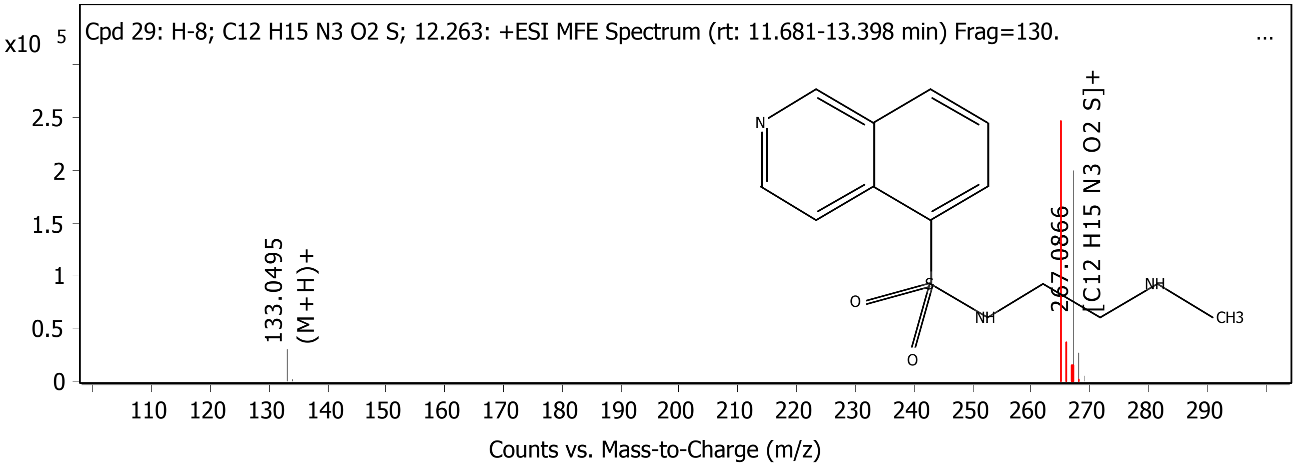

MS Spectrum

Qualitative Compound Identification Report

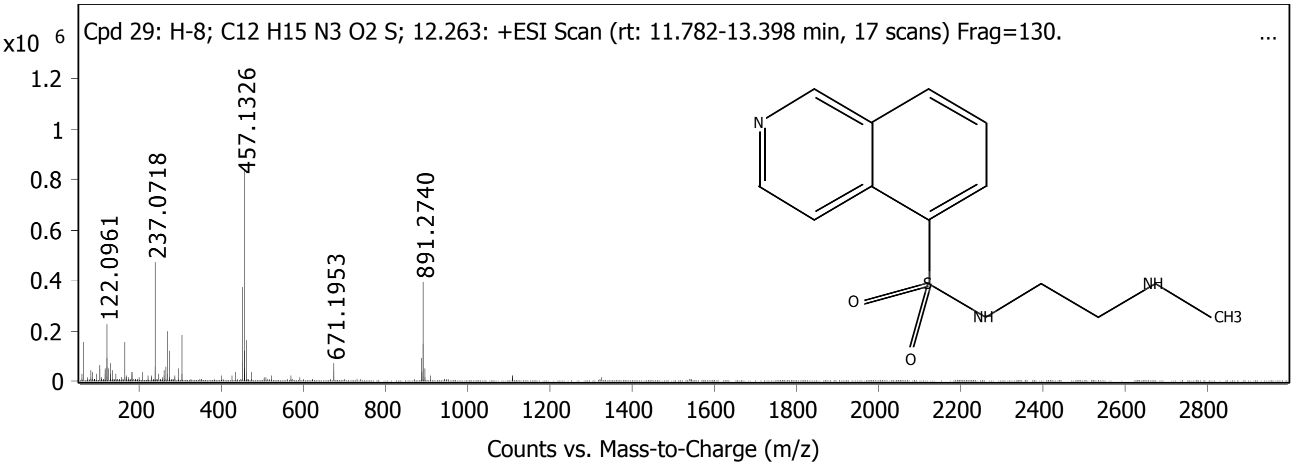

MS Zoomed Spectrum

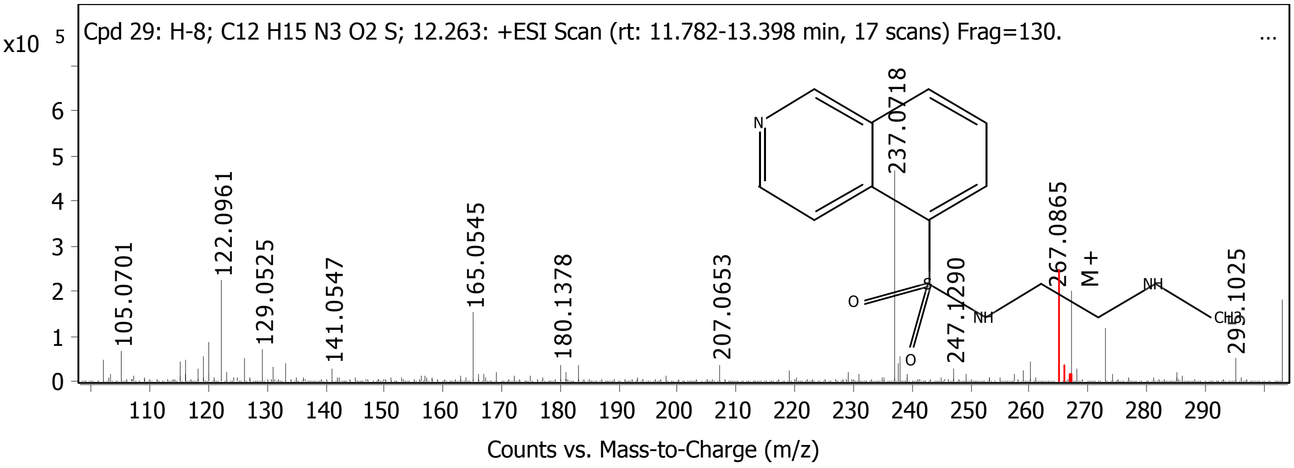

Identification Hit Table

Best Hit	Compound Name	RT	Formula	Notes	Match Score	Mass	Difference	Ion Species
✓	H-8	12.263	C12 H15 N3 O2 S		57.38	265.09	-1.5	M+
	Albendazole	12.263	C12 H15 N3 O2 S	antiparasitic Dollery, Colin Therapeutic Drugs, 2nd Ed. 1999 p. A51	57.38	265.09	-1.5	M+

Identification Hit Table

Best Hit	Compound Name	RT	Formula	Notes	Match Score	Mass	Difference	Ion Species

Identification Hit Table

Best Hit	Compound Name	RT	Formula	Notes	Match Score	Mass	Difference	Ion Species

Compound Label	Name	*m/z*	RT	Algorithm	Mass
Cpd 30: Met Gly Asn; C11 H20 N4 O5 S; 12.268	**Met Gly Asn**	303.1076	12.268	Find by Molecular Feature	320.1112

Compound Chromatograms

Agilent Technologies

Qualitative Compound Identification Report

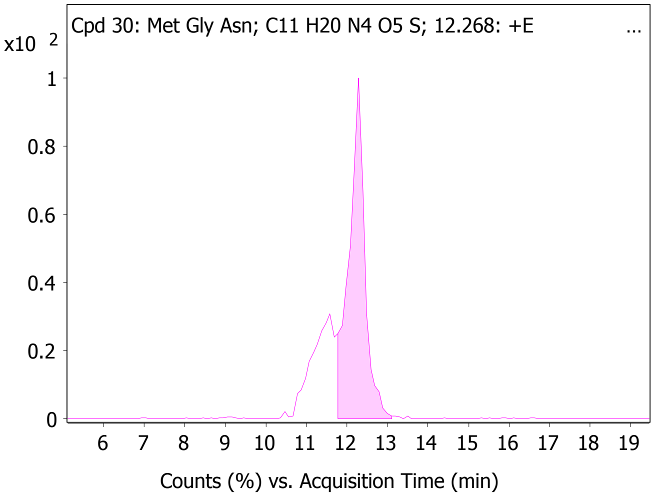

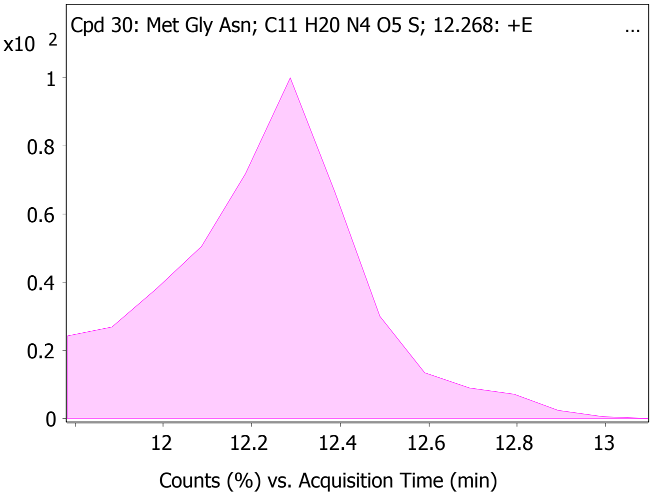

MFE MS Spectrum

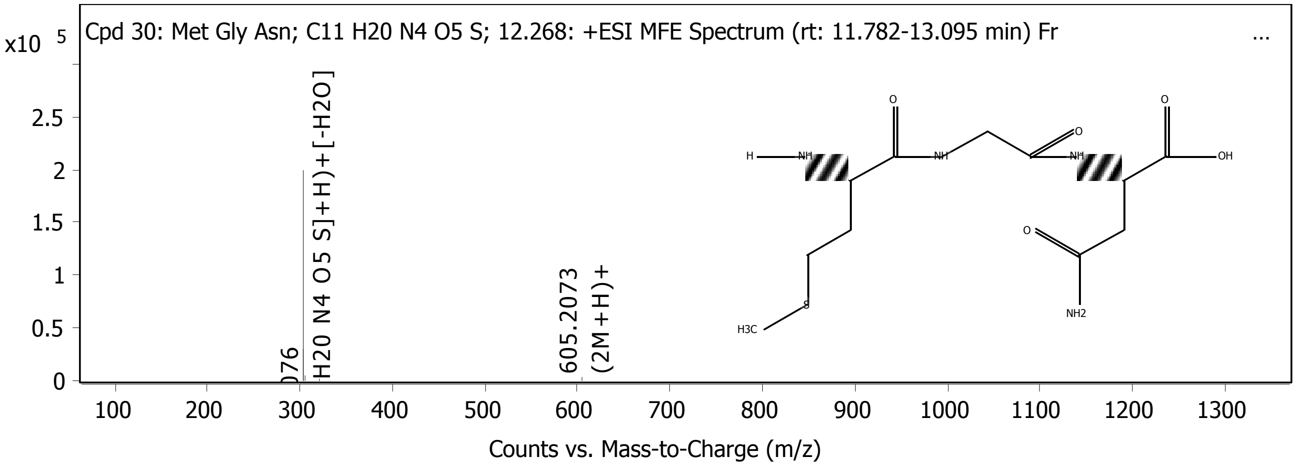

MFE MS Zoomed Spectrum

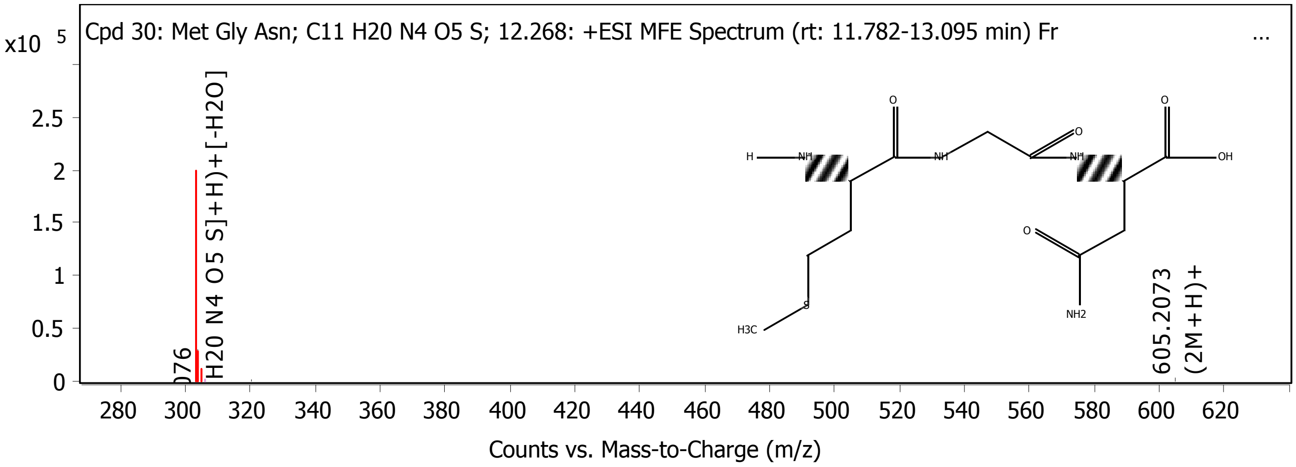

MS Spectrum

Qualitative Compound Identification Report

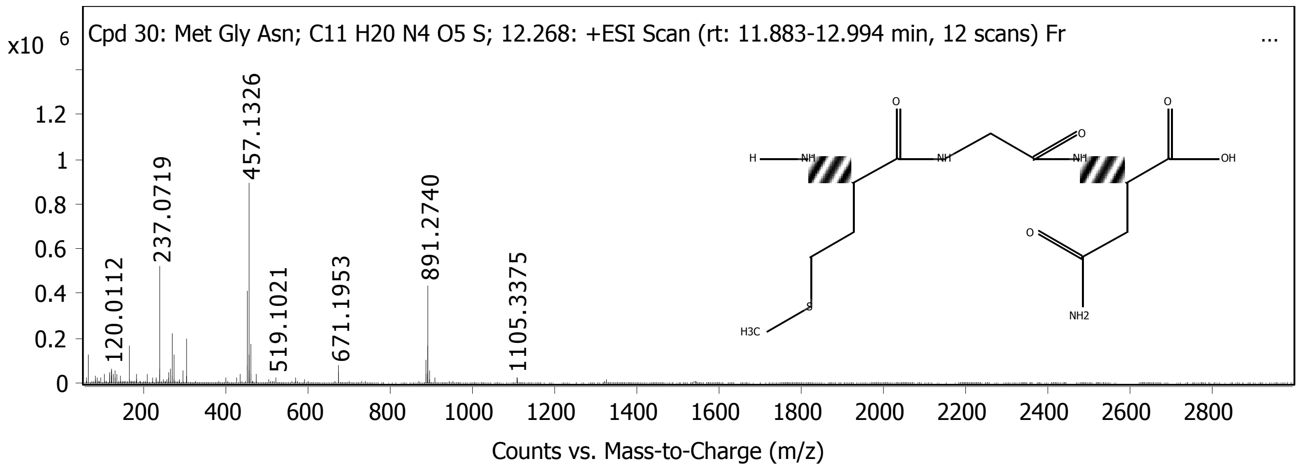

MS Zoomed Spectrum

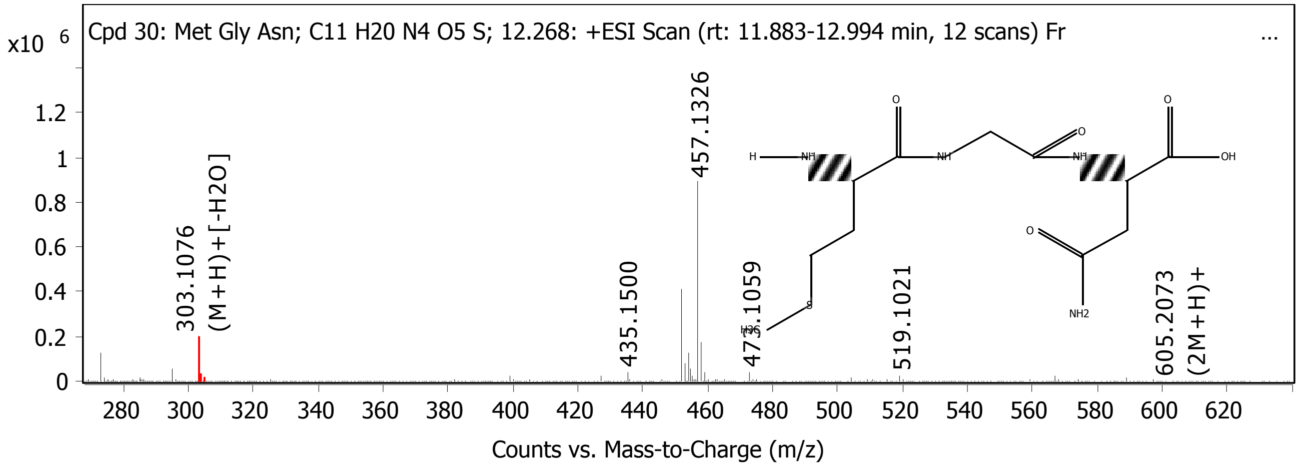

Identification Hit Table

Best Hit	Compound Name	RT	Formula	Notes	Match Score	Mass	Difference	Ion Species
✓	Met Gly Asn	12.268	C11 H20 N4 O5 S		58.93	320.1112	4.28	(M+H)+[-H2O]
	Ala Gln Cys	12.268	C11 H20 N4 O5 S		58.93	320.1112	4.28	(M+H)+[-H2O]
	Cys Gln Ala	12.268	C11 H20 N4 O5 S		58.93	320.1112	4.28	(M+H)+[-H2O]
	Asn Met Gly	12.268	C11 H20 N4 O5 S		58.93	320.1112	4.28	(M+H)+[-H2O]
	Gly Asn Met	12.268	C11 H20 N4 O5 S		58.93	320.1112	4.28	(M+H)+[-H2O]
	Gly Met Asn	12.268	C11 H20 N4 O5 S		58.93	320.1112	4.28	(M+H)+[-H2O]
	Ala Cys Gln	12.268	C11 H20 N4 O5 S		58.93	320.1112	4.28	(M+H)+[-H2O]
	Cys Ala Gln	12.268	C11 H20 N4 O5 S		58.93	320.1112	4.28	(M+H)+[-H2O]
	Met Asn Gly	12.268	C11 H20 N4 O5 S		58.93	320.1112	4.28	(M+H)+[-H2O]
	Gln Cys Ala	12.268	C11 H20 N4 O5 S		58.93	320.1112	4.28	(M+H)+[-H2O]

Identification Hit Table

Best Hit	Compound Name	RT	Formula	Notes	Match Score	Mass	Difference	Ion Species

Identification Hit Table

Best Hit	Compound Name	RT	Formula	Notes	Match Score	Mass	Difference	Ion Species
✓	Gly Cys Ala Ala	12.268	C11 H20 N4 O5 S		58.93	320.1112	4.28	(M+H)+[-H2O]
	Ala Gln Cys	12.268	C11 H20 N4 O5 S		58.93	320.1112	4.28	(M+H)+[-H2O]
	Gly Met Gly Gly	12.268	C11 H20 N4 O5 S		58.93	320.1112	4.28	(M+H)+[-H2O]
	Asn Gly Met	12.268	C11 H20 N4 O5 S		58.93	320.1112	4.28	(M+H)+[-H2O]
	Gln Cys Ala	12.268	C11 H20 N4 O5 S		58.93	320.1112	4.28	(M+H)+[-H2O]
	Met Asn Gly	12.268	C11 H20 N4 O5 S		58.93	320.1112	4.28	(M+H)+[-H2O]
	Cys Ala Gln	12.268	C11 H20 N4 O5 S		58.93	320.1112	4.28	(M+H)+[-H2O]
	Ala Cys Gln	12.268	C11 H20 N4 O5 S		58.93	320.1112	4.28	(M+H)+[-H2O]
	Gly Met Asn	12.268	C11 H20 N4 O5 S		58.93	320.1112	4.28	(M+H)+[-H2O]
	Gly Asn Met	12.268	C11 H20 N4 O5 S		58.93	320.1112	4.28	(M+H)+[-H2O]

Compound Label	Name	*m/z*	RT	Algorithm	Mass
Cpd 31: Furaneol 4-glucoside; C12 H18 O8; 12.268	**Furaneol 4-glucoside**	273.097	12.268	Find by Molecular Feature	290.1003

Compound Chromatograms

Qualitative Compound Identification Report

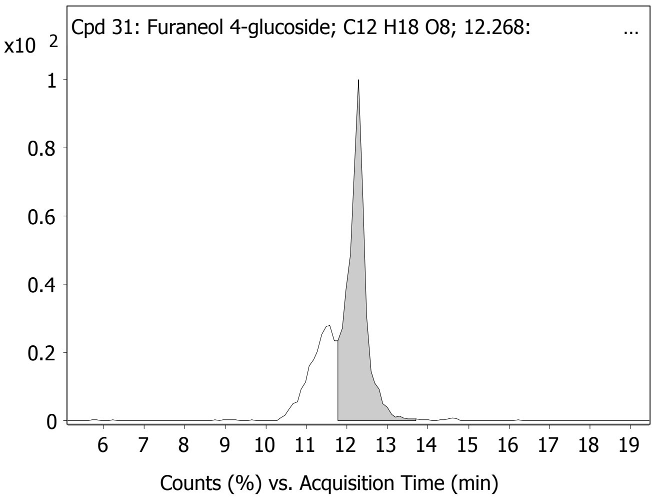

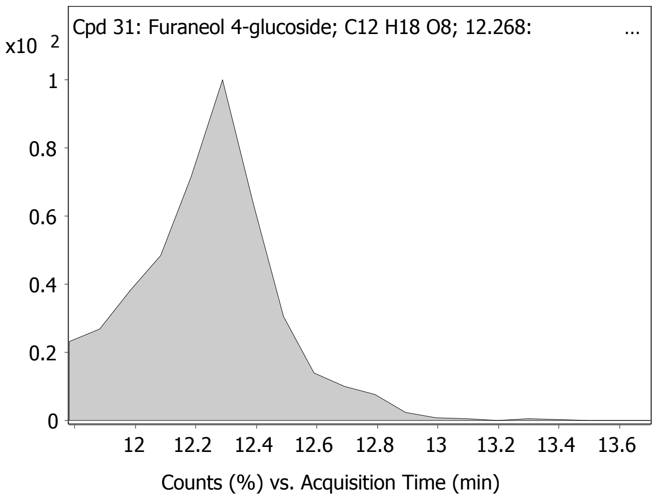

MFE MS Spectrum

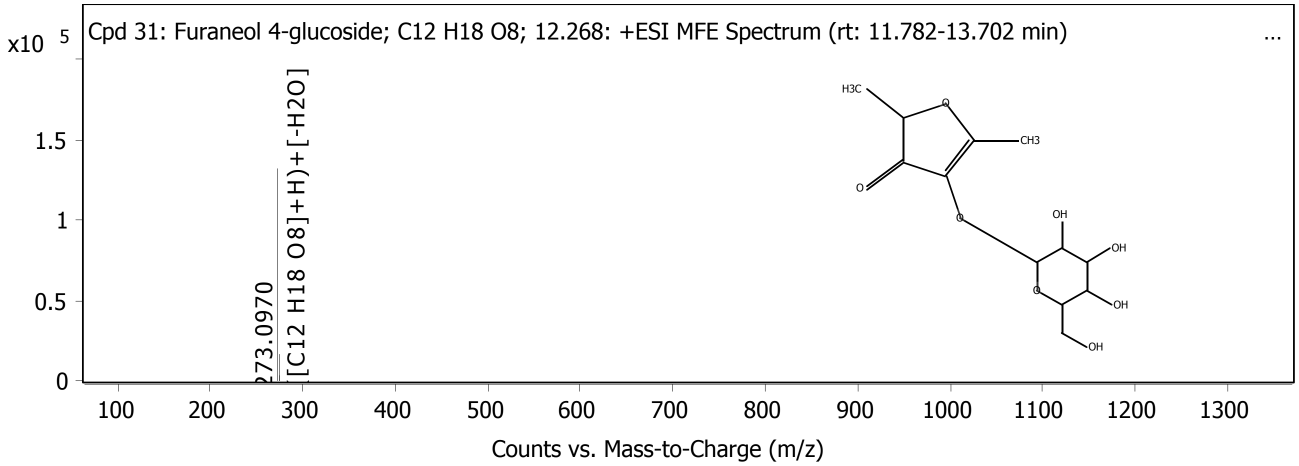

MFE MS Zoomed Spectrum

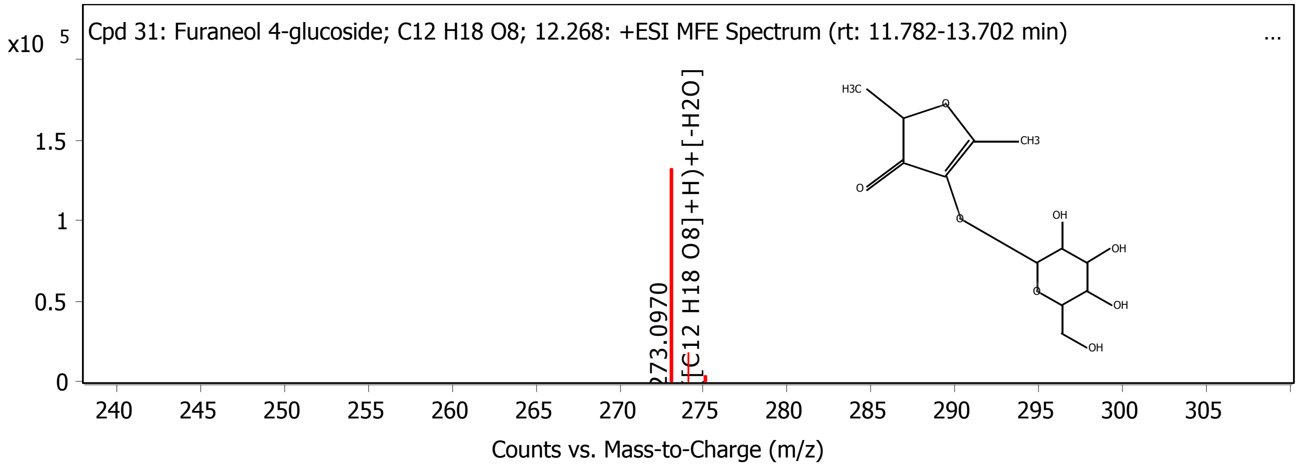

MS Spectrum

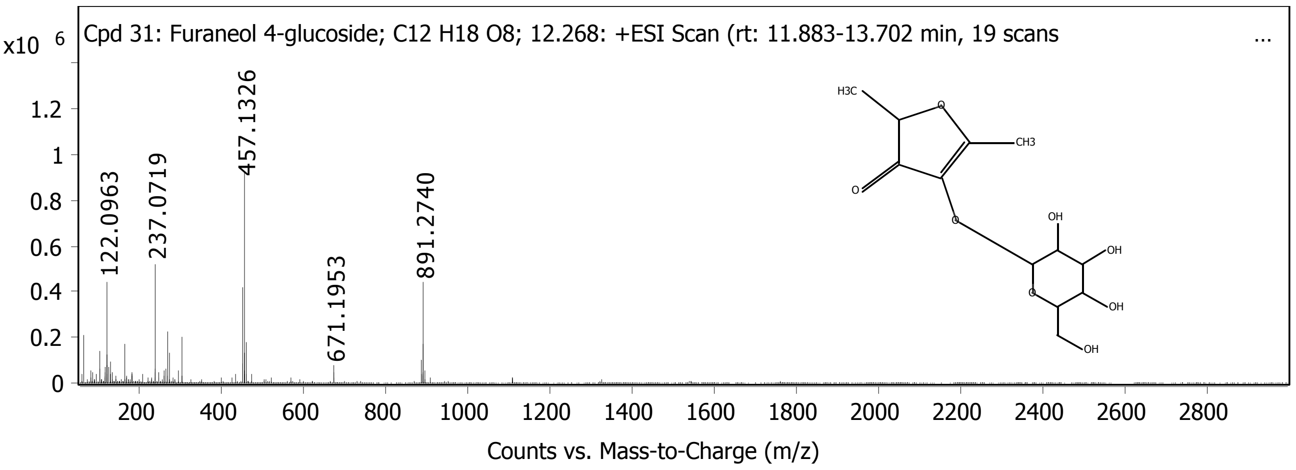

MS Zoomed Spectrum

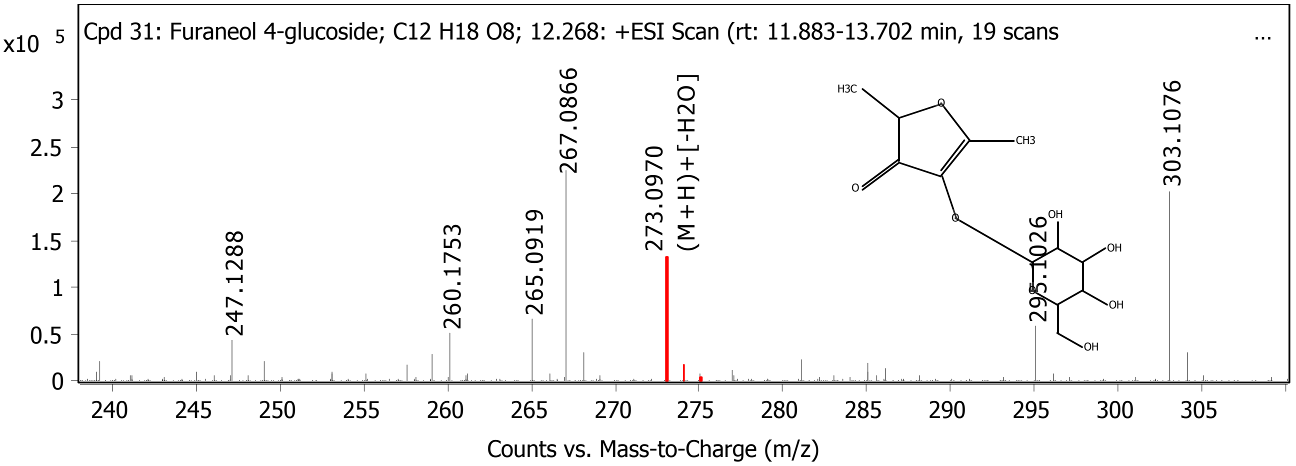

Identification Hit Table

Best Hit	Compound Name	RT	Formula	Notes	Match Score	Mass	Difference	Ion Species
✓	Furaneol 4-glucoside	12.268	C12 H18 O8		99.55	290.1003	-0.09	(M+H)+[-H2O]
	Osmundalin	12.268	C12 H18 O8		99.55	290.1003	-0.09	(M+H)+[-H2O]
	Arbutin	12.268	C12 H16 O7	Berginia crassifolia	99.54	272.0897	-0.09	(M+H)+
	Hydroxyprolyl-Histidine	12.268	C11 H16 N4 O4		92.88	268.1184	-1.24	(M+Na)+[-H2O]
	Dexrazoxane	12.268	C11 H16 N4 O4		92.88	268.1184	-1.24	(M+Na)+[-H2O]
	Histidinyl-Hydroxyproline	12.268	C11 H16 N4 O4		92.88	268.1184	-1.24	(M+Na)+[-H2O]
	Deoxycoformycin	12.268	C11 H16 N4 O4		92.88	268.1184	-1.24	(M+Na)+[-H2O]
	Isobutylglycine	12.268	C11 H16 N4 O4		92.88	268.1184	-1.24	(M+Na)+[-H2O]
	3-Deaza-2'-deoxyadenosine	12.268	C11 H14 N4 O3		92.57	250.1078	-1.24	(M+Na)+
	Thr Asp Gly	12.268	C10 H17 N3 O7		91.81	291.1082	-1.5	M+[-H2O]

Identification Hit Table

Best Hit	Compound Name	RT	Formula	Notes	Match Score	Mass	Difference	Ion Species

Identification Hit Table

Best Hit	Compound Name	RT	Formula	Notes	Match Score	Mass	Difference	Ion Species
✓	Glu Ser Gly	12.268	C10 H17 N3 O7		91.81	291.1082	-1.5	M+[-H2O]
	Gly Glu Ser	12.268	C10 H17 N3 O7		91.81	291.1082	-1.5	M+[-H2O]
	Asp Thr Gly	12.268	C10 H17 N3 O7		91.81	291.1082	-1.5	M+[-H2O]
	Asp Ser Ala	12.268	C10 H17 N3 O7		91.81	291.1082	-1.5	M+[-H2O]
	Gly Thr Asp	12.268	C10 H17 N3 O7		91.81	291.1082	-1.5	M+[-H2O]
	Gly Ser Glu	12.268	C10 H17 N3 O7		91.81	291.1082	-1.5	M+[-H2O]
	Ala Asp Ser	12.268	C10 H17 N3 O7		91.81	291.1082	-1.5	M+[-H2O]
	Thr Asp Gly	12.268	C10 H17 N3 O7		91.81	291.1082	-1.5	M+[-H2O]
	Ser Gly Glu	12.268	C10 H17 N3 O7		91.81	291.1082	-1.5	M+[-H2O]
	Asp Gly Thr	12.268	C10 H17 N3 O7		91.81	291.1082	-1.5	M+[-H2O]

Compound Label	Name	*m/z*	RT	Algorithm	Mass
Cpd 32: 3,4-Dimethylphenol; C8 H10 O; 13.446	**3,4-Dimethylphenol**	122.0965	13.446	Find by Molecular Feature	122.0733

Compound Chromatograms

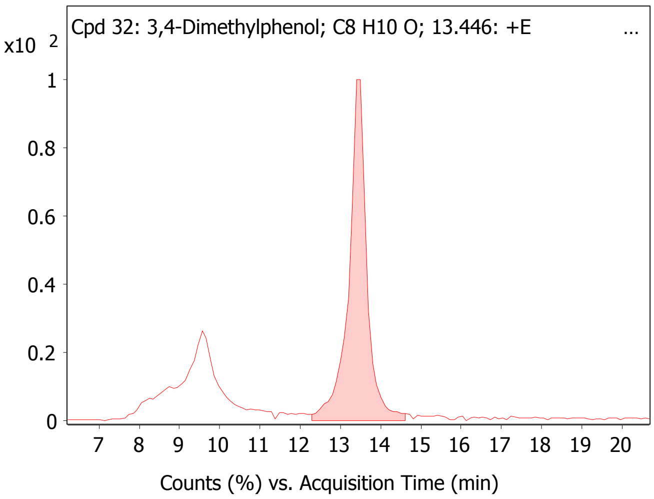

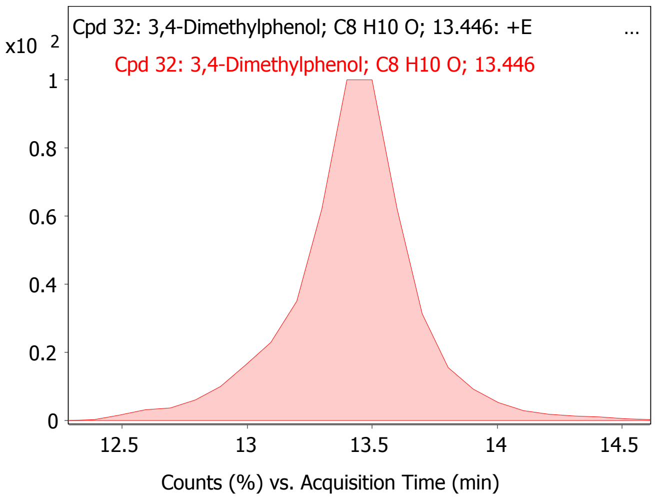

MFE MS Spectrum

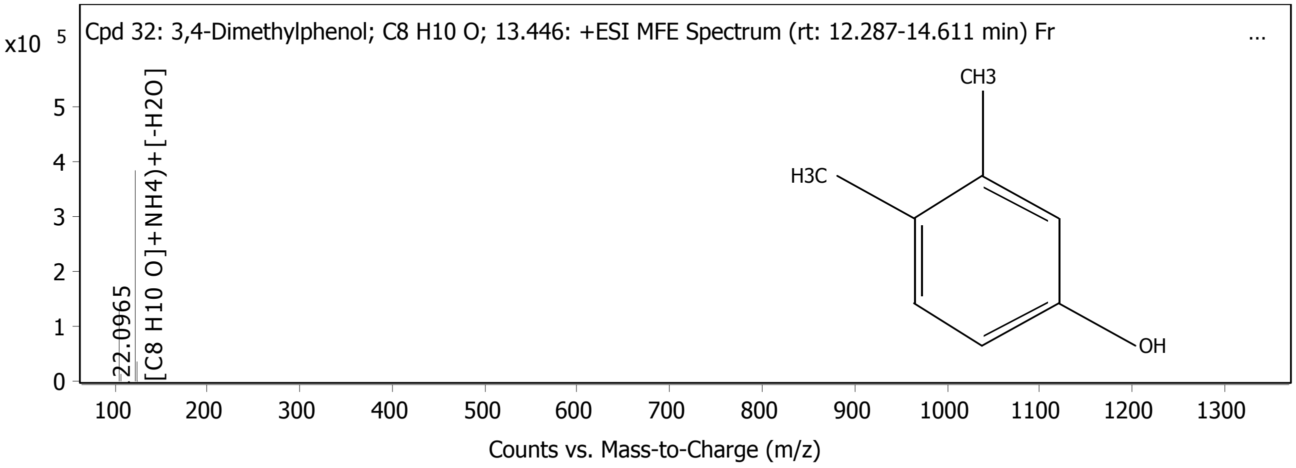

MFE MS Zoomed Spectrum

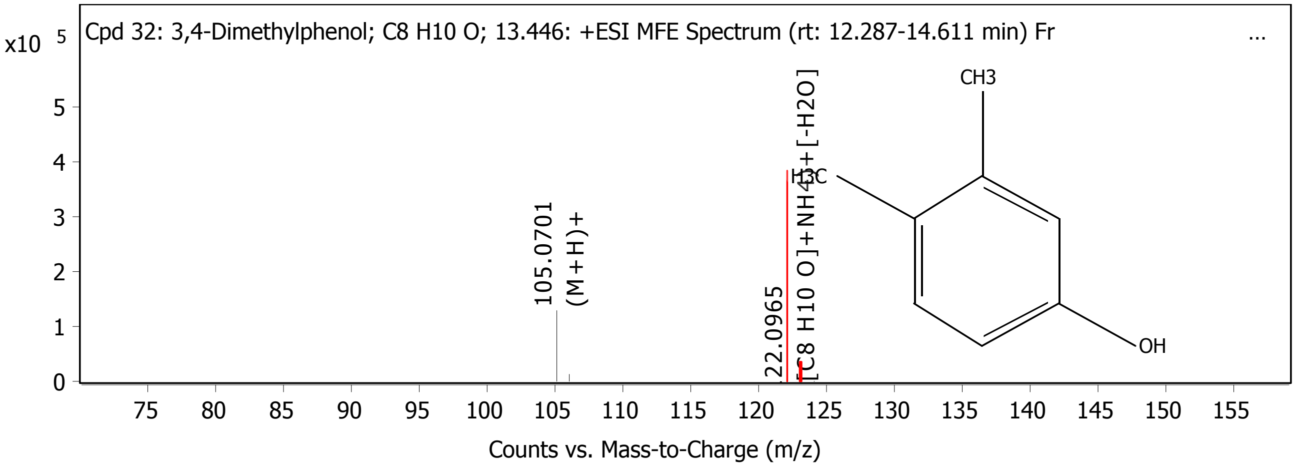

MS Spectrum

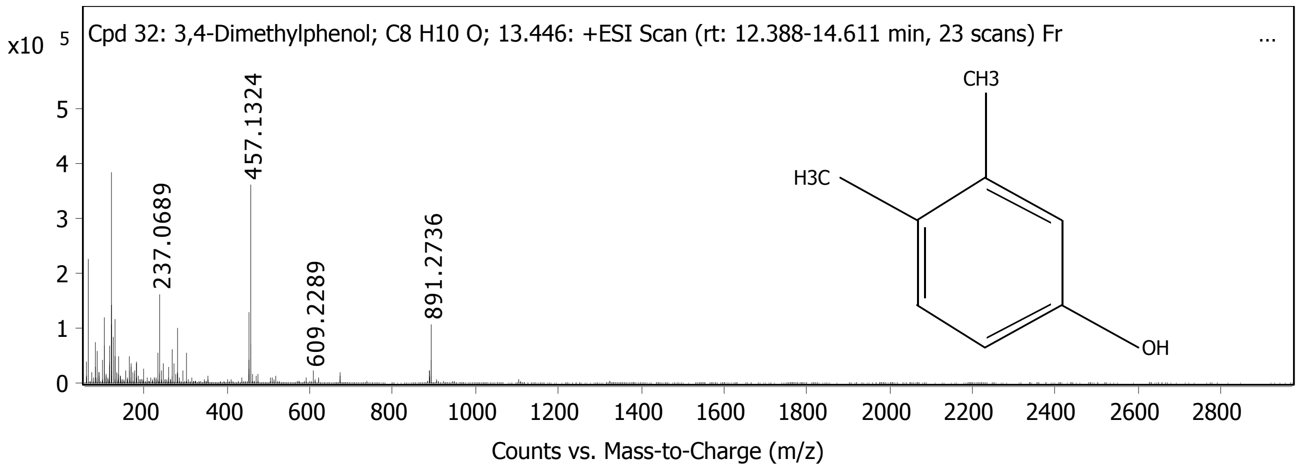

MS Zoomed Spectrum

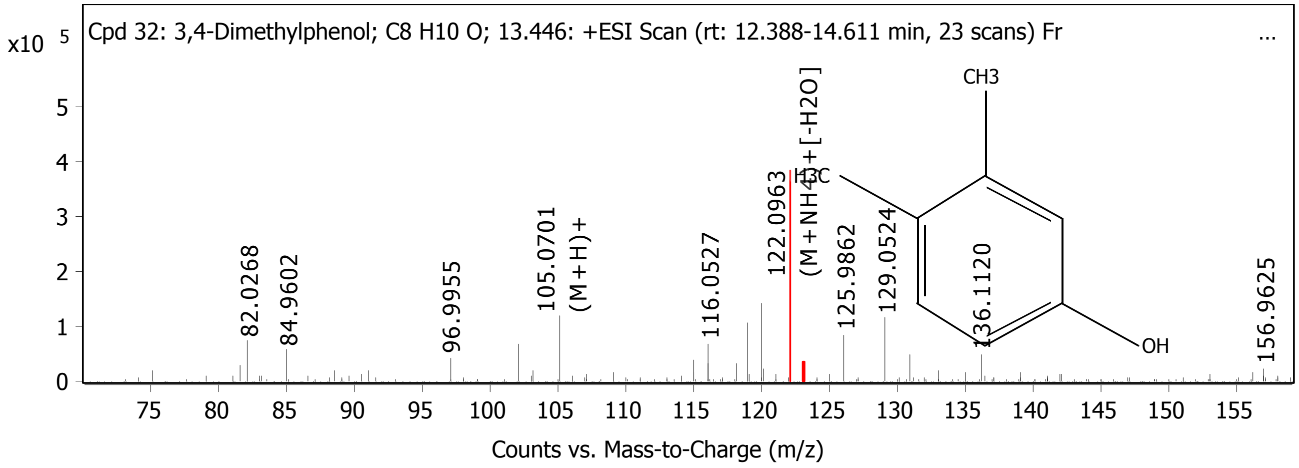

Library Spectrum

Difference Spectrum

MSMS Spectrum

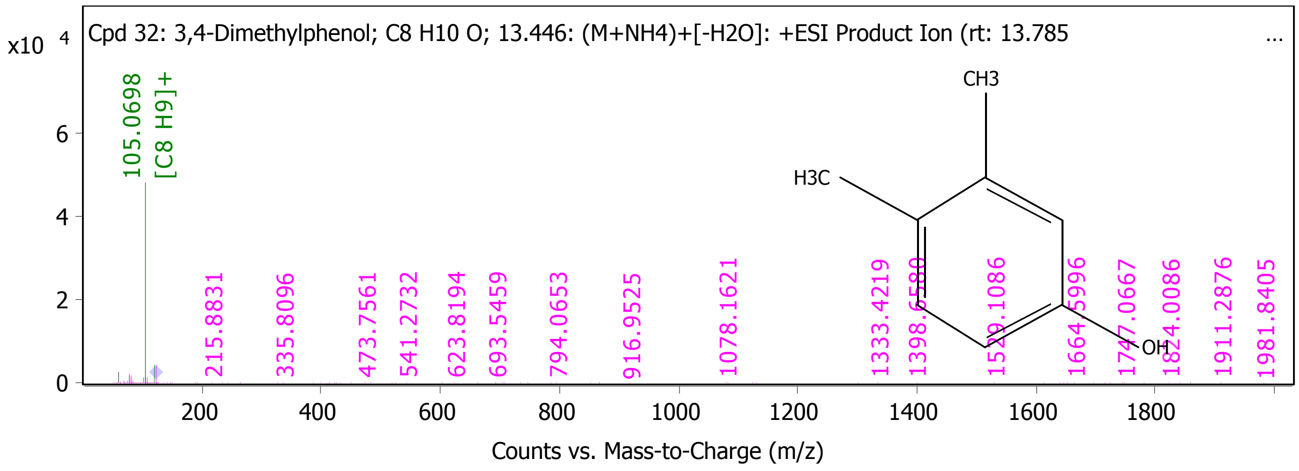

Identification Hit Table

Best Hit	Compound Name	RT	Formula	Notes	Match Score	Mass	Difference	Ion Species
✓	3,4-Dimethylphenol	13.446	C8 H10 O		99.86	122.0733	-0.09	(M+NH4)+[-H2O]
	2,6-Dimethylphenol	13.446	C8 H10 O		99.86	122.0733	-0.09	(M+NH4)+[-H2O]
	2-Phenylethanol	13.446	C8 H10 O		99.86	122.0733	-0.09	(M+NH4)+[-H2O]
	4-Methylbenzyl alcohol	13.446	C8 H10 O		99.86	122.0733	-0.09	(M+NH4)+[-H2O]
	1-Phenylethanol	13.446	C8 H10 O		99.86	122.0733	-0.09	(M+NH4)+[-H2O]
	2-Methylbenzyl alcohol	13.446	C8 H10 O		99.86	122.0733	-0.09	(M+NH4)+[-H2O]
	3-Methylbenzyl alcohol	13.446	C8 H10 O		99.86	122.0733	-0.09	(M+NH4)+[-H2O]
	(S)-1-Phenylethanol	13.446	C8 H10 O		99.86	122.0733	-0.09	(M+NH4)+[-H2O]

	4-Ethylphenol	13.446	C8 H10 O		99.86	122.0733	-0.09	(M+NH4)+[-H2O]
	2-Ethylphenol	13.446	C8 H10 O		99.86	122.0733	-0.09	(M+NH4)+[-H2O]

Identification Hit Table

Best Hit	Compound Name	RT	Formula	Notes	Match Score	Mass	Difference	Ion Species
✓	2,4,6-octatrienal	13.446	C8 H10 O		99.86	122.0733	-0.09	(M+NH4)+[-H2O]

Identification Hit Table

Best Hit	Compound Name	RT	Formula	Notes	Match Score	Mass	Difference	Ion Species

Compound Label	Name	*m/z*	RT	Algorithm	Mass
Cpd 33: Tyrosyl-Proline; C14 H18 N2 O4; 14.673	**Tyrosyl-Proline**	279.134	14.673	Find by Molecular Feature	278.1267

Compound Chromatograms

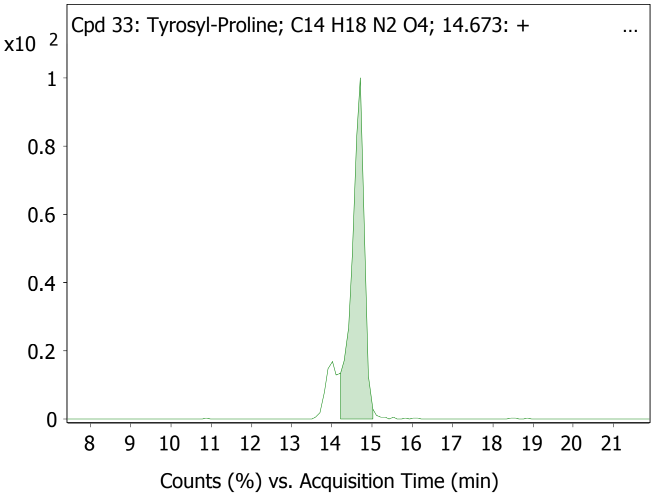

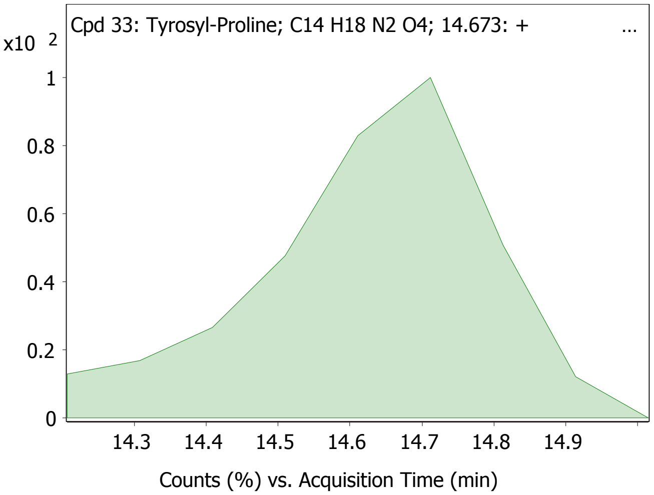

MFE MS Spectrum

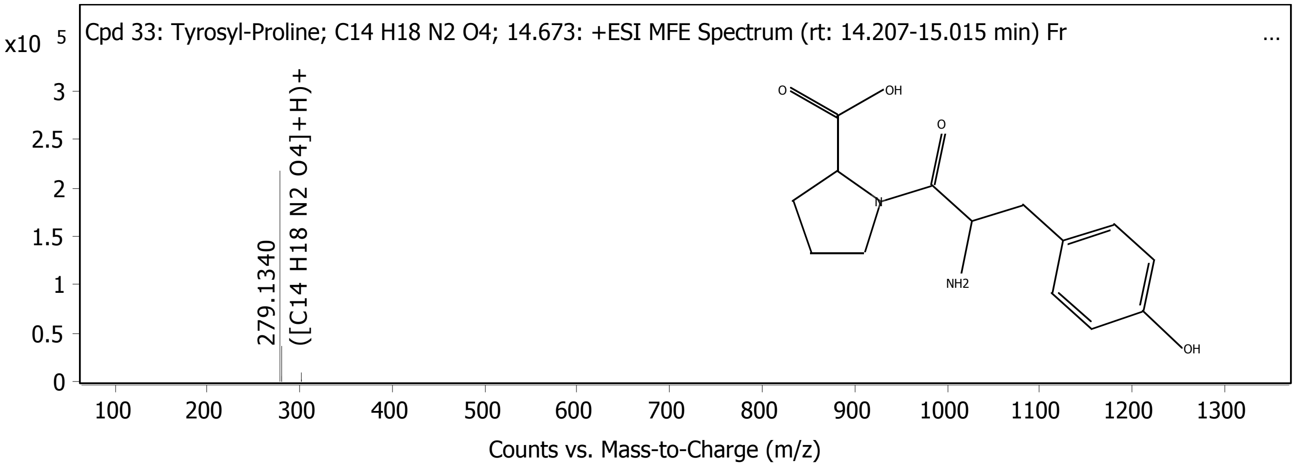

MFE MS Zoomed Spectrum

Qualitative Compound Identification Report

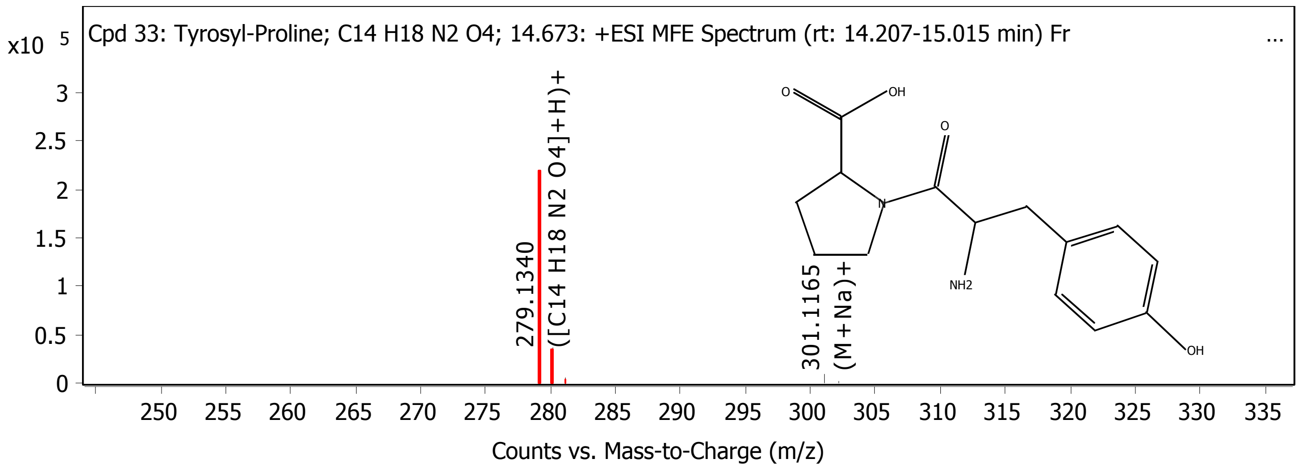

MS Spectrum

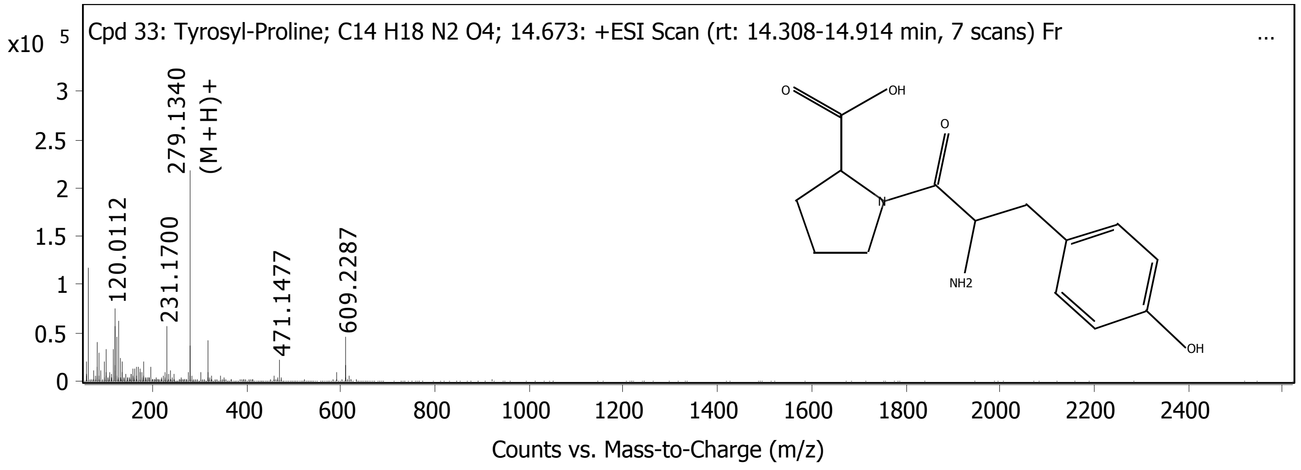

MS Zoomed Spectrum

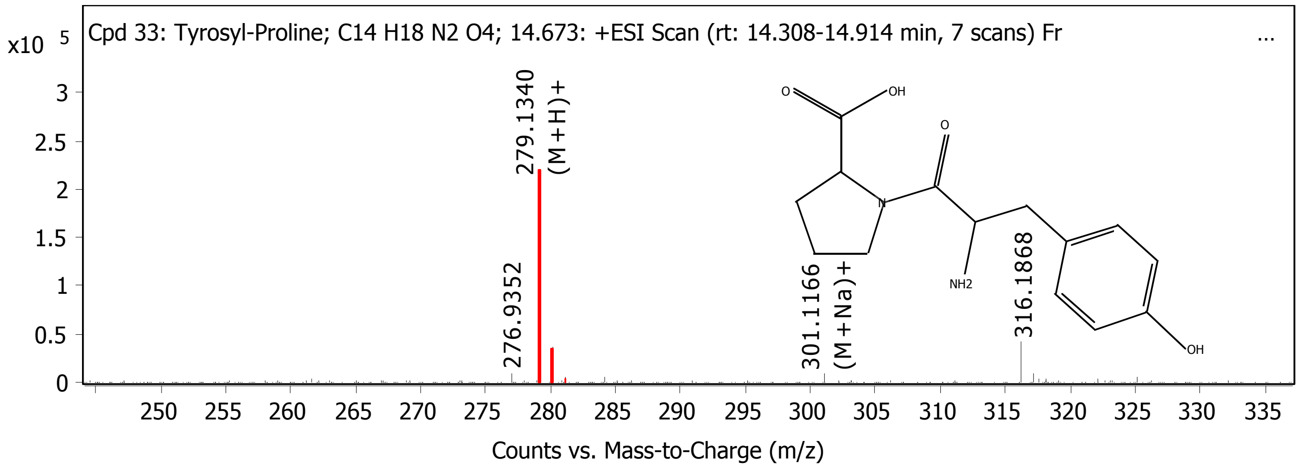

Identification Hit Table

Best Hit	Compound Name	RT	Formula	Notes	Match Score	Mass	Difference	Ion Species
✓	Tyrosyl-Proline	14.673	C14 H18 N2 O4		99.89	278.1267	-0.05	(M+H)+
	Prolyl-Tyrosine	14.673	C14 H18 N2 O4		99.89	278.1267	-0.05	(M+H)+
	Oxadixyl	14.673	C14 H18 N2 O4		99.89	278.1267	-0.05	(M+H)+
	Carboxy-PTIO	14.673	C14 H18 N2 O4		99.89	278.1267	-0.05	(M+H)+
	L-phenylalanyl-L-hydroxyproline	14.673	C14 H18 N2 O4		99.89	278.1267	-0.05	(M+H)+
	N1-(alpha-D-ribosyl)-5,6-dimethyl-benzimidazole	14.673	C14 H18 N2 O4		99.89	278.1267	-0.05	(M+H)+
	Tyr Pro	14.673	C14 H18 N2 O4		99.89	278.1267	-0.05	(M+H)+
	Pro Tyr	14.673	C14 H18 N2 O4		99.89	278.1267	-0.05	(M+H)+
	Pantetheine	14.673	C11 H22 N2 O4 S		64.82	278.1269	3.14	(M+H)+
	exo-Dehydrochalepin	14.673	C19 H20 O3		62.19	296.1372	4.02	(M+H)+[-H2O]

Qualitative Compound Identification Report

Identification Hit Table

Best Hit	Compound Name	RT	Formula	Notes	Match Score	Mass	Difference	Ion Species
✓	4-Prenylresveratrol	14.673	C19 H20 O3		62.19	296.1372	4.02	(M+H)+[-H2O]

Identification Hit Table

Best Hit	Compound Name	RT	Formula	Notes	Match Score	Mass	Difference	Ion Species
✓	Tyr Pro	14.673	C14 H18 N2 O4		99.89	278.1267	-0.05	(M+H)+
	Pro Tyr	14.673	C14 H18 N2 O4		99.89	278.1267	-0.05	(M+H)+

Compound Label	Name	*m/z*	RT	Algorithm	Mass
Cpd 34: Tetrahydropteroyltri-L-glutamate; C24 H34 N8 O12; 15.311	**Tetrahydropteroyltri-L-glutamate**	609.2292	15.311	Find by Molecular Feature	626.2327

Compound Chromatograms

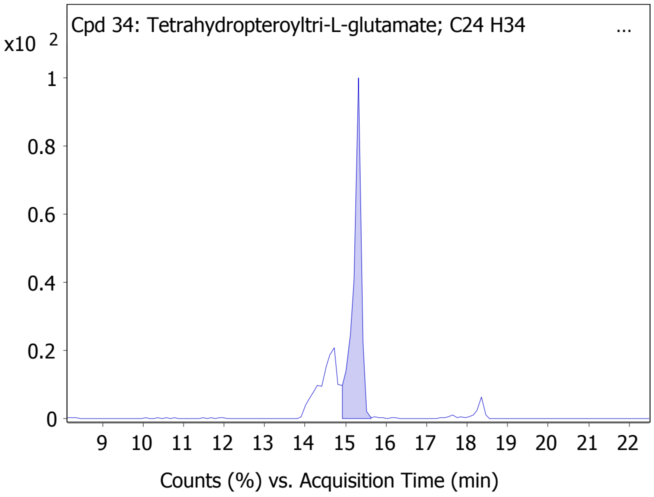

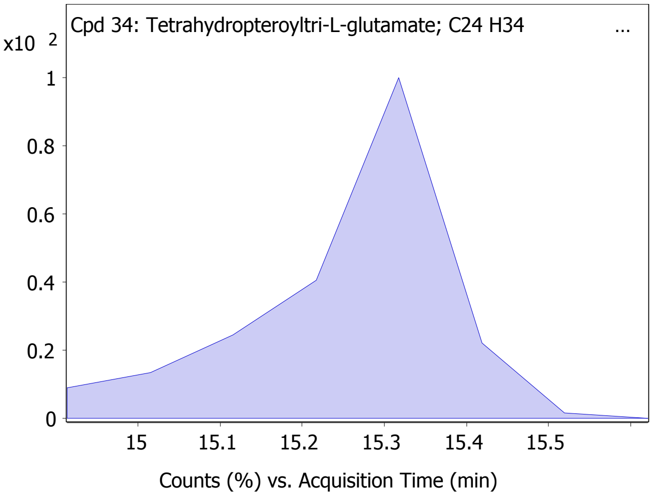

MFE MS Spectrum

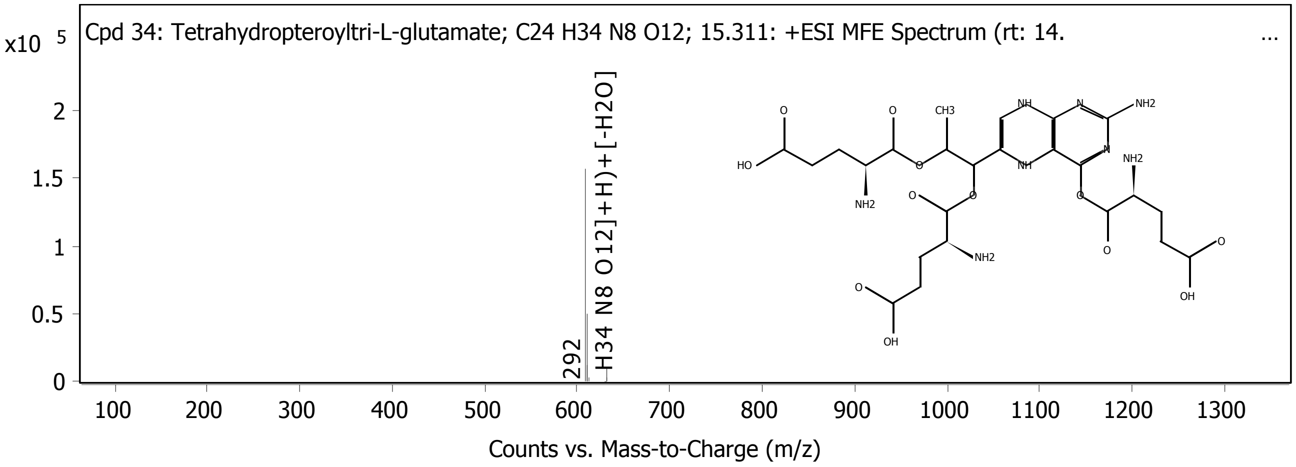

MFE MS Zoomed Spectrum

Qualitative Compound Identification Report

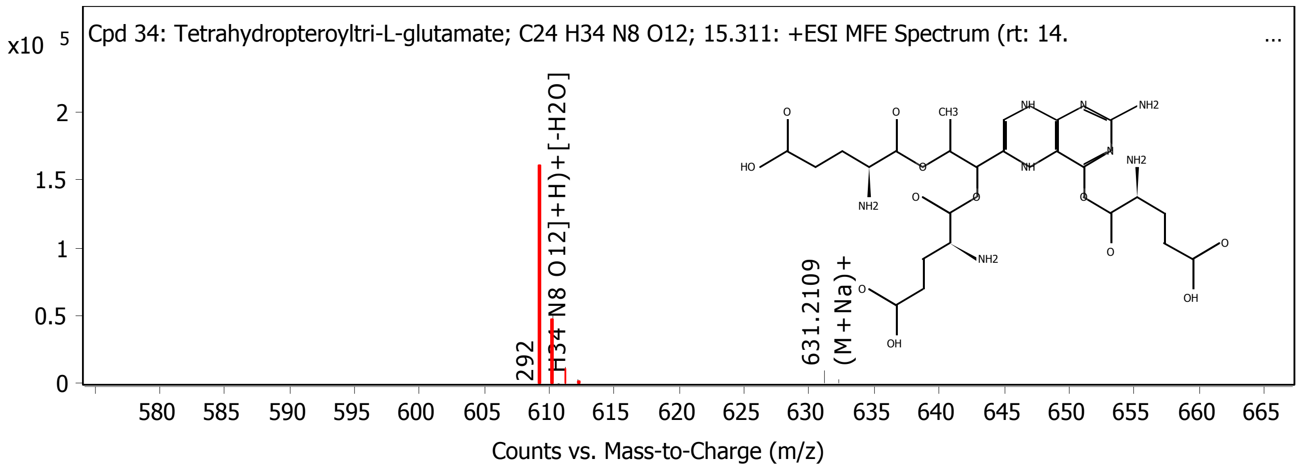

MS Spectrum

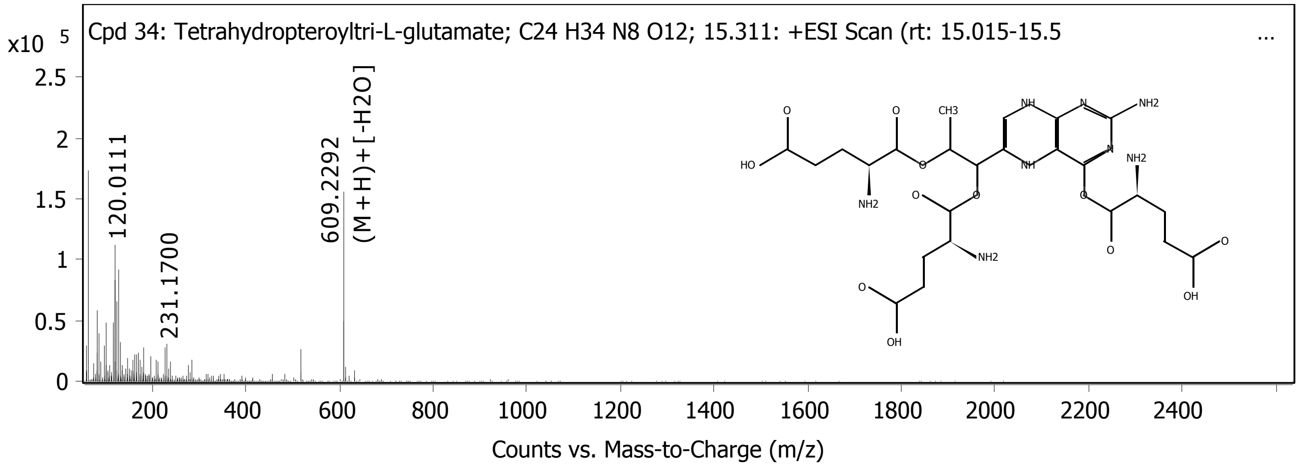

MS Zoomed Spectrum

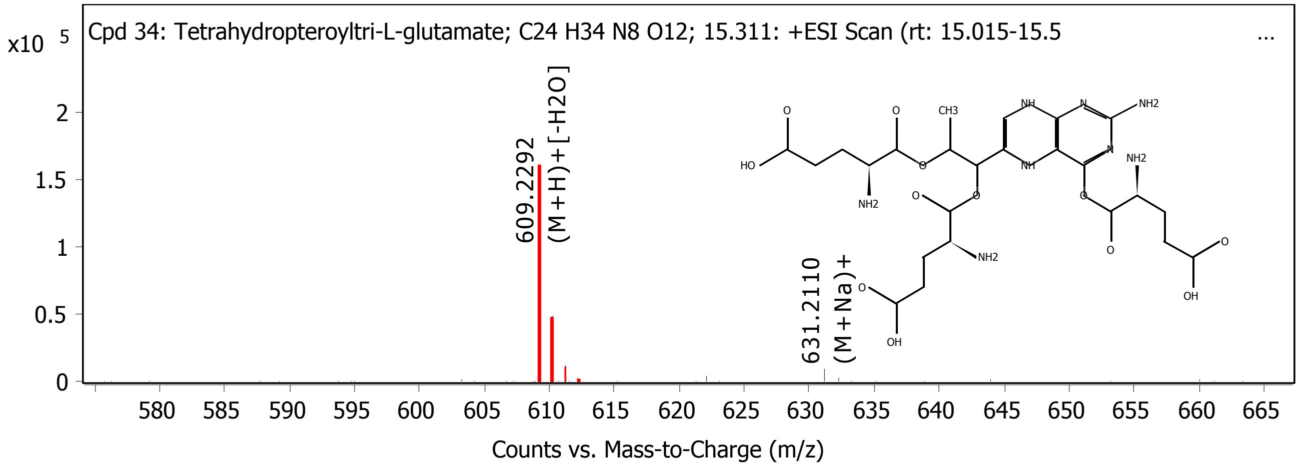

Identification Hit Table

Best Hit	Compound Name	RT	Formula	Notes	Match Score	Mass	Difference	Ion Species
✓	Tetrahydropteroyltri-L-glutamate	15.311	C24 H34 N8 O12	Endogenous Metabolite http://dbk.ch.umist.ac.uk/ExactMasses.htm	86.03	626.2327	-3.07	(M+H)+[-H2O]

Identification Hit Table

Best Hit	Compound Name	RT	Formula	Notes	Match Score	Mass	Difference	Ion Species

Identification Hit Table

Best Hit	Compound Name	RT	Formula	Notes	Match Score	Mass	Difference	Ion Species

Qualitative Compound Identification Report

Compound Label	Name	*m/z*	RT	Algorithm	Mass
Cpd 35: Methyl 1-methoxy-1H-indole-3-carboxylate; C11 H11 N O3; 17.013	**Methyl 1-methoxy-1H-indole-3-carboxylate**	205.0971	17.013	Find by Molecular Feature	205.0738

Compound Chromatograms

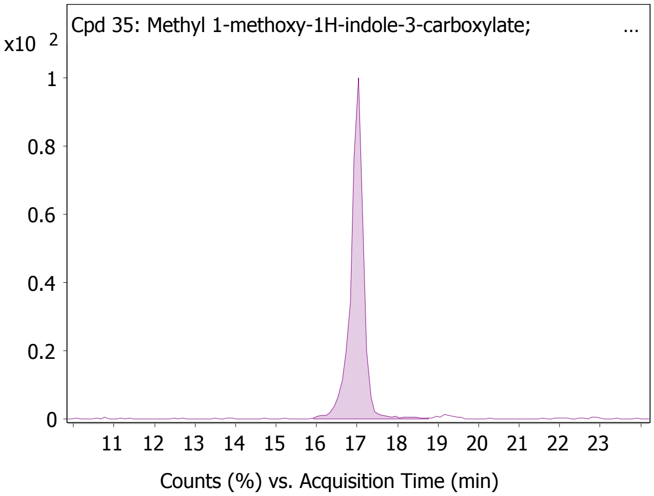

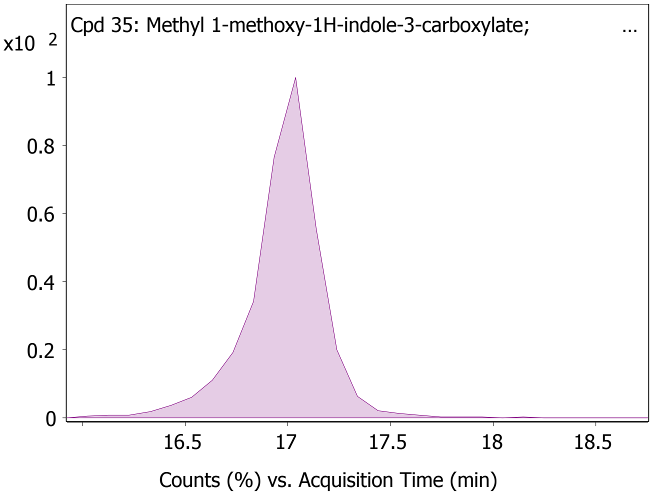

MFE MS Spectrum

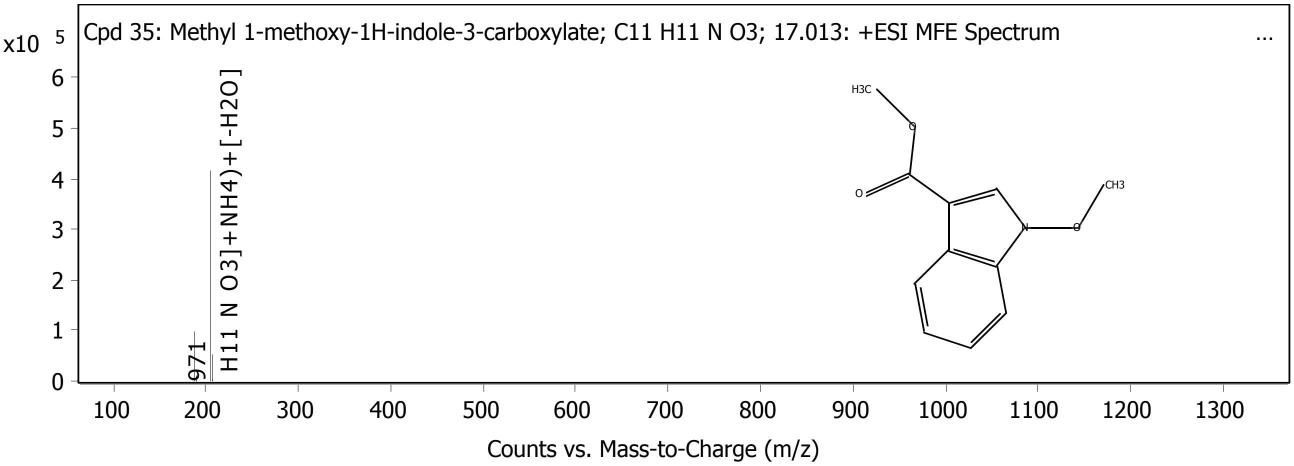

MFE MS Zoomed Spectrum

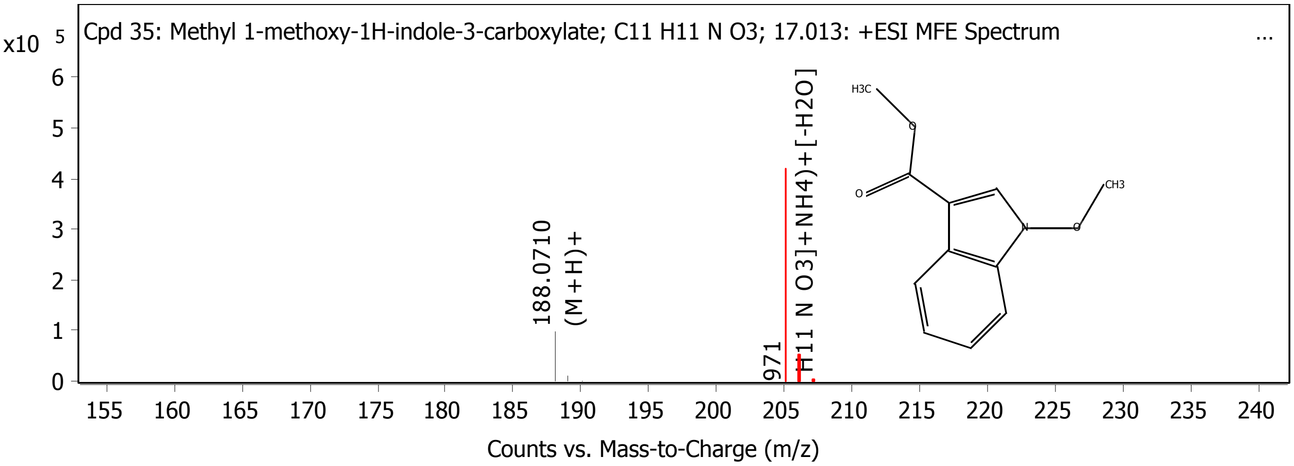

MS Spectrum

Qualitative Compound Identification Report

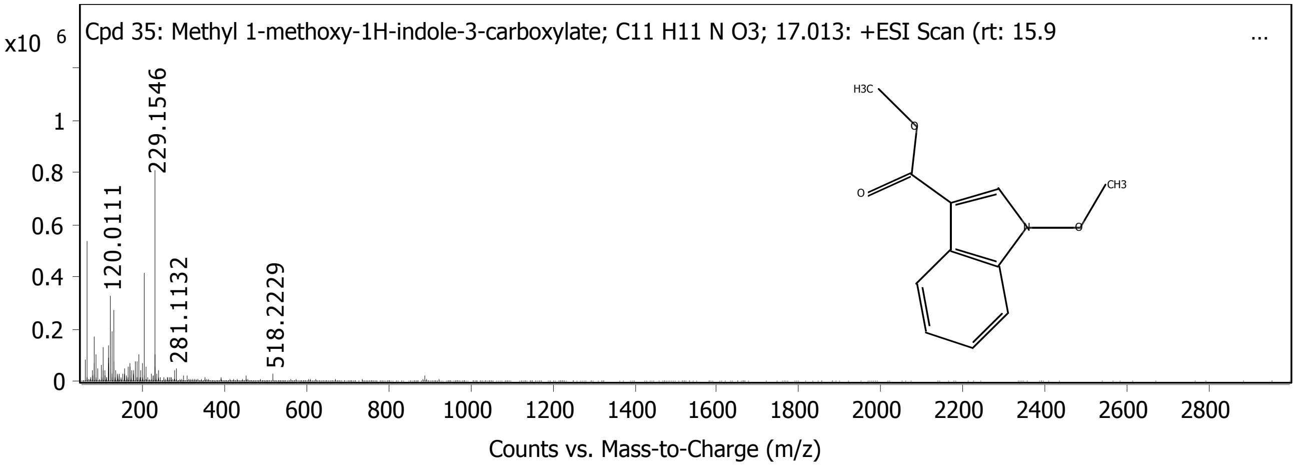

MS Zoomed Spectrum

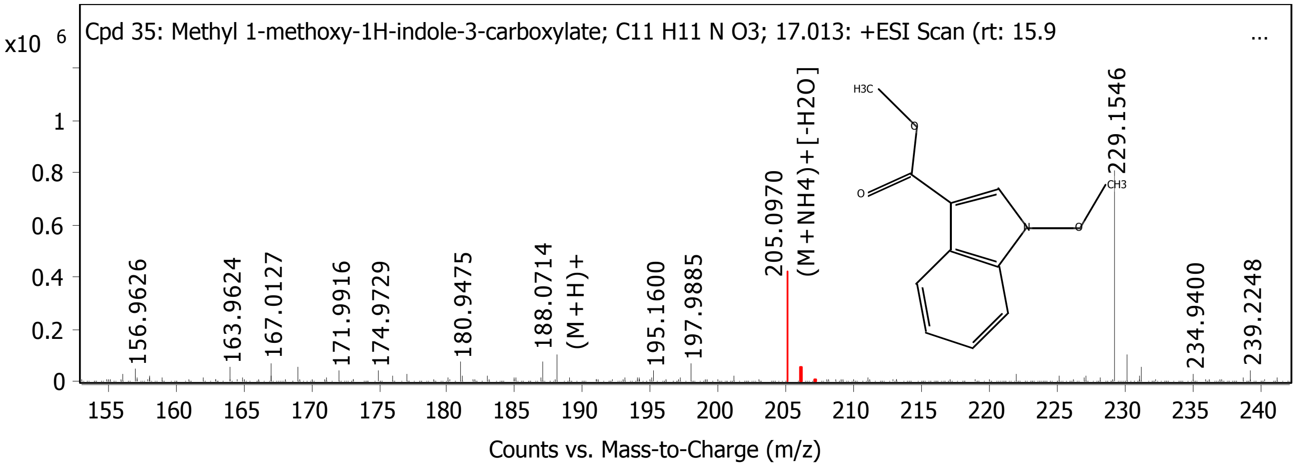

Identification Hit Table

Best Hit	Compound Name	RT	Formula	Notes	Match Score	Mass	Difference	Ion Species
✓	Methyl 1-methoxy-1H-indole-3-carboxylate	17.013	C11 H11 N O3		99.96	205.0738	0.06	(M+NH4)+[-H2O]
	N-(3-Hydroxypropyl)phthalimide	17.013	C11 H11 N O3		99.96	205.0738	0.06	(M+NH4)+[-H2O]
	Swietenidin B	17.013	C11 H11 N O3		99.96	205.0738	0.06	(M+NH4)+[-H2O]
	Gentianamine	17.013	C11 H11 N O3		99.96	205.0738	0.06	(M+NH4)+[-H2O]
	Cinnamoylglycine	17.013	C11 H11 N O3	Pubchem 709625	99.96	205.0738	0.06	(M+NH4)+[-H2O]
	5-Methoxyindoleacetate	17.013	C11 H11 N O3		99.96	205.0738	0.06	(M+NH4)+[-H2O]
	Indolelactic acid	17.013	C11 H11 N O3	Endogenous Metabolite Geigy vol. 3 p. 98. Nugo Website Http://Nugo.org/metabolomics/13496	99.96	205.0738	0.06	(M+NH4)+[-H2O]
	Quinacetol	17.013	C11 H9 N O2		99.96	187.0633	0.06	(M+NH4)+
	3-Amino-2-naphthoic acid	17.013	C11 H9 N O2	Pubchem 22244	99.96	187.0633	0.06	(M+NH4)+
	Indoleacrylic acid	17.013	C11 H9 N O2		99.96	187.0633	0.06	(M+NH4)+

Identification Hit Table

Best Hit	Compound Name	RT	Formula	Notes	Match Score	Mass	Difference	Ion Species

Identification Hit Table

Best Hit	Compound Name	RT	Formula	Notes	Match Score	Mass	Difference	Ion Species

Compound Label	Name	*m/z*	RT	Algorithm	Mass
Cpd 36: L-isoleucyl-L-proline; C11 H20 N2 O3; 17.368	**L-isoleucyl-L-proline**	229.1547	17.368	Find by Molecular Feature	228.1474

Compound Chromatograms

Qualitative Compound Identification Report

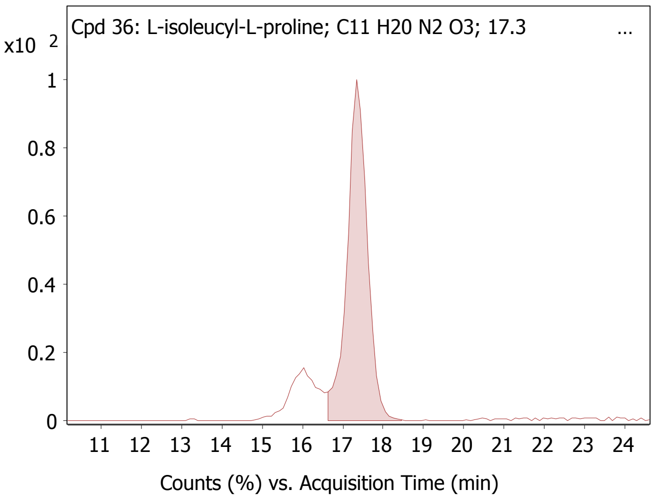

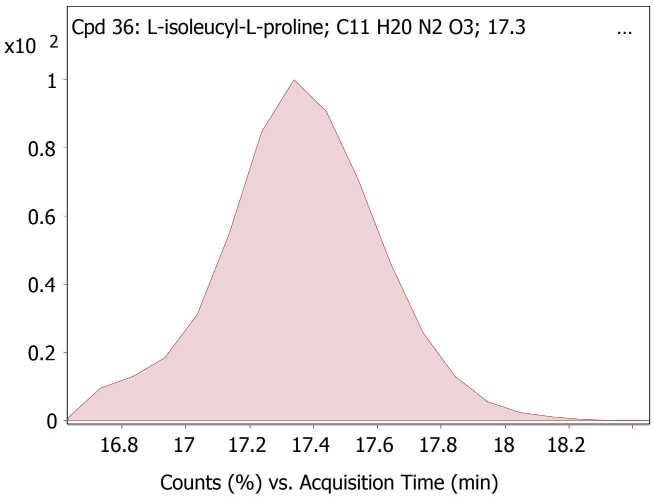

MFE MS Spectrum

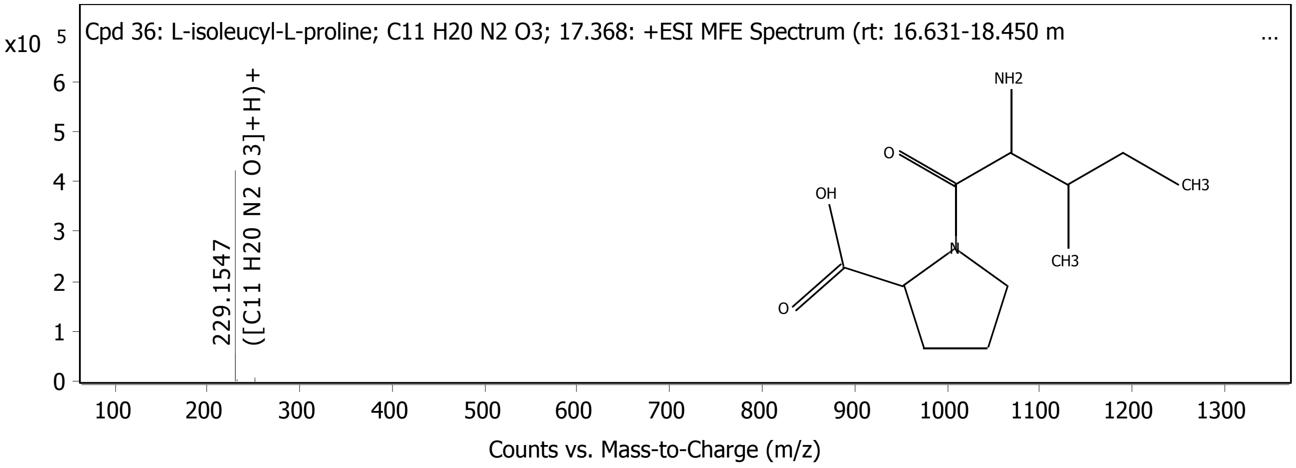

MFE MS Zoomed Spectrum

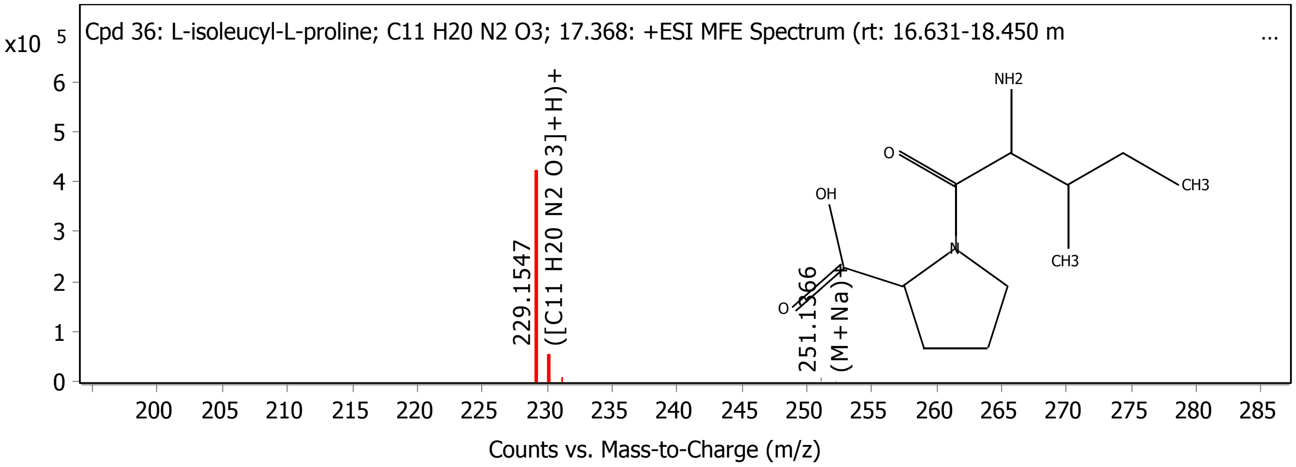

MS Spectrum

Qualitative Compound Identification Report

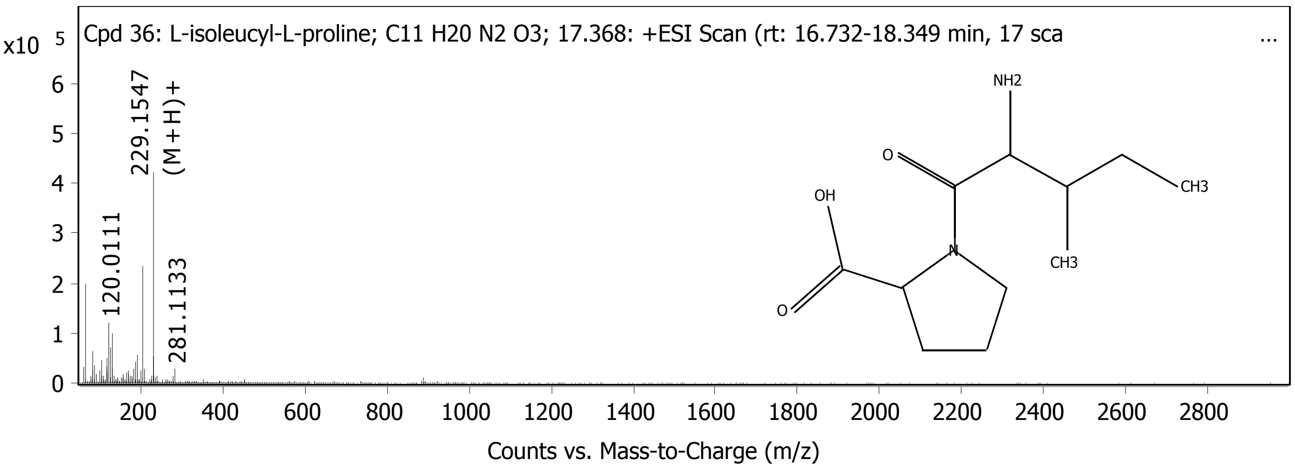

MS Zoomed Spectrum

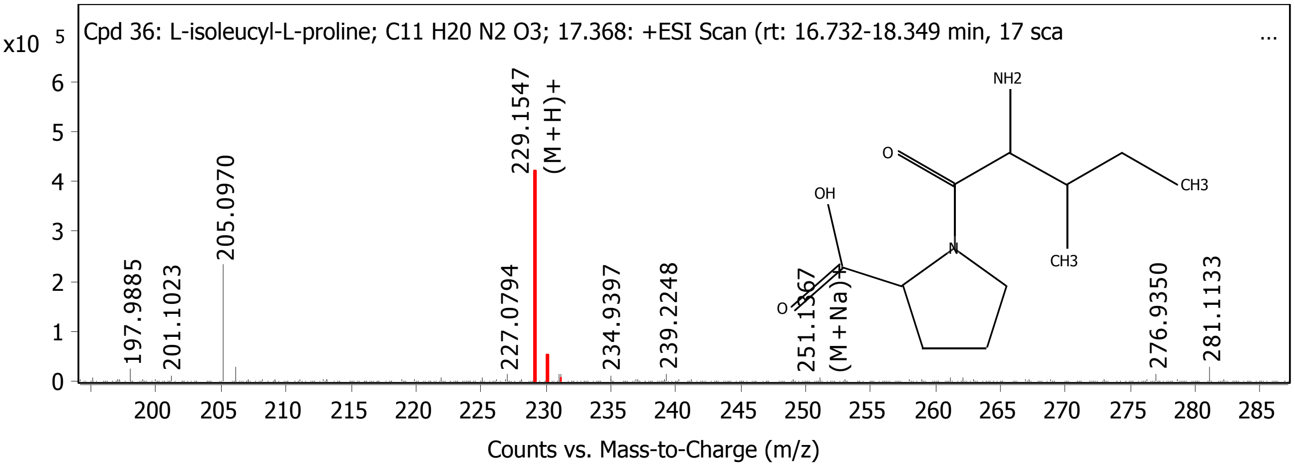

Identification Hit Table

Best Hit	Compound Name	RT	Formula	Notes	Match Score	Mass	Difference	Ion Species
✓	L-isoleucyl-L-proline	17.368	C11 H20 N2 O3		99.92	228.1474	0.01	(M+H)+
	Pro Ile	17.368	C11 H20 N2 O3		99.92	228.1474	0.01	(M+H)+
	Ile Pro	17.368	C11 H20 N2 O3		99.92	228.1474	0.01	(M+H)+
	Pro Leu	17.368	C11 H20 N2 O3		99.92	228.1474	0.01	(M+H)+
	Leu Pro	17.368	C11 H20 N2 O3		99.92	228.1474	0.01	(M+H)+
	1-L-Leucyl-L-Proline	17.368	C11 H20 N2 O3		99.92	228.1474	0.01	(M+H)+

Identification Hit Table

Best Hit	Compound Name	RT	Formula	Notes	Match Score	Mass	Difference	Ion Species

Identification Hit Table

Best Hit	Compound Name	RT	Formula	Notes	Match Score	Mass	Difference	Ion Species
✓	Pro Ile	17.368	C11 H20 N2 O3		99.92	228.1474	0.01	(M+H)+
	Ile Pro	17.368	C11 H20 N2 O3		99.92	228.1474	0.01	(M+H)+
	Pro Leu	17.368	C11 H20 N2 O3		99.92	228.1474	0.01	(M+H)+
	Leu Pro	17.368	C11 H20 N2 O3		99.92	228.1474	0.01	(M+H)+

Compound Label	Name	*m/z*	RT	Algorithm	Mass
Cpd 37: Quercetin 7-[xylosyl-(1->2)-rhamnosyl-(1->2)-rhamnosyl]-(1->6)-glucoside; C38 H48 O24; 18.971	**Quercetin 7-[xylosyl-(1->2)-rhamnosyl-(1->2)-rhamnosyl]-(1->6)-glucoside**	889.2583	18.971	Find by Molecular Feature	888.2509

Compound Chromatograms

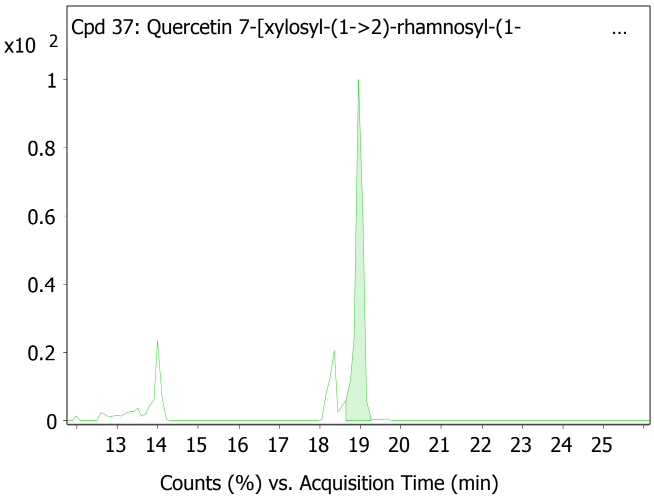

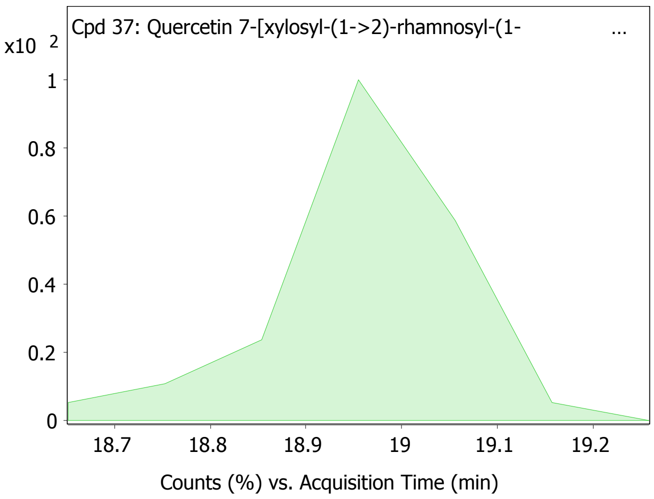

MFE MS Spectrum

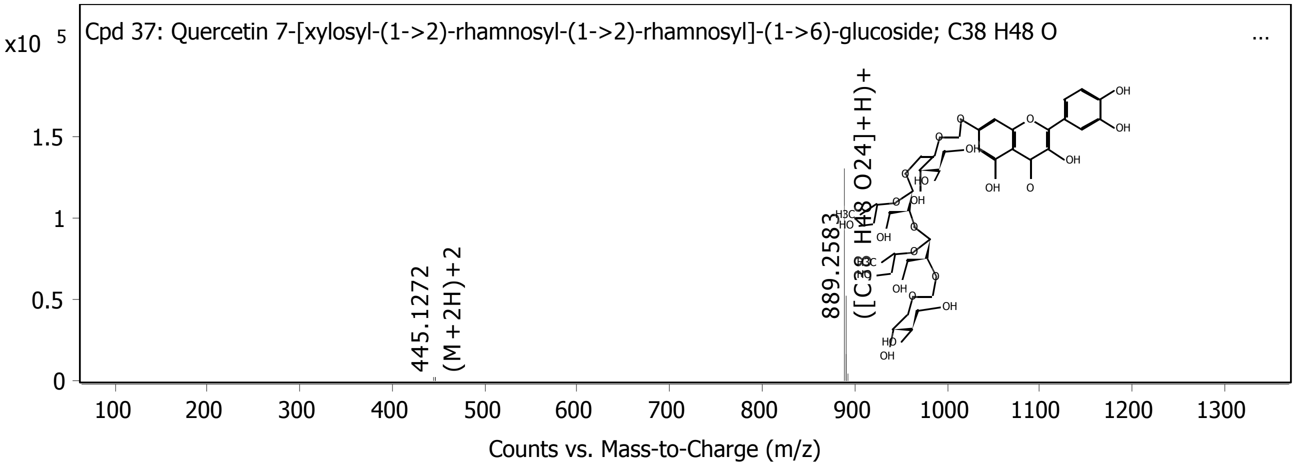

MFE MS Zoomed Spectrum

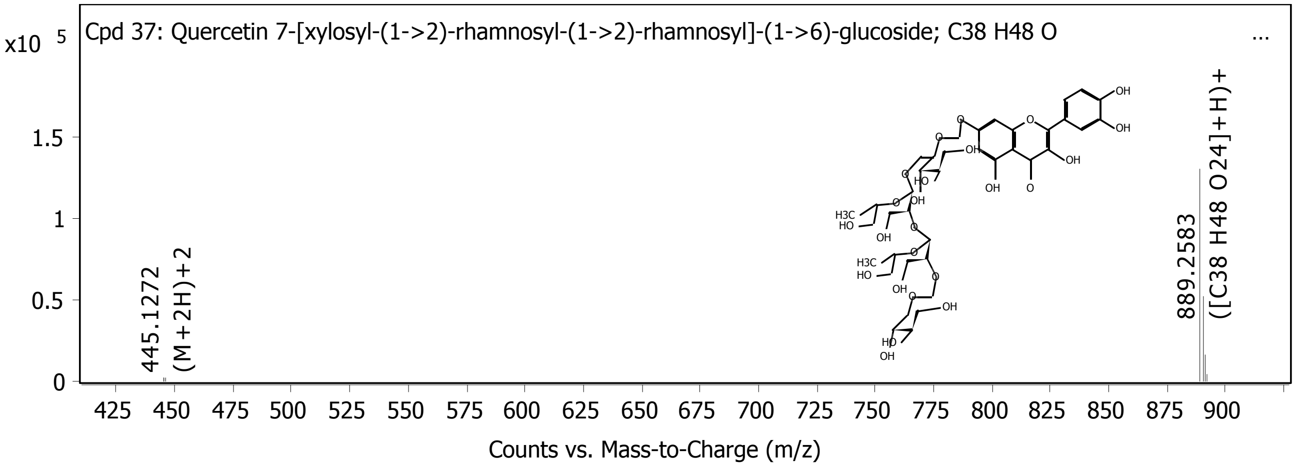

MS Spectrum

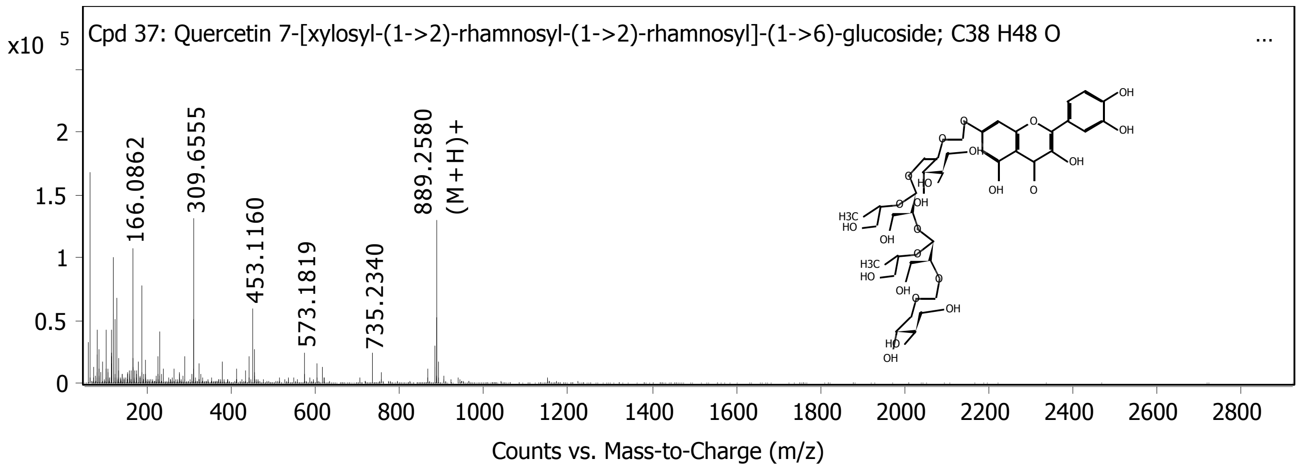

MS Zoomed Spectrum

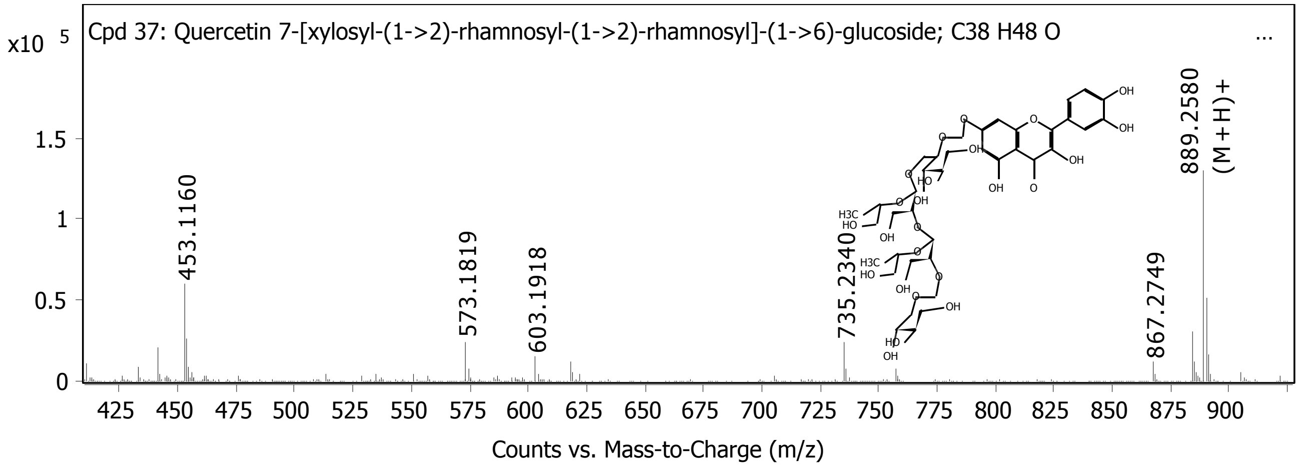

Identification Hit Table

Best Hit	Compound Name	RT	Formula	Notes	Match Score	Mass	Difference	Ion Species
✓	Quercetin 7-[xylosyl-(1->2)-rhamnosyl-(1->2)-rhamnosyl]-(1->6)-glucoside	18.971	C38 H48 O24		92.64	888.2509	2.69	(M+H)+
	Kaempferol 3-(2G-xylosylrutinoside)-7-glucoside	18.971	C38 H48 O24		92.64	888.2509	2.69	(M+H)+
	r-Viniferin	18.971	C56 H42 O12		55.35	906.2614	6.26	(M+H)+[-H2O]
	Viniferol A	18.971	C56 H42 O12		55.35	906.2614	6.26	(M+H)+[-H2O]
	Isohopeaphenol	18.971	C56 H42 O12		55.35	906.2614	6.26	(M+H)+[-H2O]
	Vitisin C	18.971	C56 H42 O12		55.35	906.2614	6.26	(M+H)+[-H2O]

Identification Hit Table

Best Hit	Compound Name	RT	Formula	Notes	Match Score	Mass	Difference	Ion Species
✓	Kaempferol 3-(2G-xylosylrutinoside)-7-glucoside	18.971	C38 H48 O24		92.64	888.2509	2.69	(M+H)+
	Quercetin 7-[xylosyl-(1->2)-rhamnosyl-(1->2)-rhamnosyl]-(1->6)-glucoside	18.971	C38 H48 O24		92.64	888.2509	2.69	(M+H)+

Identification Hit Table

Best Hit	Compound Name	RT	Formula	Notes	Match Score	Mass	Difference	Ion Species

Compound Label	Name	*m/z*	RT	Algorithm	Mass
Cpd 38: Asn Trp Trp Leu; C32 H39 N7 O6; 19.178	**Asn Trp Trp Leu**	309.6556	19.178	Find by Molecular Feature	617.2966

Compound Chromatograms

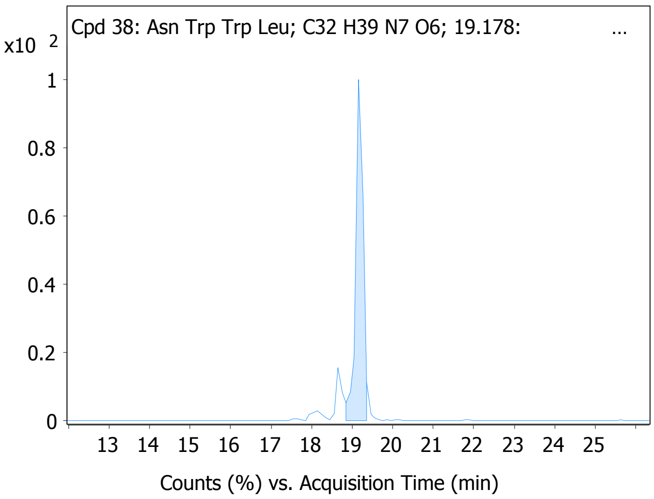

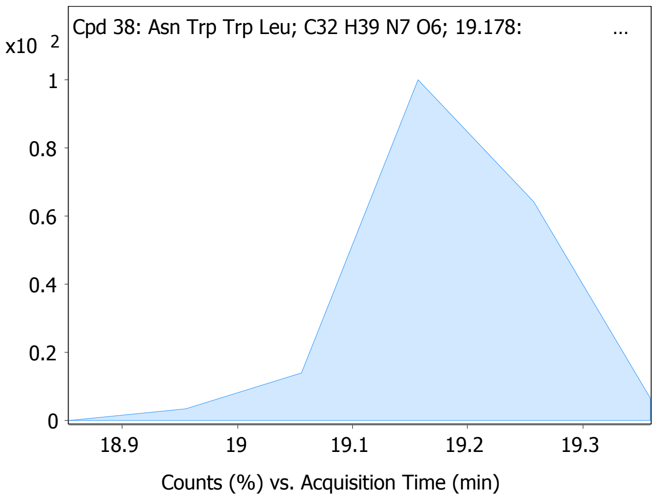

MFE MS Spectrum

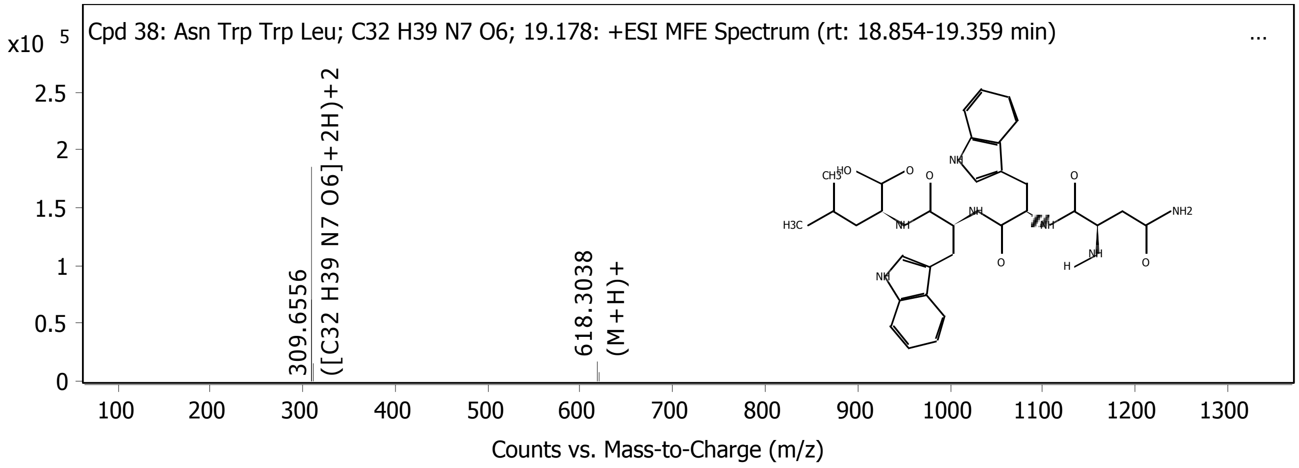

MFE MS Zoomed Spectrum

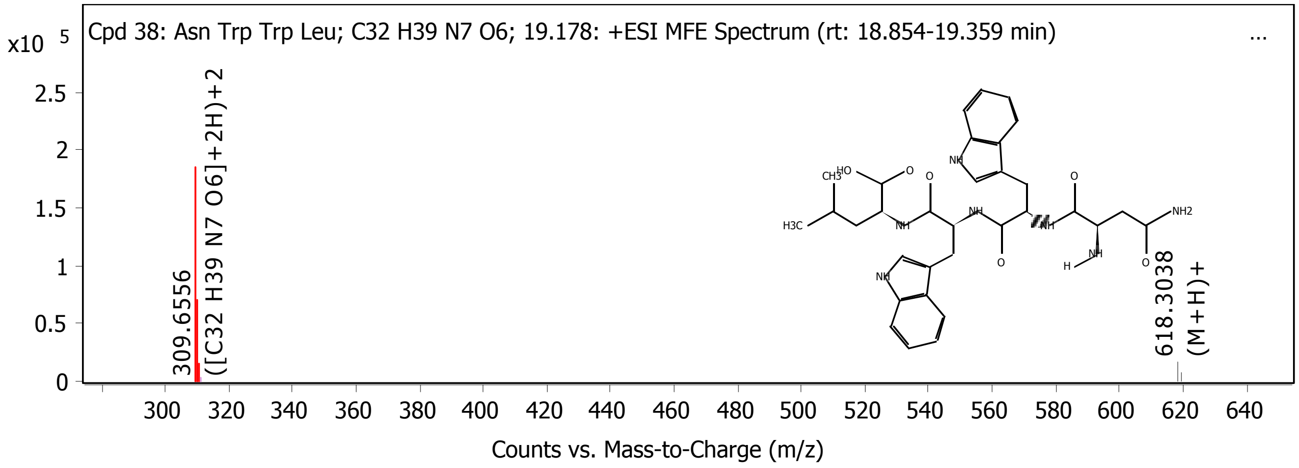

MS Spectrum

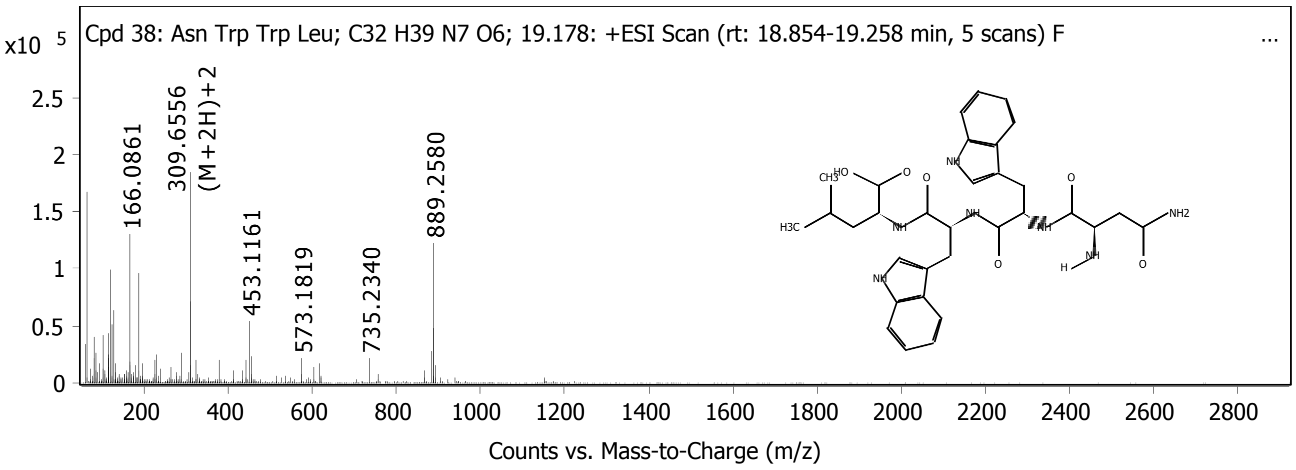

MS Zoomed Spectrum

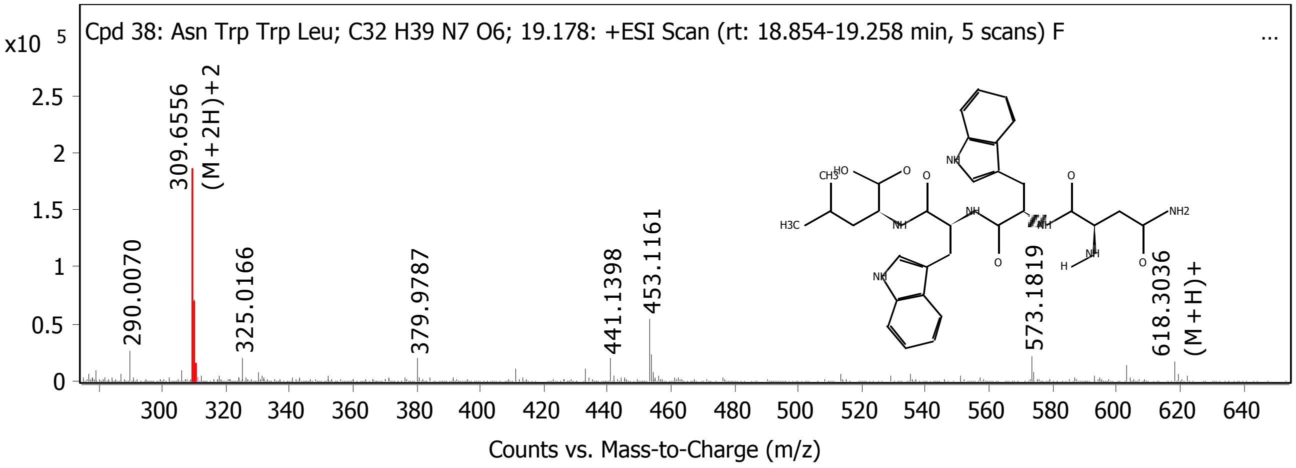

MSMS Spectrum

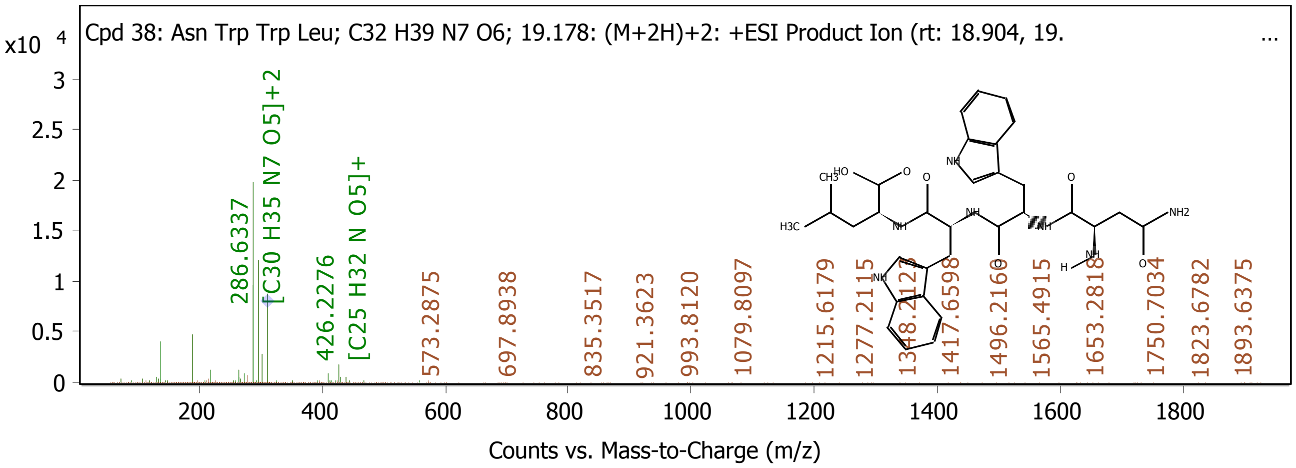

Identification Hit Table

Best Hit	Compound Name	RT	Formula	Notes	Match Score	Mass	Difference	Ion Species
✓	Glycylserylprolylmethionylphenylal anylvalinamide	19.178	C29 H45 N7 O7 S		82.38	635.3074	2.71	(M+2H)+2[-H2O]

Identification Hit Table

Best Hit	Compound Name	RT	Formula	Notes	Match Score	Mass	Difference	Ion Species

Identification Hit Table

Best Hit	Compound Name	RT	Formula	Notes	Match Score	Mass	Difference	Ion Species
✓	Asn Trp Trp Leu	19.178	C32 H39 N7 O6		99.13	617.2966	-0.41	(M+2H)+2
	His Trp Ile Tyr	19.178	C32 H39 N7 O6		99.13	617.2966	-0.41	(M+2H)+2
	Ile His Trp Tyr	19.178	C32 H39 N7 O6		99.13	617.2966	-0.41	(M+2H)+2
	His Tyr Trp Ile	19.178	C32 H39 N7 O6		99.13	617.2966	-0.41	(M+2H)+2
	Gln Val Trp Trp	19.178	C32 H39 N7 O6		99.13	617.2966	-0.41	(M+2H)+2

	His Tyr Leu Trp	19.178	C32 H39 N7 O6		99.13	617.2966	-0.41	(M+2H)+2
	His Tyr Ile Trp	19.178	C32 H39 N7 O6		99.13	617.2966	-0.41	(M+2H)+2
	Gln Trp Val Trp	19.178	C32 H39 N7 O6		99.13	617.2966	-0.41	(M+2H)+2
	Gln Trp Trp Val	19.178	C32 H39 N7 O6		99.13	617.2966	-0.41	(M+2H)+2
	His Trp Tyr Leu	19.178	C32 H39 N7 O6		99.13	617.2966	-0.41	(M+2H)+2

Compound Label	*m/z*	RT	Algorithm	Mass
Cpd 39: C38 H46 N6 O9; 21.102	366.1738	21.102	Find by Molecular Feature	730.3332

Compound Chromatograms

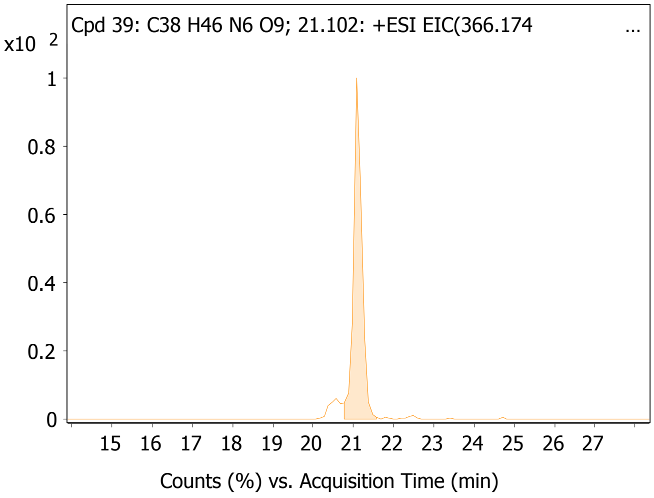

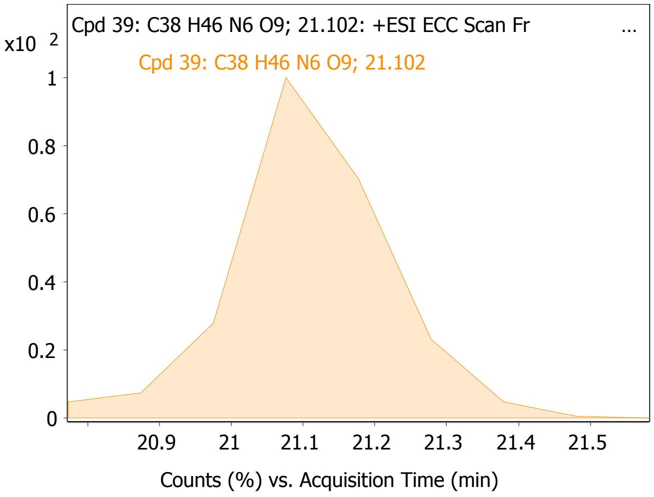

MFE MS Spectrum

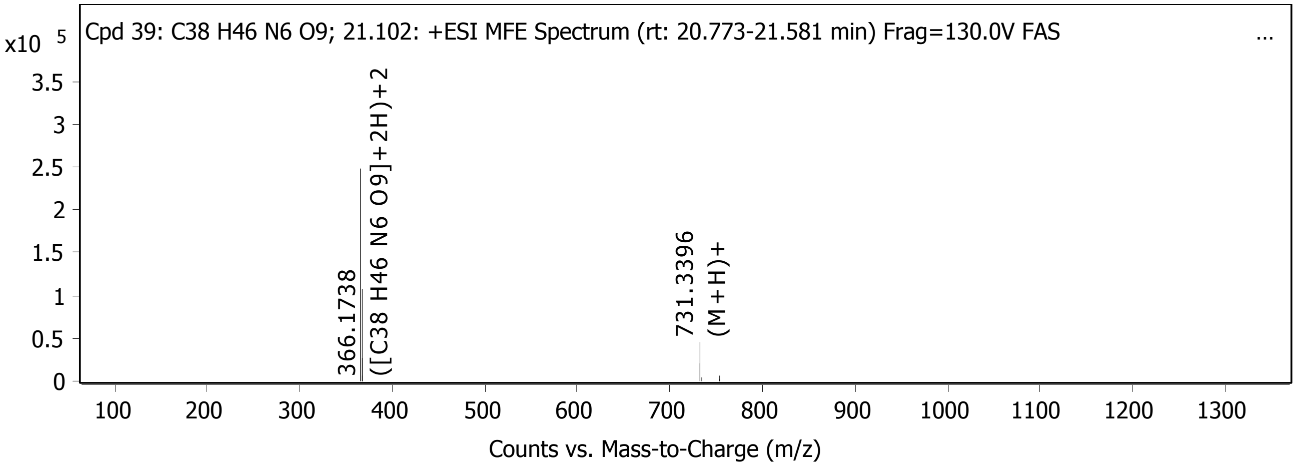

MFE MS Zoomed Spectrum

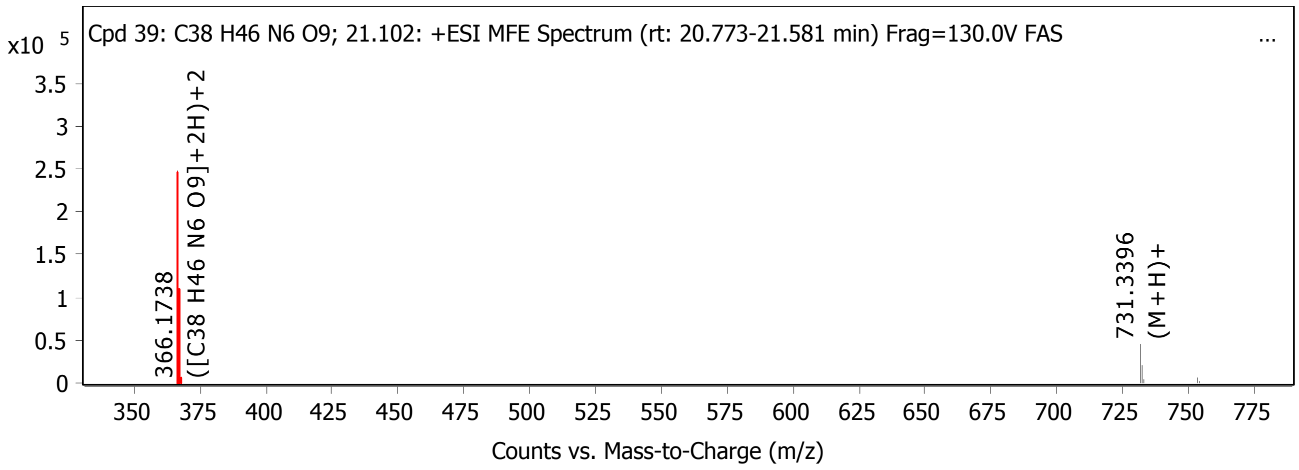

MS Spectrum

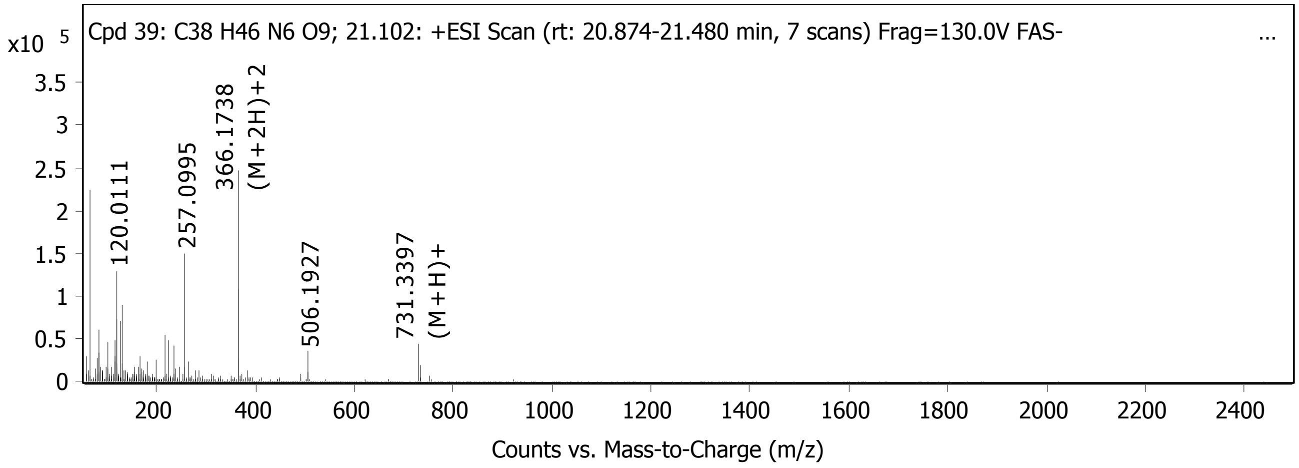

MS Zoomed Spectrum

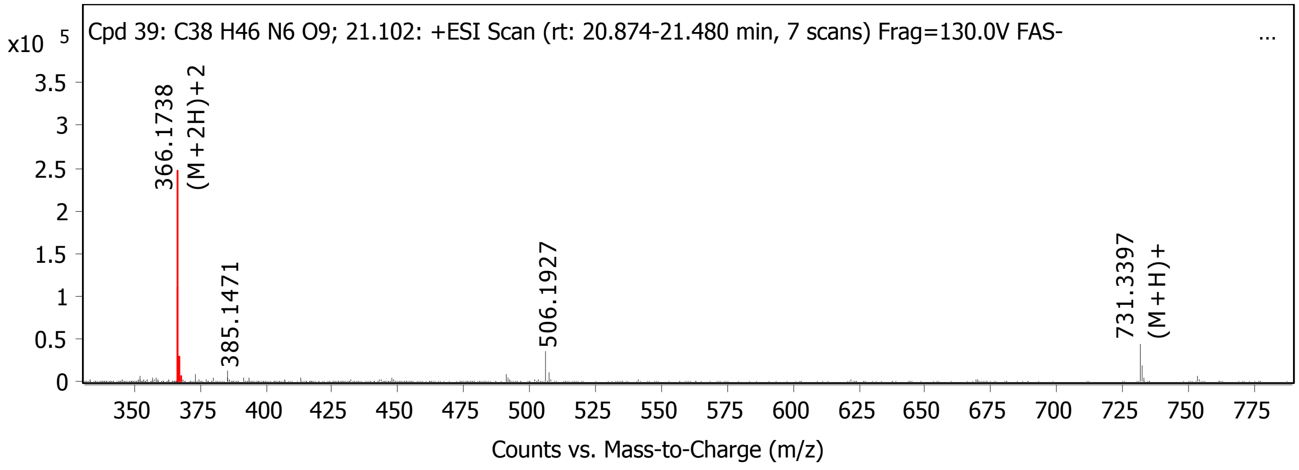

MSMS Spectrum

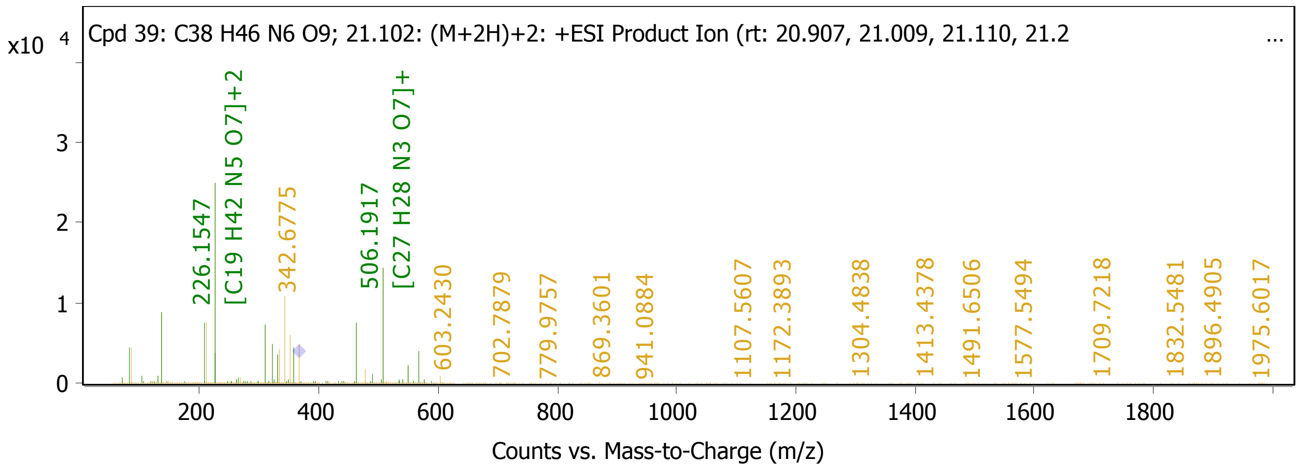

Compound Label	*m/z*	RT	Algorithm	Mass
Cpd 40: C27 H27 N3 O7; 23.441	506.1934	23.441	Find by Molecular Feature	505.1852

Compound Chromatograms

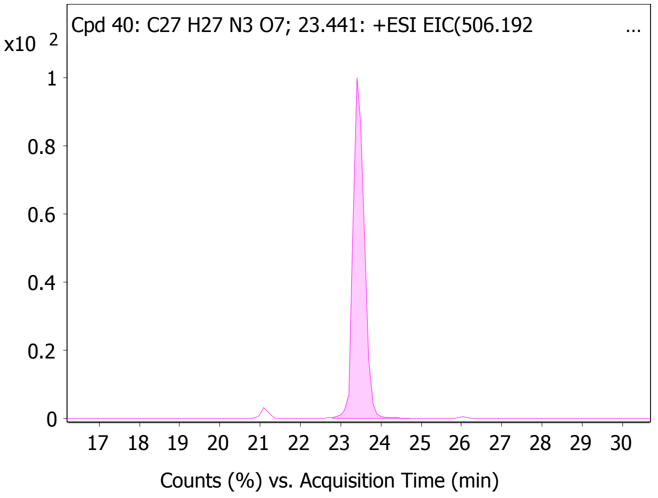

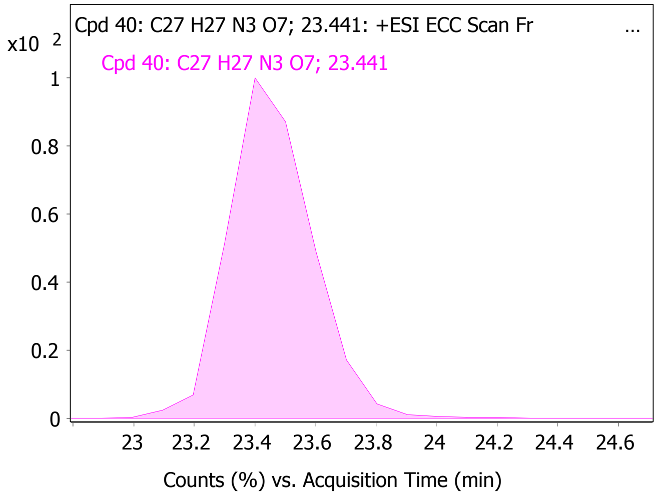

MFE MS Spectrum

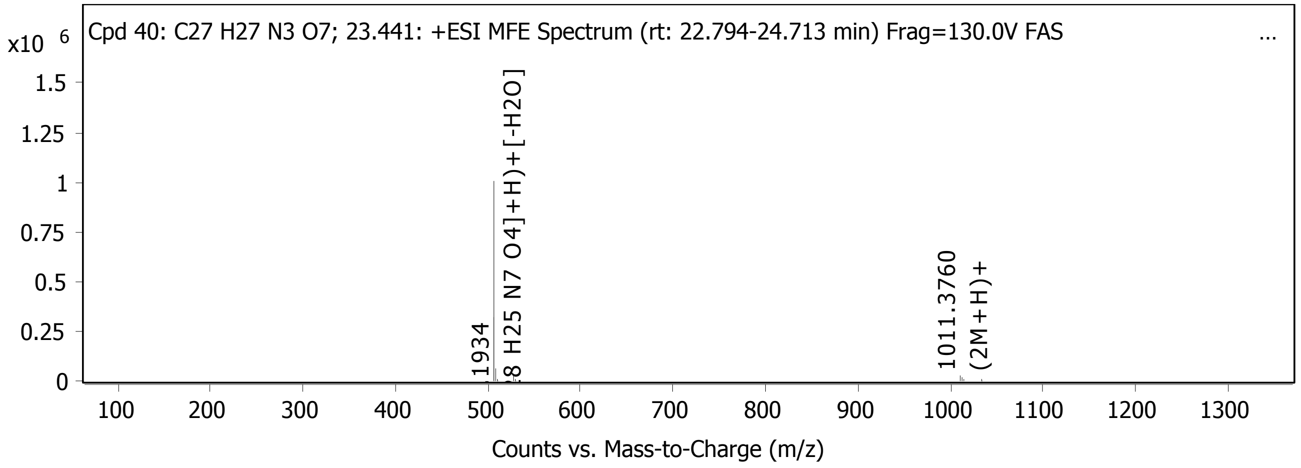

MFE MS Zoomed Spectrum

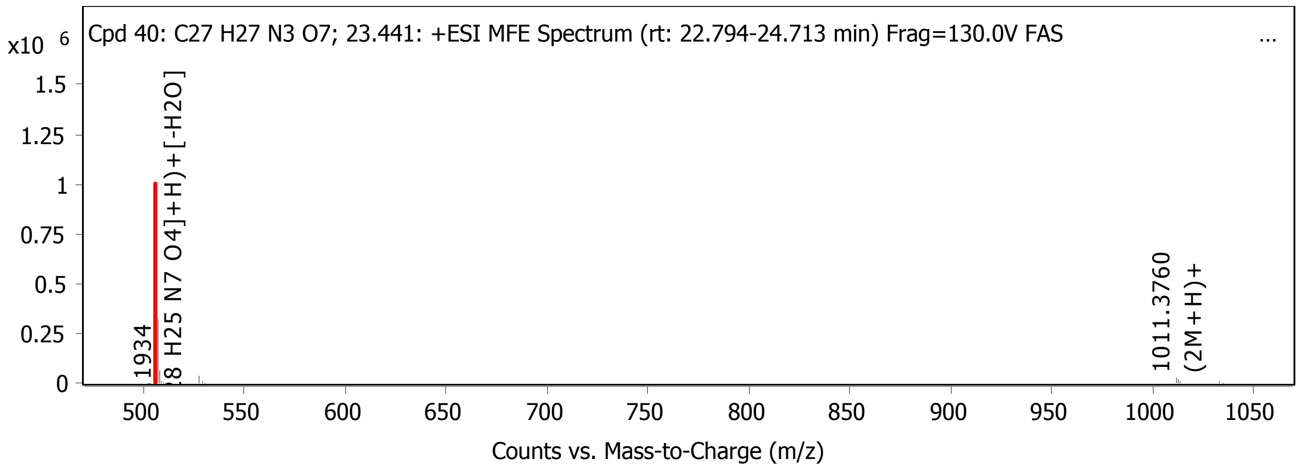

MS Spectrum

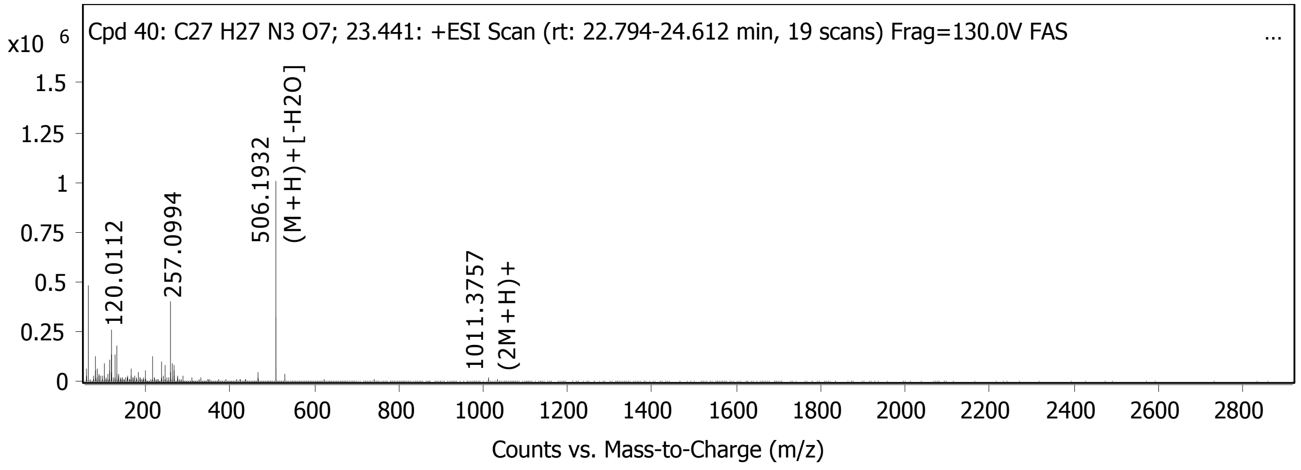

MS Zoomed Spectrum

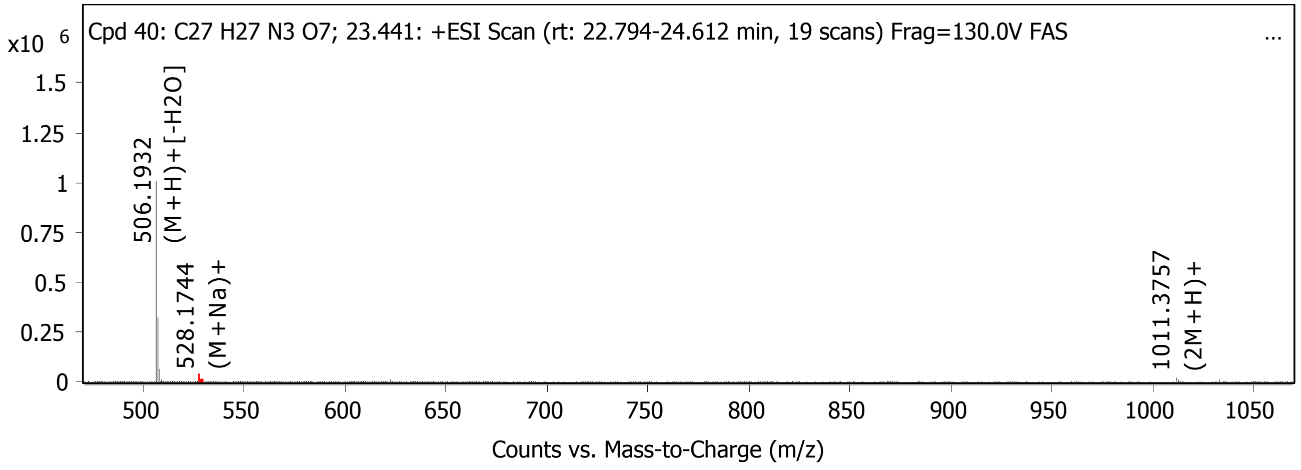

Compound Label	*m/z*	RT	Algorithm	Mass
Cpd 41: C41 H50 N3 O10; 29.806	373.1821	29.806	Find by Molecular Feature	744.3494

Compound Chromatograms

Qualitative Compound Identification Report

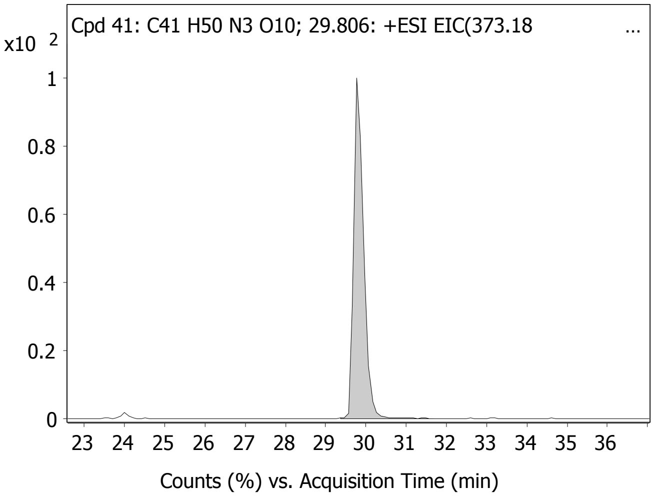

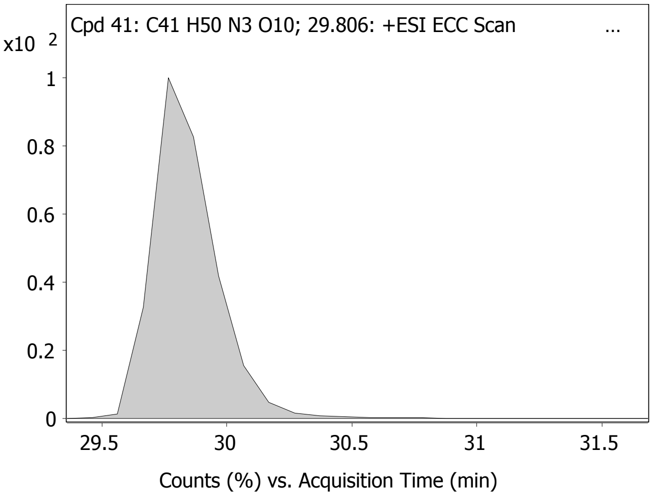

MFE MS Spectrum

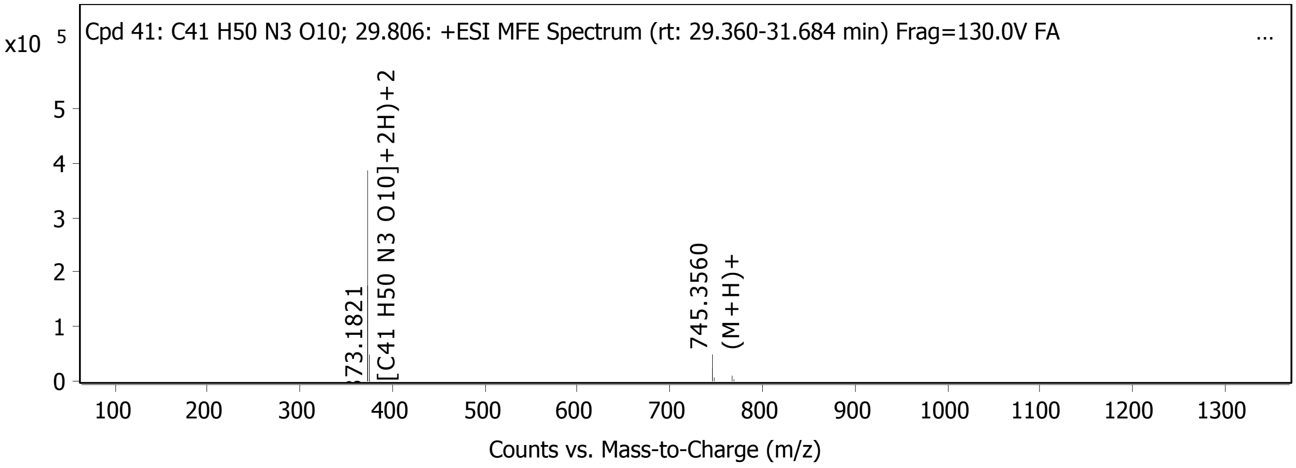

MFE MS Zoomed Spectrum

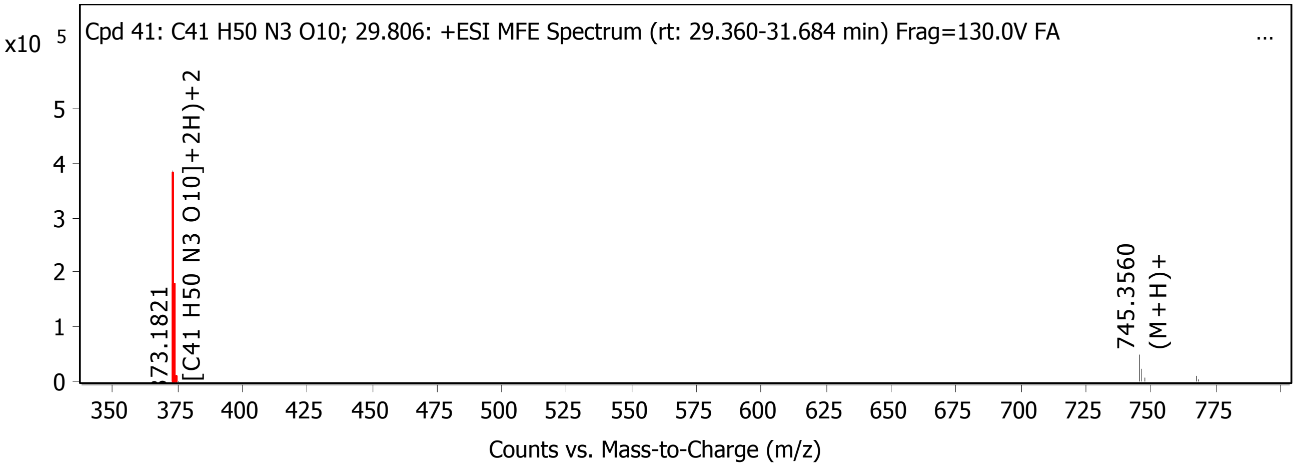

MS Spectrum

Qualitative Compound Identification Report

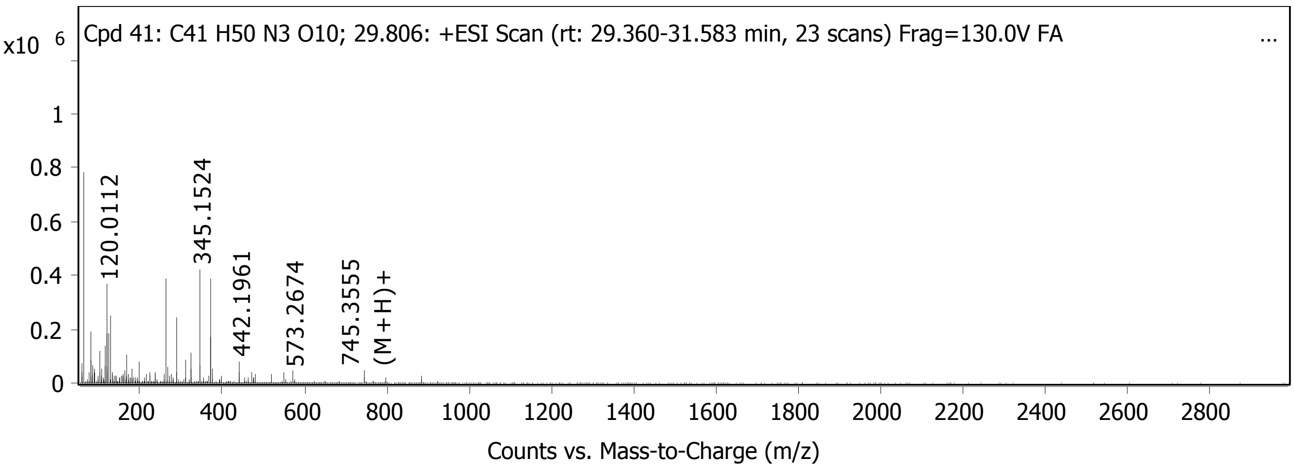

MS Zoomed Spectrum

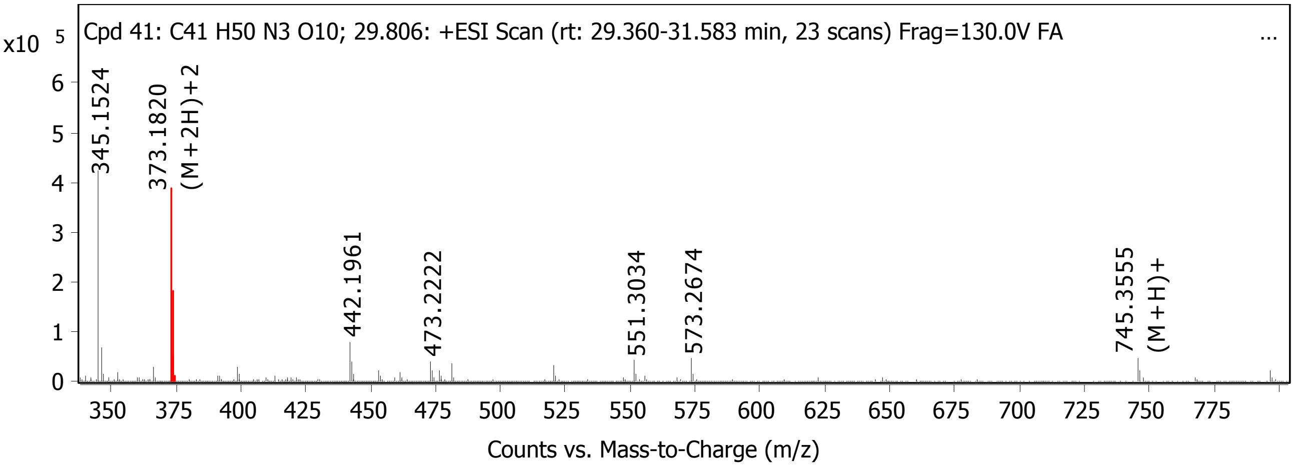

MSMS Spectrum

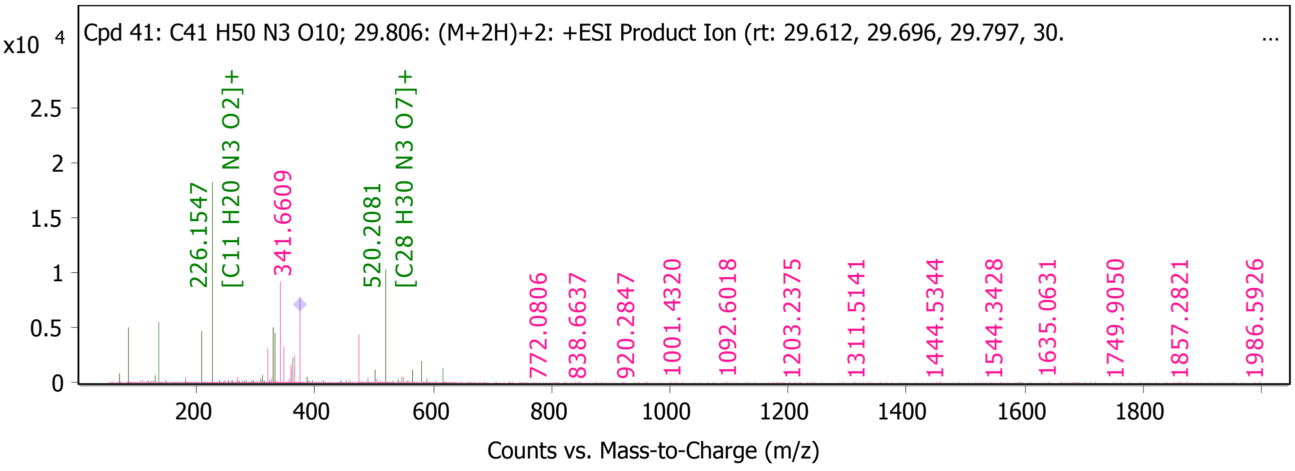

Compound Label	Name	*m/z*	RT	Algorithm	Mass
Cpd 42: Butyl 3-O-beta-D-glucopyranosyl-butanoate; C14 H26 O8; 30.862	**Butyl 3-O-beta-D-glucopyranosyl-butanoate**	345.1524	30.862	Find by Molecular Feature	322.1632

Compound Chromatograms

Qualitative Compound Identification Report

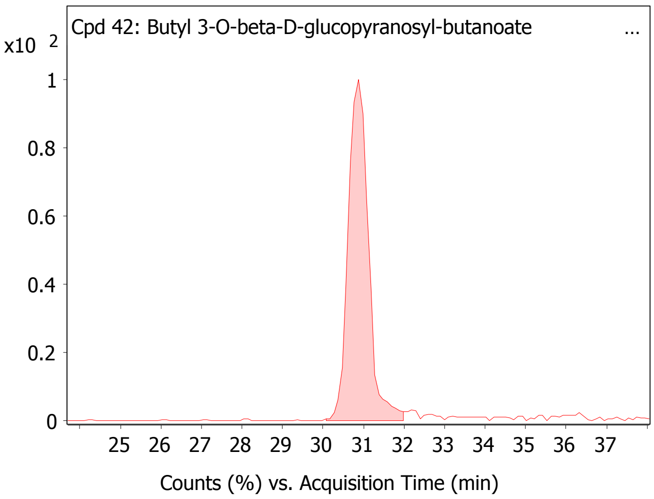

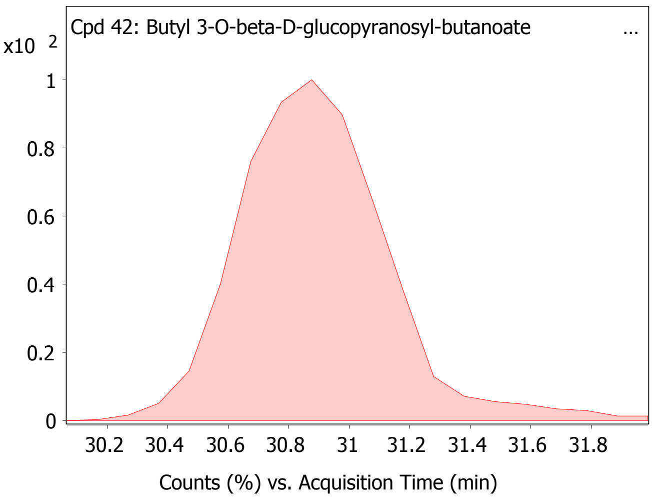

MFE MS Spectrum

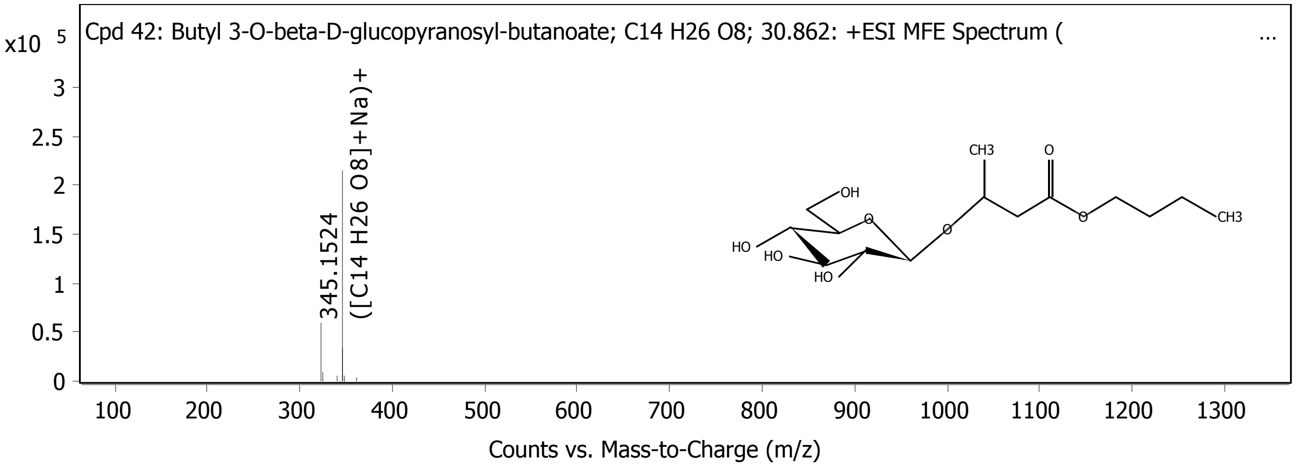

MFE MS Zoomed Spectrum

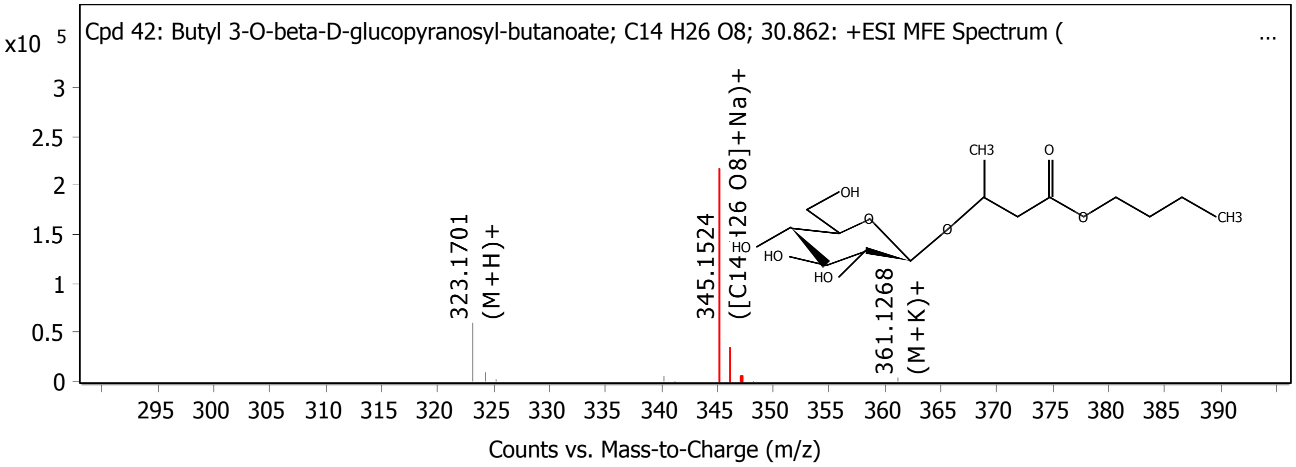

MS Spectrum

Qualitative Compound Identification Report

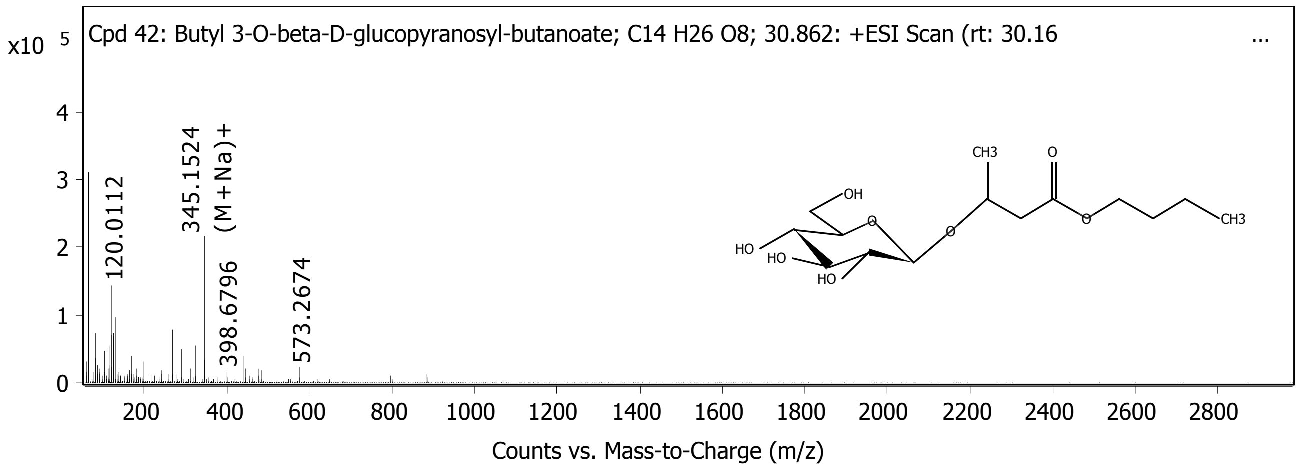

MS Zoomed Spectrum

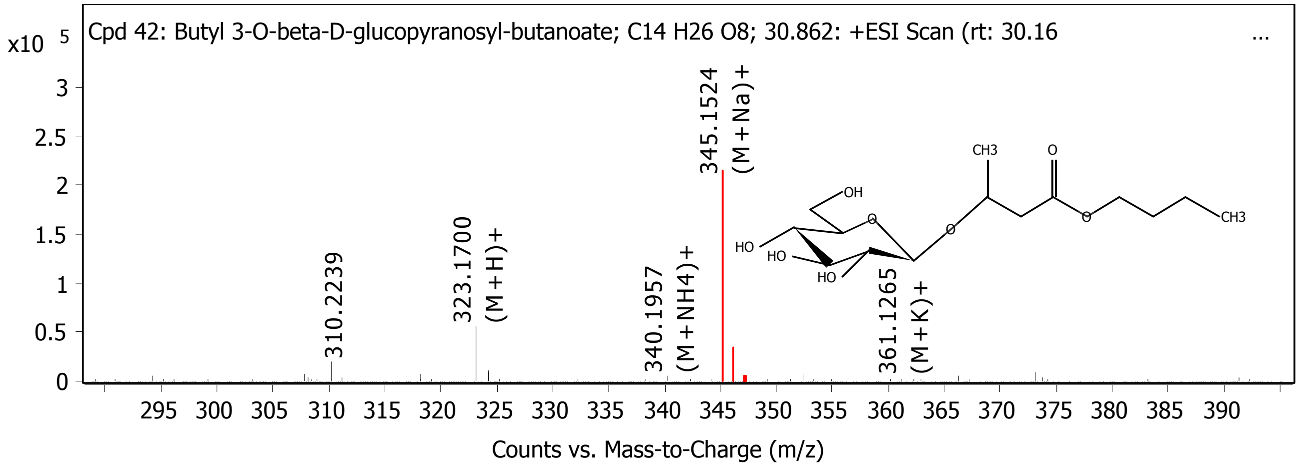

Identification Hit Table

Best Hit	Compound Name	RT	Formula	Notes	Match Score	Mass	Difference	Ion Species
✓	Butyl 3-O-beta-D-glucopyranosyl-butanoate	30.862	C14 H26 O8		99.43	322.1632	-0.39	(M+Na)+
	Butyl (S)-3-hydroxybutyrate glucoside	30.862	C14 H26 O8		99.43	322.1632	-0.39	(M+Na)+
	Gln Pro Pro	30.862	C15 H24 N4 O5		96.25	340.1738	0.88	(M+Na)+[-H2O]
	Pro Pro Gln	30.862	C15 H24 N4 O5		96.25	340.1738	0.88	(M+Na)+[-H2O]
	Pro Gln Pro	30.862	C15 H24 N4 O5		96.25	340.1738	0.88	(M+Na)+[-H2O]
	PtdIns-(1-arachidonoyl-d8, 2-arachidonoyl)	30.862	C14 H22 D3 N O5 S		91.75	322.1634	0.81	(M+Na)+
	4-hydroxy Nonenal Mercapturic Acid-d3	30.862	C14 H22 D3 N O5 S		91.75	322.1634	0.81	(M+Na)+
	11-Hydroxytubotaiwine	30.862	C20 H24 N2 O3		52.84	340.1737	4.96	(M+Na)+[-H2O]
	Yohimbic acid	30.862	C20 H24 N2 O3	derivative	52.84	340.1737	4.96	(M+Na)+[-H2O]
	Quinine-N-Oxide	30.862	C20 H24 N2 O3	Antimalarial Metabolite of Quinine Dollery, Colin Therapeutic Drugs, 2nd Ed. 1999 p. Q16	52.84	340.1737	4.96	(M+Na)+[-H2O]

Identification Hit Table

Best Hit	Compound Name	RT	Formula	Notes	Match Score	Mass	Difference	Ion Species
✓	Butyl 3-O-beta-D-glucopyranosyl-butanoate	30.862	C14 H26 O8		99.43	322.1632	-0.39	(M+Na)+

Identification Hit Table

Best Hit	Compound Name	RT	Formula	Notes	Match Score	Mass	Difference	Ion Species
✓	Pro Pro Gly Ala	30.862	C15 H24 N4 O5		96.25	340.1738	0.88	(M+Na)+[-H2O]
	Pro Pro Ala Gly	30.862	C15 H24 N4 O5		96.25	340.1738	0.88	(M+Na)+[-H2O]
	Pro Gly Pro Ala	30.862	C15 H24 N4 O5		96.25	340.1738	0.88	(M+Na)+[-H2O]
	Pro Gly Ala Pro	30.862	C15 H24 N4 O5		96.25	340.1738	0.88	(M+Na)+[-H2O]
	Pro Ala Pro Gly	30.862	C15 H24 N4 O5		96.25	340.1738	0.88	(M+Na)+[-H2O]
	Pro Ala Gly Pro	30.862	C15 H24 N4 O5		96.25	340.1738	0.88	(M+Na)+[-H2O]
	Gly Pro Pro Ala	30.862	C15 H24 N4 O5		96.25	340.1738	0.88	(M+Na)+[-H2O]
	Gly Pro Ala Pro	30.862	C15 H24 N4 O5		96.25	340.1738	0.88	(M+Na)+[-H2O]
	Gly Ala Pro Pro	30.862	C15 H24 N4 O5		96.25	340.1738	0.88	(M+Na)+[-H2O]
	Ala Pro Pro Gly	30.862	C15 H24 N4 O5		96.25	340.1738	0.88	(M+Na)+[-H2O]

Compound Label	m/z	RT	Algorithm	Mass
Cpd 43: C28 H29 N3 O7; 35.517	520.2093	35.517	Find by Molecular Feature	519.2009

Compound Chromatograms

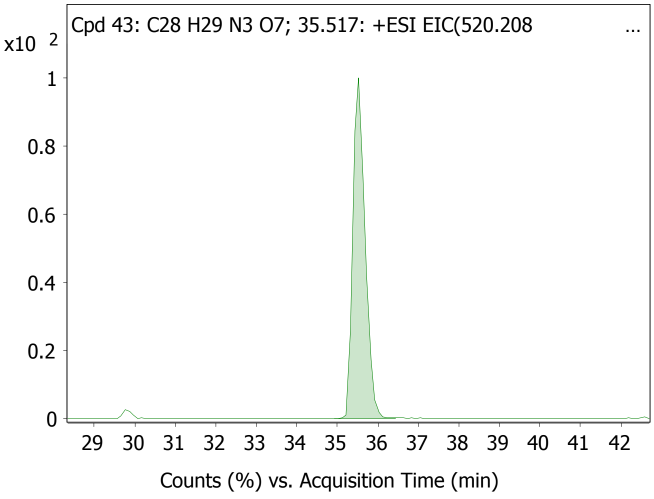

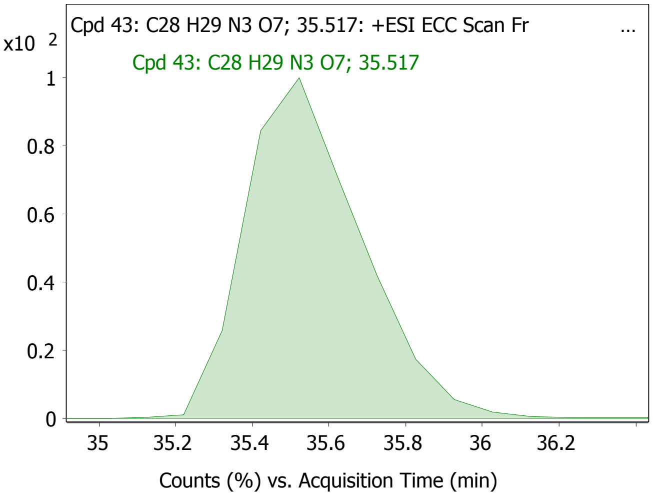

MFE MS Spectrum

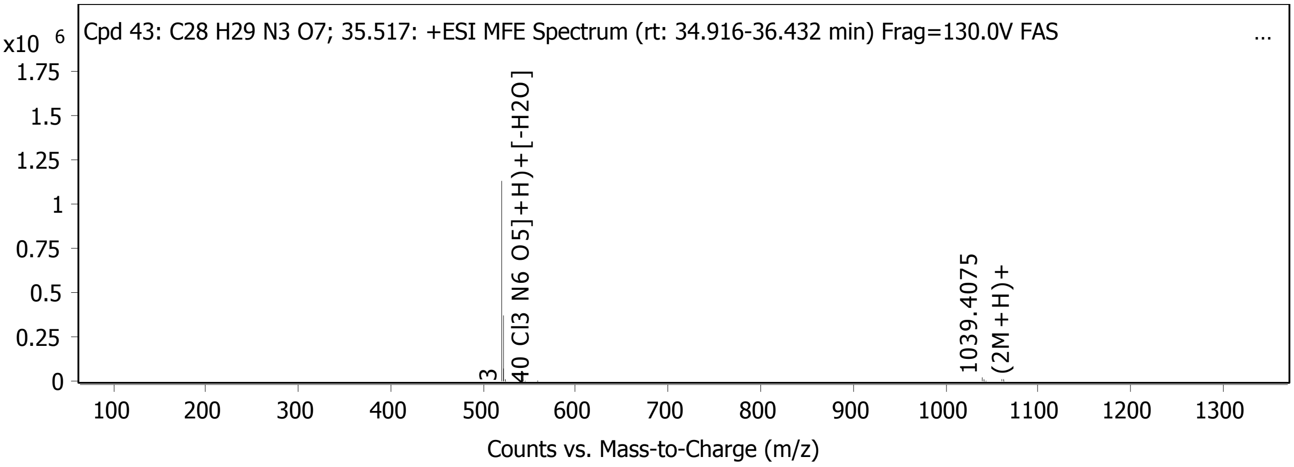

MFE MS Zoomed Spectrum

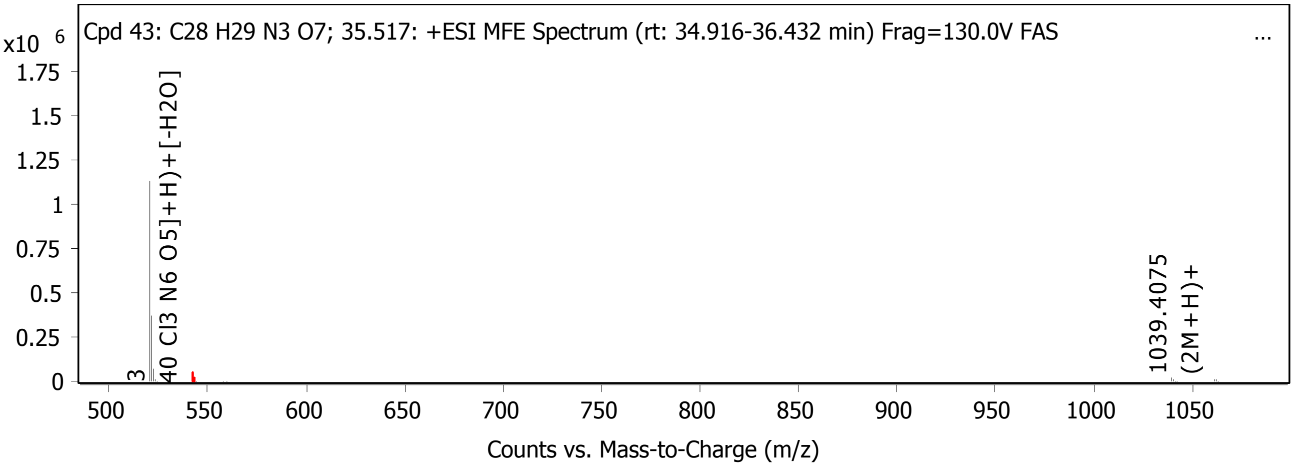

MS Spectrum

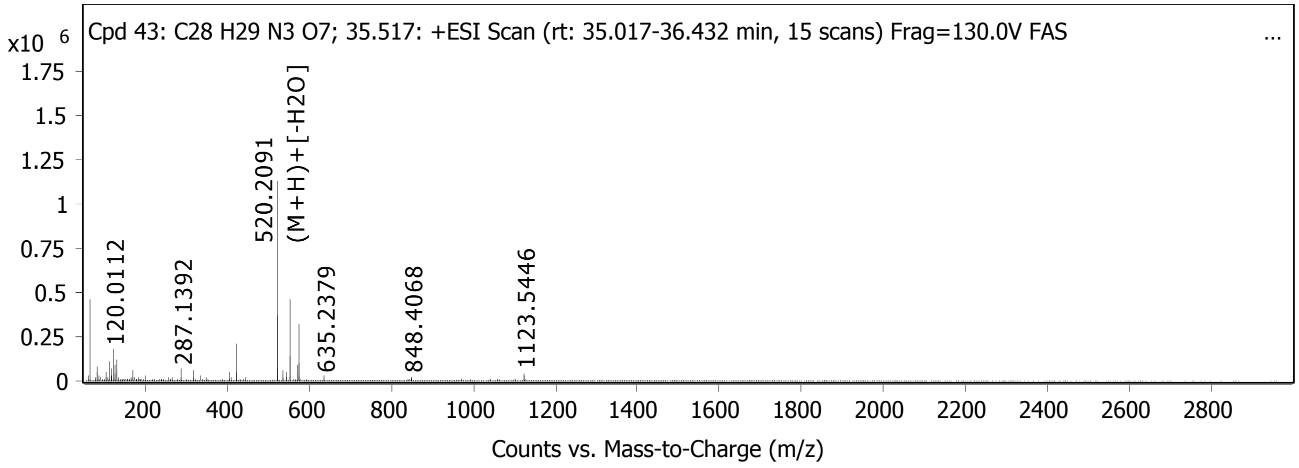

MS Zoomed Spectrum

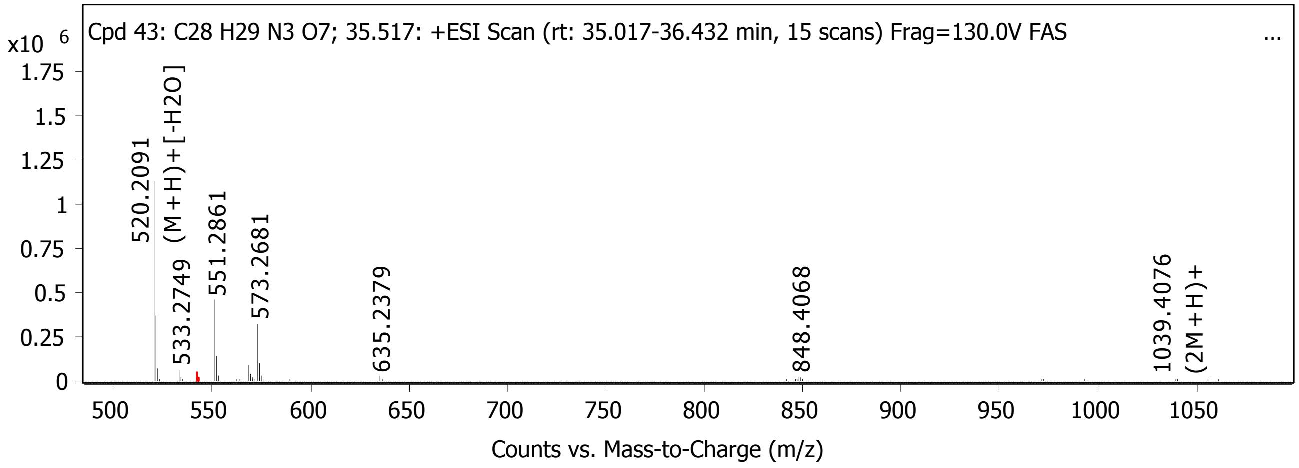

Compound Label	*m/z*	RT	Algorithm	Mass
Cpd 44: 36.212	570.2865	36.212	Find by Molecular Feature	569.2792

Compound Chromatograms

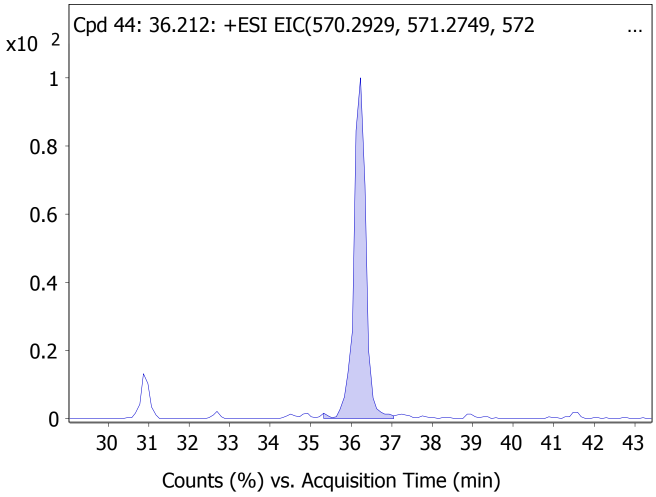

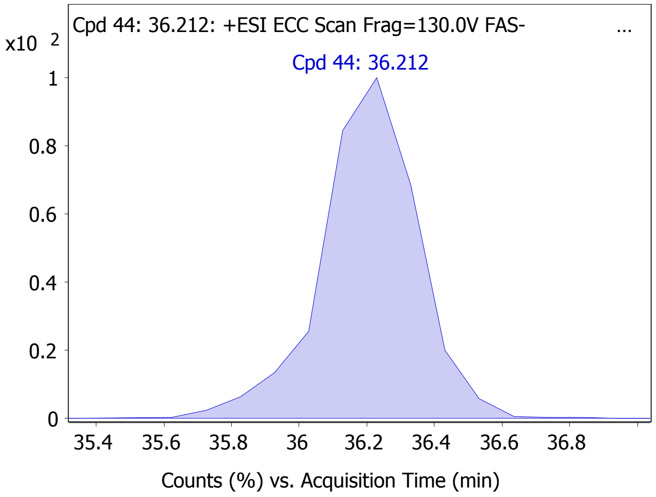

MFE MS Spectrum

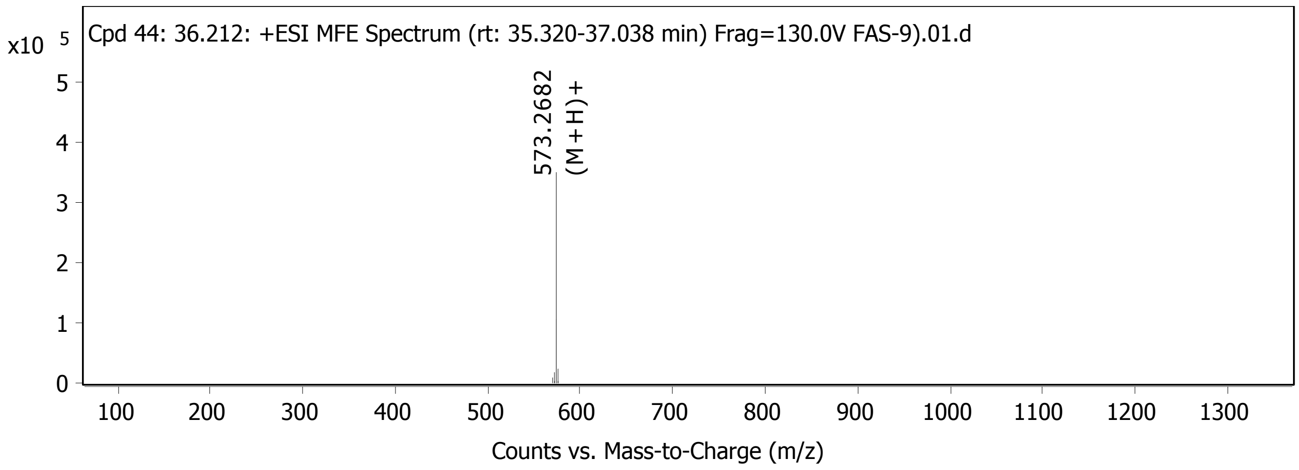

MFE MS Zoomed Spectrum

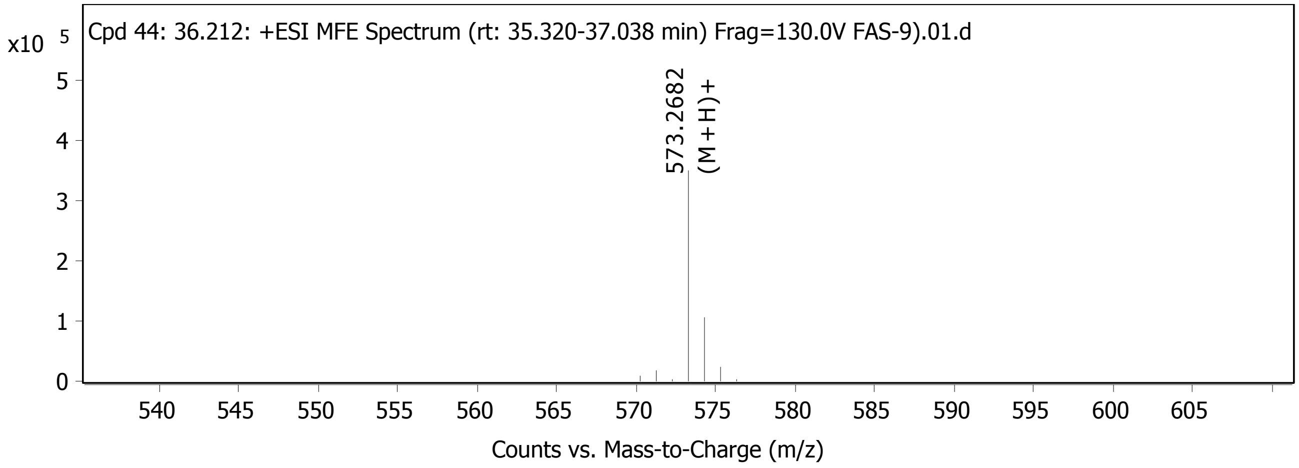

MS Spectrum

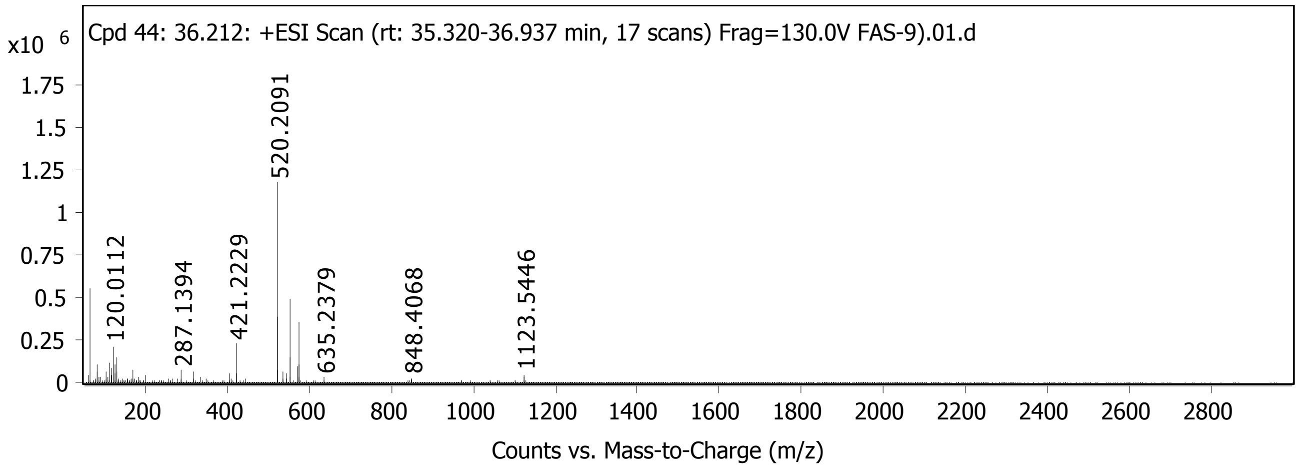

MS Zoomed Spectrum

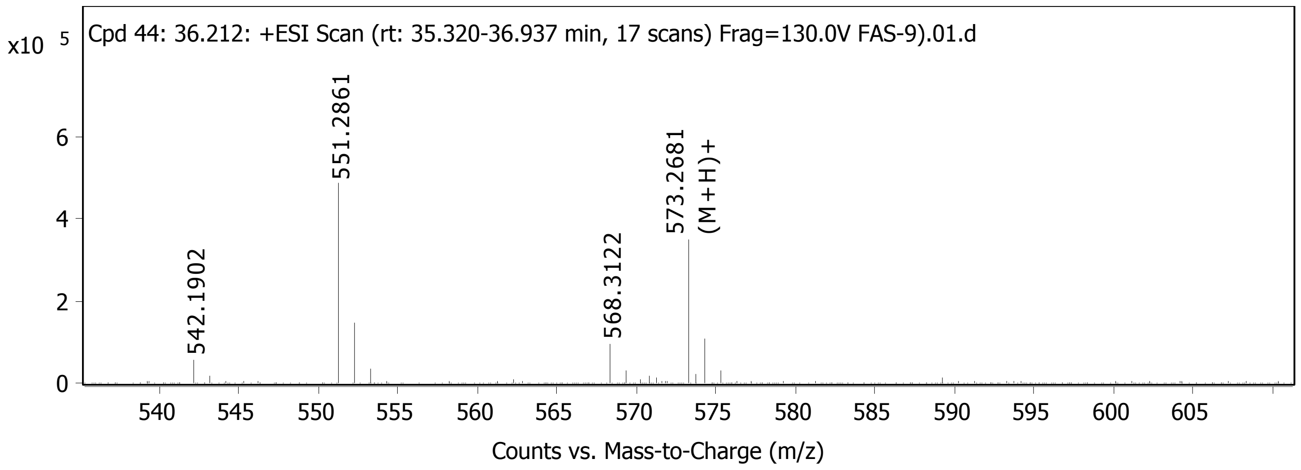

MSMS Spectrum

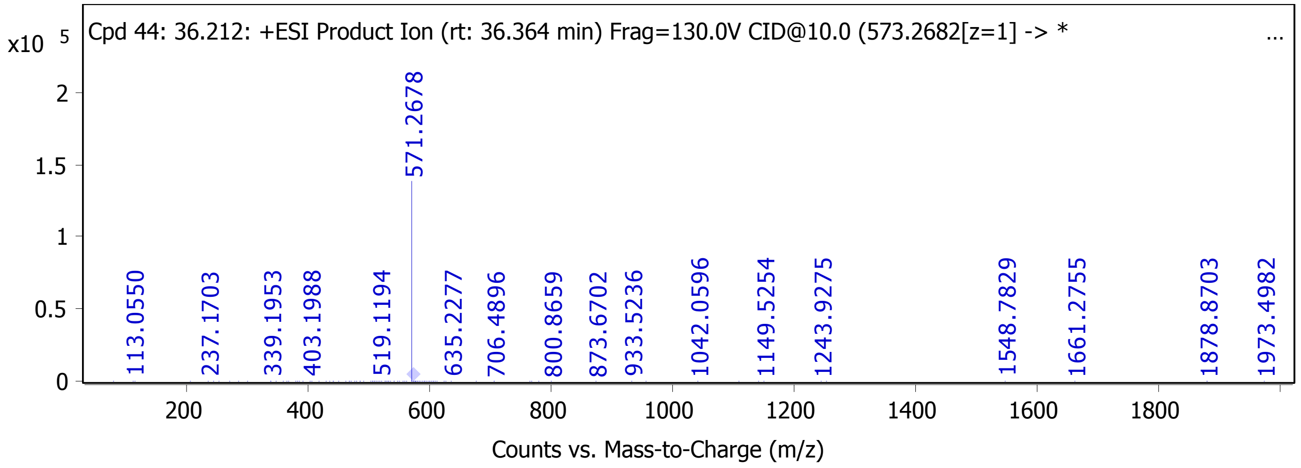

Compound Label	Name	*m/z*	RT	Algorithm	Mass
Cpd 45: Ouabagenin; C23 H34 O8; 36.212	**Ouabagenin**	421.223	36.212	Find by Molecular Feature	438.2262

Compound Chromatograms

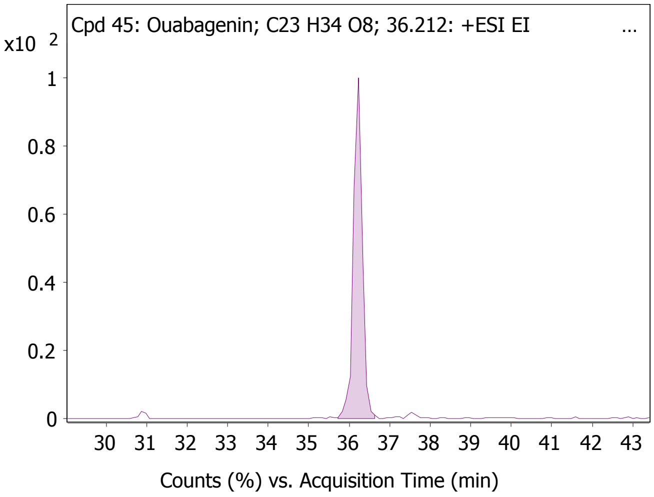

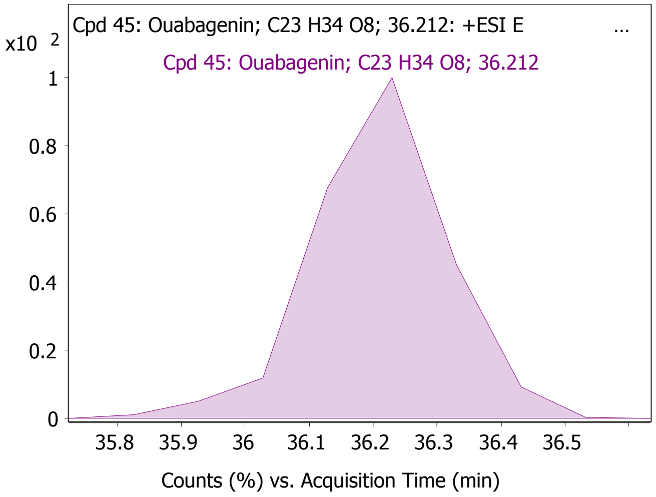

MFE MS Spectrum

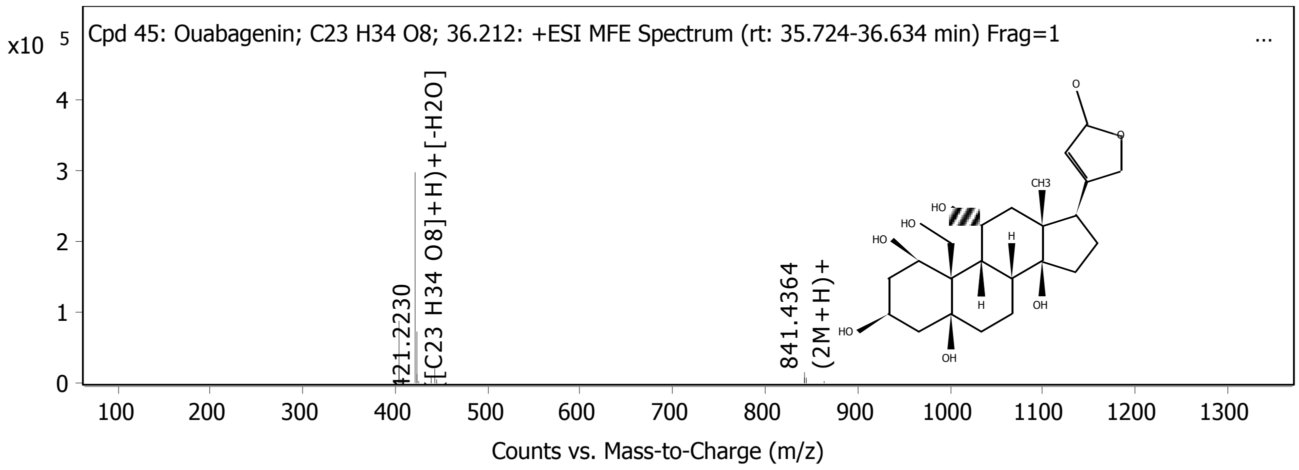

MFE MS Zoomed Spectrum

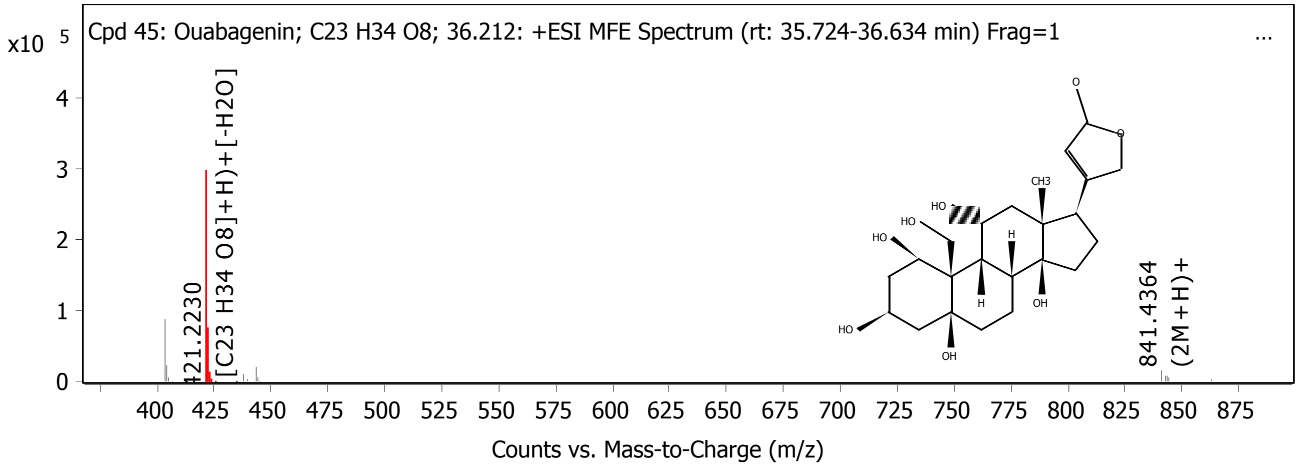

MS Spectrum

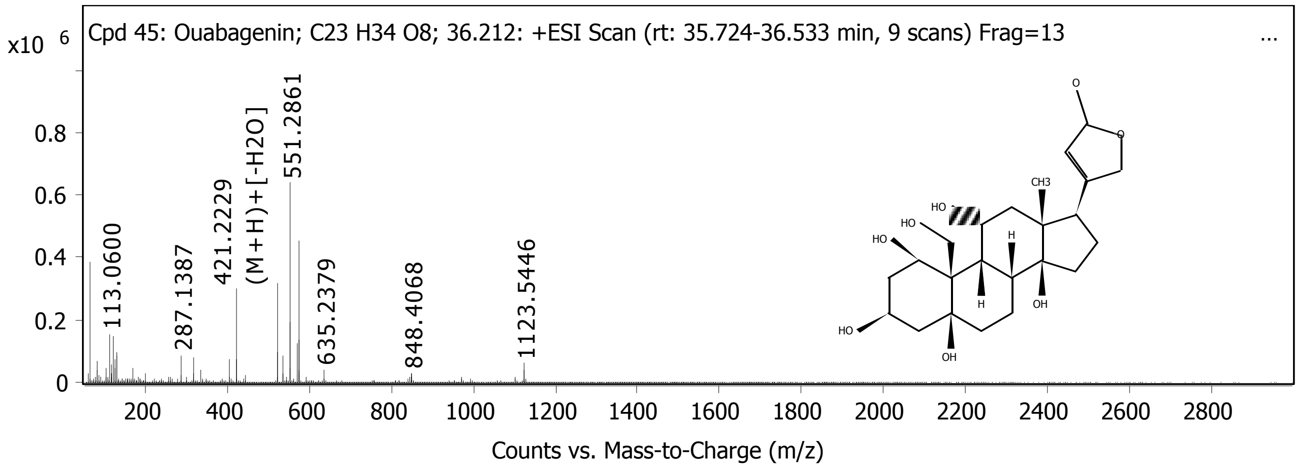

MS Zoomed Spectrum

Agilent Technologies

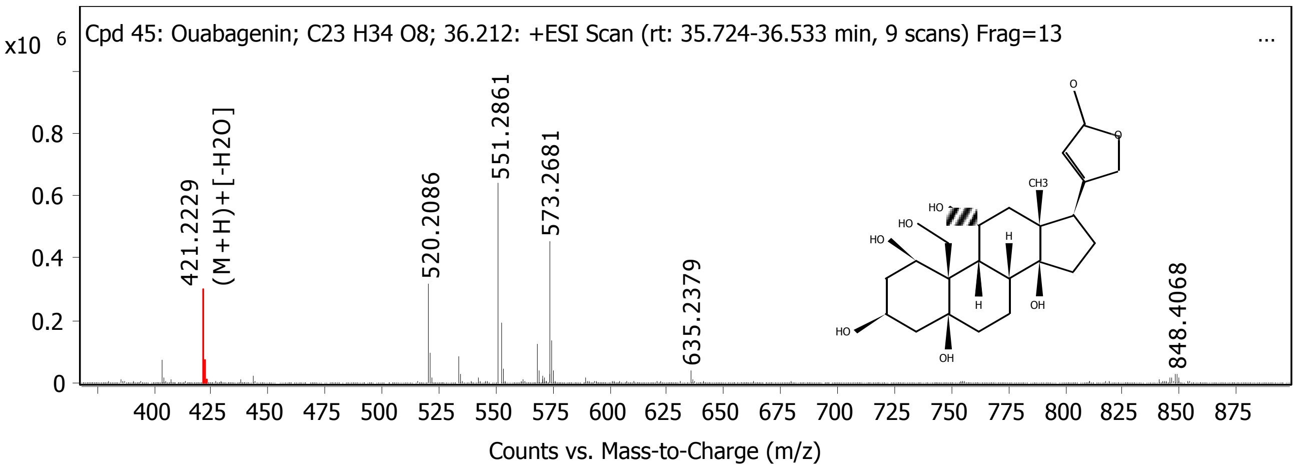

Identification Hit Table

Best Hit	Compound Name	RT	Formula	Notes	Match Score	Mass	Difference	Ion Species
✓	Ouabagenin	36.212	C23 H34 O8	Treatment of cardiac arrhythmias and heart failure Metabolite of Ouabain Dollery, Colin Therapeutic Drugs, 2nd Ed. 1999 p. O32	98.13	438.2262	-0.82	(M+H)+[-H2O]
	Armillane	36.212	C23 H32 O7		98.06	420.2156	-0.82	(M+H)+
	Arg Thr Tyr	36.212	C19 H30 N6 O6		76.16	438.2263	-3.65	(M+H)+[-H2O]
	Thr Tyr Arg	36.212	C19 H30 N6 O6		76.16	438.2263	-3.65	(M+H)+[-H2O]
	Thr Arg Tyr	36.212	C19 H30 N6 O6		76.16	438.2263	-3.65	(M+H)+[-H2O]
	Tyr Arg Thr	36.212	C19 H30 N6 O6		76.16	438.2263	-3.65	(M+H)+[-H2O]
	Tyr Thr Arg	36.212	C19 H30 N6 O6		76.16	438.2263	-3.65	(M+H)+[-H2O]
	Arg Tyr Thr	36.212	C19 H30 N6 O6		76.16	438.2263	-3.65	(M+H)+[-H2O]
	Levocabastine	36.212	C26 H29 F N2 O2		57.52	420.2156	5.66	(M+H)+

Identification Hit Table

Best Hit	Compound Name	RT	Formula	Notes	Match Score	Mass	Difference	Ion Species

Identification Hit Table

Best Hit	Compound Name	RT	Formula	Notes	Match Score	Mass	Difference	Ion Species
✓	Ser Pro Val His	36.212	C19 H30 N6 O6		76.16	438.2263	-3.65	(M+H)+[-H2O]
	His Ser Val Pro	36.212	C19 H30 N6 O6		76.16	438.2263	-3.65	(M+H)+[-H2O]
	Pro His Val Ser	36.212	C19 H30 N6 O6		76.16	438.2263	-3.65	(M+H)+[-H2O]
	Arg Tyr Thr	36.212	C19 H30 N6 O6		76.16	438.2263	-3.65	(M+H)+[-H2O]
	Tyr Thr Arg	36.212	C19 H30 N6 O6		76.16	438.2263	-3.65	(M+H)+[-H2O]
	Tyr Arg Thr	36.212	C19 H30 N6 O6		76.16	438.2263	-3.65	(M+H)+[-H2O]
	Thr Arg Tyr	36.212	C19 H30 N6 O6		76.16	438.2263	-3.65	(M+H)+[-H2O]
	Thr Tyr Arg	36.212	C19 H30 N6 O6		76.16	438.2263	-3.65	(M+H)+[-H2O]
	Arg Thr Tyr	36.212	C19 H30 N6 O6		76.16	438.2263	-3.65	(M+H)+[-H2O]
	His Pro Ser Val	36.212	C19 H30 N6 O6		76.16	438.2263	-3.65	(M+H)+[-H2O]

Compound Label	Name	*m/z*	RT	Algorithm	Mass
Cpd 46: Tyr Tyr Leu Lys; C30 H43 N5 O7; 36.213	**Tyr Tyr Leu Lys**	568.3122	36.213	Find by Molecular Feature	585.3155

Compound Chromatograms

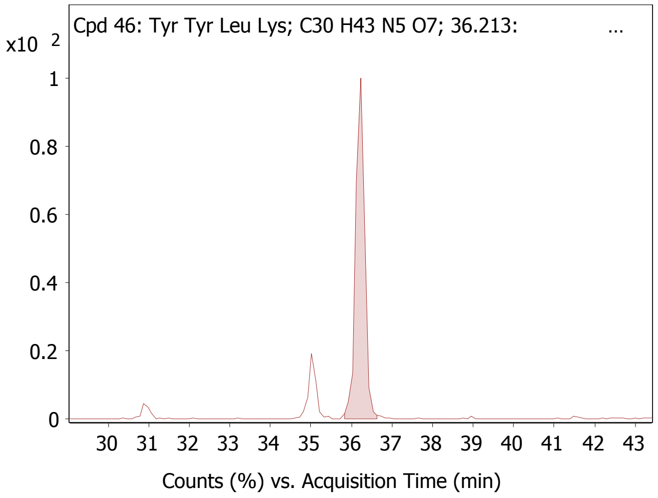

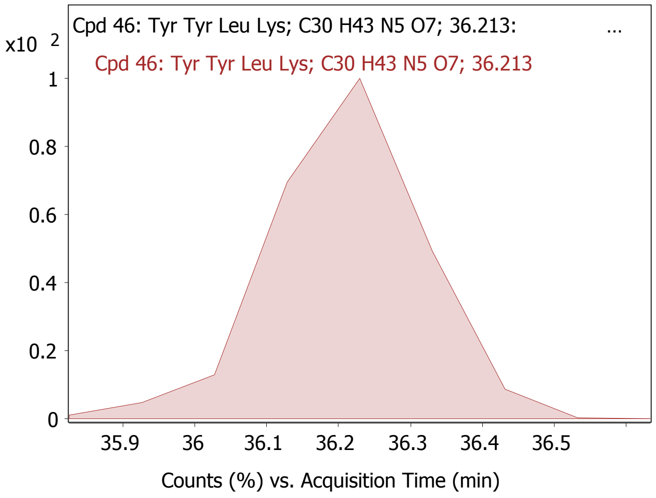

MFE MS Spectrum

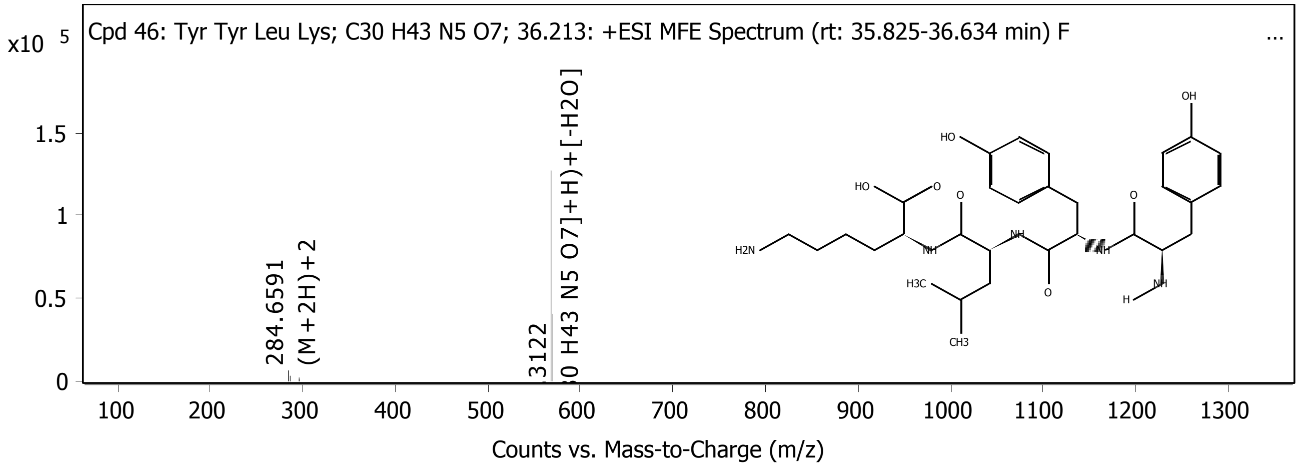

MFE MS Zoomed Spectrum

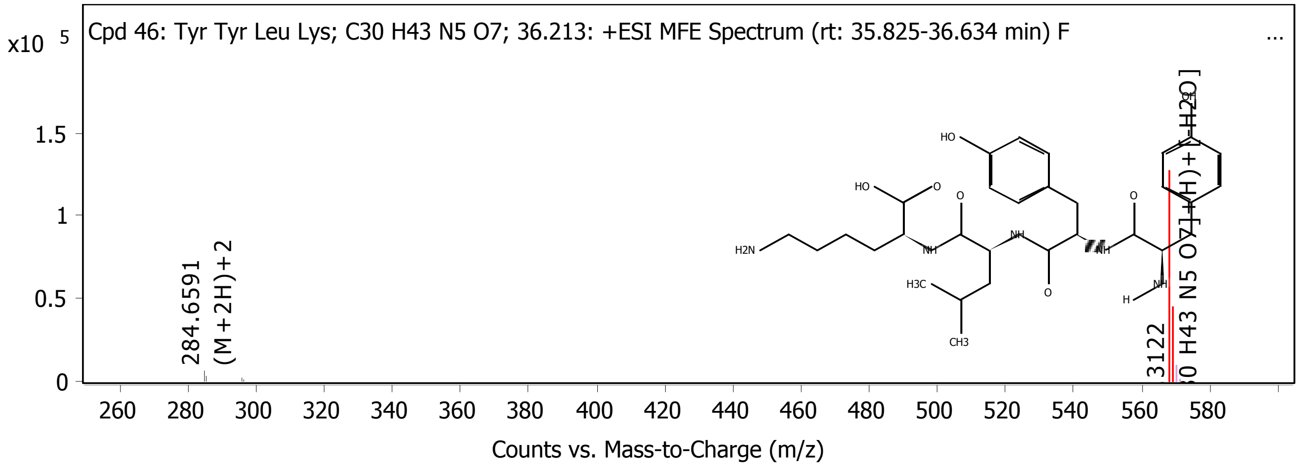

MS Spectrum

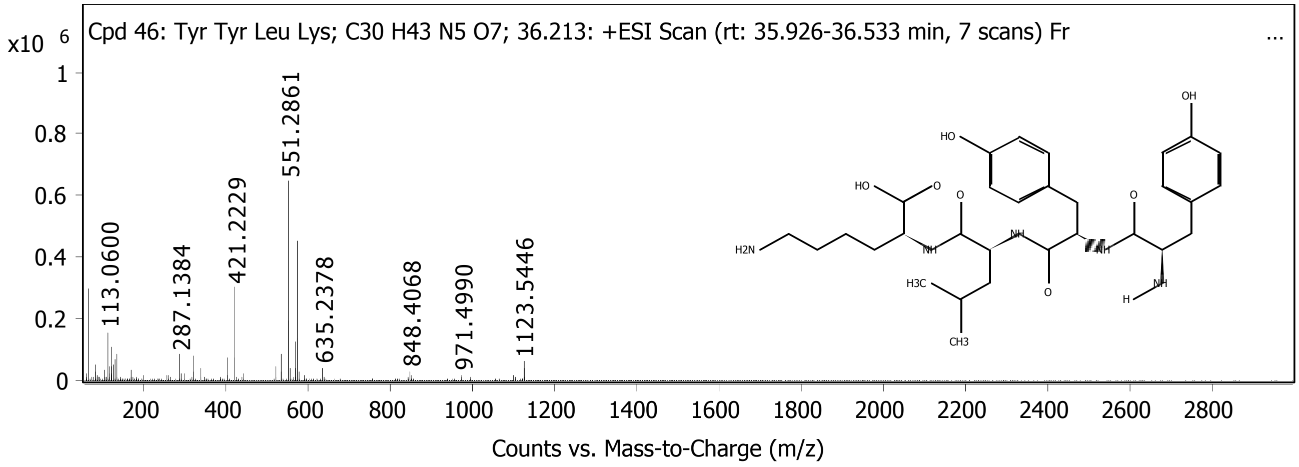

MS Zoomed Spectrum

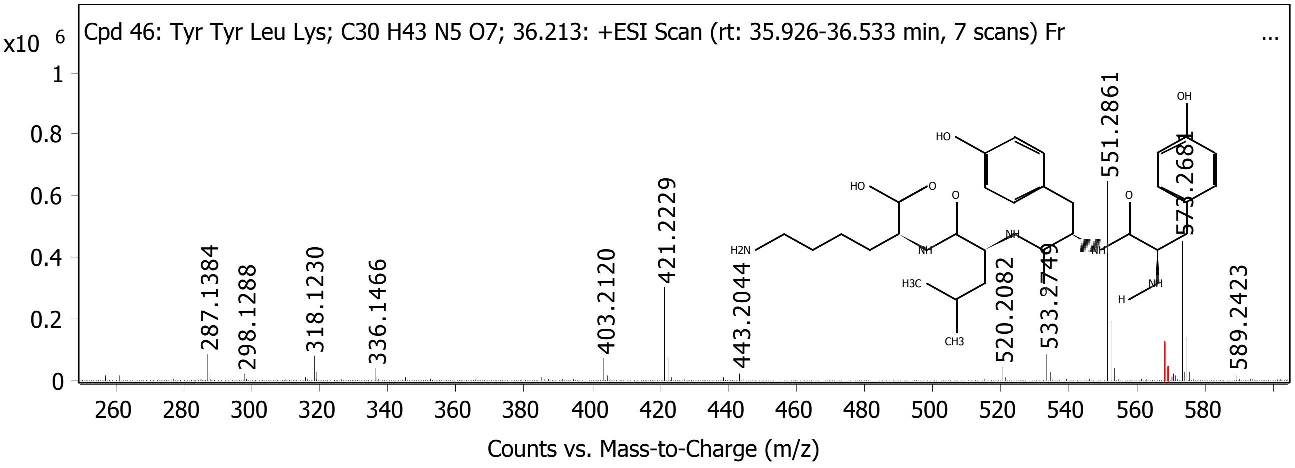

Identification Hit Table

Best Hit	Compound Name	RT	Formula	Notes	Match Score	Mass	Difference	Ion Species
✓	Nelfinavir	36.213	C32 H45 N3 O4 S		24.91	567.305	8.12	(M+H)+

Identification Hit Table

Best Hit	Compound Name	RT	Formula	Notes	Match Score	Mass	Difference	Ion Species

Identification Hit Table

Best Hit	Compound Name	RT	Formula	Notes	Match Score	Mass	Difference	Ion Species
✓	Tyr Tyr Leu Lys	36.213	C30 H43 N5 O7		76.77	585.3155	0.71	(M+H)+[-H2O]
	Tyr Tyr Lys Leu	36.213	C30 H43 N5 O7		76.77	585.3155	0.71	(M+H)+[-H2O]
	Ile Tyr Lys Tyr	36.213	C30 H43 N5 O7		76.77	585.3155	0.71	(M+H)+[-H2O]
	Ile Tyr Tyr Lys	36.213	C30 H43 N5 O7		76.77	585.3155	0.71	(M+H)+[-H2O]
	Lys Ile Tyr Tyr	36.213	C30 H43 N5 O7		76.77	585.3155	0.71	(M+H)+[-H2O]
	Lys Leu Tyr Tyr	36.213	C30 H43 N5 O7		76.77	585.3155	0.71	(M+H)+[-H2O]
	Lys Tyr Ile Tyr	36.213	C30 H43 N5 O7		76.77	585.3155	0.71	(M+H)+[-H2O]
	Lys Tyr Leu Tyr	36.213	C30 H43 N5 O7		76.77	585.3155	0.71	(M+H)+[-H2O]
	Lys Tyr Tyr Ile	36.213	C30 H43 N5 O7		76.77	585.3155	0.71	(M+H)+[-H2O]
	Lys Tyr Tyr Leu	36.213	C30 H43 N5 O7		76.77	585.3155	0.71	(M+H)+[-H2O]

Compound Label	Name	*m/z*	RT	Algorithm	Mass
Cpd 47: 11-alpha-O-beta-D-Glucopyranosyl-16alpha-O-methylneoquassin; C29 H44 O11; 36.215	**11-alpha-O-beta-D-Glucopyranosyl-16alpha-O-methylneoquassin**	551.2862	36.215	Find by Molecular Feature	568.2893

Compound Chromatograms

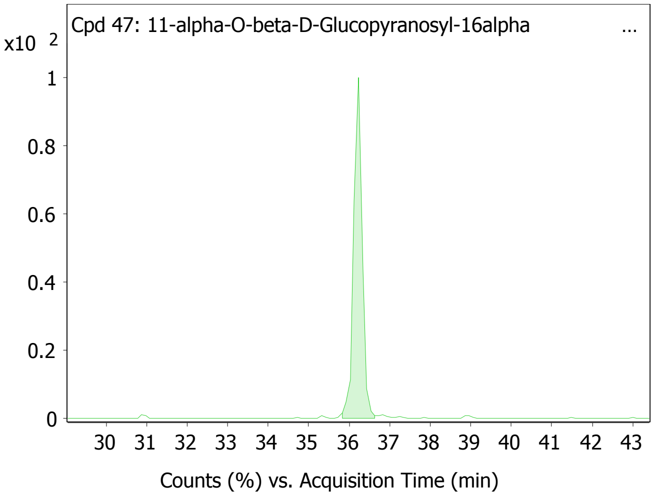

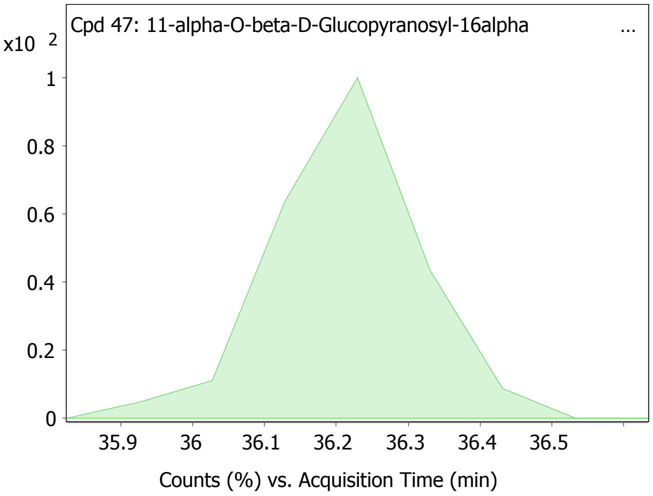

MFE MS Spectrum

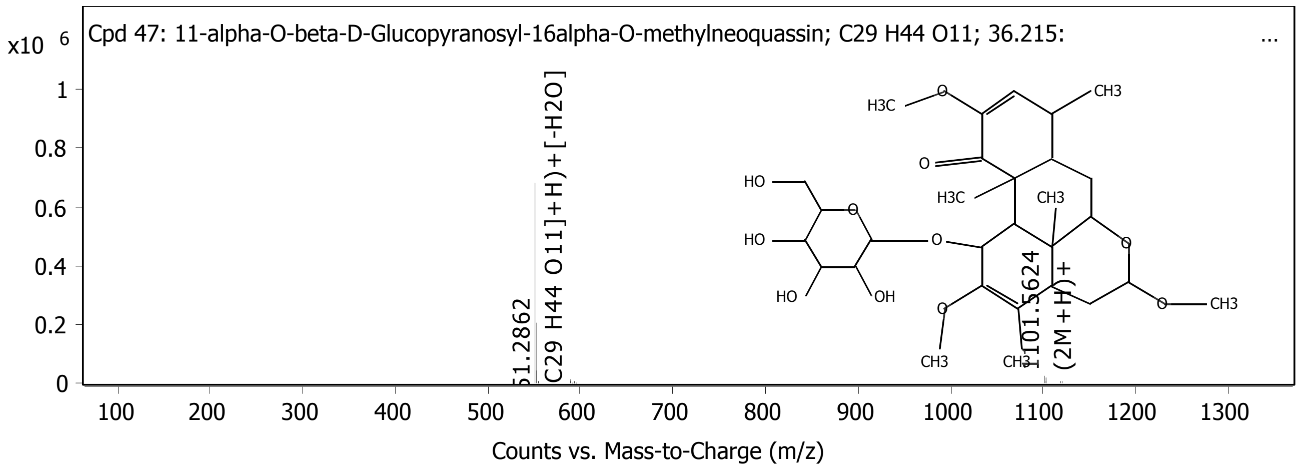

MFE MS Zoomed Spectrum

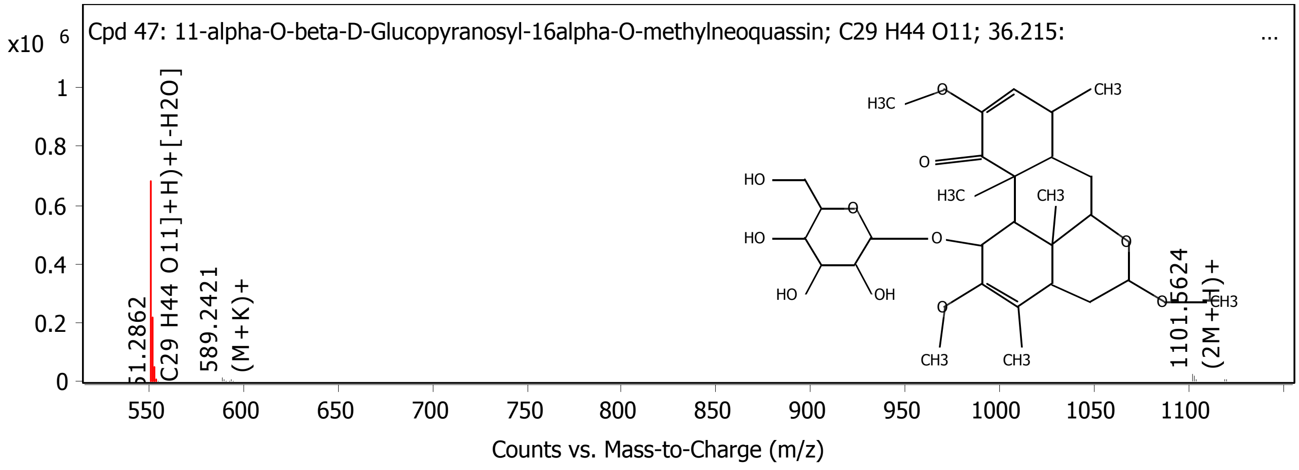

MS Spectrum

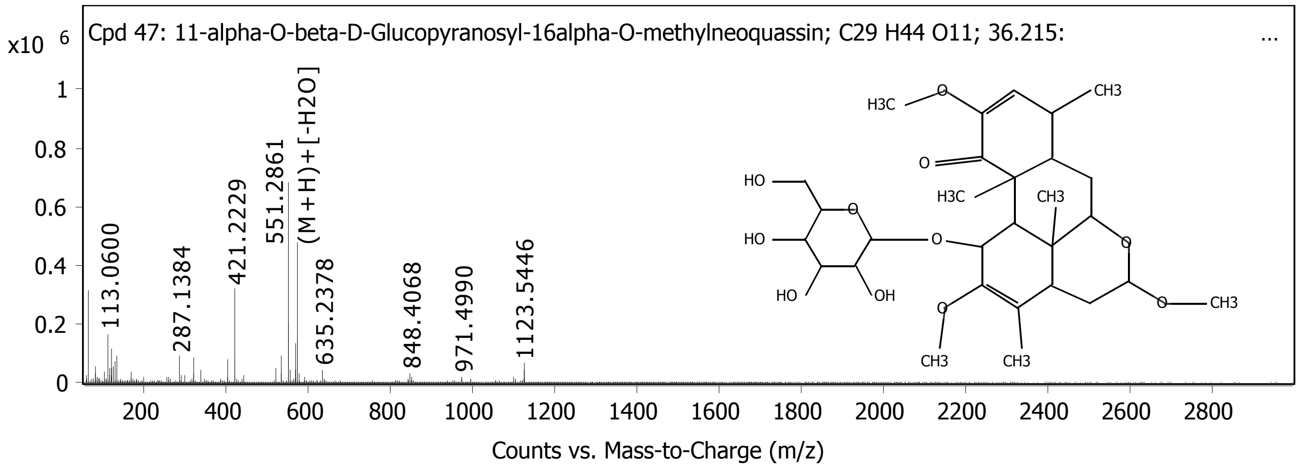

MS Zoomed Spectrum

Qualitative Compound Identification Report

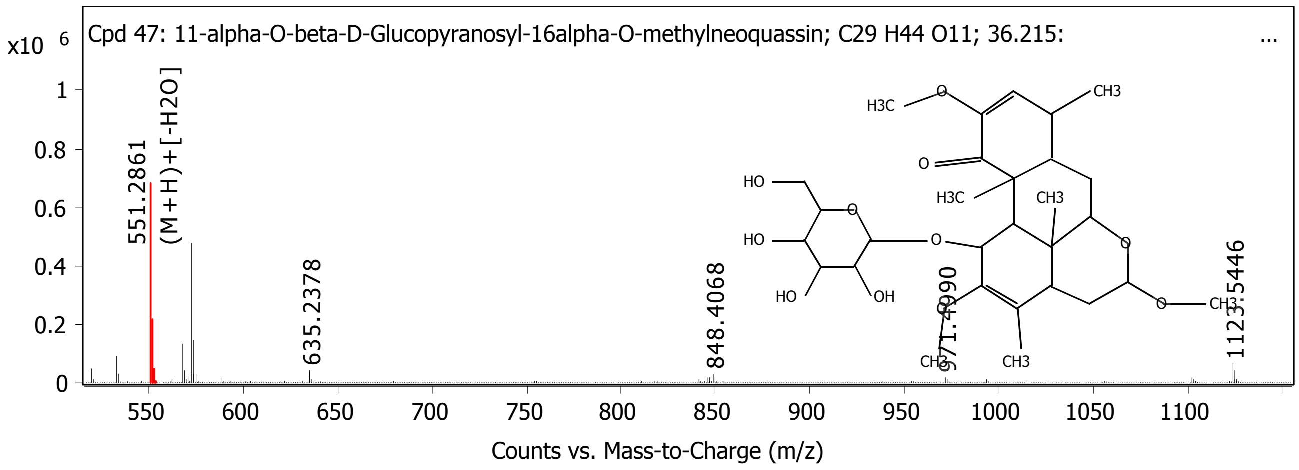

Identification Hit Table

Best Hit	Compound Name	RT	Formula	Notes	Match Score	Mass	Difference	Ion Species
✓	11-alpha-O-beta-D-Glucopyranosyl-16alpha-O-methylneoquassin	36.215	C29 H44 O11		97.51	568.2893	-0.98	(M+H)+[-H2O]
	Canesceol	36.215	C29 H44 O11		97.51	568.2893	-0.98	(M+H)+[-H2O]
	Sarmentoloside	36.215	C29 H44 O11		97.51	568.2893	-0.98	(M+H)+[-H2O]
	Aspecioside	36.215	C29 H42 O10		97.45	550.2788	-0.98	(M+H)+
	adonitoxin	36.215	C29 H42 O10		97.45	550.2788	-0.98	(M+H)+
	Convallatoxin	36.215	C29 H42 O10	Convallaria majalis	97.45	550.2788	-0.98	(M+H)+
	Leukotriene F4	36.215	C28 H44 N2 O8 S	Endogenous Metabolite http://dbk.ch.umist.ac.uk/ExactMasses.htm	51.96	568.2897	-7.82	(M+H)+[-H2O]

Identification Hit Table

Best Hit	Compound Name	RT	Formula	Notes	Match Score	Mass	Difference	Ion Species
✓	adonitoxin	36.215	C29 H42 O10		97.45	550.2788	-0.98	(M+H)+
	Convallatoxin	36.215	C29 H42 O10	Convallaria majalis	97.45	550.2788	-0.98	(M+H)+
	Leukotriene F4	36.215	C28 H44 N2 O8 S	Endogenous Metabolite http://dbk.ch.umist.ac.uk/ExactMasses.htm	51.96	568.2897	-7.82	(M+H)+[-H2O]

Identification Hit Table

Best Hit	Compound Name	RT	Formula	Notes	Match Score	Mass	Difference	Ion Species
✓	Ala His Arg Trp	36.215	C26 H36 N10 O5		88.61	568.2896	-2.54	(M+H)+[-H2O]
	His Trp Ala Arg	36.215	C26 H36 N10 O5		88.61	568.2896	-2.54	(M+H)+[-H2O]
	His Trp Arg Ala	36.215	C26 H36 N10 O5		88.61	568.2896	-2.54	(M+H)+[-H2O]
	Ala His Trp Arg	36.215	C26 H36 N10 O5		88.61	568.2896	-2.54	(M+H)+[-H2O]
	Ala Arg His Trp	36.215	C26 H36 N10 O5		88.61	568.2896	-2.54	(M+H)+[-H2O]
	Ala Arg Trp His	36.215	C26 H36 N10 O5		88.61	568.2896	-2.54	(M+H)+[-H2O]
	Ala Trp His Arg	36.215	C26 H36 N10 O5		88.61	568.2896	-2.54	(M+H)+[-H2O]
	Ala Trp Arg His	36.215	C26 H36 N10 O5		88.61	568.2896	-2.54	(M+H)+[-H2O]
	His Ala Arg Trp	36.215	C26 H36 N10 O5		88.61	568.2896	-2.54	(M+H)+[-H2O]
	Trp Arg His Ala	36.215	C26 H36 N10 O5		88.61	568.2896	-2.54	(M+H)+[-H2O]

Compound Label	Name	*m/z*	RT	Algorithm	Mass
Cpd 48: Methyl levulinate; C6 H10 O3; 36.216	**Methyl levulinate**	113.0601	36.216	Find by Molecular Feature	130.0634

Compound Chromatograms

Qualitative Compound Identification Report

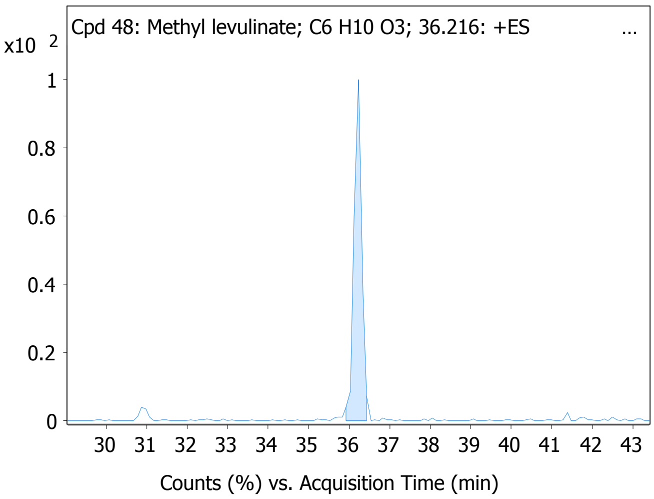

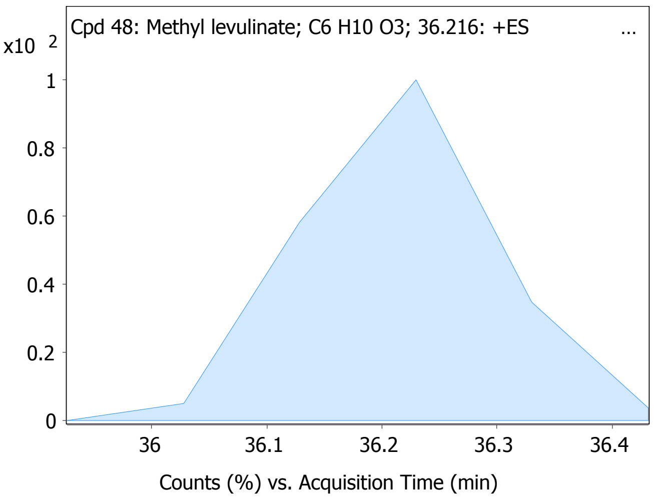

MFE MS Spectrum

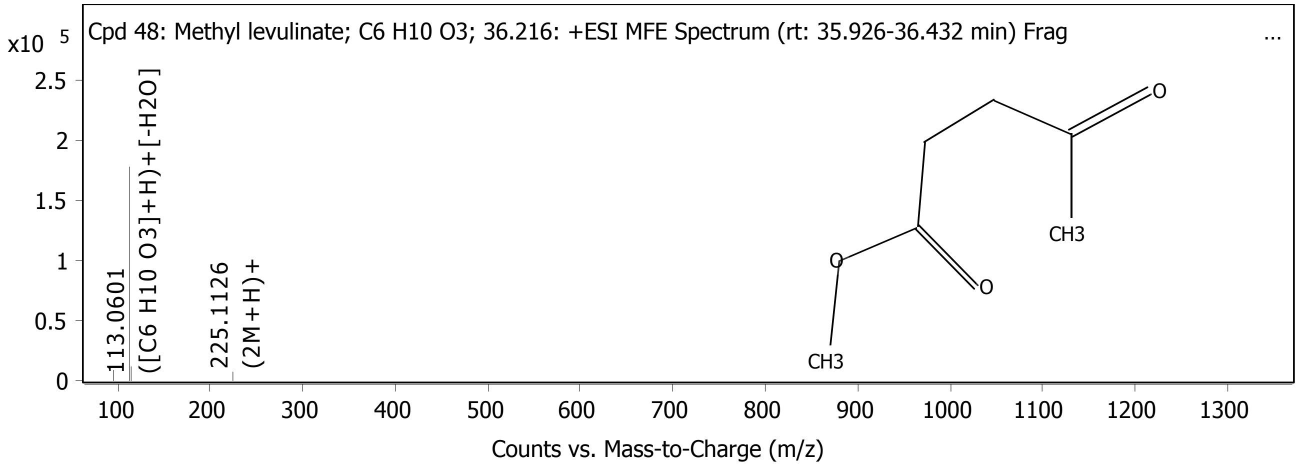

MFE MS Zoomed Spectrum

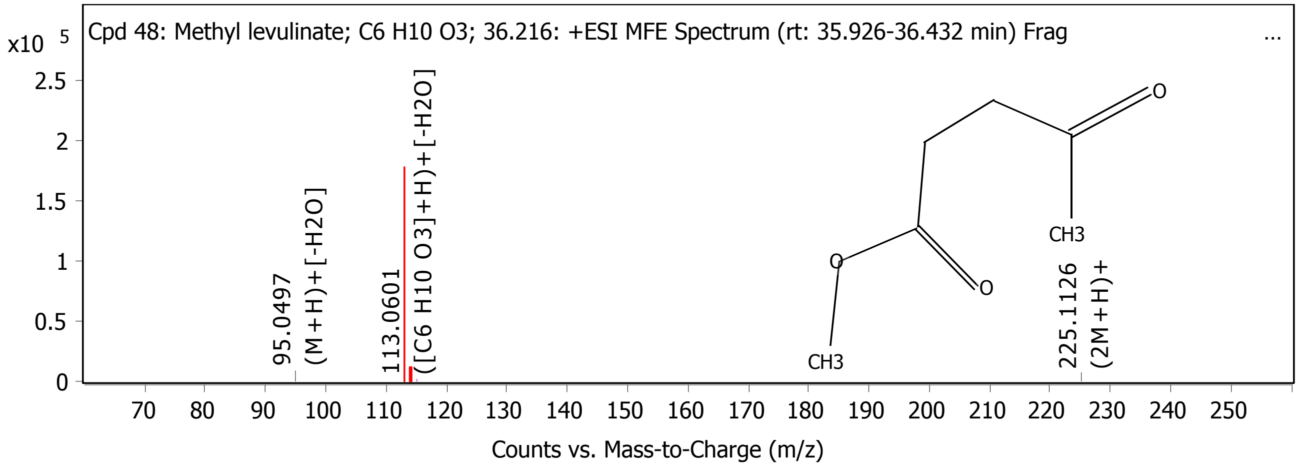

MS Spectrum

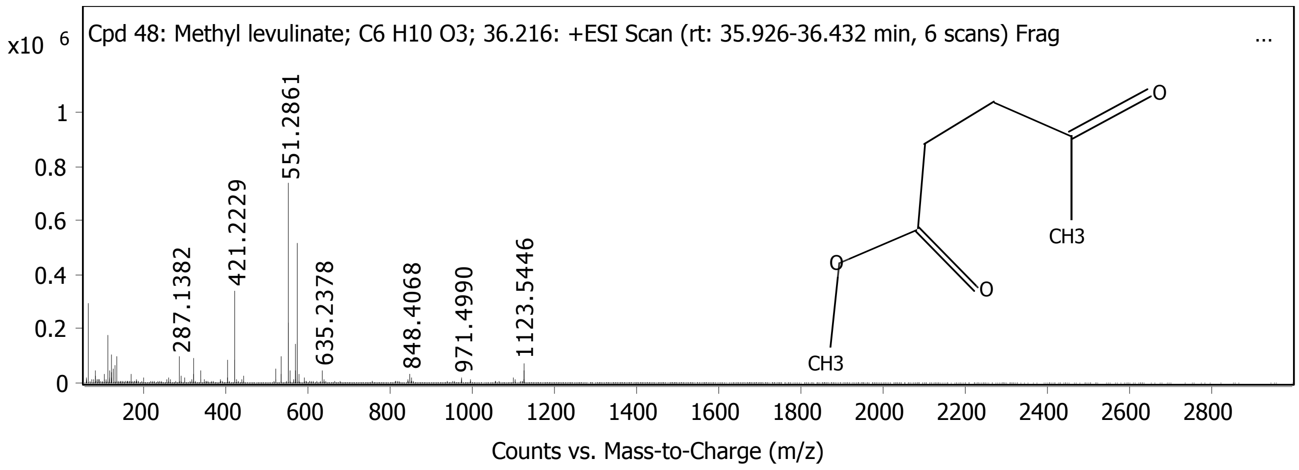

MS Zoomed Spectrum

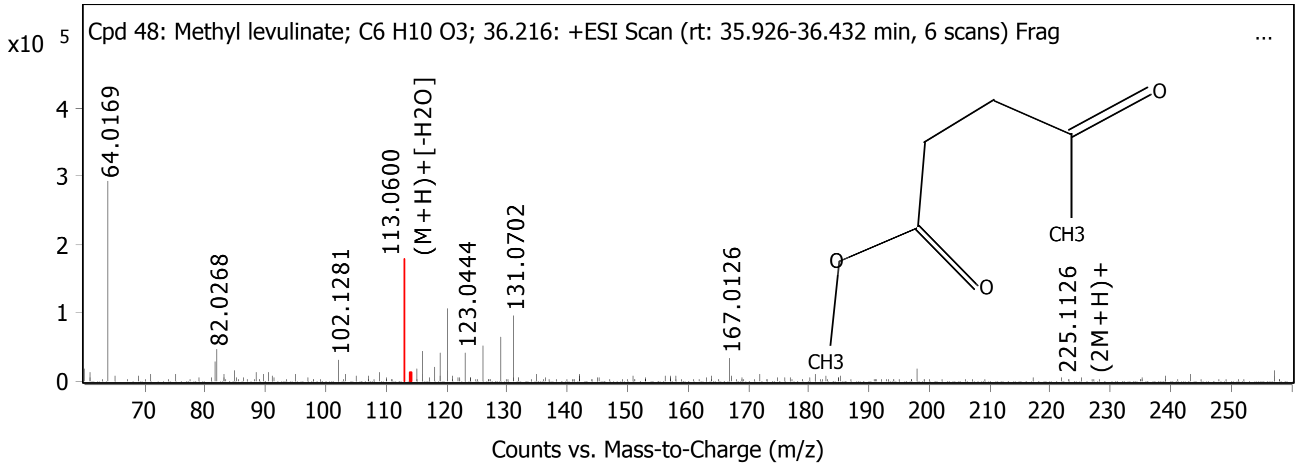

Identification Hit Table

Best Hit	Compound Name	RT	Formula	Notes	Match Score	Mass	Difference	Ion Species
✓	Methyl levulinate	36.216	C6 H10 O3		87.11	130.0634	-0.36	(M+H)+[-H2O]
	5-keto-n-caproic acid	36.216	C6 H10 O3		87.11	130.0634	-0.36	(M+H)+[-H2O]
	Acetoin acetate	36.216	C6 H10 O3		87.11	130.0634	-0.36	(M+H)+[-H2O]
	2-Ketohexanoic acid	36.216	C6 H10 O3	á-Ketocaproic acid, 2-Ketocaproic acid	87.11	130.0634	-0.36	(M+H)+[-H2O]
	MVSL / ±-Mevalonolactone	36.216	C6 H10 O3	Deleted CAS: 503-48-0	87.11	130.0634	-0.36	(M+H)+[-H2O]
	Ketoleucine	36.216	C6 H10 O3	Geigy vol.3 p.109 Na Salt CAS: 4502-00-5	87.11	130.0634	-0.36	(M+H)+[-H2O]
	2-Methyl-3-ketovaleric acid	36.216	C6 H10 O3		87.11	130.0634	-0.36	(M+H)+[-H2O]
	4,5-Dihydroxyhexanoic acid lactone	36.216	C6 H10 O3		87.11	130.0634	-0.36	(M+H)+[-H2O]
	(R)-3-Methyl-2-oxo-pentanoic acid	36.216	C6 H10 O3	2oxo-3R-methyl-pentanoic acid	87.11	130.0634	-0.36	(M+H)+[-H2O]
	3-Oxo-4-Methyl-pentanoic acid	36.216	C6 H10 O3		87.11	130.0634	-0.36	(M+H)+[-H2O]

Identification Hit Table

Best Hit	Compound Name	RT	Formula	Notes	Match Score	Mass	Difference	Ion Species
✓	3-Methyl-2-oxovaleric acid	36.216	C6 H10 O3	Geigy vol.3 p.109	87.11	130.0634	-0.36	(M+H)+[-H2O]
	(R)-3-Methyl-2-oxo-pentanoic acid	36.216	C6 H10 O3	2oxo-3R-methyl-pentanoic acid	87.11	130.0634	-0.36	(M+H)+[-H2O]
	2-keto-n-caproic acid	36.216	C6 H10 O3		87.11	130.0634	-0.36	(M+H)+[-H2O]
	3-keto-n-caproic acid	36.216	C6 H10 O3		87.11	130.0634	-0.36	(M+H)+[-H2O]
	4-keto-n-caproic acid	36.216	C6 H10 O3		87.11	130.0634	-0.36	(M+H)+[-H2O]
	5-keto-n-caproic acid	36.216	C6 H10 O3		87.11	130.0634	-0.36	(M+H)+[-H2O]
	MVSL / ±-Mevalonolactone	36.216	C6 H10 O3	Deleted CAS: 503-48-0	87.11	130.0634	-0.36	(M+H)+[-H2O]
	3-Oxo-4-Methyl-pentanoic acid	36.216	C6 H10 O3		87.11	130.0634	-0.36	(M+H)+[-H2O]
	Aleprolic acid	36.216	C6 H8 O2		87.04	112.0528	-0.36	(M+H)+
	4-oxo-2E-Hexenal	36.216	C6 H8 O2		87.04	112.0528	-0.36	(M+H)+

Identification Hit Table

Best Hit	Compound Name	RT	Formula	Notes	Match Score	Mass	Difference	Ion Species

Compound Label	Name	*m/z*	RT	Algorithm	Mass
Cpd 49: Ginsenoside Rh8; C36 H60 O9; 44.110	**Ginsenoside Rh8**	310.2122	44.11	Find by Molecular Feature	636.4204

Compound Chromatograms

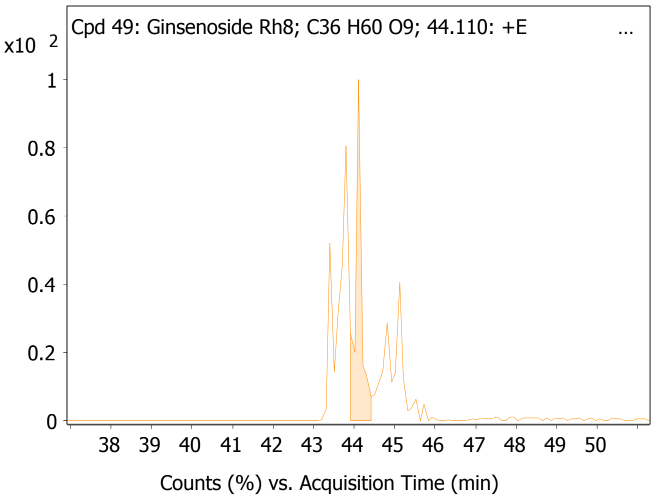

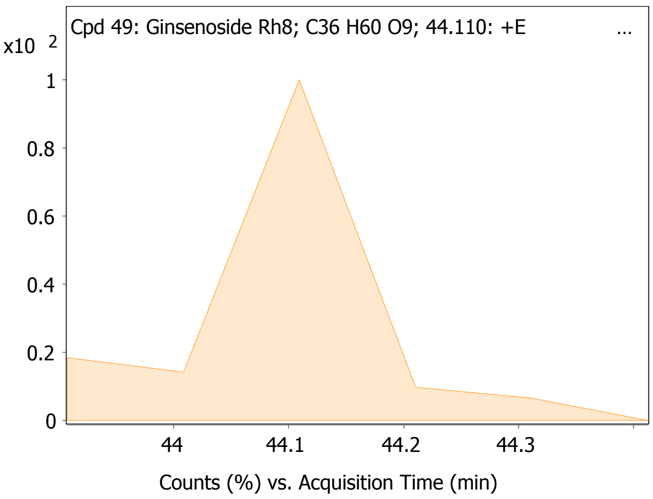

MFE MS Spectrum

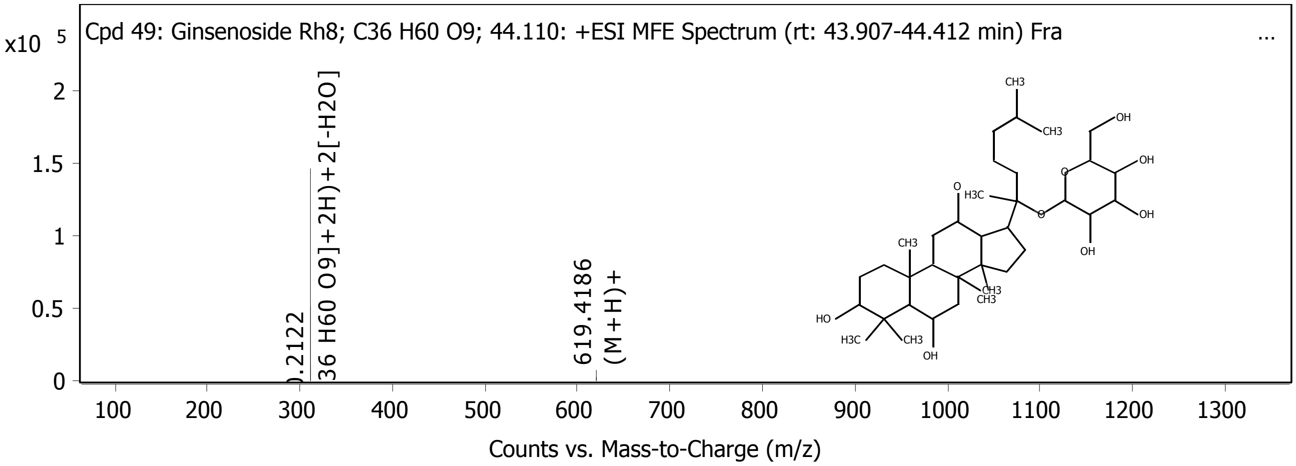

MFE MS Zoomed Spectrum

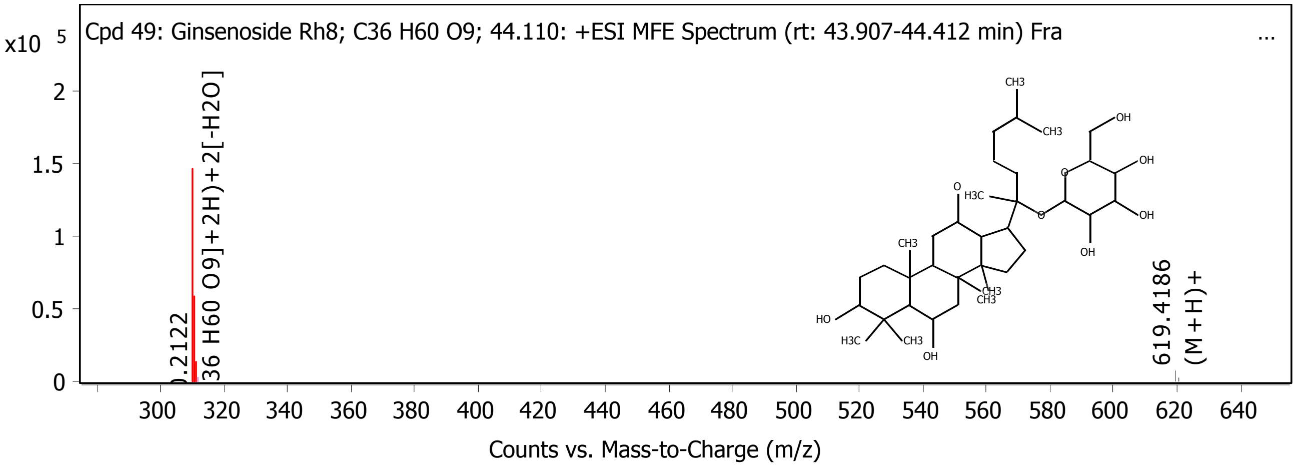

MS Spectrum

Qualitative Compound Identification Report

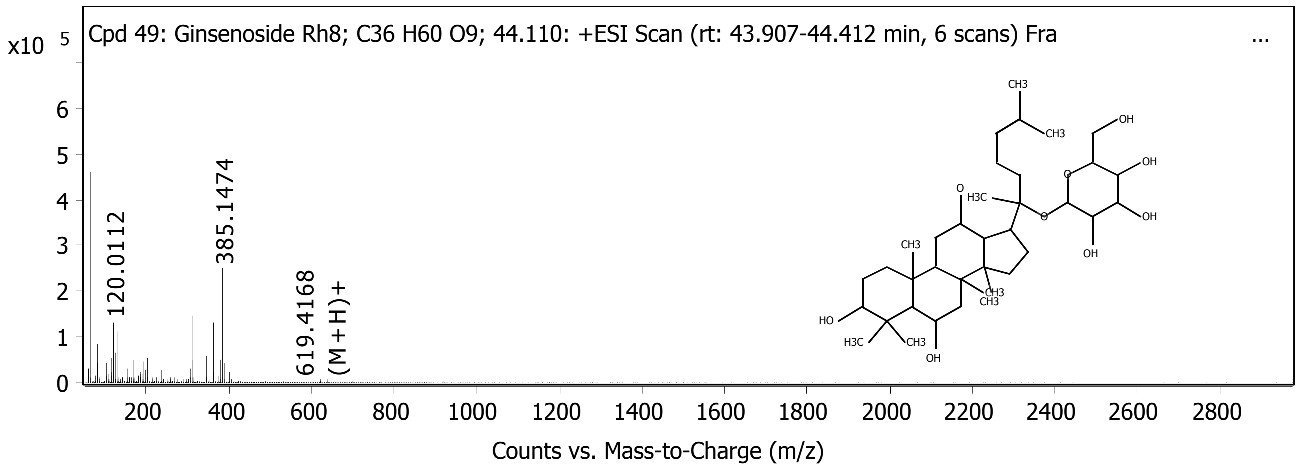

MS Zoomed Spectrum

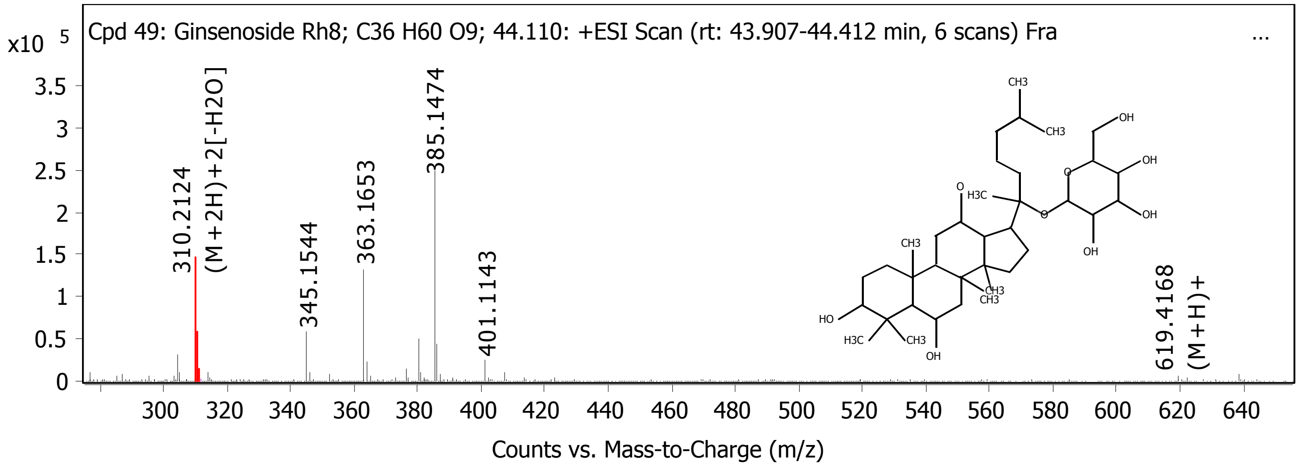

MSMS Spectrum

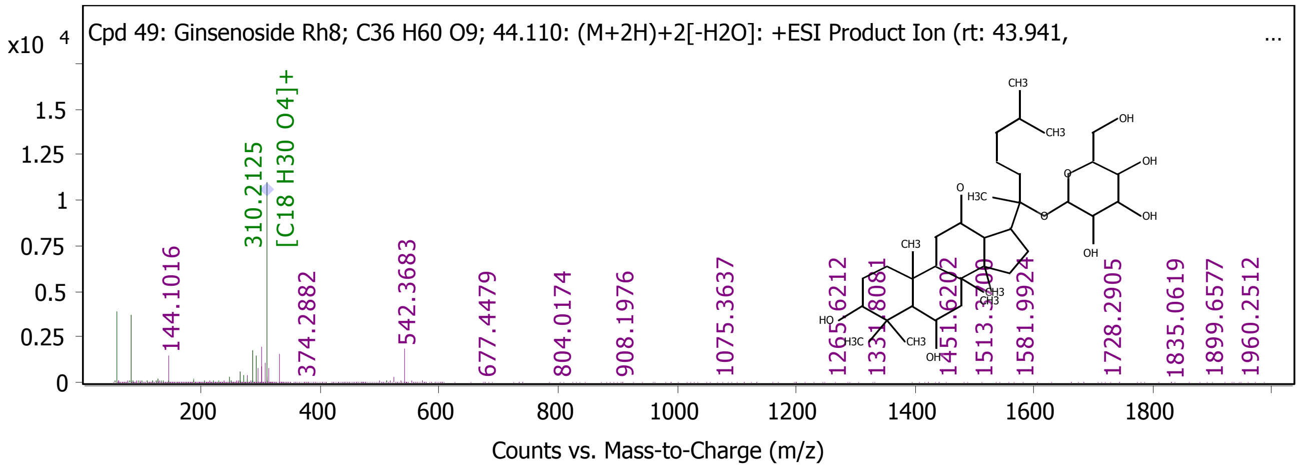

Identification Hit Table

Best Hit	Compound Name	RT	Formula	Notes	Match Score	Mass	Difference	Ion Species
✓	Ginsenoside Rh8	44.11	C36 H60 O9		82.87	636.4204	3.31	(M+2H)+2[-H2O]
	Ginsenoside Rh7	44.11	C36 H60 O9		82.87	636.4204	3.31	(M+2H)+2[-H2O]
	Fasciculic acid B	44.11	C36 H60 O9		82.87	636.4204	3.31	(M+2H)+2[-H2O]
	Momordicoside I	44.11	C36 H58 O8		82.44	618.4099	3.31	(M+2H)+2
	Lansioside B	44.11	C36 H58 O8		82.44	618.4099	3.31	(M+2H)+2
	19-Methoxypomolic acid 3-arabinoside	44.11	C36 H58 O8		82.44	618.4099	3.31	(M+2H)+2

Identification Hit Table

Best Hit	Compound Name	RT	Formula	Notes	Match Score	Mass	Difference	Ion Species

Identification Hit Table

Best Hit	Compound Name	RT	Formula	Notes	Match Score	Mass	Difference	Ion Species

Agilent Technologies

Qualitative Compound Identification Report

Compound Label	Name	*m/z*	RT	Algorithm	Mass
Cpd 50: Prenyl arabinosyl-(1->6)-glucoside; C16 H28 O10; 44.196	**Prenyl arabinosyl-(1->6)-glucoside**	385.1474	44.196	Find by Molecular Feature	380.1687

Compound Chromatograms

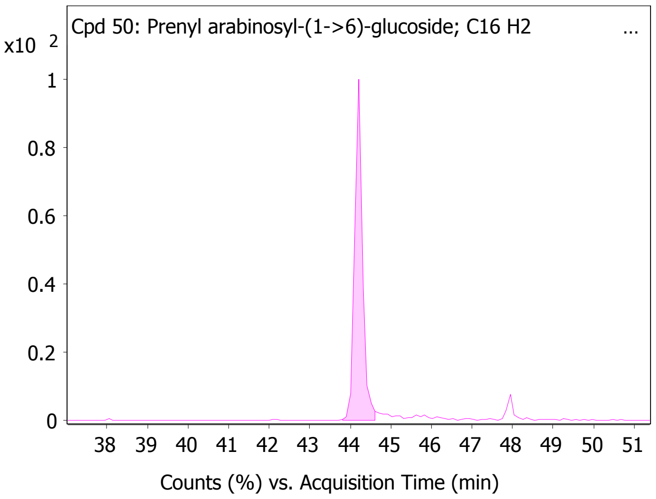

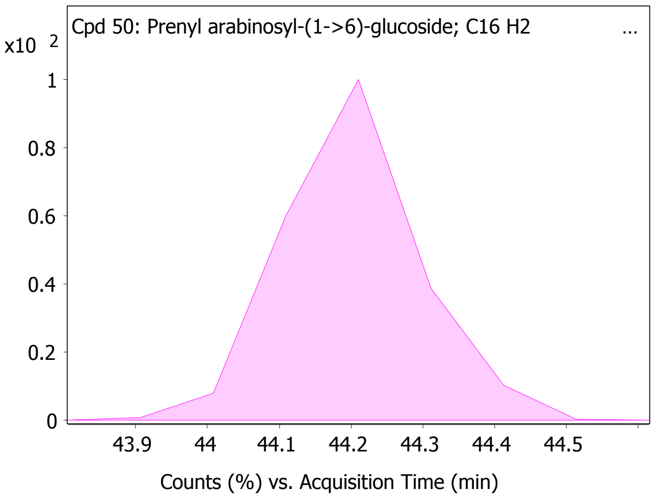

MFE MS Spectrum

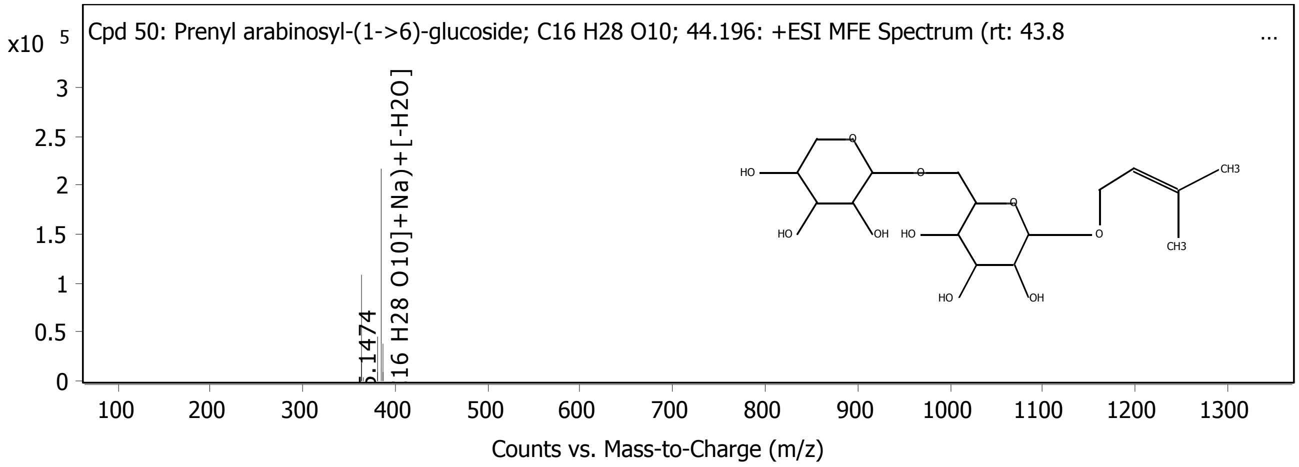

MFE MS Zoomed Spectrum

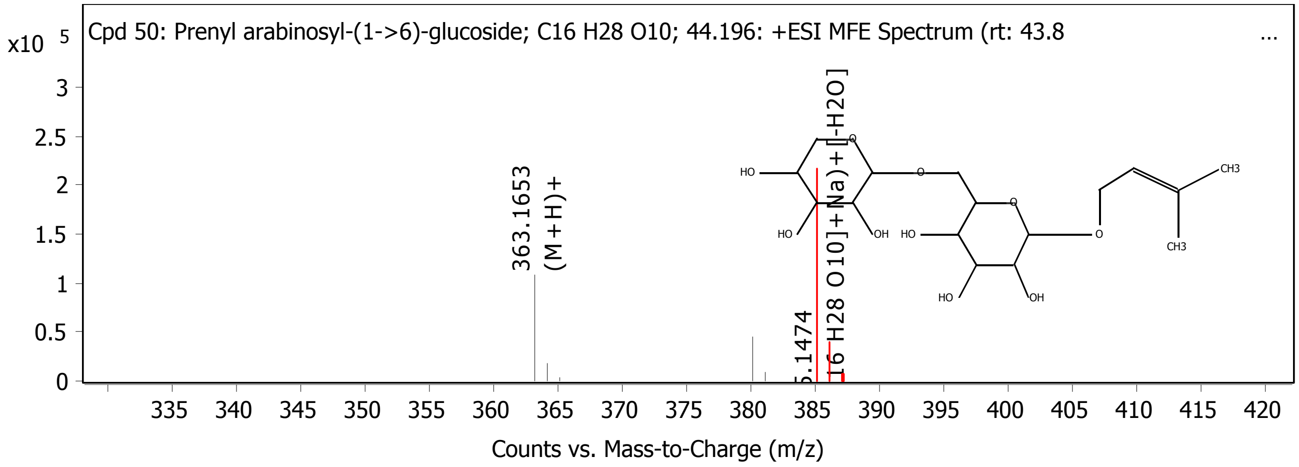

Qualitative Compound Identification Report

MS Spectrum

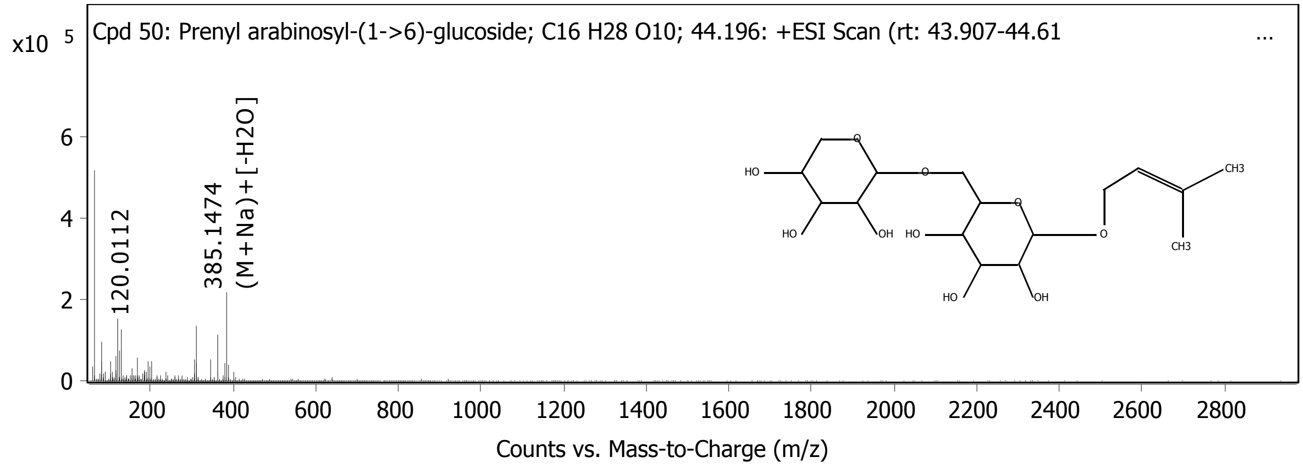

MS Zoomed Spectrum

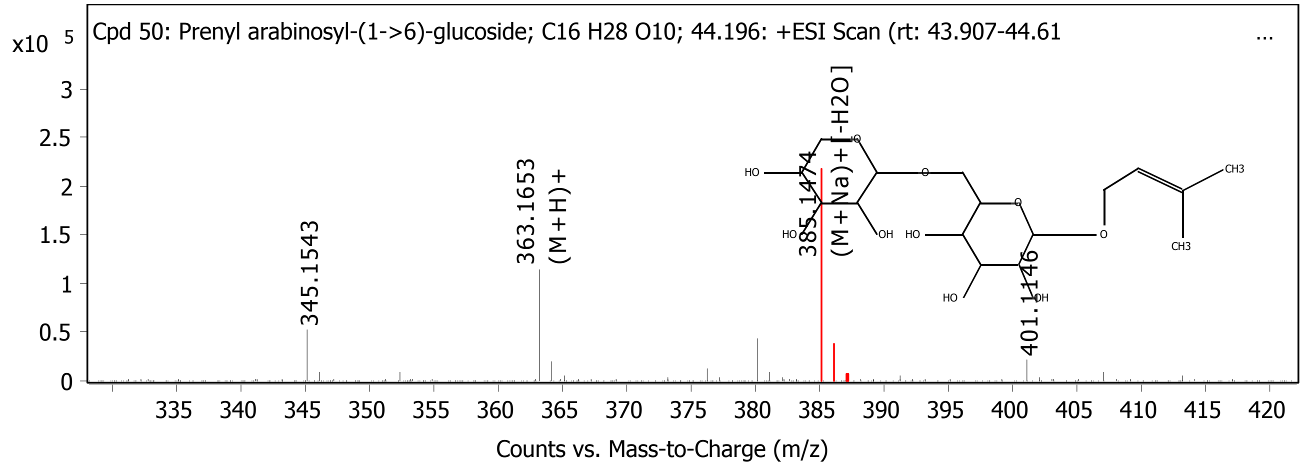

Identification Hit Table

Best Hit	Compound Name	RT	Formula	Notes	Match Score	Mass	Difference	Ion Species
✓	Prenyl arabinosyl-(1->6)-glucoside	44.196	C16 H28 O10		99.16	380.1687	-0.48	(M+Na)+[-H2O]
	Prenyl apiosyl-(1->6)-glucoside	44.196	C16 H28 O10		99.16	380.1687	-0.48	(M+Na)+[-H2O]
	3-Methyl-3-butenyl apiosyl-(1->6)-glucoside	44.196	C16 H28 O10		99.16	380.1687	-0.48	(M+Na)+[-H2O]
	Gln Ala Tyr	44.196	C17 H24 N4 O6		96.4	380.1688	0.78	(M+Na)+[-H2O]
	Gln Phe Ser	44.196	C17 H24 N4 O6		96.4	380.1688	0.78	(M+Na)+[-H2O]
	Ser Gln Phe	44.196	C17 H24 N4 O6		96.4	380.1688	0.78	(M+Na)+[-H2O]
	Phe Asn Thr	44.196	C17 H24 N4 O6		96.4	380.1688	0.78	(M+Na)+[-H2O]
	Phe Gln Ser	44.196	C17 H24 N4 O6		96.4	380.1688	0.78	(M+Na)+[-H2O]
	Ala Tyr Gln	44.196	C17 H24 N4 O6		96.4	380.1688	0.78	(M+Na)+[-H2O]
	Thr Asn Phe	44.196	C17 H24 N4 O6		96.4	380.1688	0.78	(M+Na)+[-H2O]

Identification Hit Table

Best Hit	Compound Name	RT	Formula	Notes	Match Score	Mass	Difference	Ion Species

Identification Hit Table

Best Hit	Compound Name	RT	Formula	Notes	Match Score	Mass	Difference	Ion Species
✓	Asn Thr Phe	44.196	C17 H24 N4 O6		96.4	380.1688	0.78	(M+Na)+[-H2O]
	Ala Phe Gly Ser	44.196	C17 H24 N4 O6		96.4	380.1688	0.78	(M+Na)+[-H2O]
	Ser Phe Gln	44.196	C17 H24 N4 O6		96.4	380.1688	0.78	(M+Na)+[-H2O]
	Ala Tyr Gly Ala	44.196	C17 H24 N4 O6		96.4	380.1688	0.78	(M+Na)+[-H2O]
	Ala Tyr Ala Gly	44.196	C17 H24 N4 O6		96.4	380.1688	0.78	(M+Na)+[-H2O]
	Ala Ser Gly Phe	44.196	C17 H24 N4 O6		96.4	380.1688	0.78	(M+Na)+[-H2O]
	Ala Ser Phe Gly	44.196	C17 H24 N4 O6		96.4	380.1688	0.78	(M+Na)+[-H2O]
	Ala Gly Tyr Ala	44.196	C17 H24 N4 O6		96.4	380.1688	0.78	(M+Na)+[-H2O]
	Ala Gly Ser Phe	44.196	C17 H24 N4 O6		96.4	380.1688	0.78	(M+Na)+[-H2O]
	Ala Gly Phe Ser	44.196	C17 H24 N4 O6		96.4	380.1688	0.78	(M+Na)+[-H2O]

Compound Label	Name	*m/z*	RT	Algorithm	Mass
Cpd 51: Ginsenoside Rh8; C36 H60 O9; 45.110	**Ginsenoside Rh8**	310.2122	45.11	Find by Molecular Feature	636.4284

Compound Chromatograms

Qualitative Compound Identification Report

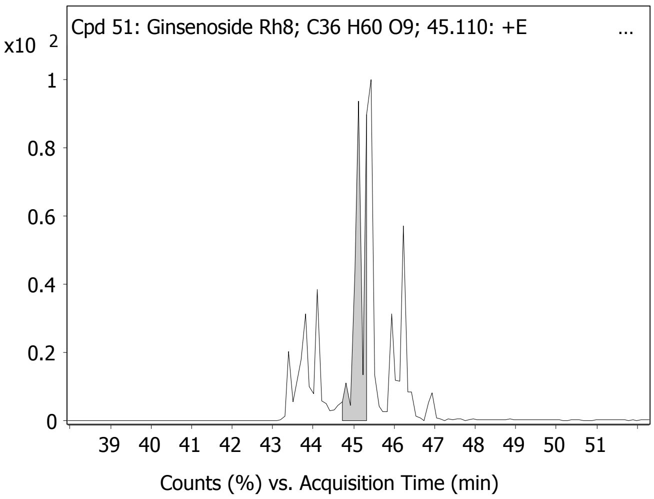

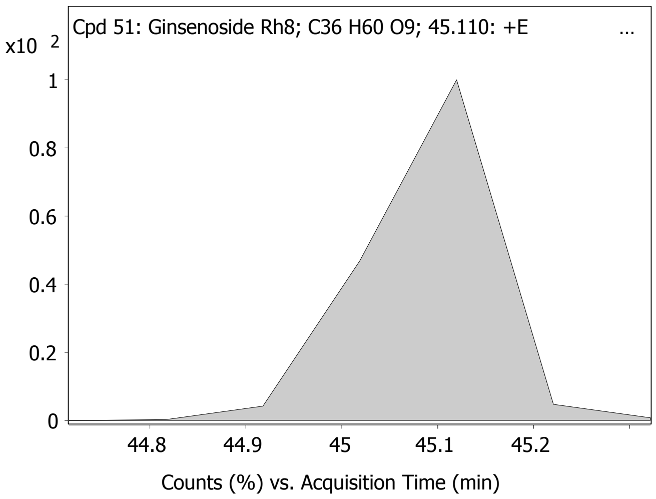

MFE MS Spectrum

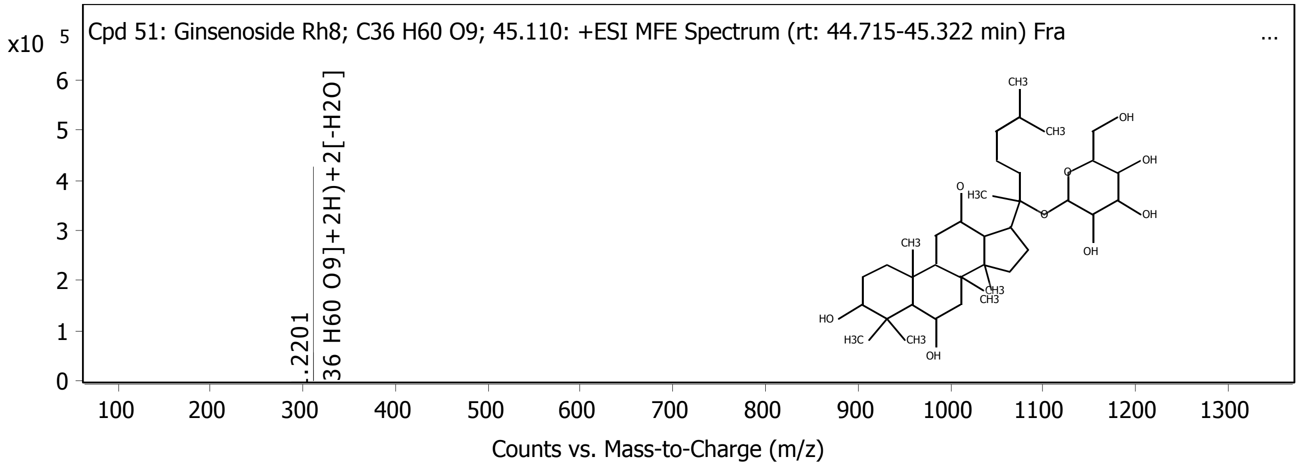

MFE MS Zoomed Spectrum

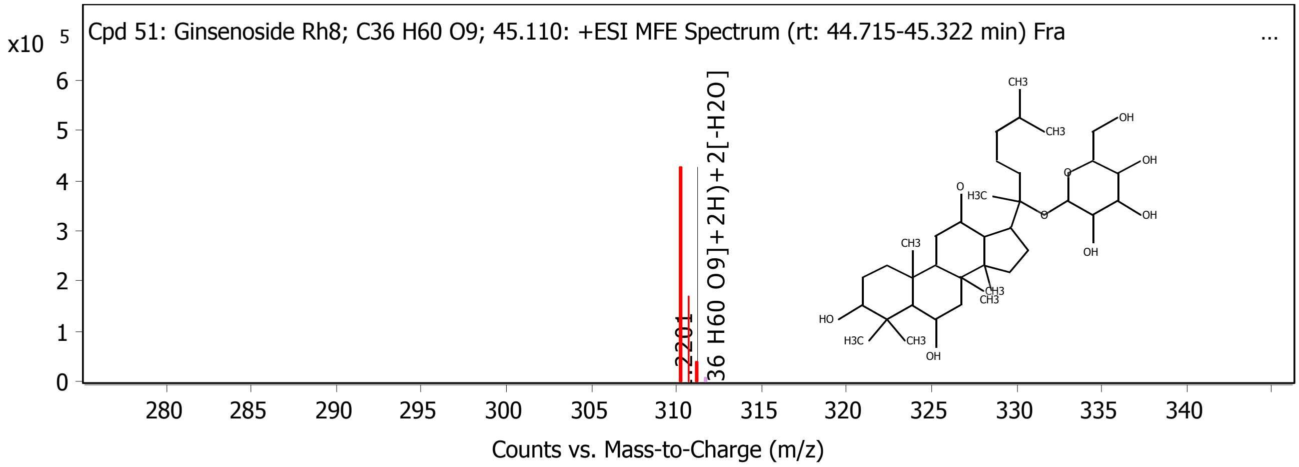

MS Spectrum

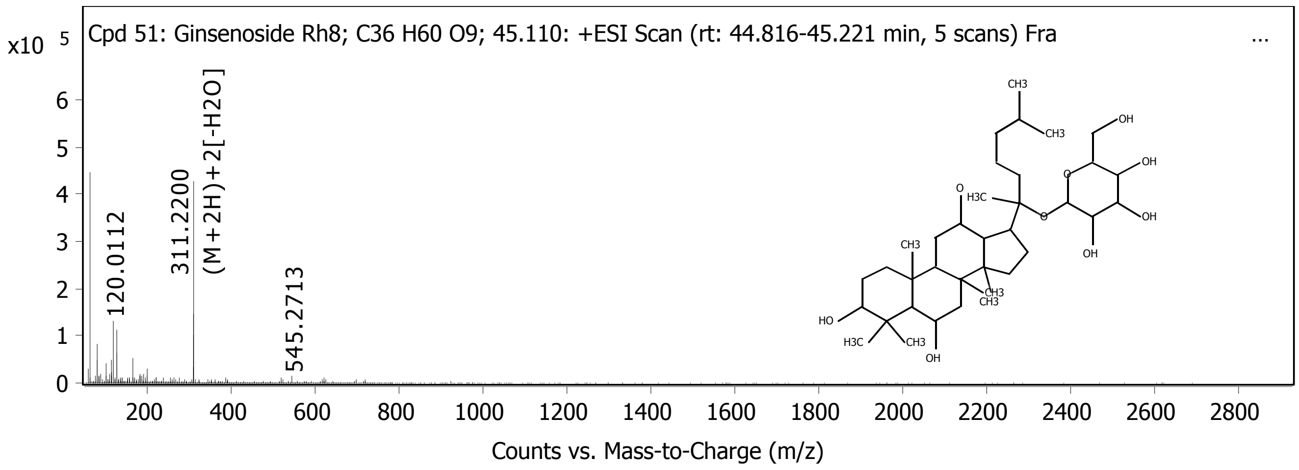

MS Zoomed Spectrum

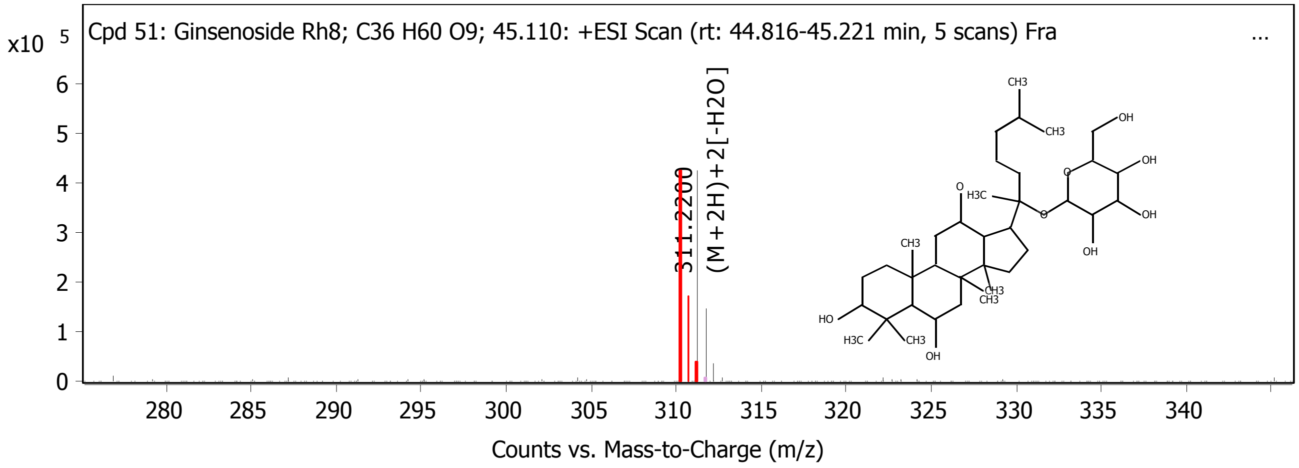

MSMS Spectrum

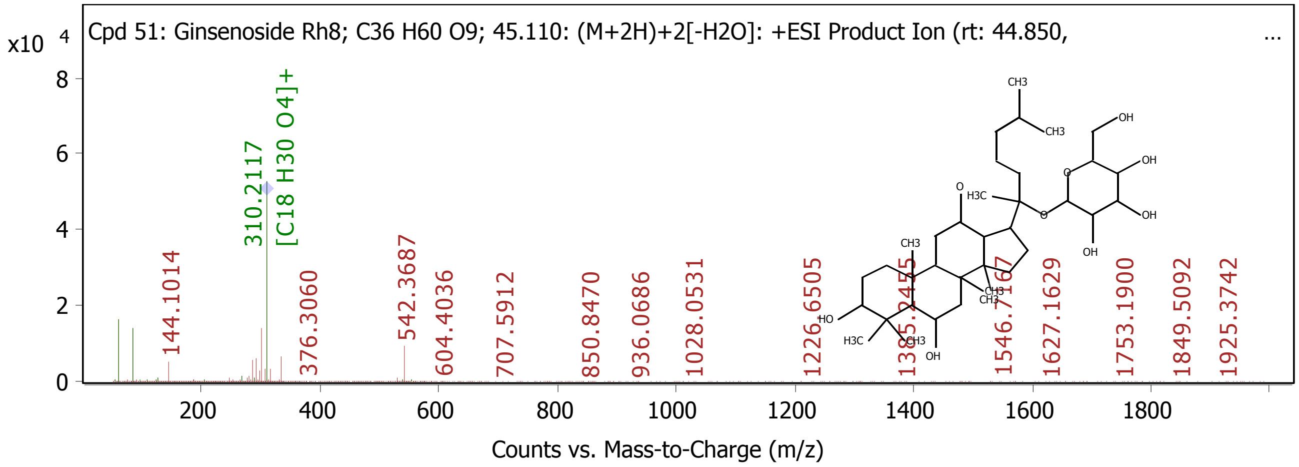

Identification Hit Table

Best Hit	Compound Name	RT	Formula	Notes	Match Score	Mass	Difference	Ion Species
✓	Ginsenoside Rh8	45.11	C36 H60 O9		40.22	636.4284	-4.62	(M+2H)+2[-H2O]
	Ginsenoside Rh7	45.11	C36 H60 O9		40.22	636.4284	-4.62	(M+2H)+2[-H2O]
	Fasciculic acid B	45.11	C36 H60 O9		40.22	636.4284	-4.62	(M+2H)+2[-H2O]
	Momordicoside I	45.11	C36 H58 O8		39.61	618.4178	-4.62	(M+2H)+2
	Lansioside B	45.11	C36 H58 O8		39.61	618.4178	-4.62	(M+2H)+2
	19-Methoxypomolic acid 3-arabinoside	45.11	C36 H58 O8		39.61	618.4178	-4.62	(M+2H)+2

Identification Hit Table

Best Hit	Compound Name	RT	Formula	Notes	Match Score	Mass	Difference	Ion Species

Identification Hit Table

Best Hit	Compound Name	RT	Formula	Notes	Match Score	Mass	Difference	Ion Species

Qualitative Compound Identification Report

Compound Label	Name	m/z	RT	Algorithm	Mass
Cpd 52: Sanchinoside B1; C36 H62 O9; 45.358	**Sanchinoside B1**	311.2202	45.358	Find by Molecular Feature	638.4363

Compound Chromatograms

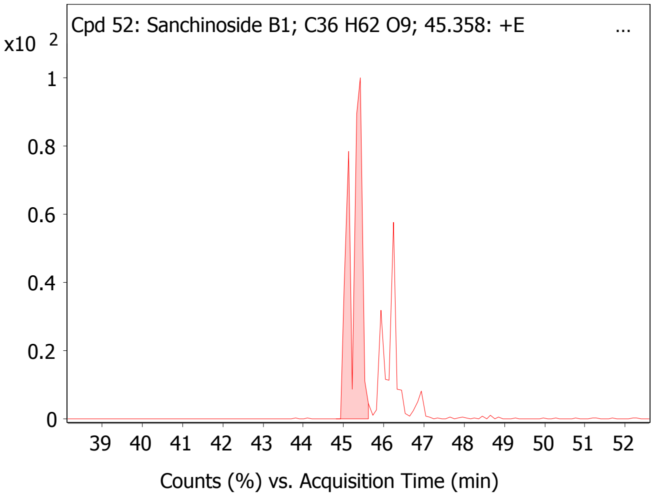

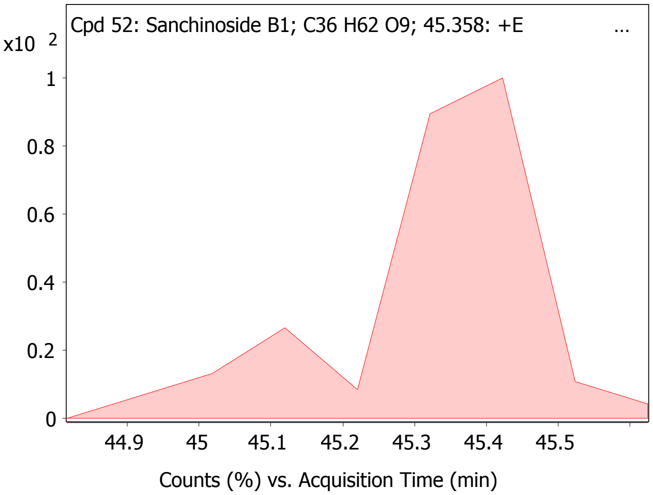

MFE MS Spectrum

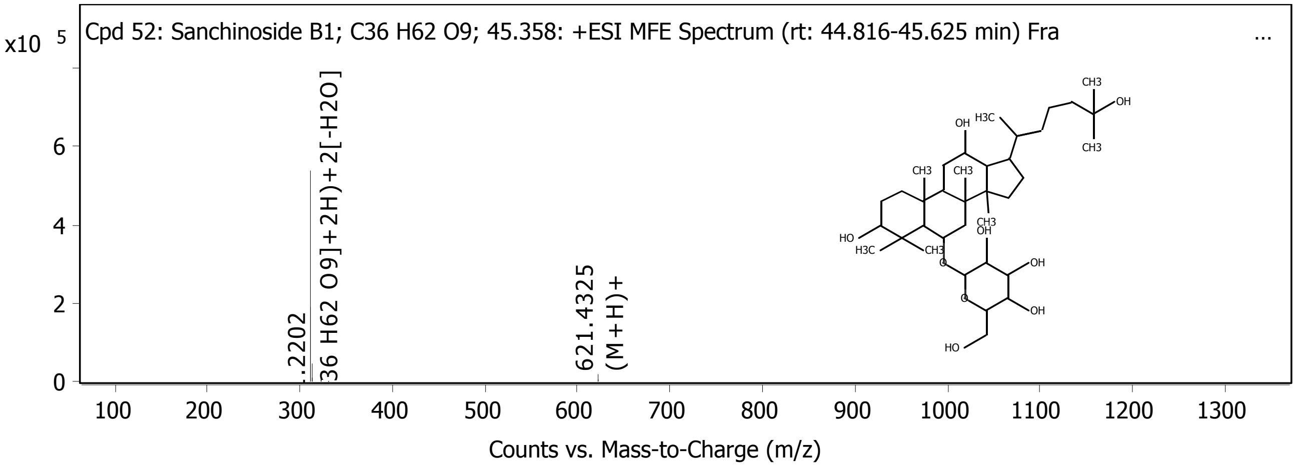

MFE MS Zoomed Spectrum

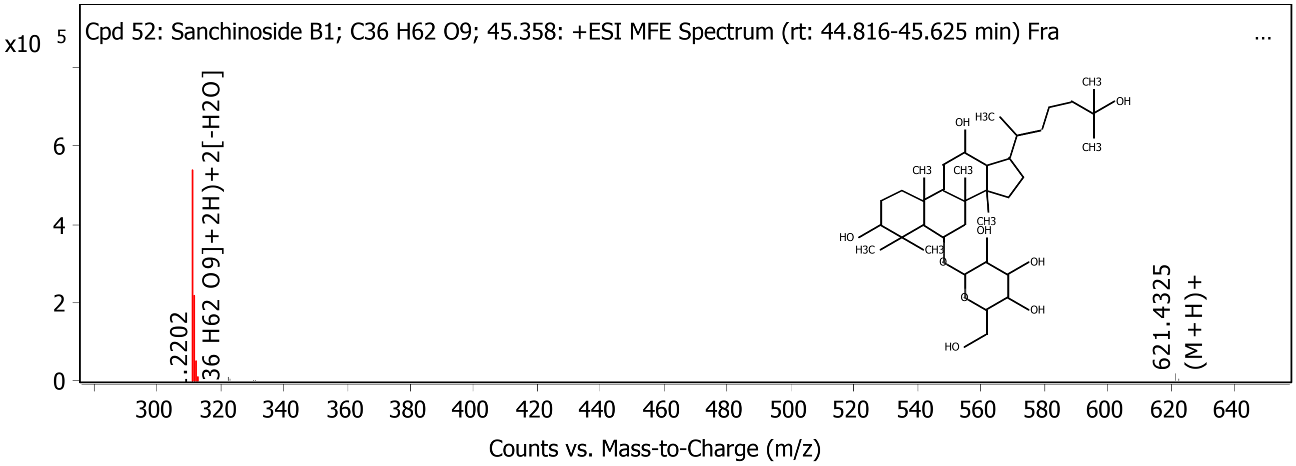

MS Spectrum

Qualitative Compound Identification Report

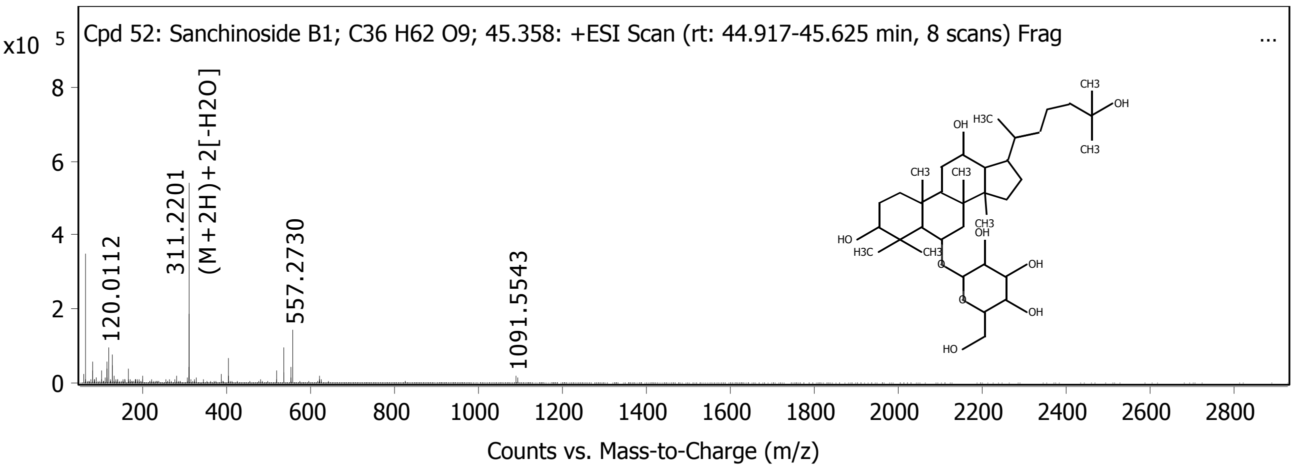

MS Zoomed Spectrum

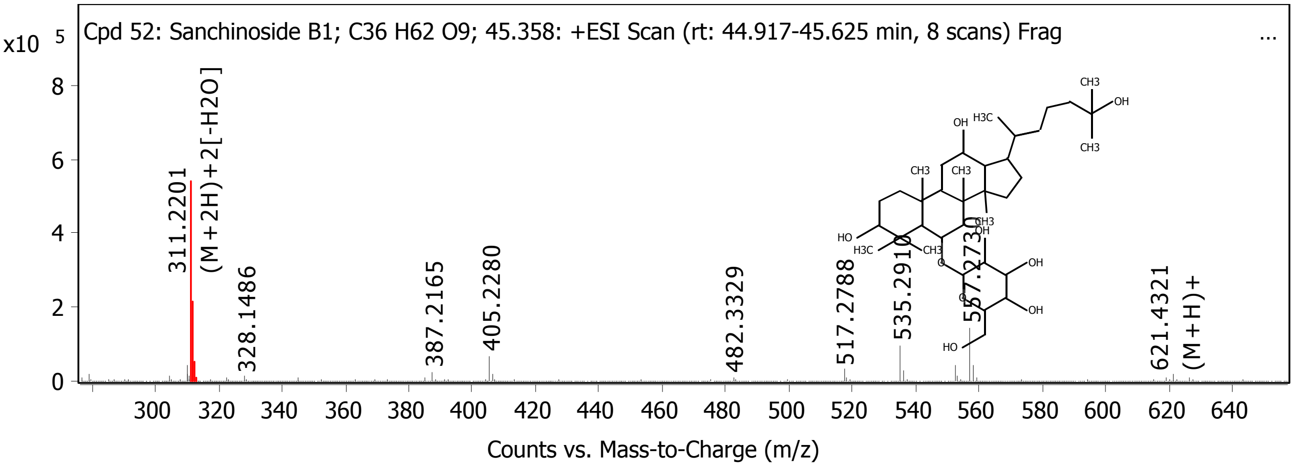

MSMS Spectrum

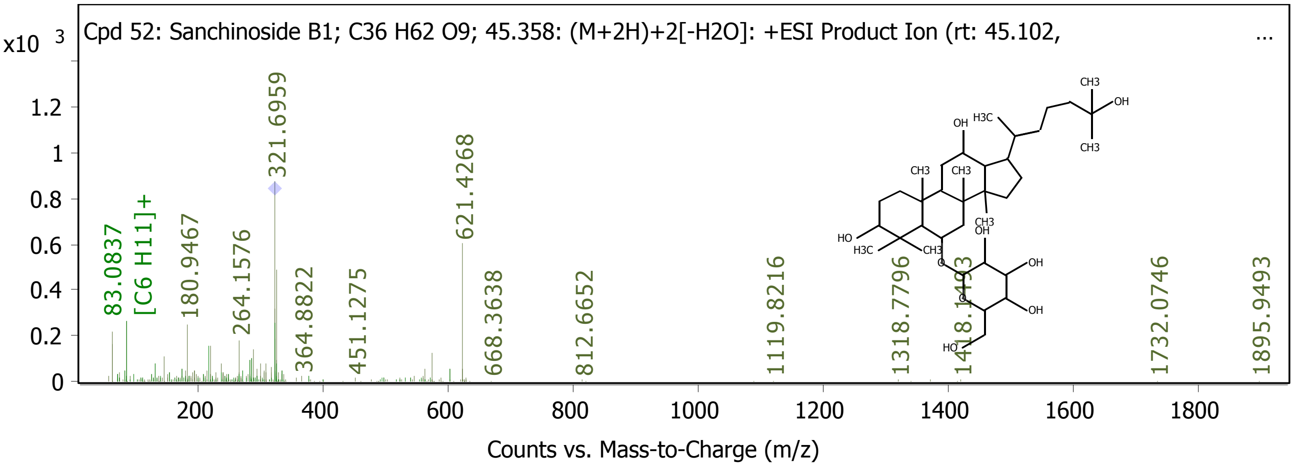

Identification Hit Table

Best Hit	Compound Name	RT	Formula	Notes	Match Score	Mass	Difference	Ion Species
✓	Sanchinoside B1	45.358	C36 H62 O9		85.1	638.4363	3.08	(M+2H)+2[-H2O]
	Ginsenoside Rh1	45.358	C36 H62 O9		85.1	638.4363	3.08	(M+2H)+2[-H2O]
	Ginsenoside F1	45.358	C36 H62 O9		85.1	638.4363	3.08	(M+2H)+2[-H2O]
	Fasciculic acid A	45.358	C36 H60 O8		84.7	620.4257	3.08	(M+2H)+2
	(3b,7b,22x)-Cucurbita-5,24-diene-3,7,23-triol 7-glucoside	45.358	C36 H60 O8		84.7	620.4257	3.08	(M+2H)+2
	Soyasapogenol B 24-O-b-D-glucoside	45.358	C36 H60 O8		84.7	620.4257	3.08	(M+2H)+2
	Ginsenoside Rh4	45.358	C36 H60 O8		84.7	620.4257	3.08	(M+2H)+2
	PC(10:0/14:0)[U]	45.358	C32 H65 N O8 P		11.75	311.0269	311417.91	M+
	PC(9:0/15:0)[U]	45.358	C32 H65 N O8 P		11.75	311.0269	311417.91	M+
	PC(6:0/18:0)	45.358	C32 H65 N O8 P		11.75	311.0269	311417.91	M+

Qualitative Compound Identification Report

Identification Hit Table

Best Hit	Compound Name	RT	Formula	Notes	Match Score	Mass	Difference	Ion Species
✓	PC(10:0/14:0)[U]	45.358	C32 H65 N O8 P		11.75	311.0269	311417.91	M+
	PC(11:0/13:0)[U]	45.358	C32 H65 N O8 P		11.75	311.0269	311417.91	M+
	PC(12:0/12:0)	45.358	C32 H65 N O8 P		11.75	311.0269	311417.91	M+
	PC(12:0/12:0)[U]	45.358	C32 H65 N O8 P		11.75	311.0269	311417.91	M+
	PC(13:0/11:0)[U]	45.358	C32 H65 N O8 P		11.75	311.0269	311417.91	M+
	PC(14:0/10:0)[U]	45.358	C32 H65 N O8 P		11.75	311.0269	311417.91	M+
	PC(15:0/9:0)[U]	45.358	C32 H65 N O8 P		11.75	311.0269	311417.91	M+
	PC(18:0/6:0)[U]	45.358	C32 H65 N O8 P		11.75	311.0269	311417.91	M+
	PC(6:0/18:0)	45.358	C32 H65 N O8 P		11.75	311.0269	311417.91	M+
	PC(9:0/15:0)[U]	45.358	C32 H65 N O8 P		11.75	311.0269	311417.91	M+

Identification Hit Table

Best Hit	Compound Name	RT	Formula	Notes	Match Score	Mass	Difference	Ion Species

Compound Label	Name	*m/z*	RT	Algorithm	Mass
Cpd 53: Desglucocheirotoxol; C29 H44 O10; 45.727	**Desglucocheirotoxol**	535.2911	45.727	Find by Molecular Feature	552.2943

Compound Chromatograms

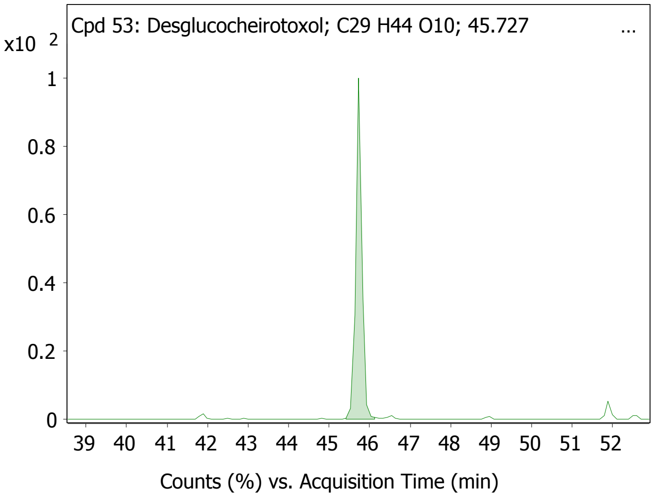

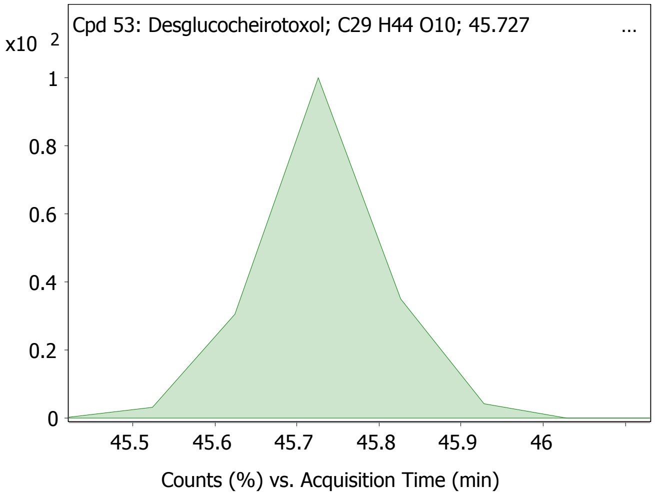

MFE MS Spectrum

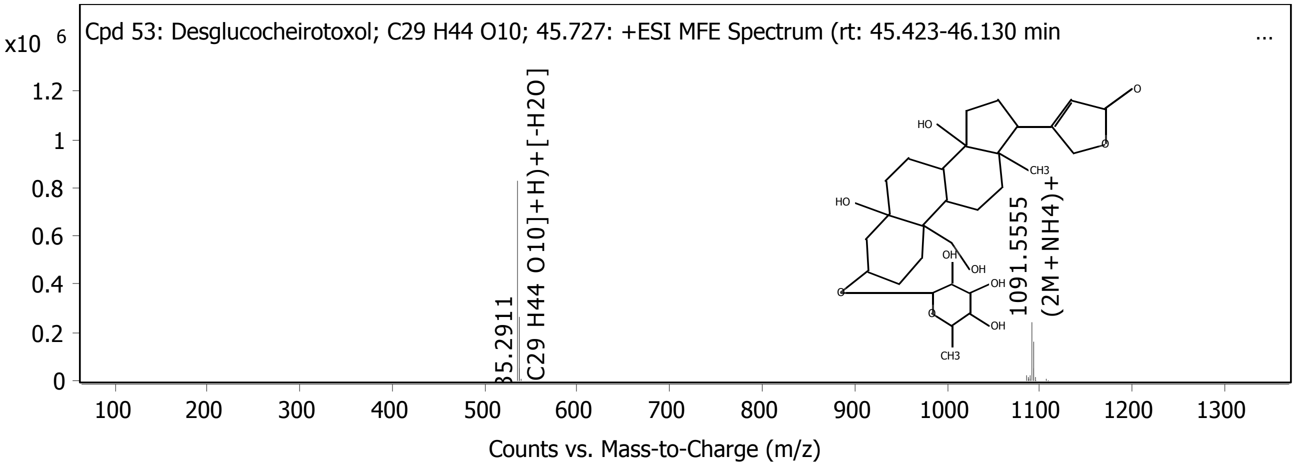

MFE MS Zoomed Spectrum

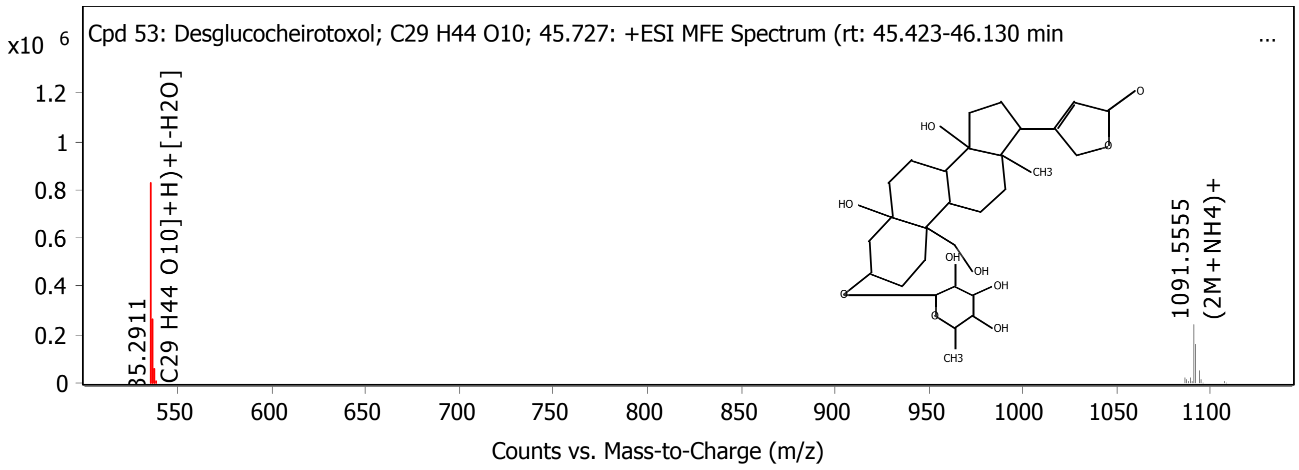

MS Spectrum

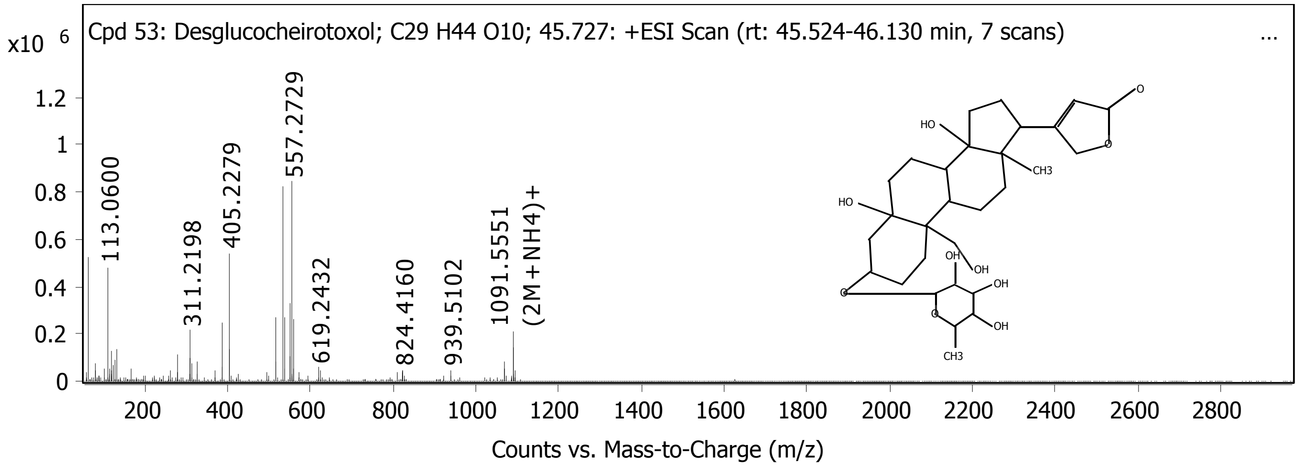

MS Zoomed Spectrum

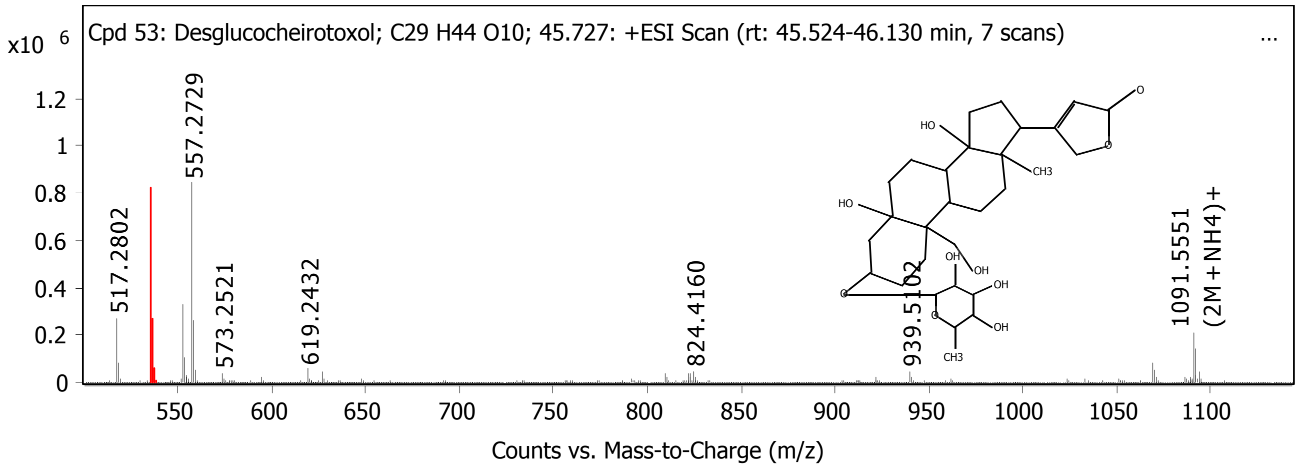

MSMS Spectrum

Qualitative Compound Identification Report

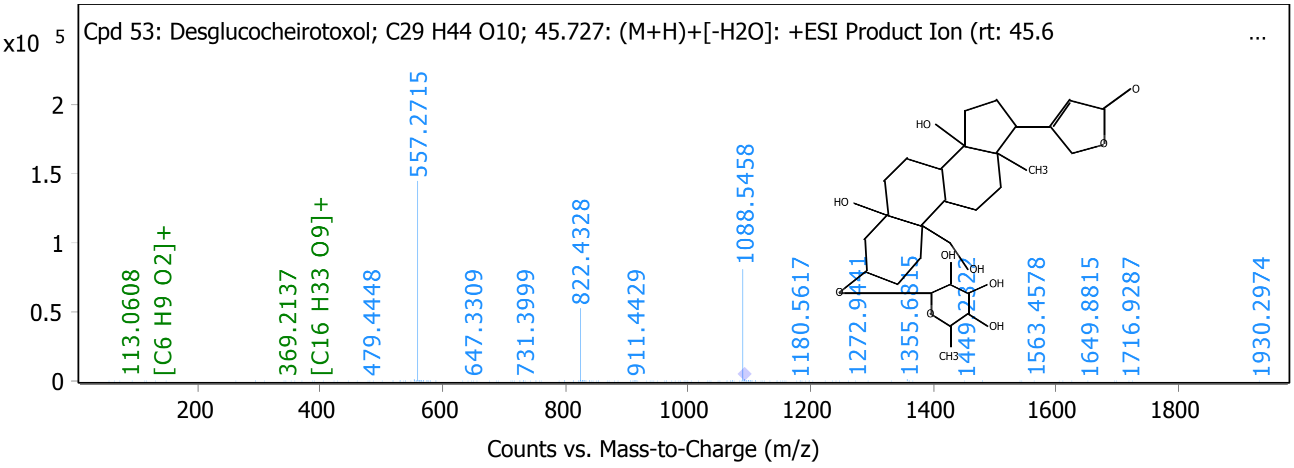

Identification Hit Table

Best Hit	Compound Name	RT	Formula	Notes	Match Score	Mass	Difference	Ion Species
✓	Desglucocheirotoxol	45.727	C29 H44 O10		98.4	552.2943	-0.85	(M+H)+[-H2O]
	Bipindogulomethyloside	45.727	C29 H44 O10		98.4	552.2943	-0.85	(M+H)+[-H2O]
	Panogenin-3-o-alpha-L-rhamnopyranoside	45.727	C29 H44 O10		98.4	552.2943	-0.85	(M+H)+[-H2O]
	antioside	45.727	C29 H44 O10		98.4	552.2943	-0.85	(M+H)+[-H2O]
	lokundjoside	45.727	C29 H44 O10		98.4	552.2943	-0.85	(M+H)+[-H2O]
	Periplogenin glucoside	45.727	C29 H44 O10	Deleted CAS: 911652-24-9 MolPort compound number: MolPort-005-945-533	98.4	552.2943	-0.85	(M+H)+[-H2O]
	Corchoroside A	45.727	C29 H42 O9		98.35	534.2837	-0.85	(M+H)+
	Corotoxigenin-3-O-alpha-L-rhamnopyranoside	45.727	C29 H42 O9		98.35	534.2837	-0.85	(M+H)+
	Helveticoside	45.727	C29 H42 O9		98.35	534.2837	-0.85	(M+H)+

Identification Hit Table

Best Hit	Compound Name	RT	Formula	Notes	Match Score	Mass	Difference	Ion Species
✓	lokundjoside	45.727	C29 H44 O10		98.4	552.2943	-0.85	(M+H)+[-H2O]
	antioside	45.727	C29 H44 O10		98.4	552.2943	-0.85	(M+H)+[-H2O]

Identification Hit Table

Best Hit	Compound Name	RT	Formula	Notes	Match Score	Mass	Difference	Ion Species
✓	Glu Lys Lys Met	45.727	C22 H42 N6 O7 S		90.04	534.2842	-0.61	(M+H)+
	Glu Lys Met Lys	45.727	C22 H42 N6 O7 S		90.04	534.2842	-0.61	(M+H)+
	Lys Met Glu Lys	45.727	C22 H42 N6 O7 S		90.04	534.2842	-0.61	(M+H)+
	Lys Lys Glu Met	45.727	C22 H42 N6 O7 S		90.04	534.2842	-0.61	(M+H)+
	Lys Glu Met Lys	45.727	C22 H42 N6 O7 S		90.04	534.2842	-0.61	(M+H)+
	Lys Glu Lys Met	45.727	C22 H42 N6 O7 S		90.04	534.2842	-0.61	(M+H)+
	Lys Met Lys Glu	45.727	C22 H42 N6 O7 S		90.04	534.2842	-0.61	(M+H)+
	Lys Lys Met Glu	45.727	C22 H42 N6 O7 S		90.04	534.2842	-0.61	(M+H)+
	Met Glu Lys Lys	45.727	C22 H42 N6 O7 S		90.04	534.2842	-0.61	(M+H)+
	Met Lys Glu Lys	45.727	C22 H42 N6 O7 S		90.04	534.2842	-0.61	(M+H)+

Compound Label	Name	*m/z*	RT	Algorithm	Mass
Cpd 54: Corchoroside A; C29 H42 O9; 45.727	**Corchoroside A**	517.2803	45.727	Find by Molecular Feature	534.2835

Compound Chromatograms

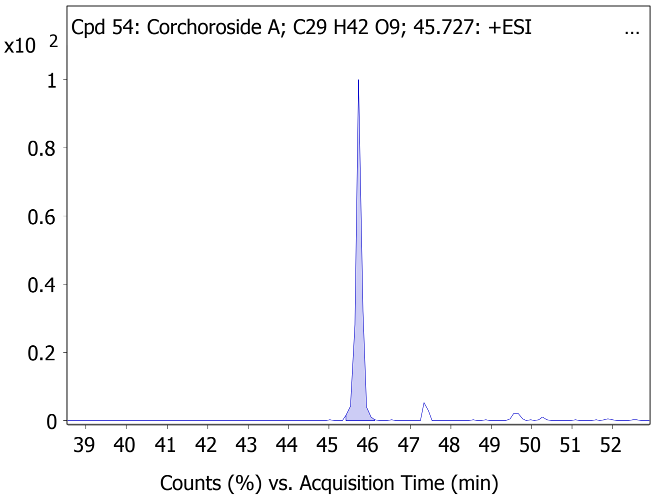

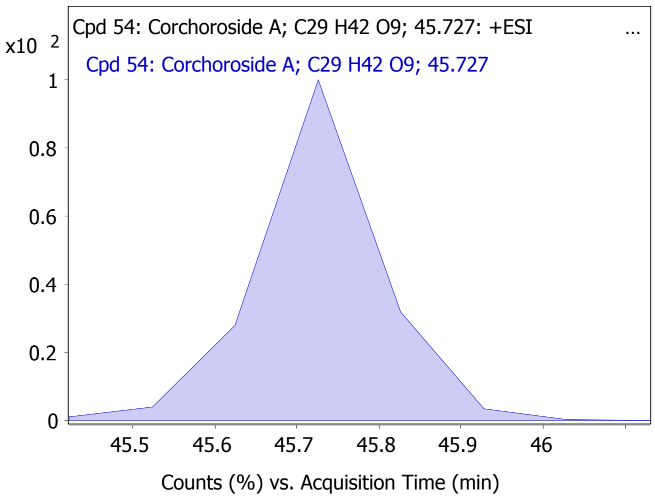

MFE MS Spectrum

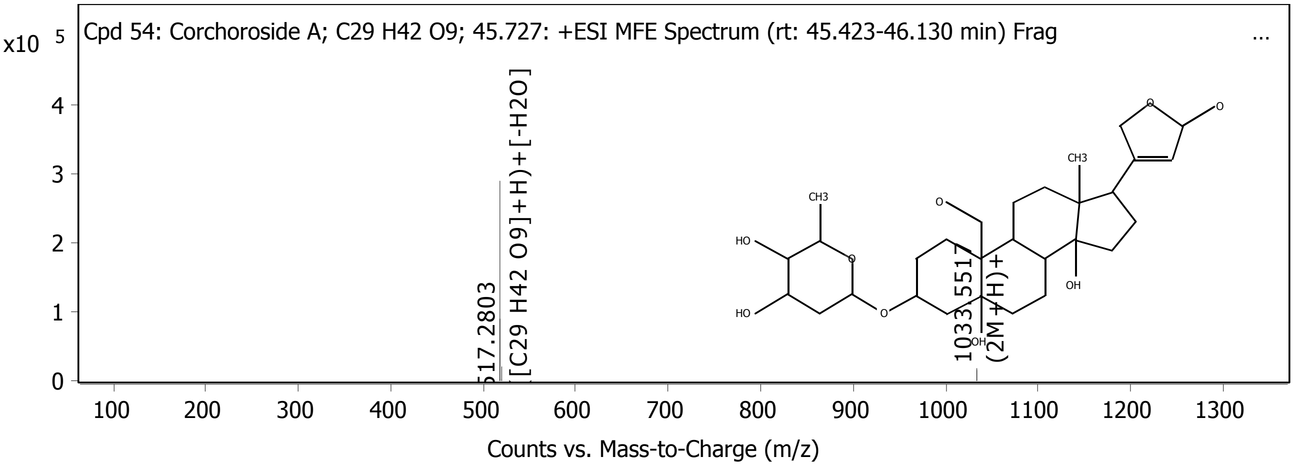

MFE MS Zoomed Spectrum

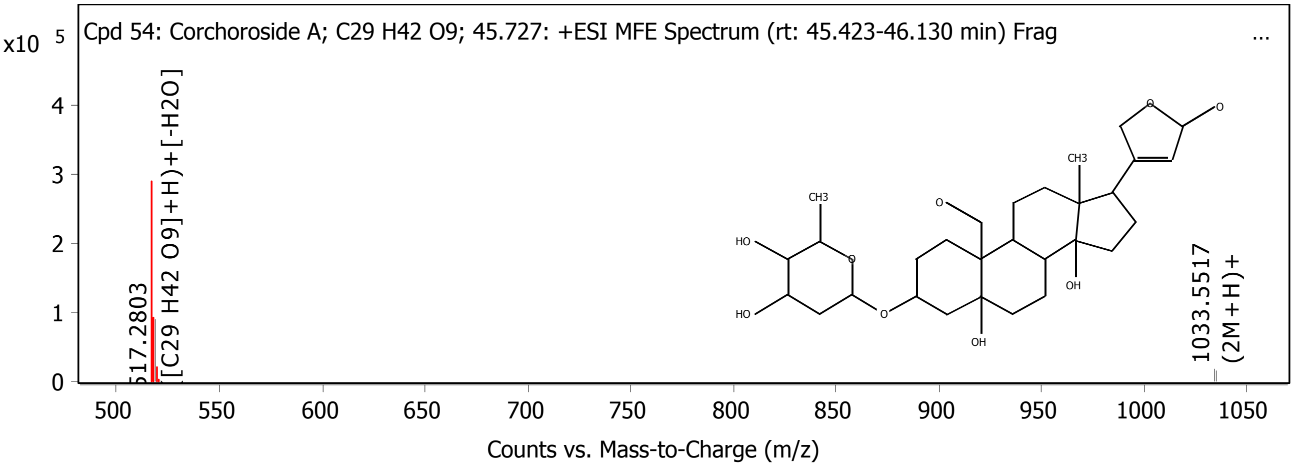

MS Spectrum

Qualitative Compound Identification Report

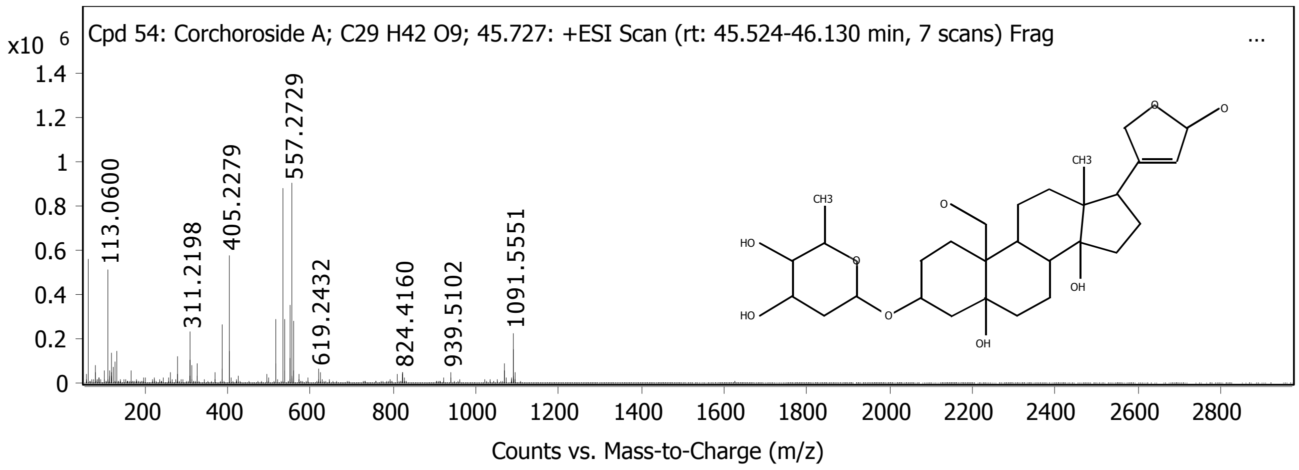

MS Zoomed Spectrum

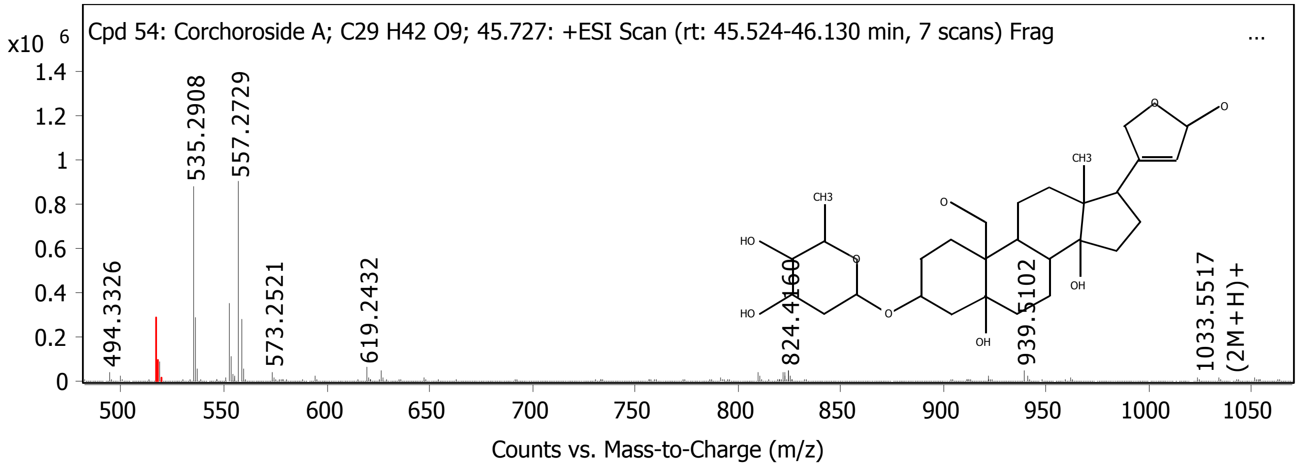

MSMS Spectrum

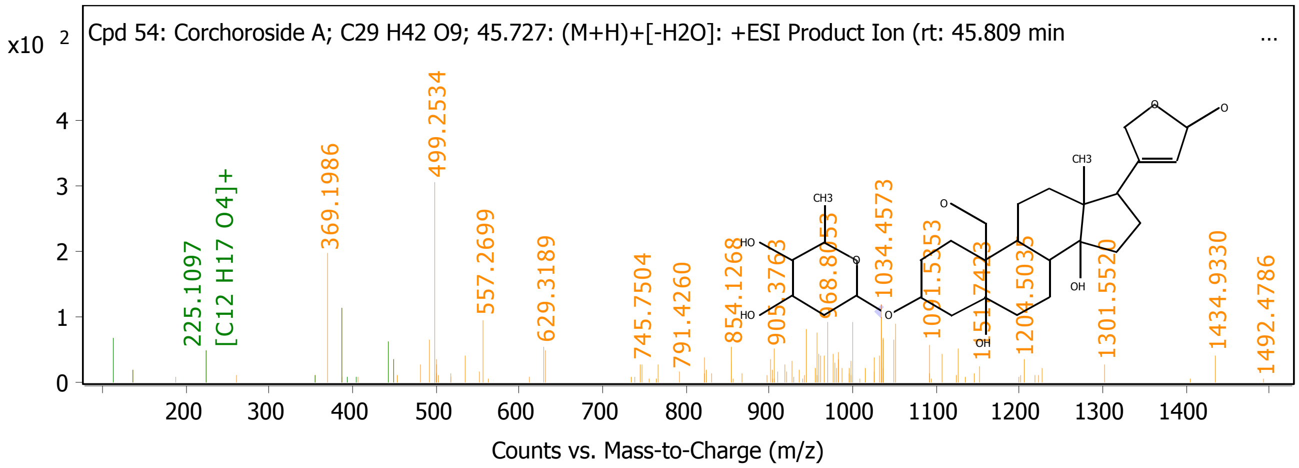

Identification Hit Table

Best Hit	Compound Name	RT	Formula	Notes	Match Score	Mass	Difference	Ion Species
✓	Corchoroside A	45.727	C29 H42 O9		98.72	534.2835	-0.6	(M+H)+[-H2O]
	Corotoxigenin-3-O-alpha-L-rhamnopyranoside	45.727	C29 H42 O9		98.72	534.2835	-0.6	(M+H)+[-H2O]
	Helveticoside	45.727	C29 H42 O9		98.72	534.2835	-0.6	(M+H)+[-H2O]
	Lucidenic acid E2	45.727	C29 H40 O8		98.69	516.2729	-0.6	(M+H)+
	ajugalactone	45.727	C29 H40 O8		98.69	516.2729	-0.6	(M+H)+
	(25R)-11alpha,20,26-trihydroxyecdysone	45.727	C27 H44 O9		84.34	512.3015	-3.01	(M+Na)+[-H2O]
	(24R)-11alpha,20,24-trihydroxyecdysone	45.727	C27 H44 O9		84.34	512.3015	-3.01	(M+Na)+[-H2O]
	(25S)-11alpha,20,26-trihydroxyecdysone	45.727	C27 H44 O9		84.34	512.3015	-3.01	(M+Na)+[-H2O]

	(3b,4b,11b,14b)-11-Ethoxy-3,4-epoxy-14-hydroxy-12-cyathen-15-al 14-xyloside	45.727	C27 H42 O8		83.81	494.291	-3.01	(M+Na)+
	Thiobinupharidine	45.727	C30 H42 N2 O2 S		60.64	494.2913	5.41	(M+Na)+

Identification Hit Table

Best Hit	Compound Name	RT	Formula	Notes	Match Score	Mass	Difference	Ion Species
✓	ajugalactone	45.727	C29 H40 O8		98.69	516.2729	-0.6	(M+H)+
	(25S)-11alpha,20,26-trihydroxyecdysone	45.727	C27 H44 O9		84.34	512.3015	-3.01	(M+Na)+[-H2O]
	(24R)-11alpha,20,24-trihydroxyecdysone	45.727	C27 H44 O9		84.34	512.3015	-3.01	(M+Na)+[-H2O]
	(25R)-11alpha,20,26-trihydroxyecdysone	45.727	C27 H44 O9		84.34	512.3015	-3.01	(M+Na)+[-H2O]
	24-methylene-cholesterol sulfate	45.727	C28 H46 O4 S		43.51	478.3174	-5.73	(M+K)+

Identification Hit Table

Best Hit	Compound Name	RT	Formula	Notes	Match Score	Mass	Difference	Ion Species
✓	Lys Met Lys Glu	45.727	C22 H42 N6 O7 S		92.48	534.2839	-0.38	(M+H)+[-H2O]
	Lys Glu Met Lys	45.727	C22 H42 N6 O7 S		92.48	534.2839	-0.38	(M+H)+[-H2O]
	Lys Lys Glu Met	45.727	C22 H42 N6 O7 S		92.48	534.2839	-0.38	(M+H)+[-H2O]
	Glu Lys Lys Met	45.727	C22 H42 N6 O7 S		92.48	534.2839	-0.38	(M+H)+[-H2O]
	Glu Lys Met Lys	45.727	C22 H42 N6 O7 S		92.48	534.2839	-0.38	(M+H)+[-H2O]
	Glu Met Lys Lys	45.727	C22 H42 N6 O7 S		92.48	534.2839	-0.38	(M+H)+[-H2O]
	Met Glu Lys Lys	45.727	C22 H42 N6 O7 S		92.48	534.2839	-0.38	(M+H)+[-H2O]
	Met Lys Glu Lys	45.727	C22 H42 N6 O7 S		92.48	534.2839	-0.38	(M+H)+[-H2O]
	Met Lys Lys Glu	45.727	C22 H42 N6 O7 S		92.48	534.2839	-0.38	(M+H)+[-H2O]
	Lys Met Glu Lys	45.727	C22 H42 N6 O7 S		92.48	534.2839	-0.38	(M+H)+[-H2O]

Compound Label	Name	*m/z*	RT	Algorithm	Mass
Cpd 55: Picrasin C; C23 H34 O7; 45.727	**Picrasin C**	405.228	45.727	Find by Molecular Feature	422.2313

Compound Chromatograms

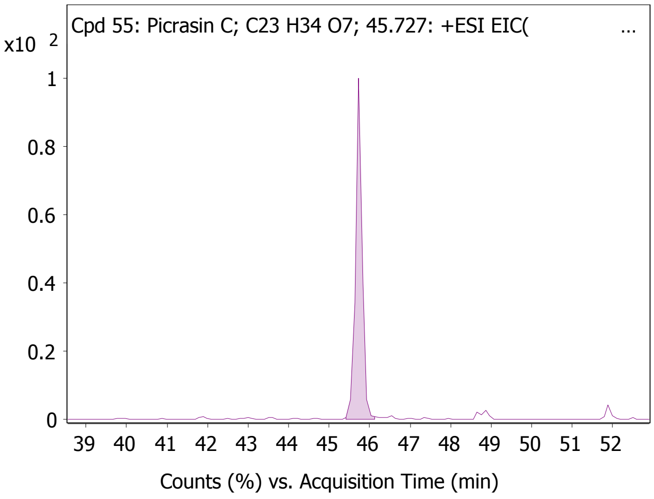

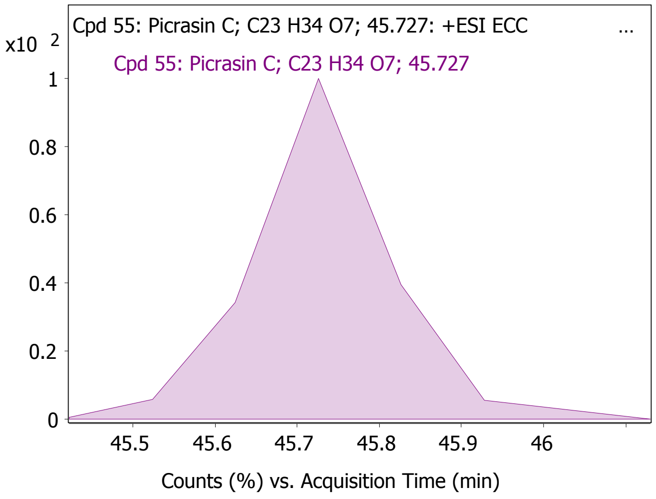

MFE MS Spectrum

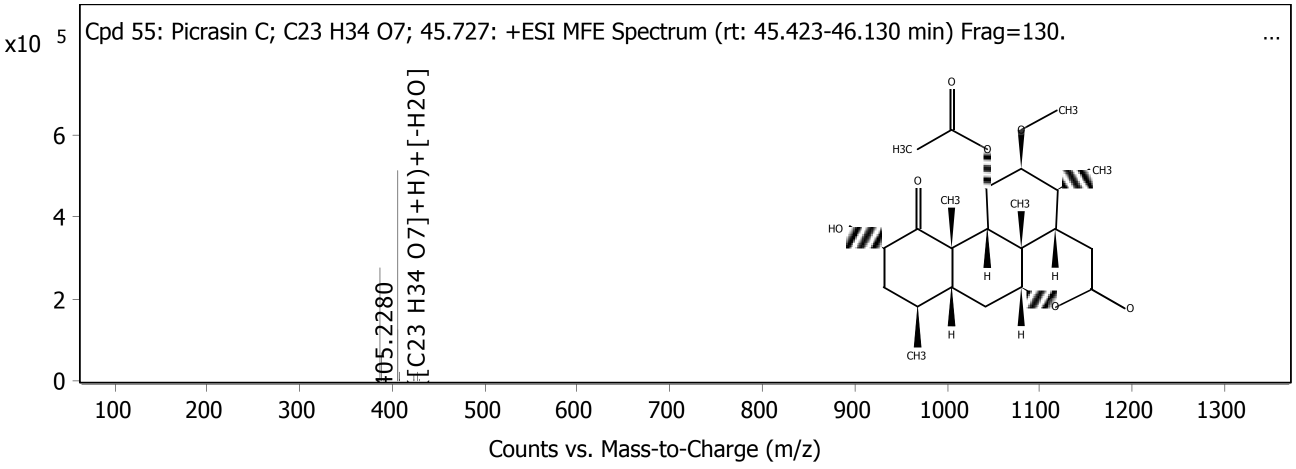

MFE MS Zoomed Spectrum

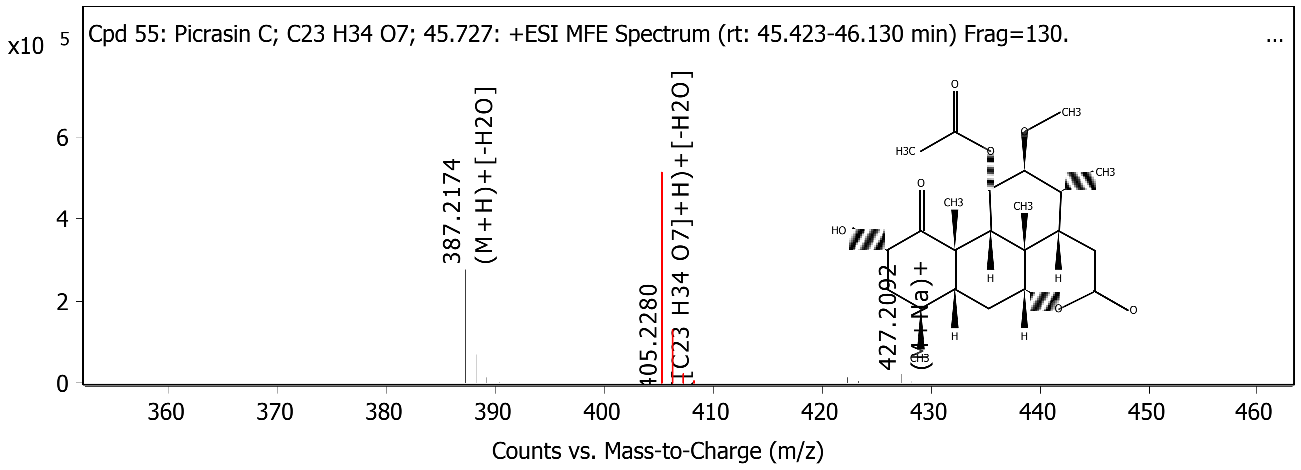

MS Spectrum

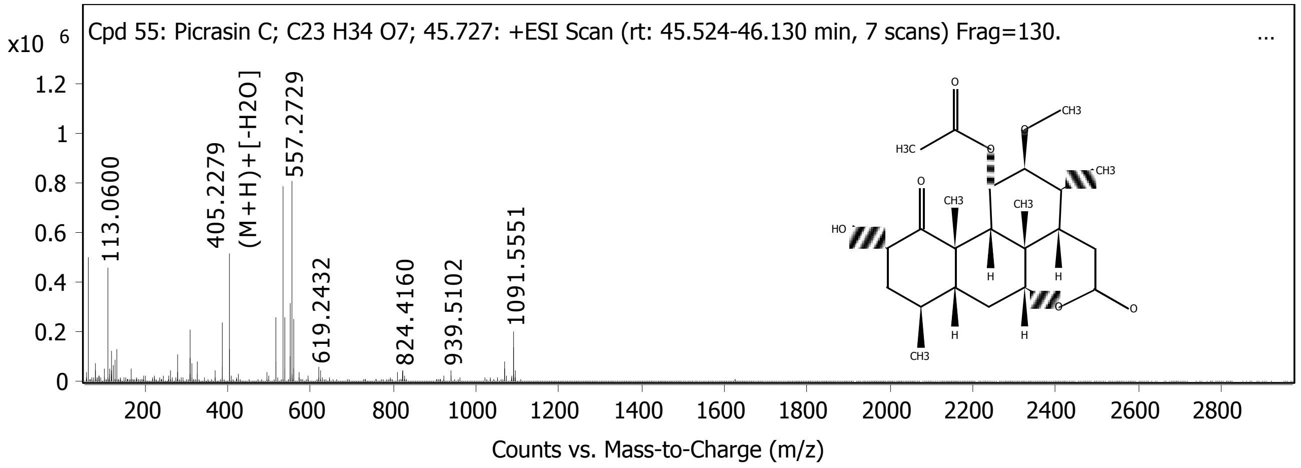

MS Zoomed Spectrum

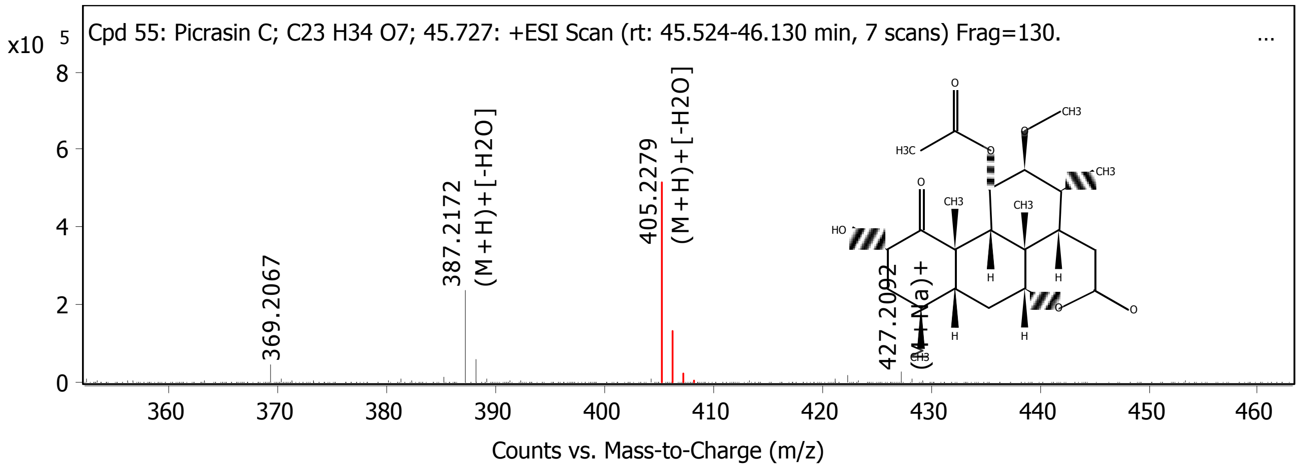

Identification Hit Table

Best Hit	Compound Name	RT	Formula	Notes	Match Score	Mass	Difference	Ion Species
✓	Picrasin C	45.727	C23 H34 O7		98.08	422.2313	-0.8	(M+H)+[-H2O]
	16-Methyl-epi-nigakihemiacetal B	45.727	C23 H32 O6		98.01	404.2207	-0.8	(M+H)+
	Cortisol 21-acetate	45.727	C23 H32 O6		98.01	404.2207	-0.8	(M+H)+
	17-phenoxy trinor Prostaglandin F2α	45.727	C23 H32 O6		98.01	404.2207	-0.8	(M+H)+
	Caesalpinin N	45.727	C23 H32 O6		98.01	404.2207	-0.8	(M+H)+
	Strophanthidin	45.727	C23 H32 O6		98.01	404.2207	-0.8	(M+H)+
	16-phenoxy tetranor PGF2α methyl ester	45.727	C23 H32 O6		98.01	404.2207	-0.8	(M+H)+
	1alpha-O-Methylquassin	45.727	C23 H32 O6		98.01	404.2207	-0.8	(M+H)+
	adonitoxigenin	45.727	C23 H32 O6		98.01	404.2207	-0.8	(M+H)+
	Val Arg Met	45.727	C16 H32 N6 O4 S		90.18	404.2211	-0.52	(M+H)+

Qualitative Compound Identification Report

Identification Hit Table

Best Hit	Compound Name	RT	Formula	Notes	Match Score	Mass	Difference	Ion Species
✓	Caesalpinin N	45.727	C23 H32 O6		98.01	404.2207	-0.8	(M+H)+
	Strophanthidin	45.727	C23 H32 O6		98.01	404.2207	-0.8	(M+H)+
	adonitoxigenin	45.727	C23 H32 O6		98.01	404.2207	-0.8	(M+H)+
	Cortisol 21-acetate	45.727	C23 H32 O6		98.01	404.2207	-0.8	(M+H)+
	Docusate	45.727	C20 H38 O7 S	The United States Pharmacopeia 29, The National Formulary 24, 2006	81.25	422.2315	2.33	(M+H)+[-H2O]

Identification Hit Table

Best Hit	Compound Name	RT	Formula	Notes	Match Score	Mass	Difference	Ion Species
✓	Val Met Arg	45.727	C16 H32 N6 O4 S		90.18	404.2211	-0.52	(M+H)+
	Arg Met Val	45.727	C16 H32 N6 O4 S		90.18	404.2211	-0.52	(M+H)+
	Met Val Arg	45.727	C16 H32 N6 O4 S		90.18	404.2211	-0.52	(M+H)+
	Met Arg Val	45.727	C16 H32 N6 O4 S		90.18	404.2211	-0.52	(M+H)+
	Arg Val Met	45.727	C16 H32 N6 O4 S		90.18	404.2211	-0.52	(M+H)+
	Val Arg Met	45.727	C16 H32 N6 O4 S		90.18	404.2211	-0.52	(M+H)+
	Gly Pro Leu His	45.727	C19 H30 N6 O5		75.65	422.2314	-3.63	(M+H)+[-H2O]
	His Gly Ile Pro	45.727	C19 H30 N6 O5		75.65	422.2314	-3.63	(M+H)+[-H2O]
	Gly Leu Pro His	45.727	C19 H30 N6 O5		75.65	422.2314	-3.63	(M+H)+[-H2O]
	Gly Pro His Ile	45.727	C19 H30 N6 O5		75.65	422.2314	-3.63	(M+H)+[-H2O]

Compound Label	*m/z*	RT	Algorithm	Mass
Cpd 56: C29 H35 N12; 45.728	552.3175	45.728	Find by Molecular Feature	551.3093

Compound Chromatograms

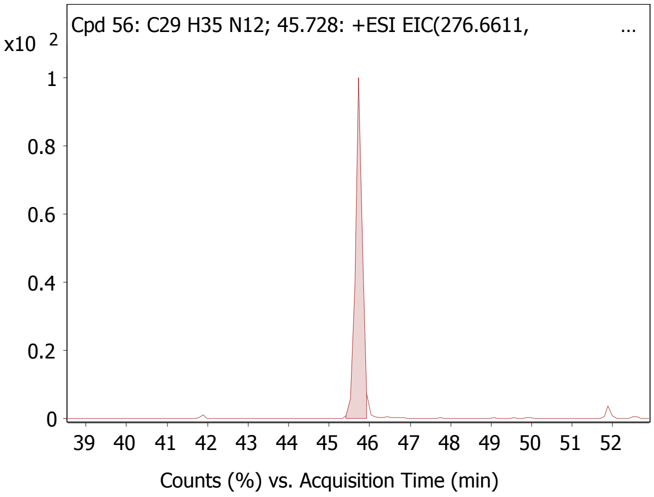

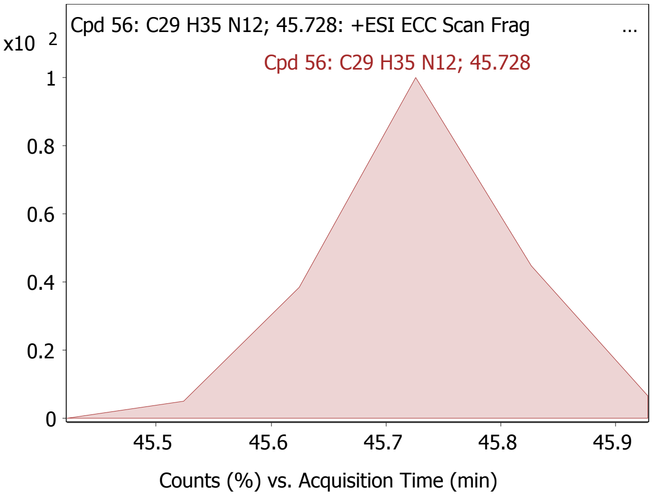

MFE MS Spectrum

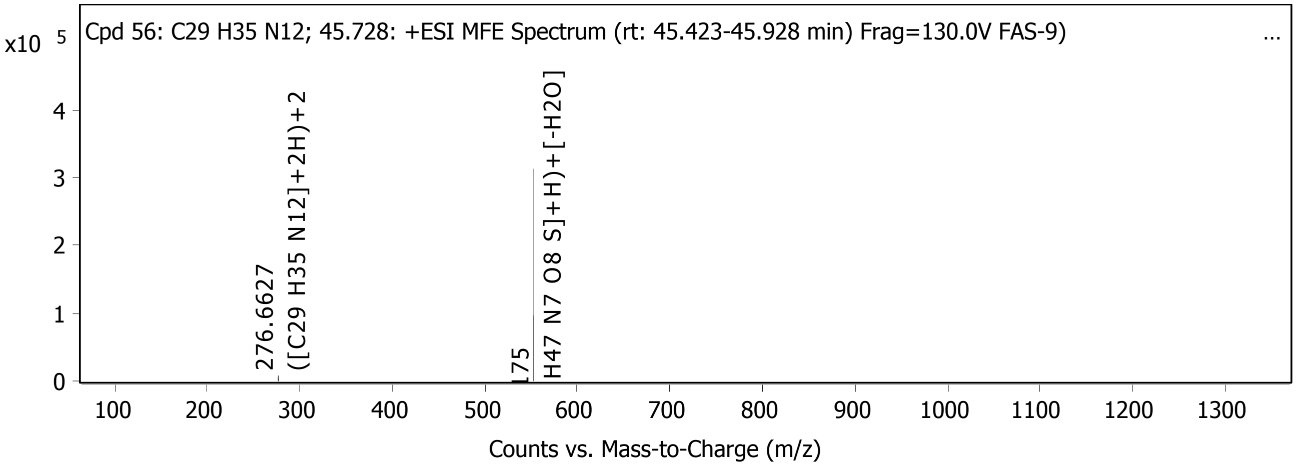

MFE MS Zoomed Spectrum

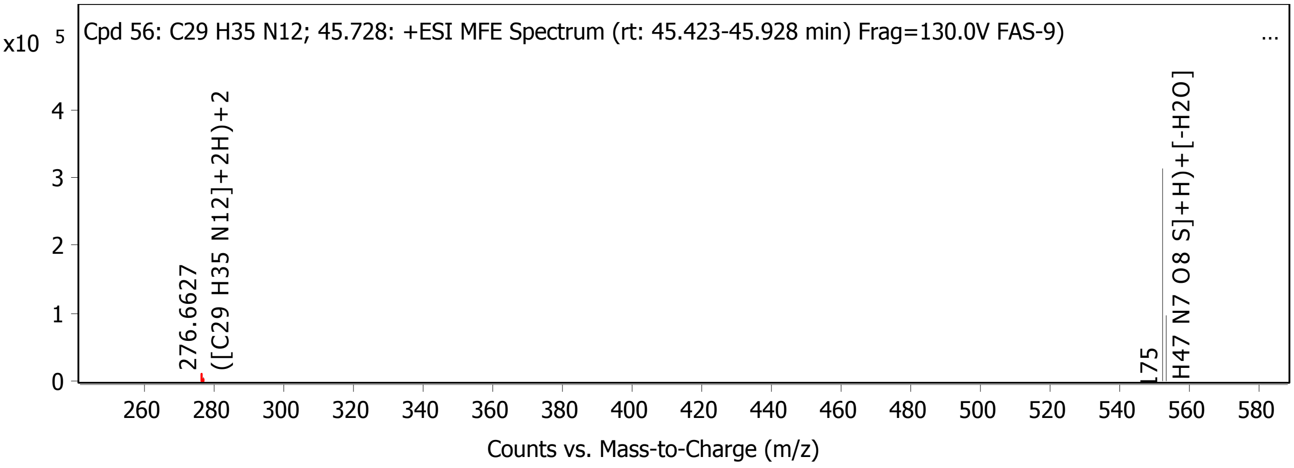

MS Spectrum

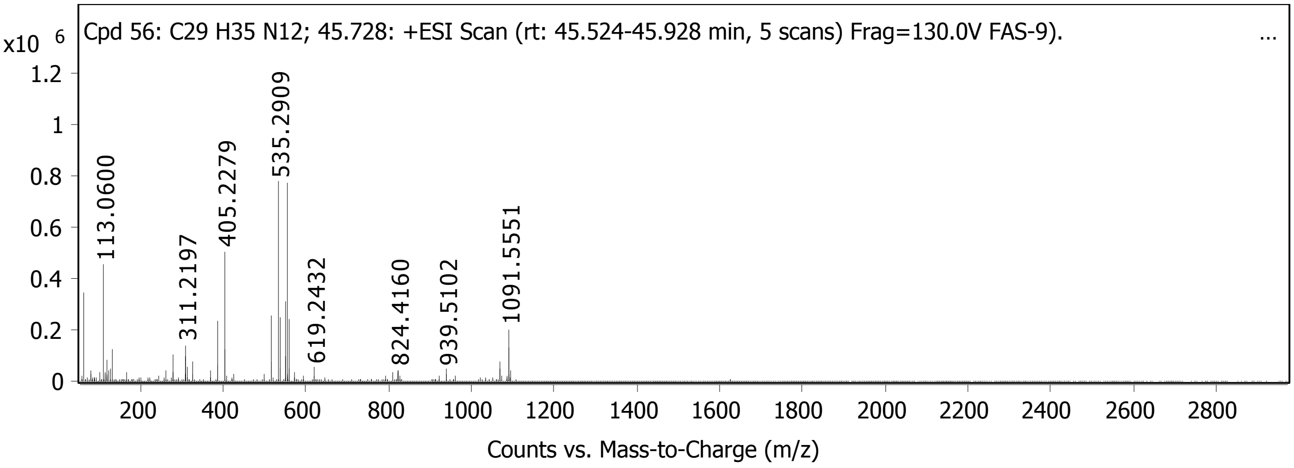

MS Zoomed Spectrum

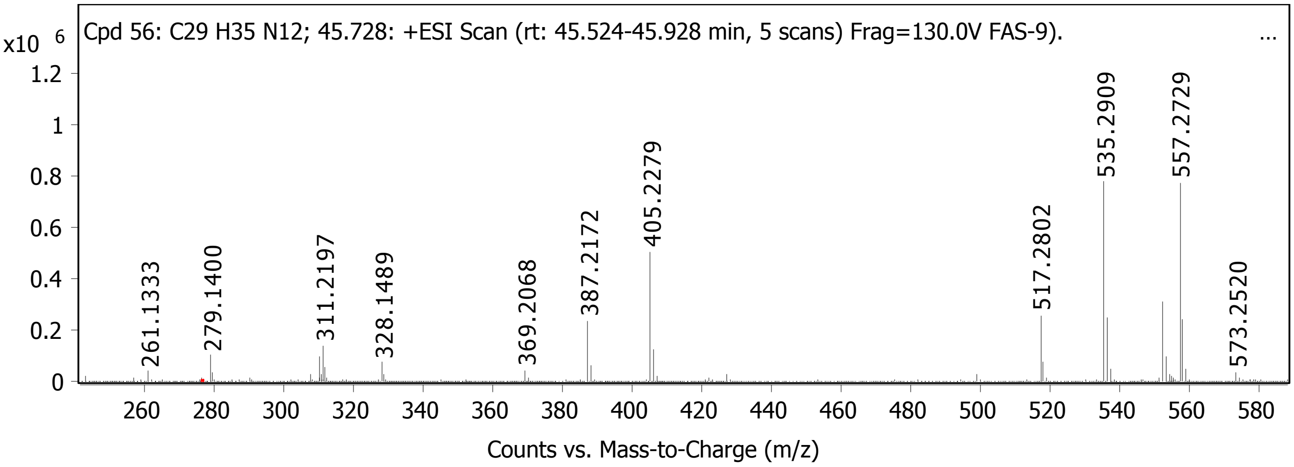

Compound Label	Name	m/z	RT	Algorithm	Mass
Cpd 57: Glu Trp Leu Gln; C27 H38 N6 O8; 45.728	**Glu Trp Leu Gln**	557.273	45.728	Find by Molecular Feature	574.2763

Compound Chromatograms

Qualitative Compound Identification Report

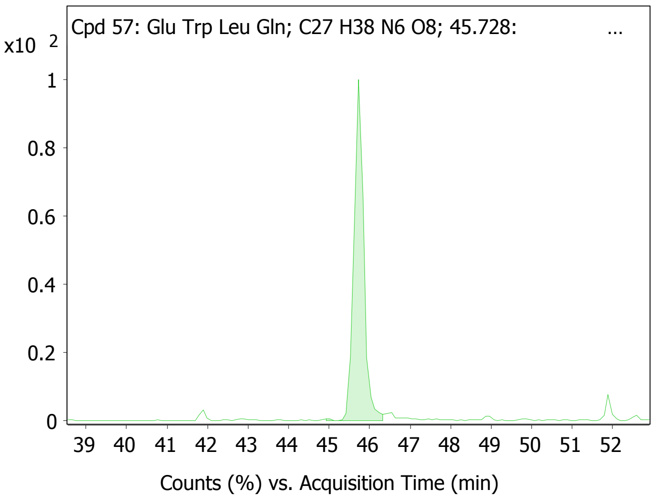

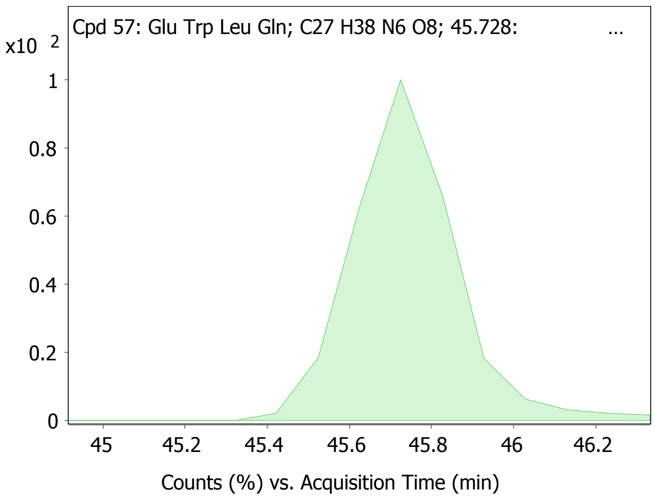

MFE MS Spectrum

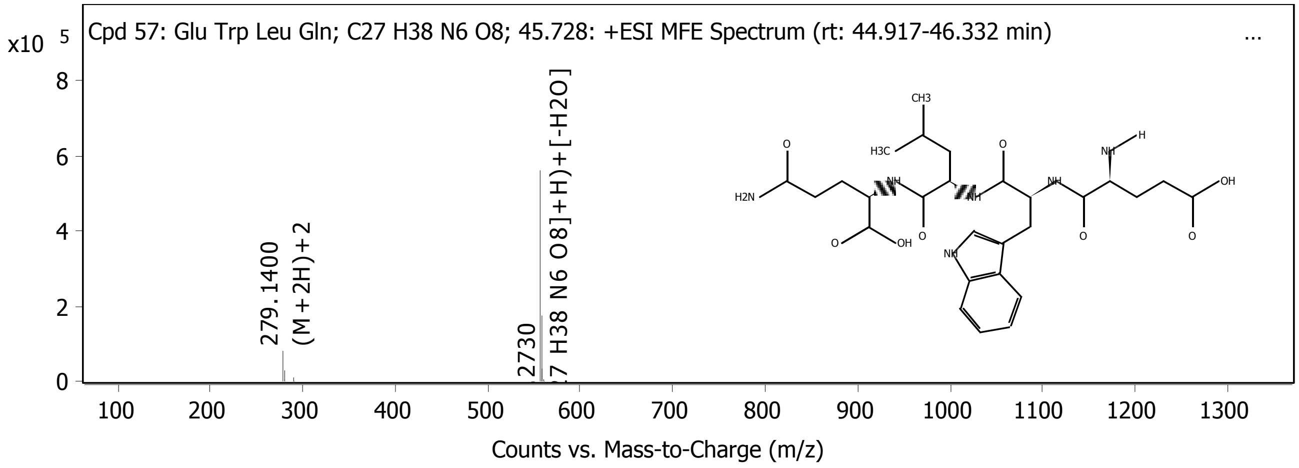

MFE MS Zoomed Spectrum

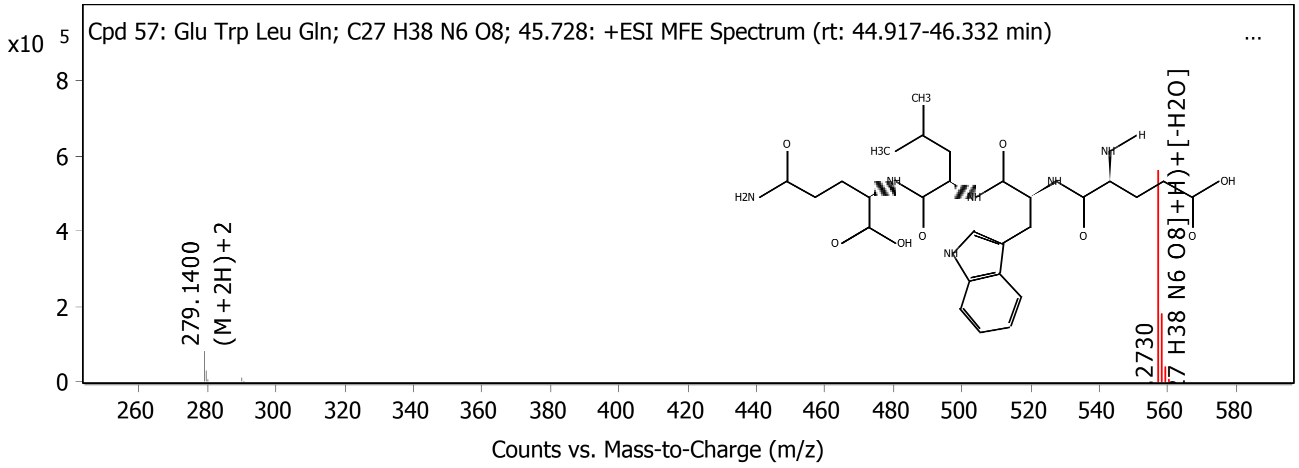

MS Spectrum

Qualitative Compound Identification Report

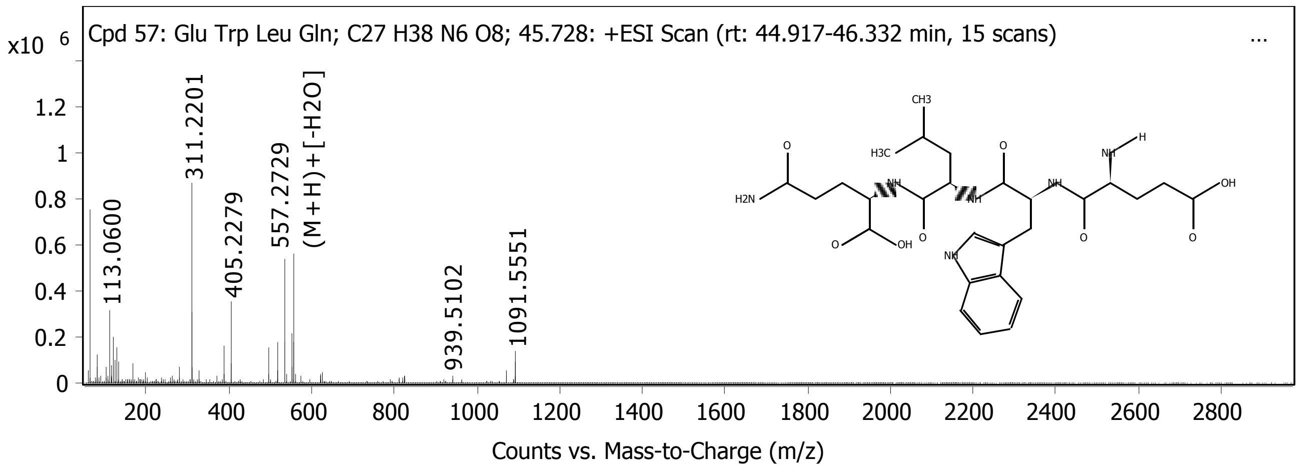

MS Zoomed Spectrum

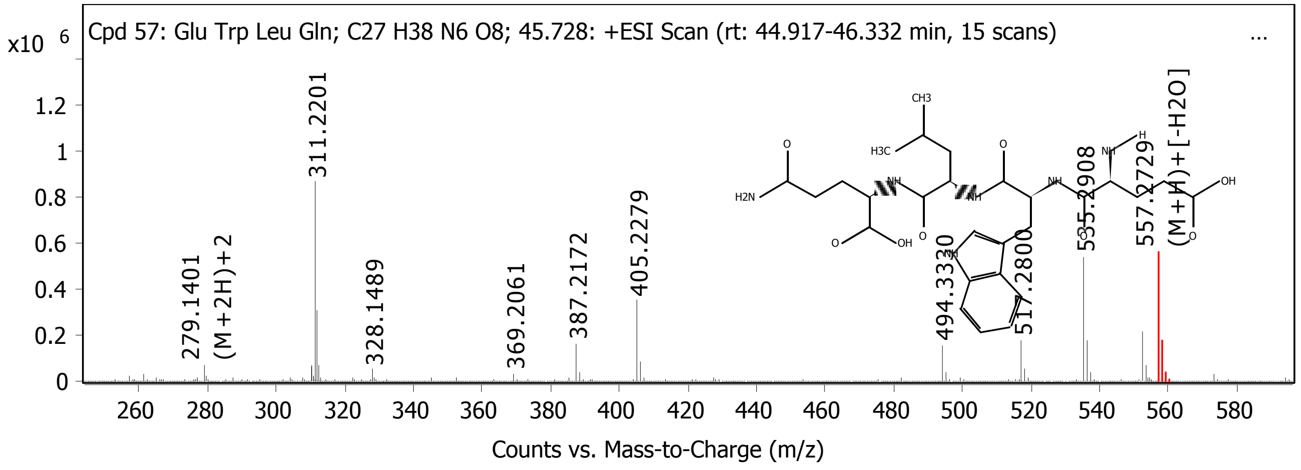

MSMS Spectrum

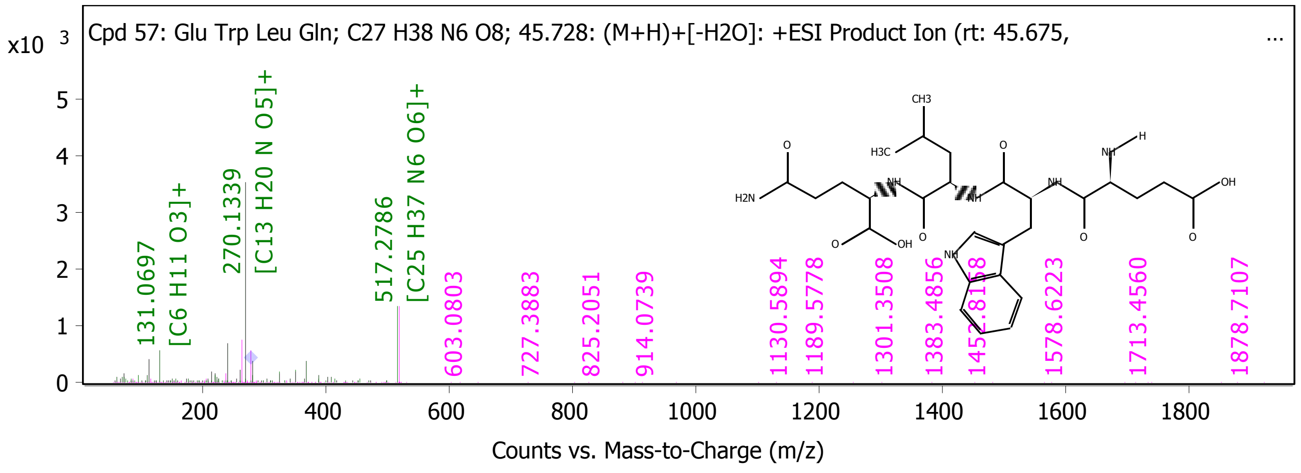

Identification Hit Table

Best Hit	Compound Name	RT	Formula	Notes	Match Score	Mass	Difference	Ion Species
✓	PI(15:1(9Z)/0:0)	45.728	C24 H45 O12 P		96.63	556.2656	-0.73	(M+H)+
	Asclepin	45.728	C31 H42 O10		93.57	574.2761	1.65	(M+H)+[-H2O]
	Dipiperamide C	45.728	C33 H36 N2 O6		51.01	556.2656	-8.27	(M+H)+

Identification Hit Table

Best Hit	Compound Name	RT	Formula	Notes	Match Score	Mass	Difference	Ion Species
✓	PI(15:1(9Z)/0:0)	45.728	C24 H45 O12 P		96.63	556.2656	-0.73	(M+H)+

Identification Hit Table

Best Hit	Compound Name	RT	Formula	Notes	Match Score	Mass	Difference	Ion Species
✓	Glu Trp Leu Gln	45.728	C27 H38 N6 O8		97.34	574.2763	-1.17	(M+H)+[-H2O]
	Leu Gln Trp Glu	45.728	C27 H38 N6 O8		97.34	574.2763	-1.17	(M+H)+[-H2O]
	Leu Glu Gln Trp	45.728	C27 H38 N6 O8		97.34	574.2763	-1.17	(M+H)+[-H2O]

Qualitative Compound Identification Report

	Glu Gln Trp Ile	45.728	C27 H38 N6 O8		97.34	574.2763	-1.17	(M+H)+[-H2O]
	Gln Ile Trp Glu	45.728	C27 H38 N6 O8		97.34	574.2763	-1.17	(M+H)+[-H2O]
	Gln Ile Glu Trp	45.728	C27 H38 N6 O8		97.34	574.2763	-1.17	(M+H)+[-H2O]
	Gln Glu Trp Leu	45.728	C27 H38 N6 O8		97.34	574.2763	-1.17	(M+H)+[-H2O]
	Gln Glu Trp Ile	45.728	C27 H38 N6 O8		97.34	574.2763	-1.17	(M+H)+[-H2O]
	Gln Glu Leu Trp	45.728	C27 H38 N6 O8		97.34	574.2763	-1.17	(M+H)+[-H2O]
	Gln Glu Ile Trp	45.728	C27 H38 N6 O8		97.34	574.2763	-1.17	(M+H)+[-H2O]

Compound Label	Name	*m/z*	RT	Algorithm	Mass
Cpd 58: Sanchinoside B1; C36 H62 O9; 46.198	**Sanchinoside B1**	311.22	46.198	Find by Molecular Feature	638.436

Compound Chromatograms

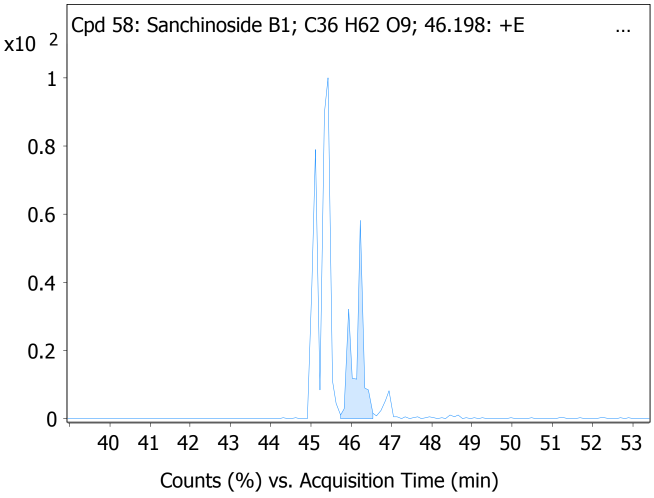

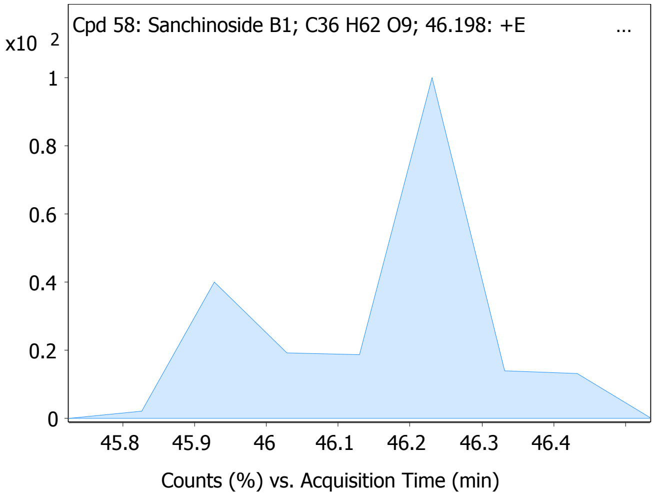

MFE MS Spectrum

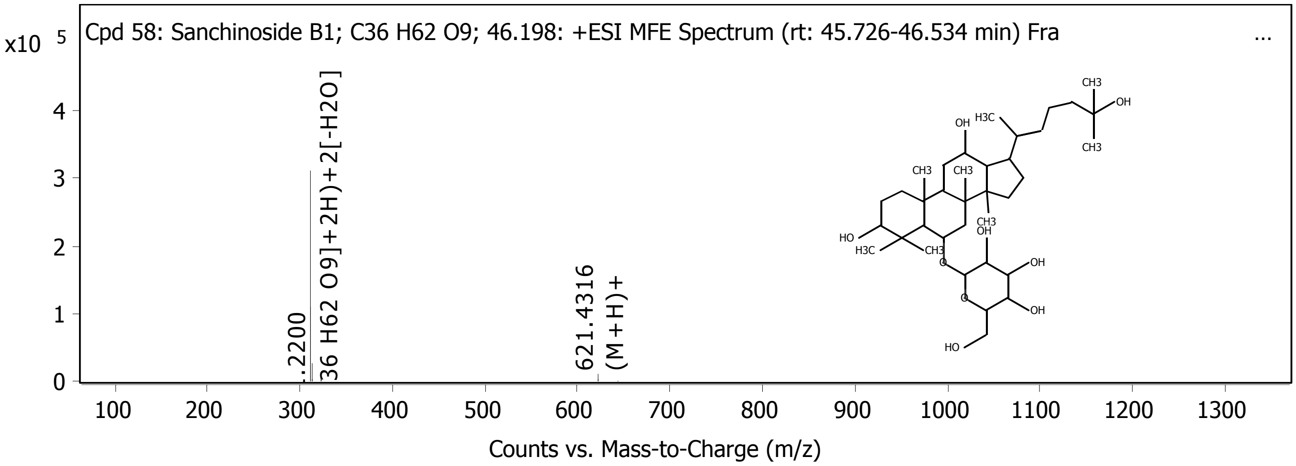

MFE MS Zoomed Spectrum

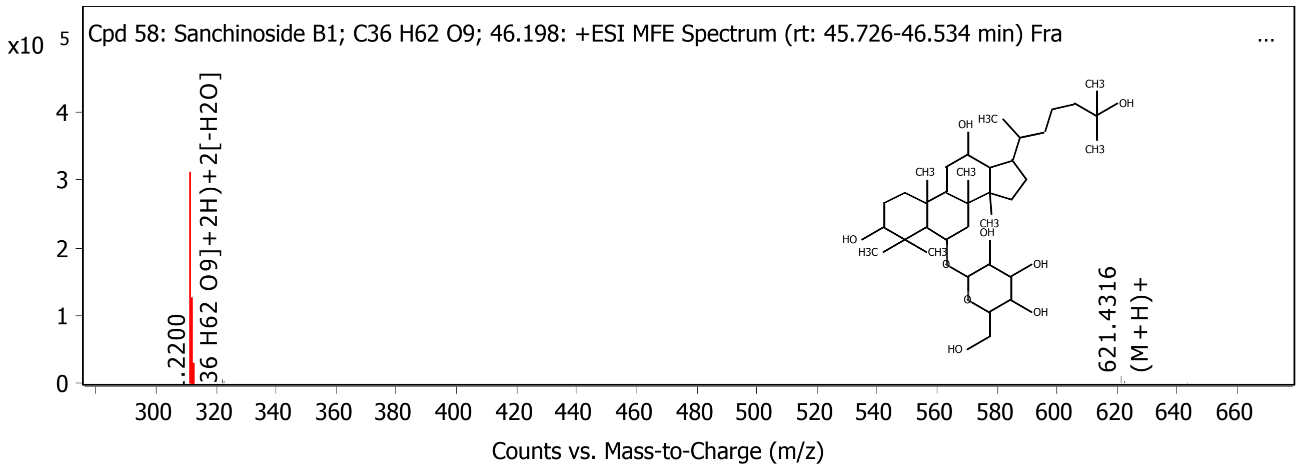

MS Spectrum

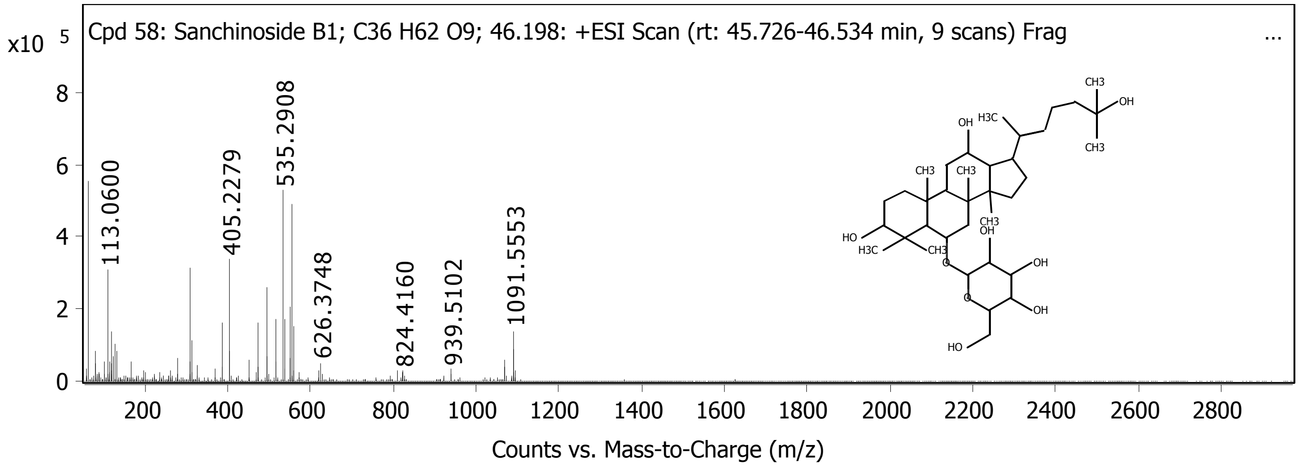

MS Zoomed Spectrum

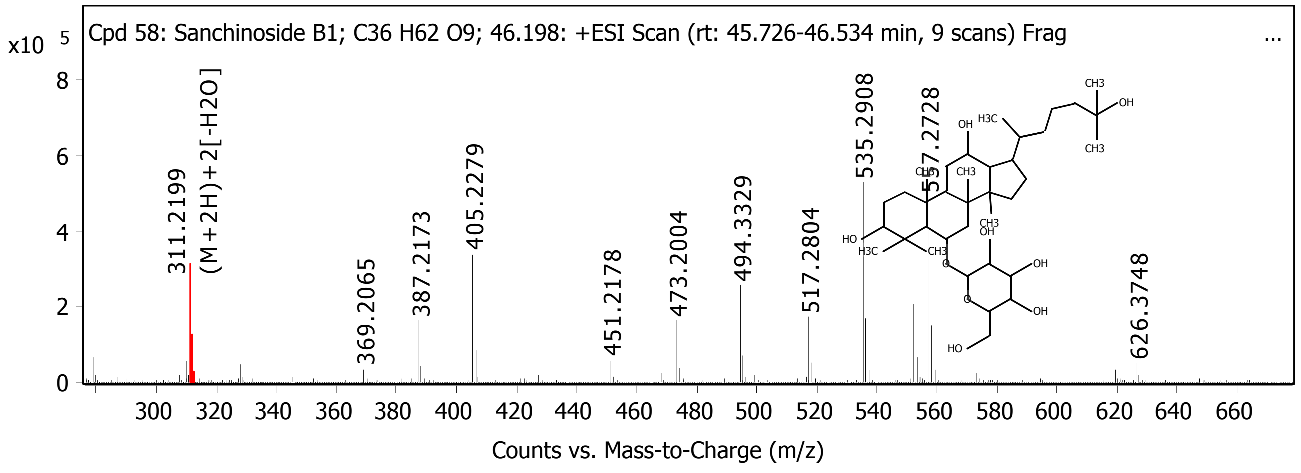

MSMS Spectrum

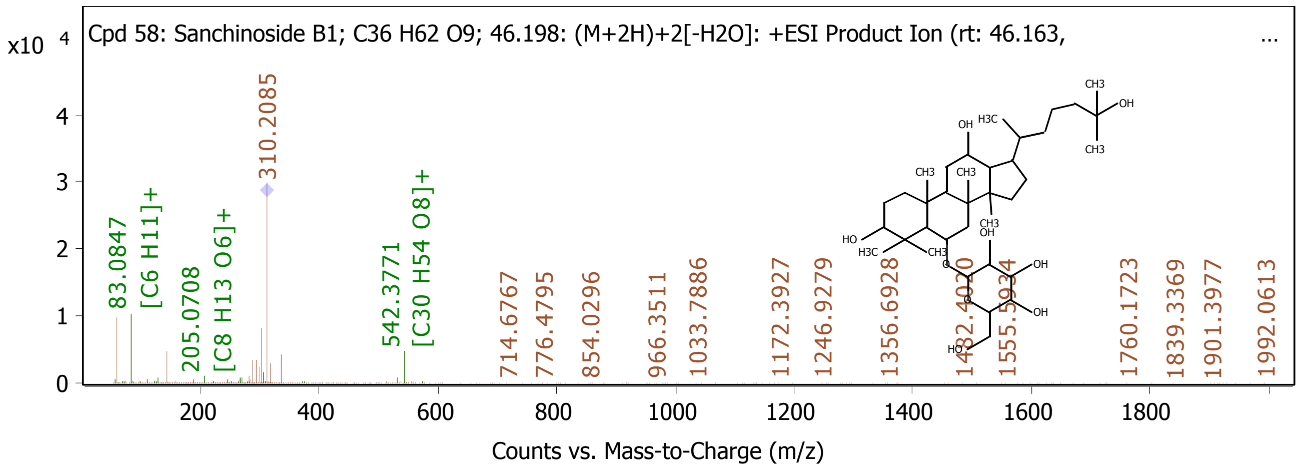

MSMS Spectrum

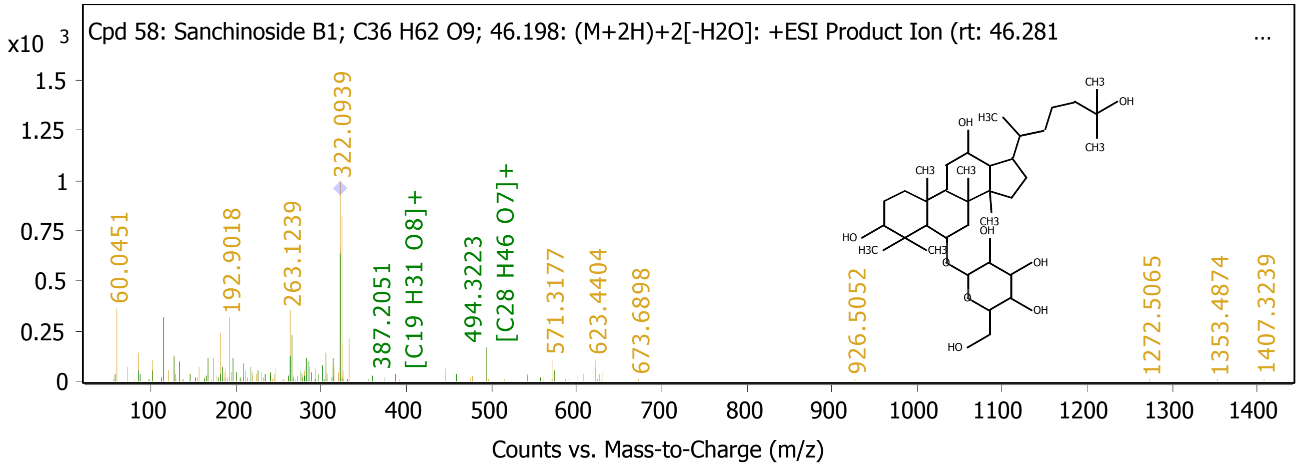

Identification Hit Table

Best Hit	Compound Name	RT	Formula	Notes	Match Score	Mass	Difference	Ion Species
✓	Sanchinoside B1	46.198	C36 H62 O9		83.03	638.436	3.37	(M+2H)+2[-H2O]
	Ginsenoside Rh1	46.198	C36 H62 O9		83.03	638.436	3.37	(M+2H)+2[-H2O]
	Ginsenoside F1	46.198	C36 H62 O9		83.03	638.436	3.37	(M+2H)+2[-H2O]
	Fasciculic acid A	46.198	C36 H60 O8		82.58	620.4254	3.37	(M+2H)+2
	(3b,7b,22x)-Cucurbita-5,24-diene-3,7,23-triol 7-glucoside	46.198	C36 H60 O8		82.58	620.4254	3.37	(M+2H)+2
	Soyasapogenol B 24-O-b-D-glucoside	46.198	C36 H60 O8		82.58	620.4254	3.37	(M+2H)+2
	Ginsenoside Rh4	46.198	C36 H60 O8		82.58	620.4254	3.37	(M+2H)+2
	PC(10:0/14:0)[U]	46.198	C32 H65 N O8 P		11.33	311.0283	311416.45	M+
	PC(9:0/15:0)[U]	46.198	C32 H65 N O8 P		11.33	311.0283	311416.45	M+
	PC(6:0/18:0)	46.198	C32 H65 N O8 P		11.33	311.0283	311416.45	M+

Identification Hit Table

Best Hit	Compound Name	RT	Formula	Notes	Match Score	Mass	Difference	Ion Species
✓	PC(10:0/14:0)[U]	46.198	C32 H65 N O8 P		11.33	311.0283	311416.45	M+
	PC(11:0/13:0)[U]	46.198	C32 H65 N O8 P		11.33	311.0283	311416.45	M+
	PC(12:0/12:0)	46.198	C32 H65 N O8 P		11.33	311.0283	311416.45	M+
	PC(12:0/12:0)[U]	46.198	C32 H65 N O8 P		11.33	311.0283	311416.45	M+
	PC(13:0/11:0)[U]	46.198	C32 H65 N O8 P		11.33	311.0283	311416.45	M+
	PC(14:0/10:0)[U]	46.198	C32 H65 N O8 P		11.33	311.0283	311416.45	M+
	PC(15:0/9:0)[U]	46.198	C32 H65 N O8 P		11.33	311.0283	311416.45	M+
	PC(18:0/6:0)[U]	46.198	C32 H65 N O8 P		11.33	311.0283	311416.45	M+
	PC(6:0/18:0)	46.198	C32 H65 N O8 P		11.33	311.0283	311416.45	M+
	PC(9:0/15:0)[U]	46.198	C32 H65 N O8 P		11.33	311.0283	311416.45	M+

Identification Hit Table

Best Hit	Compound Name	RT	Formula	Notes	Match Score	Mass	Difference	Ion Species

Compound Label	Name	*m/z*	RT	Algorithm	Mass
Cpd 59: Pro Ile Lys Arg; C23 H44 N8 O5; 46.250	**Pro Ile Lys Arg**	494.333	46.25	Find by Molecular Feature	512.344

Compound Chromatograms

Qualitative Compound Identification Report

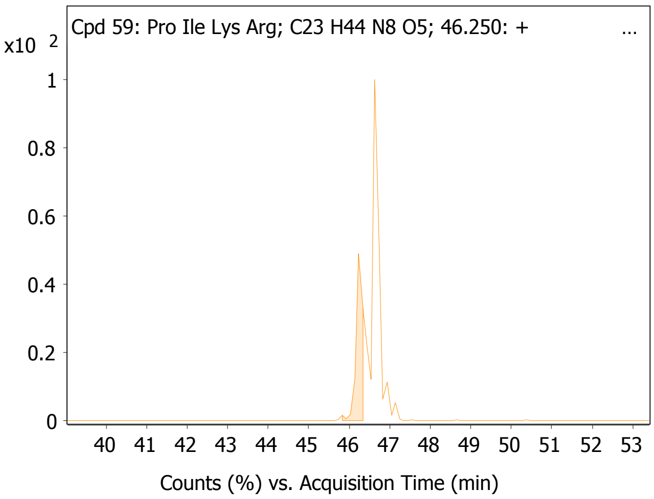

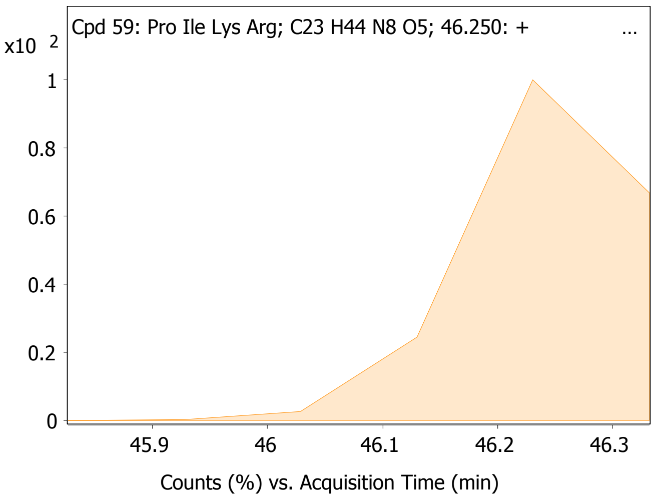

MFE MS Spectrum

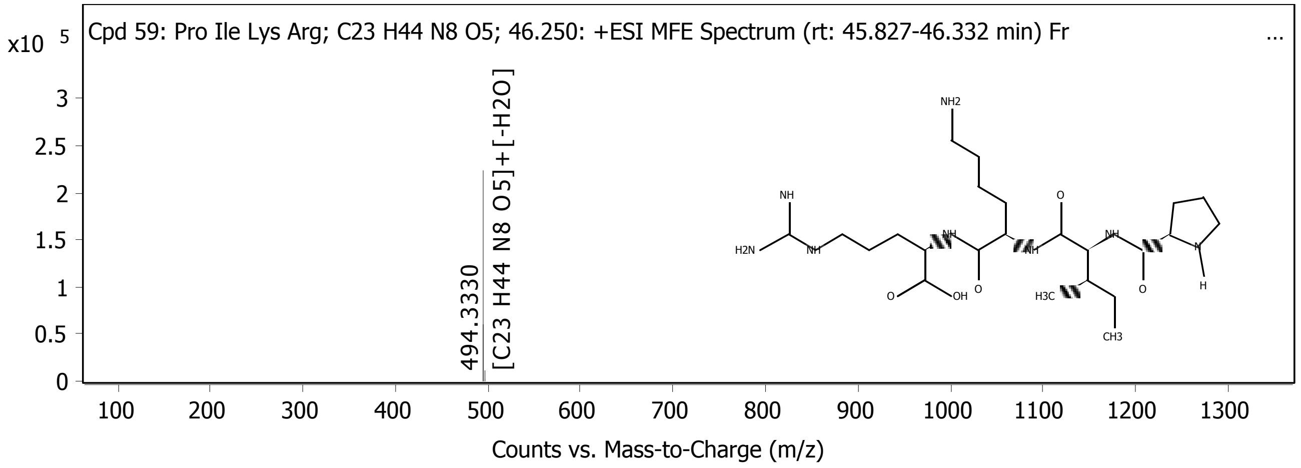

MFE MS Zoomed Spectrum

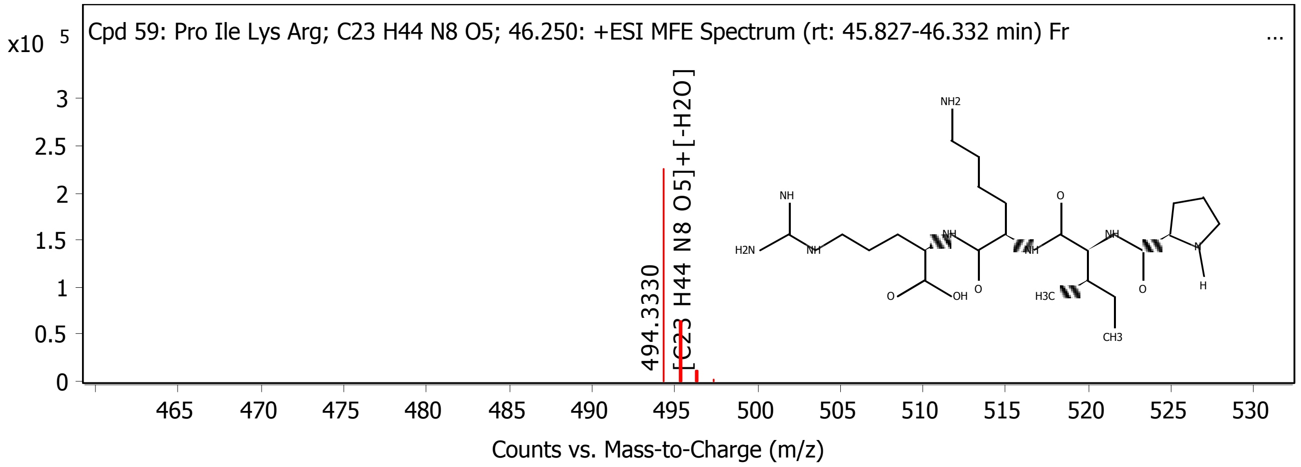

MS Spectrum

Qualitative Compound Identification Report

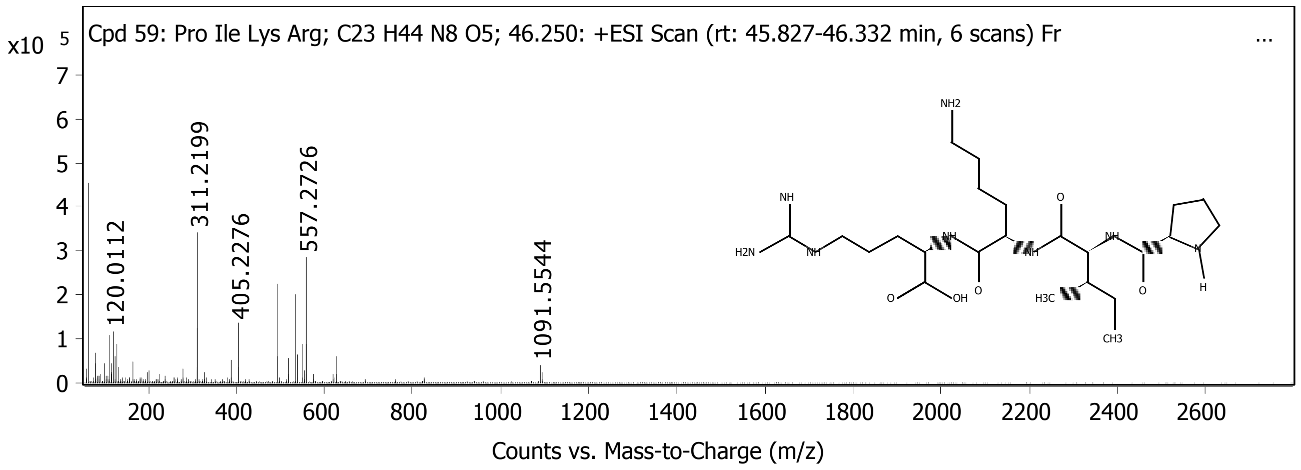

MS Zoomed Spectrum

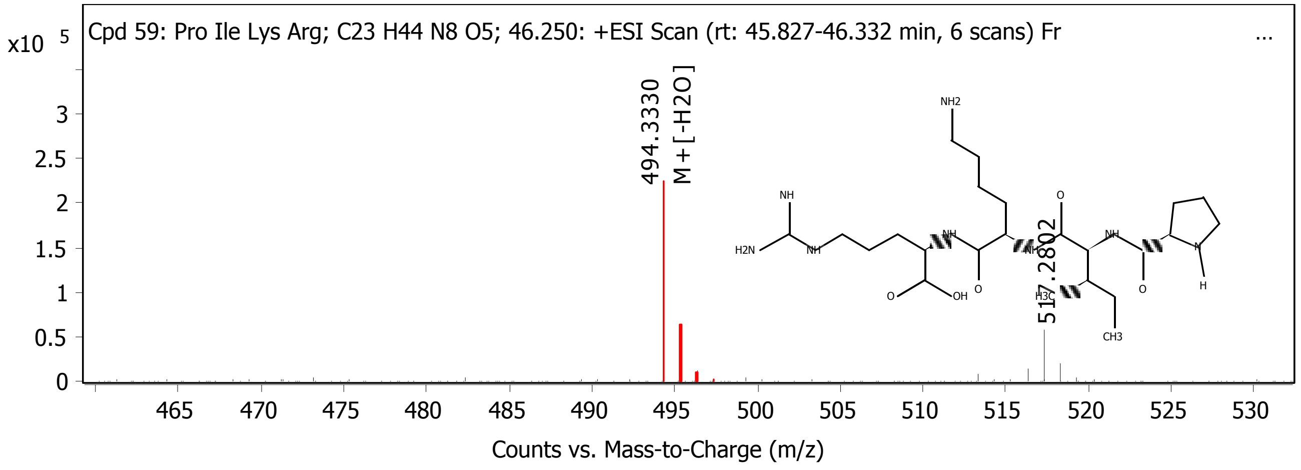

Identification Hit Table

Best Hit	Compound Name	RT	Formula	Notes	Match Score	Mass	Difference	Ion Species
✓	Tiamulin	46.25	C28 H47 N O4 S	Pubchem 656958	75.62	493.3257	-3.14	(M+H)+
	PA(22:0/0:0)	46.25	C25 H51 O7 P		75.38	494.3333	3.94	M+
	Scyphostatin A	46.25	C31 H45 N O5		53.03	511.336	-6.27	(M+H)+[-H2O]
	Norselic acid E	46.25	C31 H42 O5		52.43	494.3095	-6.27	(M+NH4)+[-H2O]
	4-O-alpha-Cadinylangolensin	46.25	C31 H40 O4		51.82	476.2989	-6.27	(M+NH4)+
	ZK 159222	46.25	C32 H48 O5		51.5	512.3438	6.33	M+[-H2O]
	11-Oxoursolic acid acetate	46.25	C32 H48 O5	Bursera delpechiana	51.5	512.3438	6.33	M+[-H2O]
	(+)-Ganoderic acid S	46.25	C32 H48 O5		51.5	512.3438	6.33	M+[-H2O]
	Acetoxolone	46.25	C32 H48 O5		51.5	512.3438	6.33	M+[-H2O]
	Acetyl-11-keto-B-Boswellic Acid, 3-	46.25	C32 H48 O5		51.5	512.3438	6.33	M+[-H2O]

Identification Hit Table

Best Hit	Compound Name	RT	Formula	Notes	Match Score	Mass	Difference	Ion Species
✓	PA(22:0/0:0)	46.25	C25 H51 O7 P		75.38	494.3333	3.94	M+
	Scyphostatin A	46.25	C31 H45 N O5		53.03	511.336	-6.27	(M+H)+[-H2O]
	Norselic acid E	46.25	C31 H42 O5		52.43	494.3095	-6.27	(M+NH4)+[-H2O]
	4-O-alpha-Cadinylangolensin	46.25	C31 H40 O4		51.82	476.2989	-6.27	(M+NH4)+
	(+)-Ganoderic acid S	46.25	C32 H48 O5		51.5	512.3438	6.33	M+[-H2O]
	ZK 159222	46.25	C32 H48 O5		51.5	512.3438	6.33	M+[-H2O]
	1α-hydroxy-18-[m-(1-hydroxy-1-methylethyl)-benzyloxy]-23,24,25,26,27-pentanorvitamin D3 / 1α-hydroxy-18-[m-(1-hydroxy-1-methylethyl)-benzyloxy]-23,24,25,26,27-pentanorcholecalciferol	46.25	C32 H46 O4		50.89	494.3333	6.33	M+
	Atocalcitol	46.25	C32 H46 O4		50.89	494.3333	6.33	M+

Identification Hit Table

Best Hit	Compound Name	RT	Formula	Notes	Match Score	Mass	Difference	Ion Species
✓	Pro Ile Lys Arg	46.25	C23 H44 N8 O5		98.63	512.344	-0.57	M+[-H2O]
	Lys Pro Arg Leu	46.25	C23 H44 N8 O5		98.63	512.344	-0.57	M+[-H2O]
	Ile Lys Pro Arg	46.25	C23 H44 N8 O5		98.63	512.344	-0.57	M+[-H2O]
	Leu Arg Lys Pro	46.25	C23 H44 N8 O5		98.63	512.344	-0.57	M+[-H2O]
	Leu Pro Arg Lys	46.25	C23 H44 N8 O5		98.63	512.344	-0.57	M+[-H2O]
	Leu Pro Lys Arg	46.25	C23 H44 N8 O5		98.63	512.344	-0.57	M+[-H2O]
	Leu Lys Arg Pro	46.25	C23 H44 N8 O5		98.63	512.344	-0.57	M+[-H2O]
	Arg Pro Leu Lys	46.25	C23 H44 N8 O5		98.63	512.344	-0.57	M+[-H2O]
	Lys Arg Pro Leu	46.25	C23 H44 N8 O5		98.63	512.344	-0.57	M+[-H2O]
	Lys Arg Pro Ile	46.25	C23 H44 N8 O5		98.63	512.344	-0.57	M+[-H2O]

Compound Label	Name	m/z	RT	Algorithm	Mass
Cpd 60: Phe Thr Thr Thr; C21 H32 N4 O8; 46.603	**Phe Thr Thr Thr**	473.2003	46.603	Find by Molecular Feature	468.2216

Compound Chromatograms

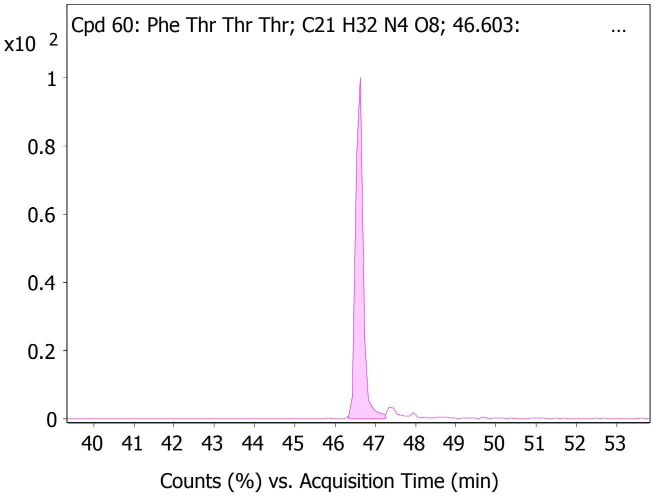

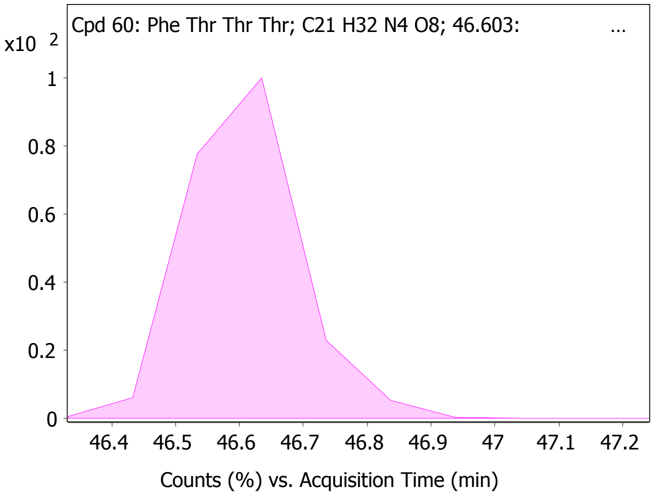

MFE MS Spectrum

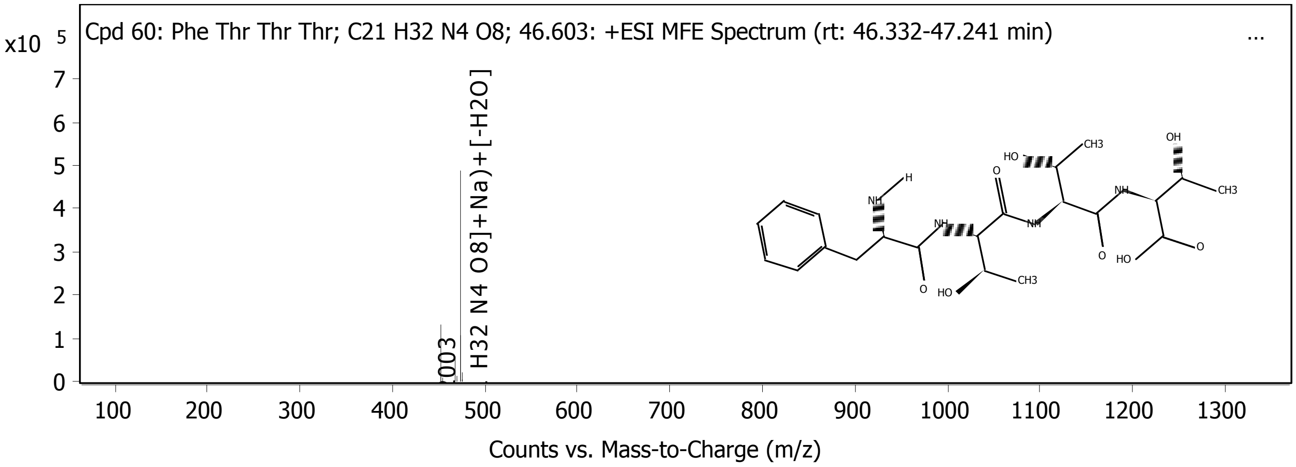

MFE MS Zoomed Spectrum

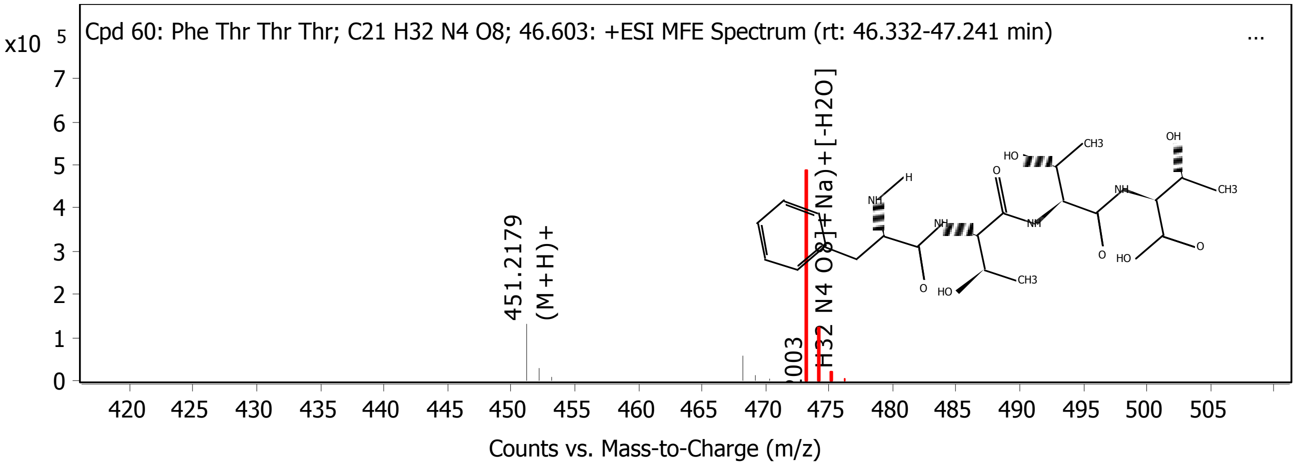

MS Spectrum

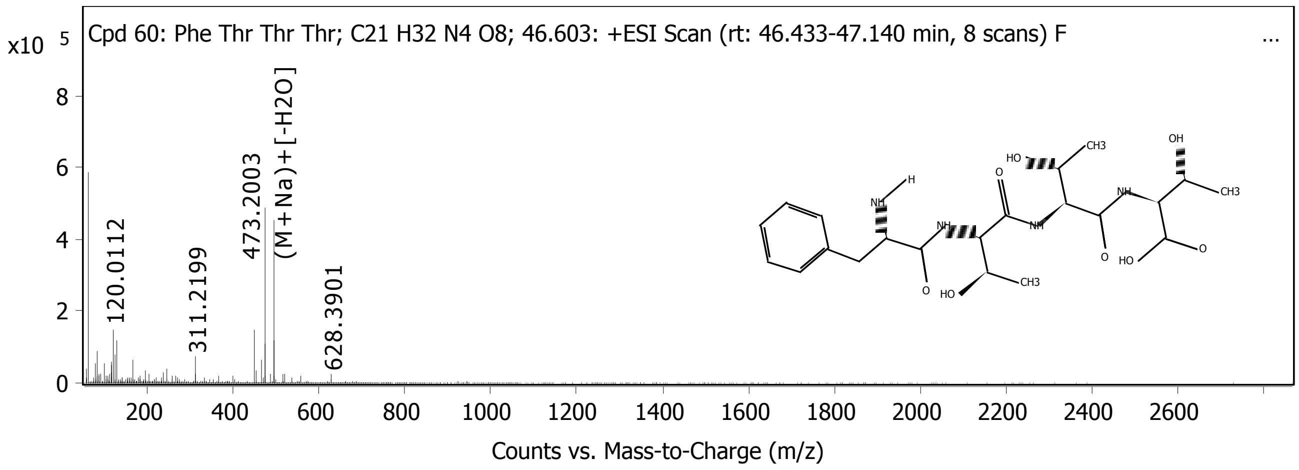

MS Zoomed Spectrum

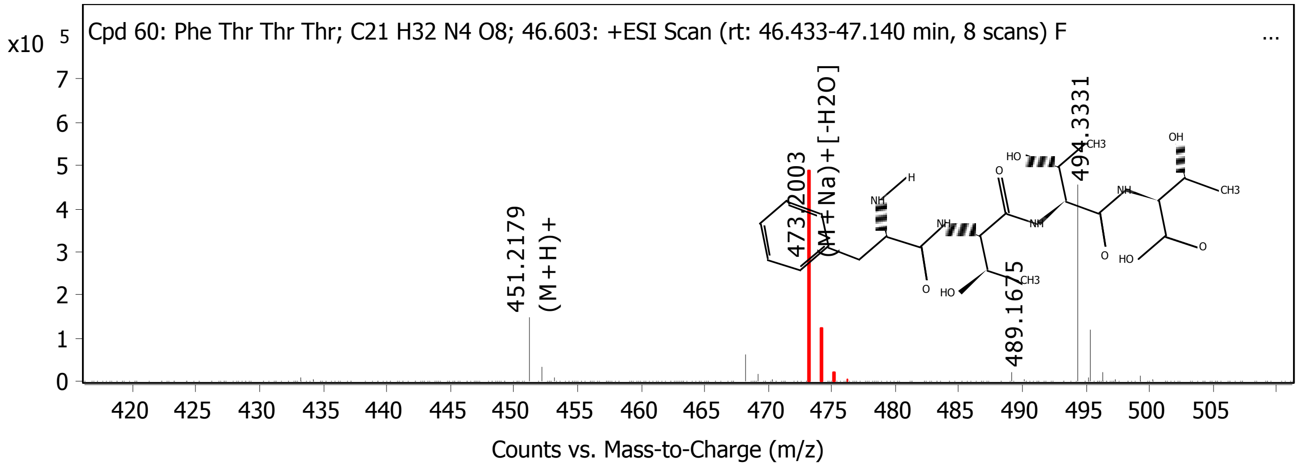

MSMS Spectrum

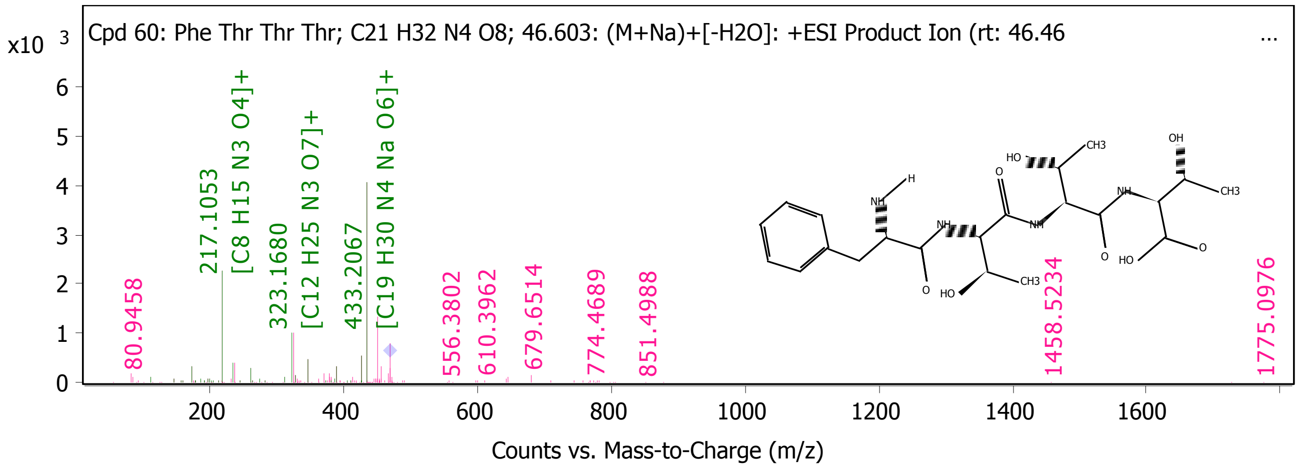

Identification Hit Table

Best Hit	Compound Name	RT	Formula	Notes	Match Score	Mass	Difference	Ion Species
✓	Rivenprost	46.603	C24 H34 O6 S		69.34	450.2112	-3.55	(M+Na)+
	6β-Hydroxytriamcinolone acetonide	46.603	C24 H31 F O7	Glucocorticoid, Treatment of autoimmune and allergic conditions Metabolite of Triamcinolone acetonide Dollery, Colin Therapeutic Drugs, 2nd Ed. 1999 p. T165	58.7	450.2109	-5.55	(M+Na)+
	Tyr Met Arg	46.603	C20 H32 N6 O5 S		53.54	468.2219	-6.37	(M+Na)+[-H2O]
	Tyr Arg Met	46.603	C20 H32 N6 O5 S		53.54	468.2219	-6.37	(M+Na)+[-H2O]
	Met Arg Tyr	46.603	C20 H32 N6 O5 S		53.54	468.2219	-6.37	(M+Na)+[-H2O]
	Met Tyr Arg	46.603	C20 H32 N6 O5 S		53.54	468.2219	-6.37	(M+Na)+[-H2O]
	Arg Tyr Met	46.603	C20 H32 N6 O5 S		53.54	468.2219	-6.37	(M+Na)+[-H2O]

	Arg Met Tyr	46.603	C20 H32 N6 O5 S		53.54	468.2219	-6.37	(M+Na)+[-H2O]
	D-Phe-Pro-Arg-CH2Cl	46.603	C21 H31 Cl N6 O3		49.13	450.2114	3.26	(M+Na)+
	Andirobin	46.603	C27 H32 O7	Carapa guayanensis	48.77	468.2215	-6.69	(M+Na)+[-H2O]

Identification Hit Table

Best Hit	Compound Name	RT	Formula	Notes	Match Score	Mass	Difference	Ion Species
✓	1'',2''-Dihydro-8-hydroxyisopentanyl-3'-methoxy-4'-O-methylalpinumisoflavone	46.603	C27 H32 O7		48.77	468.2215	-6.69	(M+Na)+[-H2O]
	1'',2''-Dihydro-8-hydroxyisopentanyl-2'-methoxy-4'-O-methylalpinumisoflavone	46.603	C27 H32 O7		48.77	468.2215	-6.69	(M+Na)+[-H2O]
	Exiguaflavanone E	46.603	C27 H32 O7		48.77	468.2215	-6.69	(M+Na)+[-H2O]
	Glabrescione B	46.603	C27 H30 O6		48.24	450.2109	-6.69	(M+Na)+

Identification Hit Table

Best Hit	Compound Name	RT	Formula	Notes	Match Score	Mass	Difference	Ion Species
✓	Phe Thr Thr Thr	46.603	C21 H32 N4 O8		97.93	468.2216	0.42	(M+Na)+[-H2O]
	Ser Ser Tyr Ile	46.603	C21 H32 N4 O8		97.93	468.2216	0.42	(M+Na)+[-H2O]
	Ser Tyr Val Thr	46.603	C21 H32 N4 O8		97.93	468.2216	0.42	(M+Na)+[-H2O]
	Ser Tyr Ser Leu	46.603	C21 H32 N4 O8		97.93	468.2216	0.42	(M+Na)+[-H2O]
	Ser Tyr Ser Ile	46.603	C21 H32 N4 O8		97.93	468.2216	0.42	(M+Na)+[-H2O]
	Ser Tyr Leu Ser	46.603	C21 H32 N4 O8		97.93	468.2216	0.42	(M+Na)+[-H2O]
	Ser Tyr Ile Ser	46.603	C21 H32 N4 O8		97.93	468.2216	0.42	(M+Na)+[-H2O]
	Ser Val Tyr Thr	46.603	C21 H32 N4 O8		97.93	468.2216	0.42	(M+Na)+[-H2O]
	Ser Val Thr Tyr	46.603	C21 H32 N4 O8		97.93	468.2216	0.42	(M+Na)+[-H2O]
	Ser Thr Tyr Val	46.603	C21 H32 N4 O8		97.93	468.2216	0.42	(M+Na)+[-H2O]

Compound Label	Name	*m/z*	RT	Algorithm	Mass
Cpd 61: Pro Ile Lys Arg; C23 H44 N8 O5; 46.644	**Pro Ile Lys Arg**	494.3332	46.644	Find by Molecular Feature	512.3443

Compound Chromatograms

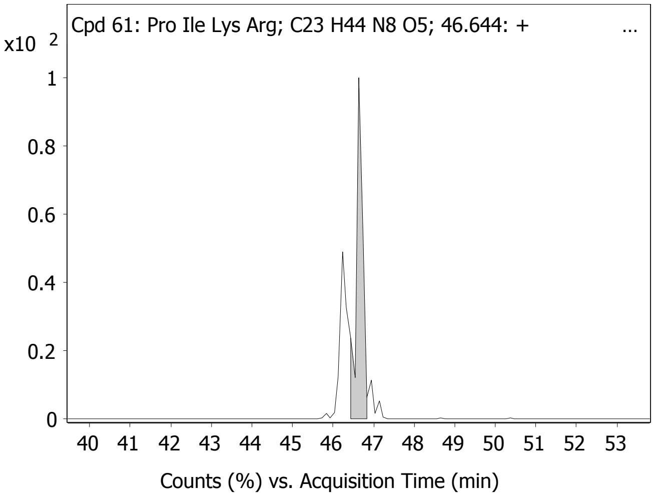

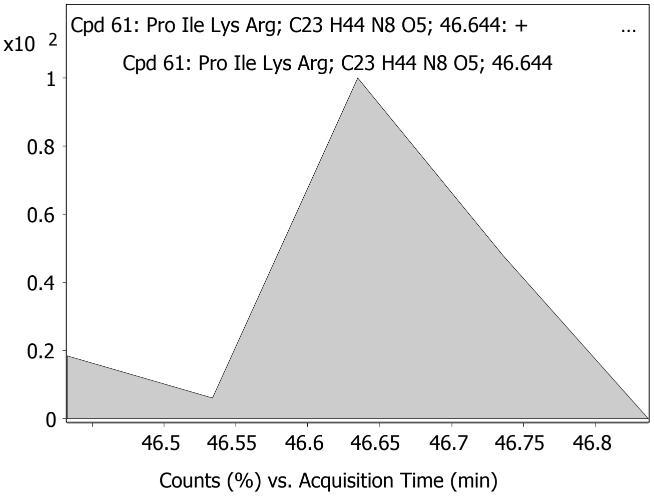

MFE MS Spectrum

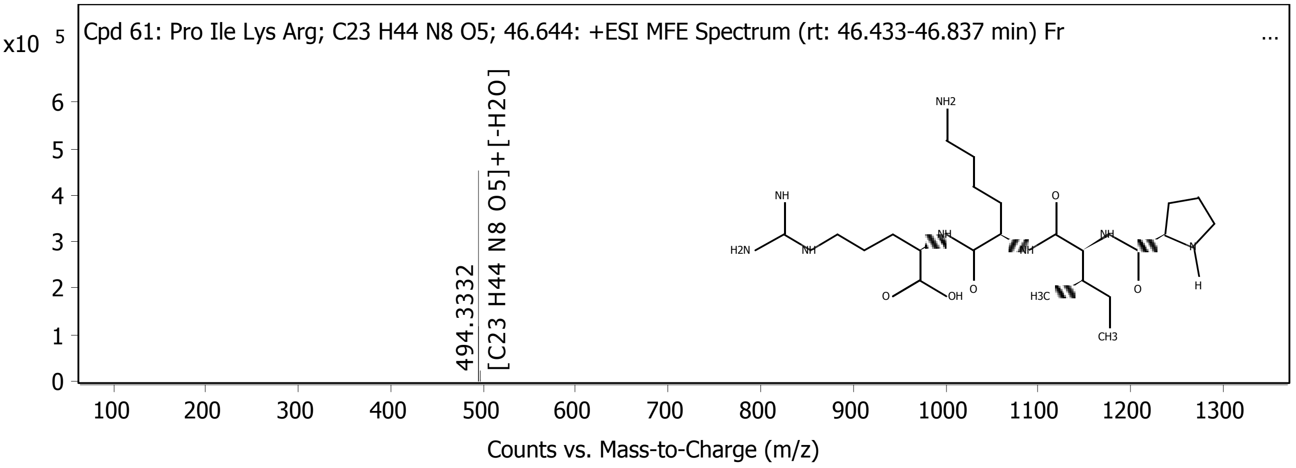

MFE MS Zoomed Spectrum

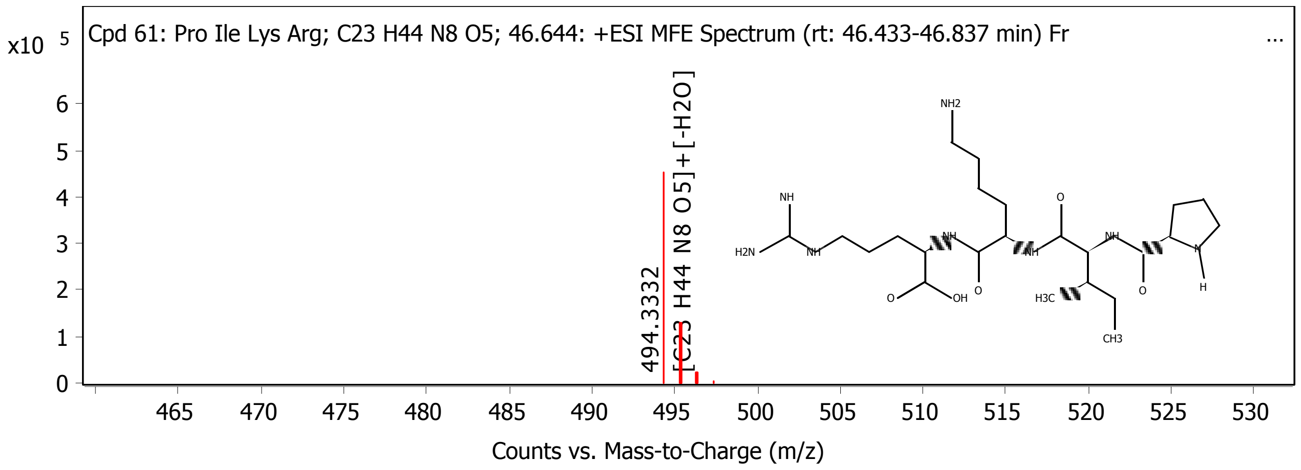

MS Spectrum

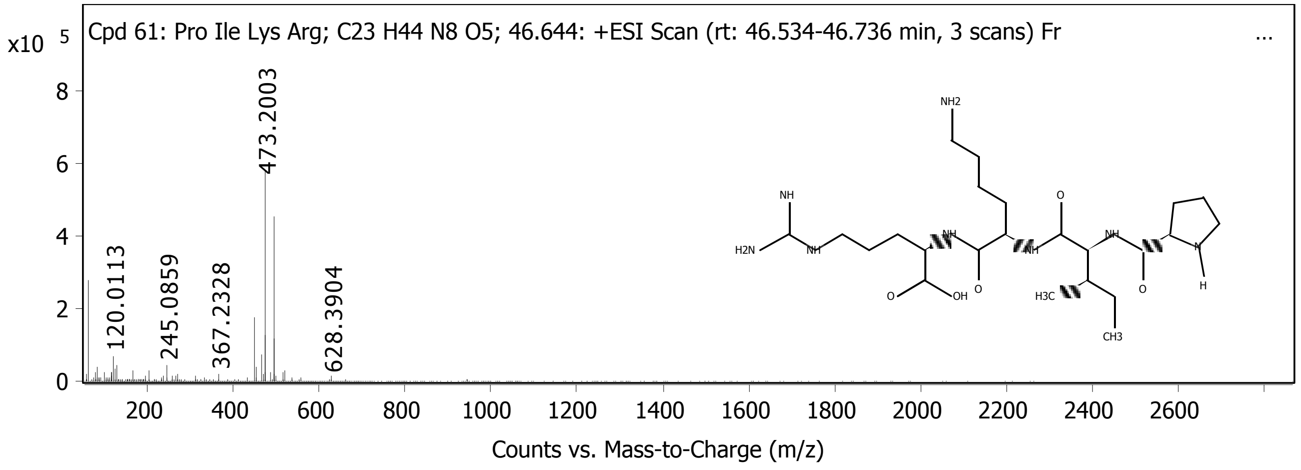

MS Zoomed Spectrum

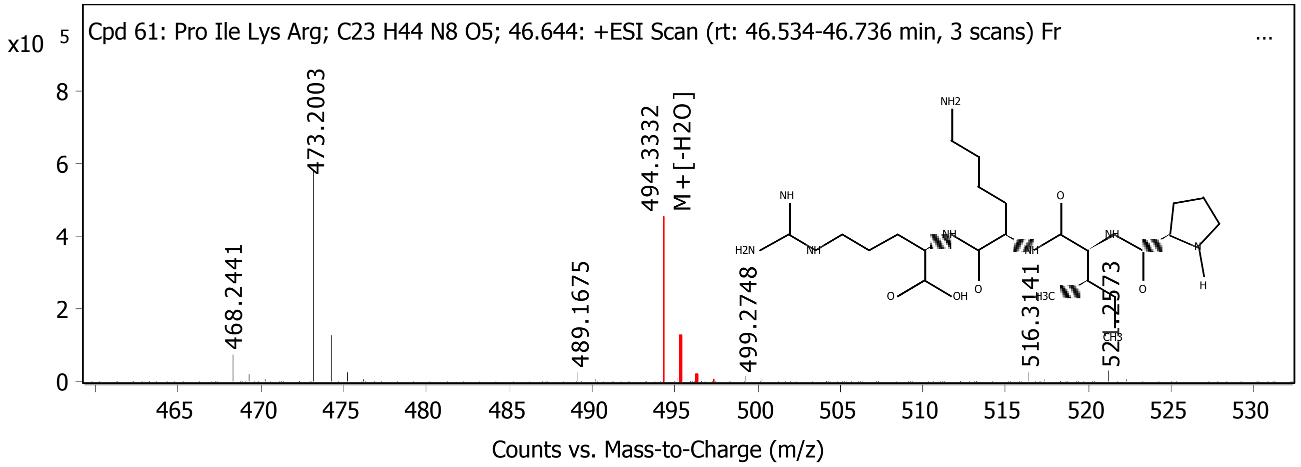

Identification Hit Table

Best Hit	Compound Name	RT	Formula	Notes	Match Score	Mass	Difference	Ion Species
✓	PA(22:0/0:0)	46.644	C25 H51 O7 P		78.22	494.3336	3.66	M+
	Tiamulin	46.644	C28 H47 N O4 S	Pubchem 656958	71.75	493.326	-3.41	(M+H)+
	(+)-Ganoderic acid S	46.644	C32 H48 O5		52.63	512.3441	6.04	M+[-H2O]
	Ganoderic acid X	46.644	C32 H48 O5		52.63	512.3441	6.04	M+[-H2O]
	Ganoderic acid Mf	46.644	C32 H48 O5		52.63	512.3441	6.04	M+[-H2O]
	Ganoderic acid S	46.644	C32 H48 O5		52.63	512.3441	6.04	M+[-H2O]
	3beta-Acetoxy-12-oxo-28,13beta-oleananolide	46.644	C32 H48 O5		52.63	512.3441	6.04	M+[-H2O]
	11a,12a-Epoxy-3b-hydroxy-28,13-oleananolide 3-acetate	46.644	C32 H48 O5		52.63	512.3441	6.04	M+[-H2O]
	Acetyl-11-keto-B-Boswellic Acid, 3-	46.644	C32 H48 O5		52.63	512.3441	6.04	M+[-H2O]

	Acetoxolone	46.644	C32 H48 O5		52.63	512.3441	6.04	M+[-H2O]

Identification Hit Table

Best Hit	Compound Name	RT	Formula	Notes	Match Score	Mass	Difference	Ion Species
✓	PA(22:0/0:0)	46.644	C25 H51 O7 P		78.22	494.3336	3.66	M+
	(+)-Ganoderic acid S	46.644	C32 H48 O5		52.63	512.3441	6.04	M+[-H2O]
	ZK 159222	46.644	C32 H48 O5		52.63	512.3441	6.04	M+[-H2O]
	1α-hydroxy-18-[m-(1-hydroxy-1-methylethyl)-benzyloxy]-23,24,25,26,27-pentanorvitamin D3 / 1α-hydroxy-18-[m-(1-hydroxy-1-methylethyl)-benzyloxy]-23,24,25,26,27-pentanorcholecalciferol	46.644	C32 H46 O4		51.98	494.3336	6.04	M+
	Atocalcitol	46.644	C32 H46 O4		51.98	494.3336	6.04	M+
	Scyphostatin A	46.644	C31 H45 N O5		51.36	511.3363	-6.56	(M+H)+[-H2O]
	Norselic acid E	46.644	C31 H42 O5		50.81	494.3098	-6.56	(M+NH4)+[-H2O]
	4-O-alpha-Cadinylangolensin	46.644	C31 H40 O4		50.25	476.2992	-6.56	(M+NH4)+

Identification Hit Table

Best Hit	Compound Name	RT	Formula	Notes	Match Score	Mass	Difference	Ion Species
✓	Pro Ile Lys Arg	46.644	C23 H44 N8 O5		97.66	512.3443	-0.85	M+[-H2O]
	Lys Pro Arg Leu	46.644	C23 H44 N8 O5		97.66	512.3443	-0.85	M+[-H2O]
	Ile Lys Pro Arg	46.644	C23 H44 N8 O5		97.66	512.3443	-0.85	M+[-H2O]
	Leu Arg Lys Pro	46.644	C23 H44 N8 O5		97.66	512.3443	-0.85	M+[-H2O]
	Leu Pro Arg Lys	46.644	C23 H44 N8 O5		97.66	512.3443	-0.85	M+[-H2O]
	Leu Pro Lys Arg	46.644	C23 H44 N8 O5		97.66	512.3443	-0.85	M+[-H2O]
	Leu Lys Arg Pro	46.644	C23 H44 N8 O5		97.66	512.3443	-0.85	M+[-H2O]
	Arg Pro Leu Lys	46.644	C23 H44 N8 O5		97.66	512.3443	-0.85	M+[-H2O]
	Lys Arg Pro Leu	46.644	C23 H44 N8 O5		97.66	512.3443	-0.85	M+[-H2O]
	Lys Arg Pro Ile	46.644	C23 H44 N8 O5		97.66	512.3443	-0.85	M+[-H2O]

Compound Label	Name	*m/z*	RT	Algorithm	Mass
Cpd 62: Mulberrofuran T; C44 H44 O9; 47.356	**Mulberrofuran T**	717.3096	47.356	Find by Molecular Feature	716.3021

Compound Chromatograms

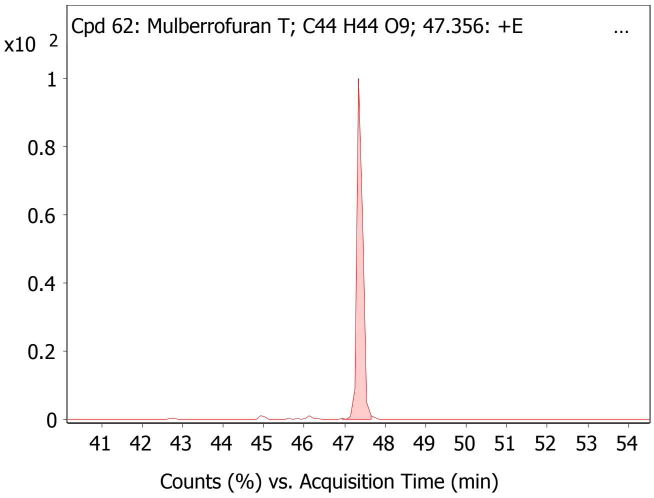

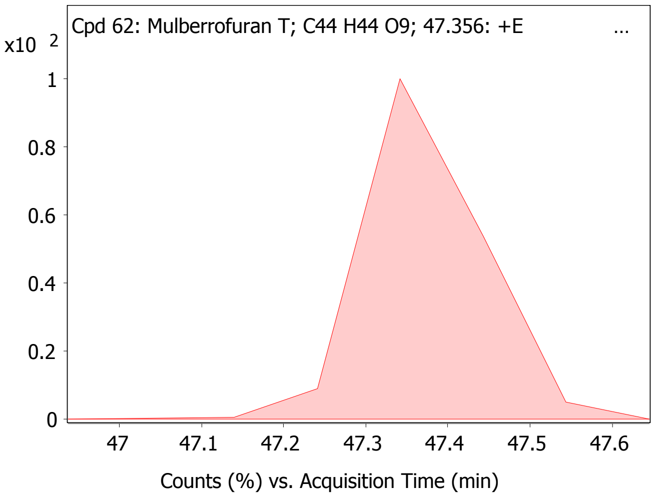

MFE MS Spectrum

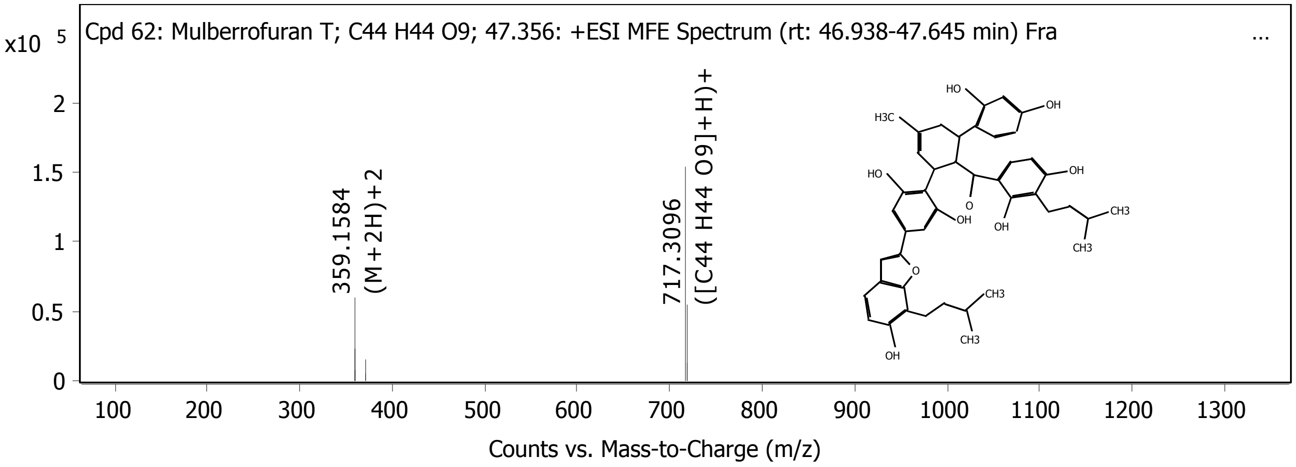

Qualitative Compound Identification Report

MFE MS Zoomed Spectrum

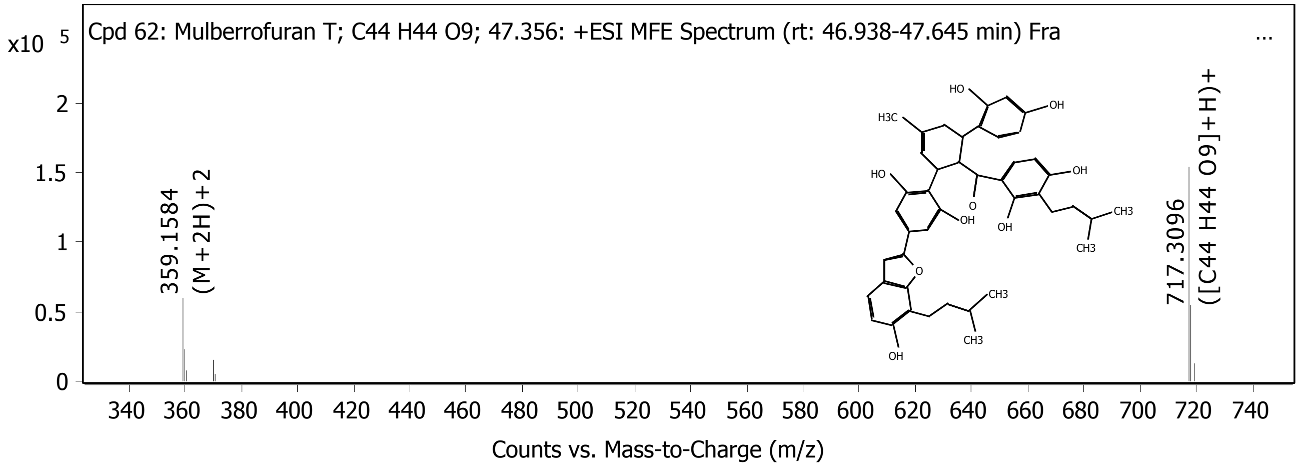

MS Spectrum

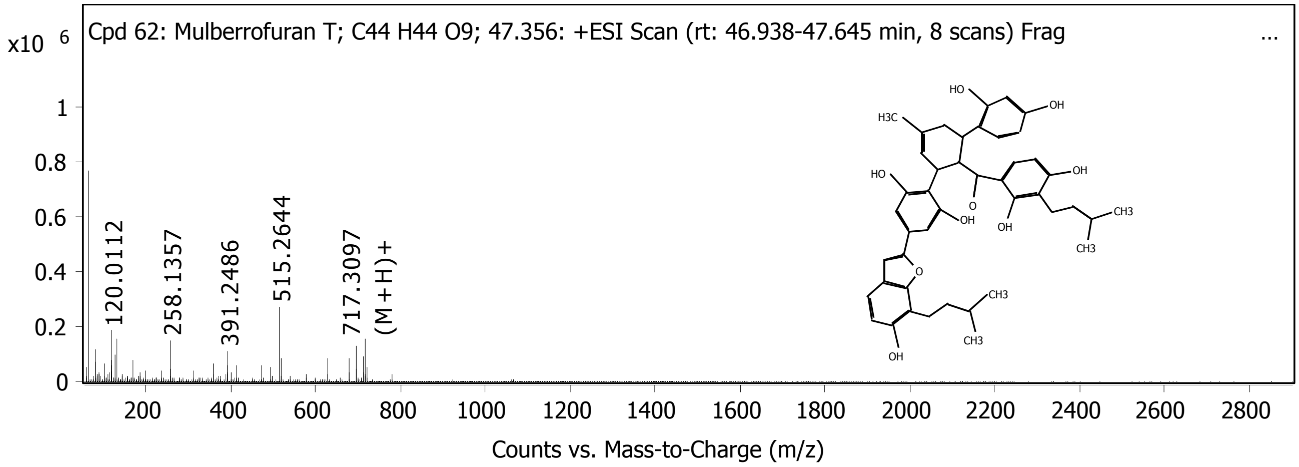

MS Zoomed Spectrum

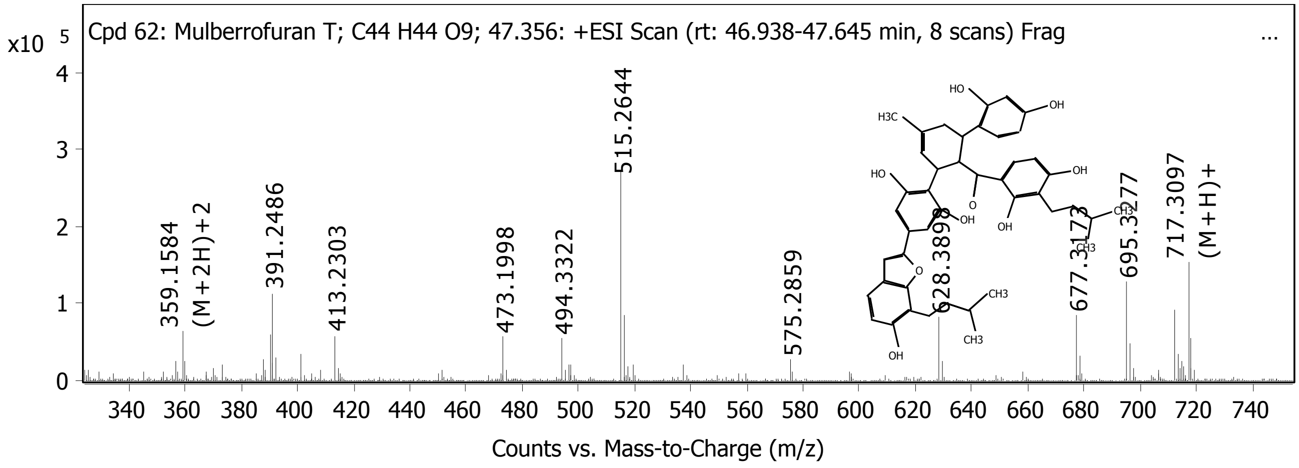

MSMS Spectrum

Agilent Technologies

Qualitative Compound Identification Report

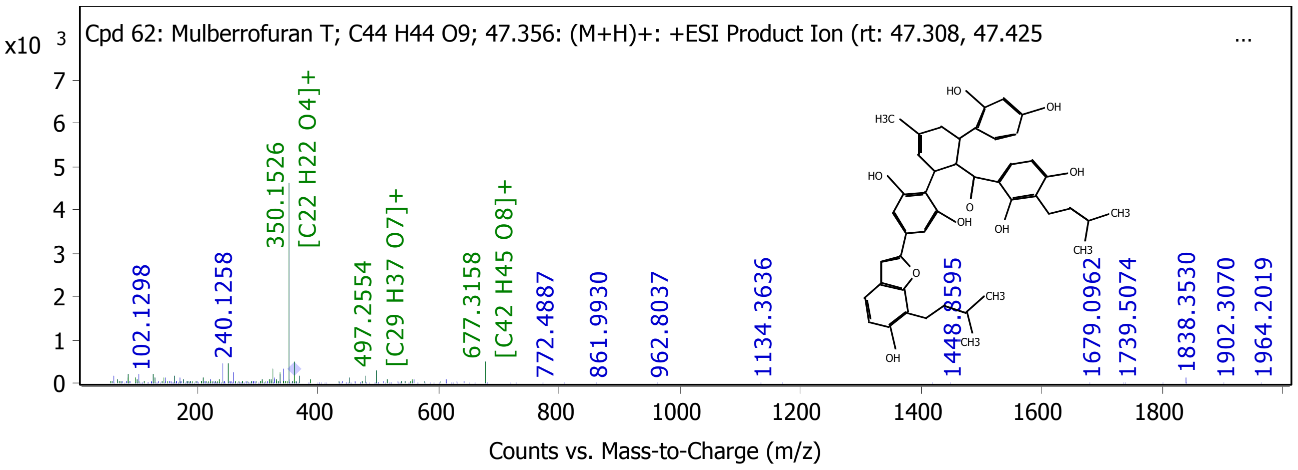

MSMS Spectrum

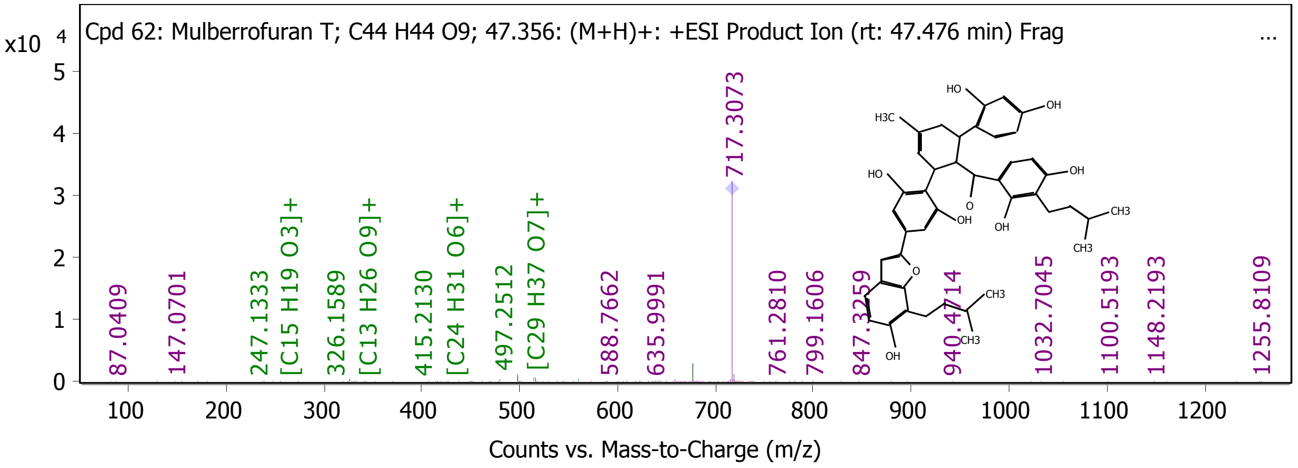

Identification Hit Table

Best Hit	Compound Name	RT	Formula	Notes	Match Score	Mass	Difference	Ion Species
✓	Mulberrofuran T	47.356	C44 H44 O9		73.48	716.3021	-3.57	(M+H)+

Identification Hit Table

Best Hit	Compound Name	RT	Formula	Notes	Match Score	Mass	Difference	Ion Species

Identification Hit Table

Best Hit	Compound Name	RT	Formula	Notes	Match Score	Mass	Difference	Ion Species
✓	Tyr Tyr Trp Trp	47.356	C40 H40 N6 O7		58.66	716.3022	-6.36	(M+H)+
	Tyr Trp Tyr Trp	47.356	C40 H40 N6 O7		58.66	716.3022	-6.36	(M+H)+
	Tyr Trp Trp Tyr	47.356	C40 H40 N6 O7		58.66	716.3022	-6.36	(M+H)+
	Trp Tyr Tyr Trp	47.356	C40 H40 N6 O7		58.66	716.3022	-6.36	(M+H)+
	Trp Tyr Trp Tyr	47.356	C40 H40 N6 O7		58.66	716.3022	-6.36	(M+H)+
	Trp Trp Tyr Tyr	47.356	C40 H40 N6 O7		58.66	716.3022	-6.36	(M+H)+

Compound Label	Name	*m/z*	RT	Algorithm	Mass
Cpd 63: Trp Ser Lys His; C26 H36 N8 O6; 48.243	**Trp Ser Lys His**	561.2526	48.243	Find by Molecular Feature	556.274

Compound Chromatograms

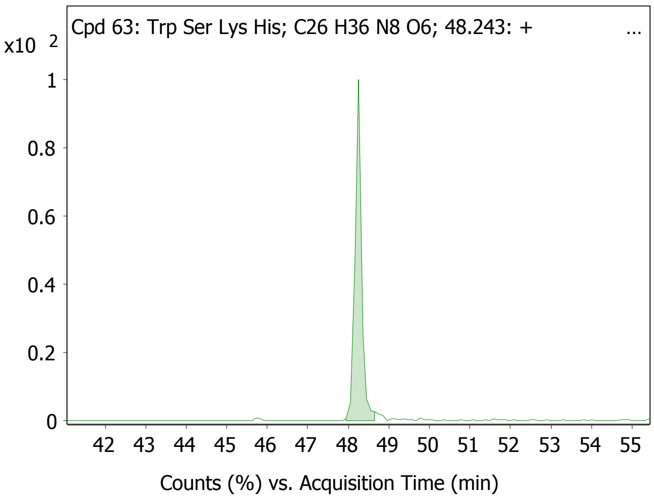

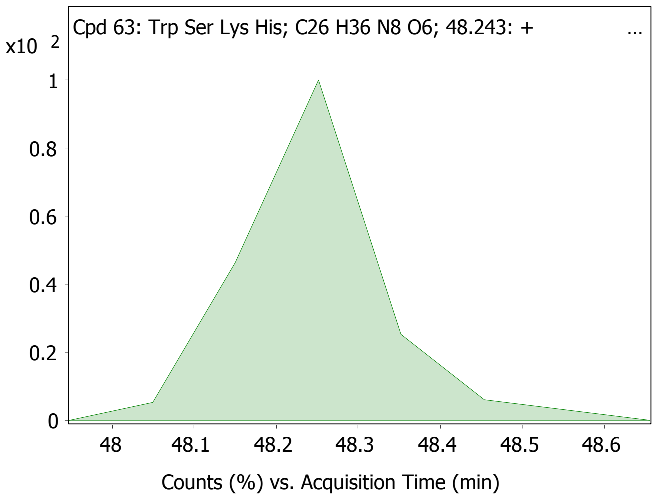

MFE MS Spectrum

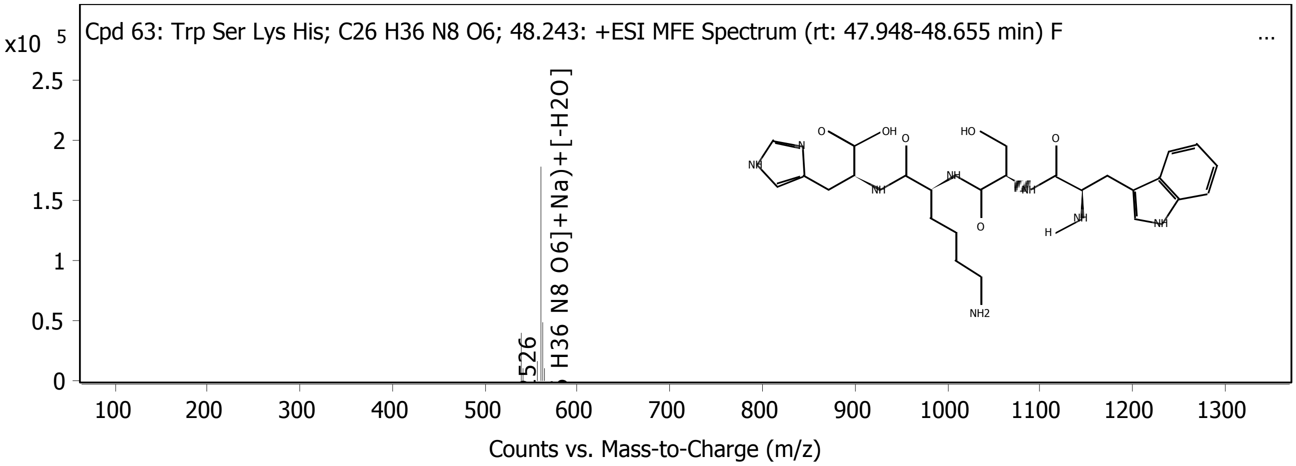

MFE MS Zoomed Spectrum

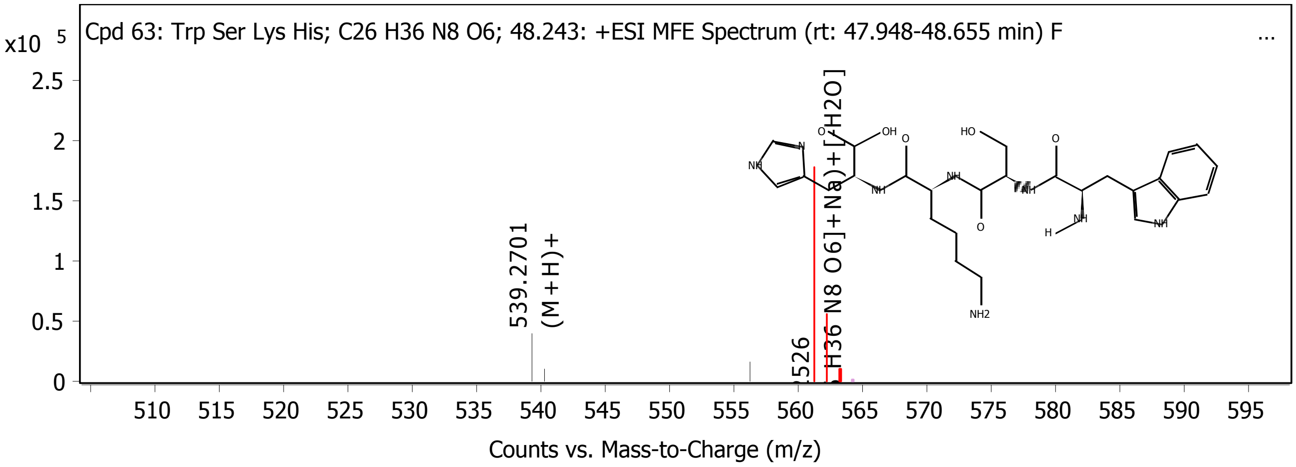

MS Spectrum

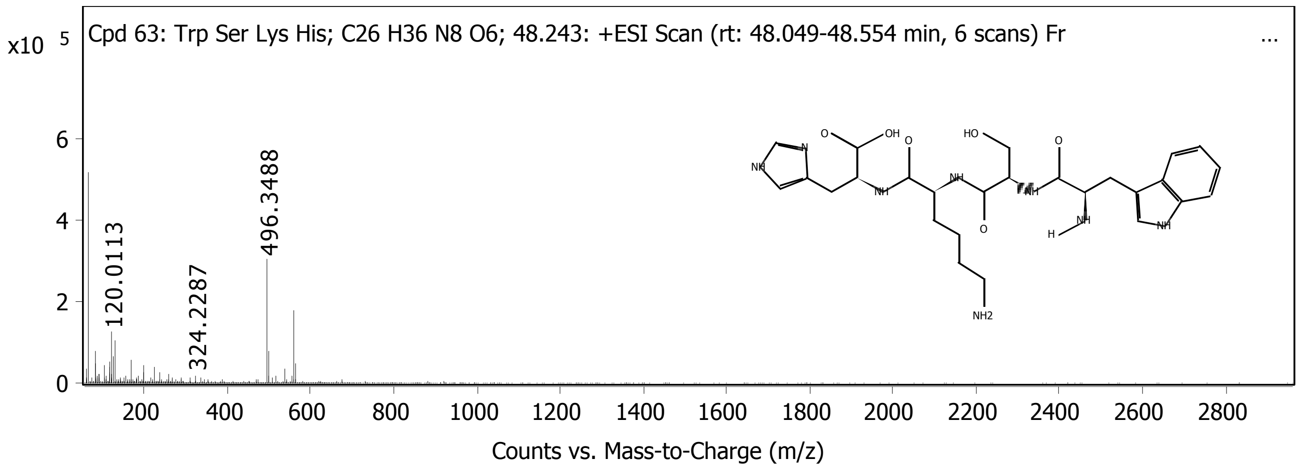

MS Zoomed Spectrum

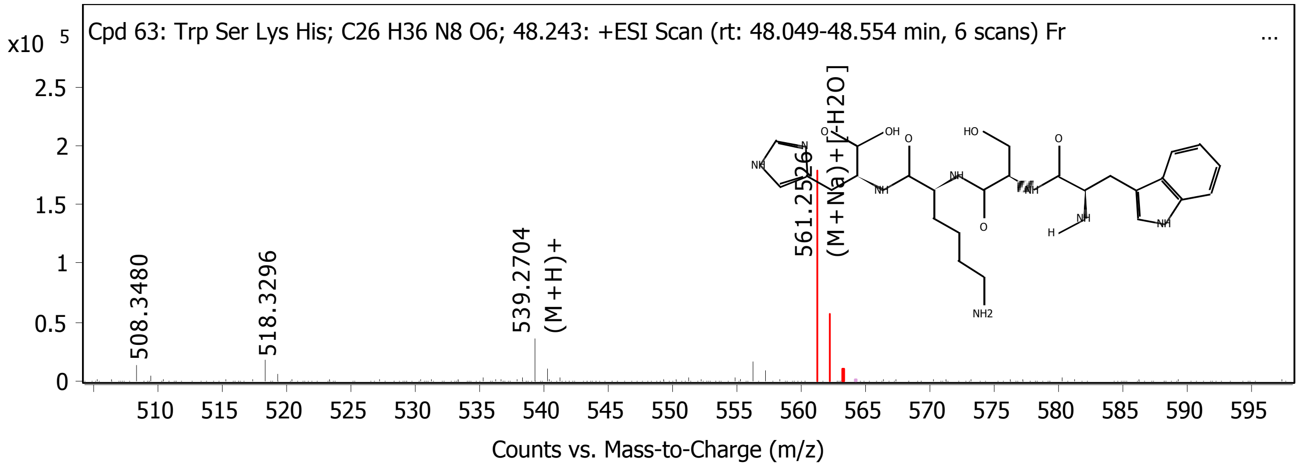

Identification Hit Table

Best Hit	Compound Name	RT	Formula	Notes	Match Score	Mass	Difference	Ion Species
✓	PG(22:6(4Z,7Z,10Z,13Z,16Z,19Z)/0:0)	48.243	C28 H45 O9 P		60.33	556.2738	6.28	(M+Na)+[-H2O]

Identification Hit Table

Best Hit	Compound Name	RT	Formula	Notes	Match Score	Mass	Difference	Ion Species
✓	PG(22:6(4Z,7Z,10Z,13Z,16Z,19Z)/0:0)	48.243	C28 H45 O9 P		60.33	556.2738	6.28	(M+Na)+[-H2O]

Identification Hit Table

Best Hit	Compound Name	RT	Formula	Notes	Match Score	Mass	Difference	Ion Species
✓	Trp Ser Lys His	48.243	C26 H36 N8 O6		91.17	556.274	1.79	(M+Na)+[-H2O]
	Lys Trp His Ser	48.243	C26 H36 N8 O6		91.17	556.274	1.79	(M+Na)+[-H2O]
	His Lys Trp Ser	48.243	C26 H36 N8 O6		91.17	556.274	1.79	(M+Na)+[-H2O]
	His Ser Trp Lys	48.243	C26 H36 N8 O6		91.17	556.274	1.79	(M+Na)+[-H2O]
	His Trp Lys Ser	48.243	C26 H36 N8 O6		91.17	556.274	1.79	(M+Na)+[-H2O]
	His Trp Ser Lys	48.243	C26 H36 N8 O6		91.17	556.274	1.79	(M+Na)+[-H2O]
	His Lys Ser Trp	48.243	C26 H36 N8 O6		91.17	556.274	1.79	(M+Na)+[-H2O]
	Trp Ser His Lys	48.243	C26 H36 N8 O6		91.17	556.274	1.79	(M+Na)+[-H2O]
	Lys His Ser Trp	48.243	C26 H36 N8 O6		91.17	556.274	1.79	(M+Na)+[-H2O]
	Lys His Trp Ser	48.243	C26 H36 N8 O6		91.17	556.274	1.79	(M+Na)+[-H2O]

Compound Label	Name	*m/z*	RT	Algorithm	Mass
Cpd 64: 14-O-(alpha-L-rhamnopyranosyl)-7S,14R-dihydroxy-7,9,13,17-tetramethyl-2E,4E,8E,10E,12E,16E-octadecahexaenoic acid; C28 H42 O8; 49.363	**14-O-(alpha-L-rhamnopyranosyl)-7S,14R-dihydroxy-7,9,13,17-tetramethyl-2E,4E,8E,10E,12E,16E-octadecahexaenoic acid**	529.2778	49.363	Find by Molecular Feature	506.2884

Compound Chromatograms

Qualitative Compound Identification Report

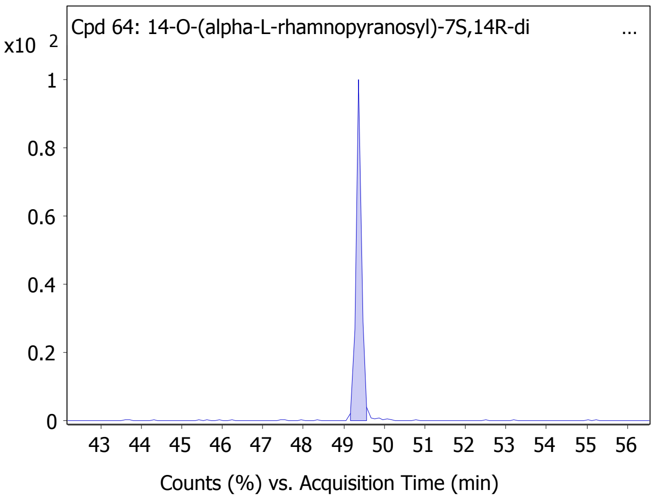

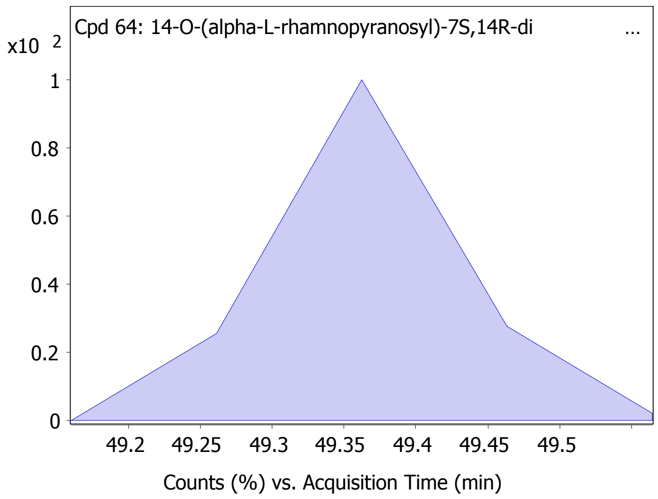

MFE MS Spectrum

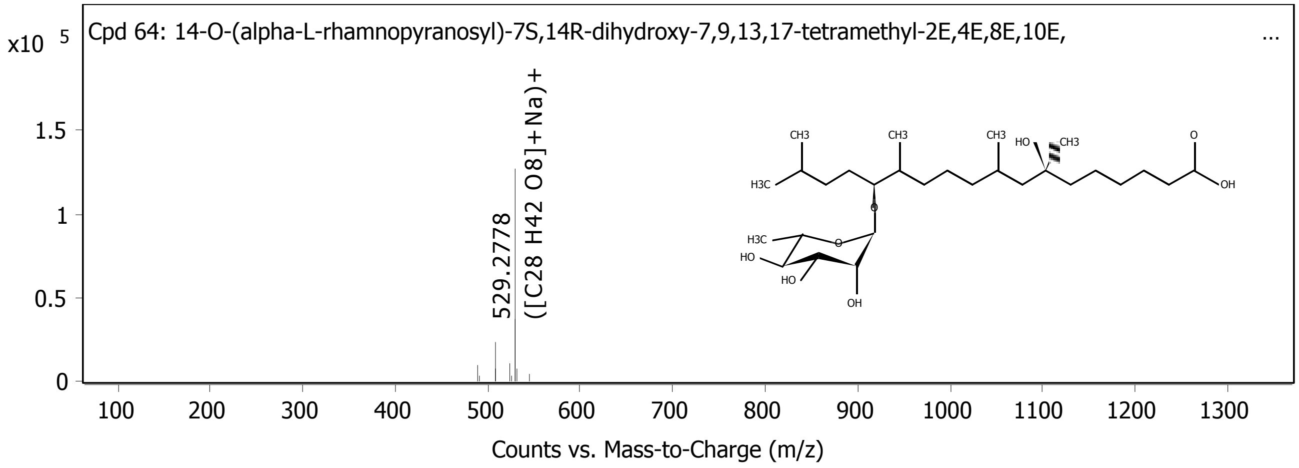

MFE MS Zoomed Spectrum

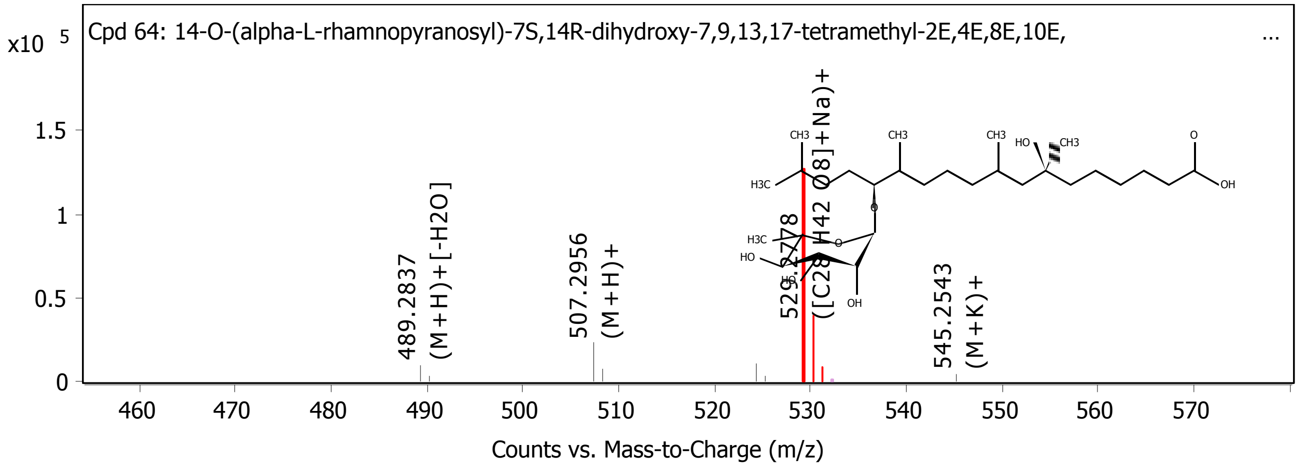

MS Spectrum

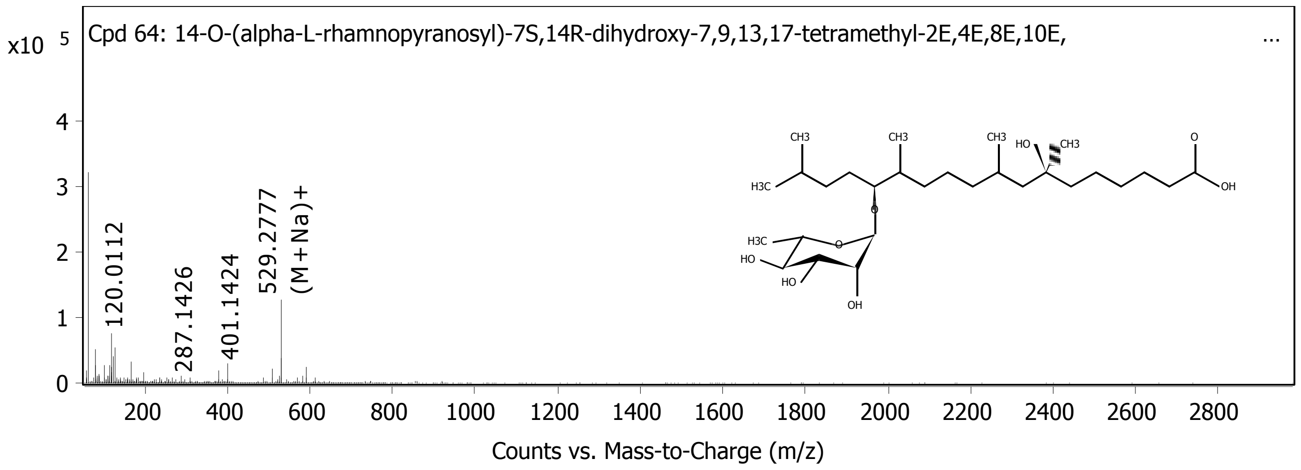

MS Zoomed Spectrum

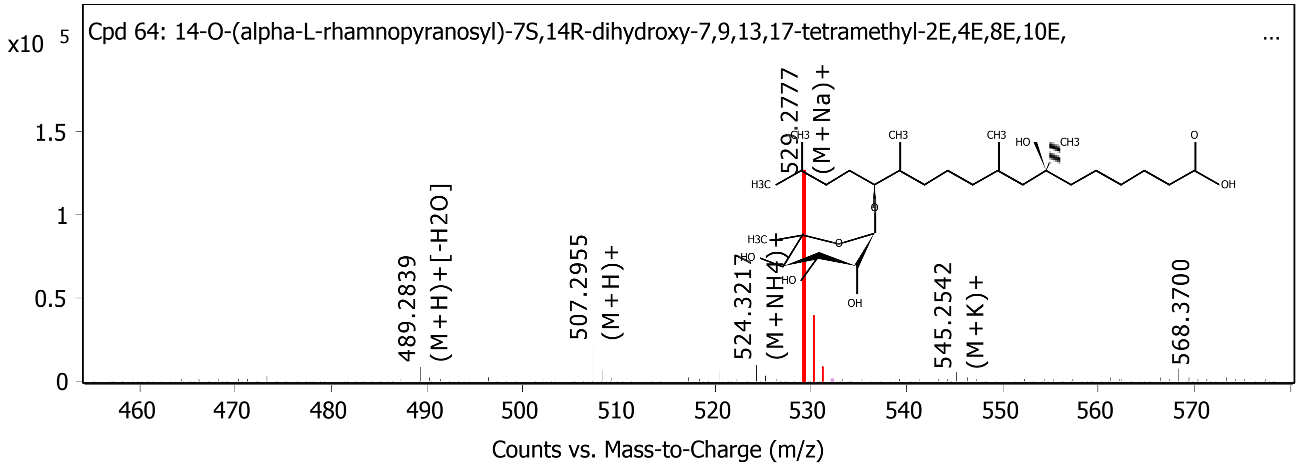

Identification Hit Table

Best Hit	Compound Name	RT	Formula	Notes	Match Score	Mass	Difference	Ion Species
✓	14-O-(alpha-L-rhamnopyranosyl)-7S,14R-dihydroxy-7,9,13,17-tetramethyl-2E,4E,8E,10E,12E,16E-octadecahexaenoic acid	49.363	C28 H42 O8		98.6	506.2884	-0.46	(M+Na)+
	(5alpha,6beta,14alpha,20R,22R)-5,6,14,20,27-Pentahydroxy-1-oxowith-24-enolide	49.363	C28 H42 O8		98.6	506.2884	-0.46	(M+Na)+
	Mucronine A	49.363	C29 H38 N4 O4		96.03	506.2885	0.8	(M+Na)+
	Ixabepilone	49.363	C27 H42 N2 O5 S		52.25	506.2887	-7.26	(M+Na)+

Identification Hit Table

Best Hit	Compound Name	RT	Formula	Notes	Match Score	Mass	Difference	Ion Species
✓	14-O-(alpha-L-rhamnopyranosyl)-7S,14R-dihydroxy-7,9,13,17-tetramethyl-2E,4E,8E,10E,12E,16E-octadecahexaenoic acid	49.363	C28 H42 O8		98.6	506.2884	-0.46	(M+Na)+

Identification Hit Table

Best Hit	Compound Name	RT	Formula	Notes	Match Score	Mass	Difference	Ion Species
✓	Phe Ile Val Phe	49.363	C29 H40 N4 O5		96.08	524.2991	0.8	(M+Na)+[-H2O]
	Phe Phe Val Ile	49.363	C29 H40 N4 O5		96.08	524.2991	0.8	(M+Na)+[-H2O]
	Phe Val Ile Phe	49.363	C29 H40 N4 O5		96.08	524.2991	0.8	(M+Na)+[-H2O]
	Phe Val Phe Leu	49.363	C29 H40 N4 O5		96.08	524.2991	0.8	(M+Na)+[-H2O]
	Phe Val Phe Ile	49.363	C29 H40 N4 O5		96.08	524.2991	0.8	(M+Na)+[-H2O]
	Phe Leu Val Phe	49.363	C29 H40 N4 O5		96.08	524.2991	0.8	(M+Na)+[-H2O]
	Phe Leu Phe Val	49.363	C29 H40 N4 O5		96.08	524.2991	0.8	(M+Na)+[-H2O]
	Phe Ile Phe Val	49.363	C29 H40 N4 O5		96.08	524.2991	0.8	(M+Na)+[-H2O]
	Ile Phe Phe Val	49.363	C29 H40 N4 O5		96.08	524.2991	0.8	(M+Na)+[-H2O]
	Ile Phe Val Phe	49.363	C29 H40 N4 O5		96.08	524.2991	0.8	(M+Na)+[-H2O]

Compound Label	*m/z*	RT	Algorithm	Mass
Cpd 65: C22 H36 N9 O3; 51.097	238.1542	51.097	Find by Molecular Feature	474.294

Compound Chromatograms

Qualitative Compound Identification Report

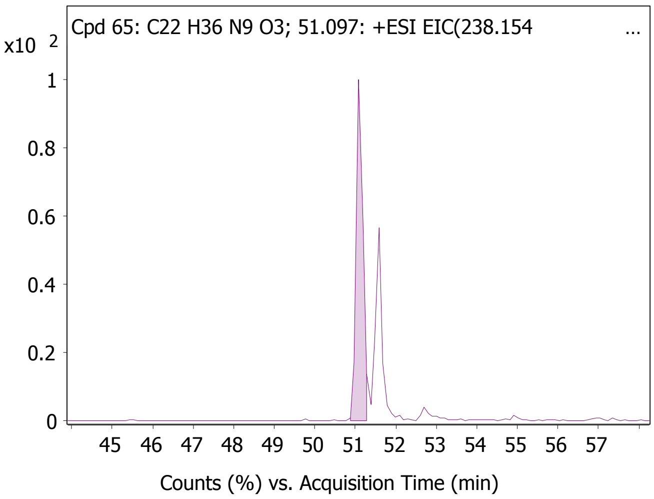

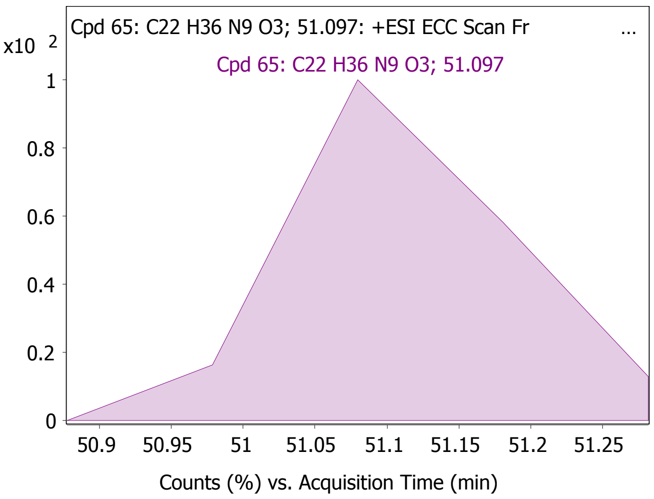

MFE MS Spectrum

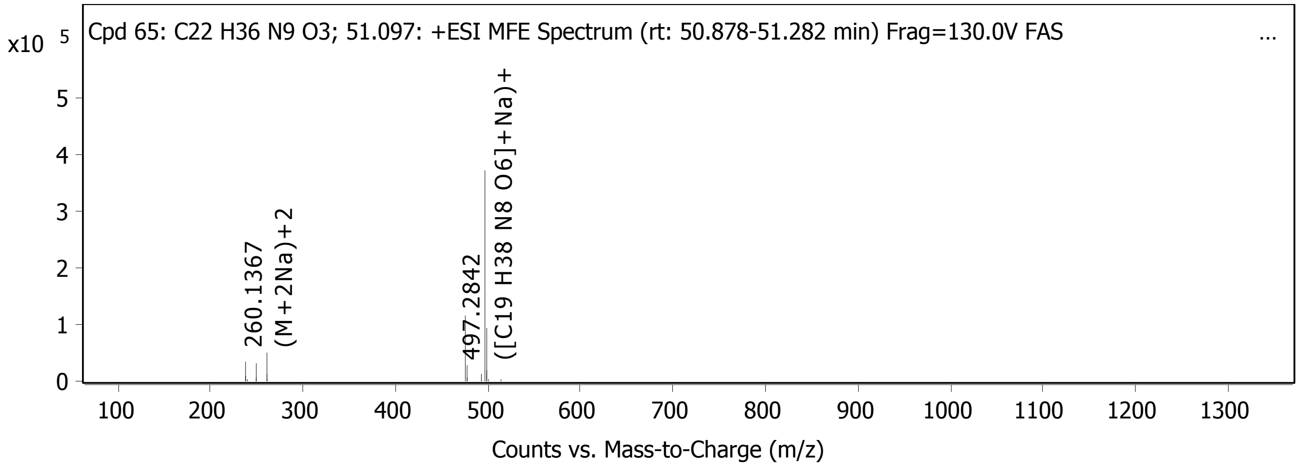

MFE MS Zoomed Spectrum

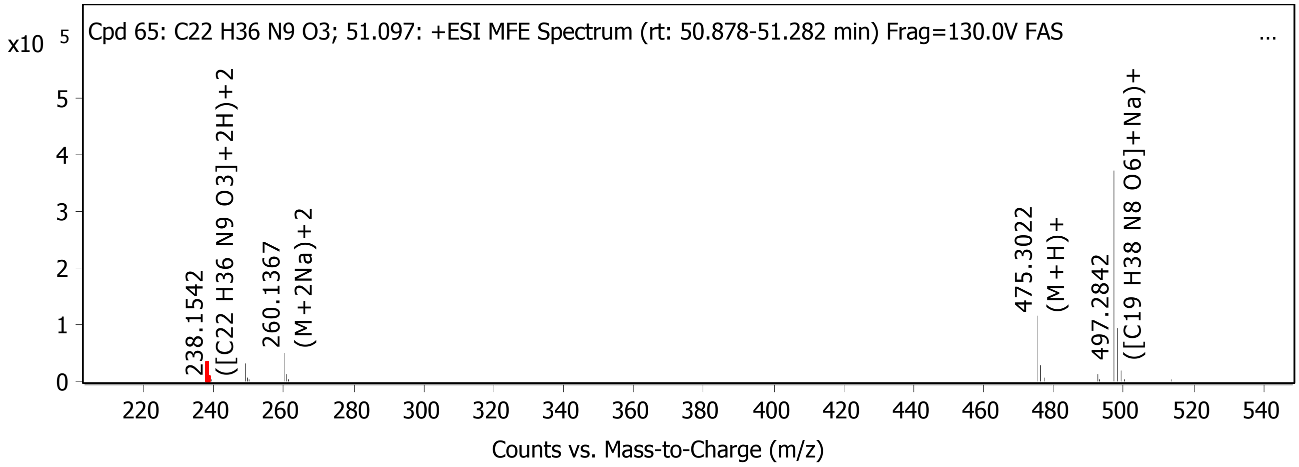

MS Spectrum

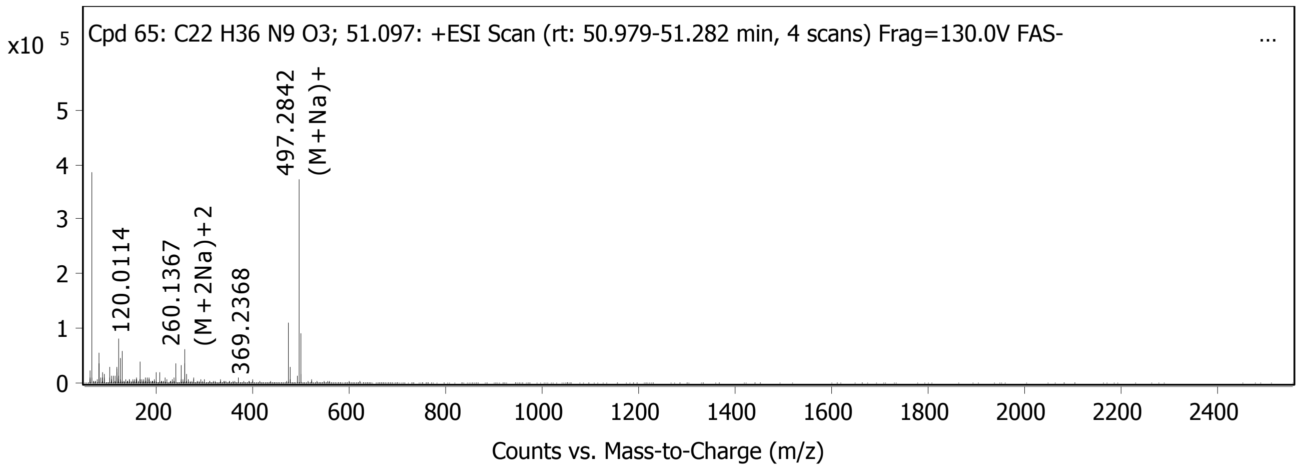

MS Zoomed Spectrum

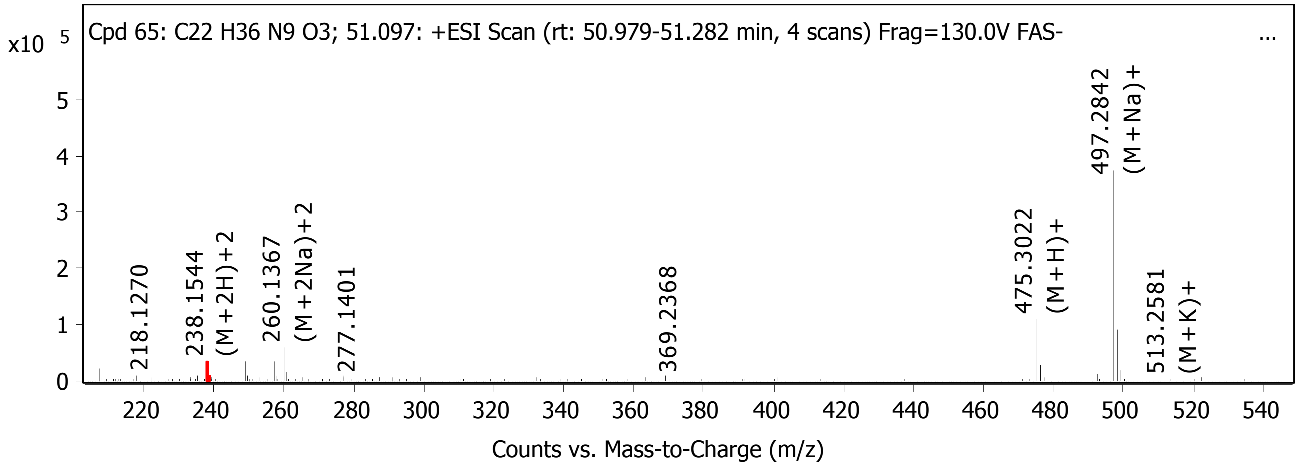

MSMS Spectrum

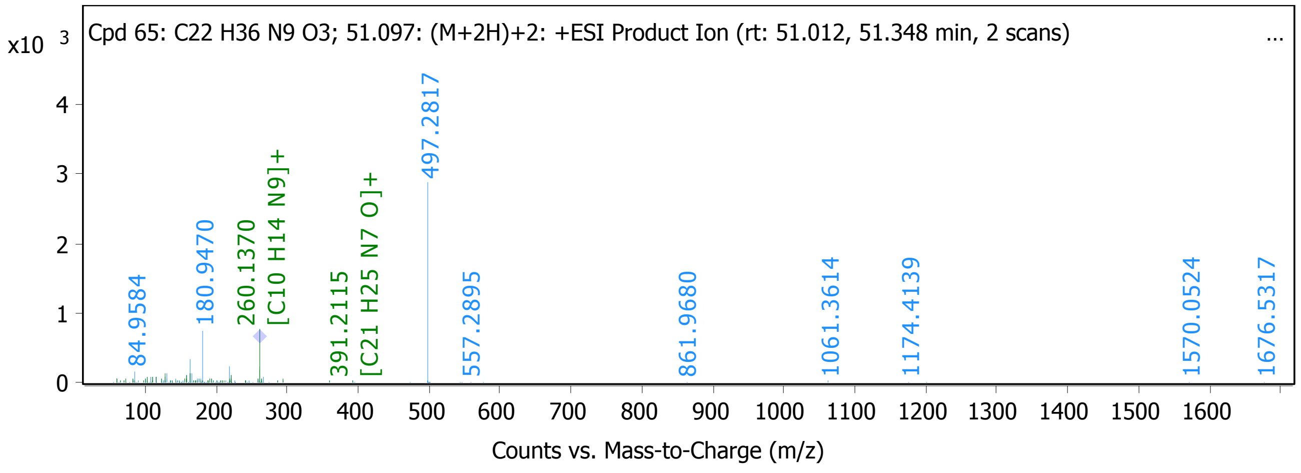

MSMS Spectrum

Qualitative Compound Identification Report

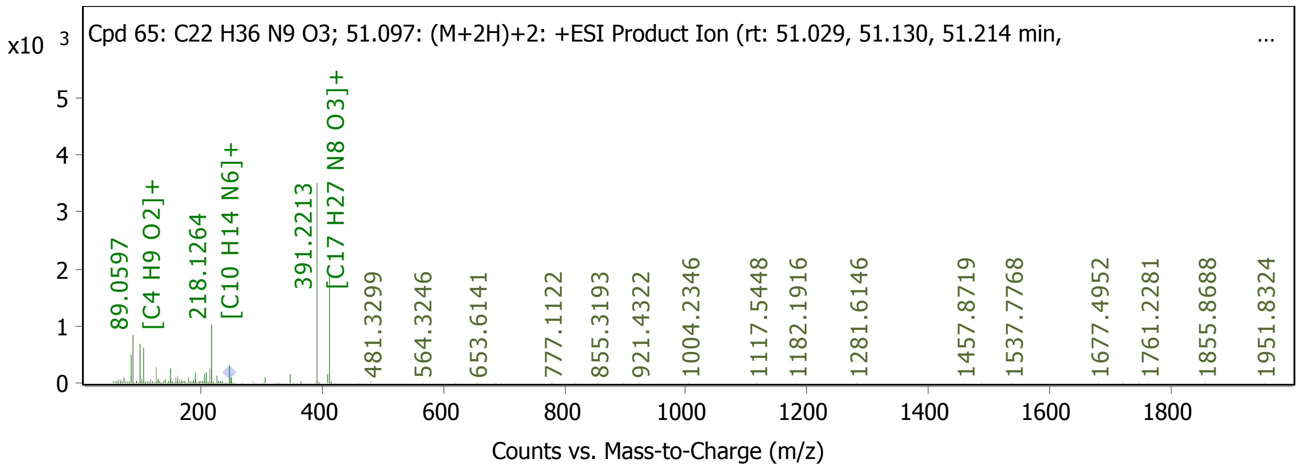

MSMS Spectrum

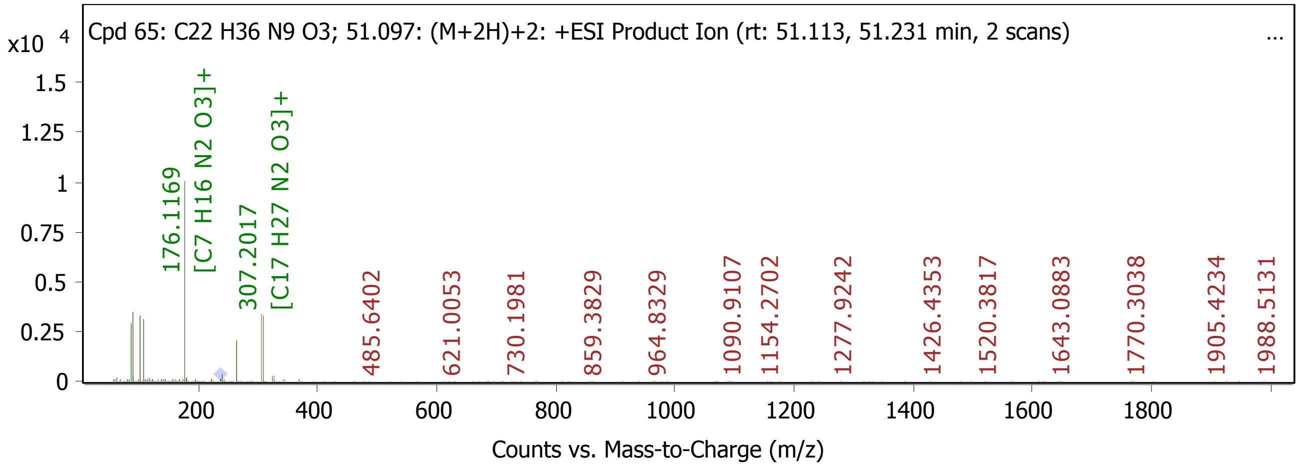

MSMS Spectrum

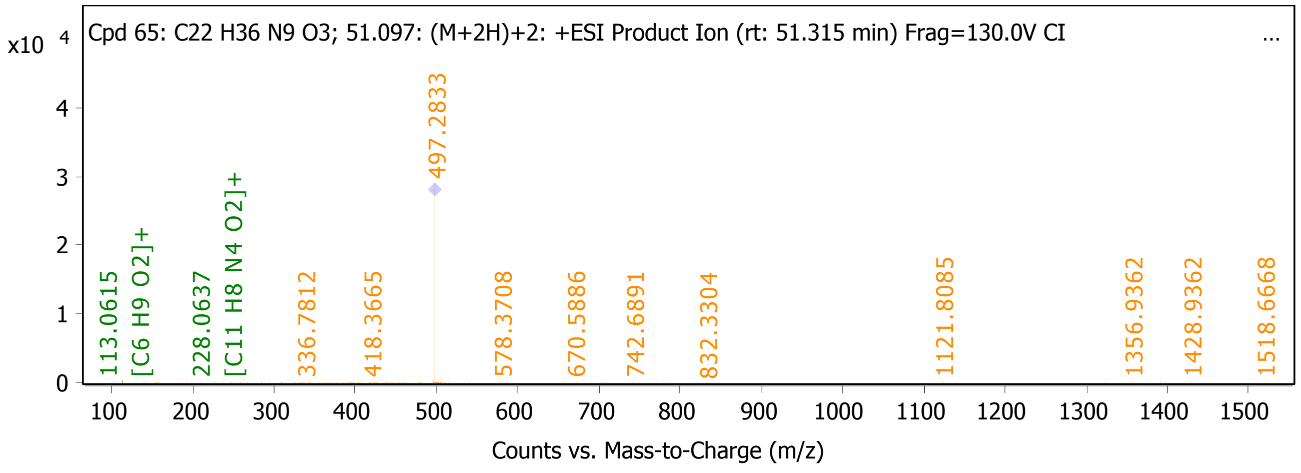

Compound Label	*m/z*	RT	Algorithm	Mass
Cpd 66: C22 H36 N9 O3; 51.581	238.1542	51.581	Find by Molecular Feature	474.2942

Compound Chromatograms

Qualitative Compound Identification Report

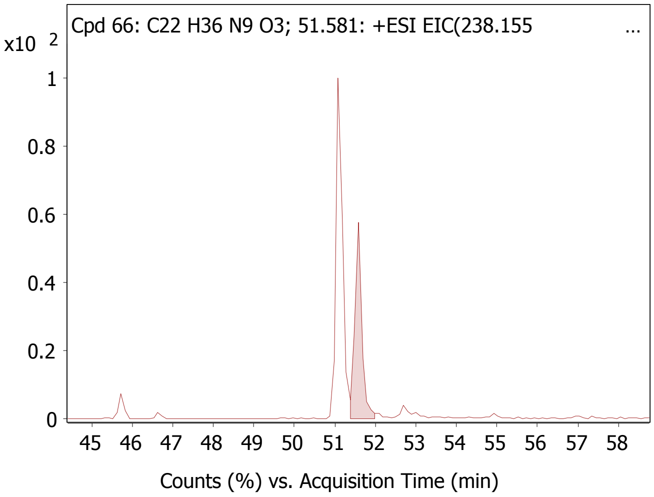

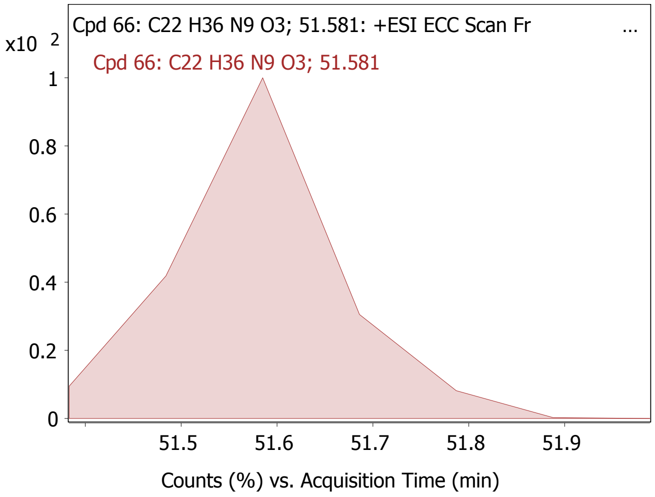

MFE MS Spectrum

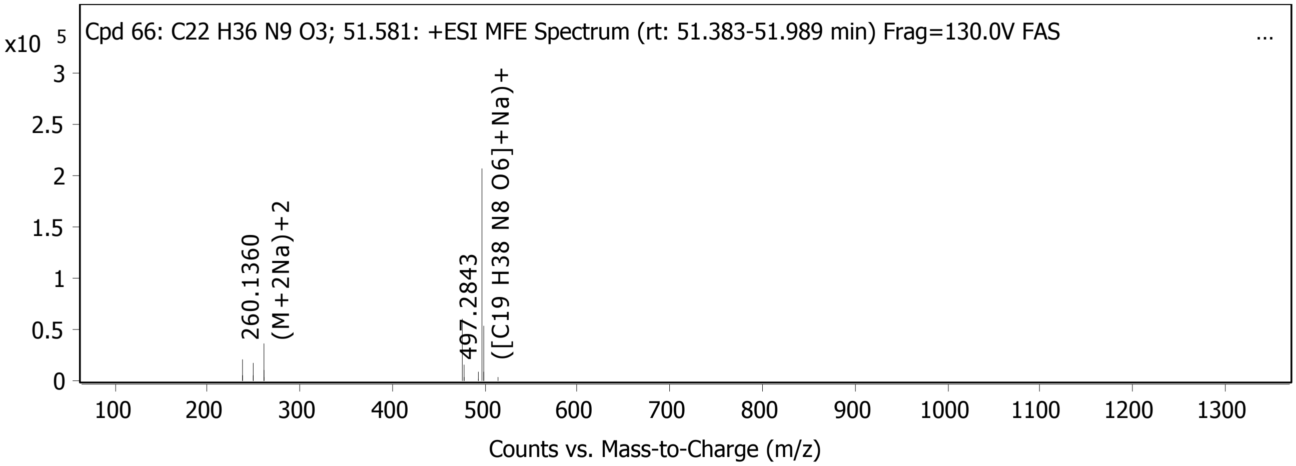

MFE MS Zoomed Spectrum

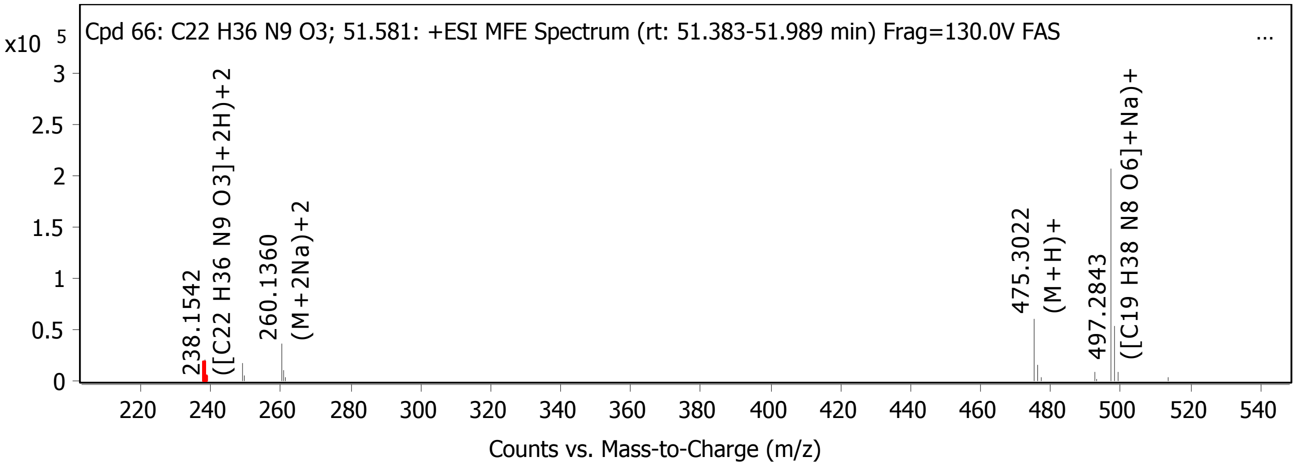

MS Spectrum

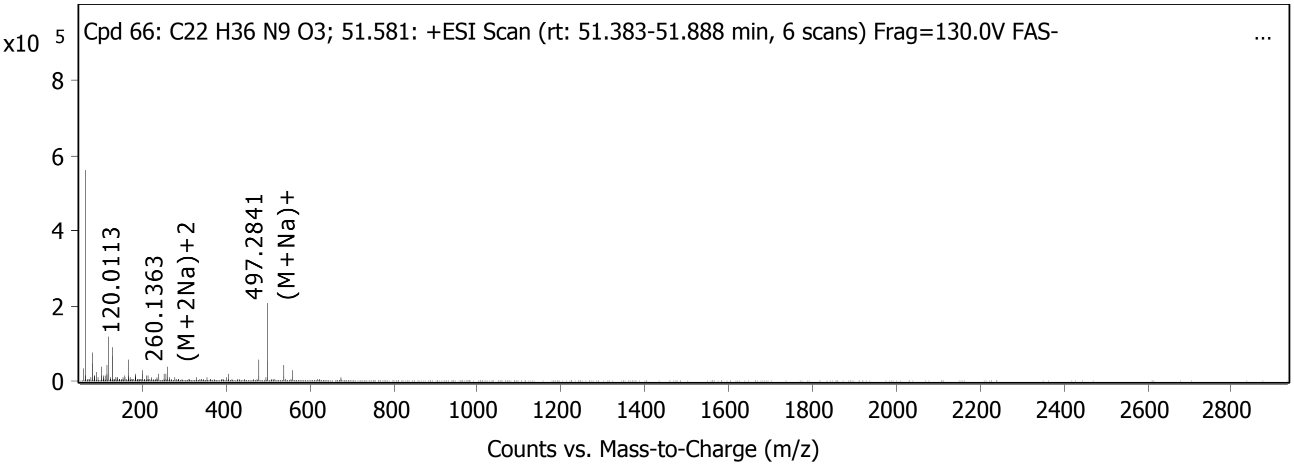

MS Zoomed Spectrum

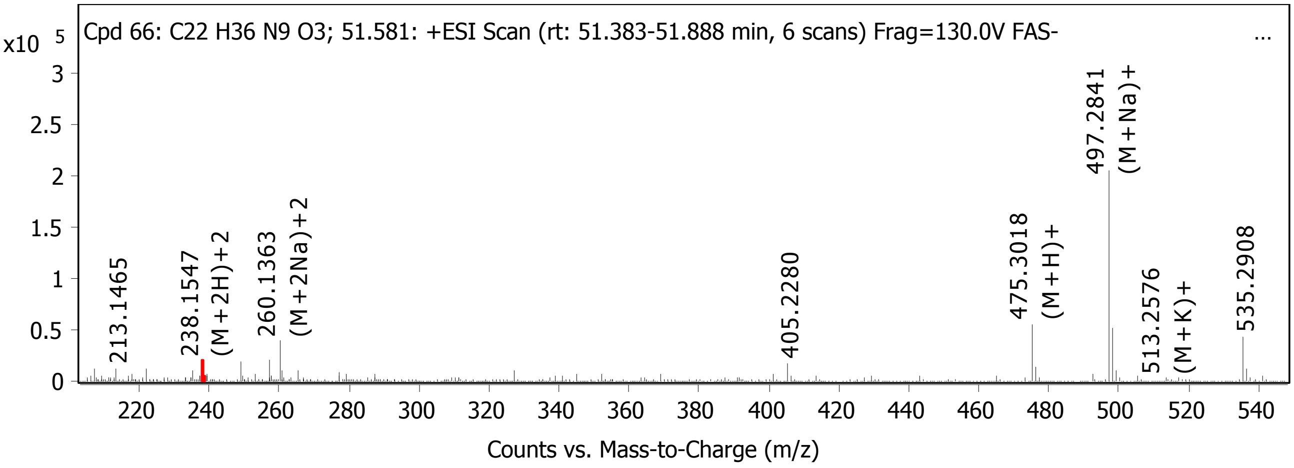

MSMS Spectrum

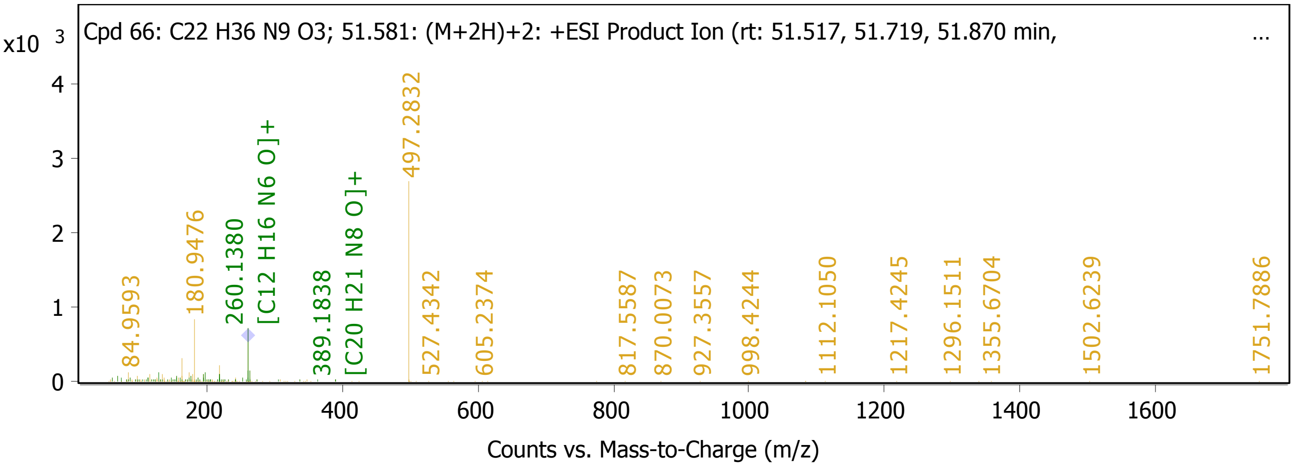

MSMS Spectrum

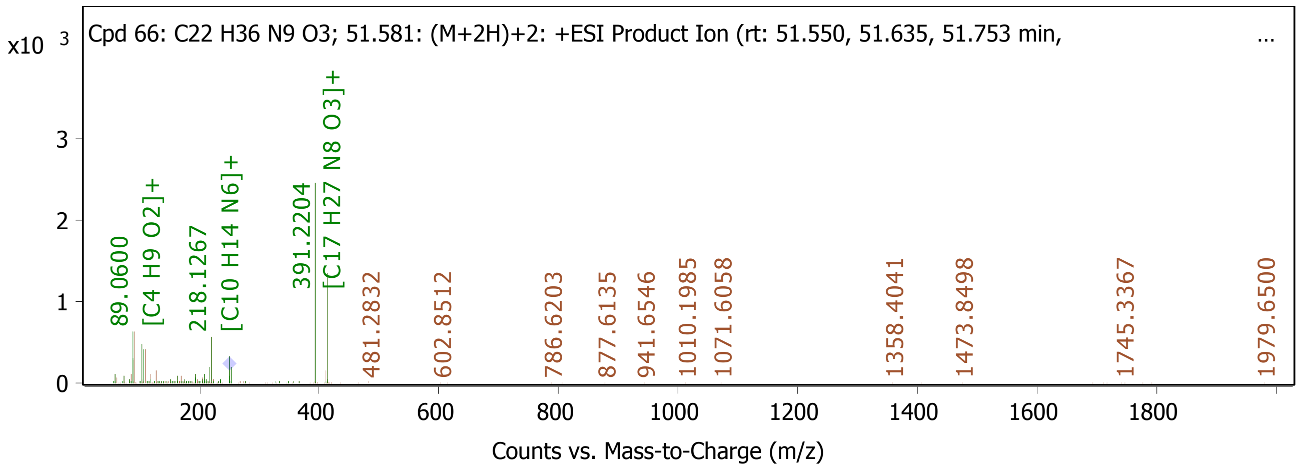

MSMS Spectrum

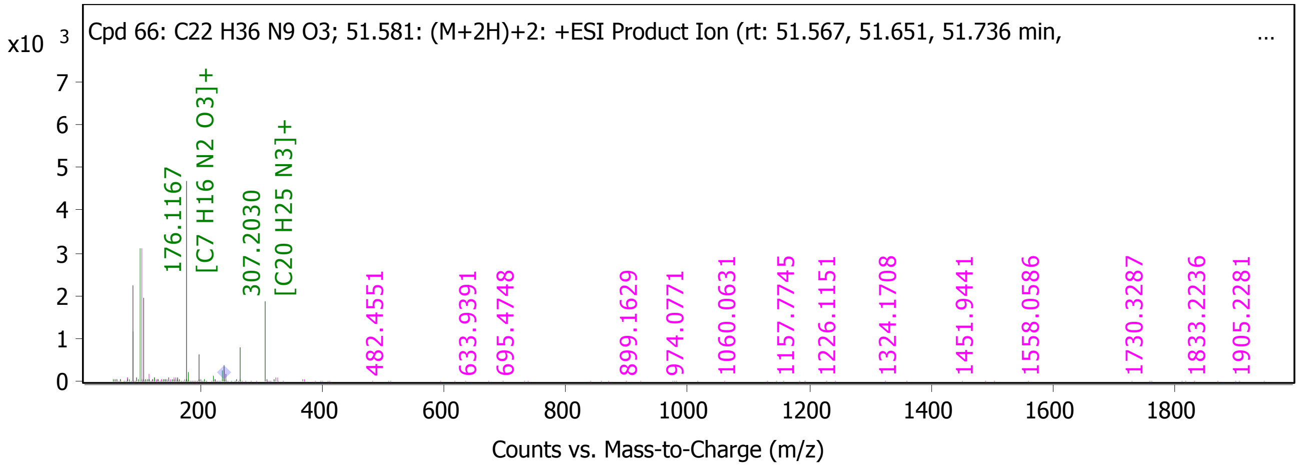

Compound Label	Name	*m/z*	RT	Algorithm	Mass
Cpd 67: QX-314; C16 H26 N2 O; 53.807	**QX-314**	244.1907	53.807	Find by Molecular Feature	262.2017

Compound Chromatograms

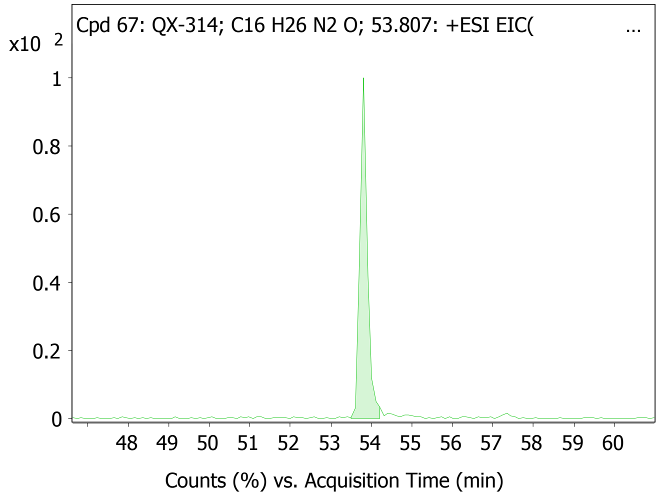

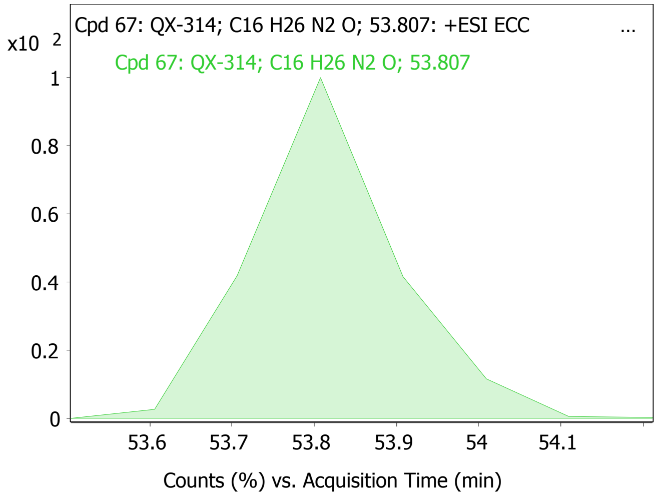

MFE MS Spectrum

Qualitative Compound Identification Report

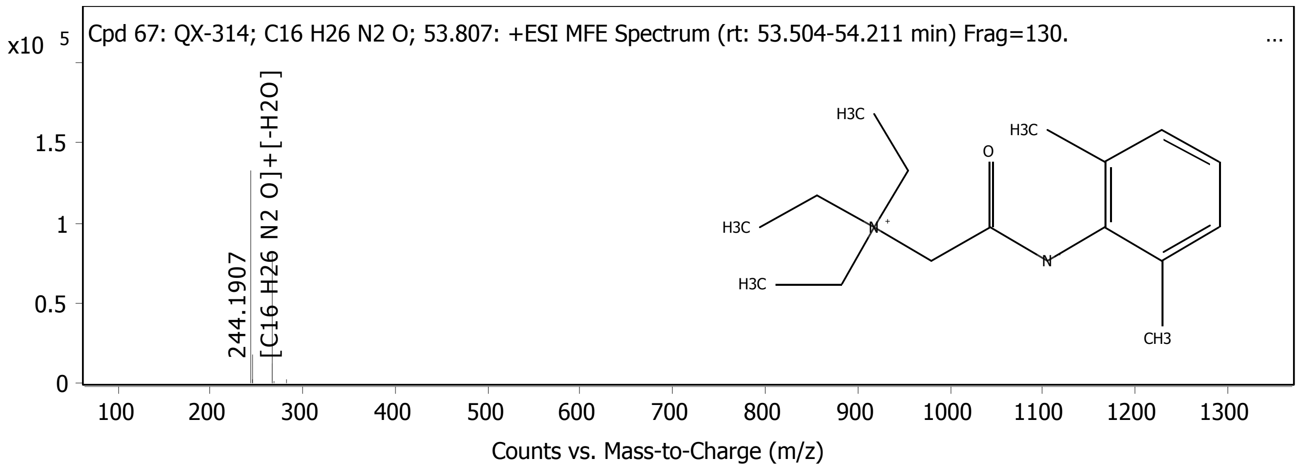

MFE MS Zoomed Spectrum

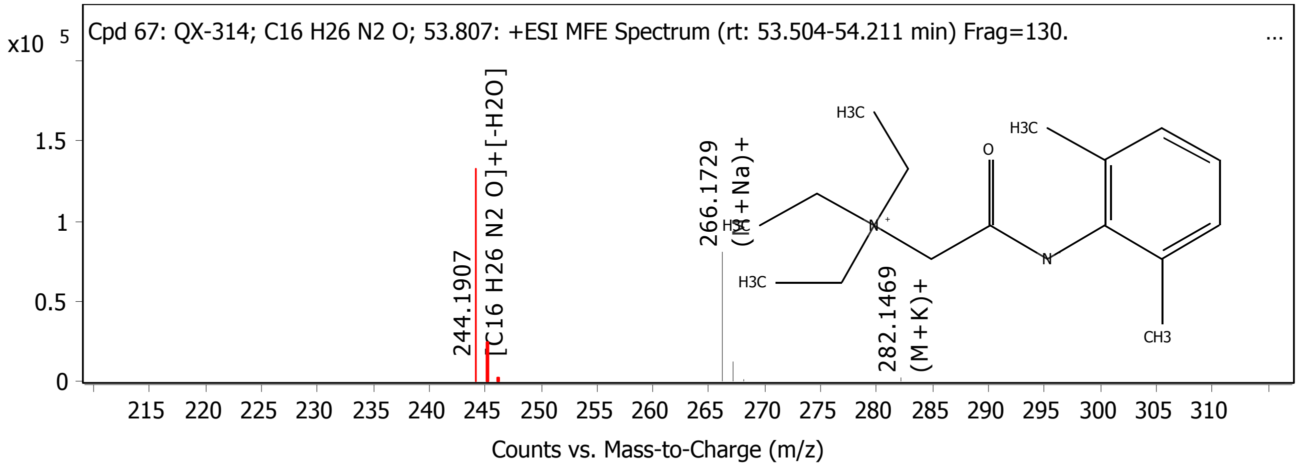

MS Spectrum

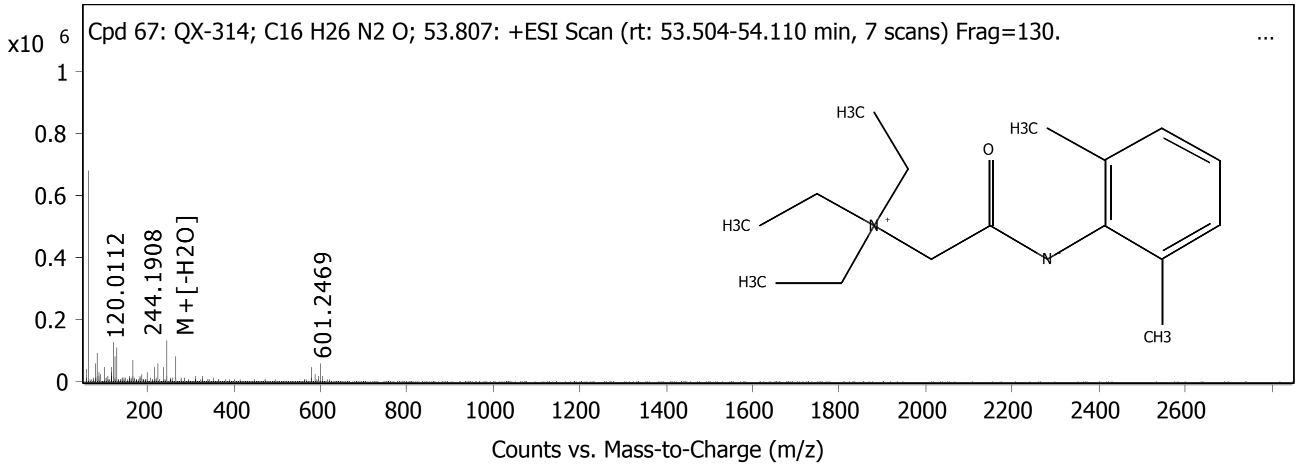

MS Zoomed Spectrum

Qualitative Compound Identification Report

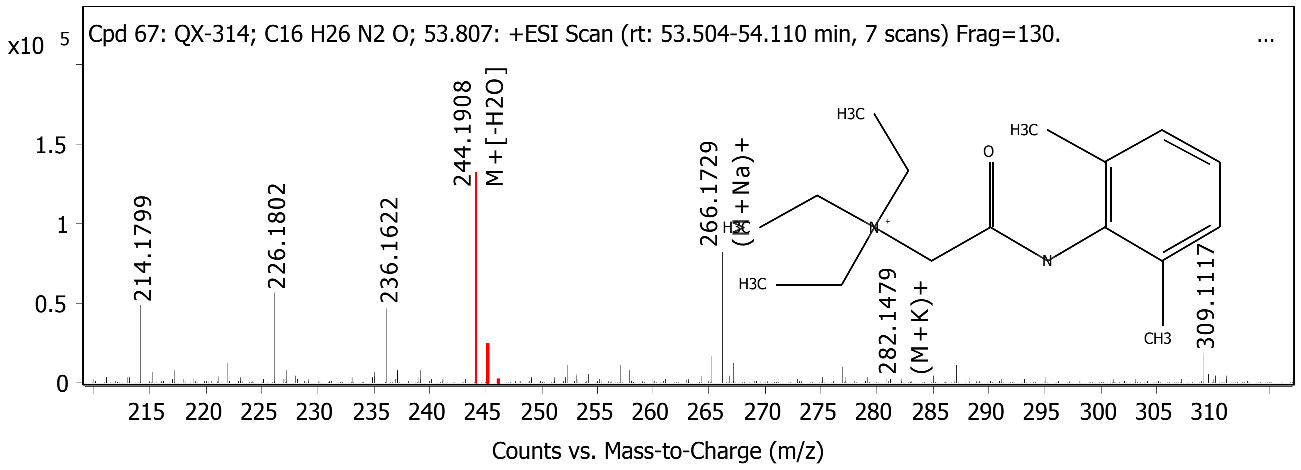

Identification Hit Table

Best Hit	Compound Name	RT	Formula	Notes	Match Score	Mass	Difference	Ion Species
✓	QX-314	53.807	C16 H26 N2 O		70.8	262.2017	2.77	M+[-H2O]

Identification Hit Table

Best Hit	Compound Name	RT	Formula	Notes	Match Score	Mass	Difference	Ion Species

Identification Hit Table

Best Hit	Compound Name	RT	Formula	Notes	Match Score	Mass	Difference	Ion Species

Compound Label	*m/z*	RT	Algorithm	Mass
Cpd 68: C13 H25 N O2; 58.225	228.1961	58.225	Find by Molecular Feature	227.1888

Compound Chromatograms

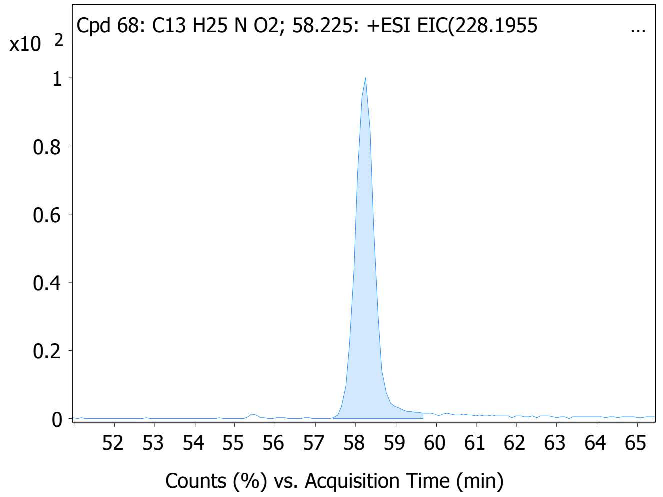

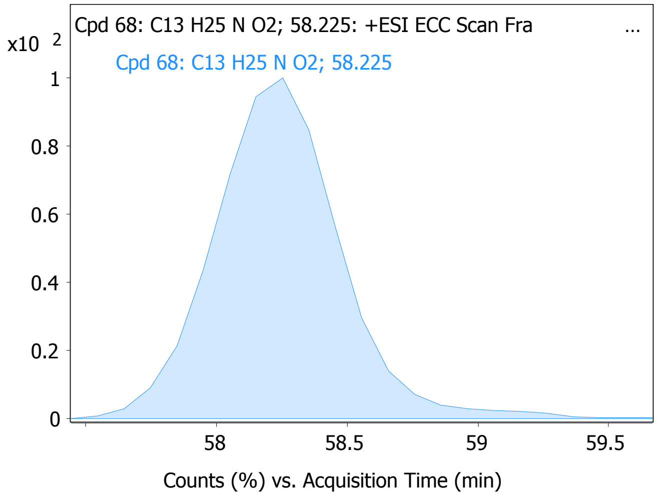

MFE MS Spectrum

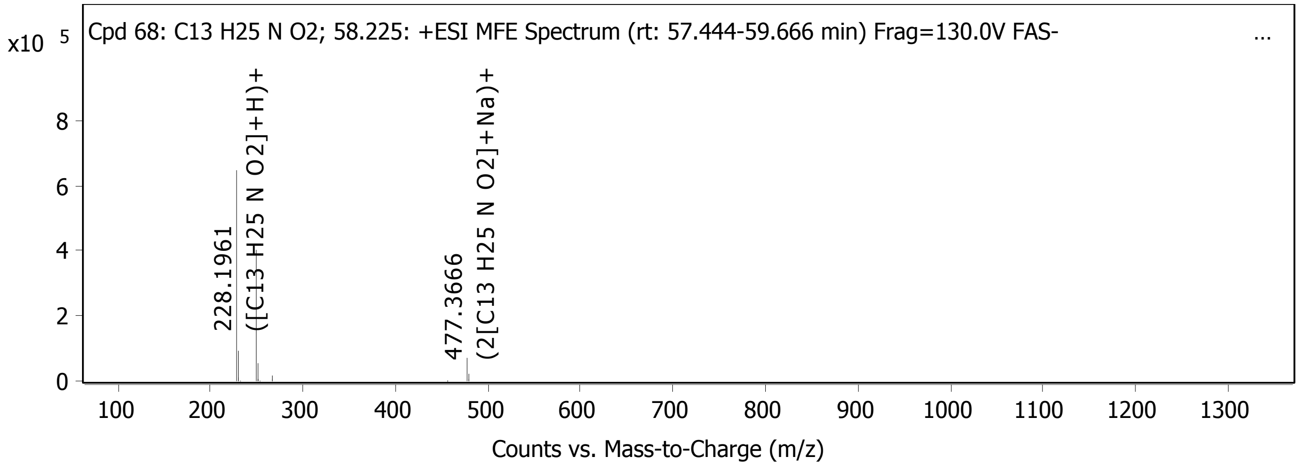

MFE MS Zoomed Spectrum

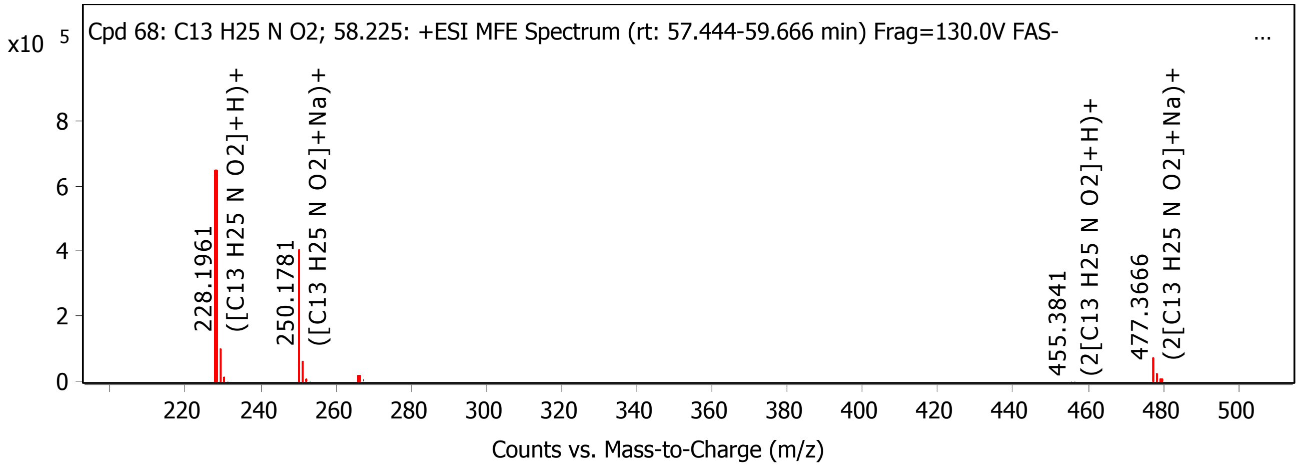

MS Spectrum

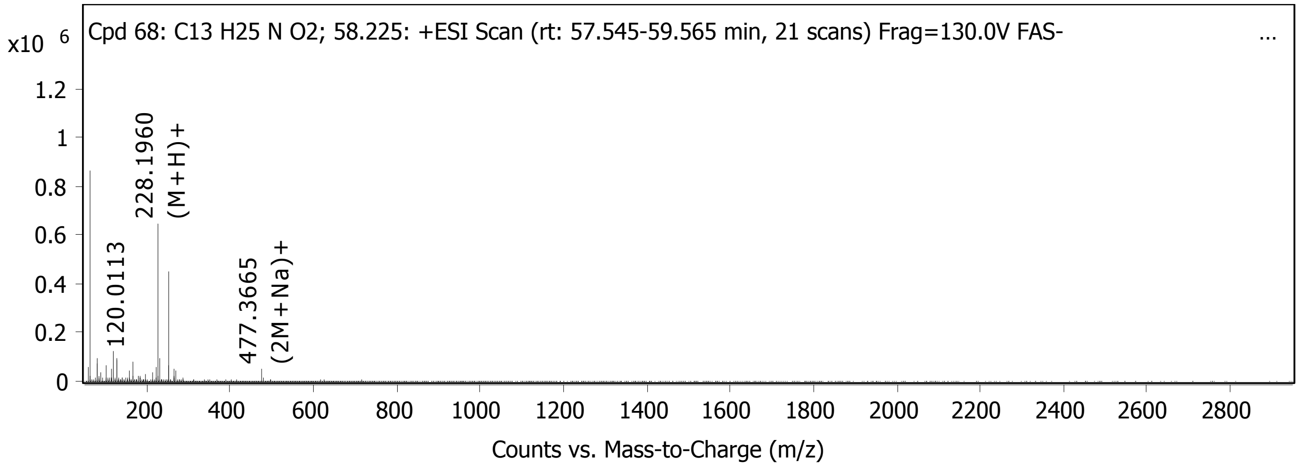

MS Zoomed Spectrum

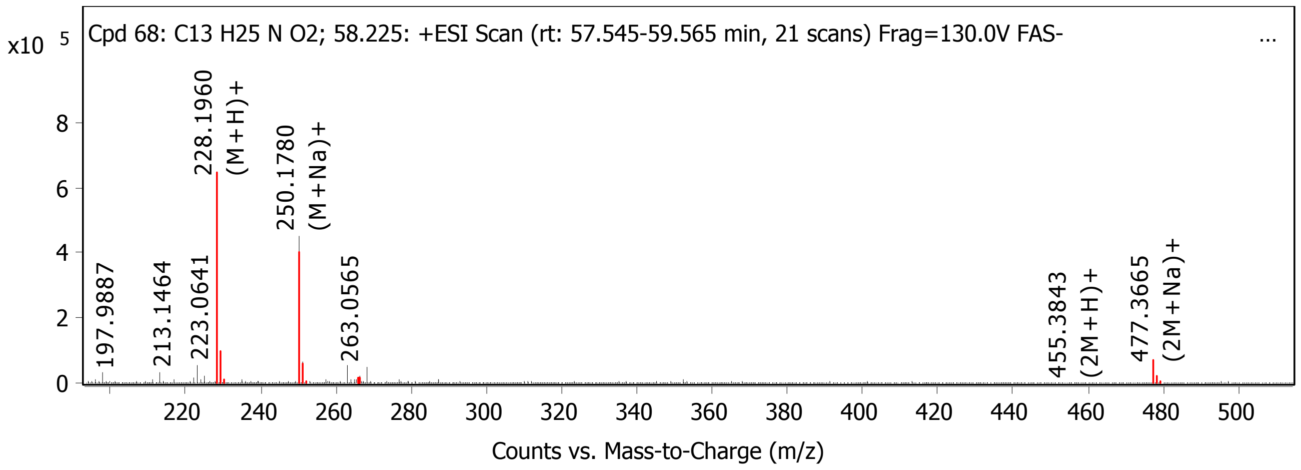

Compound Label	Name	m/z	RT	Algorithm	Mass
Cpd 69: Isoamyl p-anisate; C13 H18 O3; 62.574	**Isoamyl p-anisate**	227.1045	62.574	Find by Molecular Feature	222.1258

Compound Chromatograms

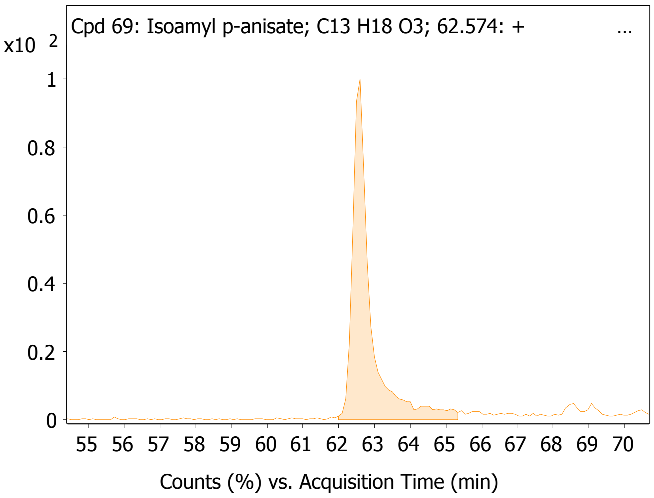

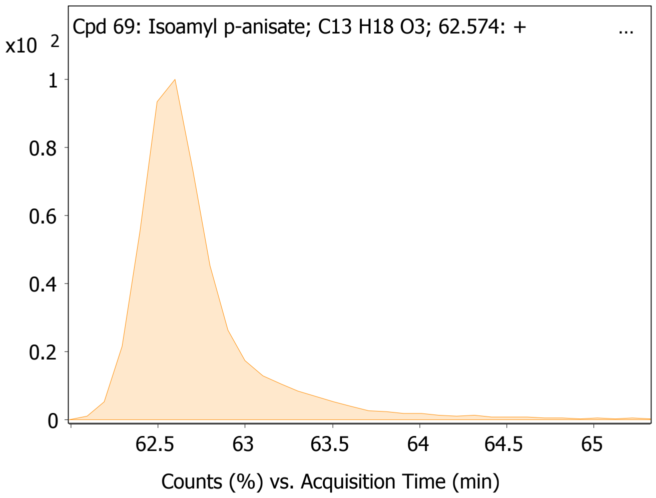

MFE MS Spectrum

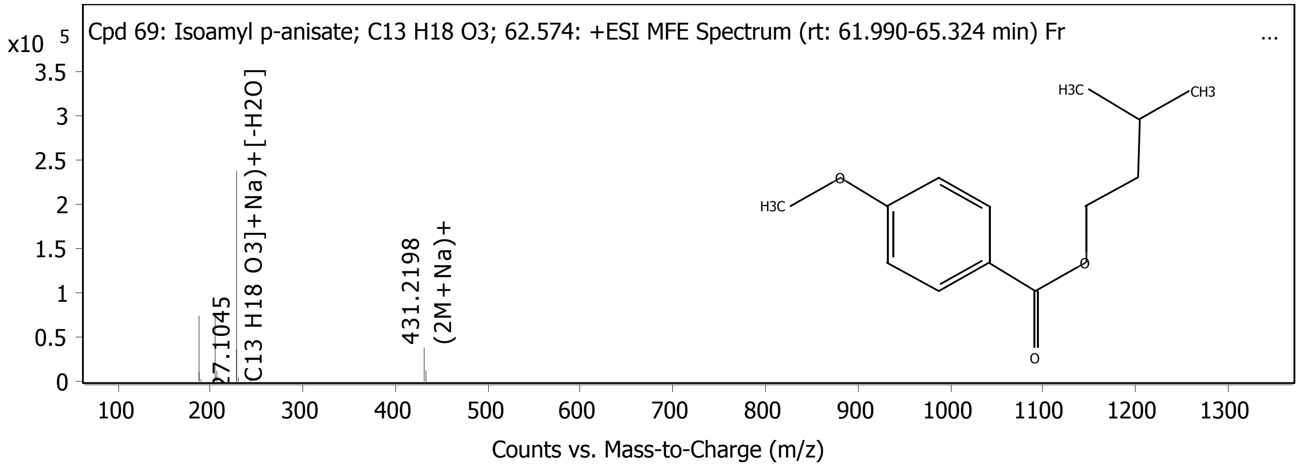

MFE MS Zoomed Spectrum

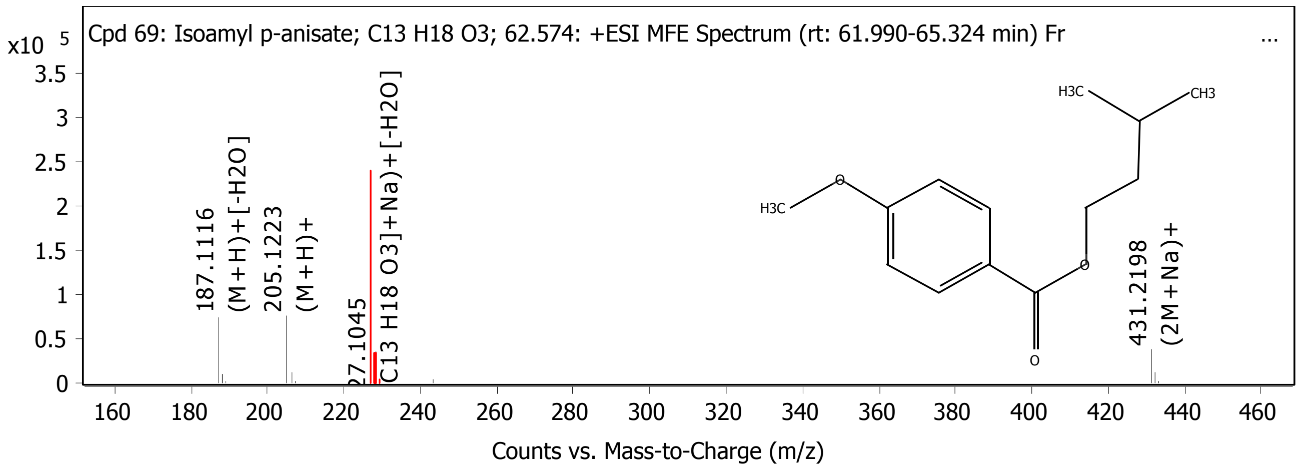

MS Spectrum

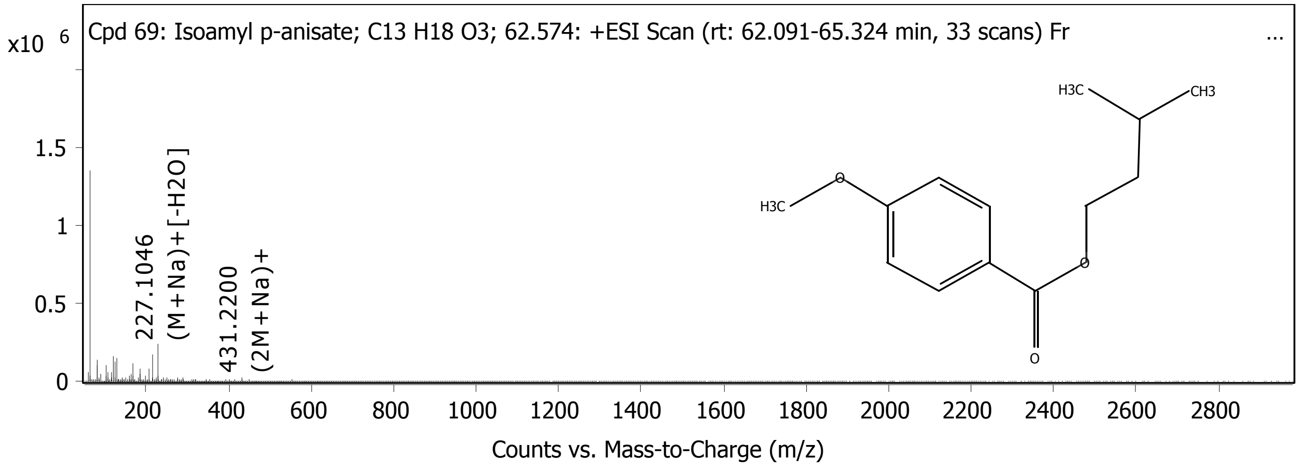

MS Zoomed Spectrum

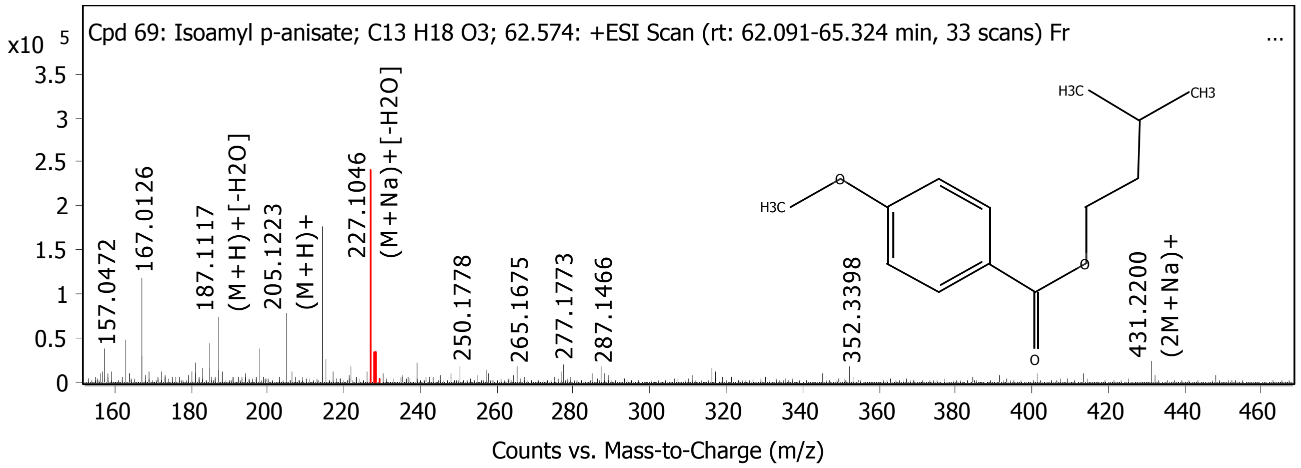

MSMS Spectrum

Qualitative Compound Identification Report

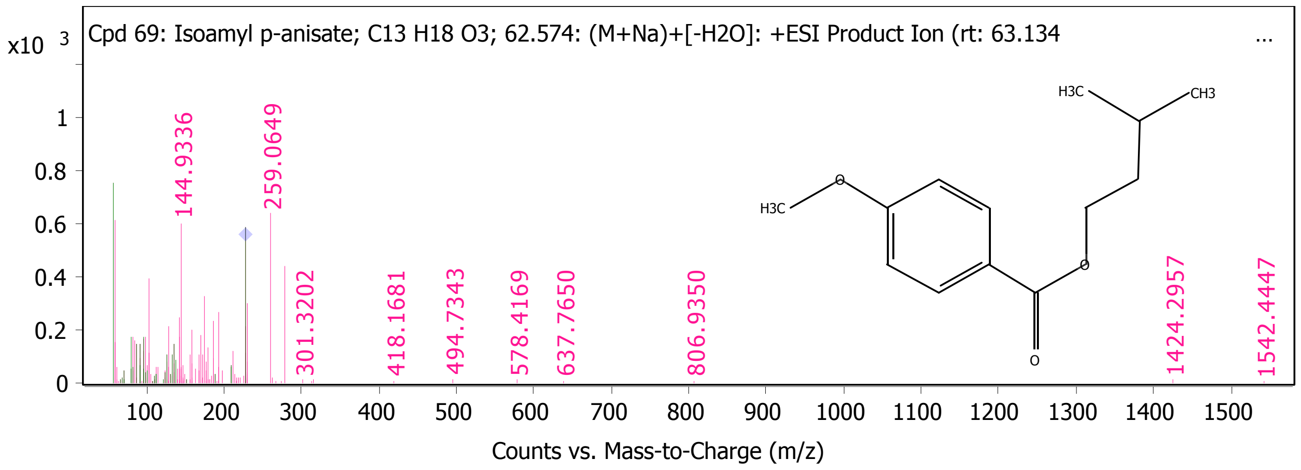

Identification Hit Table

Best Hit	Compound Name	RT	Formula	Notes	Match Score	Mass	Difference	Ion Species
✓	Isoamyl p-anisate	62.574	C13 H18 O3		99.61	222.1258	-0.17	(M+Na)+[-H2O]
	Annuionone B	62.574	C13 H18 O3		99.61	222.1258	-0.17	(M+Na)+[-H2O]
	(6S)-dehydrovomifoliol	62.574	C13 H18 O3		99.61	222.1258	-0.17	(M+Na)+[-H2O]
	Hydroxyibuprofen	62.574	C13 H18 O3	Anti-inflammatory Metabolite of Ibuprofen Dollery, Colin Therapeutic Drugs, 2nd Ed. 1999 p. I1.	99.61	222.1258	-0.17	(M+Na)+[-H2O]
	Dehydrovomifoliol	62.574	C13 H18 O3		99.61	222.1258	-0.17	(M+Na)+[-H2O]
	Benzyl 2,3-dimethyl-2-butenoate	62.574	C13 H16 O2		99.6	204.1152	-0.17	(M+Na)+
	Plastoquinone-1	62.574	C13 H16 O2		99.6	204.1152	-0.17	(M+Na)+
	alpha,alpha-Dimethylanisalacetone	62.574	C13 H16 O2		99.6	204.1152	-0.17	(M+Na)+
	6-(1-Hydroxyethyl)-2,2-dimethyl-2H-1-benzopyran	62.574	C13 H16 O2		99.6	204.1152	-0.17	(M+Na)+
	3-Isovalidene-3alpha,4-dihydrophthalide	62.574	C13 H16 O2		99.6	204.1152	-0.17	(M+Na)+

Identification Hit Table

Best Hit	Compound Name	RT	Formula	Notes	Match Score	Mass	Difference	Ion Species
✓	(6S)-dehydrovomifoliol	62.574	C13 H18 O3		99.61	222.1258	-0.17	(M+Na)+[-H2O]

Identification Hit Table

Best Hit	Compound Name	RT	Formula	Notes	Match Score	Mass	Difference	Ion Species

Compound Label	Name	*m/z*	RT	Algorithm	Mass
Cpd 70: 4-methyltridecan-7-ol; C14 H30 O; 63.107	**4-methyltridecan-7-ol**	214.2532	63.107	Find by Molecular Feature	214.2299

Compound Chromatograms

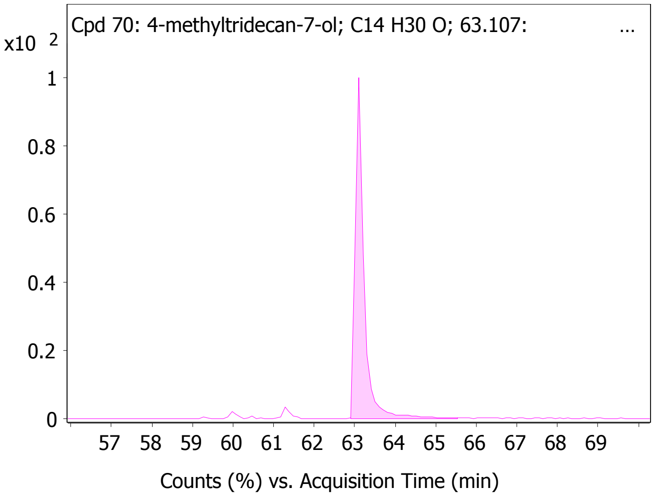

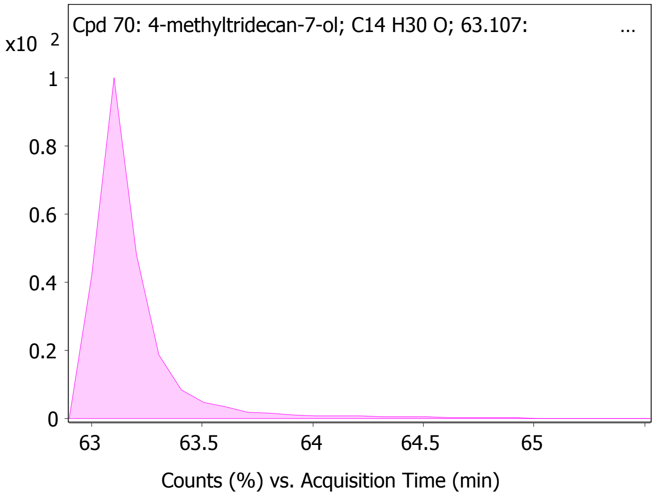

MFE MS Spectrum

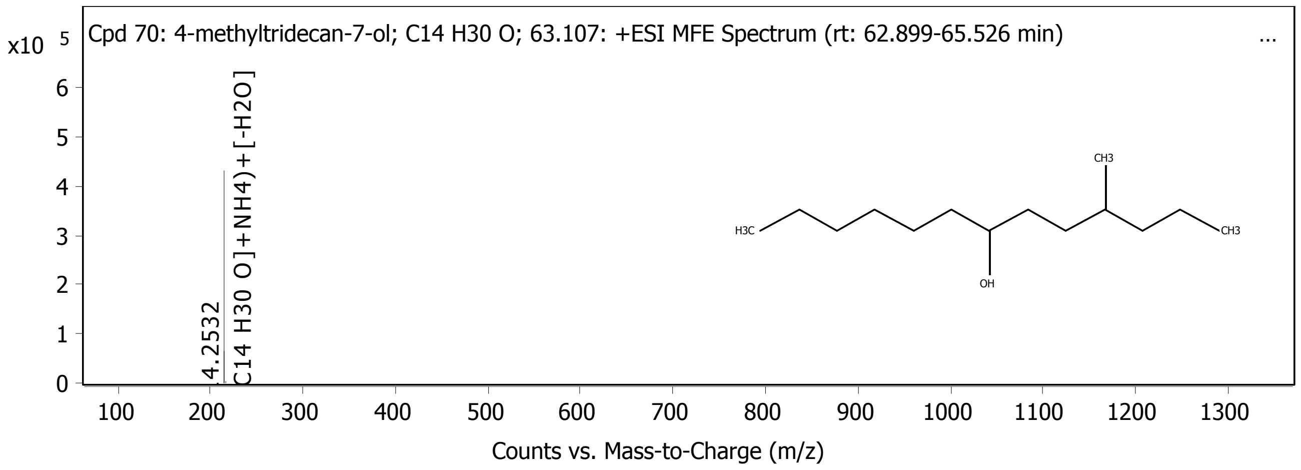

MFE MS Zoomed Spectrum

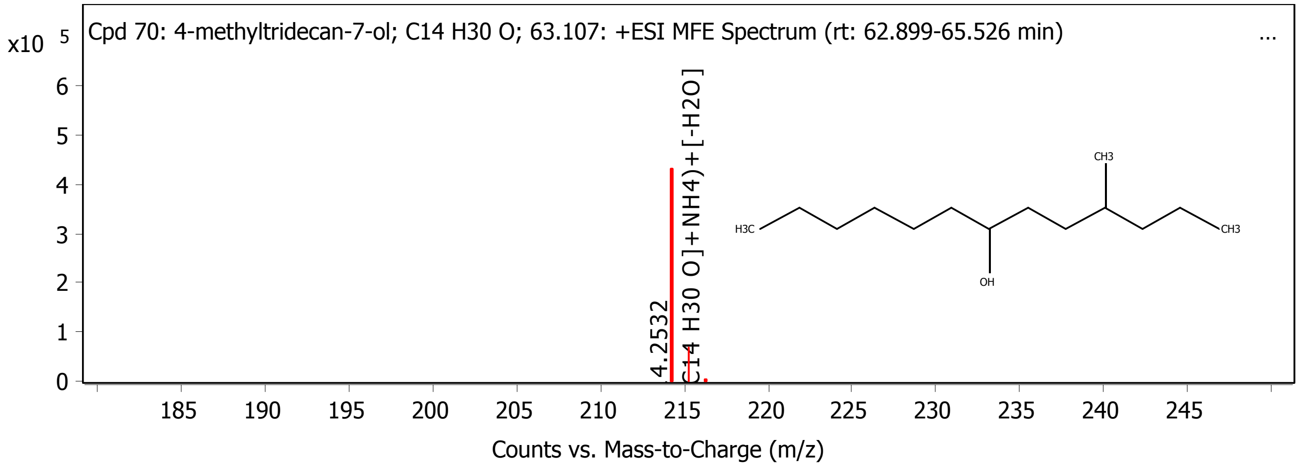

MS Spectrum

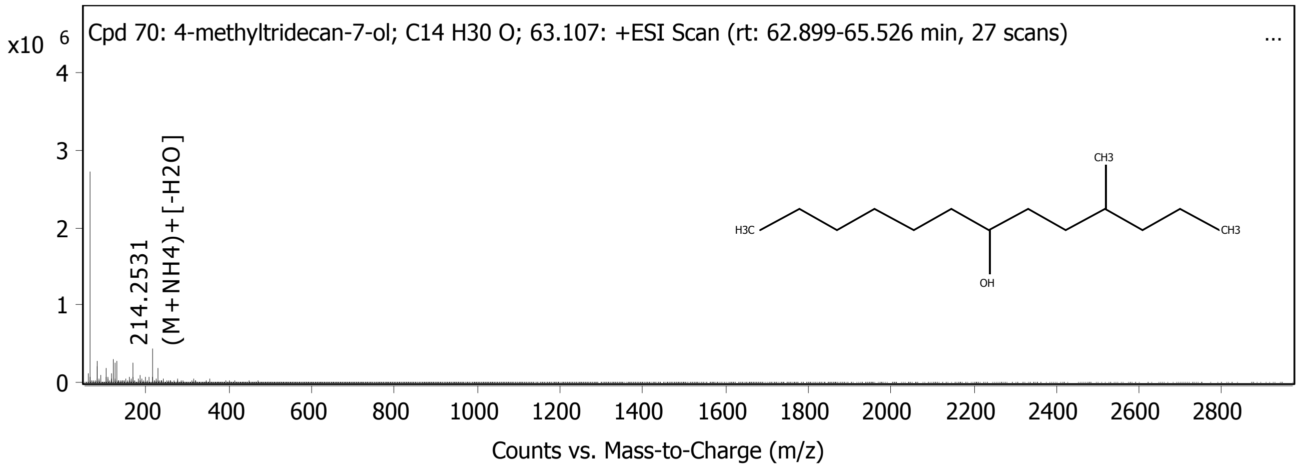

MS Zoomed Spectrum

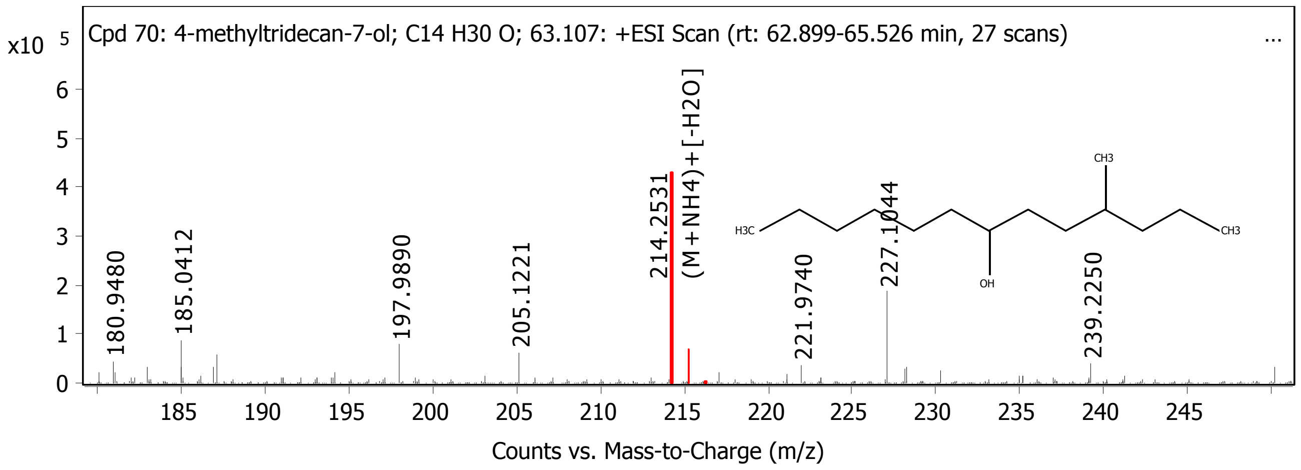

Identification Hit Table

Best Hit	Compound Name	RT	Formula	Notes	Match Score	Mass	Difference	Ion Species
✓	4-methyltridecan-7-ol	63.107	C14 H30 O		99.57	214.2299	-0.26	(M+NH4)+[-H2O]
	3,9-Dimethyldodecan-6-ol	63.107	C14 H30 O		99.57	214.2299	-0.26	(M+NH4)+[-H2O]
	1-Tetradecanol	63.107	C14 H30 O		99.57	214.2299	-0.26	(M+NH4)+[-H2O]
	Tetradecylamine	63.107	C14 H31 N	Postive MS/MS	99.56	213.2459	-0.26	(M+H)+
	3-Tetradecene	63.107	C14 H28		99.54	196.2194	-0.26	(M+NH4)+
	2,6-Dimethyl-2-dodecene	63.107	C14 H28		99.54	196.2194	-0.26	(M+NH4)+
	1-Tetradecene	63.107	C14 H28		99.54	196.2194	-0.26	(M+NH4)+
	7Z-Tetradecene	63.107	C14 H28		99.54	196.2194	-0.26	(M+NH4)+
	6Z-Tetradecene	63.107	C14 H28		99.54	196.2194	-0.26	(M+NH4)+
	Cyclotetradecane	63.107	C14 H28		99.54	196.2194	-0.26	(M+NH4)+

Identification Hit Table

Best Hit	Compound Name	RT	Formula	Notes	Match Score	Mass	Difference	Ion Species
✓	1-Tetradecanol	63.107	C14 H30 O		99.57	214.2299	-0.26	(M+NH4)+[-H2O]
	3,9-Dimethyldodecan-6-ol	63.107	C14 H30 O		99.57	214.2299	-0.26	(M+NH4)+[-H2O]
	4-methyltridecan-7-ol	63.107	C14 H30 O		99.57	214.2299	-0.26	(M+NH4)+[-H2O]
	6Z-Tetradecene	63.107	C14 H28		99.54	196.2194	-0.26	(M+NH4)+
	7Z-Tetradecene	63.107	C14 H28		99.54	196.2194	-0.26	(M+NH4)+
	1-Tetradecene	63.107	C14 H28		99.54	196.2194	-0.26	(M+NH4)+
	2,6-Dimethyl-2-dodecene	63.107	C14 H28		99.54	196.2194	-0.26	(M+NH4)+
	3-Tetradecene	63.107	C14 H28		99.54	196.2194	-0.26	(M+NH4)+

Identification Hit Table

Best Hit	Compound Name	RT	Formula	Notes	Match Score	Mass	Difference	Ion Species

Compound Label	Name	*m/z*	RT	Algorithm	Mass
Cpd 71: Phytosphingosine; C18 H39 N O3; 67.109	**Phytosphingosine**	318.3009	67.109	Find by Molecular Feature	317.2936

Compound Chromatograms

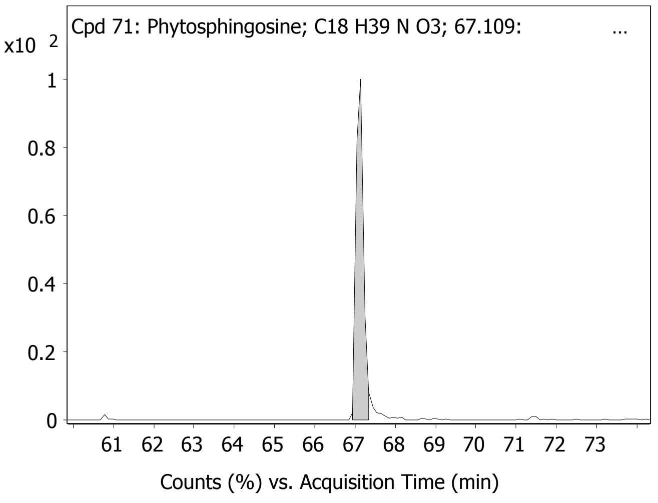

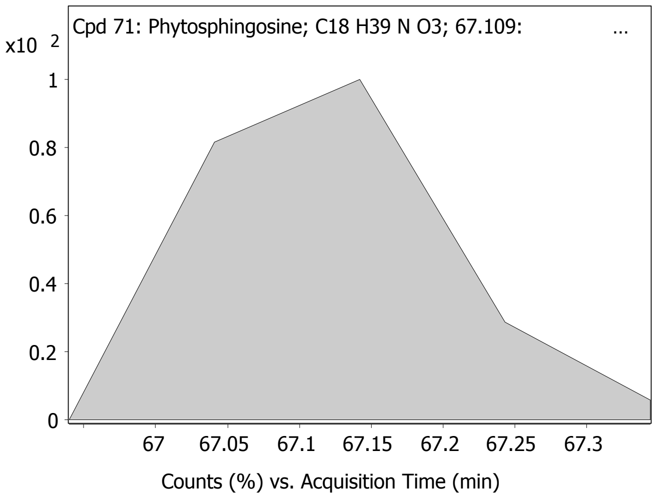

MFE MS Spectrum

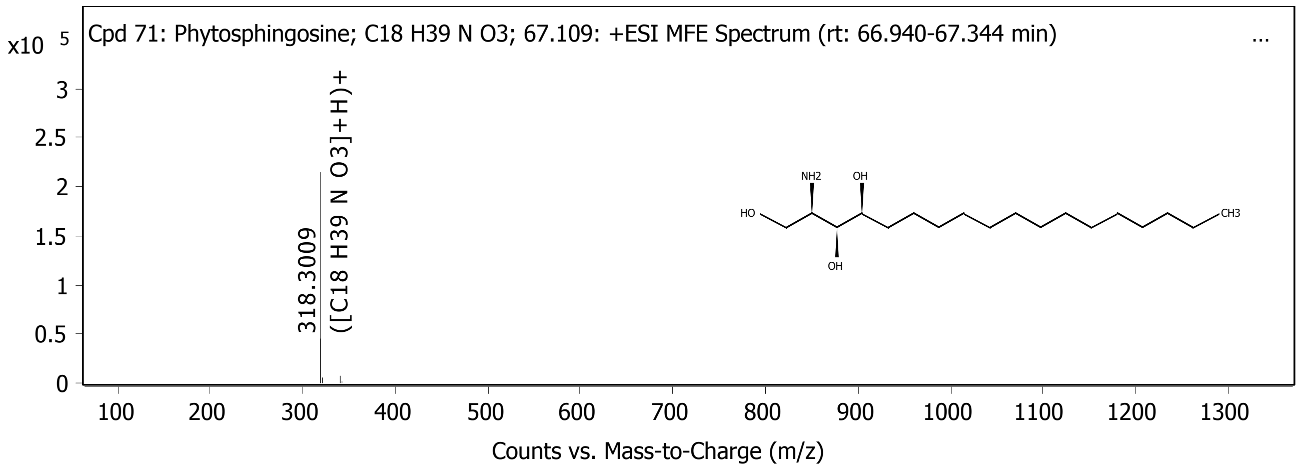

MFE MS Zoomed Spectrum

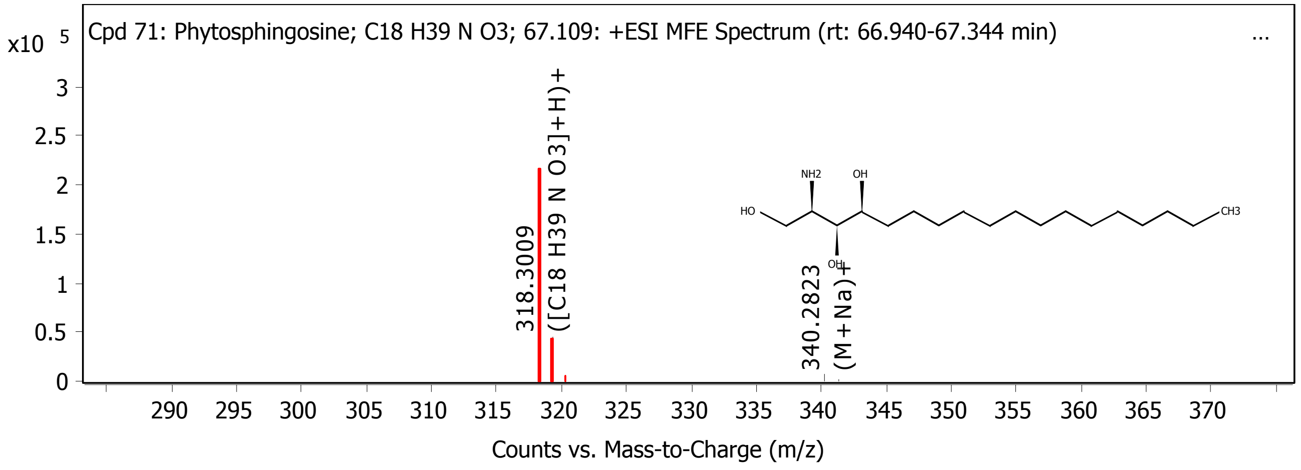

MS Spectrum

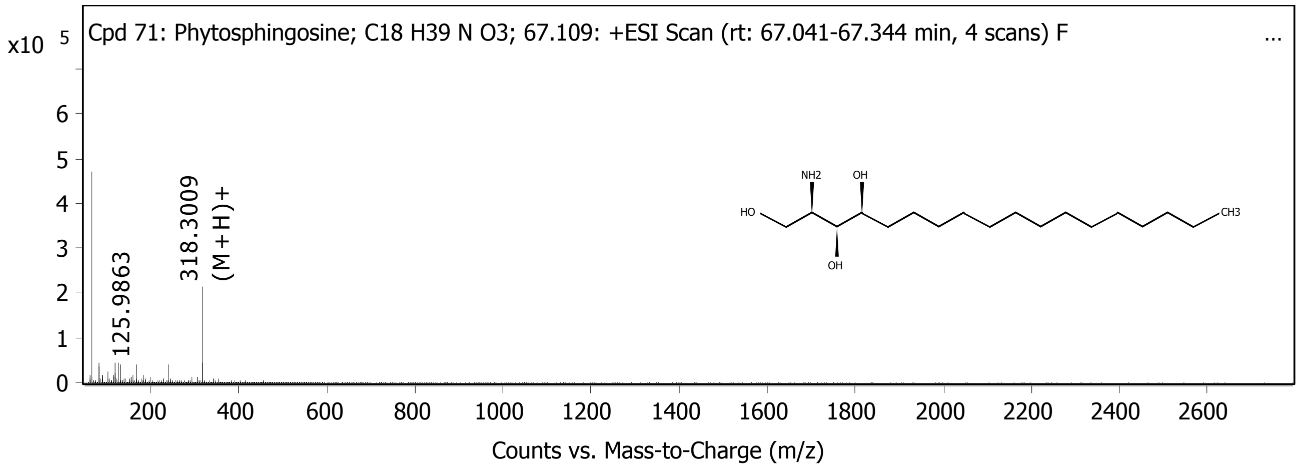

MS Zoomed Spectrum

Qualitative Compound Identification Report

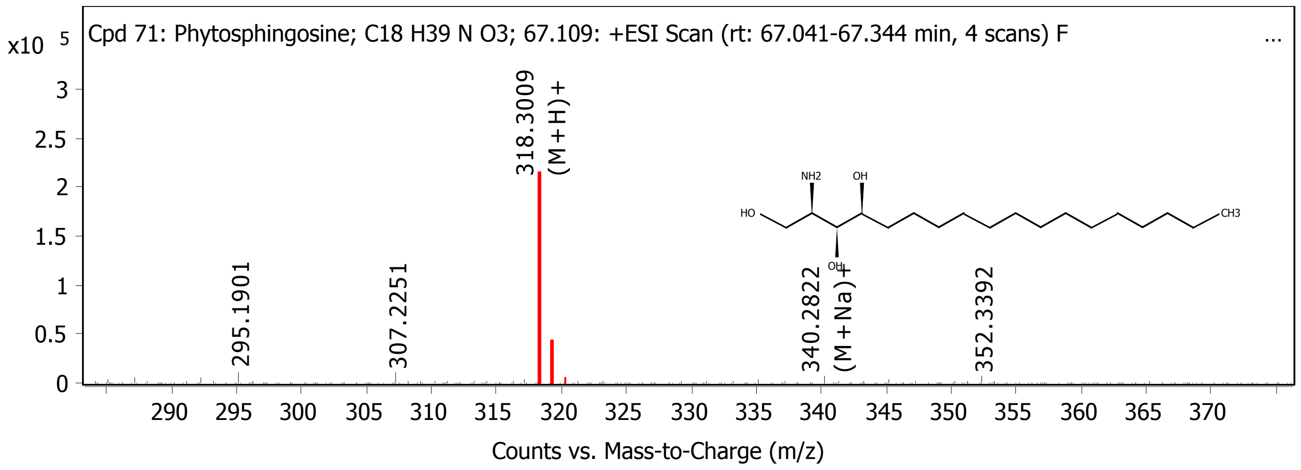

Identification Hit Table

Best Hit	Compound Name	RT	Formula	Notes	Match Score	Mass	Difference	Ion Species
✓	Phytosphingosine	67.109	C18 H39 N O3		98.58	317.2936	-0.62	(M+H)+

Identification Hit Table

Best Hit	Compound Name	RT	Formula	Notes	Match Score	Mass	Difference	Ion Species
✓	Phytosphingosine	67.109	C18 H39 N O3		98.58	317.2936	-0.62	(M+H)+

Identification Hit Table

Best Hit	Compound Name	RT	Formula	Notes	Match Score	Mass	Difference	Ion Species

Compound Label	Name	*m/z*	RT	Algorithm	Mass
Cpd 72: Xanthyletine; C14 H12 O3; 68.525	**Xanthyletine**	233.0575	68.525	Find by Molecular Feature	228.0788

Compound Chromatograms

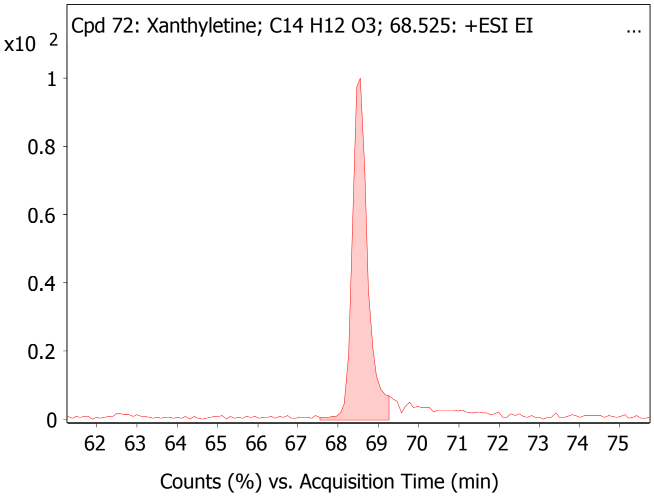

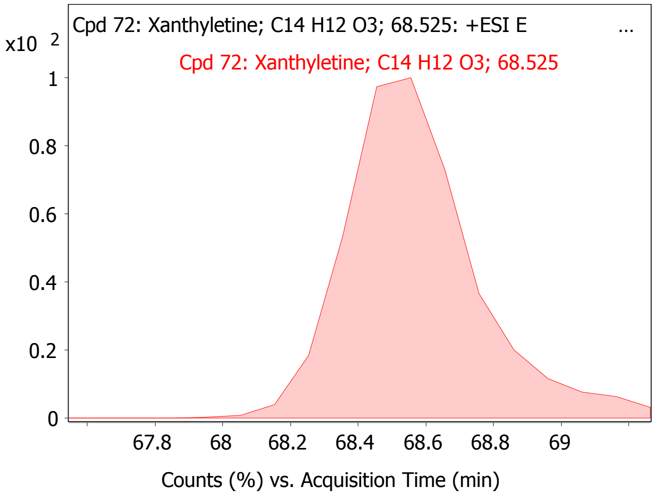

MFE MS Spectrum

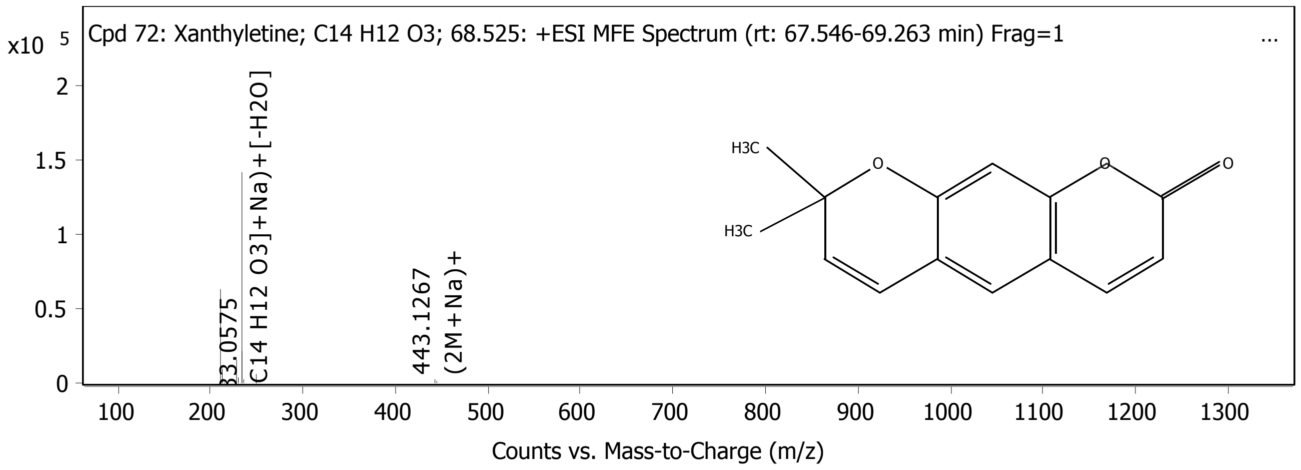

MFE MS Zoomed Spectrum

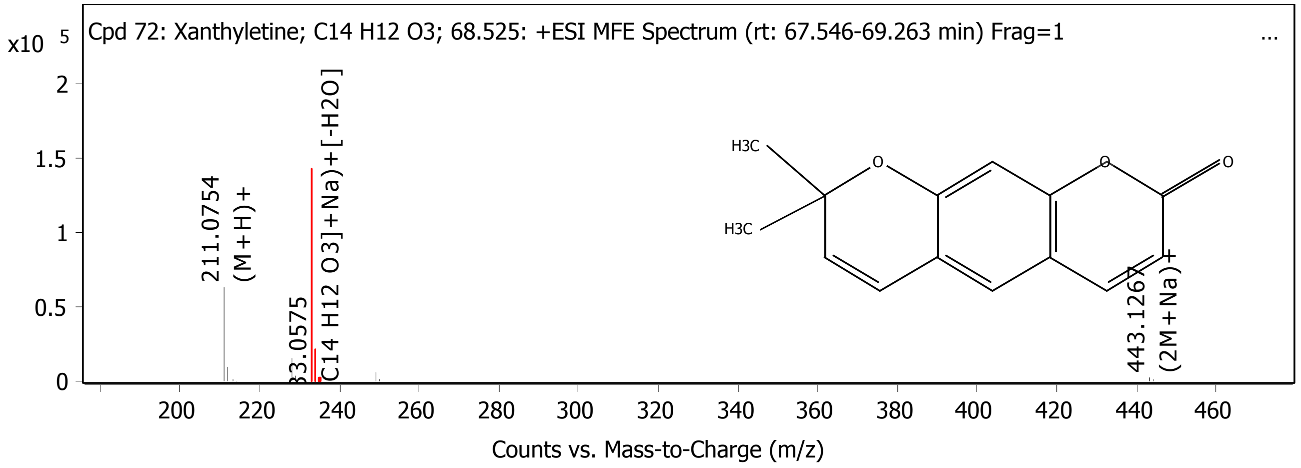

MS Spectrum

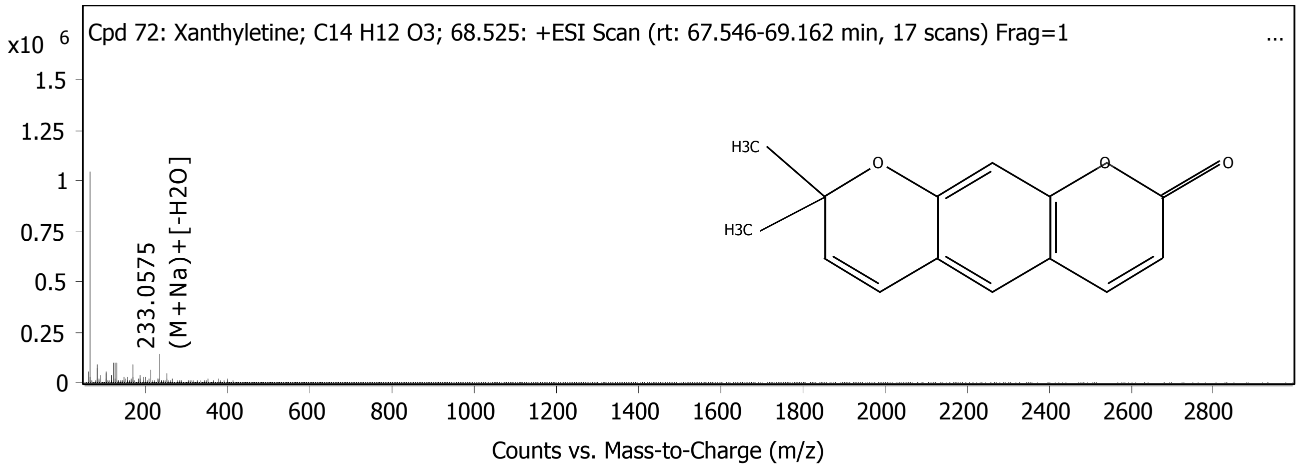

MS Zoomed Spectrum

Qualitative Compound Identification Report

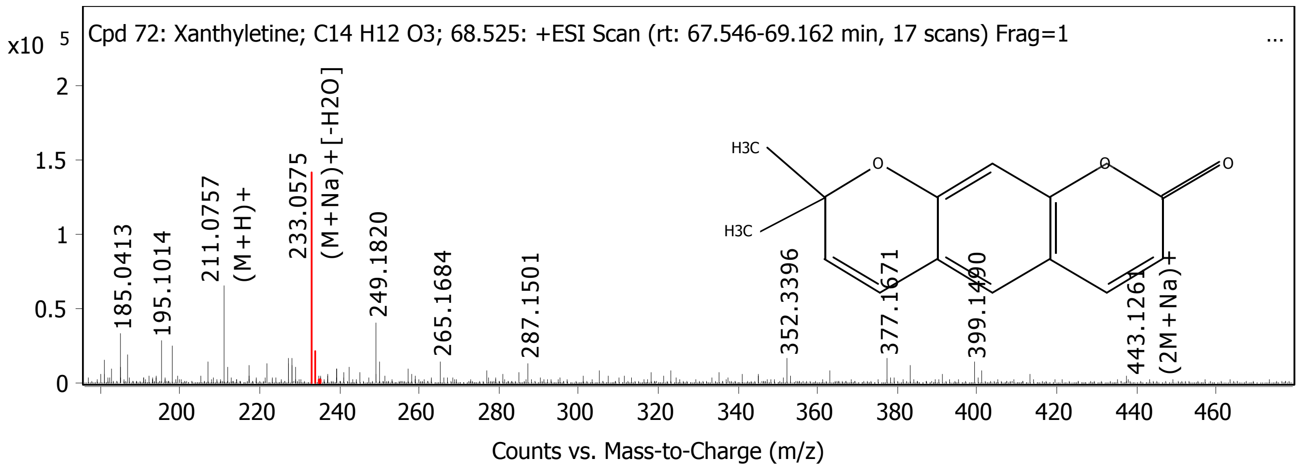

Identification Hit Table

Best Hit	Compound Name	RT	Formula	Notes	Match Score	Mass	Difference	Ion Species
✓	Xanthyletine	68.525	C14 H12 O3	Zanthoxylum spp	99.8	228.0788	-0.19	(M+Na)+[-H2O]
	o-Tolyl salicylate	68.525	C14 H12 O3		99.8	228.0788	-0.19	(M+Na)+[-H2O]
	Seselin	68.525	C14 H12 O3		99.8	228.0788	-0.19	(M+Na)+[-H2O]
	4'-Hydroxy-4-biphenylacetic acid	68.525	C14 H12 O3	prodrug used for analgesic and antipyretic action. metabolite of Fenbufen drug Dollery, Colin Therapeutic Drugs, 2nd Ed. 1999 p. F26	99.8	228.0788	-0.19	(M+Na)+[-H2O]
	trans-Resveratrol	68.525	C14 H12 O3		99.8	228.0788	-0.19	(M+Na)+[-H2O]
	Oxybenzone	68.525	C14 H12 O3	Pubchem 4632	99.8	228.0788	-0.19	(M+Na)+[-H2O]
	Trioxsalen	68.525	C14 H12 O3	Pubchem	99.8	228.0788	-0.19	(M+Na)+[-H2O]
	2,4-Difurfurylfuran	68.525	C14 H12 O3		99.8	228.0788	-0.19	(M+Na)+[-H2O]
	cis-Resveratrol	68.525	C14 H12 O3	Pubchem 1548910	99.8	228.0788	-0.19	(M+Na)+[-H2O]
	5,6-Dehydrokawain	68.525	C14 H12 O3		99.8	228.0788	-0.19	(M+Na)+[-H2O]

Identification Hit Table

Best Hit	Compound Name	RT	Formula	Notes	Match Score	Mass	Difference	Ion Species
✓	trans-Resveratrol	68.525	C14 H12 O3		99.8	228.0788	-0.19	(M+Na)+[-H2O]

Identification Hit Table

Best Hit	Compound Name	RT	Formula	Notes	Match Score	Mass	Difference	Ion Species

Compound Label	Name	*m/z*	RT	Algorithm	Mass
Cpd 73: Ser Ser Ser Ser; C12 H22 N4 O9; 74.364	**Ser Ser Ser Ser**	371.1177	74.364	Find by Molecular Feature	366.1393

Compound Chromatograms

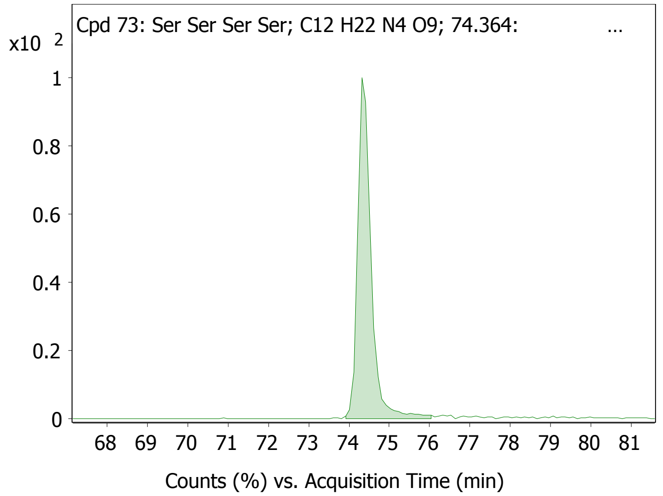

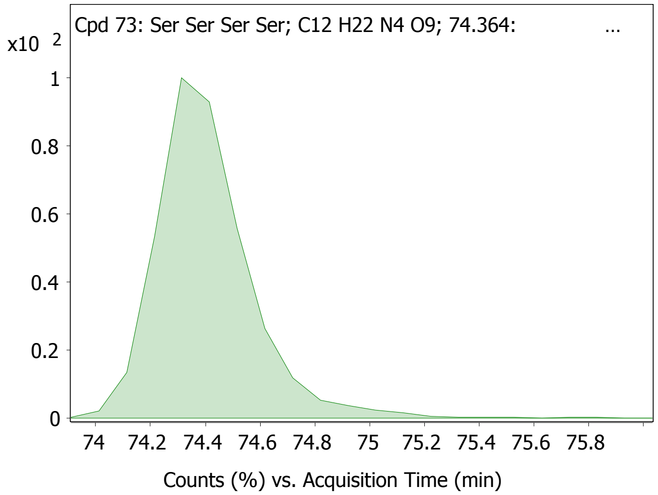

MFE MS Spectrum

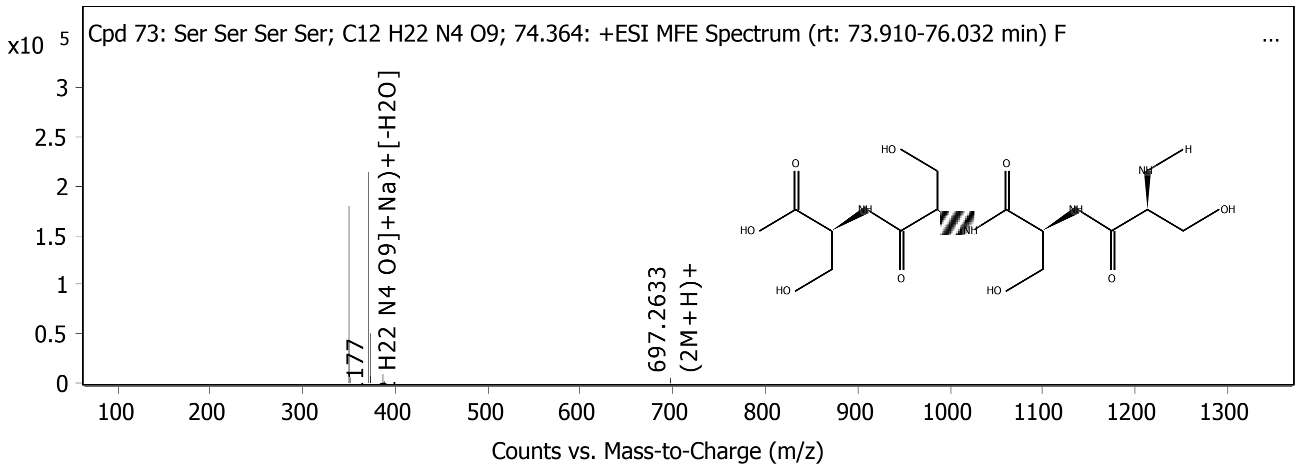

MFE MS Zoomed Spectrum

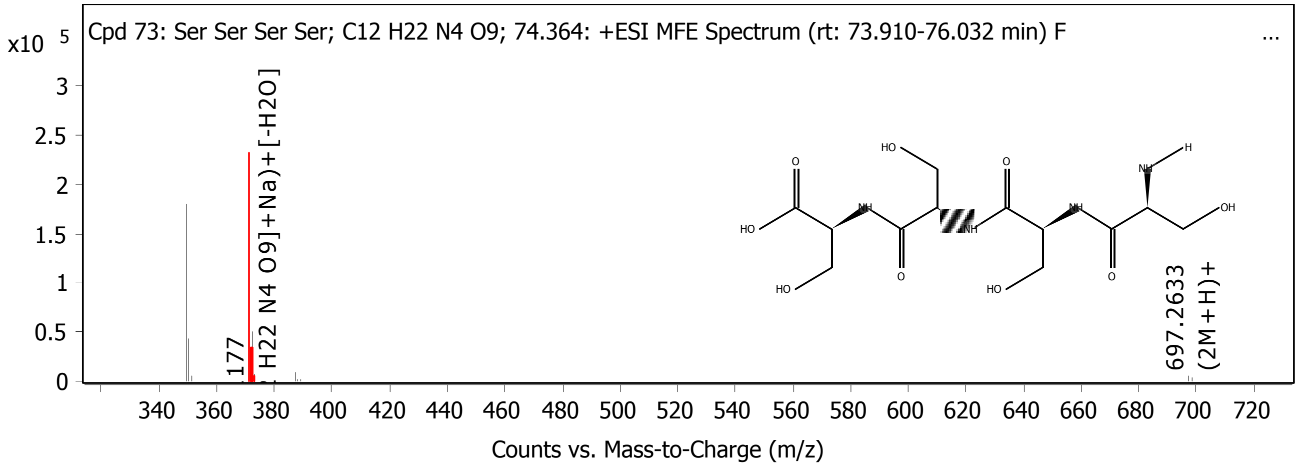

MS Spectrum

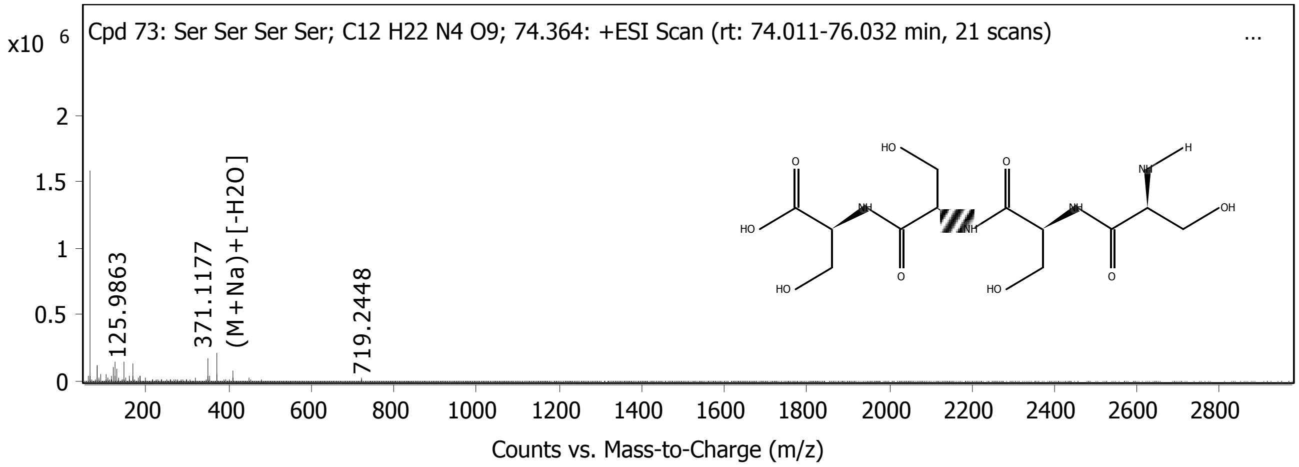

MS Zoomed Spectrum

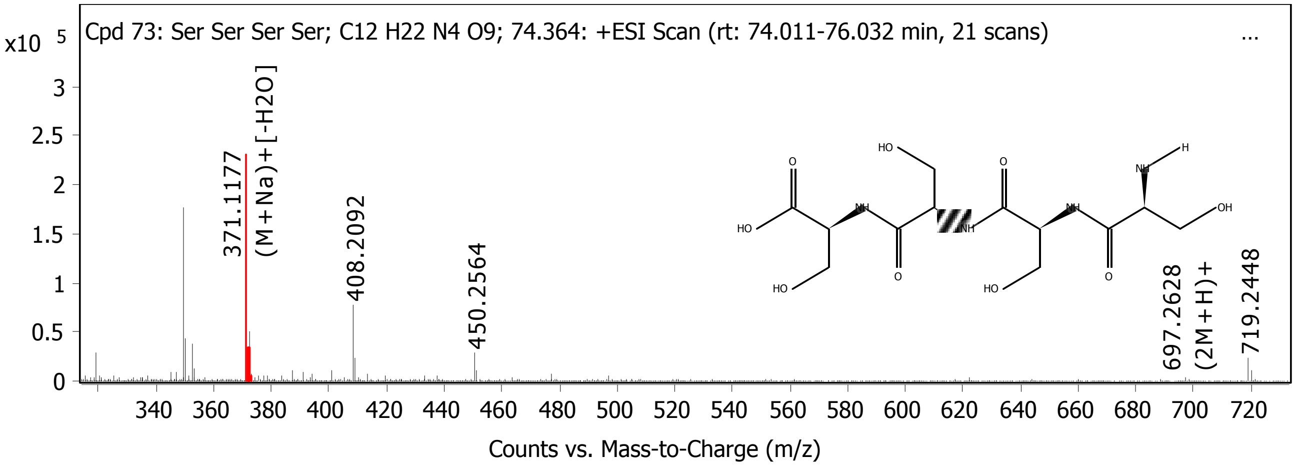

Identification Hit Table

Best Hit	Compound Name	RT	Formula	Notes	Match Score	Mass	Difference	Ion Species
✓	Asn Asp Thr	74.364	C12 H20 N4 O8		84.89	348.1287	-0.59	(M+Na)+
	Ser Gln Asp	74.364	C12 H20 N4 O8		84.89	348.1287	-0.59	(M+Na)+
	Asn Thr Asp	74.364	C12 H20 N4 O8		84.89	348.1287	-0.59	(M+Na)+
	Thr Asp Asn	74.364	C12 H20 N4 O8		84.89	348.1287	-0.59	(M+Na)+
	Asp Thr Asn	74.364	C12 H20 N4 O8		84.89	348.1287	-0.59	(M+Na)+
	Ser Glu Asn	74.364	C12 H20 N4 O8		84.89	348.1287	-0.59	(M+Na)+
	Asn Glu Ser	74.364	C12 H20 N4 O8		84.89	348.1287	-0.59	(M+Na)+
	Gln Asp Ser	74.364	C12 H20 N4 O8		84.89	348.1287	-0.59	(M+Na)+
	Ser Asp Gln	74.364	C12 H20 N4 O8		84.89	348.1287	-0.59	(M+Na)+
	Thr Asn Asp	74.364	C12 H20 N4 O8		84.89	348.1287	-0.59	(M+Na)+

Identification Hit Table

Best Hit	Compound Name	RT	Formula	Notes	Match Score	Mass	Difference	Ion Species

Identification Hit Table

Best Hit	Compound Name	RT	Formula	Notes	Match Score	Mass	Difference	Ion Species
✓	Ser Ser Ser Ser	74.364	C12 H22 N4 O9		84.93	366.1393	-0.59	(M+Na)+[-H2O]
	Ala Gly Ser Asp	74.364	C12 H20 N4 O8		84.89	348.1287	-0.59	(M+Na)+
	Asn Ser Glu	74.364	C12 H20 N4 O8		84.89	348.1287	-0.59	(M+Na)+
	Asp Ser Ala Gly	74.364	C12 H20 N4 O8		84.89	348.1287	-0.59	(M+Na)+
	Asp Gly Thr Gly	74.364	C12 H20 N4 O8		84.89	348.1287	-0.59	(M+Na)+
	Asp Gly Ser Ala	74.364	C12 H20 N4 O8		84.89	348.1287	-0.59	(M+Na)+
	Asp Gly Gly Thr	74.364	C12 H20 N4 O8		84.89	348.1287	-0.59	(M+Na)+
	Asp Gly Ala Ser	74.364	C12 H20 N4 O8		84.89	348.1287	-0.59	(M+Na)+
	Asp Ala Ser Gly	74.364	C12 H20 N4 O8		84.89	348.1287	-0.59	(M+Na)+
	Asp Ala Gly Ser	74.364	C12 H20 N4 O8		84.89	348.1287	-0.59	(M+Na)+

Compound Label	Name	*m/z*	RT	Algorithm	Mass
Cpd 74: 4-Phenyl-2-butenal; C10 H10 O; 74.365	**4-Phenyl-2-butenal**	147.0805	74.365	Find by Molecular Feature	146.0732

Compound Chromatograms

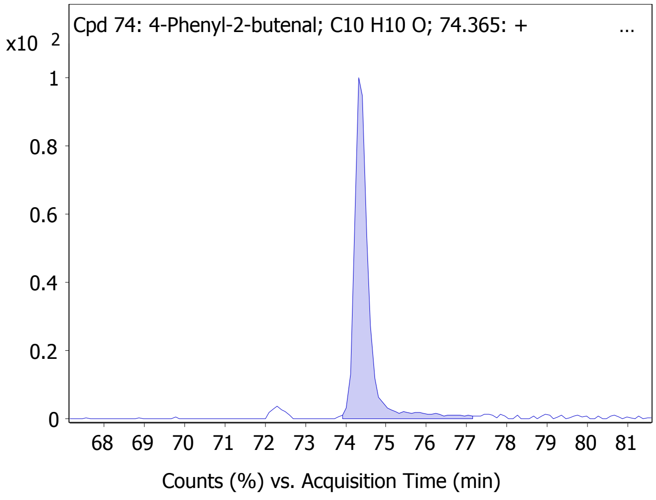

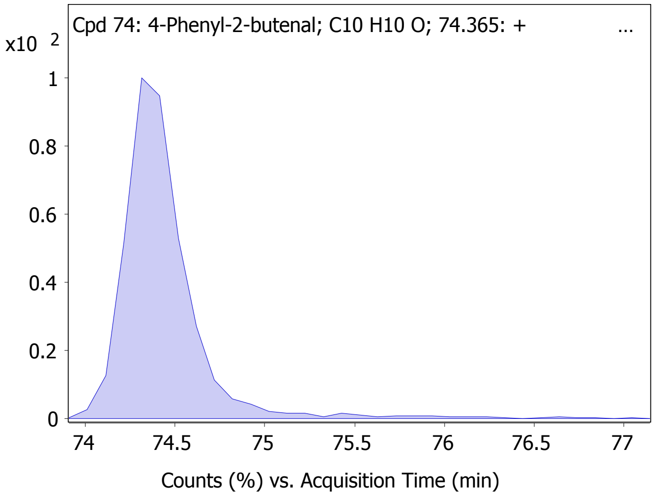

MFE MS Spectrum

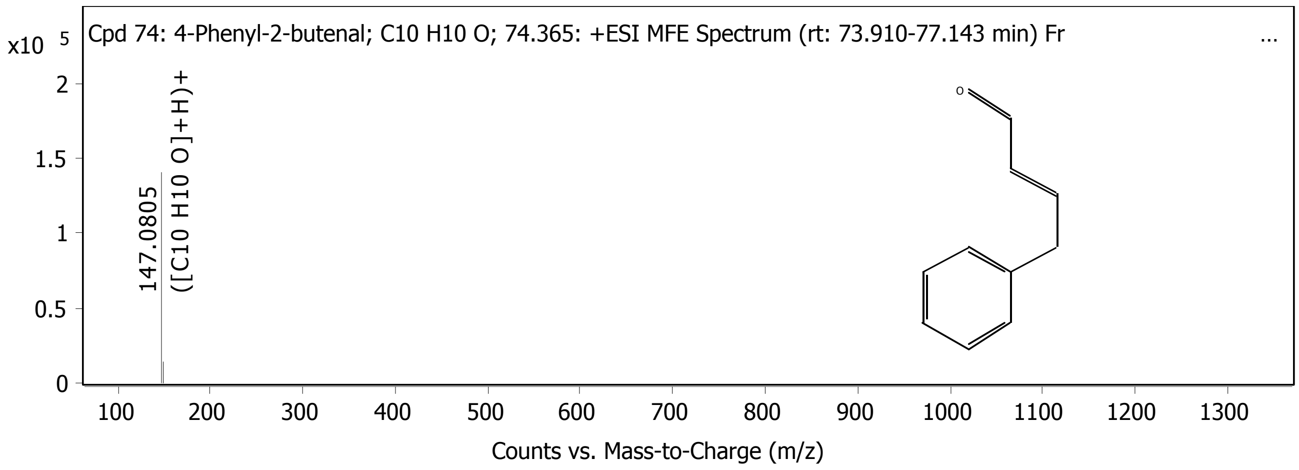

MFE MS Zoomed Spectrum

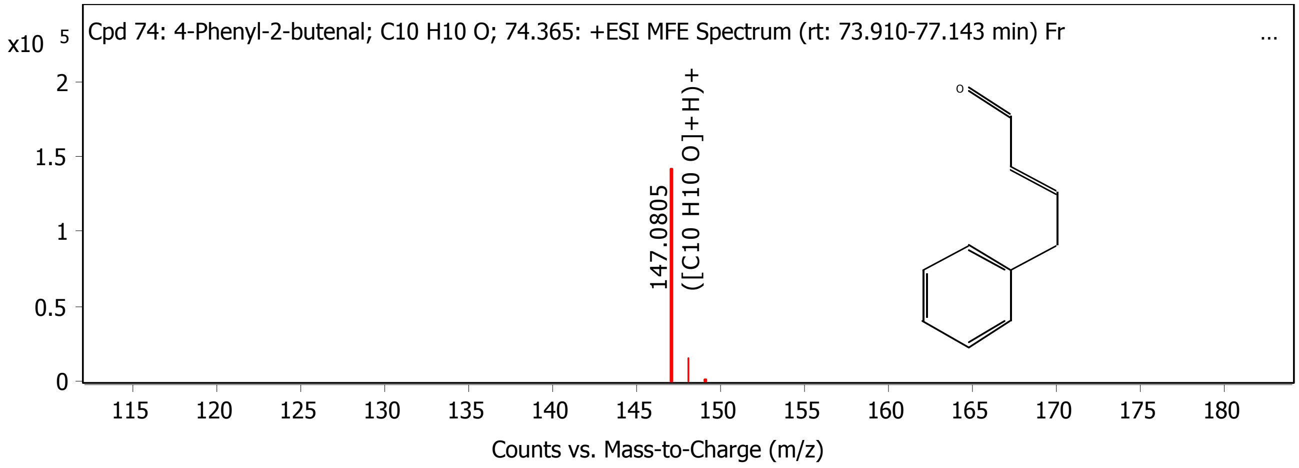

MS Spectrum

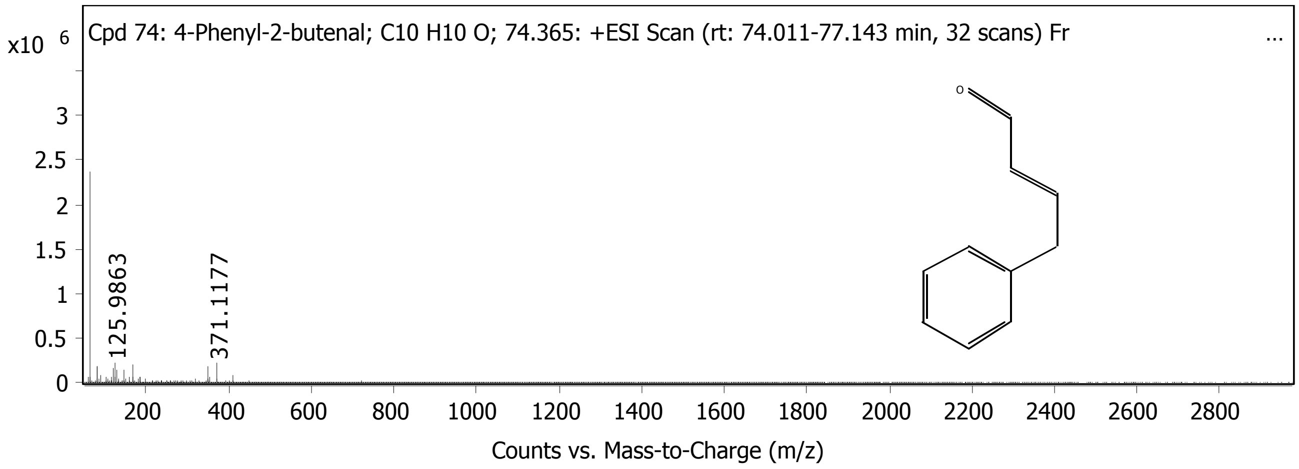

MS Zoomed Spectrum

Qualitative Compound Identification Report

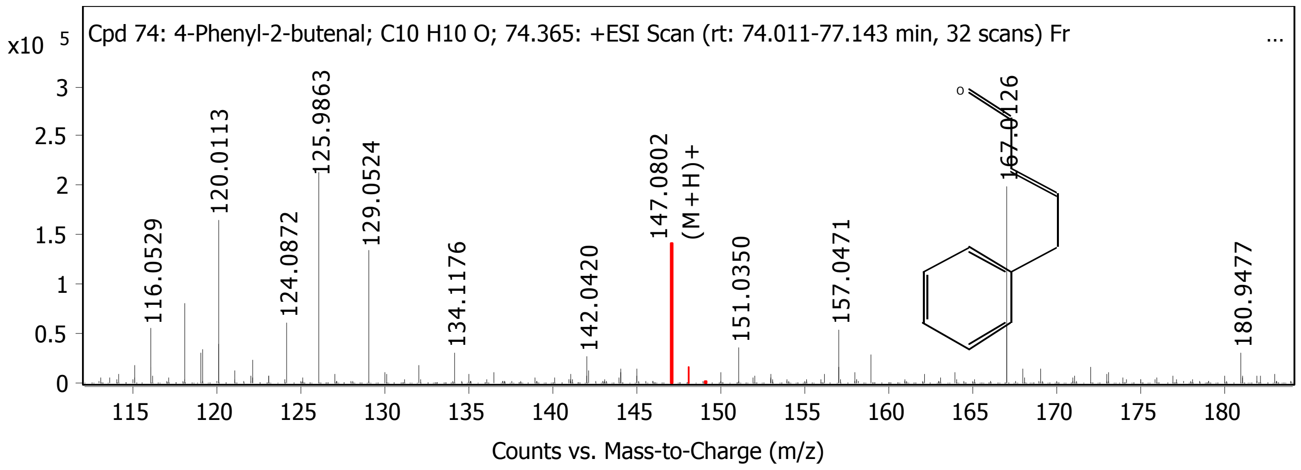

Identification Hit Table

Best Hit	Compound Name	RT	Formula	Notes	Match Score	Mass	Difference	Ion Species
✓	4-Phenyl-2-butenal	74.365	C10 H10 O		99.55	146.0732	0	(M+H)+
	3-(4-Methylphenyl)-2-propenal	74.365	C10 H10 O		99.55	146.0732	0	(M+H)+
	2-Methyl-3-phenyl-2-propenal	74.365	C10 H10 O		99.55	146.0732	0	(M+H)+
	Benzylideneacetone	74.365	C10 H10 O		99.55	146.0732	0	(M+H)+
	2-Phenyl-2-butenal	74.365	C10 H10 O		99.55	146.0732	0	(M+H)+
	2,3-Dimethylbenzofuran	74.365	C10 H10 O		99.55	146.0732	0	(M+H)+
	4-(1-Methylethenyl)benzaldehyde	74.365	C10 H10 O		99.55	146.0732	0	(M+H)+
	Evodone	74.365	C10 H12 O2		99.55	164.0837	0	(M+H)+[-H2O]
	4-Isopropylbenzoic acid	74.365	C10 H12 O2		99.55	164.0837	0	(M+H)+[-H2O]
	Phenethyl acetate	74.365	C10 H12 O2		99.55	164.0837	0	(M+H)+[-H2O]

Identification Hit Table

Best Hit	Compound Name	RT	Formula	Notes	Match Score	Mass	Difference	Ion Species
✓	2,4-Decadiynoic acid	74.365	C10 H12 O2		99.55	164.0837	0	(M+H)+[-H2O]

Identification Hit Table

Best Hit	Compound Name	RT	Formula	Notes	Match Score	Mass	Difference	Ion Species

Compound Label	Name	*m/z*	RT	Algorithm	Mass
Cpd 75: 3,8-Dihydroxy-6-methoxy-7(11)-eremophilen-12,8-olide; C16 H24 O5; 82.623	**3,8-Dihydroxy-6-methoxy-7(11)-eremophilen-12,8-olide**	301.1414	82.623	Find by Molecular Feature	296.1627

Compound Chromatograms

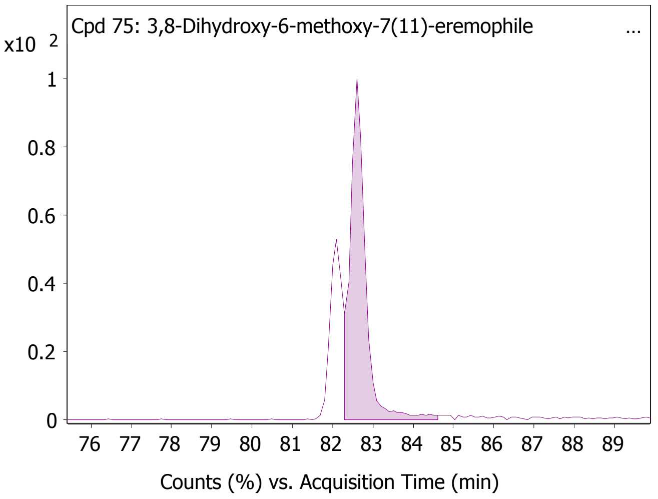

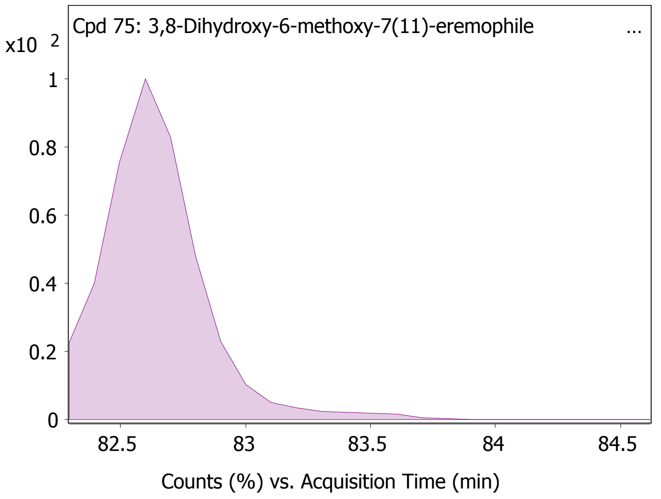

MFE MS Spectrum

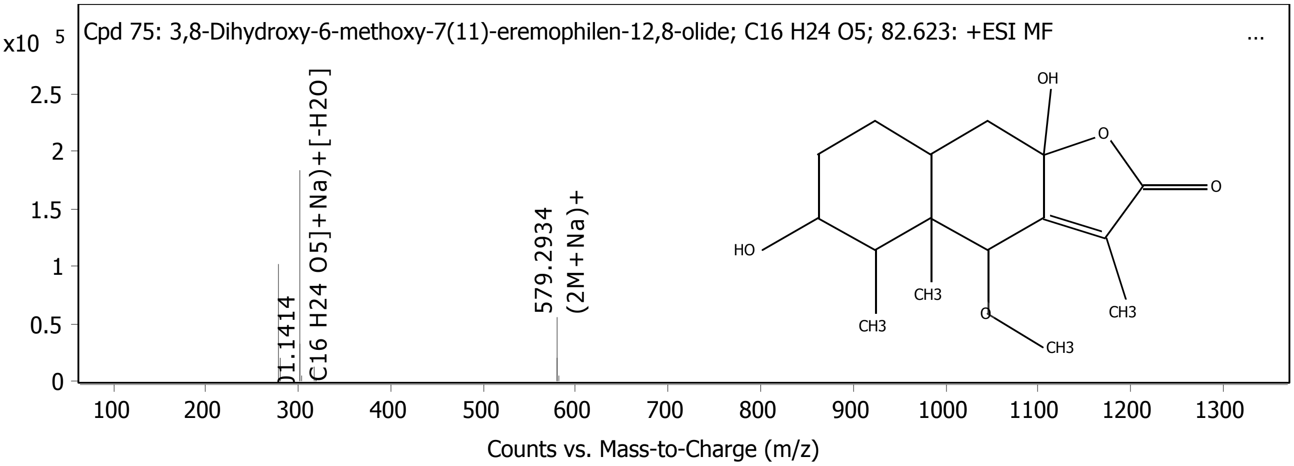

MFE MS Zoomed Spectrum

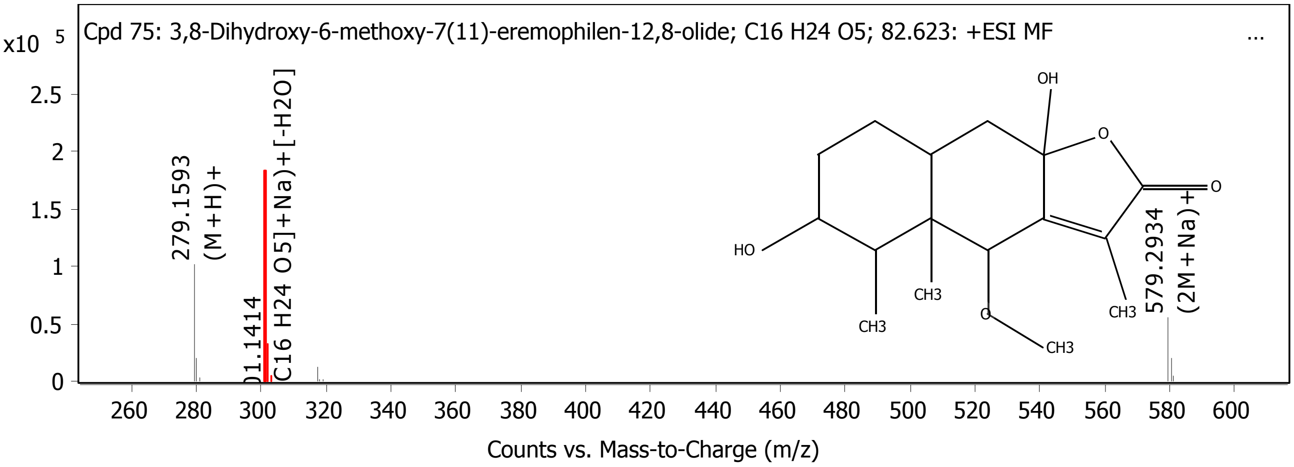

MS Spectrum

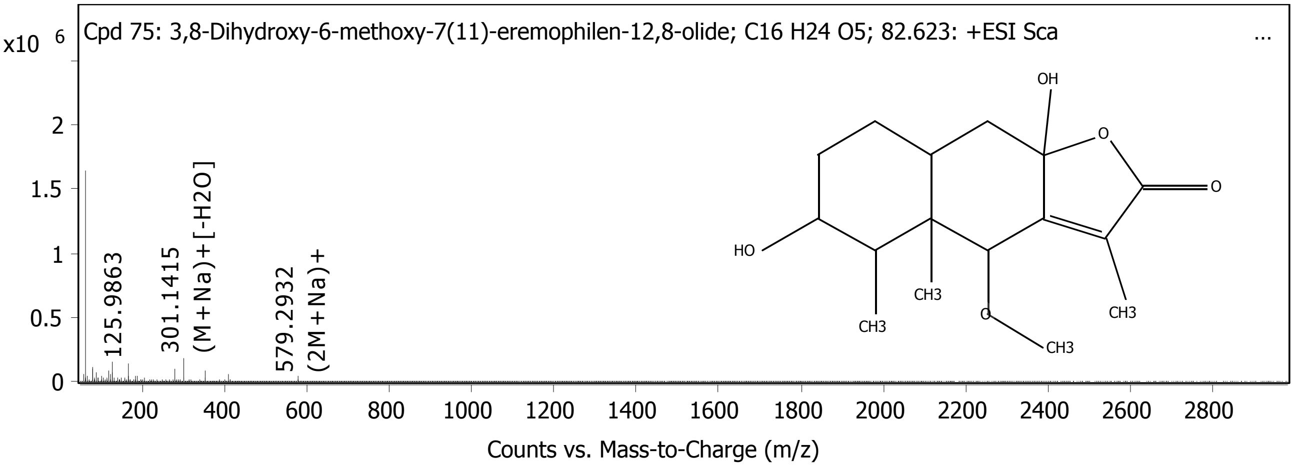

MS Zoomed Spectrum

Qualitative Compound Identification Report

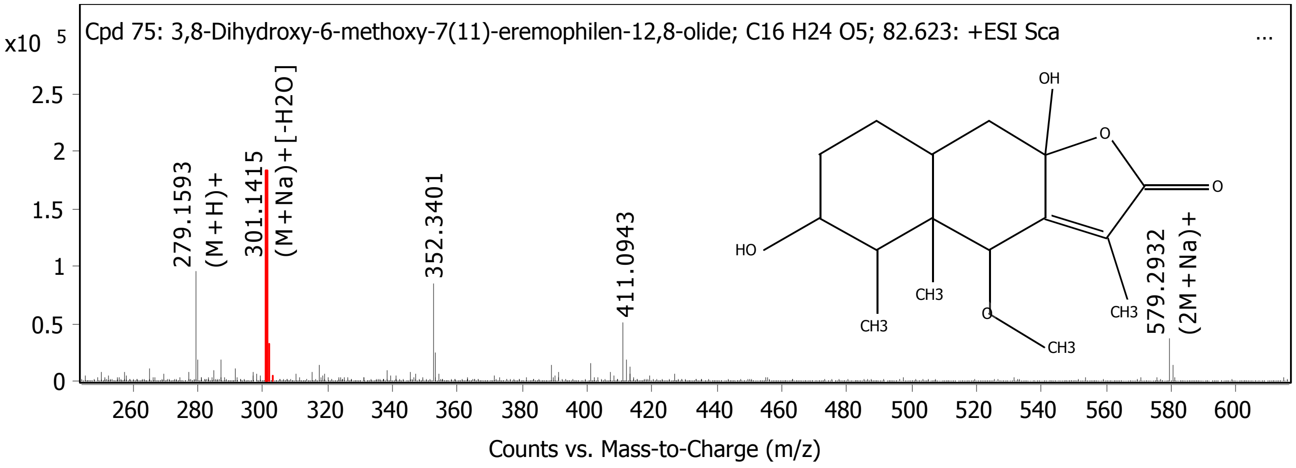

Identification Hit Table

Best Hit	Compound Name	RT	Formula	Notes	Match Score	Mass	Difference	Ion Species
✓	3,8-Dihydroxy-6-methoxy-7(11)-eremophilen-12,8-olide	82.623	C16 H24 O5		99.54	296.1627	-0.34	(M+Na)+[-H2O]
	Methyl dihydrophaseate	82.623	C16 H24 O5		99.54	296.1627	-0.34	(M+Na)+[-H2O]
	Graphinone	82.623	C16 H24 O5		99.54	296.1627	-0.34	(M+Na)+[-H2O]
	alhpa-tocopheronic acid	82.623	C16 H24 O5		99.54	296.1627	-0.34	(M+Na)+[-H2O]
	Lactone of PGF-MUM	82.623	C16 H24 O5	naturally occurring prostaglandin. Used in the 2nd trimester of pregnancy to induce abortion. and is used for medical treatment of tubal pregnancy and induction of labor at term. metabolite of Dinoprost (prostagland F2a) drug Dollery, Colin Thera	99.54	296.1627	-0.34	(M+Na)+[-H2O]
	Diisobutyl phthalate	82.623	C16 H22 O4		99.51	278.1521	-0.34	(M+Na)+
	Dibutyl phthalate	82.623	C16 H22 O4		99.51	278.1521	-0.34	(M+Na)+
	Alpha-CEHC	82.623	C16 H22 O4		99.51	278.1521	-0.34	(M+Na)+
	alhpa-tocopheronolactone	82.623	C16 H22 O4		99.51	278.1521	-0.34	(M+Na)+
	Emmotin A	82.623	C16 H22 O4		99.51	278.1521	-0.34	(M+Na)+

Identification Hit Table

Best Hit	Compound Name	RT	Formula	Notes	Match Score	Mass	Difference	Ion Species
✓	alhpa-tocopheronic acid	82.623	C16 H24 O5		99.54	296.1627	-0.34	(M+Na)+[-H2O]
	Emmotin A	82.623	C16 H22 O4		99.51	278.1521	-0.34	(M+Na)+
	alhpa-tocopheronolactone	82.623	C16 H22 O4		99.51	278.1521	-0.34	(M+Na)+

Identification Hit Table

Best Hit	Compound Name	RT	Formula	Notes	Match Score	Mass	Difference	Ion Species

Compound Label	Name	*m/z*	RT	Algorithm	Mass
Cpd 76: Pyranodelphinin B; C24 H23 O12; 89.650	**Pyranodelphinin B**	485.1132	89.65	Find by Molecular Feature	503.1218

Compound Chromatograms

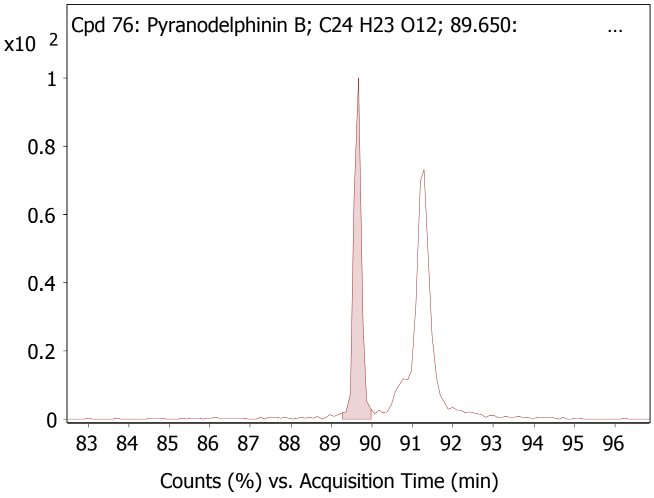

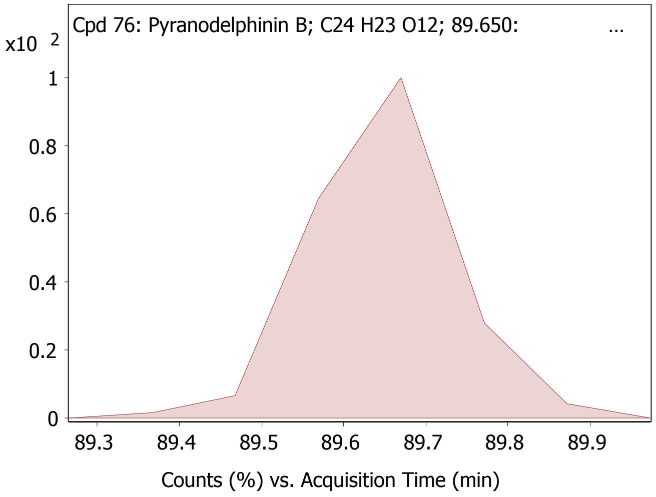

MFE MS Spectrum

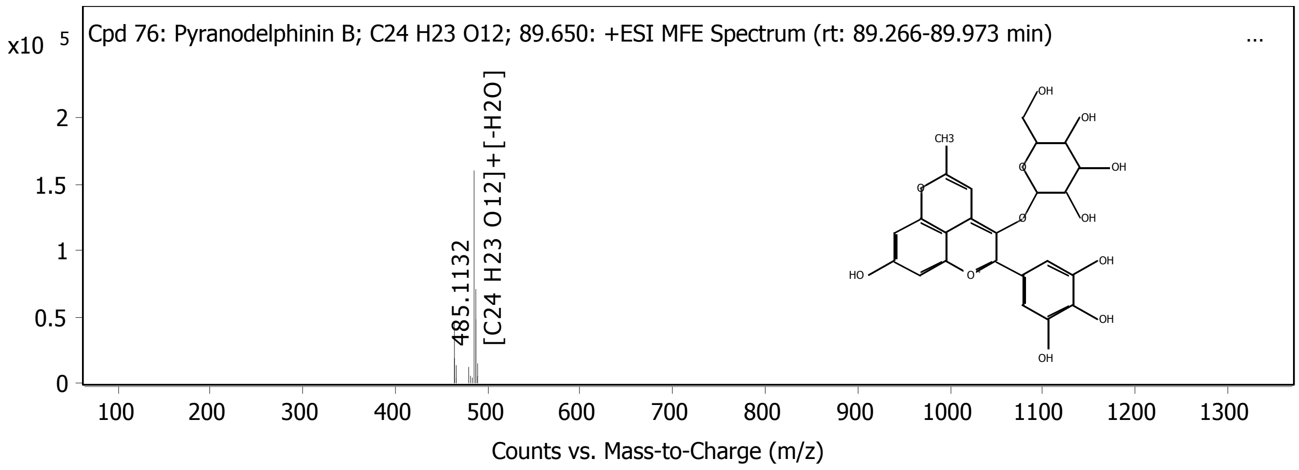

MFE MS Zoomed Spectrum

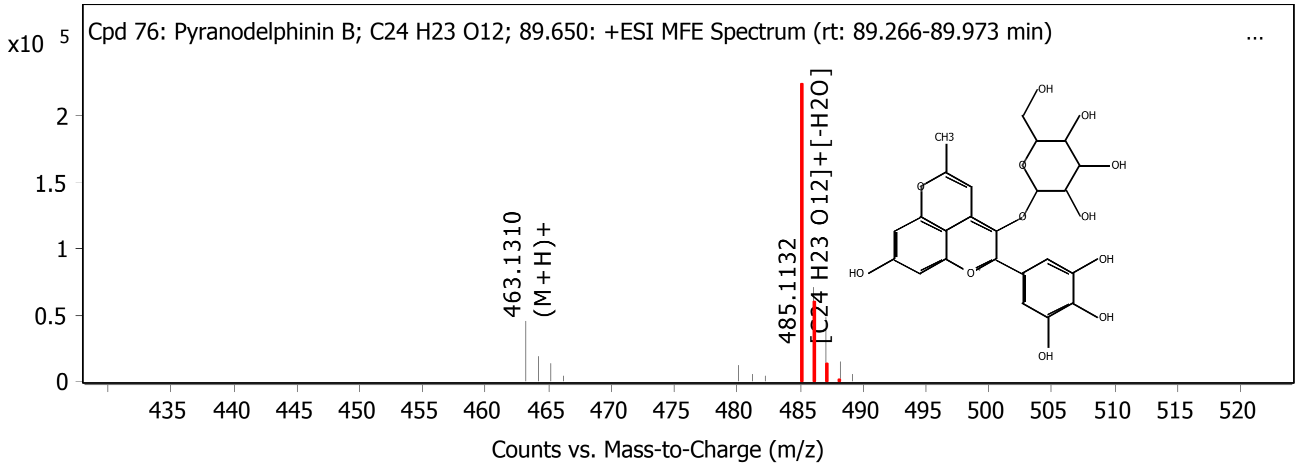

MS Spectrum

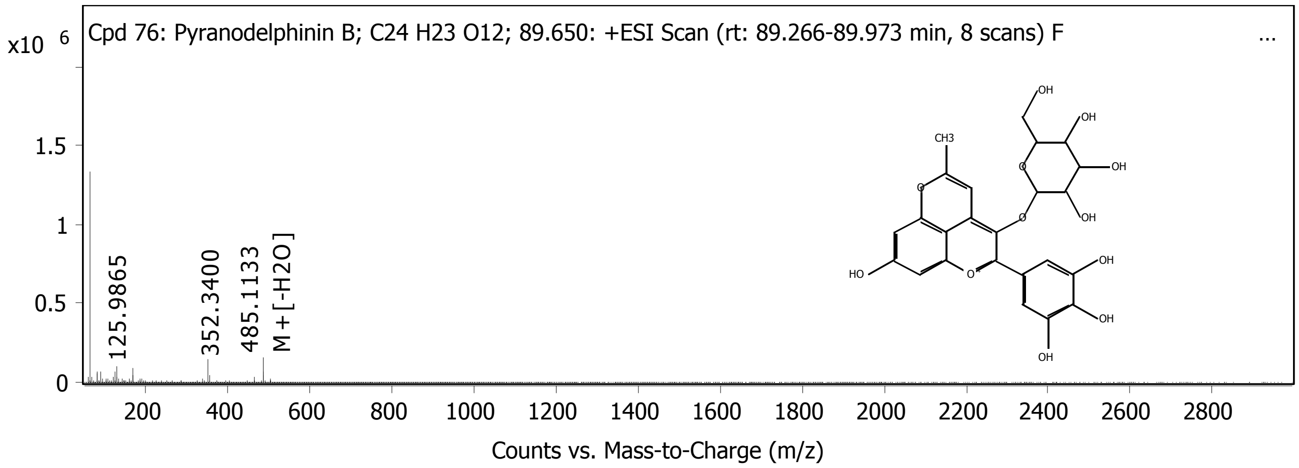

MS Zoomed Spectrum

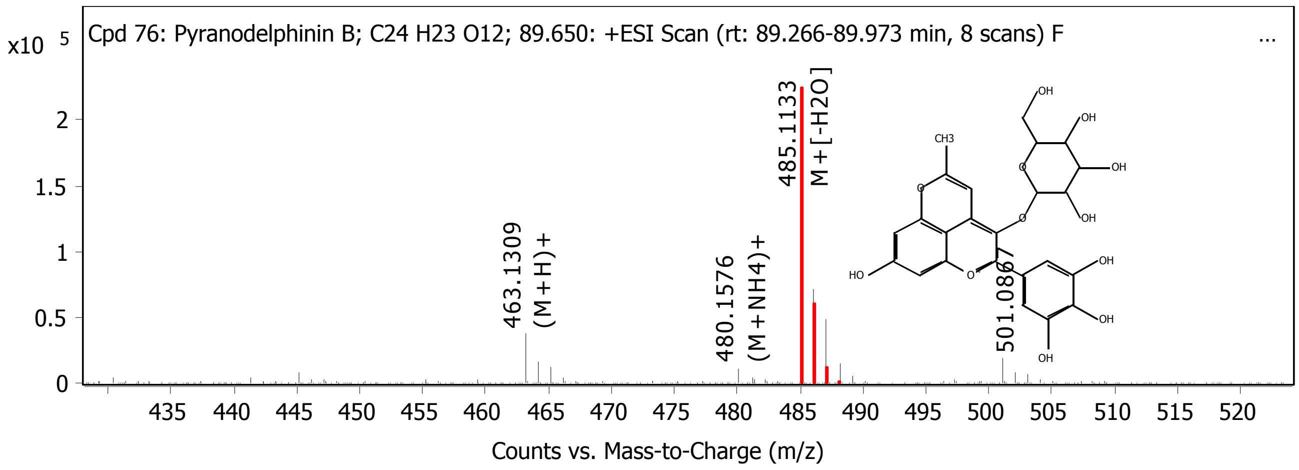

Identification Hit Table

Best Hit	Compound Name	RT	Formula	Notes	Match Score	Mass	Difference	Ion Species
✓	Pyranodelphinin B	89.65	C24 H23 O12		45.74	503.1218	-2.81	M+[-H2O]

Identification Hit Table

Best Hit	Compound Name	RT	Formula	Notes	Match Score	Mass	Difference	Ion Species

Identification Hit Table

Best Hit	Compound Name	RT	Formula	Notes	Match Score	Mass	Difference	Ion Species

Compound Label	Name	*m/z*	RT	Algorithm	Mass
Cpd 77: MG(16:0/0:0/0:0)[rac]; C19 H38 O4; 95.382	**MG(16:0/0:0/0:0)[rac]**	353.2669	95.382	Find by Molecular Feature	330.2778

Compound Chromatograms

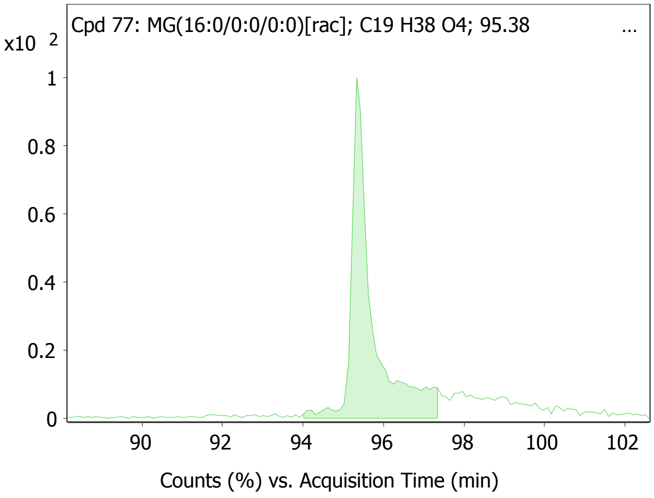

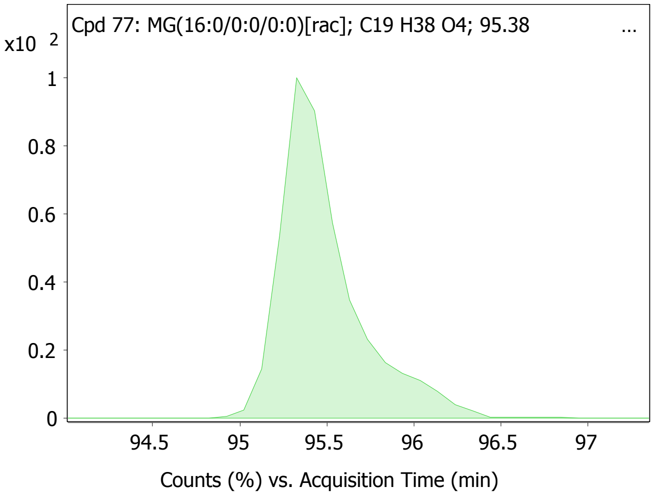

MFE MS Spectrum

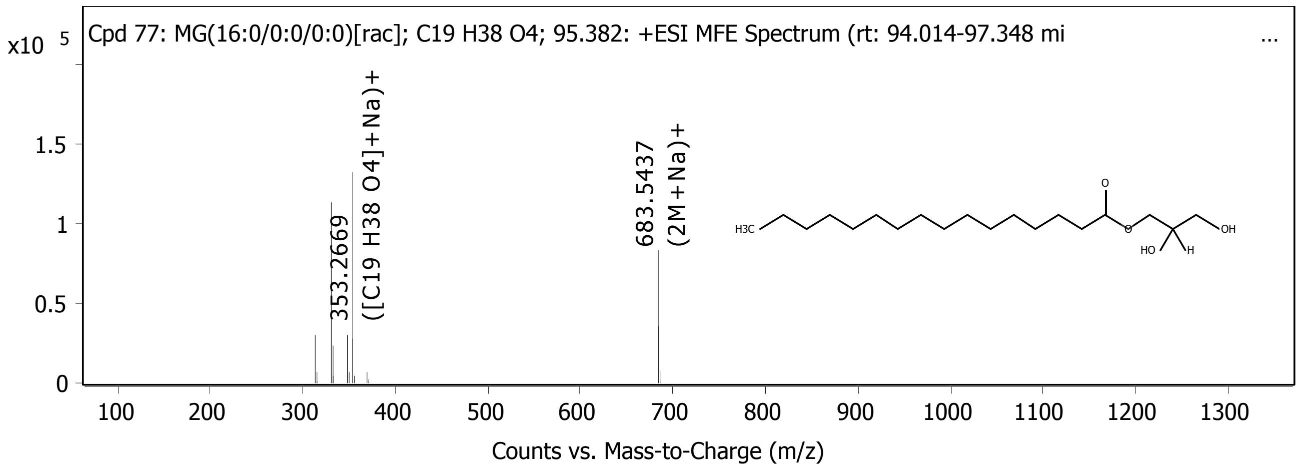

MFE MS Zoomed Spectrum

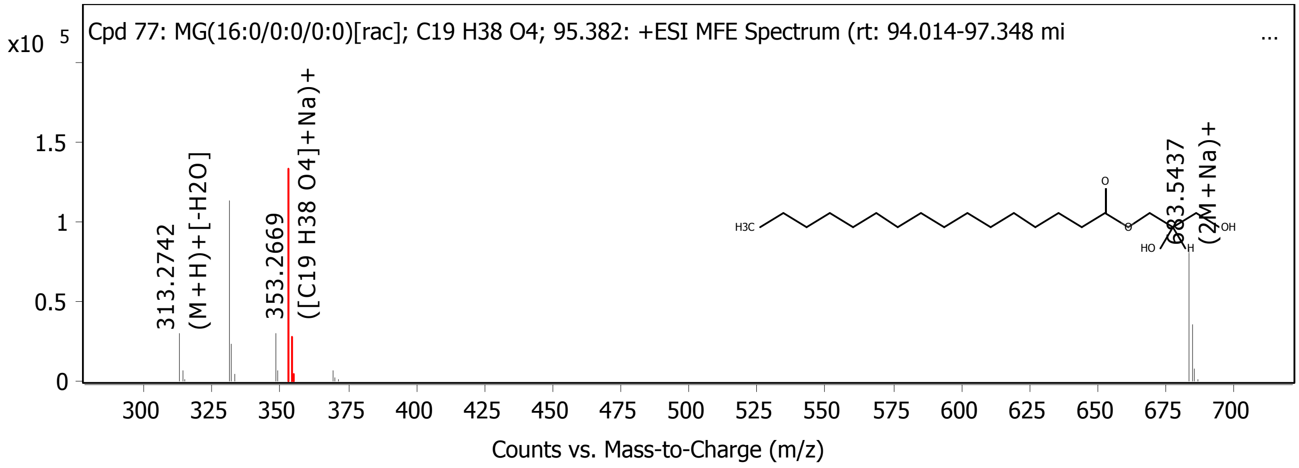

MS Spectrum

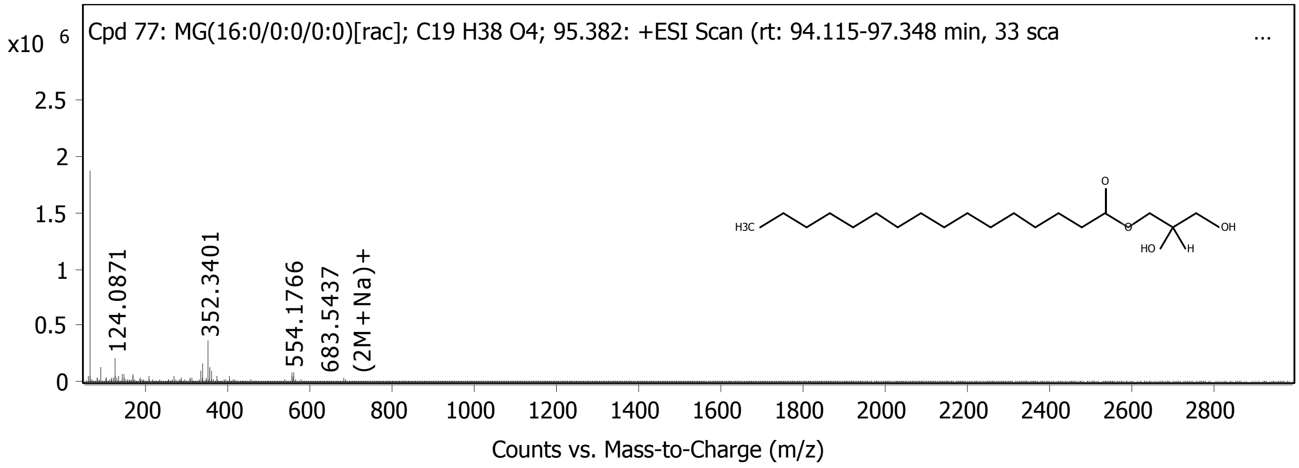

MS Zoomed Spectrum

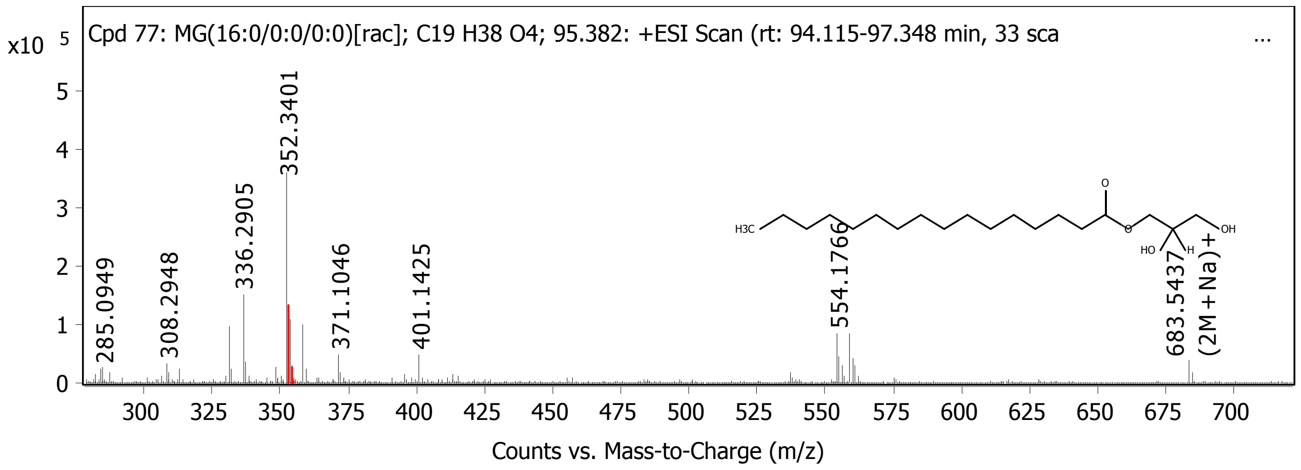

MSMS Spectrum

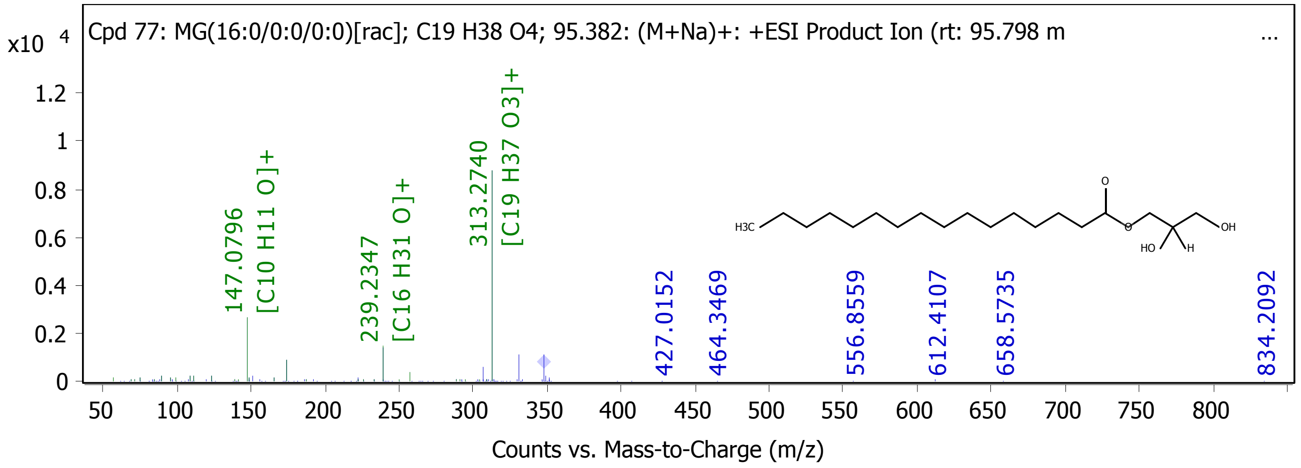

Identification Hit Table

Best Hit	Compound Name	RT	Formula	Notes	Match Score	Mass	Difference	Ion Species
✓	MG(16:0/0:0/0:0)[rac]	95.382	C19 H38 O4		97.98	330.2778	-0.76	(M+Na)+
	MG(0:0/16:0/0:0)	95.382	C19 H38 O4		97.98	330.2778	-0.76	(M+Na)+
	1-Monopalmitin	95.382	C19 H38 O4	1-Monopalmitin Palmitin, 1-mono-1-Palmitoylglycerol alpha-Monopalmitin Glycerol 1-palmitate Glycerol 3-palmitate Palmitoyl glycerol 1-Monopalmitoylglycerol .alpha.-Monopalmitin DL-alpha-Palmitin http://pubchem.ncbi.nlm.nih.gov/summary/sum	97.98	330.2778	-0.76	(M+Na)+
	MG(16:0/0:0/0:0)	95.382	C19 H38 O4	MG(16:0/0:0/0:0) http://www.lipidmaps.org/data/get_lm_lipids_dbgif.php?LM_ID=LMGL01010009&NAME=MG(16:0/0:0/0:0)	97.98	330.2778	-0.76	(M+Na)+

Identification Hit Table

Best Hit	Compound Name	RT	Formula	Notes	Match Score	Mass	Difference	Ion Species
✓	MG(16:0/0:0/0:0)[rac]	95.382	C19 H38 O4		97.98	330.2778	-0.76	(M+Na)+
	MG(16:0/0:0/0:0)	95.382	C19 H38 O4	MG(16:0/0:0/0:0) http://www.lipidmaps.org/data/get_lm_lipids_dbgif.php?LM_ID=LMGL01010009&NAME=MG(16:0/0:0/0:0)	97.98	330.2778	-0.76	(M+Na)+

Identification Hit Table

Best Hit	Compound Name	RT	Formula	Notes	Match Score	Mass	Difference	Ion Species

Compound Label	Name	*m/z*	RT	Algorithm	Mass
Cpd 78: Oleoyl sarcosine; C21 H39 N O3; 95.541	**Oleoyl sarcosine**	336.2905	95.541	Find by Molecular Feature	353.2939

Compound Chromatograms

Qualitative Compound Identification Report

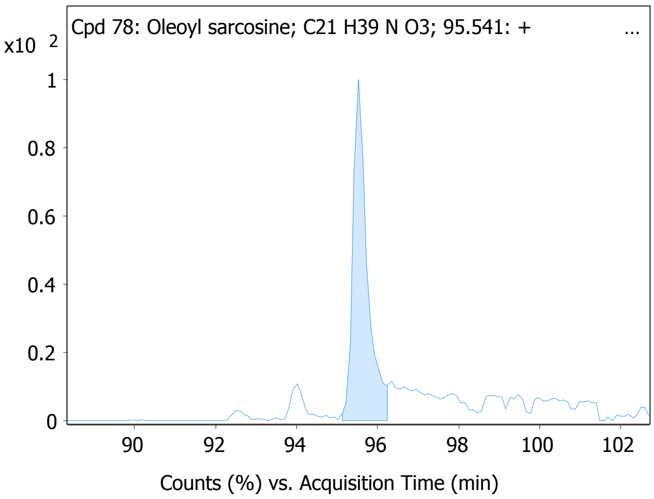

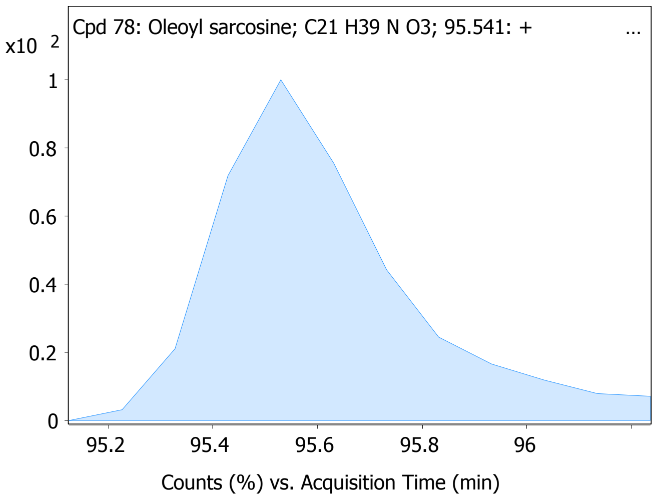

MFE MS Spectrum

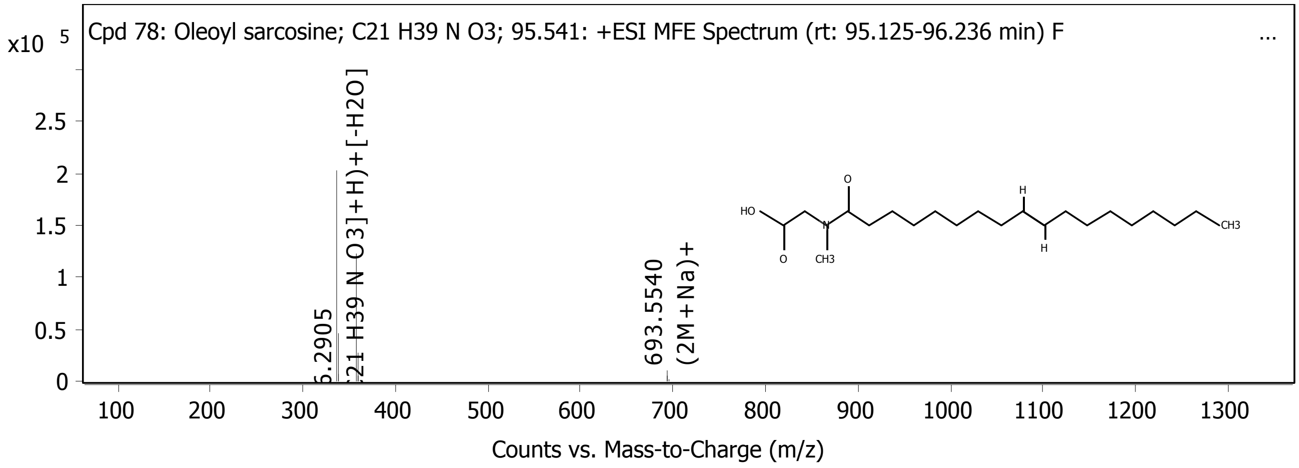

MFE MS Zoomed Spectrum

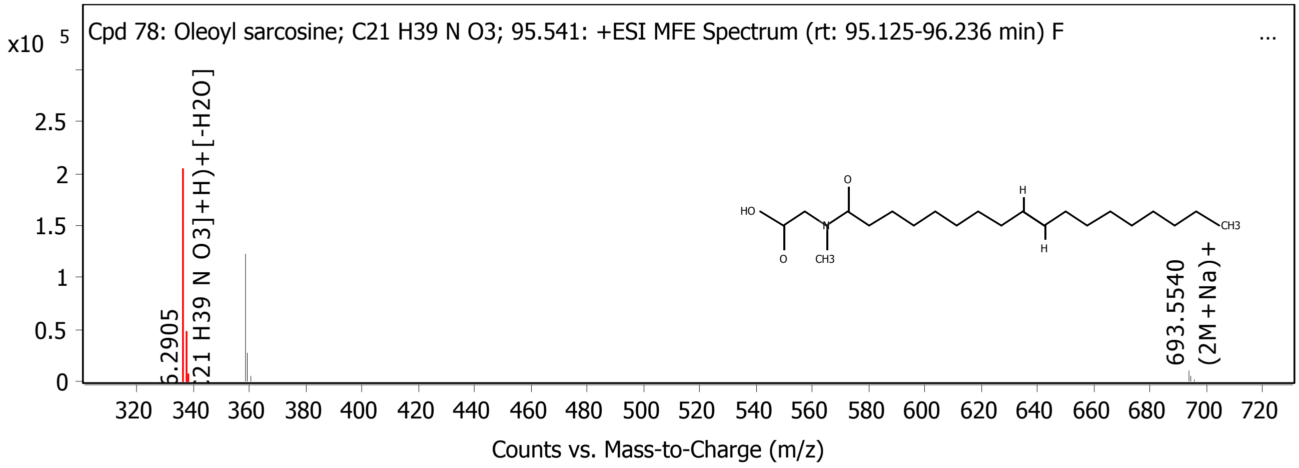

MS Spectrum

Qualitative Compound Identification Report

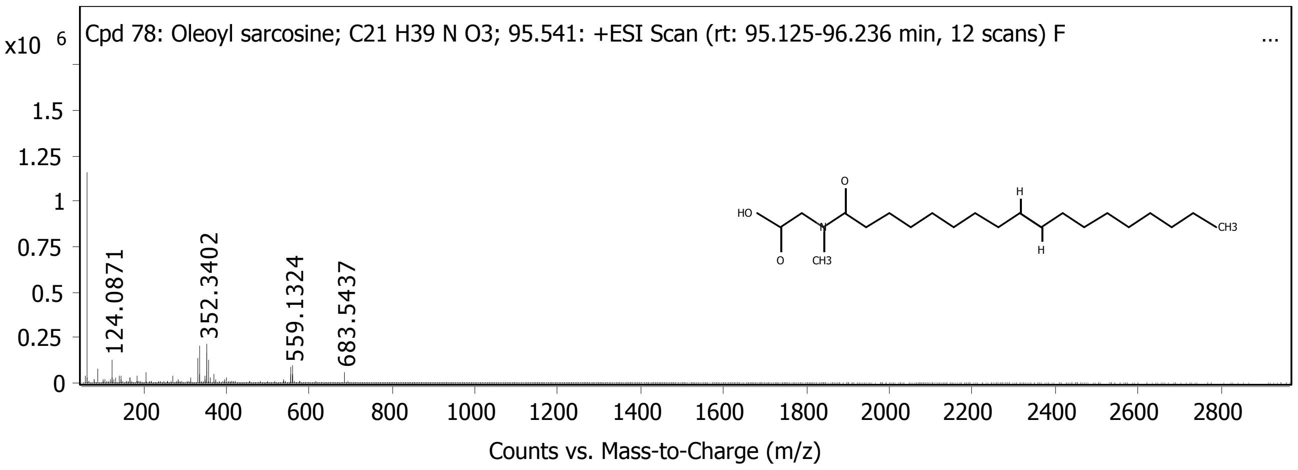

MS Zoomed Spectrum

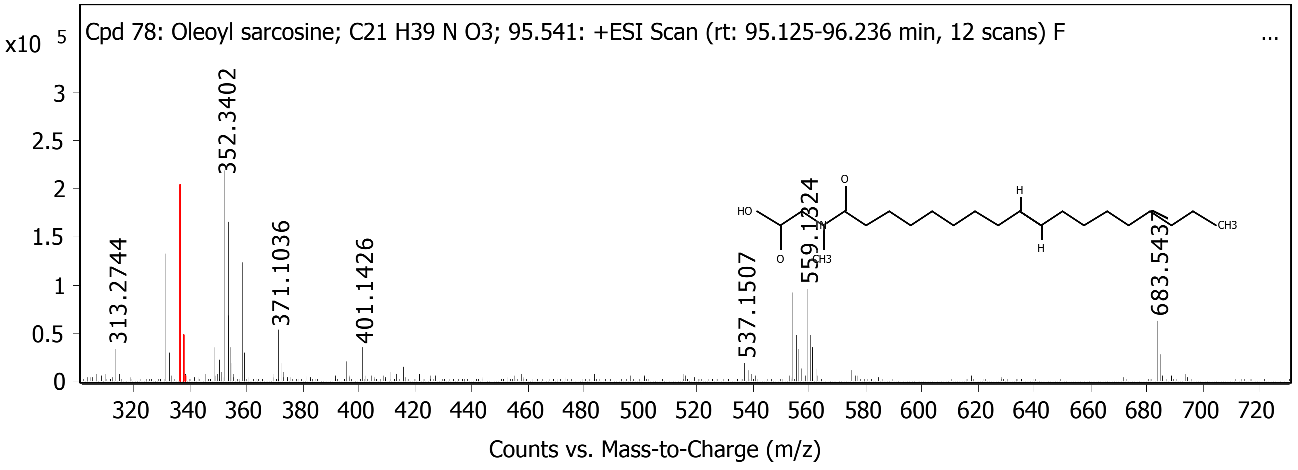

MSMS Spectrum

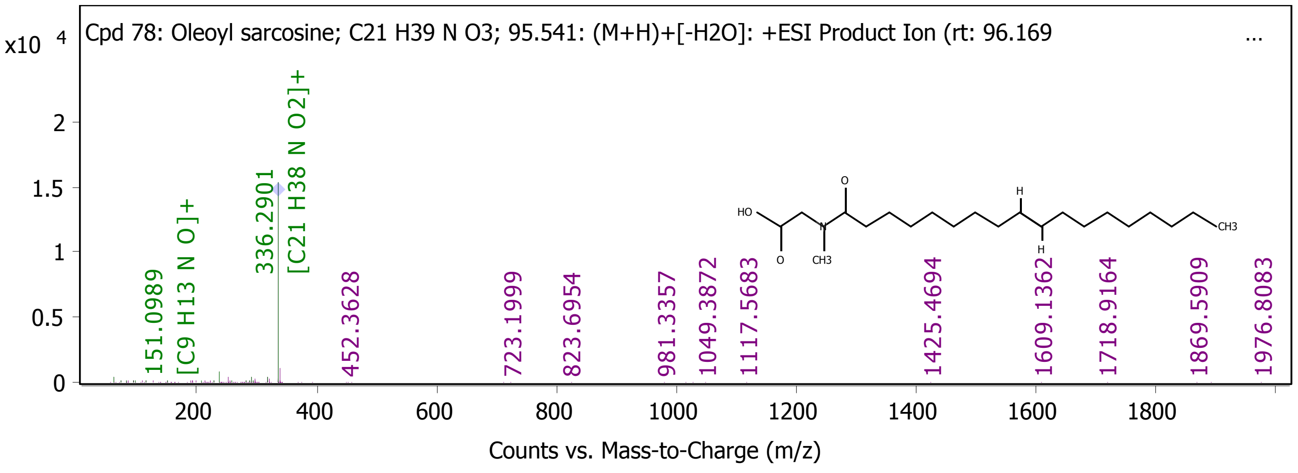

Identification Hit Table

Best Hit	Compound Name	RT	Formula	Notes	Match Score	Mass	Difference	Ion Species
✓	Oleoyl sarcosine	95.541	C21 H39 N O3	Deleted CAS: 57368-03-3	97.23	353.2939	-0.86	(M+H)+[-H2O]
	(8R)-9-((1S,Z)-1-Hydroxy-1-methylhexahydro-2H-quinolizin-3(4H)-ylidene)-5,8-dimethylnonane-3,4-diol	95.541	C21 H39 N O3	Homopumiliotoxins	97.23	353.2939	-0.86	(M+H)+[-H2O]
	N-oleoyl alanine	95.541	C21 H39 N O3		97.23	353.2939	-0.86	(M+H)+[-H2O]
	N-palmitoyl proline	95.541	C21 H39 N O3		97.23	353.2939	-0.86	(M+H)+[-H2O]
	(1S,Z)-3-((2R,E)-6-Hydroxy-2,5-dimethylnon-4-en-1-ylidene)-1-methyloctahydro-2H-quinolizin-1-ol	95.541	C21 H37 N O2	Homopumiliotoxins	97.11	335.2833	-0.86	(M+H)+

Qualitative Compound Identification Report

Identification Hit Table

Best Hit	Compound Name	RT	Formula	Notes	Match Score	Mass	Difference	Ion Species
✓	N-palmitoyl proline	95.541	C21 H39 N O3		97.23	353.2939	-0.86	(M+H)+[-H2O]
	N-oleoyl alanine	95.541	C21 H39 N O3		97.23	353.2939	-0.86	(M+H)+[-H2O]

Identification Hit Table

Best Hit	Compound Name	RT	Formula	Notes	Match Score	Mass	Difference	Ion Species

Compound Label	Name	*m/z*	RT	Algorithm	Mass
Cpd 79: R207910; C32 H31 Br N2 O2; 96.169	**R207910**	559.1324	96.169	Find by Molecular Feature	554.1528

Compound Chromatograms

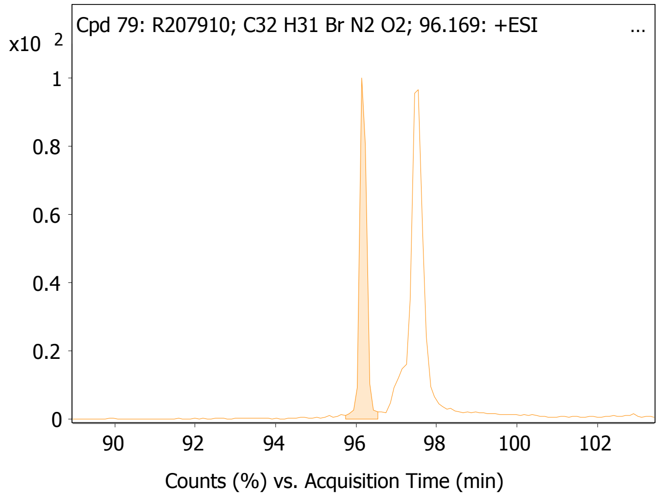

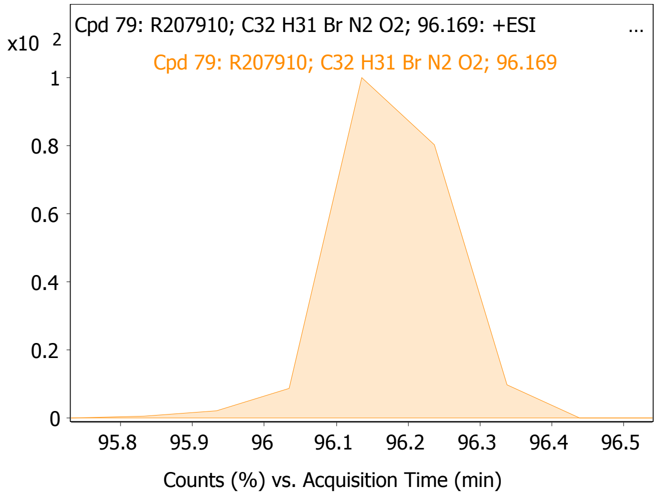

MFE MS Spectrum

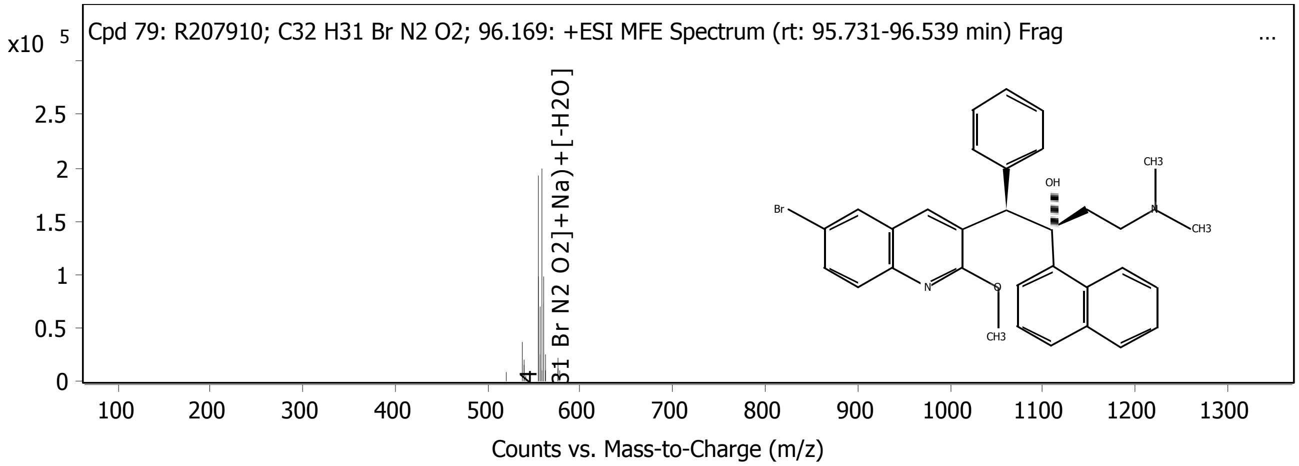

MFE MS Zoomed Spectrum

Qualitative Compound Identification Report

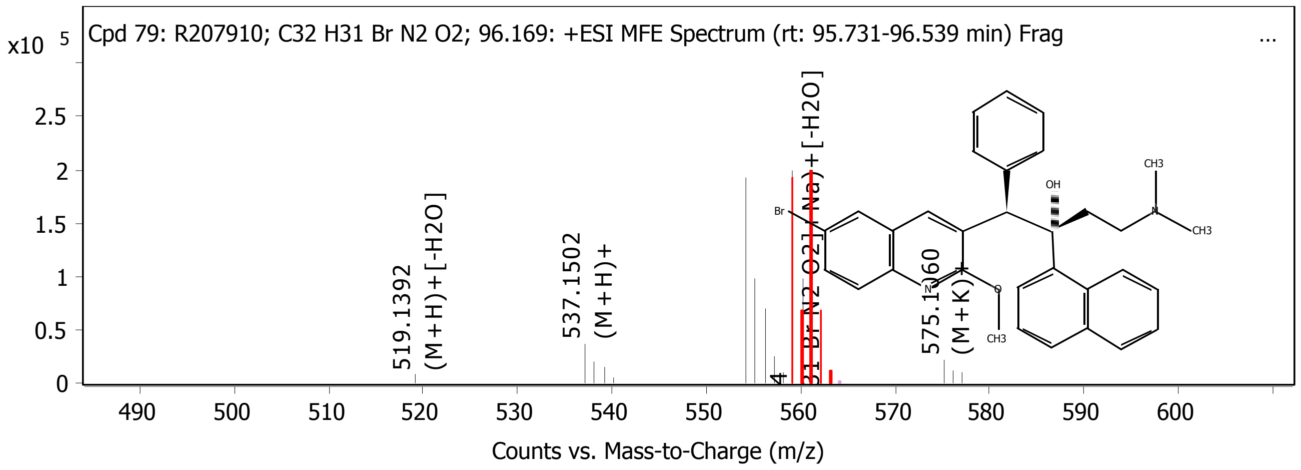

MS Spectrum

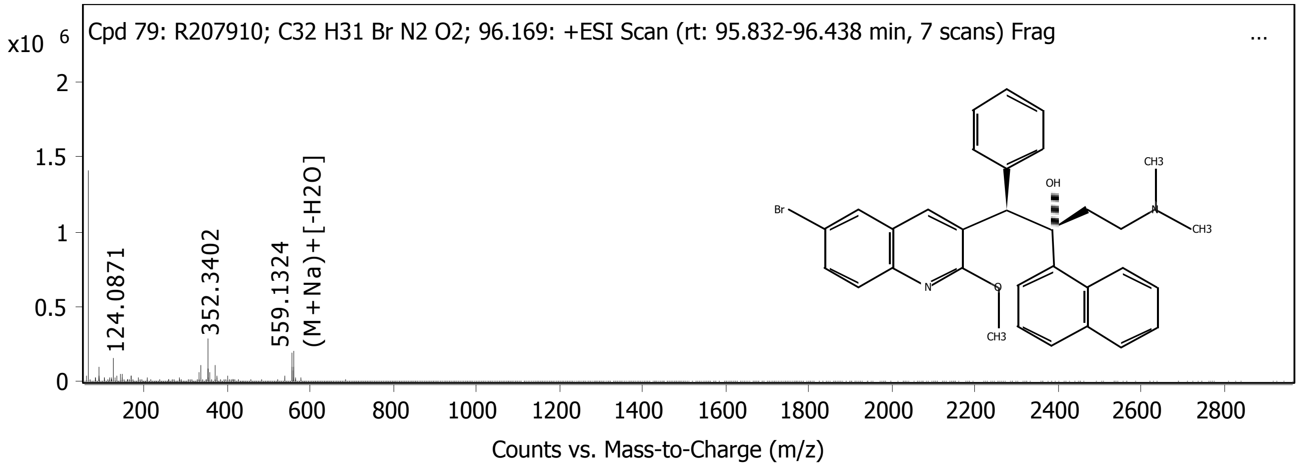

MS Zoomed Spectrum

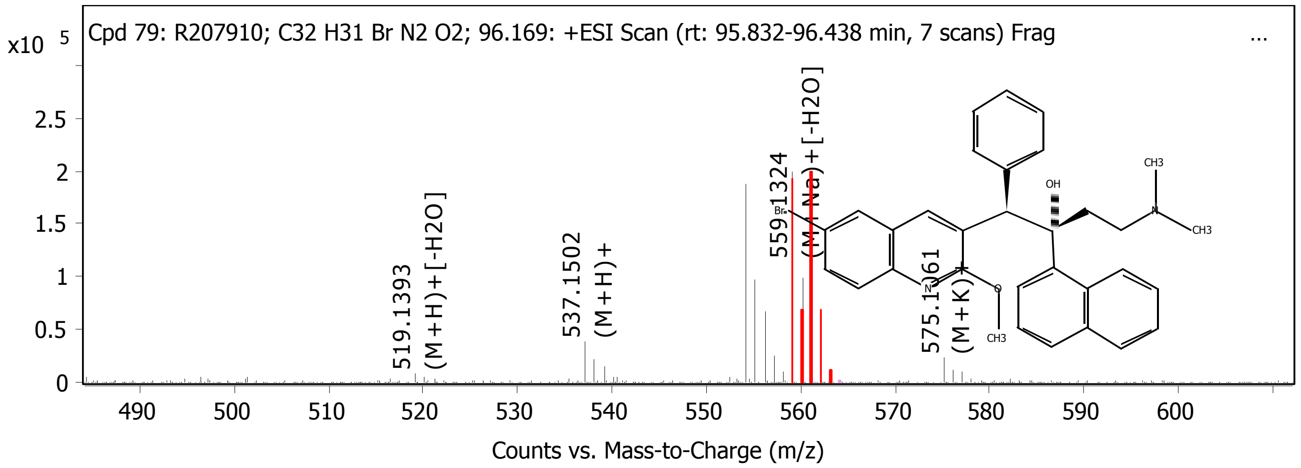

Identification Hit Table

Best Hit	Compound Name	RT	Formula	Notes	Match Score	Mass	Difference	Ion Species
✓	R207910	96.169	C32 H31 Br N2 O2		45.5	554.1528	4.06	(M+Na)+[-H2O]

Identification Hit Table

Best Hit	Compound Name	RT	Formula	Notes	Match Score	Mass	Difference	Ion Species

Identification Hit Table

Best Hit	Compound Name	RT	Formula	Notes	Match Score	Mass	Difference	Ion Species

Compound Label	Name	*m/z*	RT	Algorithm	Mass
Cpd 80: R207910; C32	**R207910**	554.1767	97.512	Find by Molecular	554.1525

Qualitative Compound Identification Report

H31 Br N2 O2; 97.512				Feature	

Compound Chromatograms

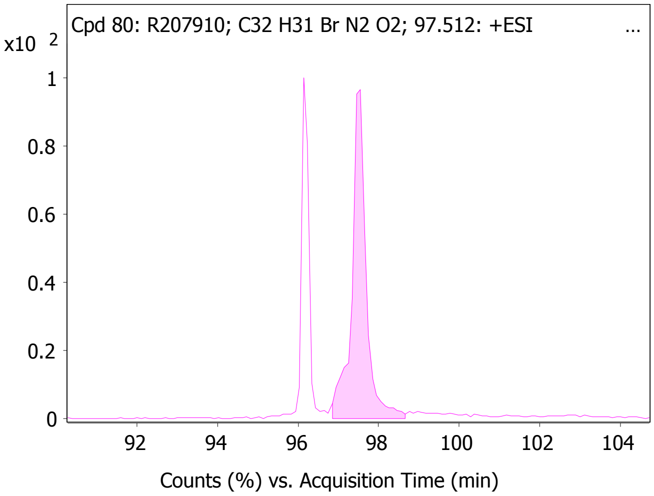

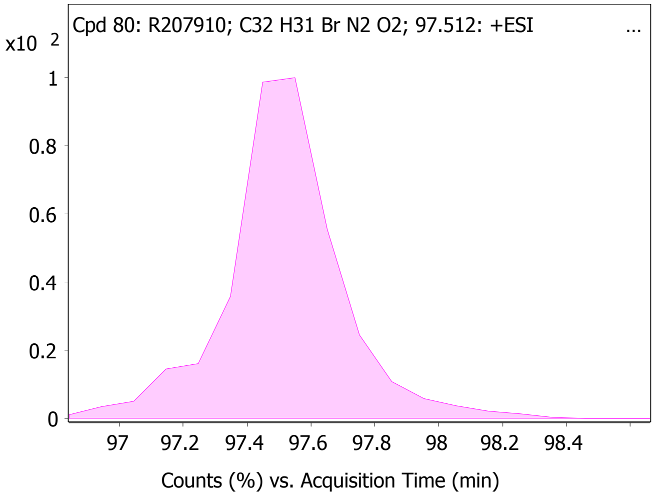

MFE MS Spectrum

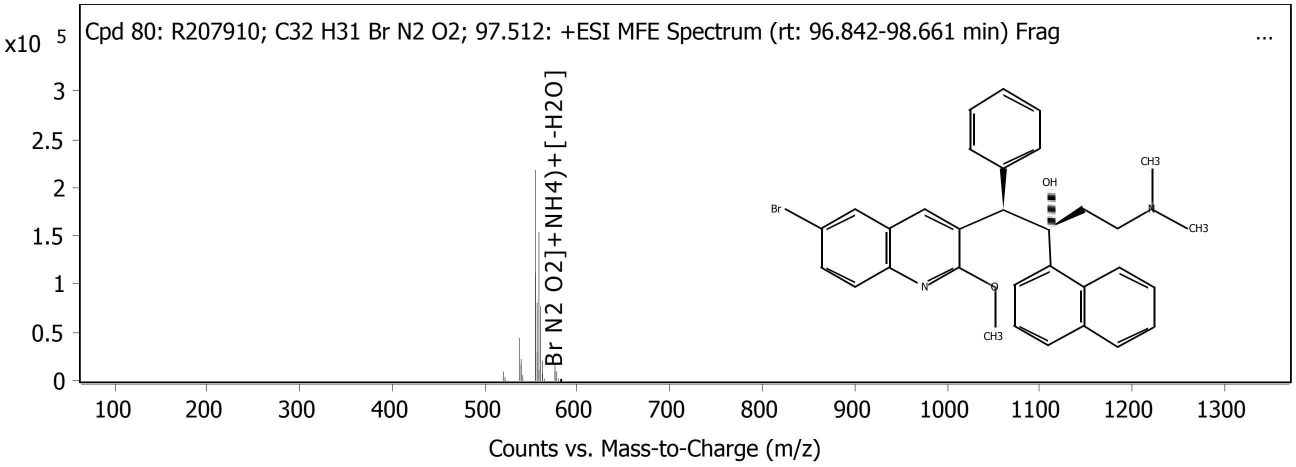

MFE MS Zoomed Spectrum

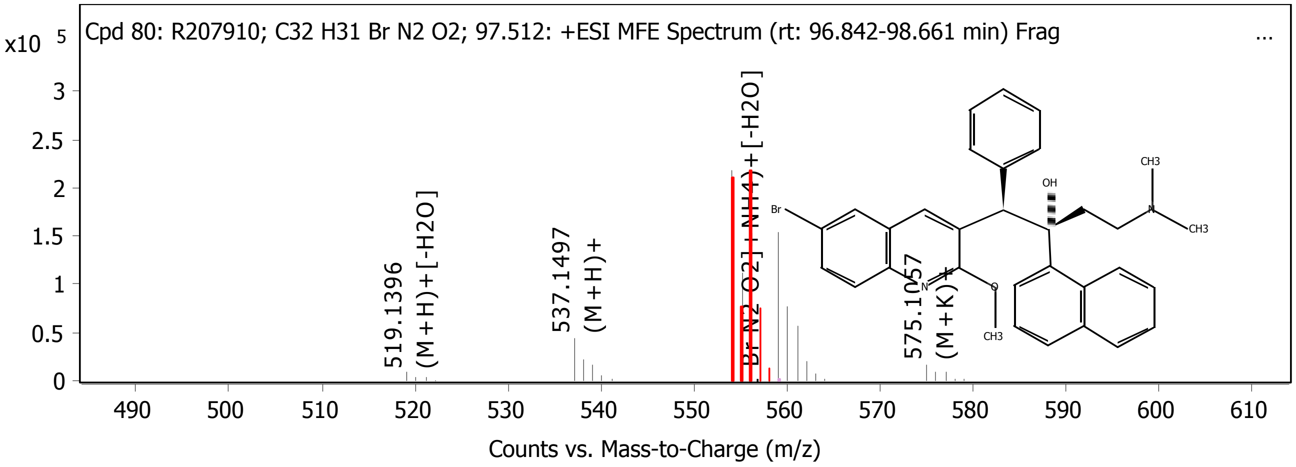

MS Spectrum

Qualitative Compound Identification Report

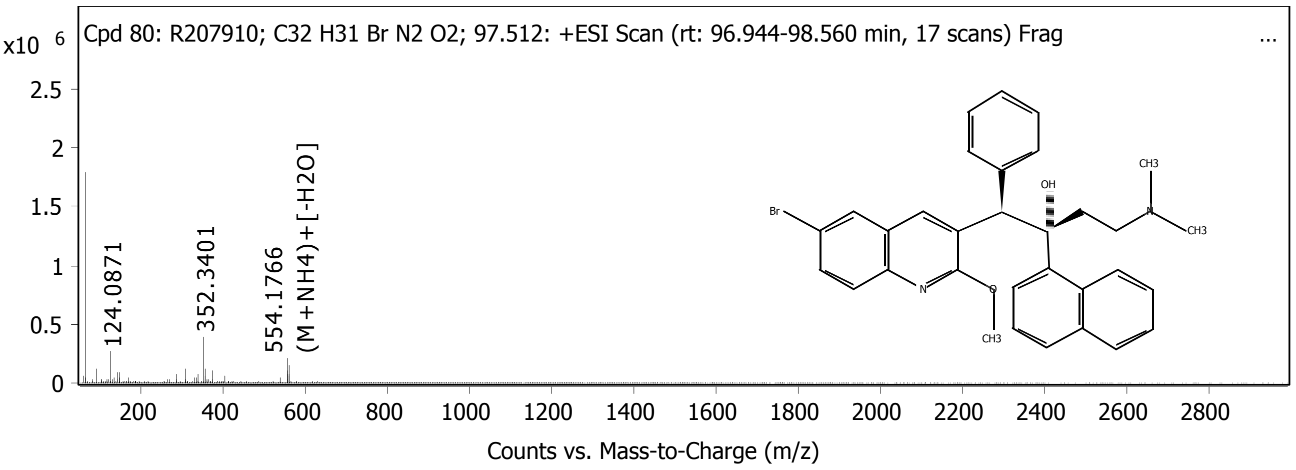

MS Zoomed Spectrum

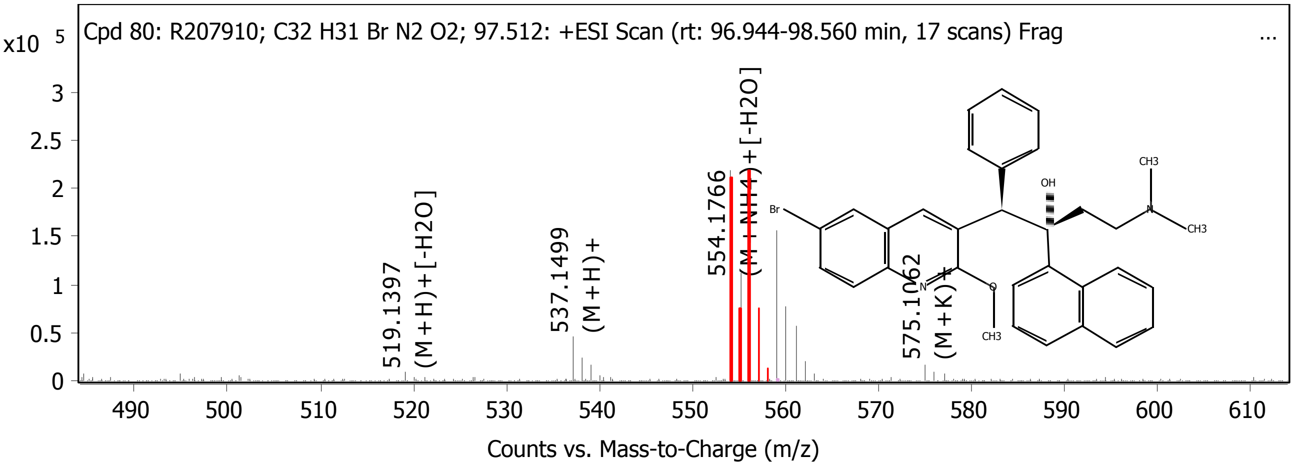

Identification Hit Table

Best Hit	Compound Name	RT	Formula	Notes	Match Score	Mass	Difference	Ion Species
✓	R207910	97.512	C32 H31 Br N2 O2		42.1	554.1525	4.44	(M+NH4)+[-H2O]

Identification Hit Table

Best Hit	Compound Name	RT	Formula	Notes	Match Score	Mass	Difference	Ion Species

Identification Hit Table

Best Hit	Compound Name	RT	Formula	Notes	Match Score	Mass	Difference	Ion Species

Compound Label	Name	*m/z*	RT	Algorithm	Mass
Cpd 81: Isorhamnetin 3-O-[b-D-xylopyranosyl-(1->6)-b-D-glucopyranoside]; C27 H30 O16; 100.855	**Isorhamnetin 3-O-[b-D-xylopyranosyl-(1->6)-b-D-glucopyranoside]**	628.1955	100.855	Find by Molecular Feature	610.1585

Compound Chromatograms

Qualitative Compound Identification Report

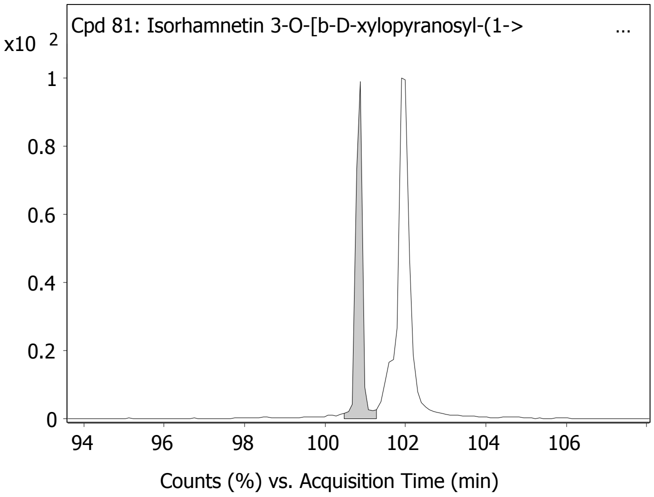

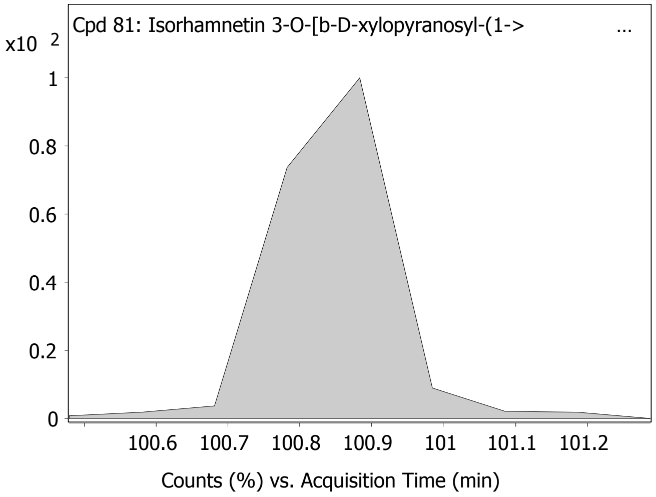

MFE MS Spectrum

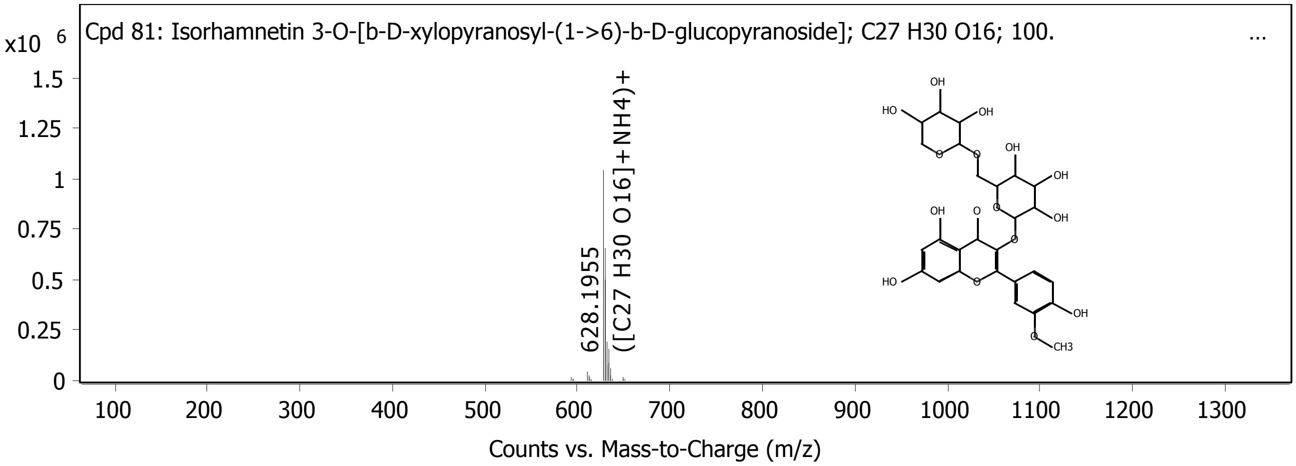

MFE MS Zoomed Spectrum

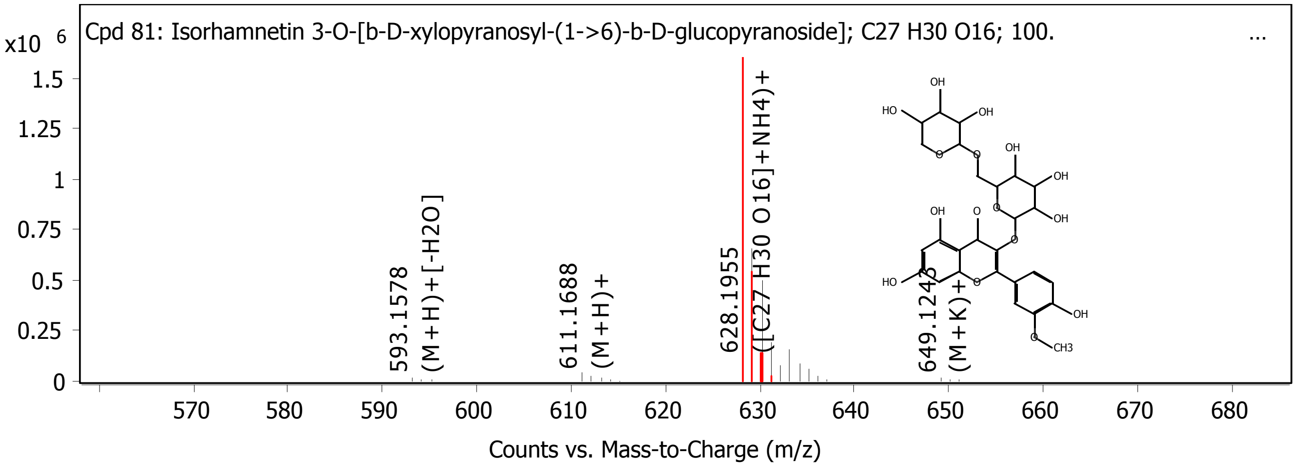

MS Spectrum

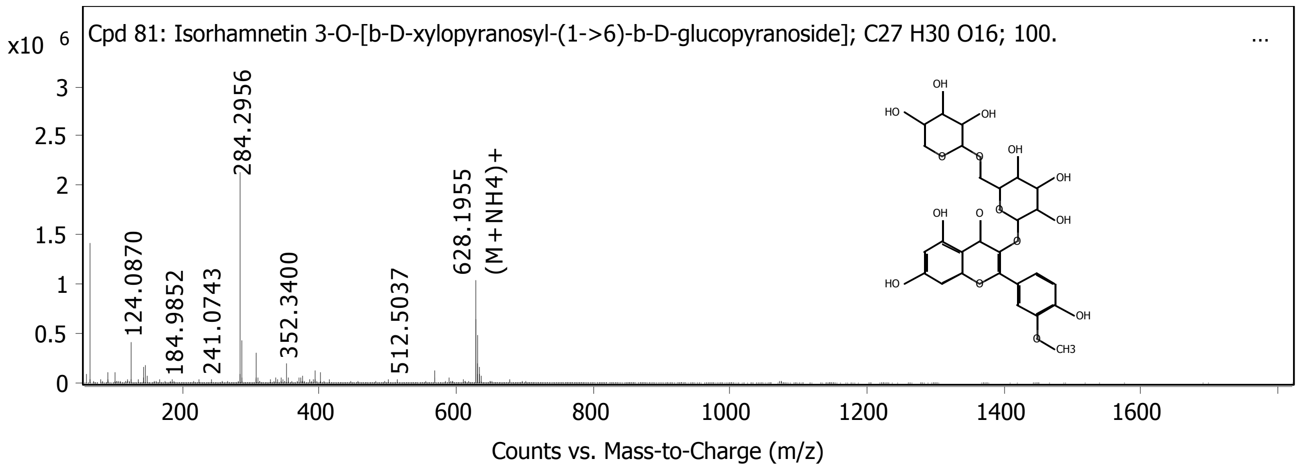

MS Zoomed Spectrum

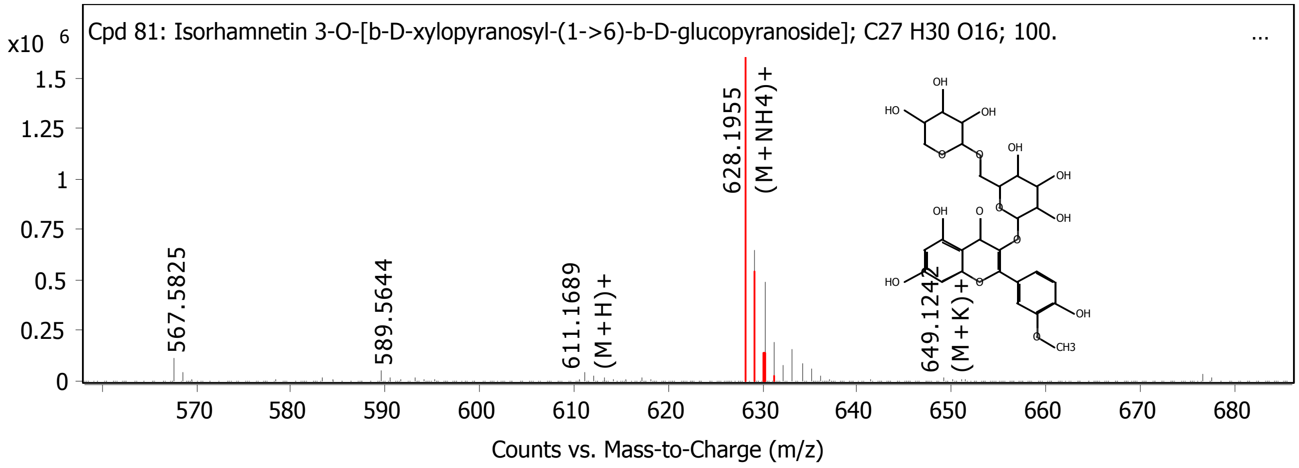

Identification Hit Table

Best Hit	Compound Name	RT	Formula	Notes	Match Score	Mass	Difference	Ion Species
✓	Isorhamnetin 3-O-[b-D-xylopyranosyl-(1->6)-b-D-glucopyranoside]	100.855	C27 H30 O16		33.56	610.1585	-5.09	(M+NH4)+
	3,7,2',3',4'-Pentahydroxyflavone 3-neohesperidoside	100.855	C27 H30 O16		33.56	610.1585	-5.09	(M+NH4)+
	Robinetin 3-rutinoside	100.855	C27 H30 O16		33.56	610.1585	-5.09	(M+NH4)+
	Luteolin 7,4'-diglucoside	100.855	C27 H30 O16		33.56	610.1585	-5.09	(M+NH4)+
	Luteolin 7-galactoside-4'-glucoside	100.855	C27 H30 O16		33.56	610.1585	-5.09	(M+NH4)+
	Luteolin 3',4'-diglucoside	100.855	C27 H30 O16		33.56	610.1585	-5.09	(M+NH4)+
	Luteolin 7-galactosyl-(1->6)-galactoside	100.855	C27 H30 O16		33.56	610.1585	-5.09	(M+NH4)+
	6-Hydroxyluteolin 6-glucoside-3'-rhamnoside	100.855	C27 H30 O16		33.56	610.1585	-5.09	(M+NH4)+
	6-Hydroxyluteolin 7-rutinoside	100.855	C27 H30 O16		33.56	610.1585	-5.09	(M+NH4)+
	Isoscutellarein 7-allosyl-(1->2)-glucoside	100.855	C27 H30 O16		33.56	610.1585	-5.09	(M+NH4)+

Identification Hit Table

Best Hit	Compound Name	RT	Formula	Notes	Match Score	Mass	Difference	Ion Species
✓	Herbacetin 3-rhamnoside-8-glucoside	100.855	C27 H30 O16		33.56	610.1585	-5.09	(M+NH4)+
	Quercetin 3-rhamnosyl-(1->2)-galactoside	100.855	C27 H30 O16		33.56	610.1585	-5.09	(M+NH4)+
	Quercetin 3-galactoside-7-rhamnoside	100.855	C27 H30 O16		33.56	610.1585	-5.09	(M+NH4)+
	Quercetin 7-rutinoside	100.855	C27 H30 O16		33.56	610.1585	-5.09	(M+NH4)+
	Quercetin 3-rhamnoside-7-glucoside	100.855	C27 H30 O16		33.56	610.1585	-5.09	(M+NH4)+
	Quercetin 3-glucosyl-(1->4)-rhamnoside	100.855	C27 H30 O16		33.56	610.1585	-5.09	(M+NH4)+
	Quercetin 3-glucosyl-(1->2)-rhamnoside	100.855	C27 H30 O16		33.56	610.1585	-5.09	(M+NH4)+
	Quercetin 3-galactosyl-(1->4)-rhamnoside	100.855	C27 H30 O16		33.56	610.1585	-5.09	(M+NH4)+
	Quercetin 3-glucoside-7-rhamnoside	100.855	C27 H30 O16		33.56	610.1585	-5.09	(M+NH4)+
	Quercetin 3-neohesperidoside	100.855	C27 H30 O16		33.56	610.1585	-5.09	(M+NH4)+

Identification Hit Table

Best Hit	Compound Name	RT	Formula	Notes	Match Score	Mass	Difference	Ion Species

Qualitative Compound Identification Report

Compound Label	Name	*m/z*	RT	Algorithm	Mass
Cpd 82: Stearamide; C18 H37 N O; 100.937	**Stearamide**	284.2957	100.937	Find by Molecular Feature	283.2883

Compound Chromatograms

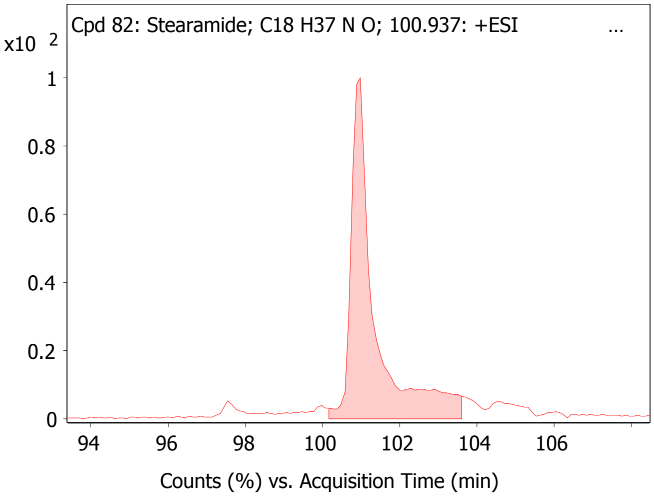

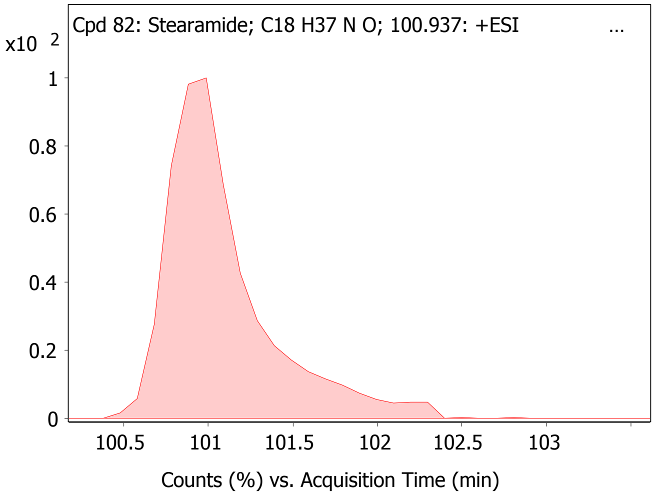

MFE MS Spectrum

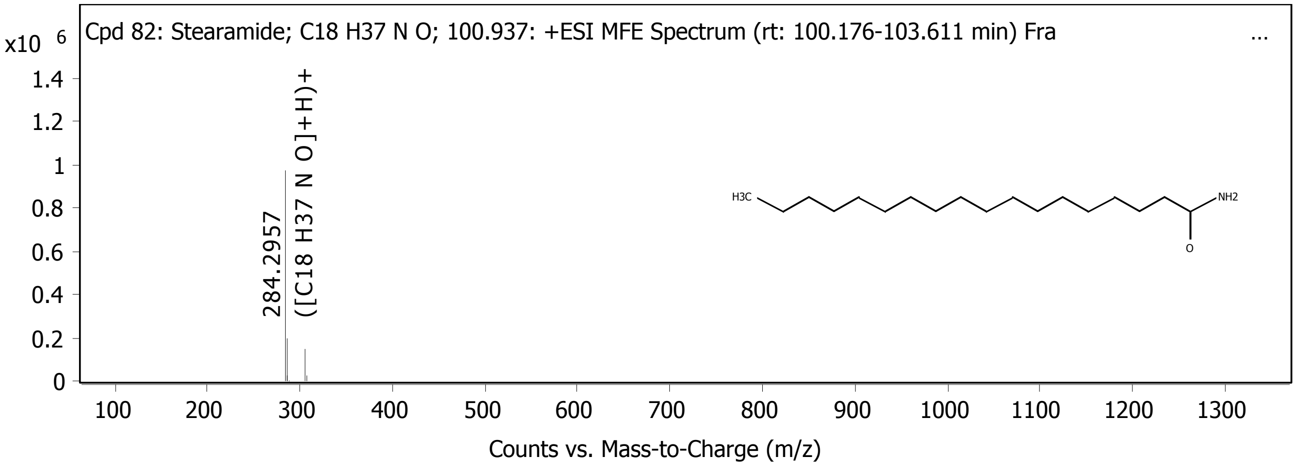

MFE MS Zoomed Spectrum

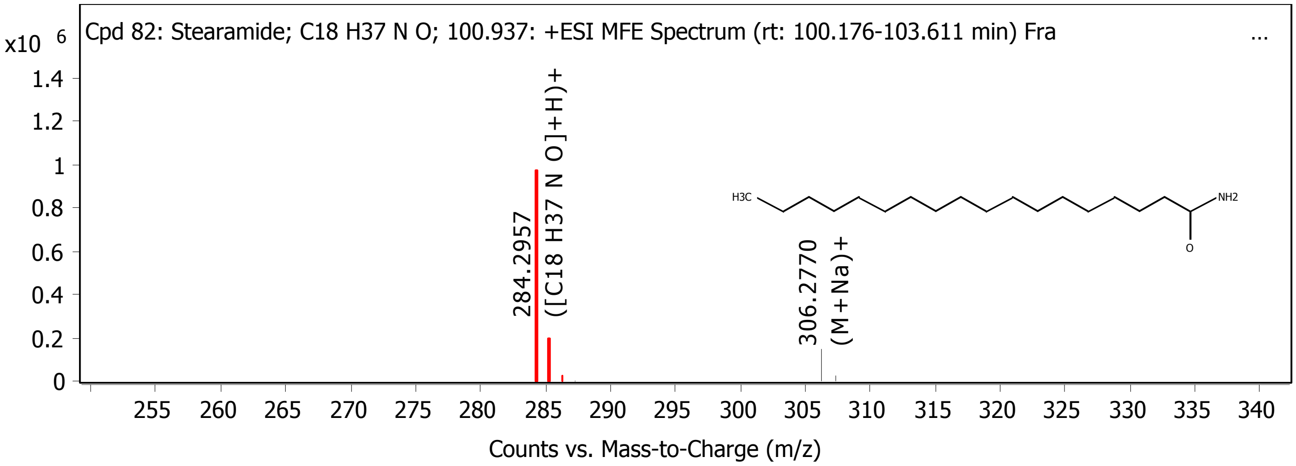

MS Spectrum

Qualitative Compound Identification Report

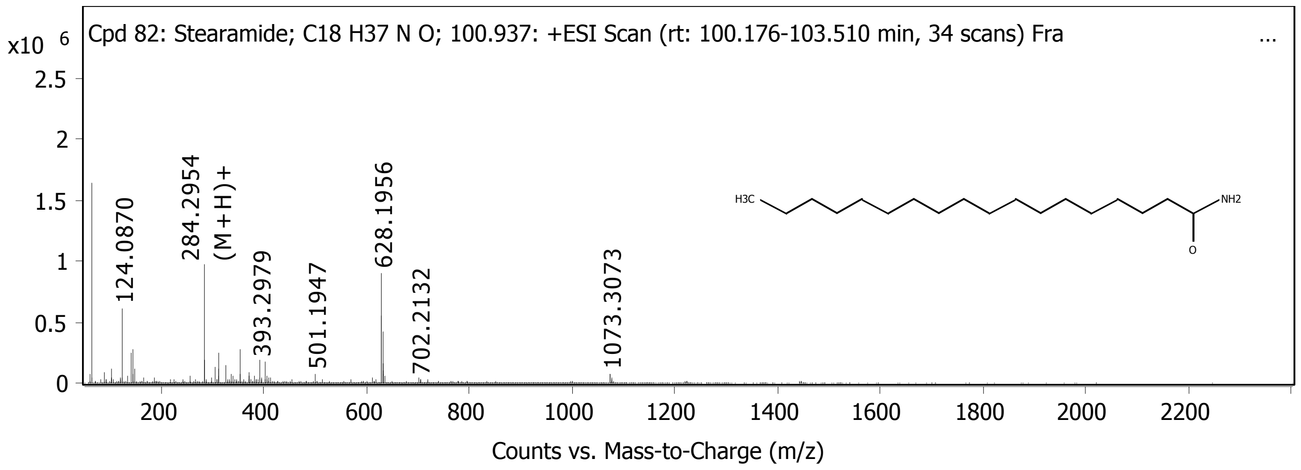

MS Zoomed Spectrum

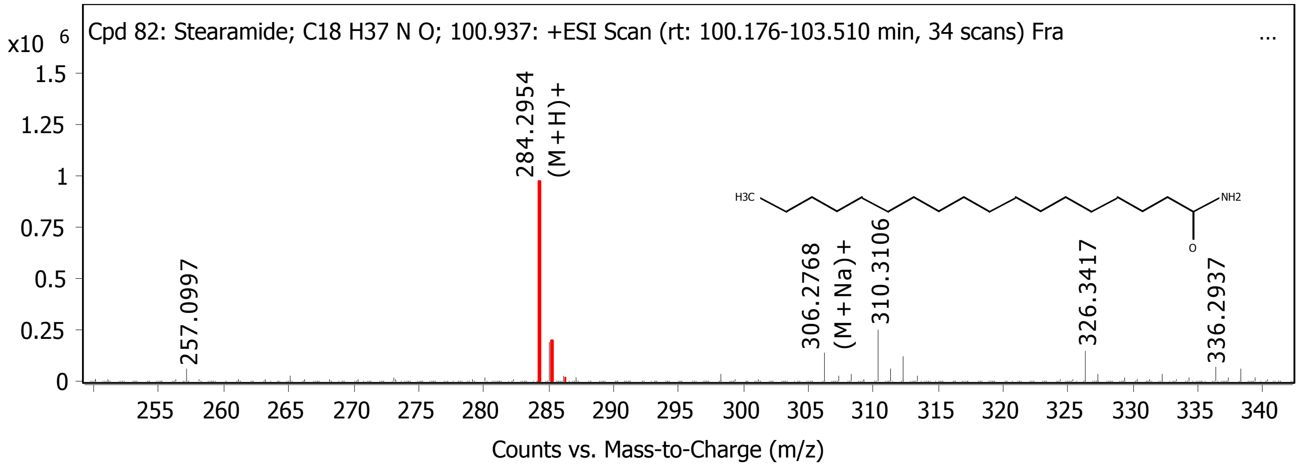

Library Spectrum

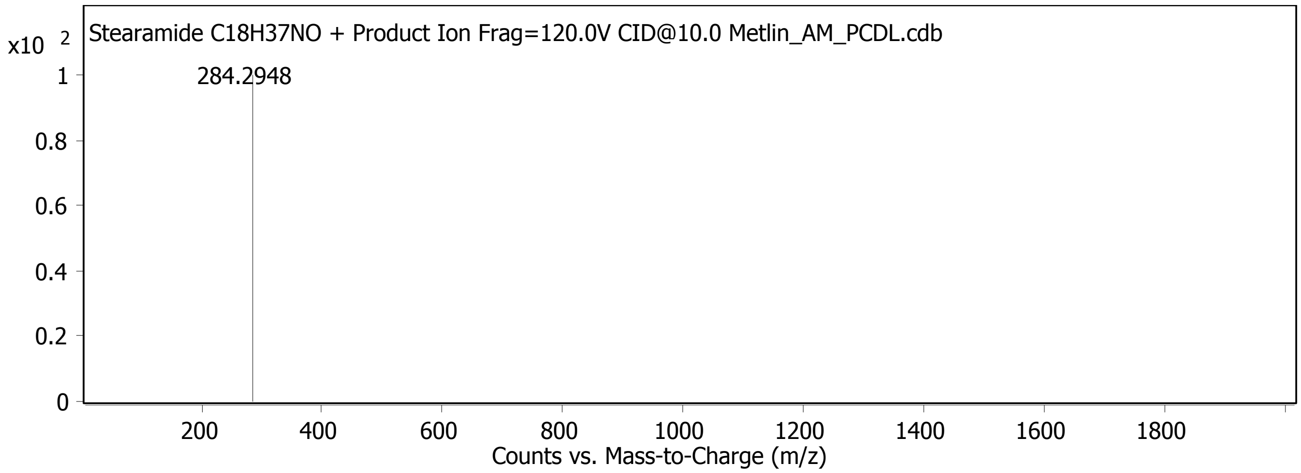

Difference Spectrum

Qualitative Compound Identification Report

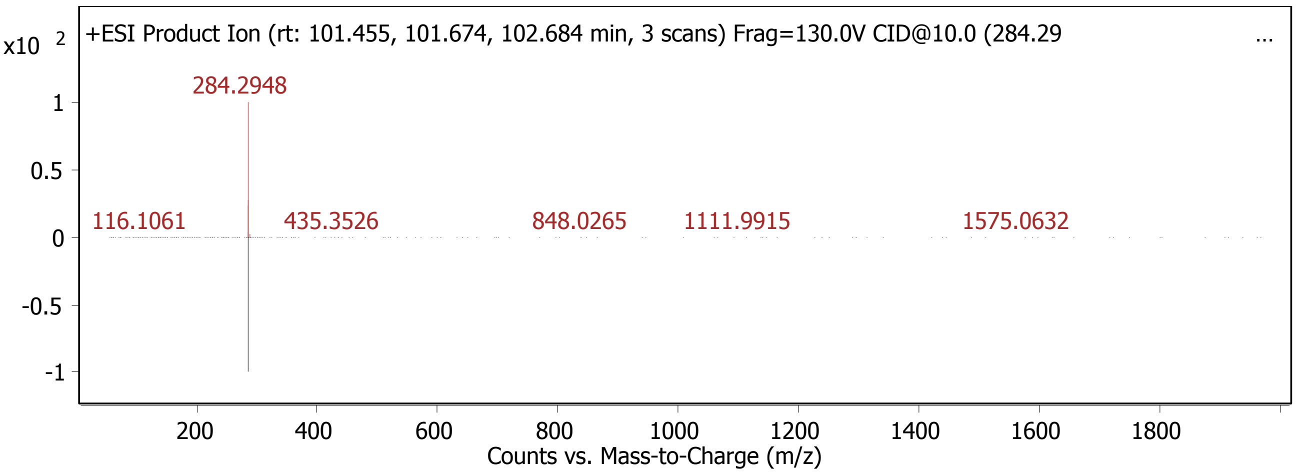

MSMS Spectrum

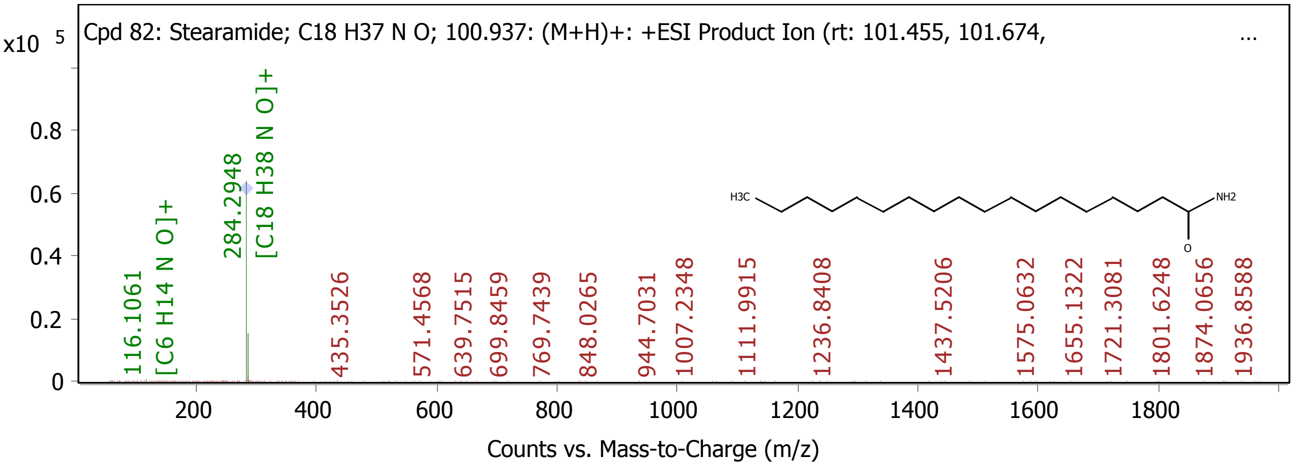

Identification Hit Table

Best Hit	Compound Name	RT	Formula	Notes	Match Score	Mass	Difference	Ion Species
✓	Enigmol	100.937	C18 H39 N O2		96.96	301.2989	-0.82	(M+H)+[-H2O]
	Safingol (L-threo-sphinganine)	100.937	C18 H39 N O2		96.96	301.2989	-0.82	(M+H)+[-H2O]
	Sphinganine	100.937	C18 H39 N O2		96.96	301.2989	-0.82	(M+H)+[-H2O]
	Stearamide	100.937	C18 H37 N O	Octadecanamide Stearamide Octadecamide Octadecylamide Stearoylamide Stearoylamine Stearylamide Stearic amide Kemamide S Adogen 42 PubChem CID: 31292	96.84	283.2883	-0.82	(M+H)+

Identification Hit Table

Best Hit	Compound Name	RT	Formula	Notes	Match Score	Mass	Difference	Ion Species
✓	Sphinganine	100.937	C18 H39 N O2		96.96	301.2989	-0.82	(M+H)+[-H2O]
	Safingol (L-threo-sphinganine)	100.937	C18 H39 N O2		96.96	301.2989	-0.82	(M+H)+[-H2O]
	Enigmol	100.937	C18 H39 N O2		96.96	301.2989	-0.82	(M+H)+[-H2O]
	Stearamide	100.937	C18 H37 N O	Octadecanamide Stearamide Octadecamide Octadecylamide Stearoylamide Stearoylamine Stearylamide Stearic amide Kemamide S Adogen 42 PubChem CID: 31292	96.84	283.2883	-0.82	(M+H)+

Identification Hit Table

Best Hit	Compound Name	RT	Formula	Notes	Match Score	Mass	Difference	Ion Species

Compound Label	Name	*m/z*	RT	Algorithm	Mass
Cpd 83: Isorhamnetin 3-O-[b-D-xylopyranosyl-(1->6)-b-D-glucopyranoside]; C27 H30 O16; 101.949	**Isorhamnetin 3-O-[b-D-xylopyranosyl-(1->6)-b-D-glucopyranoside]**	628.1958	101.949	Find by Molecular Feature	610.1589

Compound Chromatograms

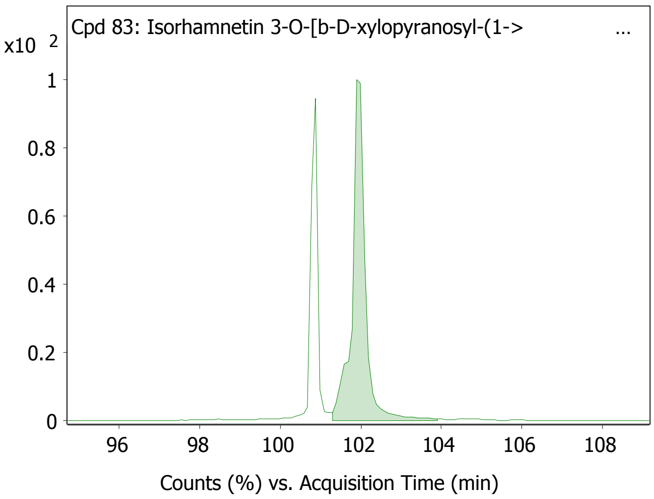

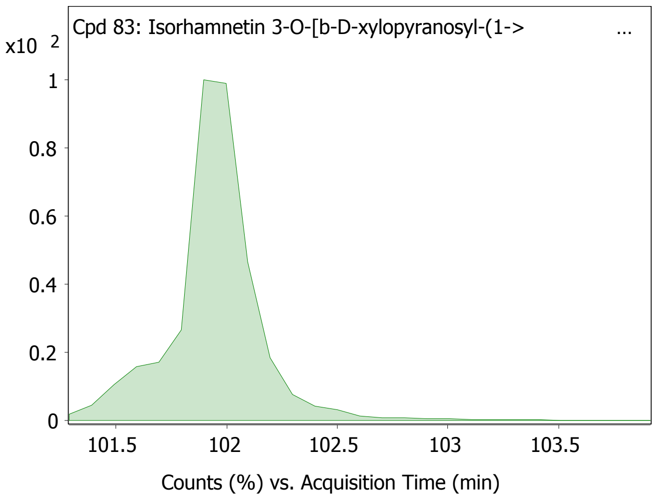

MFE MS Spectrum

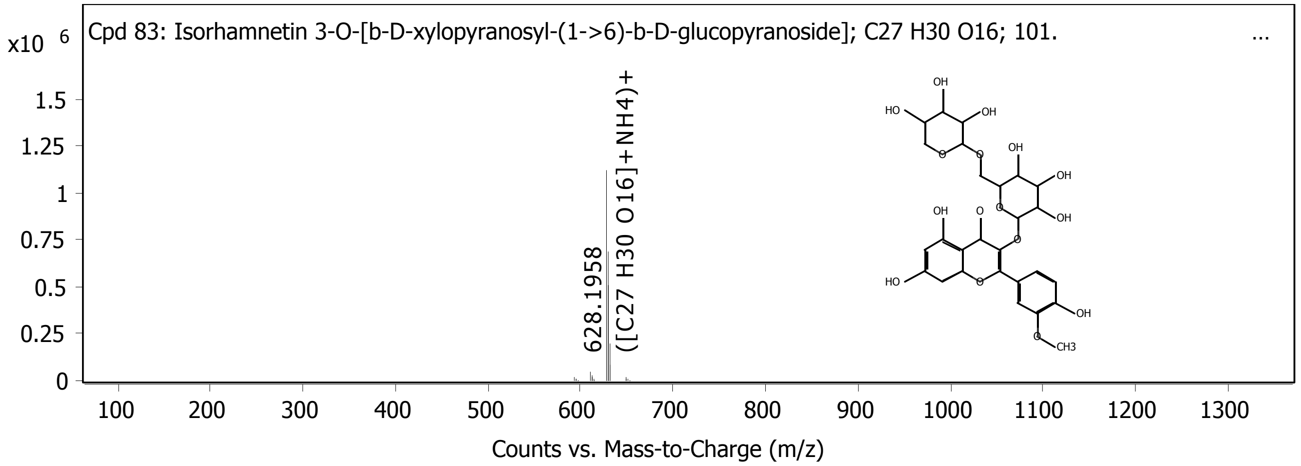

MFE MS Zoomed Spectrum

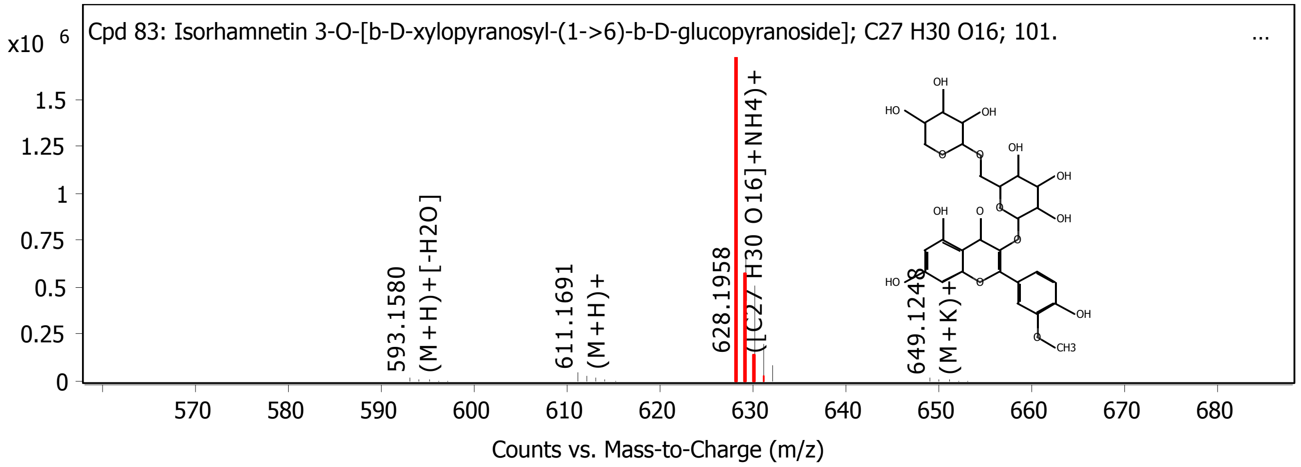

MS Spectrum

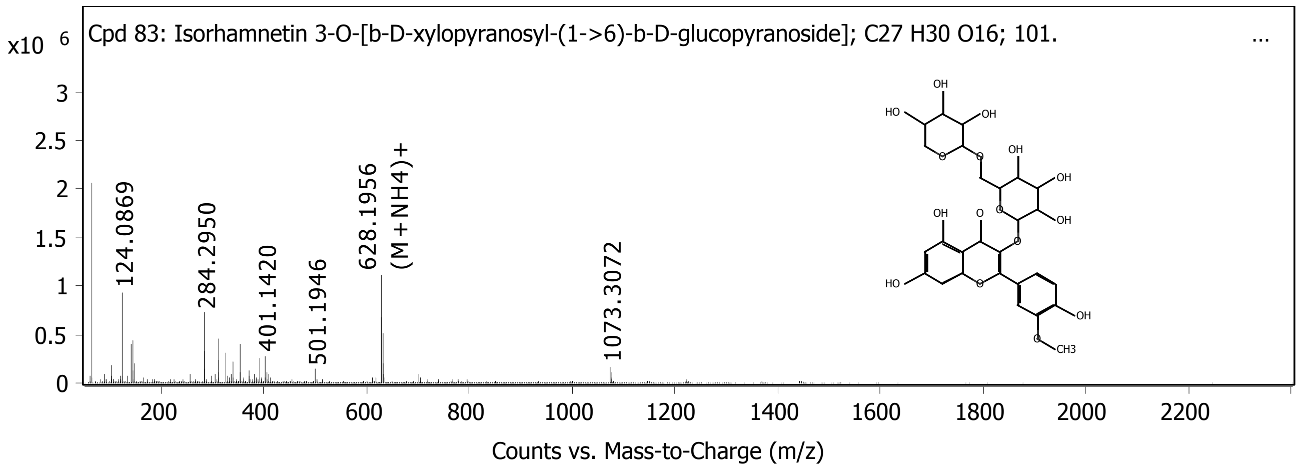

MS Zoomed Spectrum

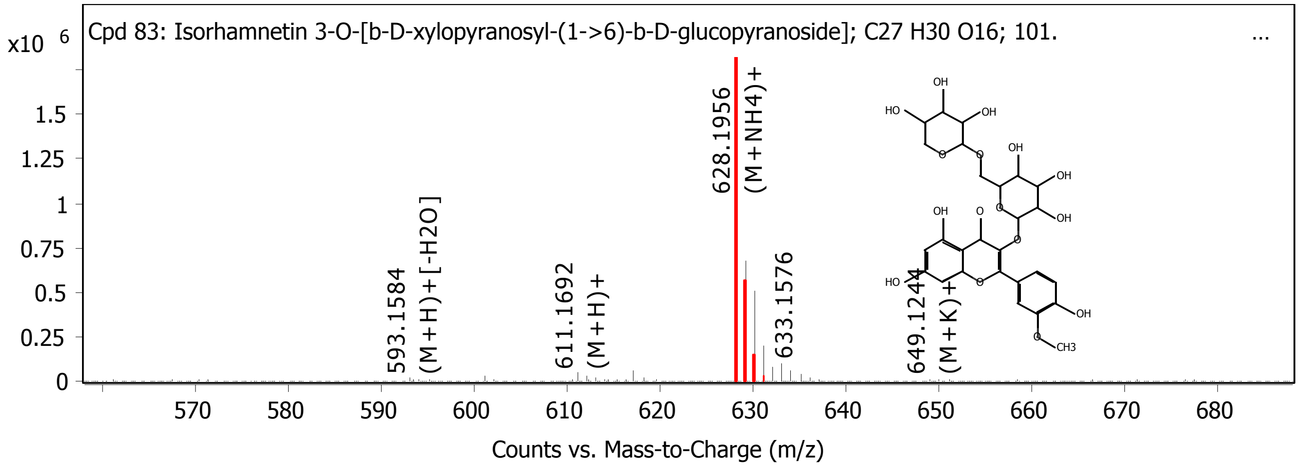

MSMS Spectrum

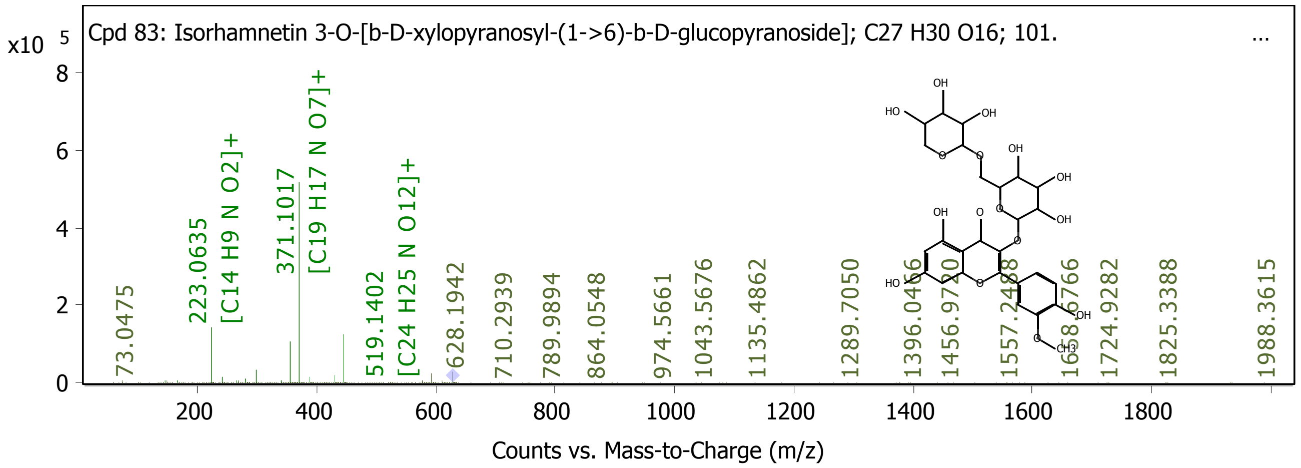

Identification Hit Table

Best Hit	Compound Name	RT	Formula	Notes	Match Score	Mass	Difference	Ion Species
✓	Isorhamnetin 3-O-[b-D-xylopyranosyl-(1->6)-b-D-glucopyranoside]	101.949	C27 H30 O16		31.08	610.1589	-5.53	(M+NH4)+
	3,7,2',3',4'-Pentahydroxyflavone 3-neohesperidoside	101.949	C27 H30 O16		31.08	610.1589	-5.53	(M+NH4)+
	Robinetin 3-rutinoside	101.949	C27 H30 O16		31.08	610.1589	-5.53	(M+NH4)+
	Luteolin 7,4'-diglucoside	101.949	C27 H30 O16		31.08	610.1589	-5.53	(M+NH4)+
	Luteolin 7-galactoside-4'-glucoside	101.949	C27 H30 O16		31.08	610.1589	-5.53	(M+NH4)+
	Luteolin 3',4'-diglucoside	101.949	C27 H30 O16		31.08	610.1589	-5.53	(M+NH4)+
	Luteolin 7-galactosyl-(1->6)-galactoside	101.949	C27 H30 O16		31.08	610.1589	-5.53	(M+NH4)+

	6-Hydroxyluteolin 6-glucoside-3'-rhamnoside	101.949	C27 H30 O16		31.08	610.1589	-5.53	(M+NH4)+
	6-Hydroxyluteolin 7-rutinoside	101.949	C27 H30 O16		31.08	610.1589	-5.53	(M+NH4)+
	Isoscutellarein 7-allosyl-(1->2)-glucoside	101.949	C27 H30 O16		31.08	610.1589	-5.53	(M+NH4)+

Identification Hit Table

Best Hit	Compound Name	RT	Formula	Notes	Match Score	Mass	Difference	Ion Species
✓	Herbacetin 3-rhamnoside-8-glucoside	101.949	C27 H30 O16		31.08	610.1589	-5.53	(M+NH4)+
	Quercetin 3-rhamnosyl-(1->2)-galactoside	101.949	C27 H30 O16		31.08	610.1589	-5.53	(M+NH4)+
	Quercetin 3-galactoside-7-rhamnoside	101.949	C27 H30 O16		31.08	610.1589	-5.53	(M+NH4)+
	Quercetin 7-rutinoside	101.949	C27 H30 O16		31.08	610.1589	-5.53	(M+NH4)+
	Quercetin 3-rhamnoside-7-glucoside	101.949	C27 H30 O16		31.08	610.1589	-5.53	(M+NH4)+
	Quercetin 3-glucosyl-(1->4)-rhamnoside	101.949	C27 H30 O16		31.08	610.1589	-5.53	(M+NH4)+
	Quercetin 3-glucosyl-(1->2)-rhamnoside	101.949	C27 H30 O16		31.08	610.1589	-5.53	(M+NH4)+
	Quercetin 3-galactosyl-(1->4)-rhamnoside	101.949	C27 H30 O16		31.08	610.1589	-5.53	(M+NH4)+
	Quercetin 3-glucoside-7-rhamnoside	101.949	C27 H30 O16		31.08	610.1589	-5.53	(M+NH4)+
	Quercetin 3-neohesperidoside	101.949	C27 H30 O16		31.08	610.1589	-5.53	(M+NH4)+

Identification Hit Table

Best Hit	Compound Name	RT	Formula	Notes	Match Score	Mass	Difference	Ion Species

Compound Label	Name	*m/z*	RT	Algorithm	Mass
Cpd 84: D-erythro-Sphingosine C-20; C20 H41 N O2; 103.030	**D-erythro-Sphingosine C-20**	310.3108	103.03	Find by Molecular Feature	327.314

Compound Chromatograms

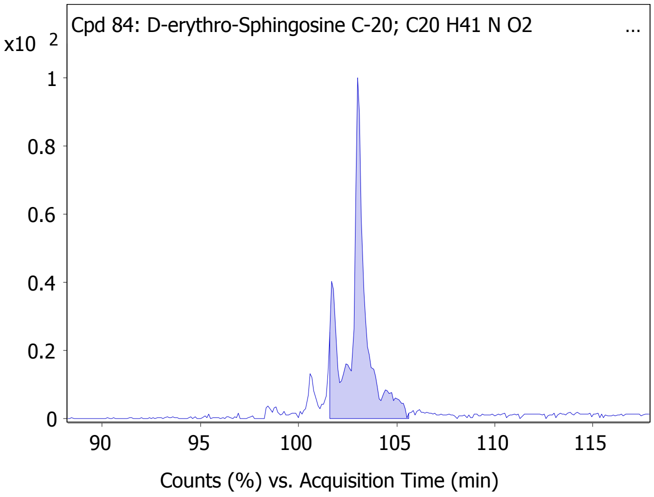

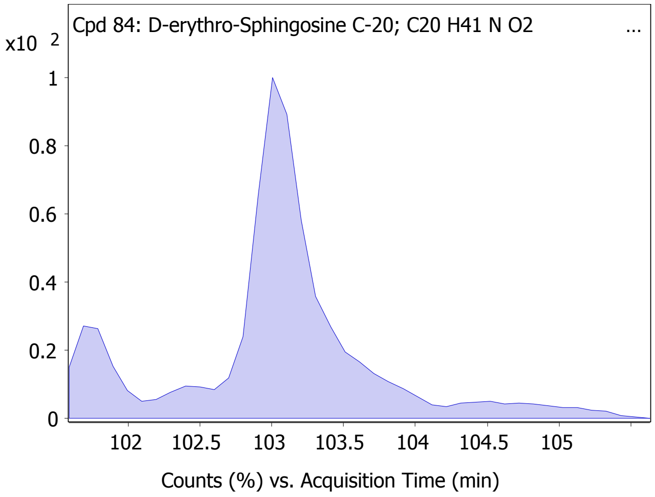

MFE MS Spectrum

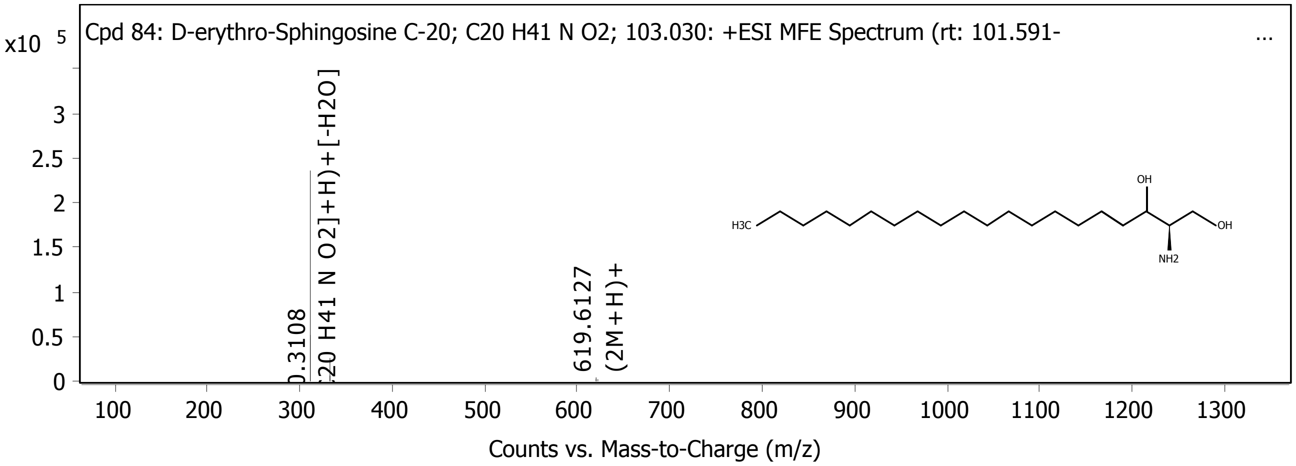

MFE MS Zoomed Spectrum

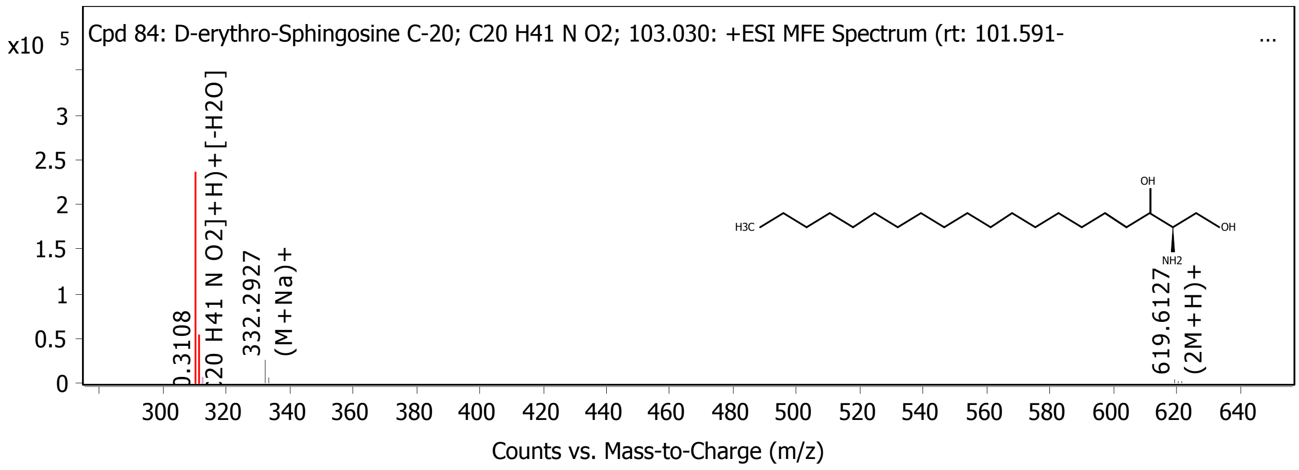

MS Spectrum

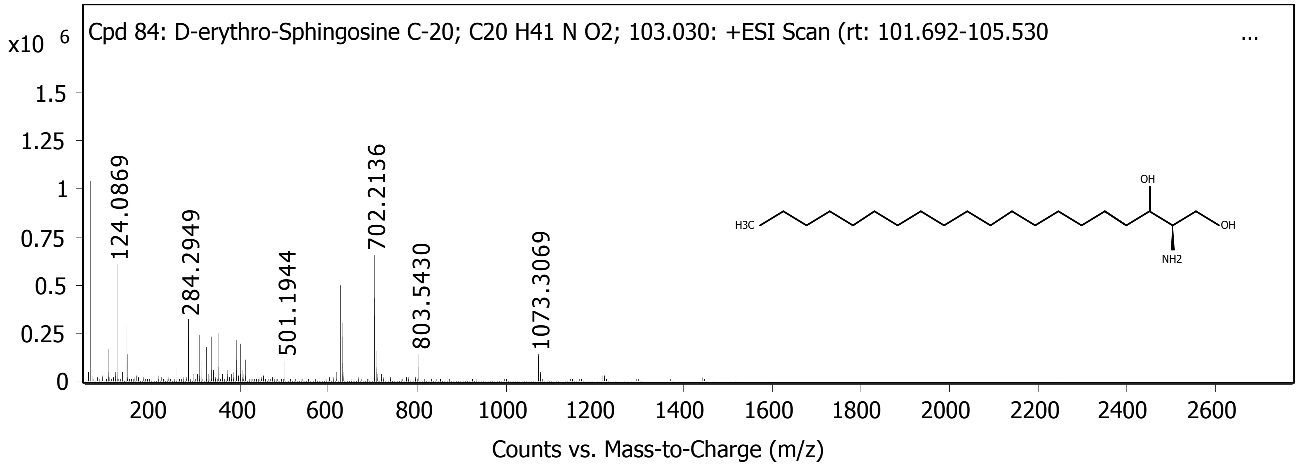

MS Zoomed Spectrum

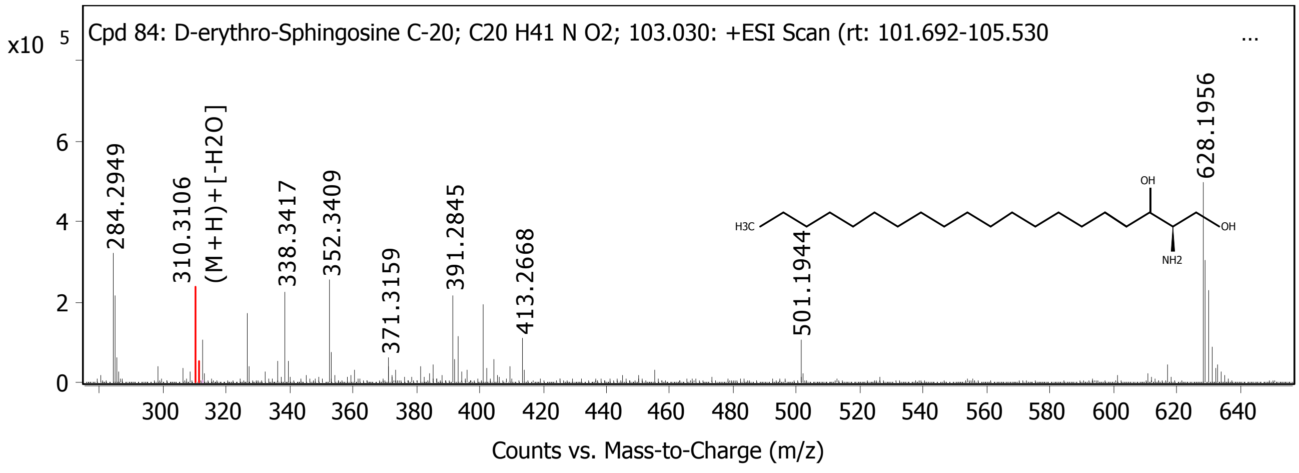

MSMS Spectrum

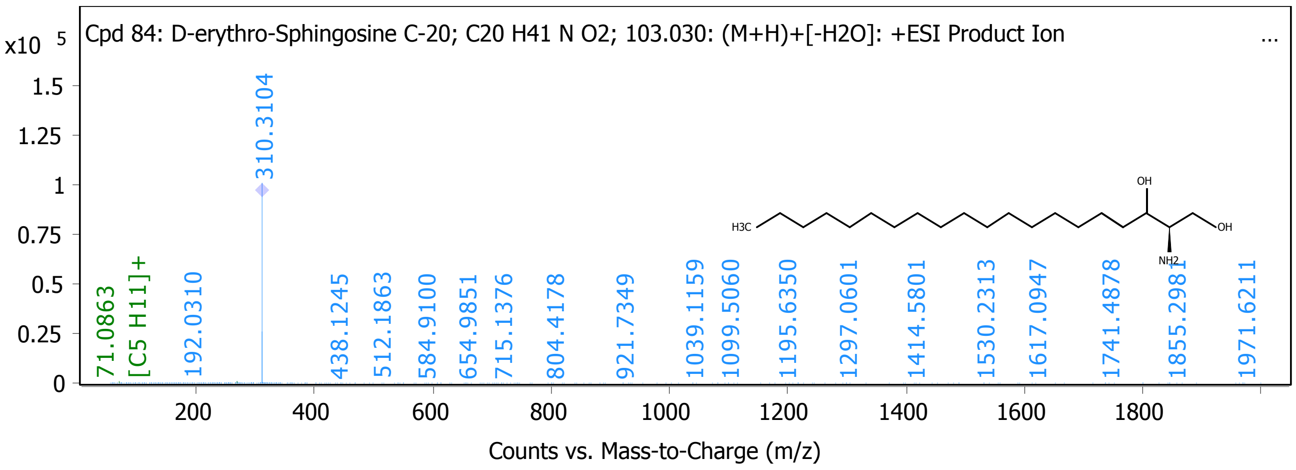

Identification Hit Table

Best Hit	Compound Name	RT	Formula	Notes	Match Score	Mass	Difference	Ion Species
✓	D-erythro-Sphingosine C-20	103.03	C20 H41 N O2		85.64	327.314	-0.3	(M+H)+[-H2O]
	N,N-Dimethylsphingosine	103.03	C20 H41 N O2	Enzyme Inhibitors N,N-dimethylsphingosine Ambap3701 N,N-DMS 4-Octadecene-1,3-diol, 2-(dimethylamino)-, (S-(R*,S*-(E)))- PubChemCID: 6438166	85.64	327.314	-0.3	(M+H)+[-H2O]
	Stearoylethanolamide	103.03	C20 H41 N O2	18:0 N-acyl ethanolamine http://dx.doi.org/10.1021/bi0480335	85.64	327.314	-0.3	(M+H)+[-H2O]
	N-Hexadecanoylpyrrolidine	103.03	C20 H39 N O		85.62	309.3035	-0.3	(M+H)+
	Oleoyl Ethyl Amide	103.03	C20 H39 N O		85.62	309.3035	-0.3	(M+H)+
	Oleoyl Ethanolamide-d2	103.03	C20 H37 D2 N O2		60.02	327.314	-3.4	(M+H)+[-H2O]
	Oleoyl-EA(d2)	103.03	C20 H37 D2 N O2		60.02	327.314	-3.4	(M+H)+[-H2O]

Identification Hit Table

Best Hit	Compound Name	RT	Formula	Notes	Match Score	Mass	Difference	Ion Species
✓	N,N-Dimethylsphingosine	103.03	C20 H41 N O2	Enzyme Inhibitors N,N-dimethylsphingosine Ambap3701 N,N-DMS 4-Octadecene-1,3-diol, 2-(dimethylamino)-, (S-(R*,S*-(E)))- PubChemCID: 6438166	85.64	327.314	-0.3	(M+H)+[-H2O]
	Stearoylethanolamide	103.03	C20 H41 N O2	18:0 N-acyl ethanolamine http://dx.doi.org/10.1021/bi0480335	85.64	327.314	-0.3	(M+H)+[-H2O]
	Oleoyl-EA(d2)	103.03	C20 H37 D2 N O2		60.02	327.314	-3.4	(M+H)+[-H2O]

Identification Hit Table

Best Hit	Compound Name	RT	Formula	Notes	Match Score	Mass	Difference	Ion Species

Compound Label	Name	*m/z*	RT	Algorithm	Mass
Cpd 85: Isopropyl stearate; C21 H42 O2; 103.249	**Isopropyl stearate**	326.342	103.249	Find by Molecular Feature	326.3188

Compound Chromatograms

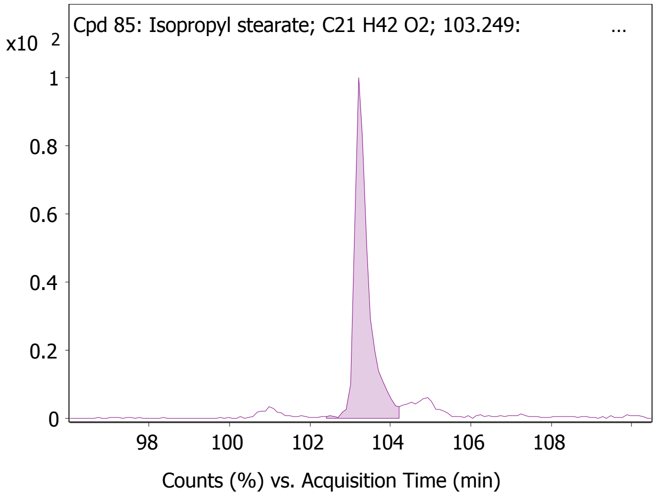

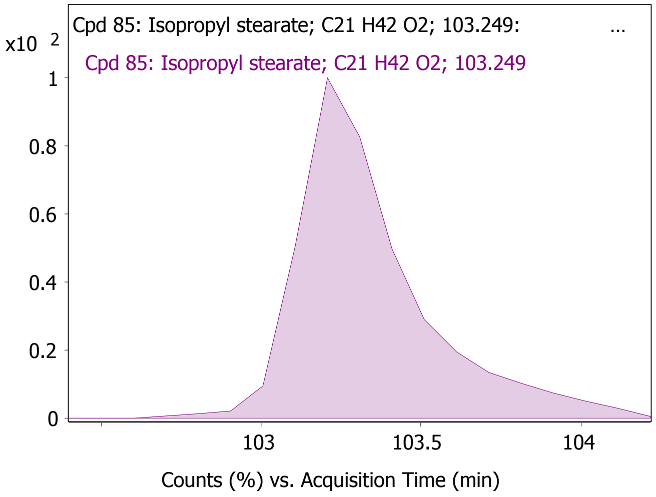

MFE MS Spectrum

Qualitative Compound Identification Report

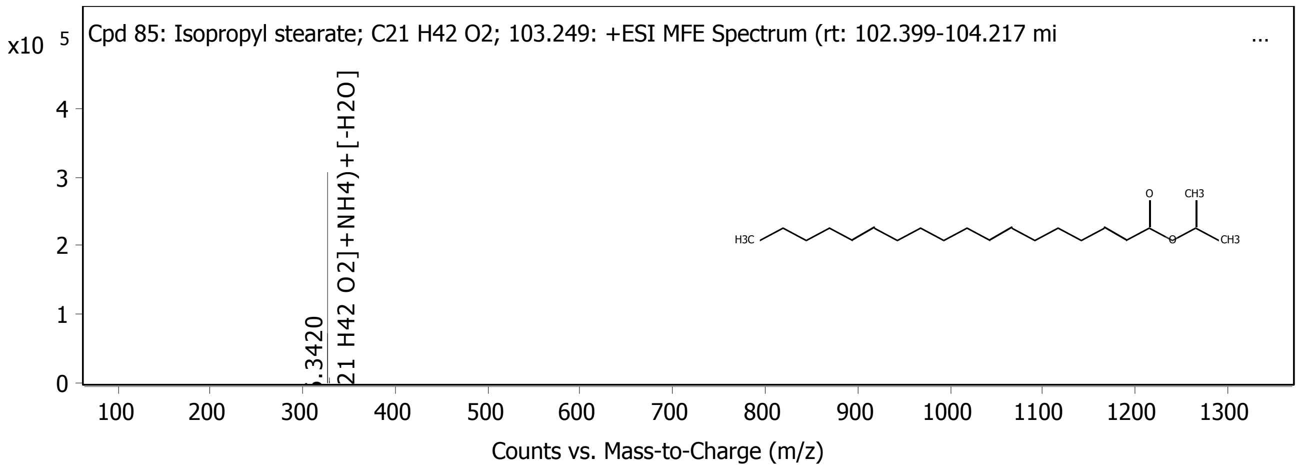

MFE MS Zoomed Spectrum

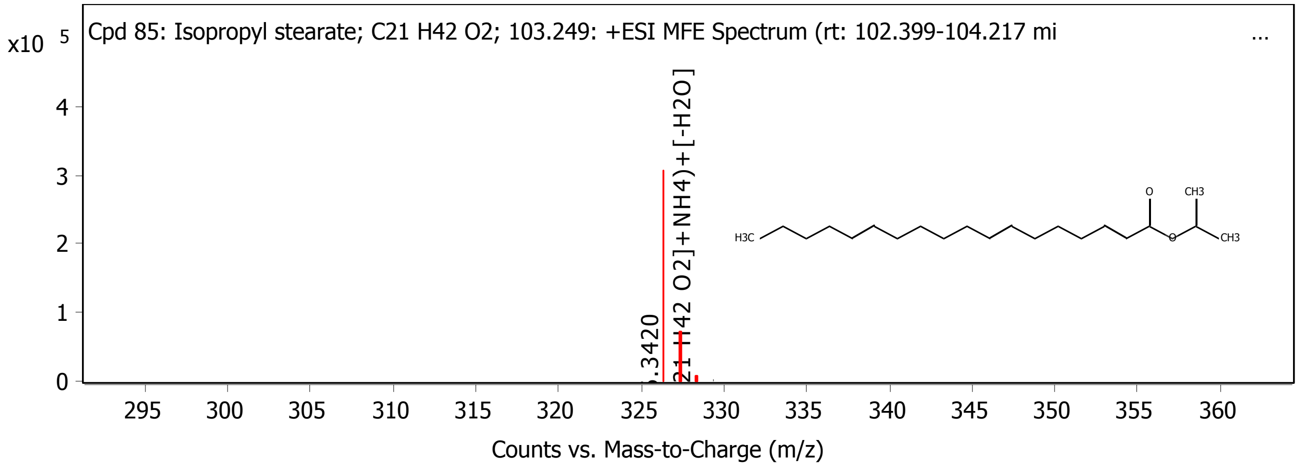

MS Spectrum

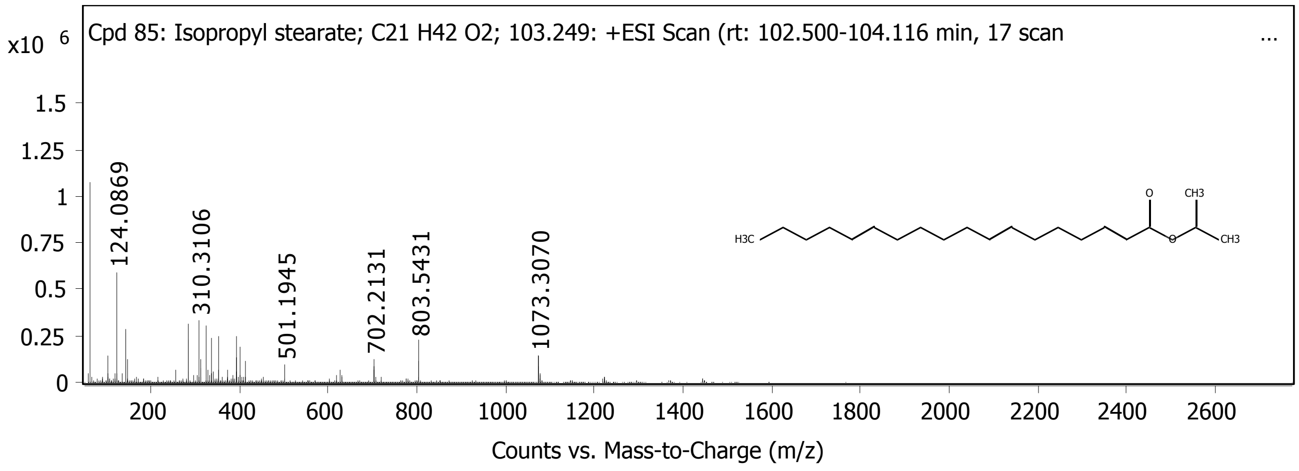

MS Zoomed Spectrum

Qualitative Compound Identification Report

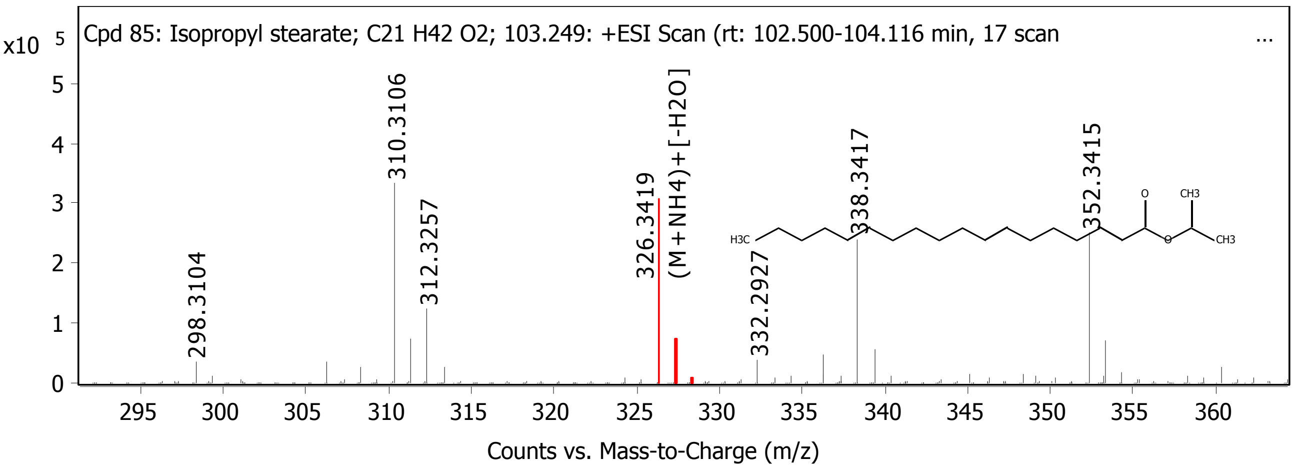

MSMS Spectrum

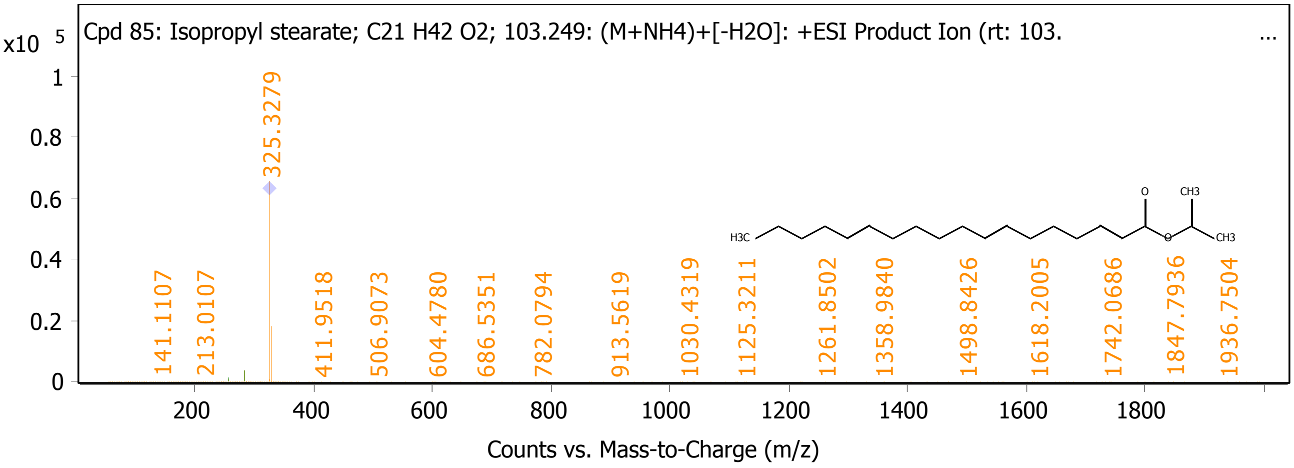

Identification Hit Table

Best Hit	Compound Name	RT	Formula	Notes	Match Score	Mass	Difference	Ion Species
✓	Isopropyl stearate	103.249	C21 H42 O2		99.58	326.3188	-0.29	(M+NH4)+[-H2O]
	4-Hydroxy-6-heneicosanone	103.249	C21 H42 O2		99.58	326.3188	-0.29	(M+NH4)+[-H2O]
	8-Hydroxy-6-heneicosanone	103.249	C21 H42 O2		99.58	326.3188	-0.29	(M+NH4)+[-H2O]
	Heneicosanoic acid	103.249	C21 H42 O2	http://lipidmaps.org/data/get_lm_lipids_dbgif.php?LM_ID=LMFA01010021	99.58	326.3188	-0.29	(M+NH4)+[-H2O]
	(+)-18-Methyl-eicosanoic acid	103.249	C21 H42 O2	IUPAC Name: 18-methylicosanoic acid http://lipidmaps.org/data/get_lm_lipids_dbgif.php?LM_ID=LMFA01020018	99.58	326.3188	-0.29	(M+NH4)+[-H2O]
	2,6-Dimethyl-nonadecanoic acid	103.249	C21 H42 O2	http://lipidmaps.org/data/get_lm_lipids_dbgif.php?LM_ID=LMFA01020033	99.58	326.3188	-0.29	(M+NH4)+[-H2O]
	19-Methyl-eicosanoic acid	103.249	C21 H42 O2		99.58	326.3188	-0.29	(M+NH4)+[-H2O]
	Homophytanic acid	103.249	C21 H42 O2		99.58	326.3188	-0.29	(M+NH4)+[-H2O]
	20:0(12Me)	103.249	C21 H42 O2		99.58	326.3188	-0.29	(M+NH4)+[-H2O]
	Eicosanoic acid, methyl ester	103.249	C21 H42 O2		99.58	326.3188	-0.29	(M+NH4)+[-H2O]

Identification Hit Table

Best Hit	Compound Name	RT	Formula	Notes	Match Score	Mass	Difference	Ion Species
✓	Heneicosanoic acid	103.249	C21 H42 O2	http://lipidmaps.org/data/get_lm_lipids_dbgif.php?LM_ID=LMFA01010021	99.58	326.3188	-0.29	(M+NH4)+[-H2O]
	dodecyl nonanoate	103.249	C21 H42 O2		99.58	326.3188	-0.29	(M+NH4)+[-H2O]
	Homophytanic acid	103.249	C21 H42 O2		99.58	326.3188	-0.29	(M+NH4)+[-H2O]
	10,14-Dimethylpentadecyl isobutyrate	103.249	C21 H42 O2		99.58	326.3188	-0.29	(M+NH4)+[-H2O]
	3-Methyl-2-butenyl hexadecanoate	103.249	C21 H42 O2		99.58	326.3188	-0.29	(M+NH4)+[-H2O]
	(+)-18-Methyl-eicosanoic acid	103.249	C21 H42 O2	IUPAC Name: 18-methylicosanoic acid http://lipidmaps.org/data/get_lm_lipids_dbgif.php?LM_ID=LMFA01020018	99.58	326.3188	-0.29	(M+NH4)+[-H2O]
	hexadecyl 2-methyl-butanoate	103.249	C21 H42 O2		99.58	326.3188	-0.29	(M+NH4)+[-H2O]
	10,14-Dimethylpentadecyl butyrate	103.249	C21 H42 O2		99.58	326.3188	-0.29	(M+NH4)+[-H2O]
	pentadecyl hexanoate	103.249	C21 H42 O2		99.58	326.3188	-0.29	(M+NH4)+[-H2O]
	octadecyl propionate	103.249	C21 H42 O2		99.58	326.3188	-0.29	(M+NH4)+[-H2O]

Identification Hit Table

Qualitative Compound Identification Report

Best Hit	Compound Name	RT	Formula	Notes	Match Score	Mass	Difference	Ion Species

Compound Label	Name	*m/z*	RT	Algorithm	Mass
Cpd 86: Eicosanoyl-EA; C22 H45 N O2; 103.898	**Eicosanoyl-EA**	338.3417	103.898	Find by Molecular Feature	355.345

Compound Chromatograms

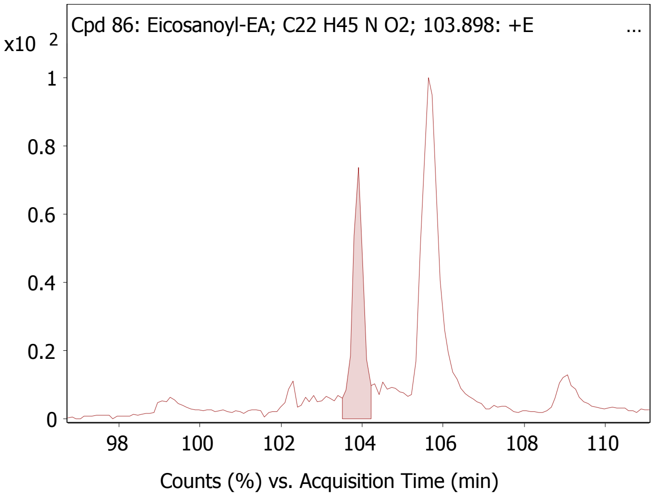

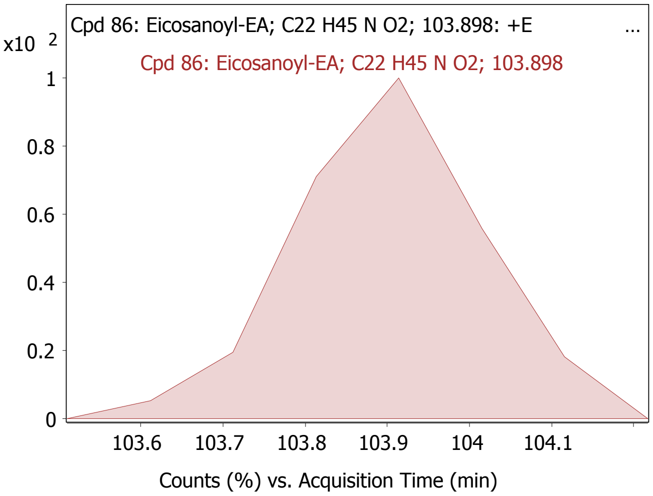

MFE MS Spectrum

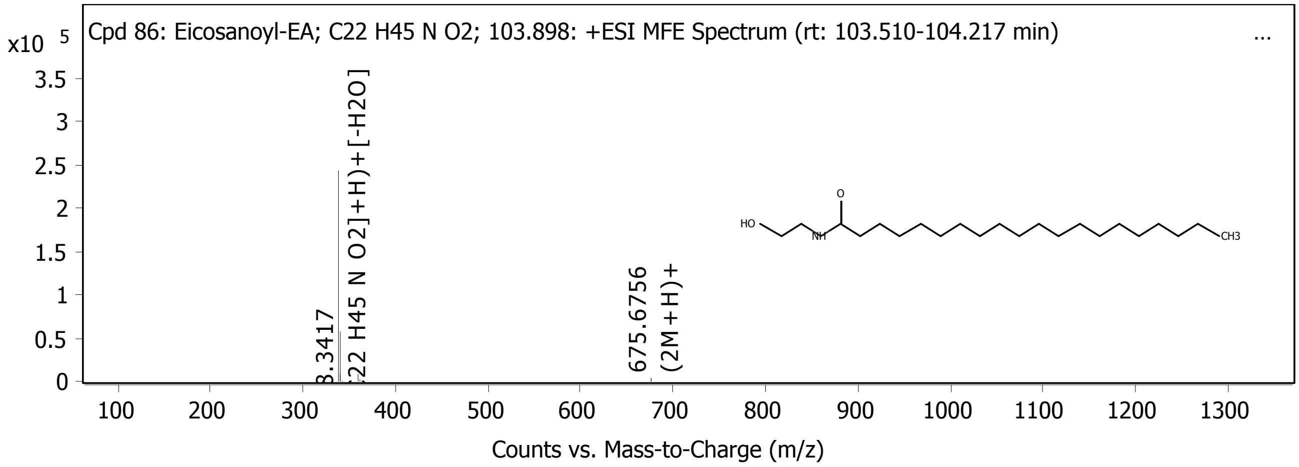

MFE MS Zoomed Spectrum

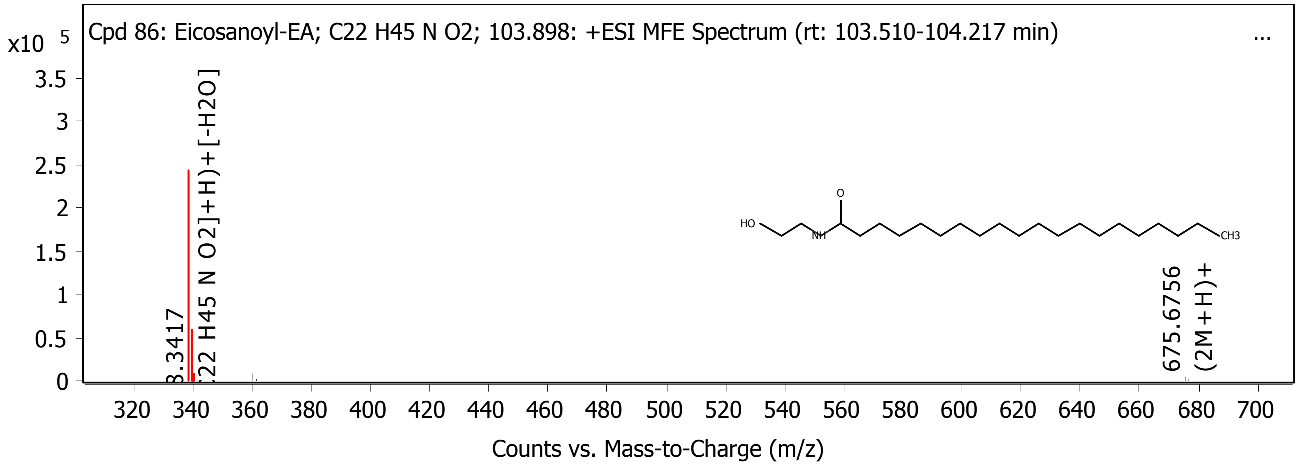

Qualitative Compound Identification Report

MS Spectrum

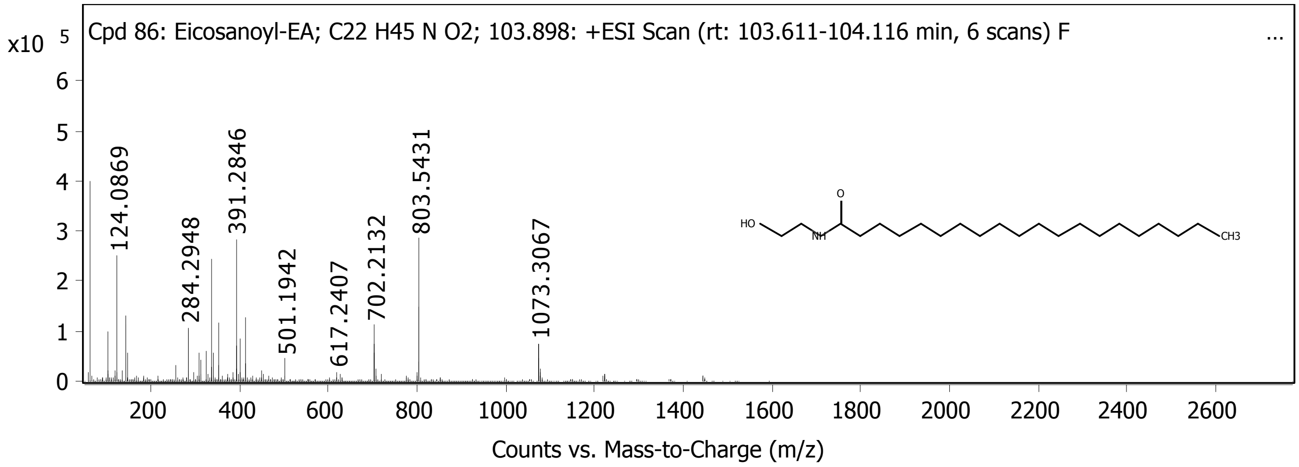

MS Zoomed Spectrum

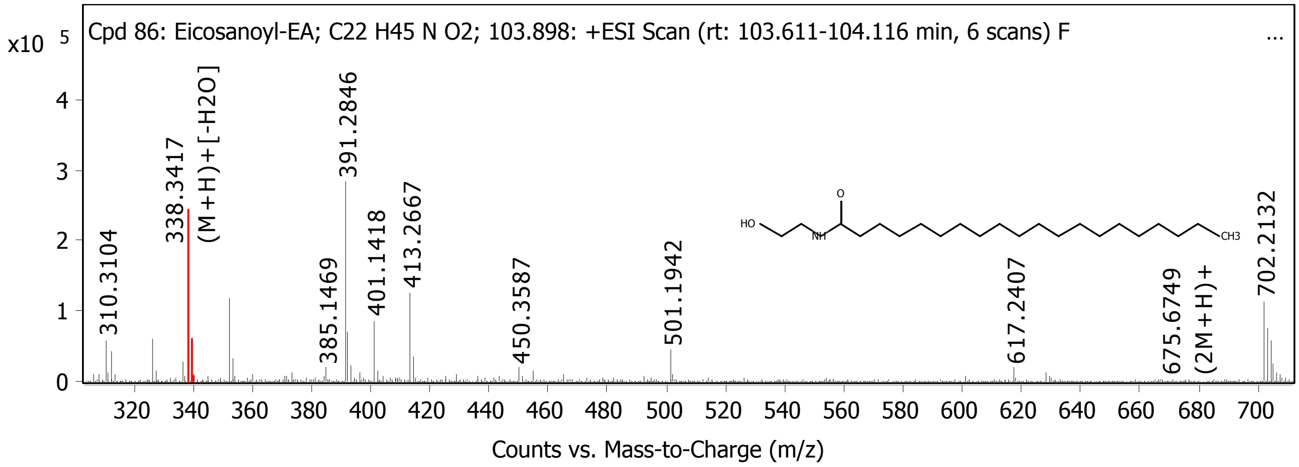

MSMS Spectrum

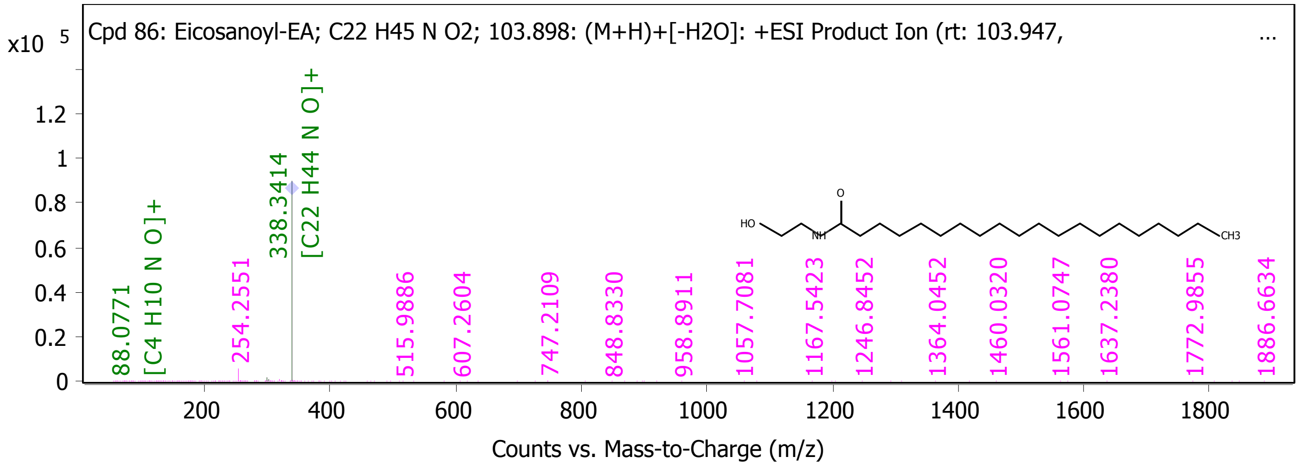

Identification Hit Table

Best Hit	Compound Name	RT	Formula	Notes	Match Score	Mass	Difference	Ion Species
✓	Eicosanoyl-EA	103.898	C22 H45 N O2	20:0 N-acyl ethanolamine http://dx.doi.org/10.1021/bi0480335	99.53	355.345	0.01	(M+H)+[-H2O]
	13E-Docosenamide	103.898	C22 H43 N O		99.53	337.3345	0.01	(M+H)+
	13Z-Docosenamide	103.898	C22 H43 N O		99.53	337.3345	0.01	(M+H)+
	N-Cyclohexanecarbonylpentadecylamine	103.898	C22 H43 N O		99.53	337.3345	0.01	(M+H)+

Identification Hit Table

Best Hit	Compound Name	RT	Formula	Notes	Match Score	Mass	Difference	Ion Species
✓								

Qualitative Compound Identification Report

	Eicosanoyl-EA	103.898	C22 H45 N O2	20:0 N-acyl ethanolamine http://dx.doi.org/10.1021/bi0480335	99.53	355.345	0.01	(M+H)+[-H2O]

Identification Hit Table

Best Hit	Compound Name	RT	Formula	Notes	Match Score	Mass	Difference	Ion Species

Compound Label	Name	*m/z*	RT	Algorithm	Mass
Cpd 87: 3α,11β,12α-Trihydroxy-5β-cholan-24-oic Acid; C24 H40 O5; 104.112	**3α,11β,12α-Trihydroxy-5β-cholan-24-oic Acid**	391.2846	104.112	Find by Molecular Feature	408.2878

Compound Chromatograms

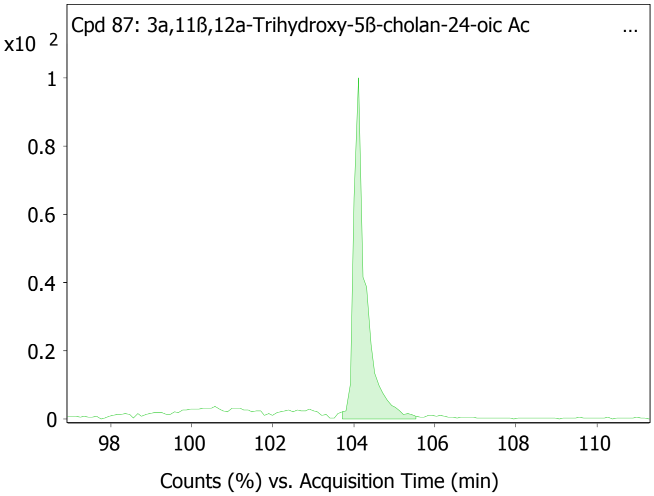

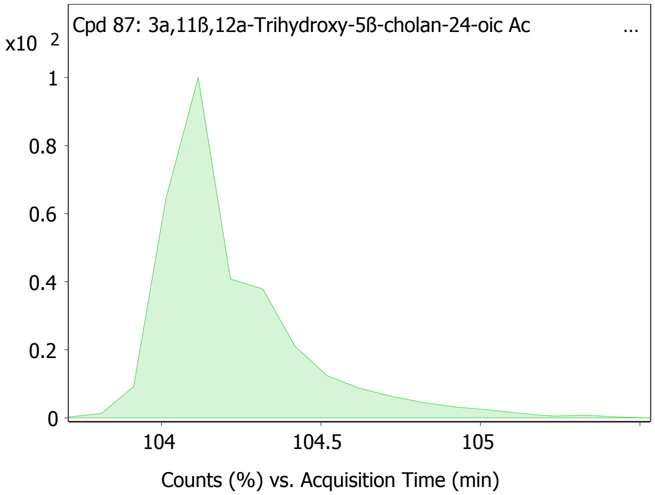

MFE MS Spectrum

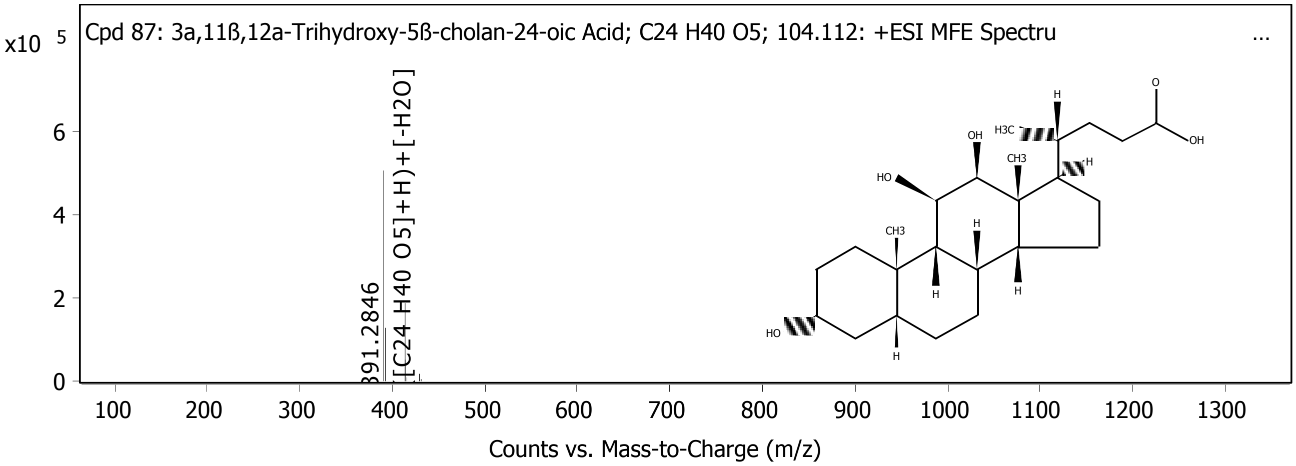

MFE MS Zoomed Spectrum

Qualitative Compound Identification Report

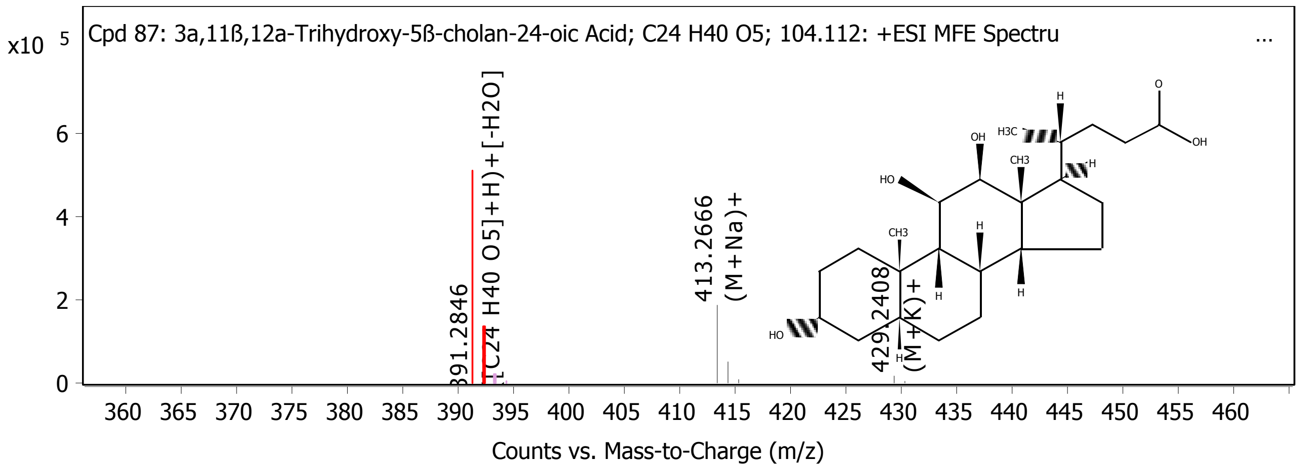

MS Spectrum

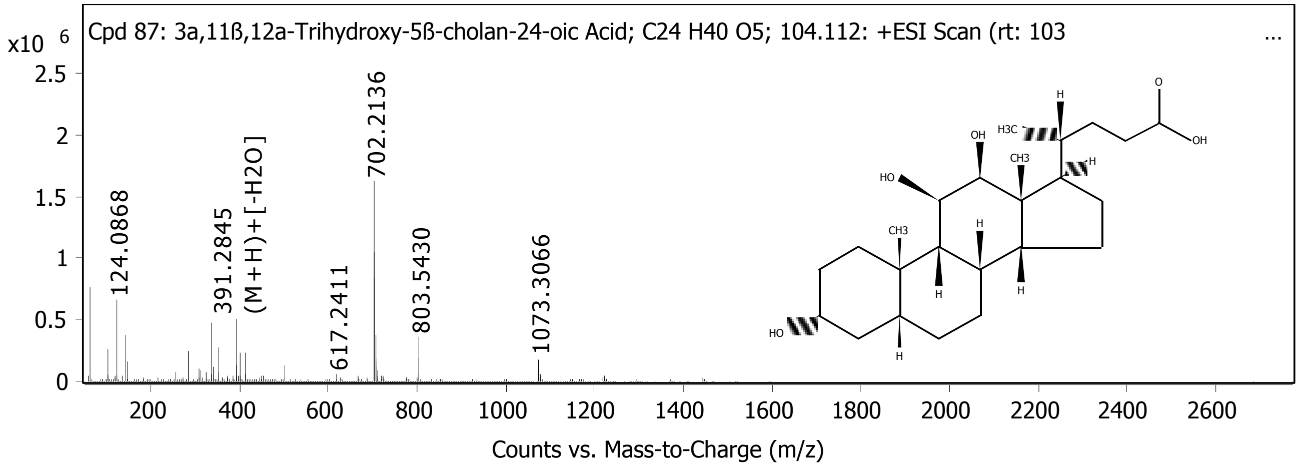

MS Zoomed Spectrum

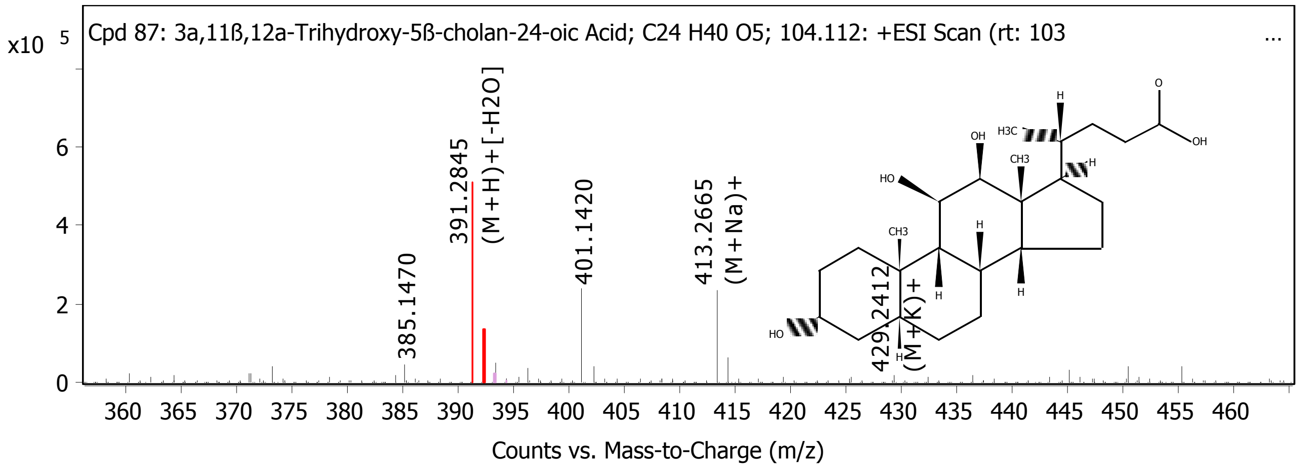

Library Spectrum

Difference Spectrum

MSMS Spectrum

Qualitative Compound Identification Report

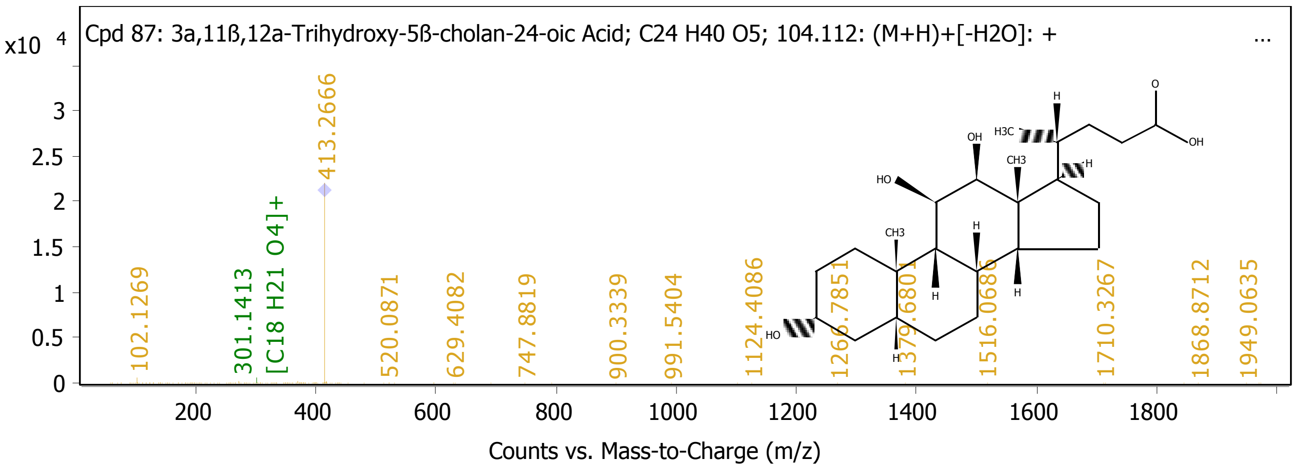

MSMS Spectrum

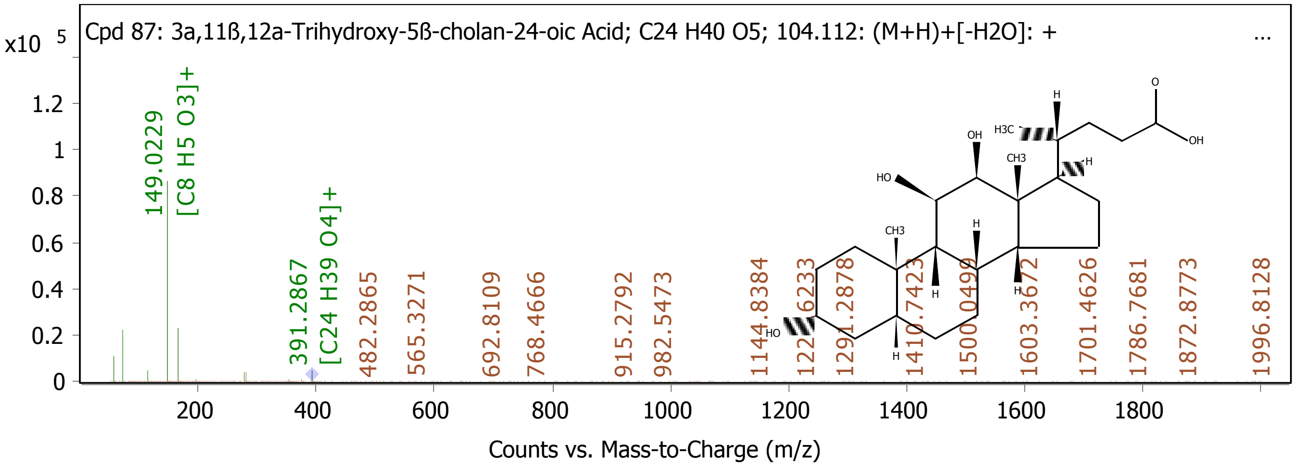

Identification Hit Table

Best Hit	Compound Name	RT	Formula	Notes	Match Score	Mass	Difference	Ion Species
✓	3α,11β,12α-Trihydroxy-5β-cholan-24-oic Acid	104.112	C24 H40 O5		83.03	408.2878	-0.27	(M+H)+[-H2O]
	3β,7β,12β-Trihydroxy-5α-cholan-24-oic Acid	104.112	C24 H40 O5		83.03	408.2878	-0.27	(M+H)+[-H2O]
	(22S)-3α,7α,22-Trihydroxy-5β-cholan-24-oic Acid	104.112	C24 H40 O5		83.03	408.2878	-0.27	(M+H)+[-H2O]
	3α,11β,15β-Trihydroxy-5β-cholan-24-oic Acid	104.112	C24 H40 O5		83.03	408.2878	-0.27	(M+H)+[-H2O]
	3α,11α,15β-Trihydroxy-5β-cholan-24-oic Acid	104.112	C24 H40 O5		83.03	408.2878	-0.27	(M+H)+[-H2O]
	3α,7α,16α-Trihydroxy-5β-cholan-24-oic Acid	104.112	C24 H40 O5		83.03	408.2878	-0.27	(M+H)+[-H2O]
	3β,9α,11β-Trihydroxy-5β-cholan-24-oic Acid	104.112	C24 H40 O5		83.03	408.2878	-0.27	(M+H)+[-H2O]
	3α,9α,11β-Trihydroxy-5β-cholan-24-oic Acid	104.112	C24 H40 O5		83.03	408.2878	-0.27	(M+H)+[-H2O]
	(23S)-3α,7α,23-Trihydroxy-5β-cholan-24-oic Acid	104.112	C24 H40 O5		83.03	408.2878	-0.27	(M+H)+[-H2O]
	(23R)-3α,7α,23-Trihydroxy-5β-cholan-24-oic Acid	104.112	C24 H40 O5		83.03	408.2878	-0.27	(M+H)+[-H2O]

Identification Hit Table

Best Hit	Compound Name	RT	Formula	Notes	Match Score	Mass	Difference	Ion Species
✓	3alpha,7beta,14alpha-Trihydroxy-5beta-cholan-24-oic Acid	104.112	C24 H40 O5		83.03	408.2878	-0.27	(M+H)+[-H2O]
	3alpha,9alpha,11beta-Trihydroxy-5beta-cholan-24-oic Acid	104.112	C24 H40 O5		83.03	408.2878	-0.27	(M+H)+[-H2O]
	Avicholic acid	104.112	C24 H40 O5		83.03	408.2878	-0.27	(M+H)+[-H2O]
	1beta,3alpha,7beta-Trihydroxy-5beta-cholan-24-oic Acid	104.112	C24 H40 O5		83.03	408.2878	-0.27	(M+H)+[-H2O]
	Bitocholic acid	104.112	C24 H40 O5		83.03	408.2878	-0.27	(M+H)+[-H2O]
	3alpha,15beta,18-Trihydroxy-5beta-cholan-24-oic Acid	104.112	C24 H40 O5		83.03	408.2878	-0.27	(M+H)+[-H2O]
	3alpha,12alpha,16alpha-Trihydroxy-5beta-cholan-24-oic Acid	104.112	C24 H40 O5		83.03	408.2878	-0.27	(M+H)+[-H2O]
	3alpha,12alpha,15alpha-Trihydroxy-5beta-cholan-24-oic Acid	104.112	C24 H40 O5		83.03	408.2878	-0.27	(M+H)+[-H2O]
	3alpha,11beta,15beta-Trihydroxy-5beta-cholan-24-oic Acid	104.112	C24 H40 O5		83.03	408.2878	-0.27	(M+H)+[-H2O]
	3alpha,11alpha,15beta-	104.112	C24 H40 O5		83.03	408.2878	-0.27	(M+H)+[-H2O]

Qualitative Compound Identification Report

	Trihydroxy-5beta-cholan-24-oic Acid							

Identification Hit Table

Best Hit	Compound Name	RT	Formula	Notes	Match Score	Mass	Difference	Ion Species

Compound Label	Name	*m/z*	RT	Algorithm	Mass
Cpd 88: Dihydrostreptomycin 3'α-phosphate; C21 H42 N7 O15 P; 105.212	**Dihydrostreptomycin 3'α-phosphate**	702.2137	105.212	Find by Molecular Feature	663.2488

Compound Chromatograms

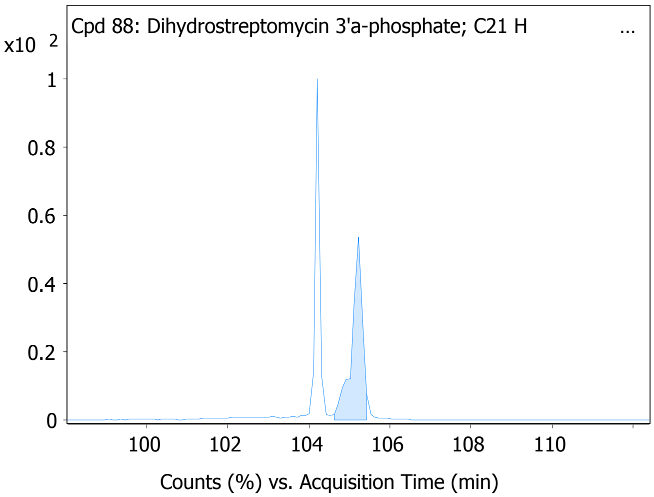

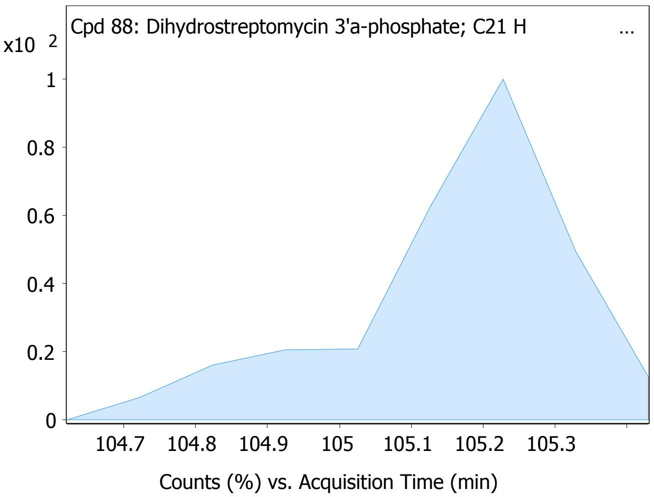

MFE MS Spectrum

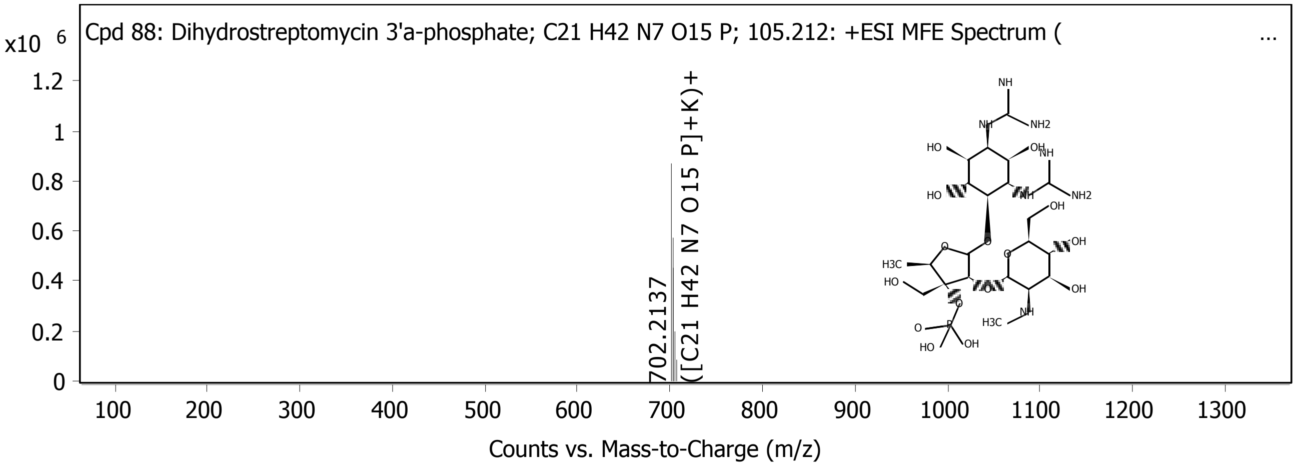

MFE MS Zoomed Spectrum

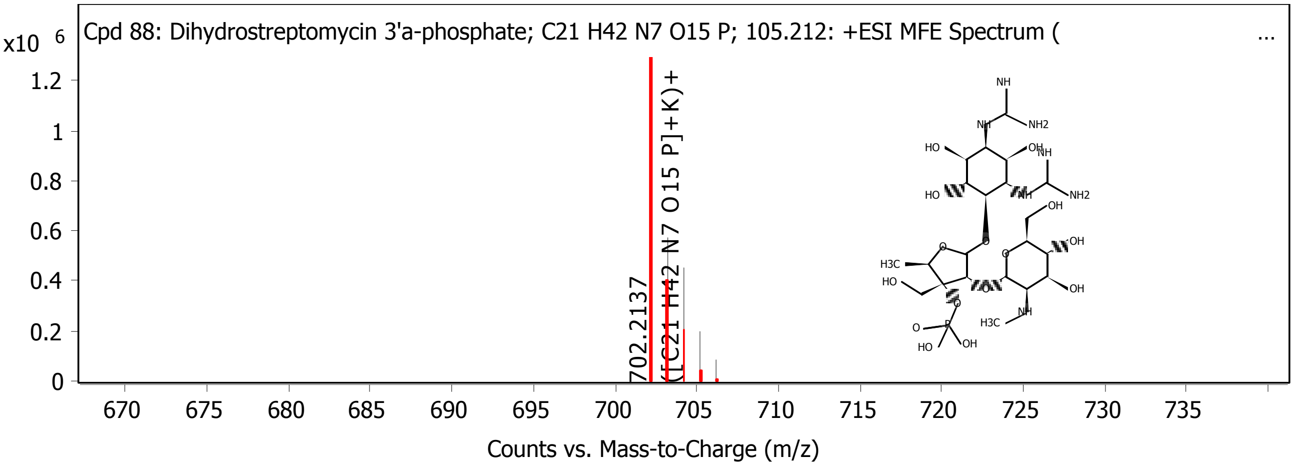

MS Spectrum

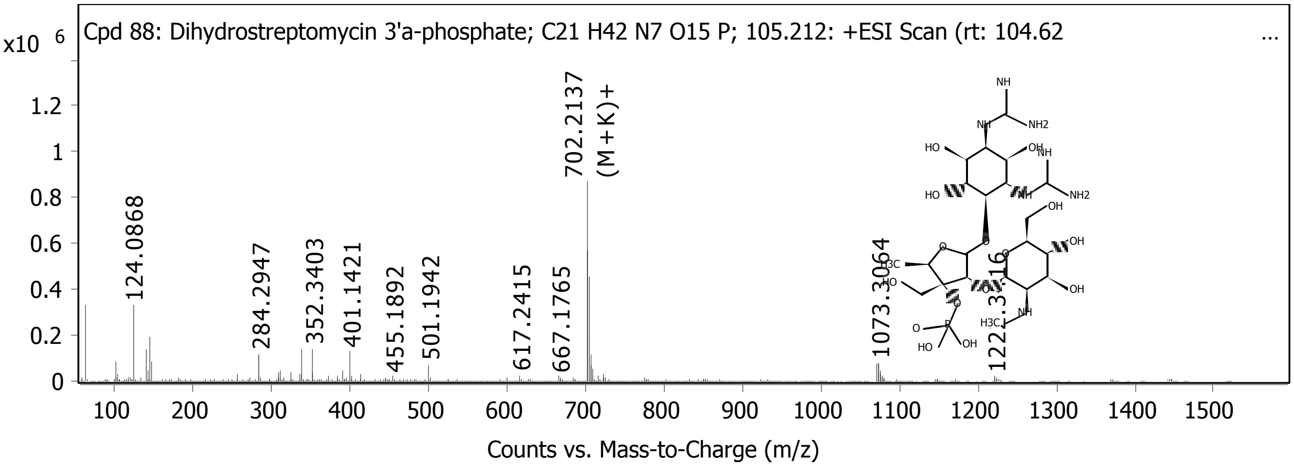

MS Zoomed Spectrum

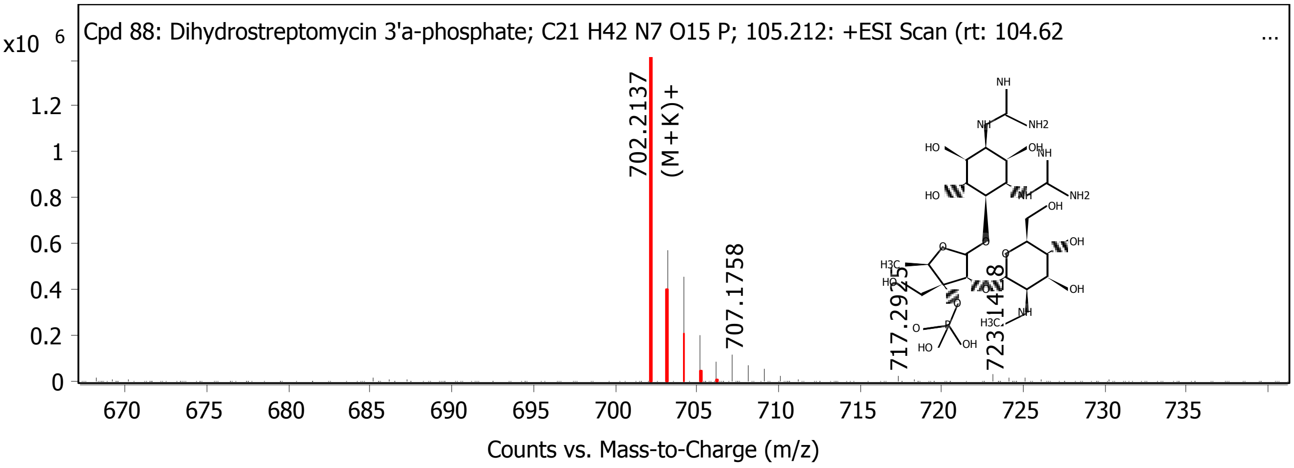

MSMS Spectrum

Qualitative Compound Identification Report

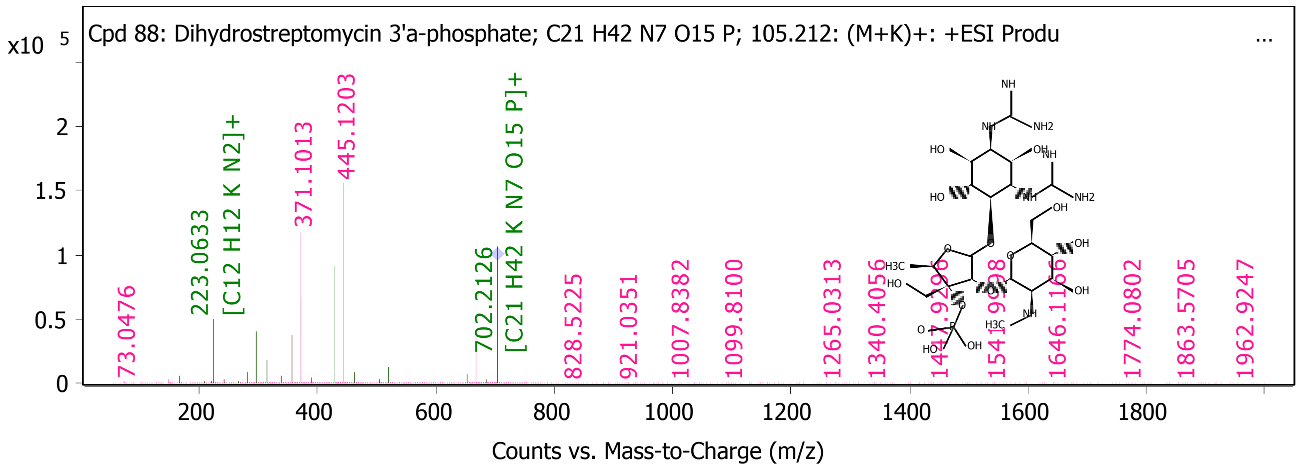

Identification Hit Table

Best Hit	Compound Name	RT	Formula	Notes	Match Score	Mass	Difference	Ion Species
✓	Dihydrostreptomycin 3'a-phosphate	105.212	C21 H42 N7 O15 P	Antibiotic, Treatment of tuberculosis Metabolite of Streptomycin Dollery, Colin Therapeutic Drugs, 2nd Ed. 1999 p. S102	66.7	663.2488	-1.19	(M+K)+
	Dihydrostreptomycin 3''-phosphate	105.212	C21 H42 N7 O15 P	Antibiotic, Treatment of tuberculosis Metabolite of Streptomycin Dollery, Colin Therapeutic Drugs, 2nd Ed. 1999 p. S102	66.7	663.2488	-1.19	(M+K)+
	Dihydrostreptomycin 6-phosphate	105.212	C21 H42 N7 O15 P	Antibiotic, Treatment of tuberculosis Metabolite of Streptomycin Dollery, Colin Therapeutic Drugs, 2nd Ed. 1999 p. S102	66.7	663.2488	-1.19	(M+K)+
	Ramontoside	105.212	C34 H38 O16		35.73	702.2107	5.31	M+
	Hydroxyitraconazole	105.212	C35 H38 Cl2 N8 O5	Antifungal Metabolite of Itraconazole Dollery, Colin Therapeutic Drugs, 2nd Ed. 1999 p. I120	26.27	720.2243	9.93	M+[-H2O]

Identification Hit Table

Best Hit	Compound Name	RT	Formula	Notes	Match Score	Mass	Difference	Ion Species

Identification Hit Table

Best Hit	Compound Name	RT	Formula	Notes	Match Score	Mass	Difference	Ion Species
✓	Trp Gln Asp Cys Glu	105.212	C28 H37 N7 O11 S		46.83	679.2227	4.48	(M+Na)+

Compound Label	Name	*m/z*	RT	Algorithm	Mass
Cpd 89: 2'',3''-Di-O-p-coumaroylafzelin; C39 H32 O14; 105.221	**2'',3''-Di-O-p-coumaroylafzelin**	707.1753	105.221	Find by Molecular Feature	724.1717

Compound Chromatograms

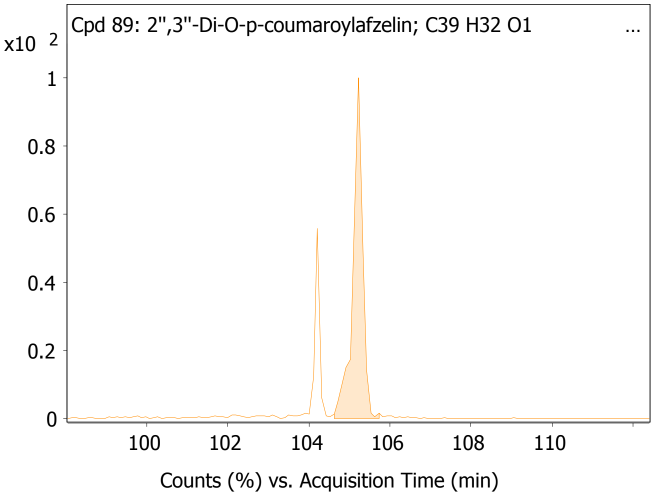

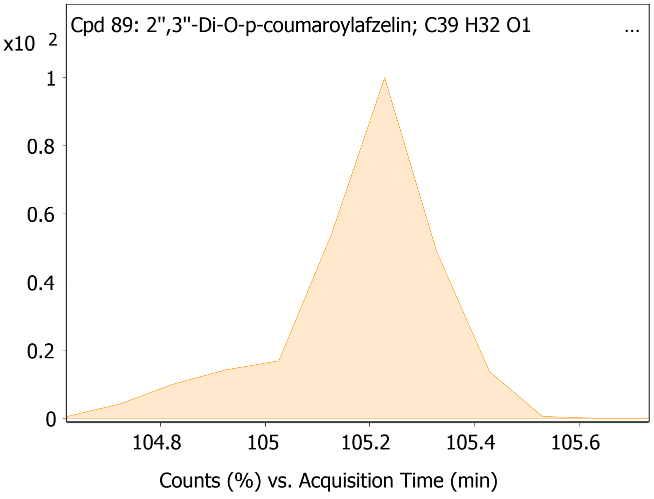

MFE MS Spectrum

Qualitative Compound Identification Report

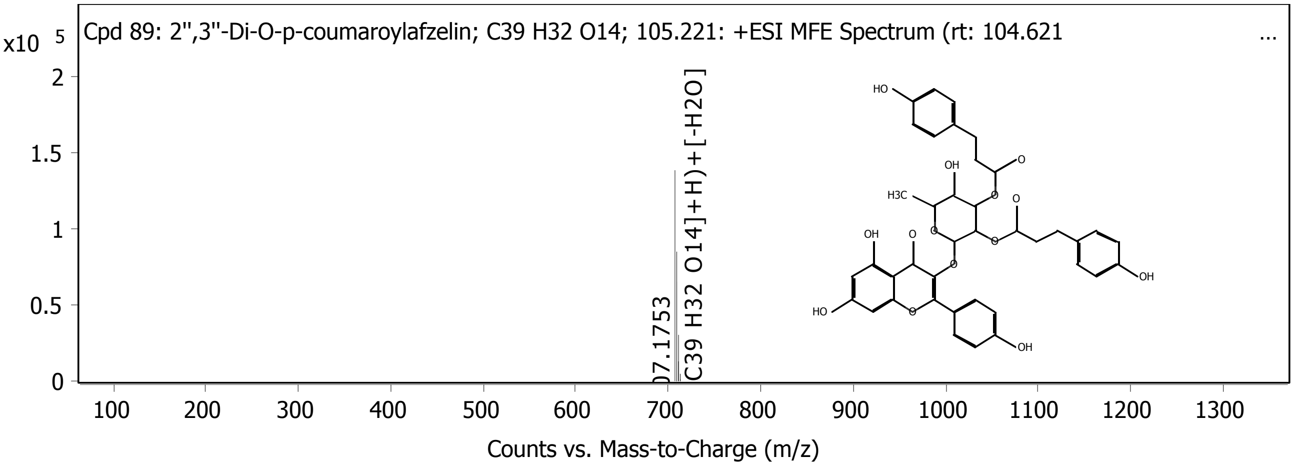

MFE MS Zoomed Spectrum

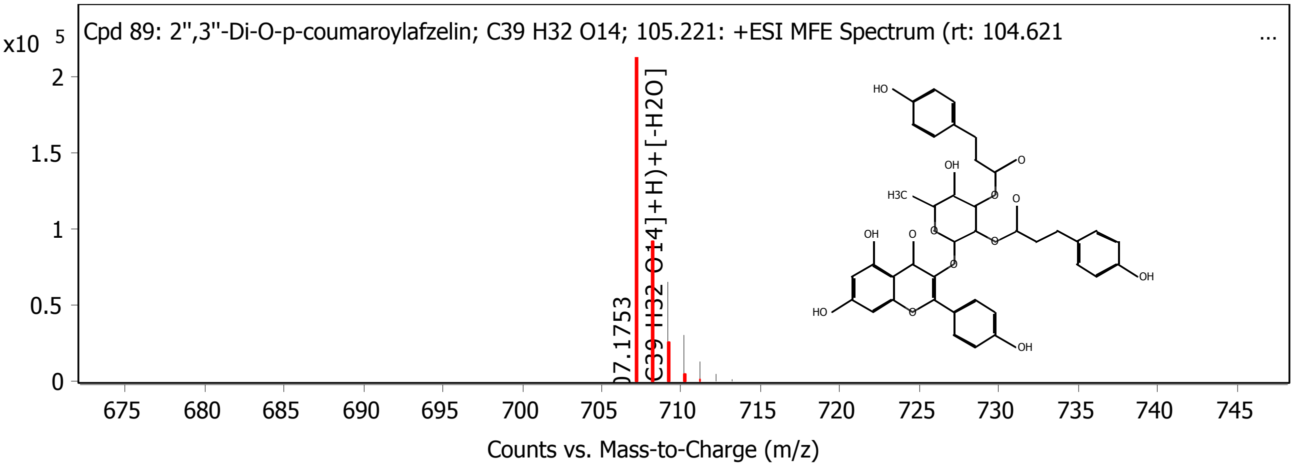

MS Spectrum

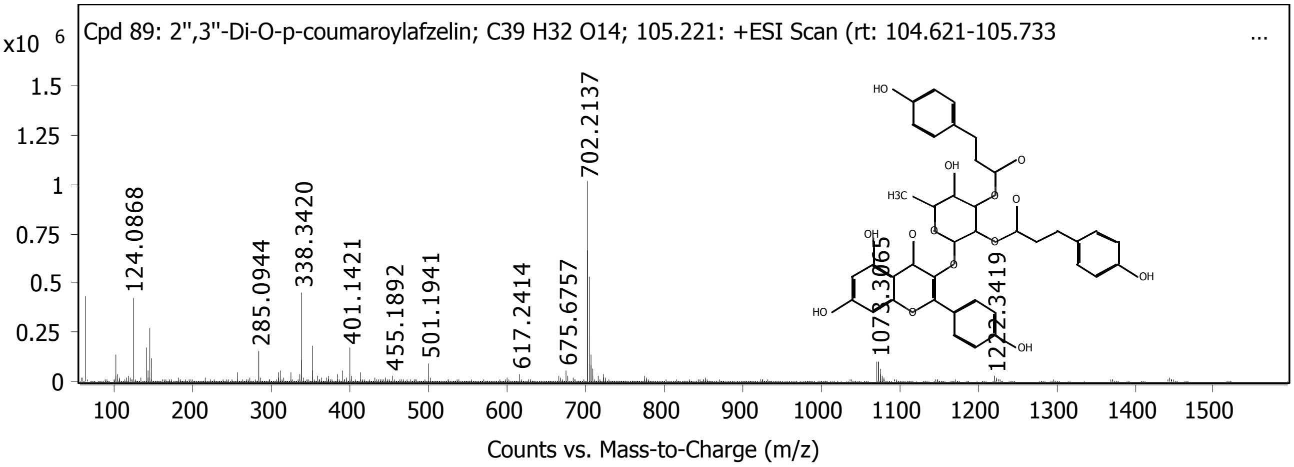

MS Zoomed Spectrum

Qualitative Compound Identification Report

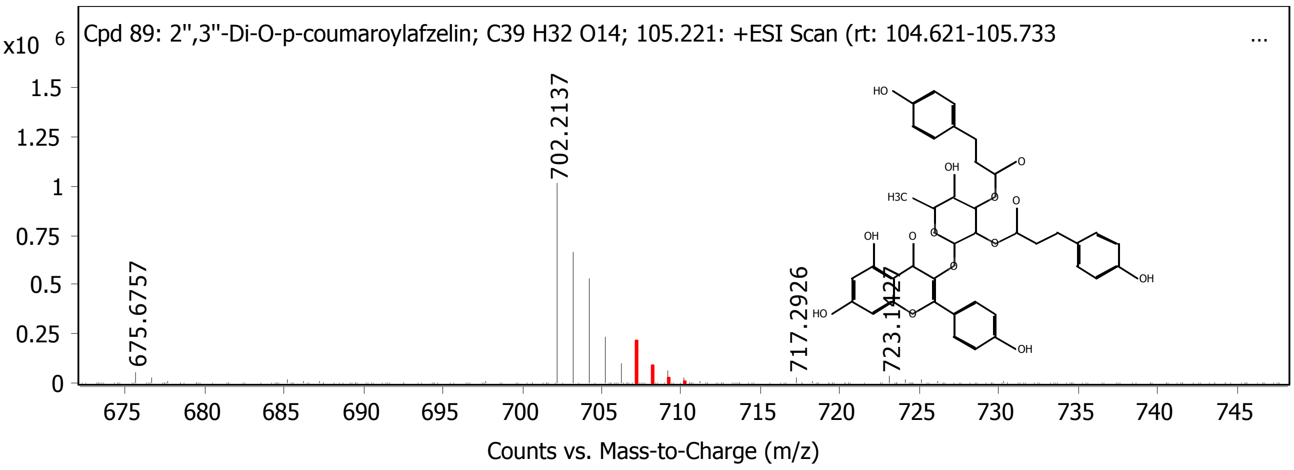

MSMS Spectrum

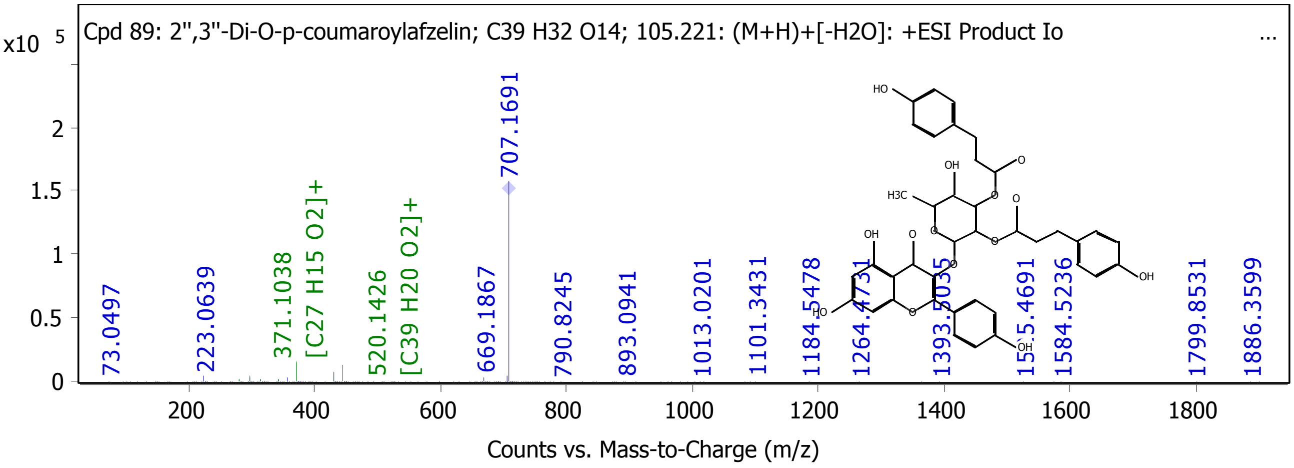

Identification Hit Table

Best Hit	Compound Name	RT	Formula	Notes	Match Score	Mass	Difference	Ion Species
✓	2",3"-Di-O-p-coumaroylafzelin	105.221	C39 H32 O14		16.72	724.1717	7.46	(M+H)+[-H2O]
	Kaempferol 3-(2",4"-di-(Z)-p-coumaroylrhamnoside)	105.221	C39 H32 O14		16.72	724.1717	7.46	(M+H)+[-H2O]
	Kaempferol 3-(3",4"-di-p-coumarylrhamnoside)	105.221	C39 H32 O14		16.72	724.1717	7.46	(M+H)+[-H2O]
	Kaempferol 3-(2",4"-di-(E)-p-coumarylrhamnoside)	105.221	C39 H32 O14		16.72	724.1717	7.46	(M+H)+[-H2O]
	Platanoside	105.221	C39 H32 O14		16.72	724.1717	7.46	(M+H)+[-H2O]
	Apigenin 7-(3",6"-Di-E-p-coumaroylgalactoside)	105.221	C39 H32 O14		16.72	724.1717	7.46	(M+H)+[-H2O]
	Apigenin 7-(4",6"-di-p-coumarylglucoside)	105.221	C39 H32 O14		16.72	724.1717	7.46	(M+H)+[-H2O]
	Apigenin 7-(3",6"-di-p-coumarylglucoside)	105.221	C39 H32 O14		16.72	724.1717	7.46	(M+H)+[-H2O]
	Apigenin 7-(2",6"-di-p-coumarylglucoside)	105.221	C39 H32 O14		16.72	724.1717	7.46	(M+H)+[-H2O]

Identification Hit Table

Best Hit	Compound Name	RT	Formula	Notes	Match Score	Mass	Difference	Ion Species
✓	Apigenin 7-(2",6"-di-p-coumarylglucoside)	105.221	C39 H32 O14		16.72	724.1717	7.46	(M+H)+[-H2O]
	Apigenin 7-(3",6"-di-p-coumarylglucoside)	105.221	C39 H32 O14		16.72	724.1717	7.46	(M+H)+[-H2O]
	Apigenin 7-(4",6"-di-p-coumarylglucoside)	105.221	C39 H32 O14		16.72	724.1717	7.46	(M+H)+[-H2O]
	Apigenin 7-(3",6"-Di-E-p-coumaroylgalactoside)	105.221	C39 H32 O14		16.72	724.1717	7.46	(M+H)+[-H2O]
	Platanoside	105.221	C39 H32 O14		16.72	724.1717	7.46	(M+H)+[-H2O]
	Kaempferol 3-(2",4"-di-(E)-p-coumarylrhamnoside)	105.221	C39 H32 O14		16.72	724.1717	7.46	(M+H)+[-H2O]
	Kaempferol 3-(3",4"-di-p-coumarylrhamnoside)	105.221	C39 H32 O14		16.72	724.1717	7.46	(M+H)+[-H2O]
	Kaempferol 3-(2",4"-di-(Z)-p-coumaroylrhamnoside)	105.221	C39 H32 O14		16.72	724.1717	7.46	(M+H)+[-H2O]

Identification Hit Table

Best Hit	Compound Name	RT	Formula	Notes	Match Score	Mass	Difference	Ion Species

Qualitative Compound Identification Report

Compound Label	Name	*m/z*	RT	Algorithm	Mass
Cpd 90: Eicosanoyl-EA; C22 H45 N O2; 105.680	**Eicosanoyl-EA**	338.342	105.68	Find by Molecular Feature	355.3453

Compound Chromatograms

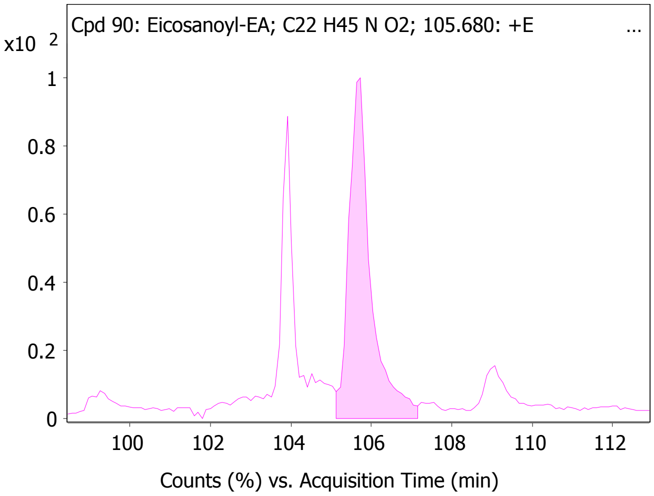

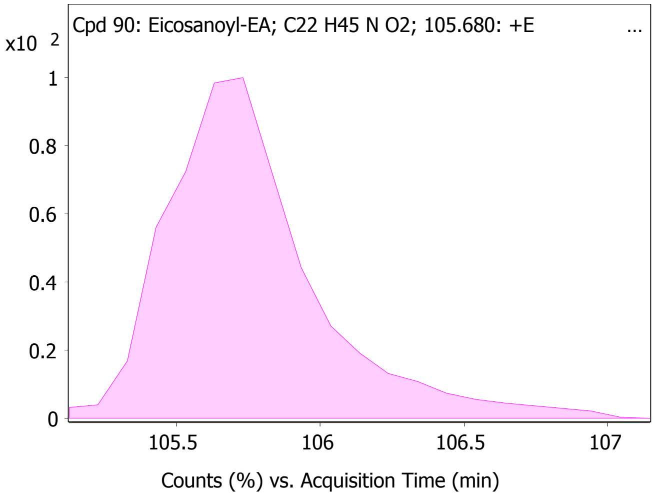

MFE MS Spectrum

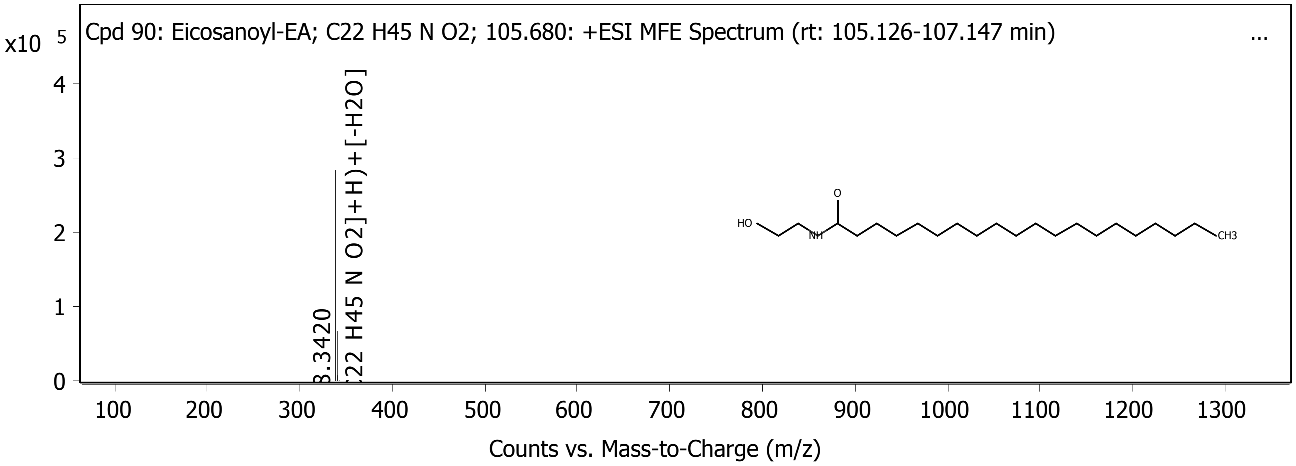

MFE MS Zoomed Spectrum

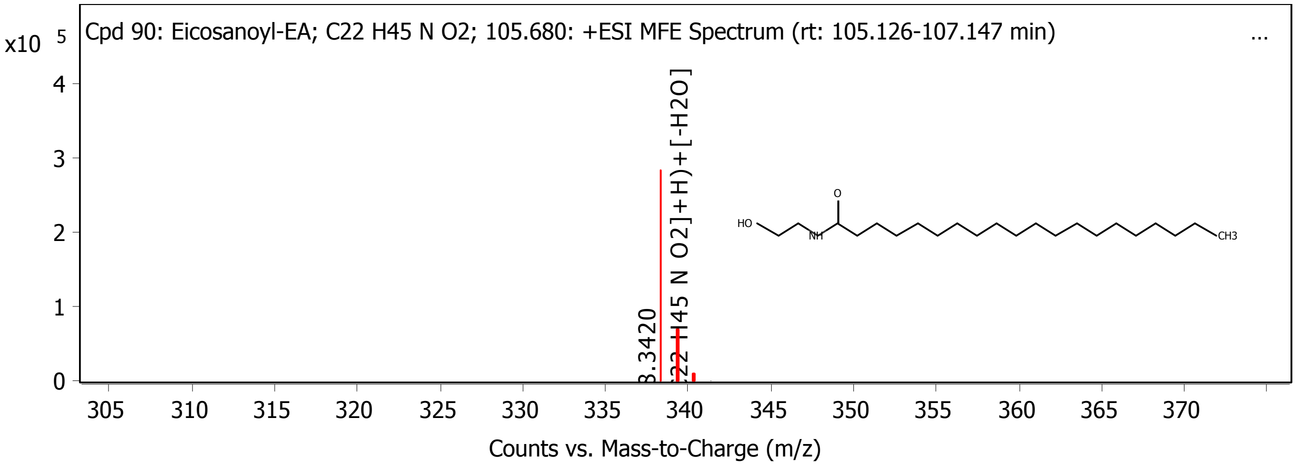

MS Spectrum

Qualitative Compound Identification Report

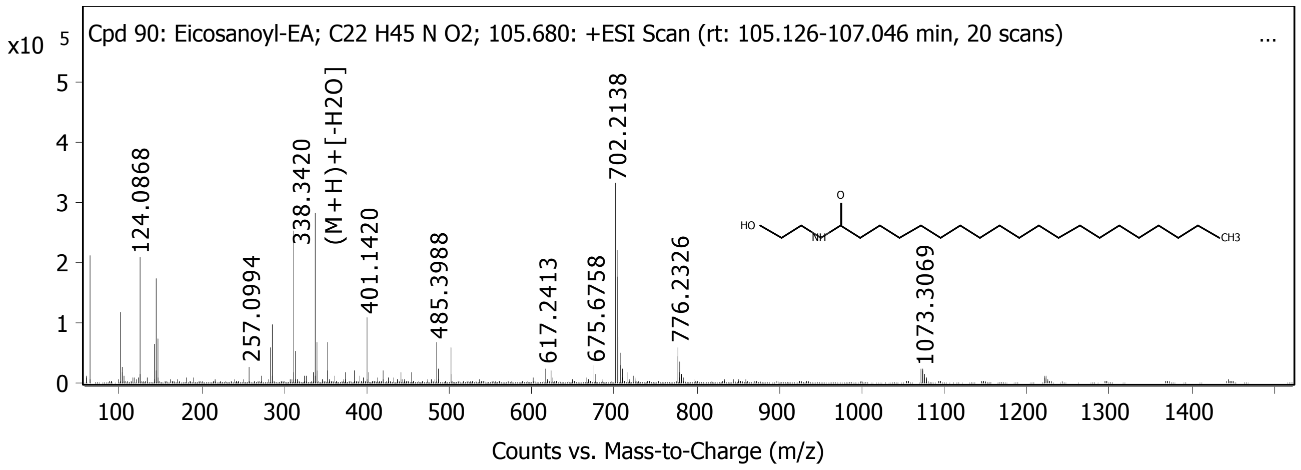

MS Zoomed Spectrum

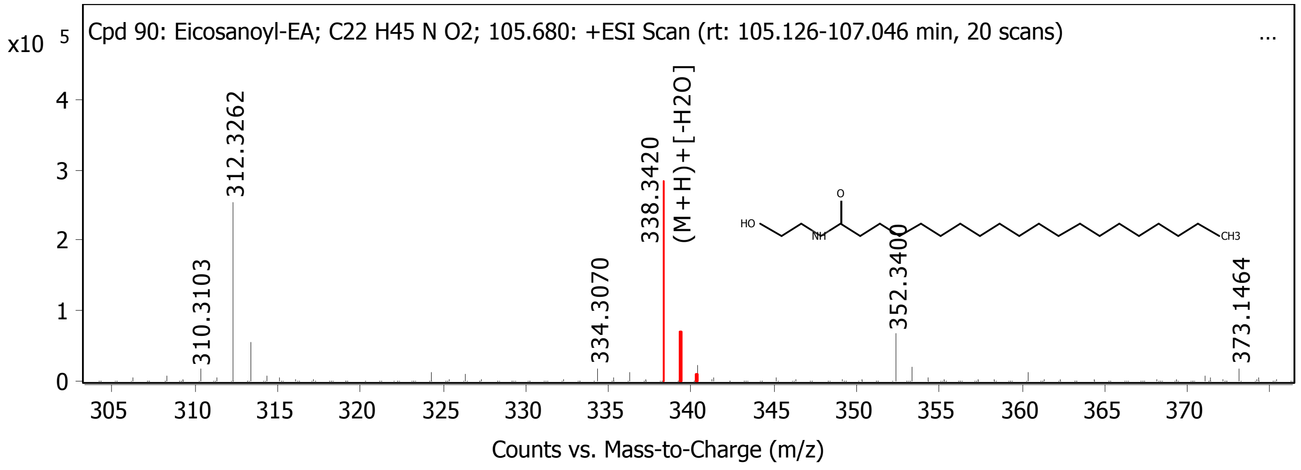

Library Spectrum

Difference Spectrum

MSMS Spectrum

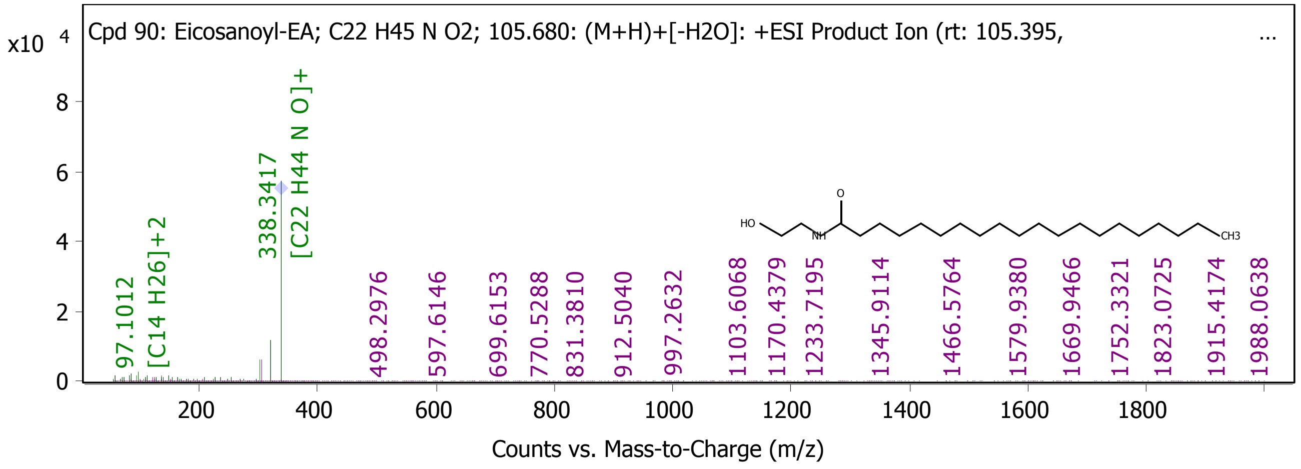

Identification Hit Table

Best Hit	Compound Name	RT	Formula	Notes	Match Score	Mass	Difference	Ion Species
✓	Eicosanoyl-EA	105.68	C22 H45 N O2	20:0 N-acyl ethanolamine http://dx.doi.org/10.1021/bi0480335	99.56	355.3453	-0.28	(M+H)+[-H2O]
	C22:1n-11	105.68	C22 H42 O2		99.55	338.3188	-0.28	(M+NH4)+[-H2O]
	22-Docosanolide	105.68	C22 H42 O2		99.55	338.3188	-0.28	(M+NH4)+[-H2O]
	15Z-docosenoic acid	105.68	C22 H42 O2		99.55	338.3188	-0.28	(M+NH4)+[-H2O]
	cis-Cetoleic acid	105.68	C22 H42 O2	Biological Data 2nd Ed. Vol. 3 p. 1815 Altman, P., Dittmer, D.	99.55	338.3188	-0.28	(M+NH4)+[-H2O]
	13(Z)-Docosenoic acid	105.68	C22 H42 O2	Negative MS/MS	99.55	338.3188	-0.28	(M+NH4)+[-H2O]

Qualitative Compound Identification Report

	trans-Brassidic acid	105.68	C22 H42 O2		99.55	338.3188	-0.28	(M+NH4)+[-H2O]
	11-docosenoic acid	105.68	C22 H42 O2		99.55	338.3188	-0.28	(M+NH4)+[-H2O]
	19Z-docosenoic acid	105.68	C22 H42 O2		99.55	338.3188	-0.28	(M+NH4)+[-H2O]
	5Z-docosenoic acid	105.68	C22 H42 O2		99.55	338.3188	-0.28	(M+NH4)+[-H2O]

Identification Hit Table

Best Hit	Compound Name	RT	Formula	Notes	Match Score	Mass	Difference	Ion Species
✓	Eicosanoyl-EA	105.68	C22 H45 N O2	20:0 N-acyl ethanolamine http://dx.doi.org/10.1021/bi0480335	99.56	355.3453	-0.28	(M+H)+[-H2O]
	cis-Cetoleic acid	105.68	C22 H42 O2	Biological Data 2nd Ed. Vol. 3 p. 1815 Altman, P., Dittmer, D.	99.55	338.3188	-0.28	(M+NH4)+[-H2O]
	22-Docosanolide	105.68	C22 H42 O2		99.55	338.3188	-0.28	(M+NH4)+[-H2O]
	7Z-Octadecenyl isobutyrate	105.68	C22 H42 O2		99.55	338.3188	-0.28	(M+NH4)+[-H2O]
	16-Methyl-9Z-heptadecenyl isobutyrate	105.68	C22 H42 O2		99.55	338.3188	-0.28	(M+NH4)+[-H2O]
	(Z)-3-Hexenyl hexadecanoate	105.68	C22 H42 O2		99.55	338.3188	-0.28	(M+NH4)+[-H2O]
	(Z)-7-Octadecenyl butyrate	105.68	C22 H42 O2		99.55	338.3188	-0.28	(M+NH4)+[-H2O]
	11Z-Eicosenyl acetate	105.68	C22 H42 O2		99.55	338.3188	-0.28	(M+NH4)+[-H2O]
	22:1(9Z)	105.68	C22 H42 O2		99.55	338.3188	-0.28	(M+NH4)+[-H2O]
	2,4-dimethyl-2-eicosenoic acid	105.68	C22 H42 O2		99.55	338.3188	-0.28	(M+NH4)+[-H2O]

Identification Hit Table

Best Hit	Compound Name	RT	Formula	Notes	Match Score	Mass	Difference	Ion Species

Compound Label	Name	*m/z*	RT	Algorithm	Mass
Cpd 91: N,N-dimethyl-Safingol; C20 H43 N O2; 106.427	**N,N-dimethyl-Safingol**	312.3262	106.427	Find by Molecular Feature	329.3295

Compound Chromatograms

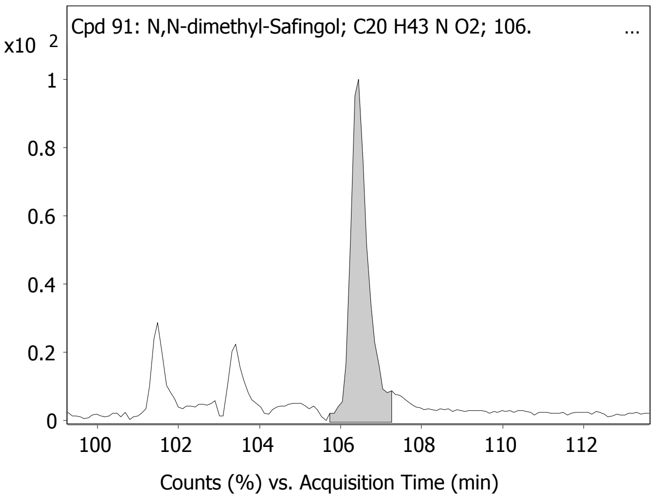

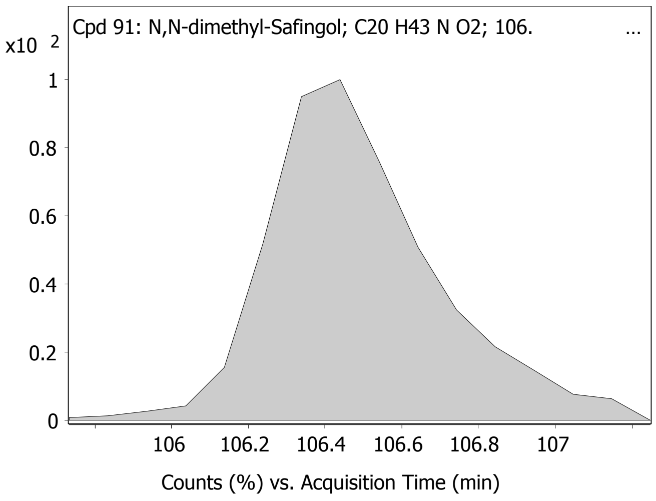

MFE MS Spectrum

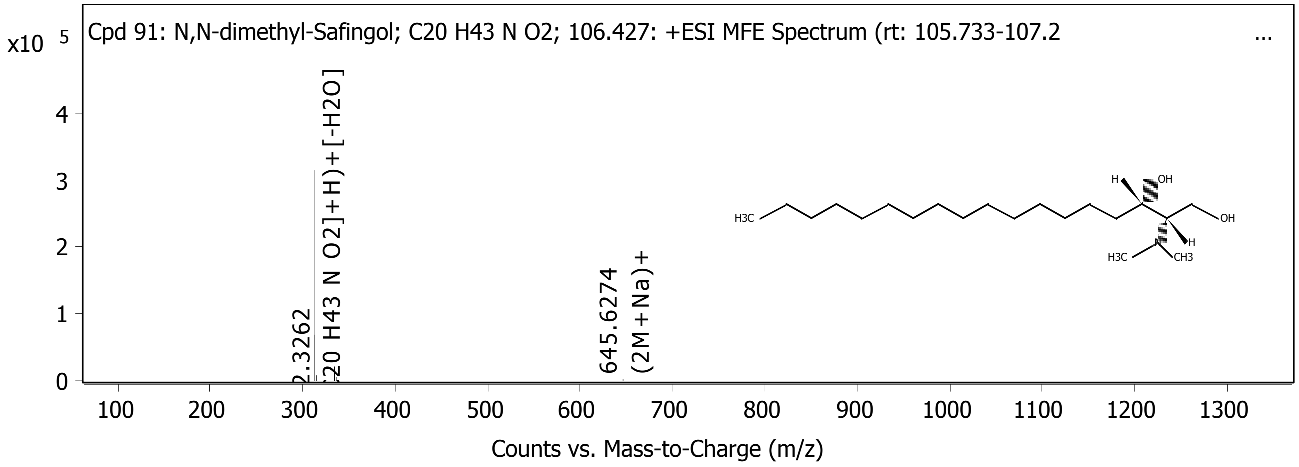

MFE MS Zoomed Spectrum

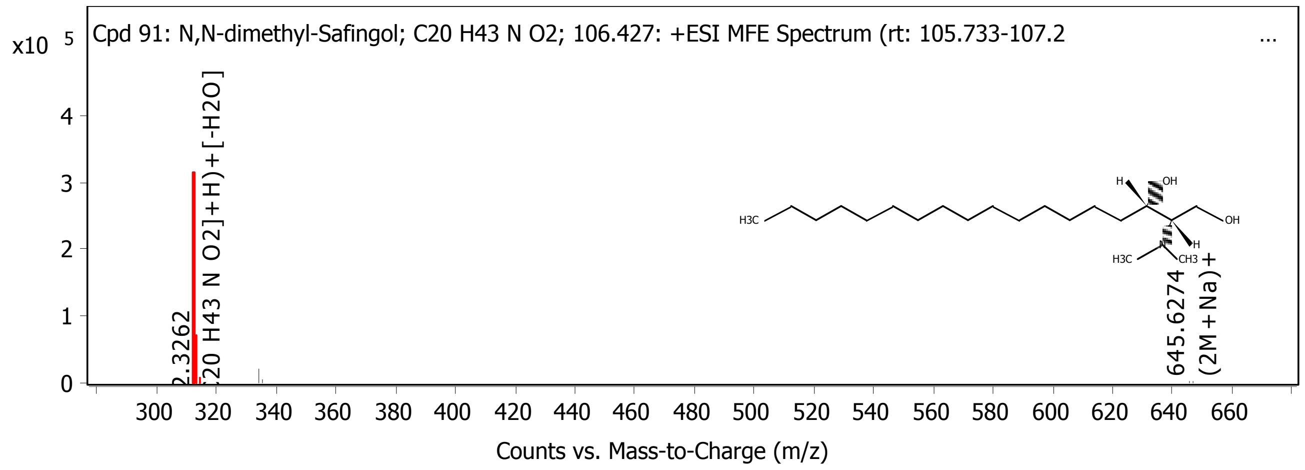

MS Spectrum

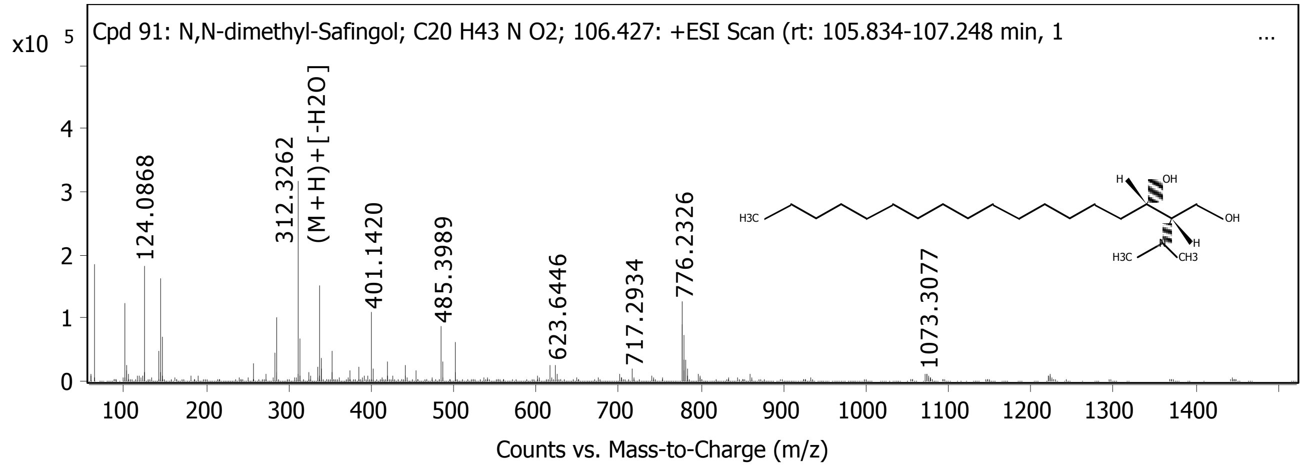

MS Zoomed Spectrum

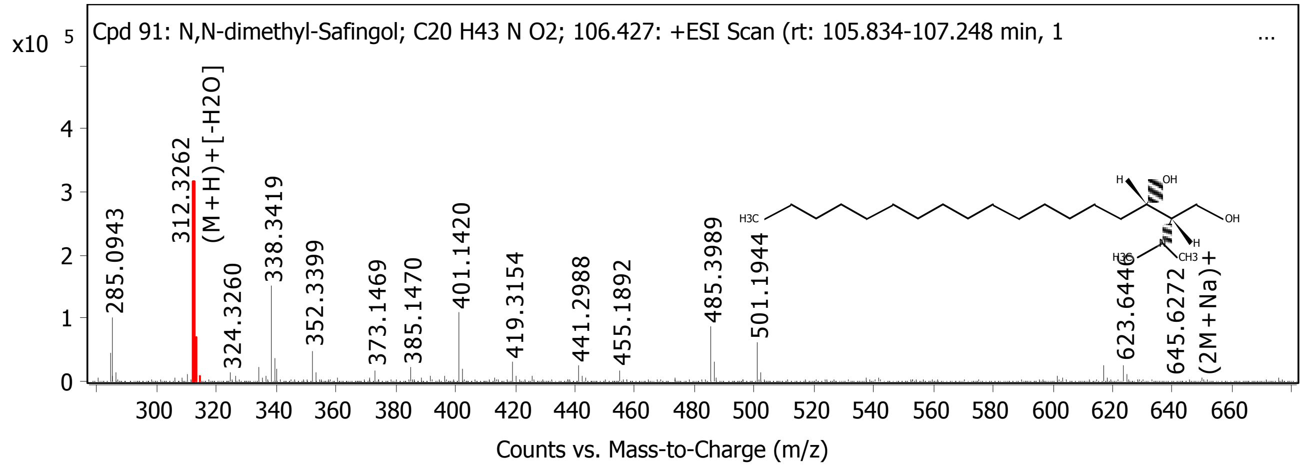

MSMS Spectrum

Qualitative Compound Identification Report

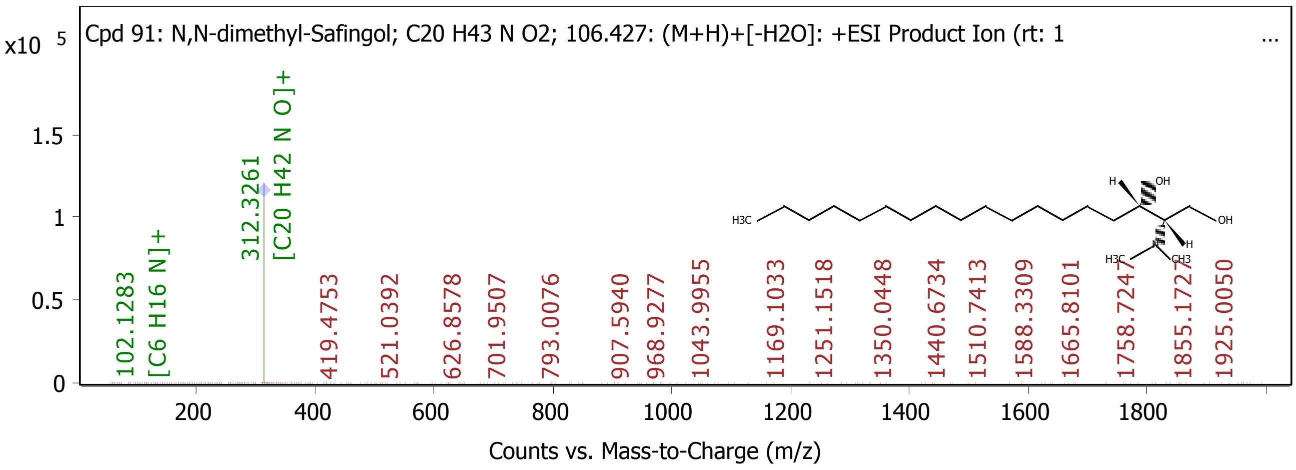

Identification Hit Table

Best Hit	Compound Name	RT	Formula	Notes	Match Score	Mass	Difference	Ion Species
✓	N,N-dimethyl-Safingol	106.427	C20 H43 N O2		99.77	329.3295	-0.09	(M+H)+[-H2O]
	(±)-CP 47,497-d11	106.427	C21 H23 D11 O2		63.81	329.3295	-4.53	(M+H)+[-H2O]

Identification Hit Table

Best Hit	Compound Name	RT	Formula	Notes	Match Score	Mass	Difference	Ion Species
✓	N,N-dimethyl-Safingol	106.427	C20 H43 N O2		99.77	329.3295	-0.09	(M+H)+[-H2O]

Identification Hit Table

Best Hit	Compound Name	RT	Formula	Notes	Match Score	Mass	Difference	Ion Species

Compound Label	Name	*m/z*	RT	Algorithm	Mass
Cpd 92: Hyaluronan; C28 H44 N2 O23; 108.421	**Hyaluronan**	776.2327	108.421	Find by Molecular Feature	776.2295

Compound Chromatograms

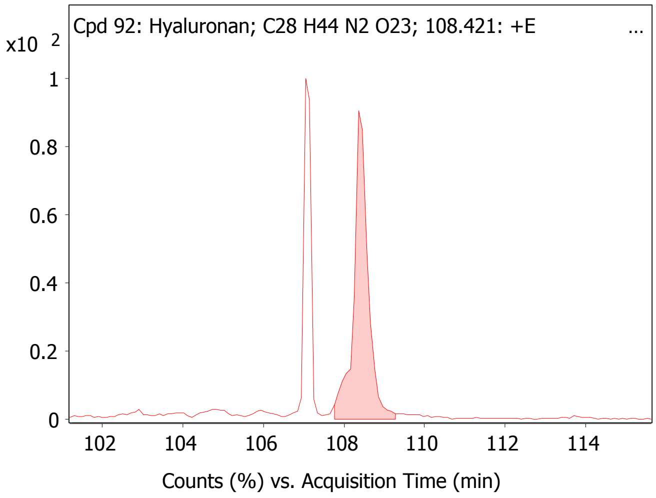

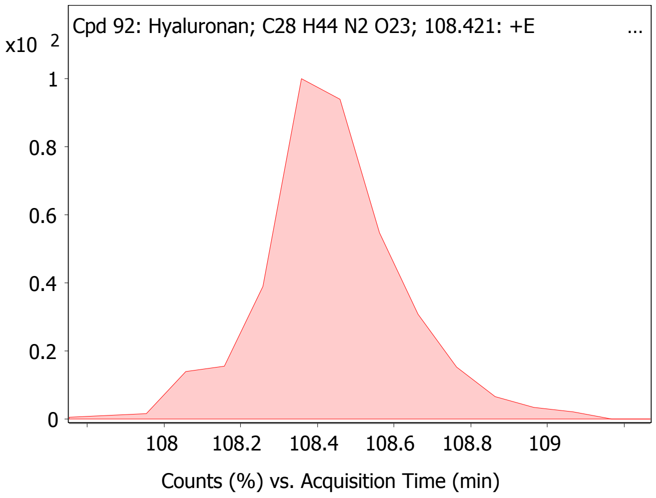

MFE MS Spectrum

Qualitative Compound Identification Report

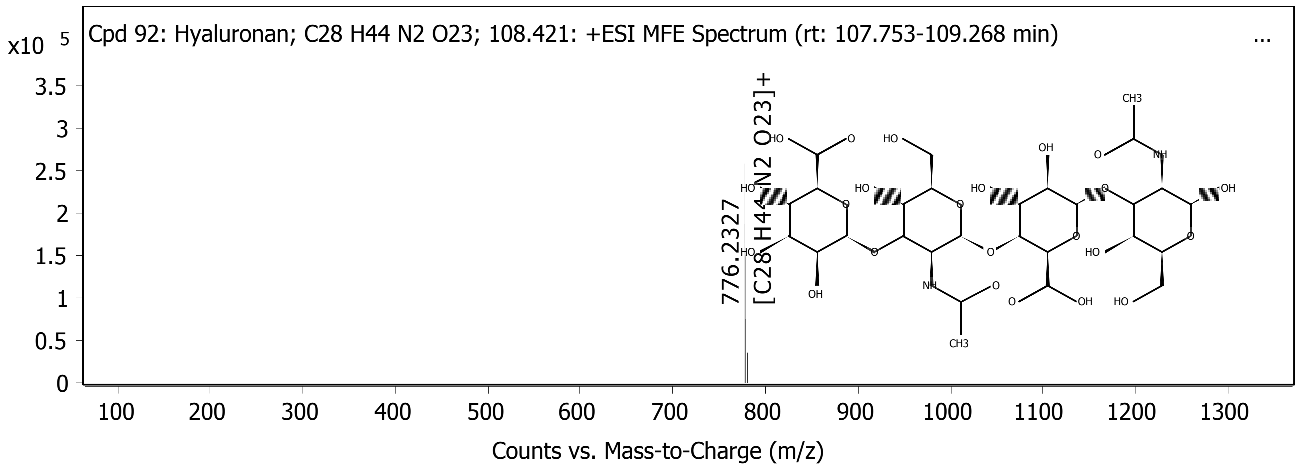

MFE MS Zoomed Spectrum

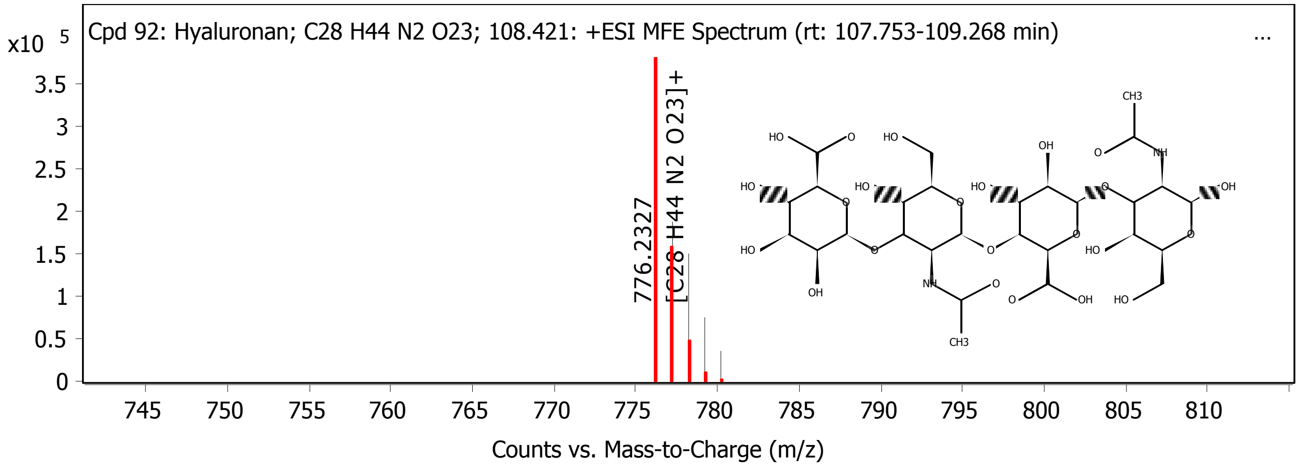

MS Spectrum

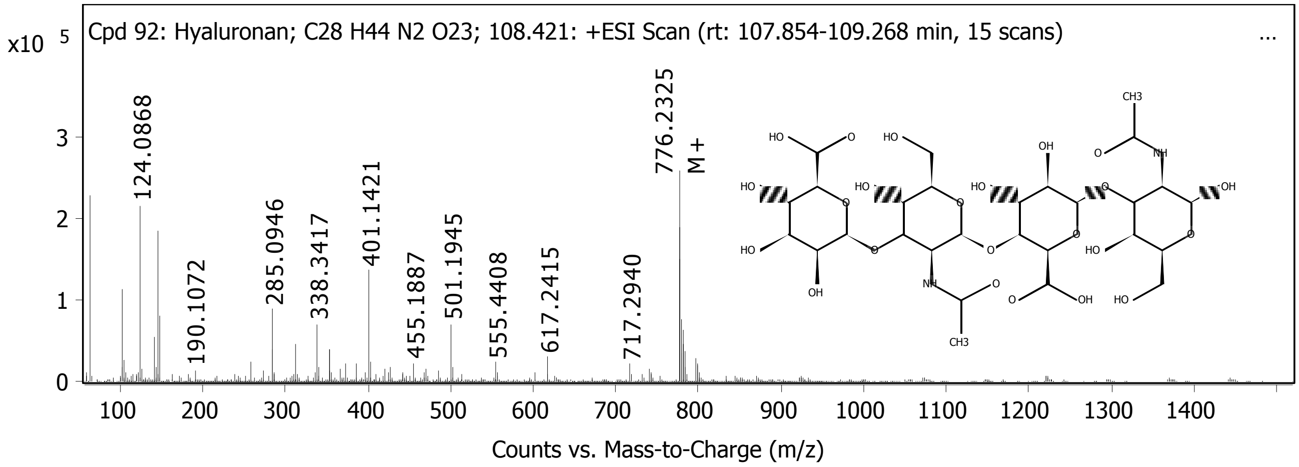

MS Zoomed Spectrum

Agilent Technologies

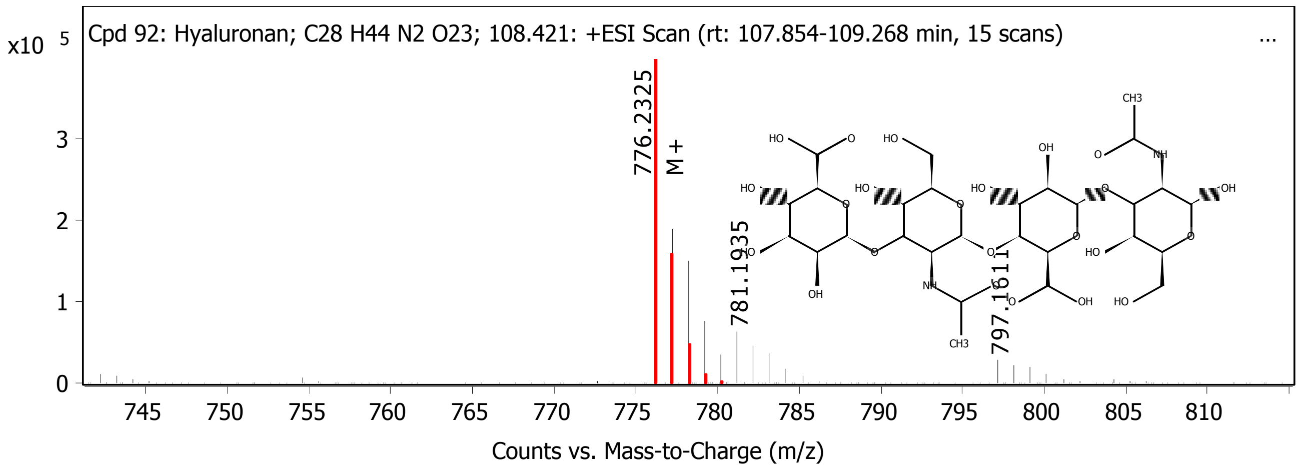

MSMS Spectrum

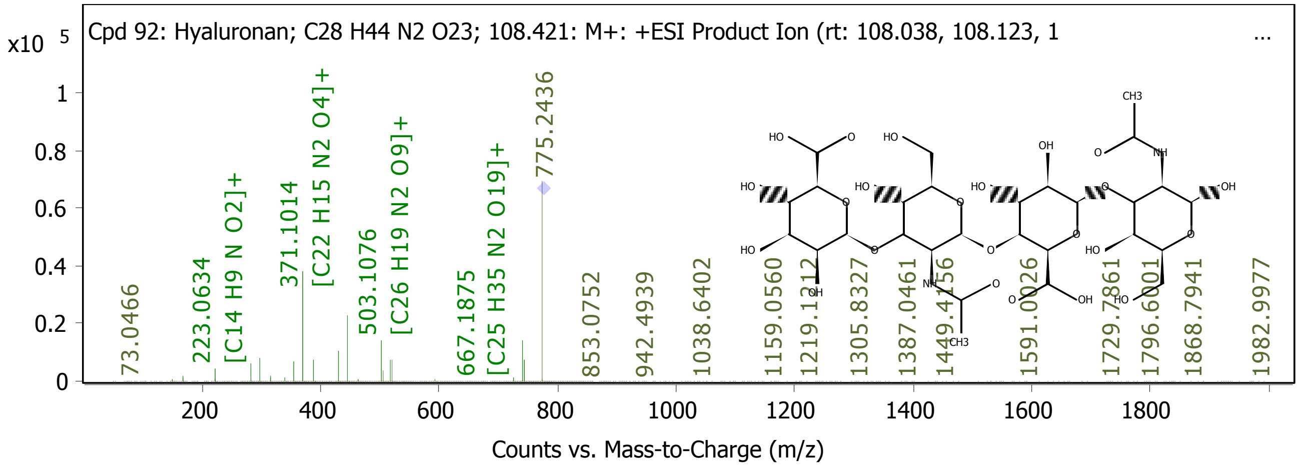

Identification Hit Table

Best Hit	Compound Name	RT	Formula	Notes	Match Score	Mass	Difference	Ion Species
✓	Hyaluronan	108.421	C28 H44 N2 O23		47.89	776.2295	3.94	M+
	3-[(2S,3R,4S,5R,6R)-4-[(2S,3R,4S,5S,6R)-4,5-Dihydroxy-6-(hydroxymethyl)-3-[(2S,3R,4S,5R)-3,4,5-trihydroxyoxan-2-yl]oxyoxan-2-yl]oxy-3,5-dihydroxy-6-(hydroxymethyl)oxan-2-yl]oxy-2-(3,4-dihydroxyphenyl)-5,7-dihydroxychromen-4-one	108.421	C32 H38 O21	MolPort Compound number: MolPort-001-741-670	43.51	758.1951	-4.49	(M+NH4)+
	Quercetin 3-sambubioside-3'-glucoside	108.421	C32 H38 O21		43.51	758.1951	-4.49	(M+NH4)+
	Quercetin 3-sambubioside-7-glucoside	108.421	C32 H38 O21		43.51	758.1951	-4.49	(M+NH4)+
	Isoorientin 3',6''-di-O-glucoside	108.421	C32 H38 O21		43.51	758.1951	-4.49	(M+NH4)+

Identification Hit Table

Best Hit	Compound Name	RT	Formula	Notes	Match Score	Mass	Difference	Ion Species
✓	Isoorientin 3',6''-di-O-glucoside	108.421	C32 H38 O21		43.51	758.1951	-4.49	(M+NH4)+
	Quercetin 3-sambubioside-7-glucoside	108.421	C32 H38 O21		43.51	758.1951	-4.49	(M+NH4)+
	Quercetin 3-sambubioside-3'-glucoside	108.421	C32 H38 O21		43.51	758.1951	-4.49	(M+NH4)+

Identification Hit Table

Best Hit	Compound Name	RT	Formula	Notes	Match Score	Mass	Difference	Ion Species

Compound Label	Name	*m/z*	RT	Algorithm	Mass
Cpd 93: Quercetin 3-(6'''-sinapylglucosyl)(1->2)-galactoside; C38 H40 O21; 112.775	**Quercetin 3-(6'''-sinapylglucosyl)(1->2)-galactoside**	850.2512	112.775	Find by Molecular Feature	832.2132

Compound Chromatograms

Agilent Technologies

Qualitative Compound Identification Report

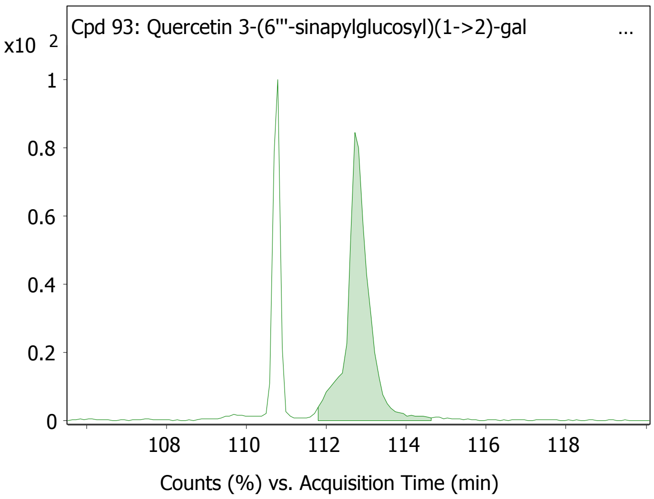

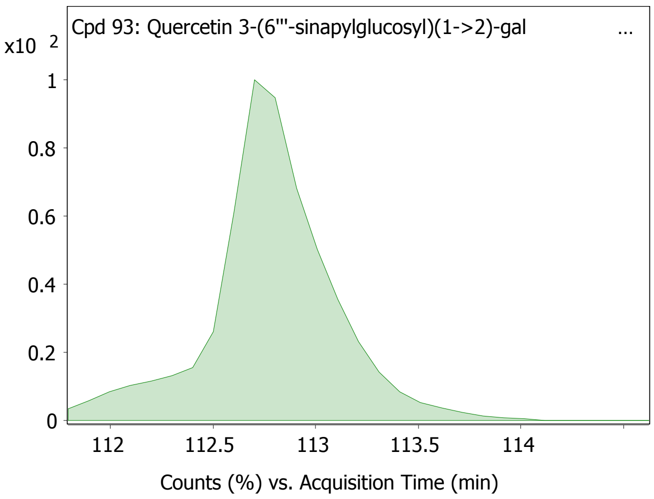

MFE MS Spectrum

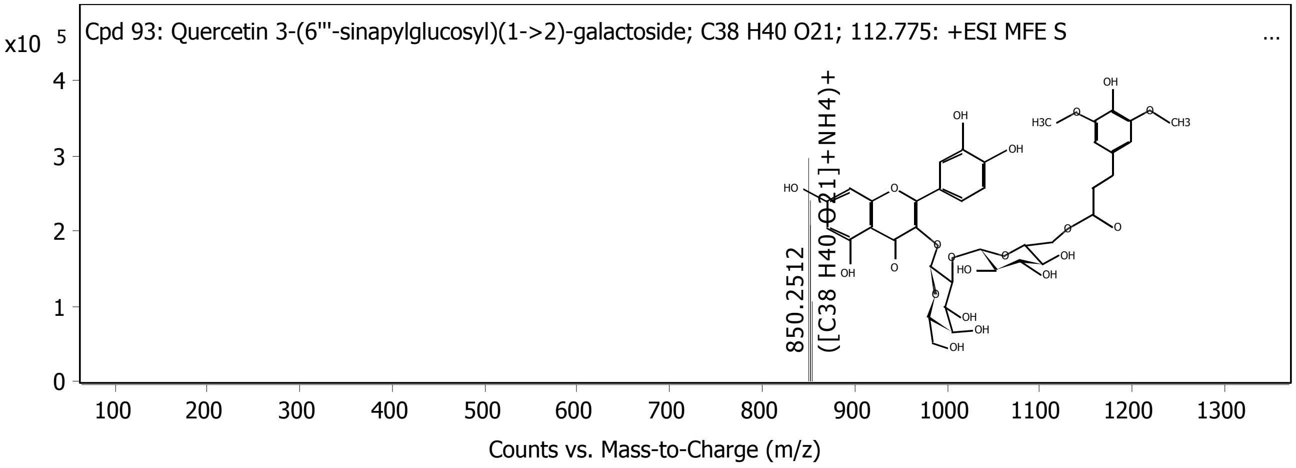

MFE MS Zoomed Spectrum

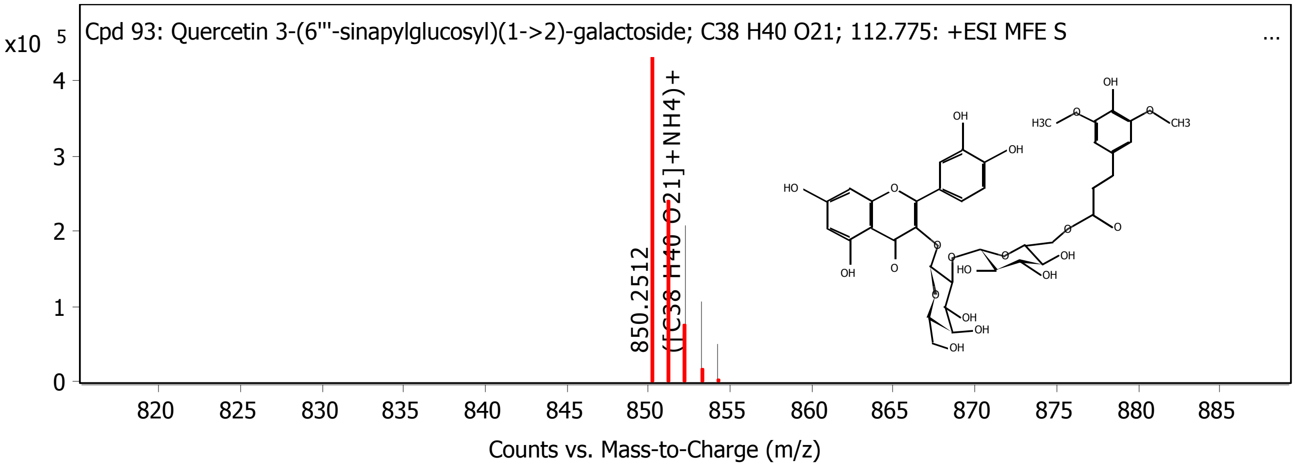

MS Spectrum

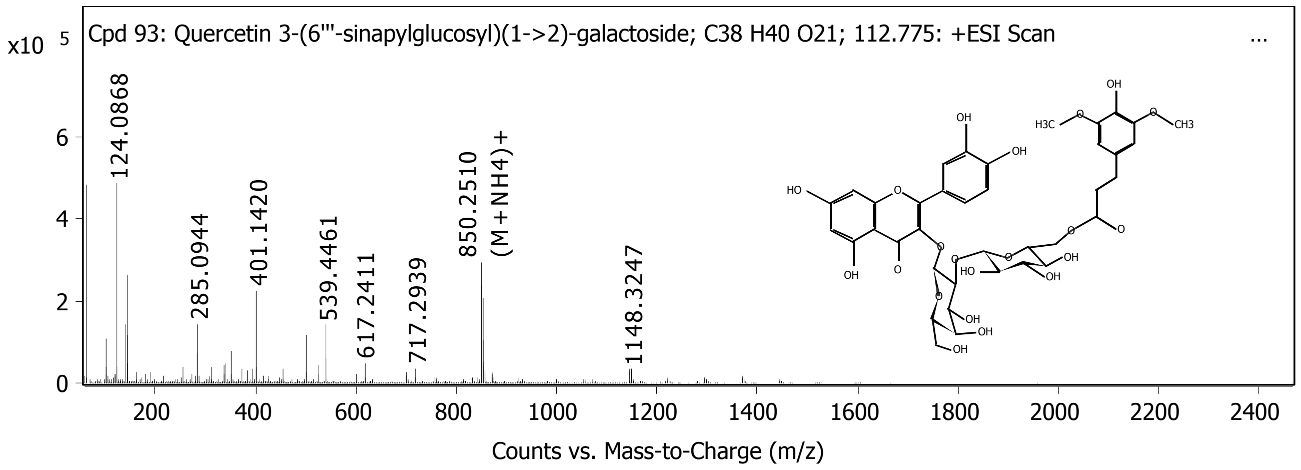

MS Zoomed Spectrum

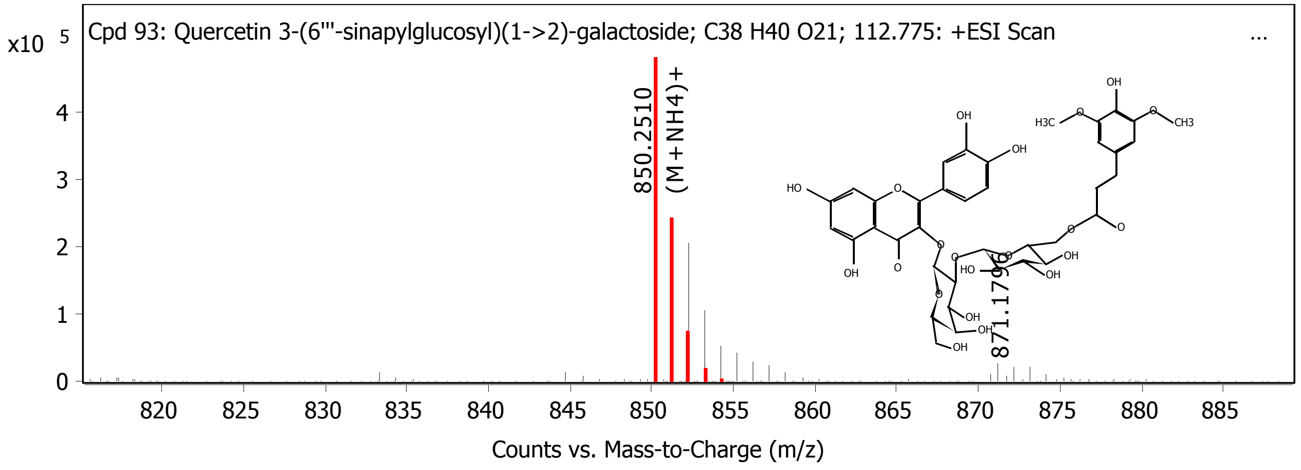

MSMS Spectrum

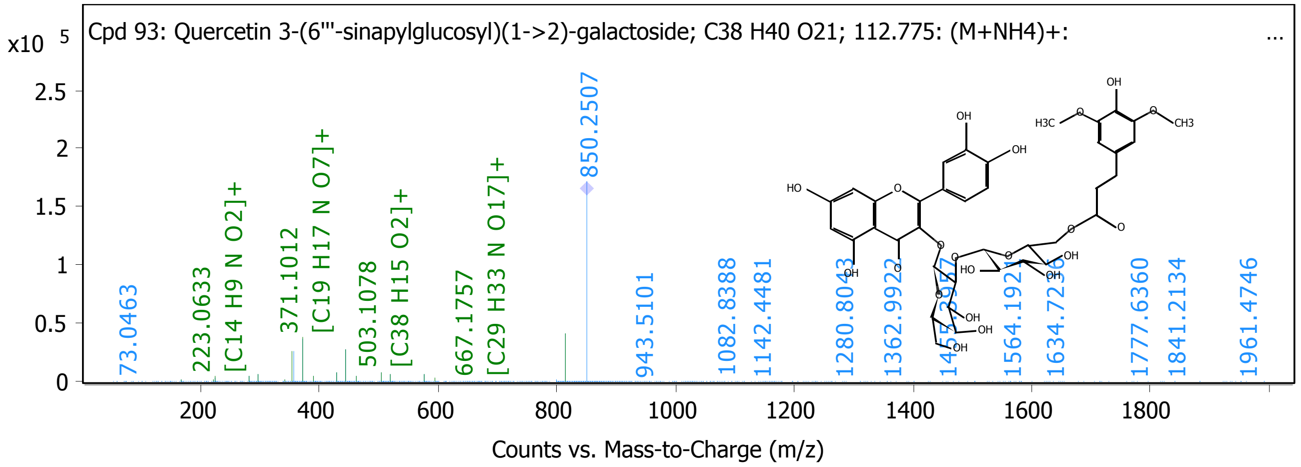

Identification Hit Table

Best Hit	Compound Name	RT	Formula	Notes	Match Score	Mass	Difference	Ion Species
✓	Quercetin 3-(6'''-sinapylglucosyl)(1->2)-galactoside	112.775	C38 H40 O21		31.56	832.2132	-6.99	(M+NH4)+

Identification Hit Table

Best Hit	Compound Name	RT	Formula	Notes	Match Score	Mass	Difference	Ion Species
✓	Quercetin 3-(6'''-sinapylglucosyl)(1->2)-galactoside	112.775	C38 H40 O21		31.56	832.2132	-6.99	(M+NH4)+

Identification Hit Table

Best Hit	Compound Name	RT	Formula	Notes	Match Score	Mass	Difference	Ion Species

Qualitative Compound Identification Report

Compound Label	*m/z*	RT	Algorithm	Mass
Cpd 94: 116.022	924.2706	116.022	Find by Molecular Feature	923.2633

Compound Chromatograms

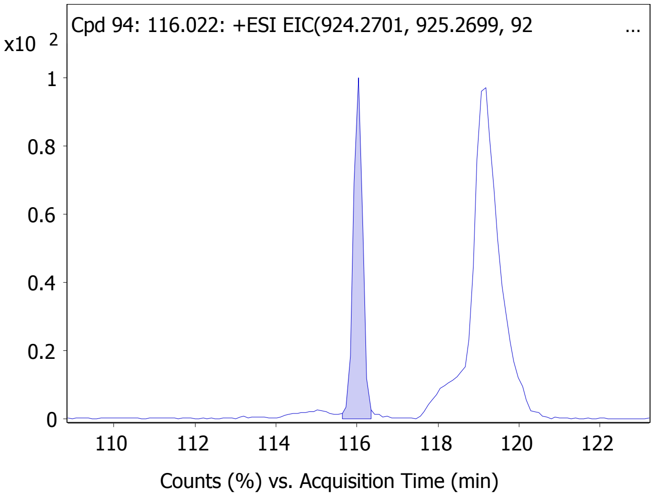

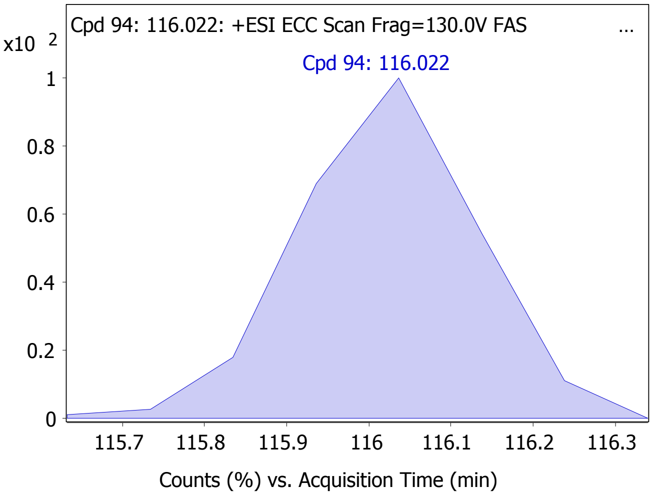

MFE MS Spectrum

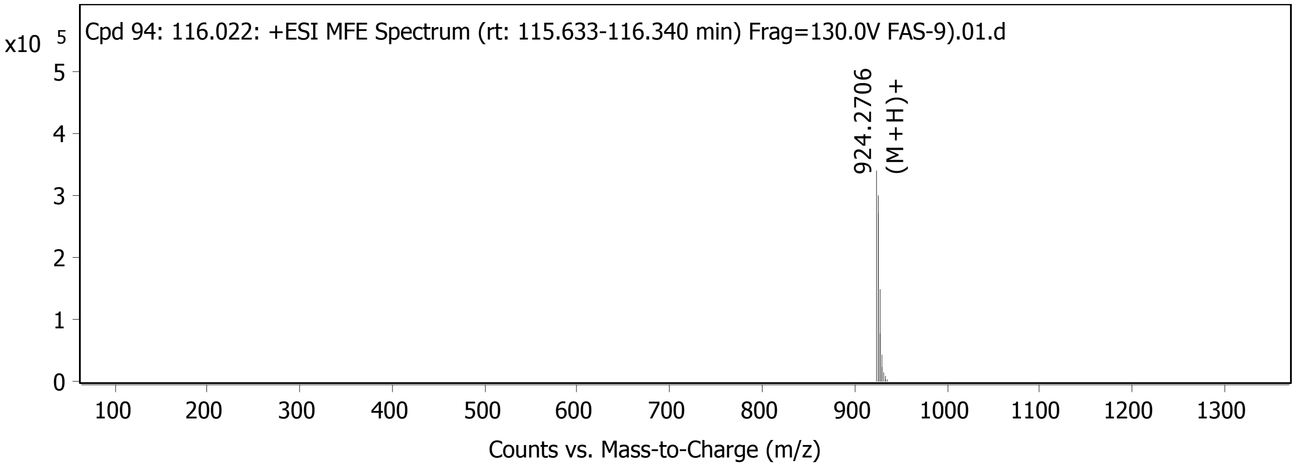

MFE MS Zoomed Spectrum

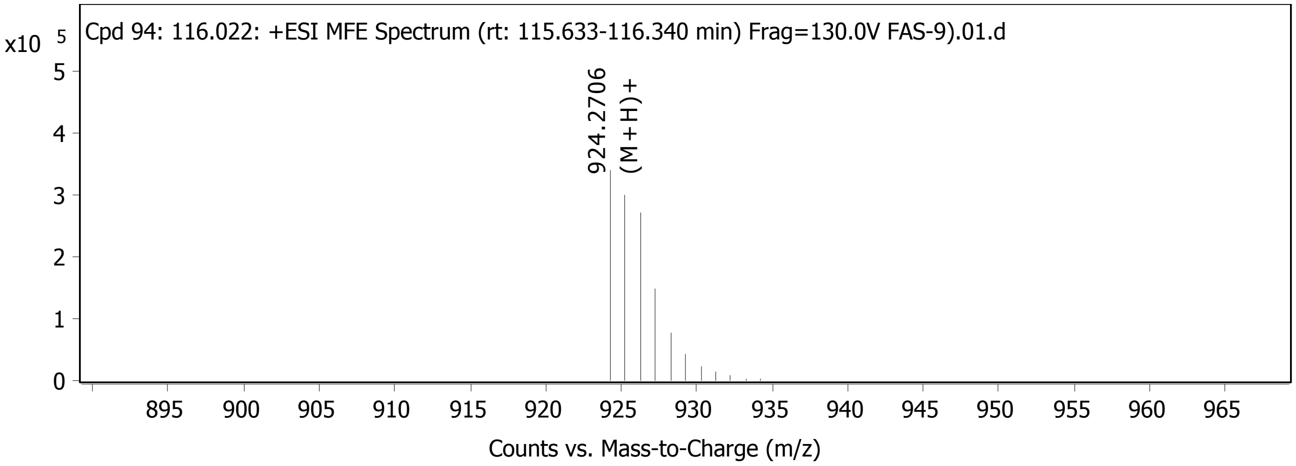

MS Spectrum

Qualitative Compound Identification Report

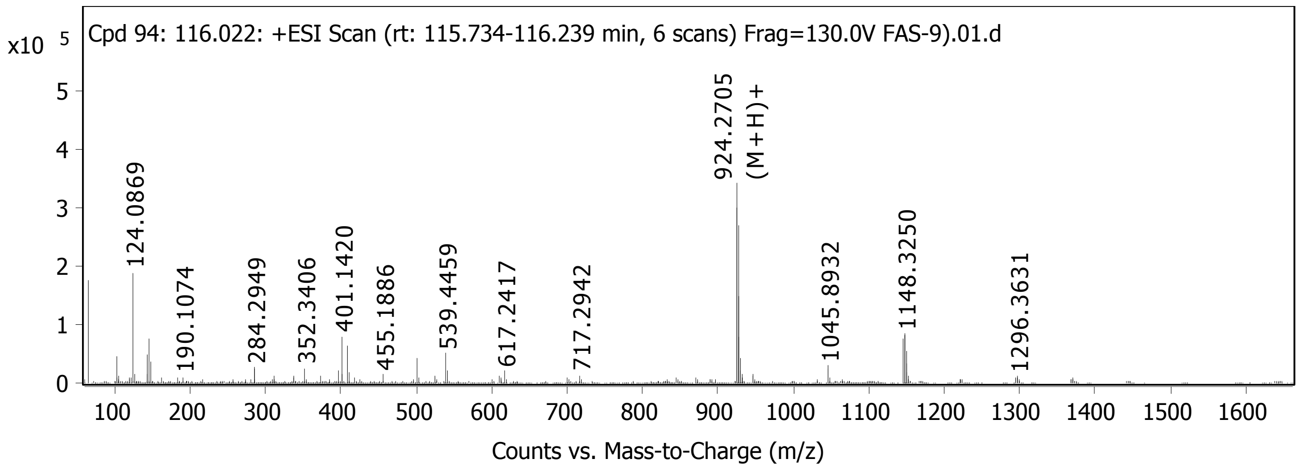

MS Zoomed Spectrum

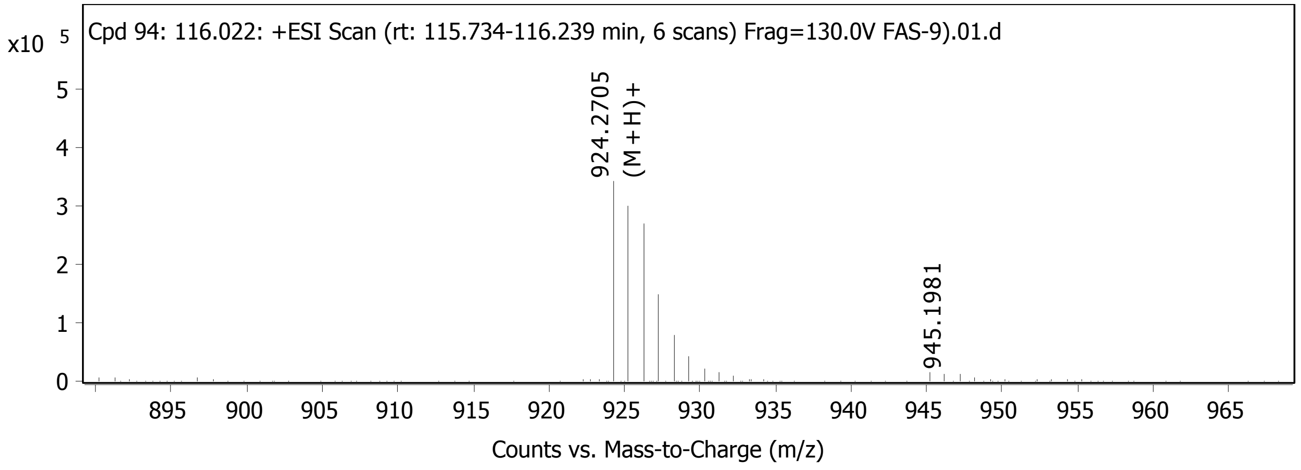

Compound Label	*m/z*	RT	Algorithm	Mass
Cpd 95: 119.170	924.2706	119.17	Find by Molecular Feature	923.2633

Compound Chromatograms

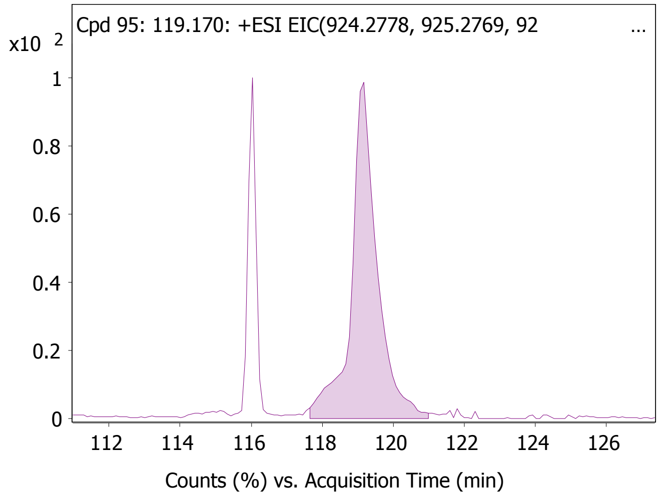

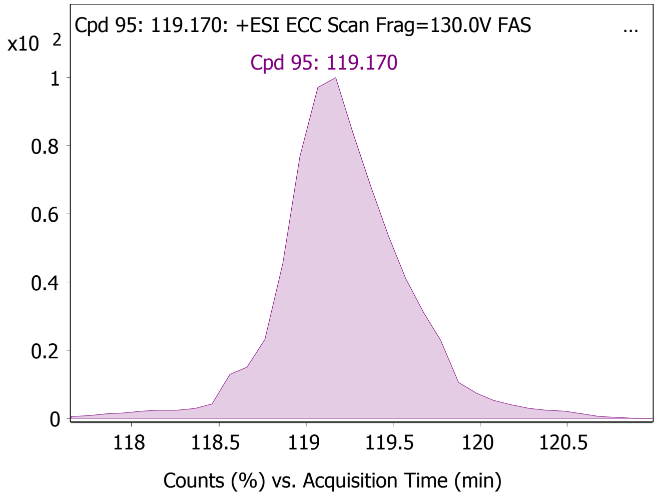

MFE MS Spectrum

Qualitative Compound Identification Report

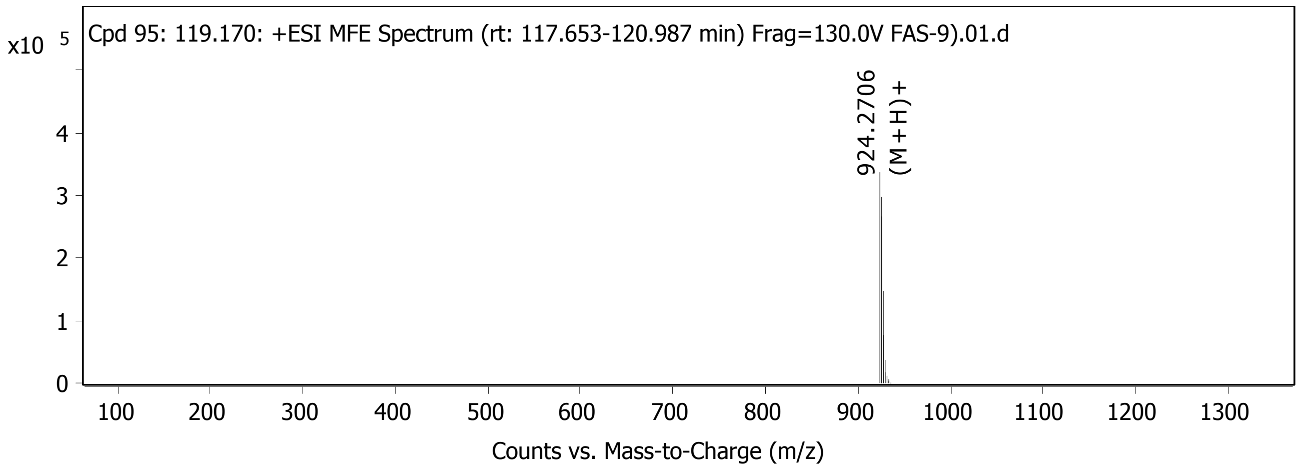

MFE MS Zoomed Spectrum

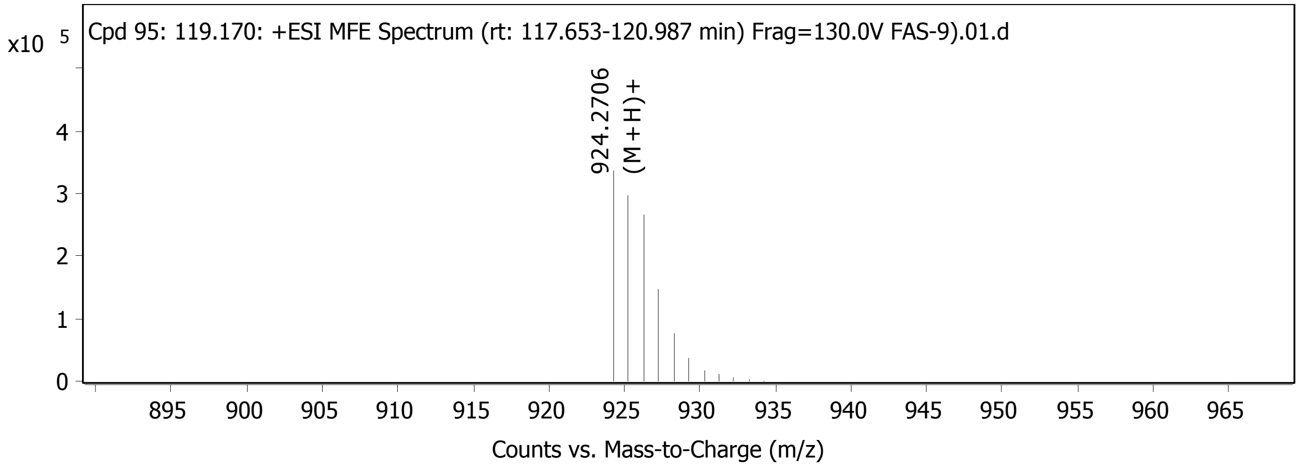

MS Spectrum

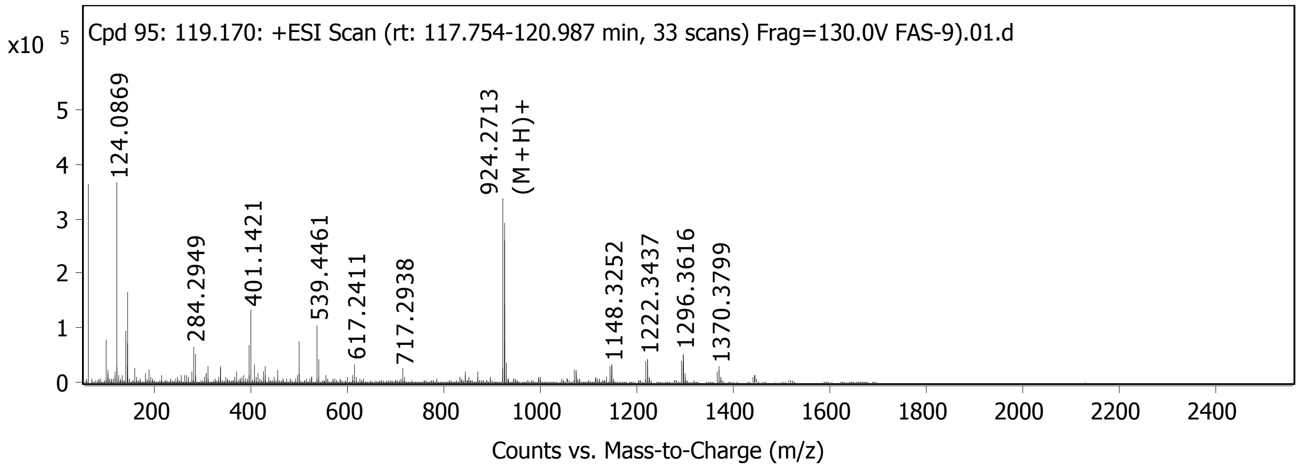

MS Zoomed Spectrum

Qualitative Compound Identification Report

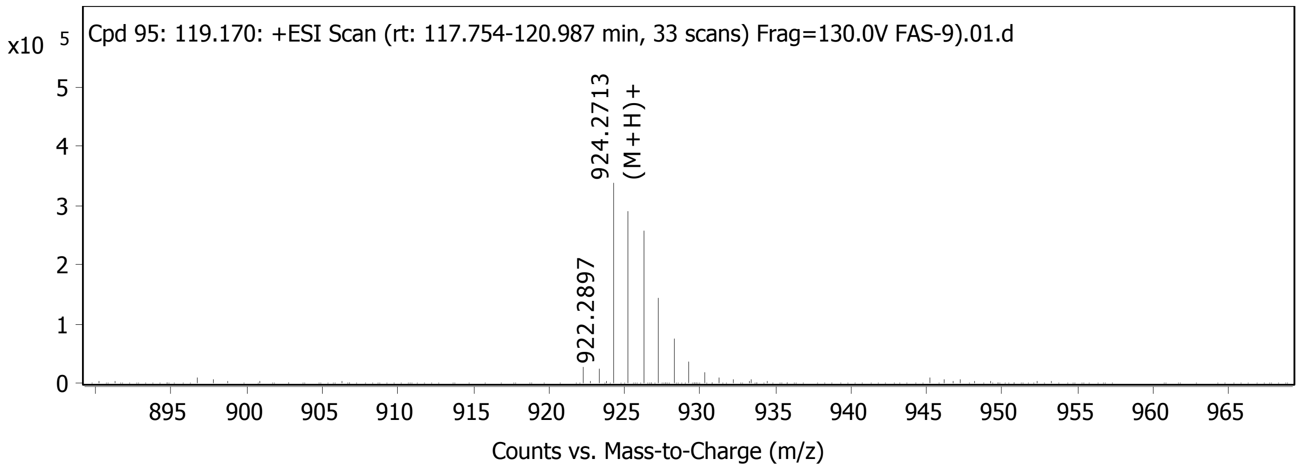

Compound Label	Name	*m/z*	RT	Algorithm	Mass
Cpd 96: (S)-3-Hydroxytetradecanoyl-CoA; C35 H62 N7 O18 P3 S; 123.955	**(S)-3-Hydroxytetradecanoyl-CoA**	998.2889	123.955	Find by Molecular Feature	993.3061

Compound Chromatograms

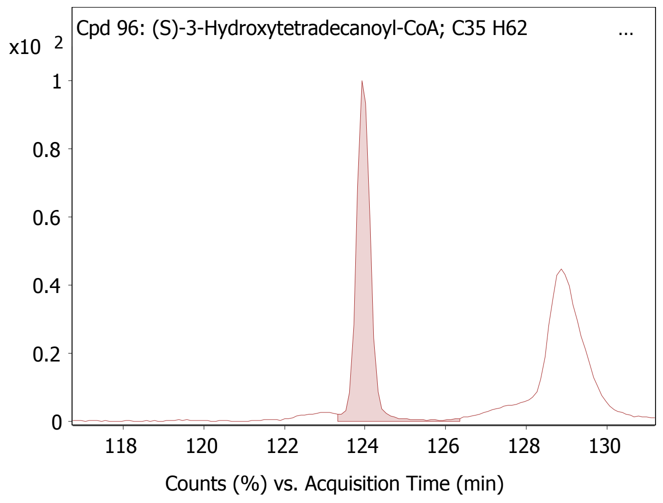

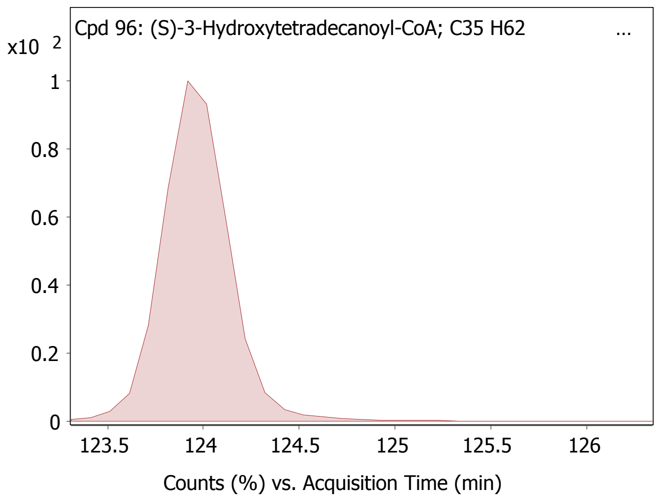

MFE MS Spectrum

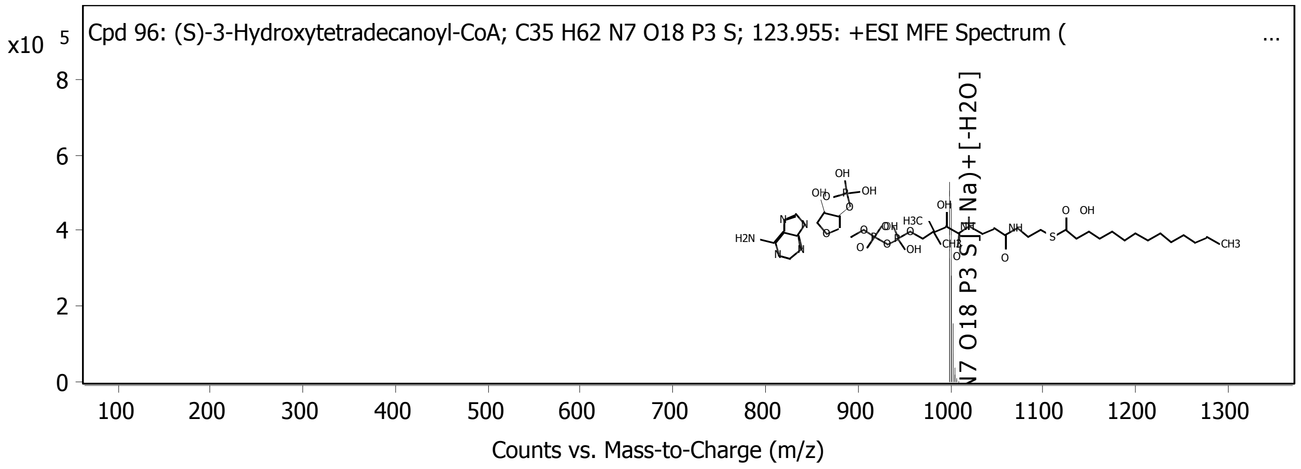

MFE MS Zoomed Spectrum

Qualitative Compound Identification Report

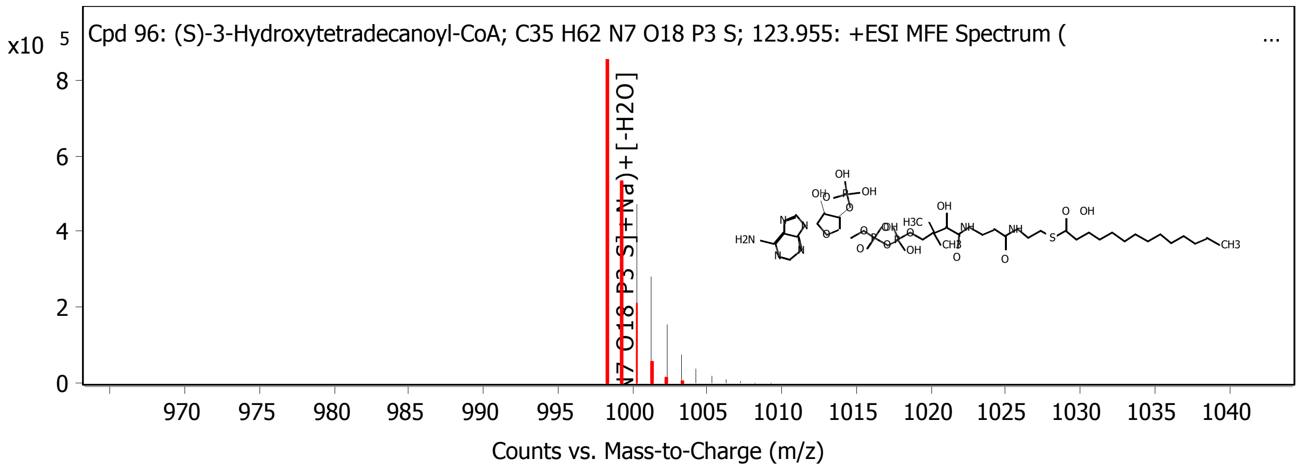

MS Spectrum

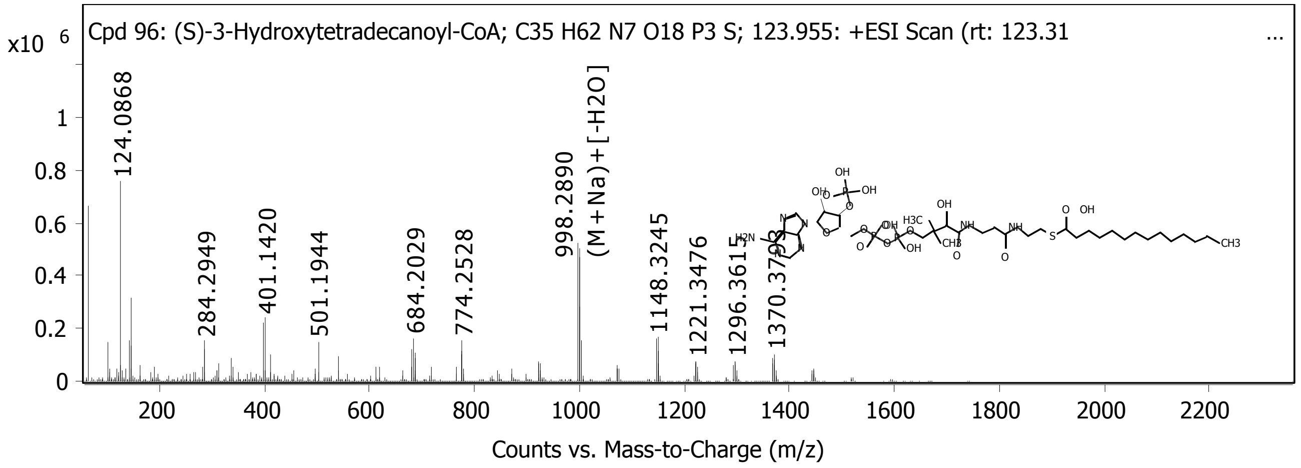

MS Zoomed Spectrum

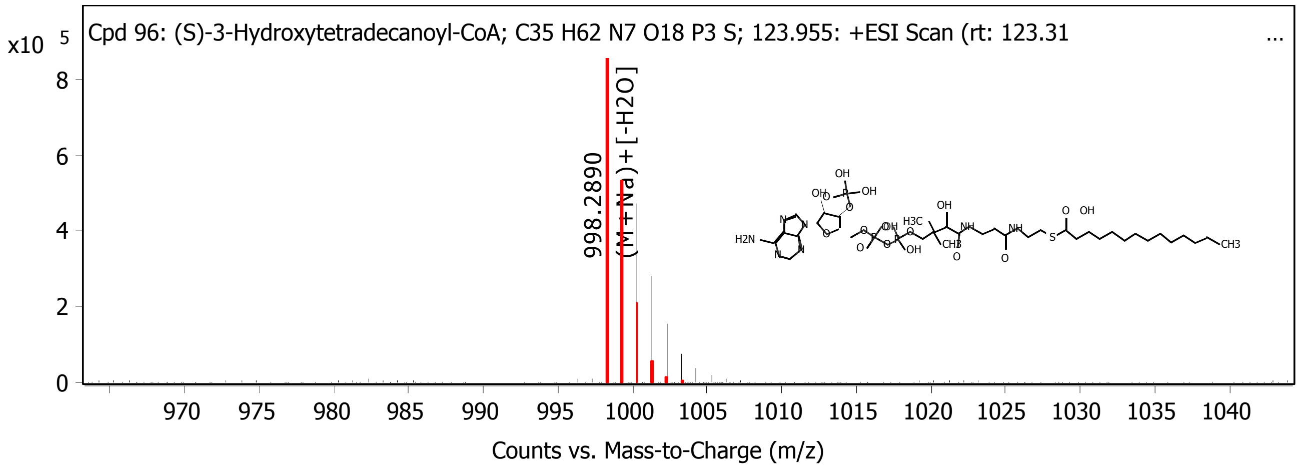

MSMS Spectrum

Qualitative Compound Identification Report

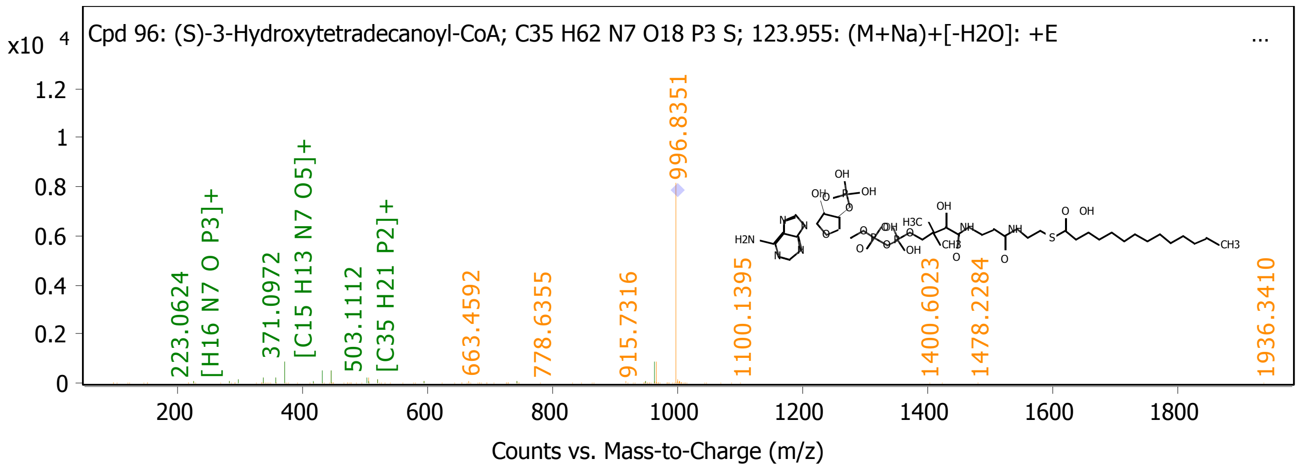

Identification Hit Table

Best Hit	Compound Name	RT	Formula	Notes	Match Score	Mass	Difference	Ion Species
✓	(S)-3-Hydroxytetradecanoyl-CoA	123.955	C35 H62 N7 O18 P3 S		63.69	993.3061	2.35	(M+Na)+[-H2O]
	3-hydroxymyristoyl-CoA	123.955	C35 H62 N7 O18 P3 S		63.69	993.3061	2.35	(M+Na)+[-H2O]
	11Z-tetradecenoyl-CoA	123.955	C35 H60 N7 O17 P3 S		63.58	975.2956	2.35	(M+Na)+
	11E-tetradecenoyl-CoA	123.955	C35 H60 N7 O17 P3 S		63.58	975.2956	2.35	(M+Na)+
	2E-tetradecenoyl-CoA	123.955	C35 H60 N7 O17 P3 S		63.58	975.2956	2.35	(M+Na)+
	OPC6-CoA	123.955	C37 H60 N7 O18 P3 S		52.28	1015.288	4.85	(M+H)+[-H2O]

Identification Hit Table

Best Hit	Compound Name	RT	Formula	Notes	Match Score	Mass	Difference	Ion Species
✓	3-hydroxymyristoyl-CoA	123.955	C35 H62 N7 O18 P3 S		63.69	993.3061	2.35	(M+Na)+[-H2O]
	2E-tetradecenoyl-CoA	123.955	C35 H60 N7 O17 P3 S		63.58	975.2956	2.35	(M+Na)+
	11E-tetradecenoyl-CoA	123.955	C35 H60 N7 O17 P3 S		63.58	975.2956	2.35	(M+Na)+
	11Z-tetradecenoyl-CoA	123.955	C35 H60 N7 O17 P3 S		63.58	975.2956	2.35	(M+Na)+

Identification Hit Table

Best Hit	Compound Name	RT	Formula	Notes	Match Score	Mass	Difference	Ion Species

Compound Label	Name	*m/z*	RT	Algorithm	Mass
Cpd 97: PG(14:0/15:0); C35 H69 O10 P; 126.955	**PG(14:0/15:0)**	680.481	126.955	Find by Molecular Feature	680.4576

Compound Chromatograms

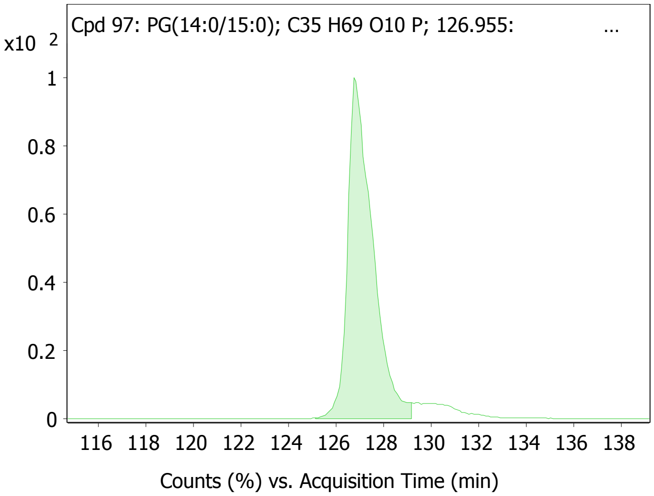

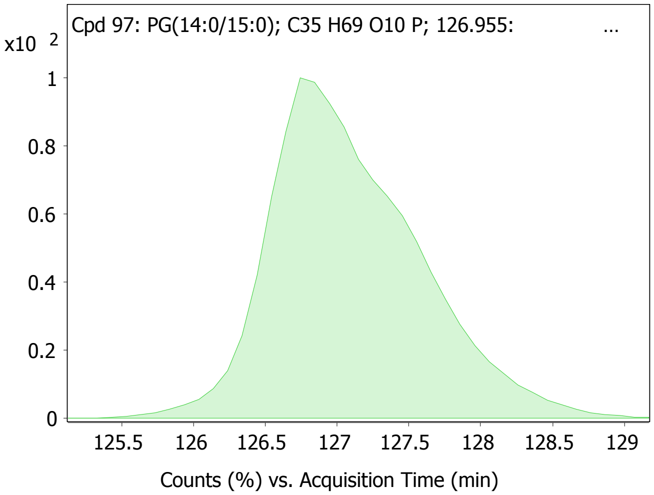

MFE MS Spectrum

Qualitative Compound Identification Report

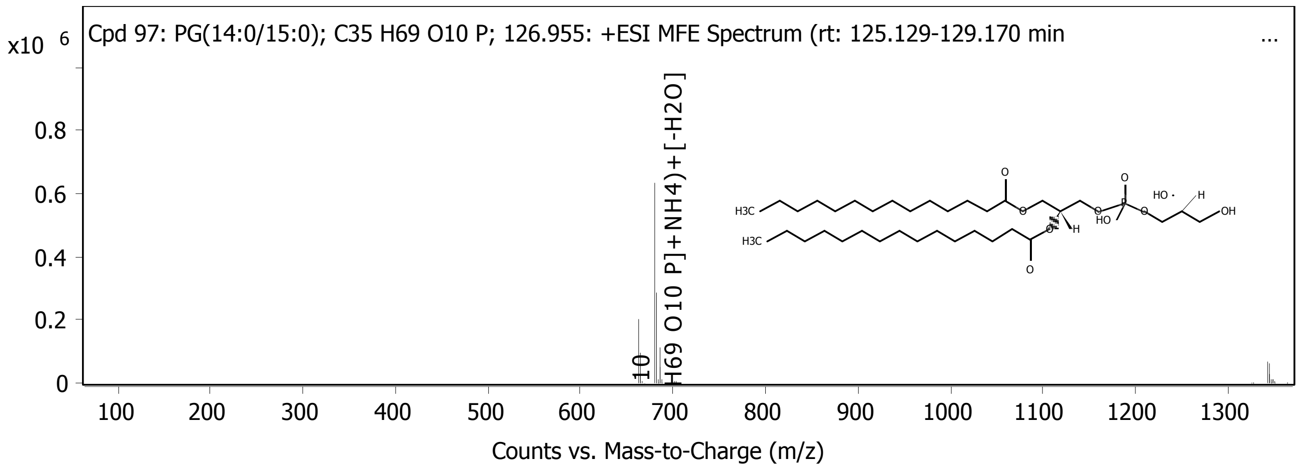

MFE MS Zoomed Spectrum

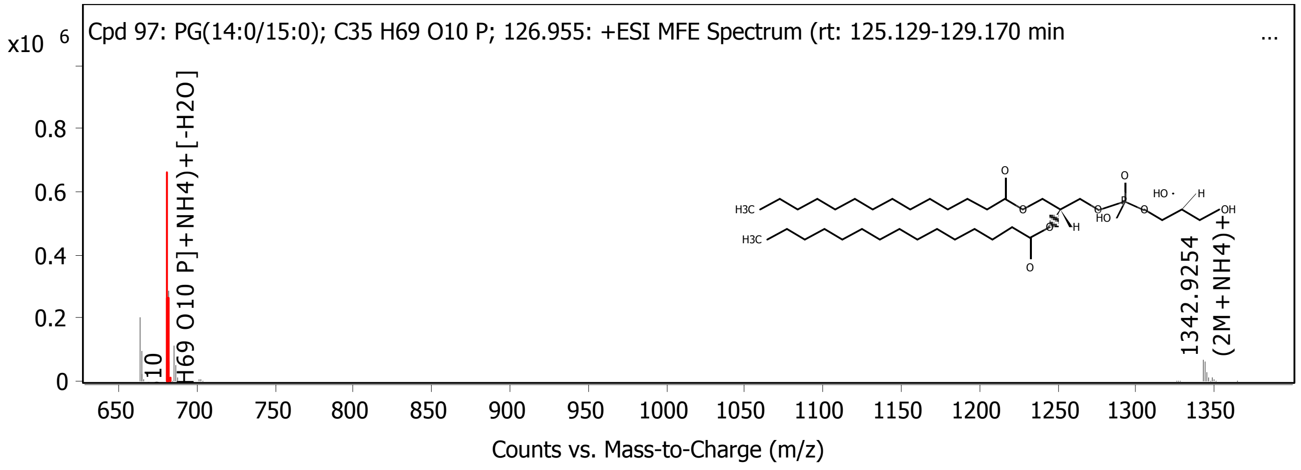

MS Spectrum

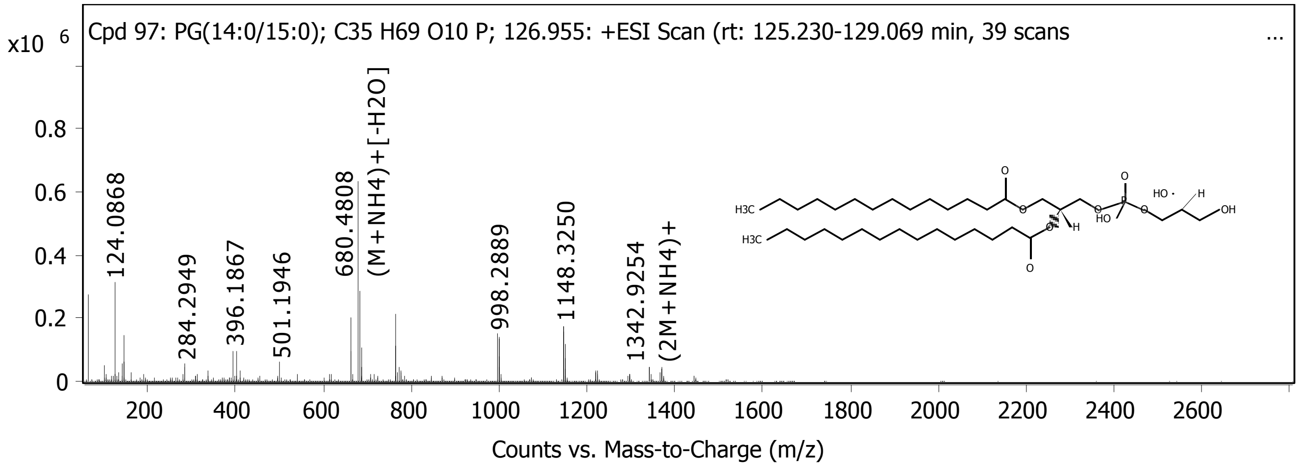

MS Zoomed Spectrum

Qualitative Compound Identification Report

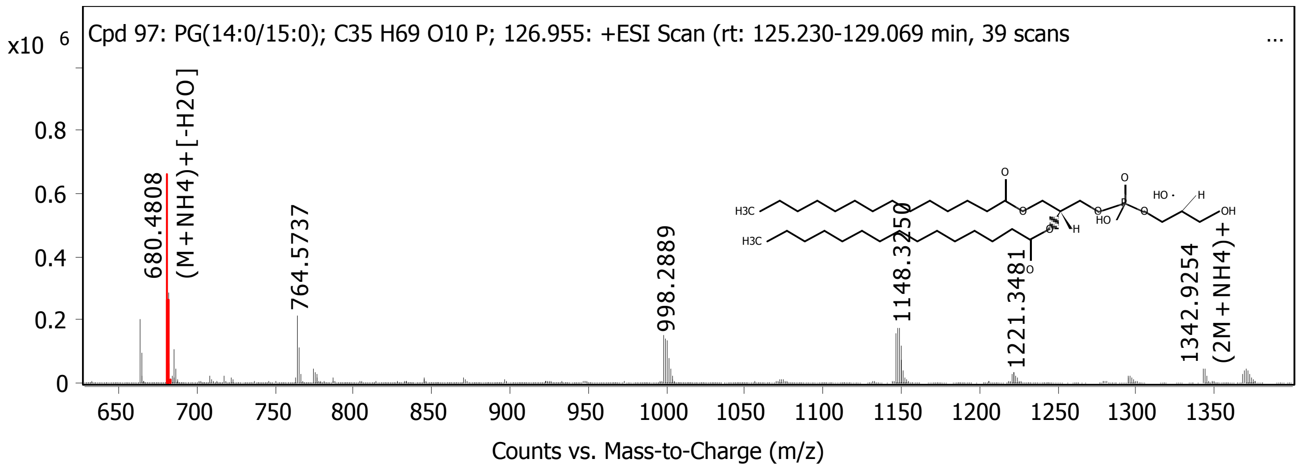

MSMS Spectrum

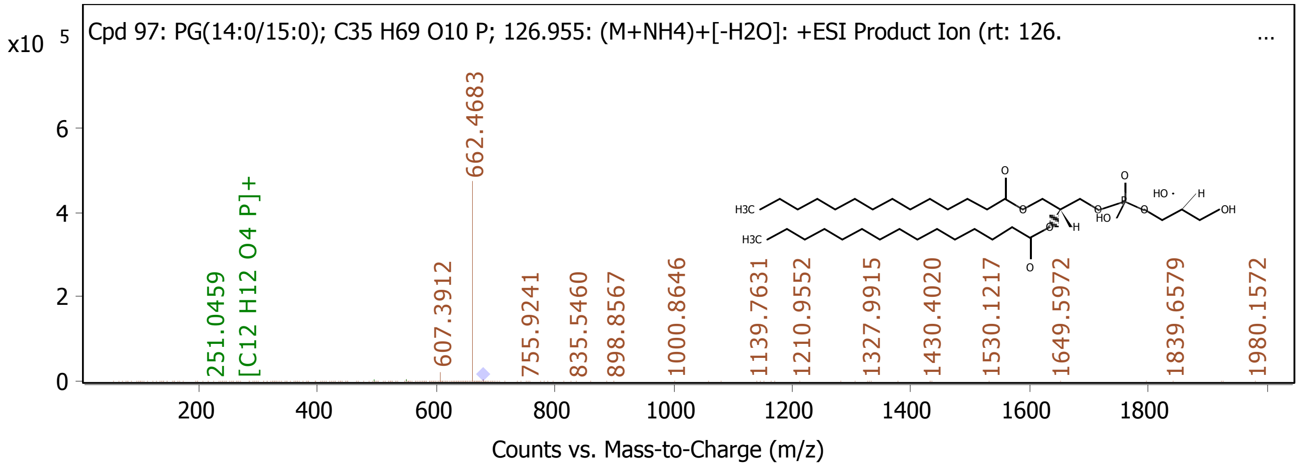

MSMS Spectrum

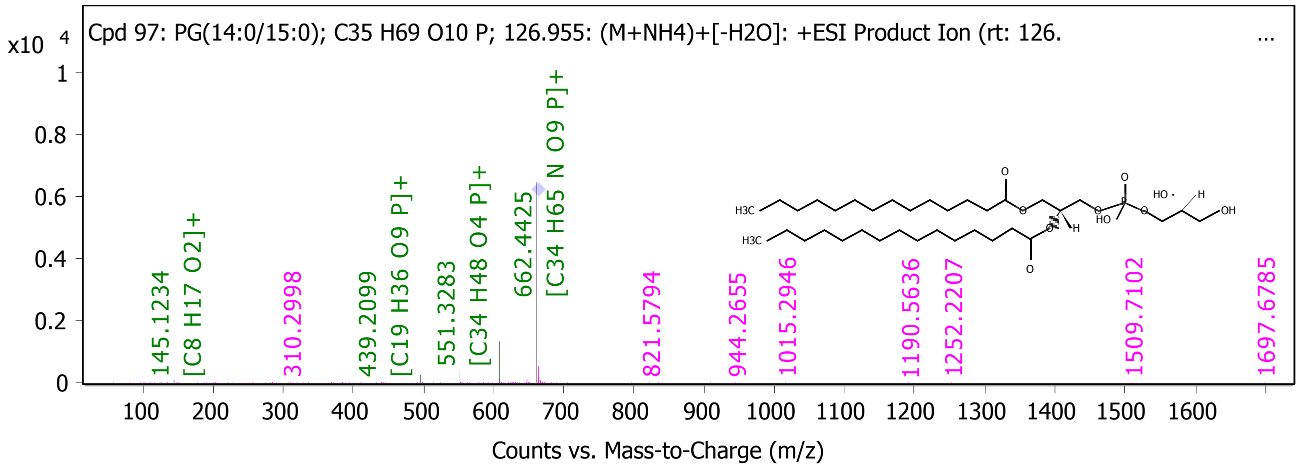

Identification Hit Table

Best Hit	Compound Name	RT	Formula	Notes	Match Score	Mass	Difference	Ion Species
✓	PG(14:0/15:0)	126.955	C35 H69 O10 P		70.49	680.4576	5.27	(M+NH4)+[-H2O]
	PG(17:0/12:0)	126.955	C35 H69 O10 P		70.49	680.4576	5.27	(M+NH4)+[-H2O]
	PG(16:0/13:0)	126.955	C35 H69 O10 P		70.49	680.4576	5.27	(M+NH4)+[-H2O]
	PG(15:0/14:0)	126.955	C35 H69 O10 P		70.49	680.4576	5.27	(M+NH4)+[-H2O]
	PG(13:0/16:0)	126.955	C35 H69 O10 P		70.49	680.4576	5.27	(M+NH4)+[-H2O]
	PG(12:0/17:0)	126.955	C35 H69 O10 P		70.49	680.4576	5.27	(M+NH4)+[-H2O]
	3-Hexanoyl-NBD cholesterol	126.955	C39 H58 N4 O5		66.16	662.4471	-6.35	(M+NH4)+
	Goyaglycoside c	126.955	C38 H62 O9		59.73	662.447	-7.61	(M+NH4)+

Identification Hit Table

Best Hit	Compound Name	RT	Formula	Notes	Match Score	Mass	Difference	Ion Species
✓	PG(12:0/17:0)	126.955	C35 H69 O10 P		70.49	680.4576	5.27	(M+NH4)+[-H2O]

Qualitative Compound Identification Report

	PG(13:0/16:0)	126.955	C35 H69 O10 P		70.49	680.4576	5.27	(M+NH4)+[-H2O]
	PG(15:0/14:0)	126.955	C35 H69 O10 P		70.49	680.4576	5.27	(M+NH4)+[-H2O]
	PG(16:0/13:0)	126.955	C35 H69 O10 P		70.49	680.4576	5.27	(M+NH4)+[-H2O]
	PG(17:0/12:0)	126.955	C35 H69 O10 P		70.49	680.4576	5.27	(M+NH4)+[-H2O]
	PG(14:0/15:0)	126.955	C35 H69 O10 P		70.49	680.4576	5.27	(M+NH4)+[-H2O]

Identification Hit Table

Best Hit	Compound Name	RT	Formula	Notes	Match Score	Mass	Difference	Ion Species

Compound Label	Name	*m/z*	RT	Algorithm	Mass
Cpd 98: Loroxanthin ester/ Loroxanthin dodecenoate; C52 H76 O4; 126.956	**Loroxanthin ester/ Loroxanthin dodecenoate**	764.5737	126.956	Find by Molecular Feature	764.5743

Compound Chromatograms

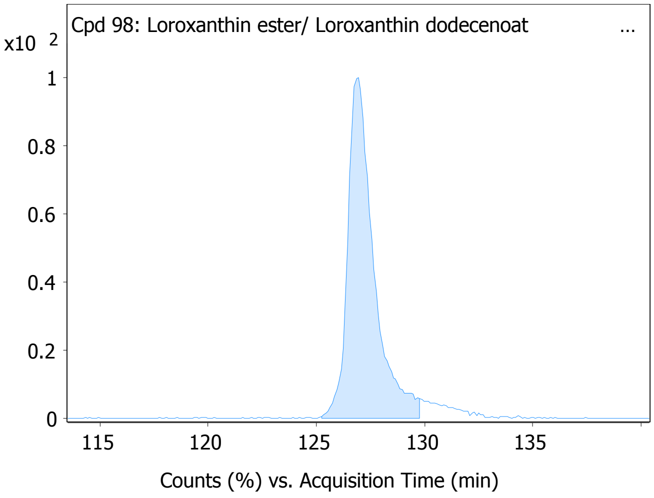

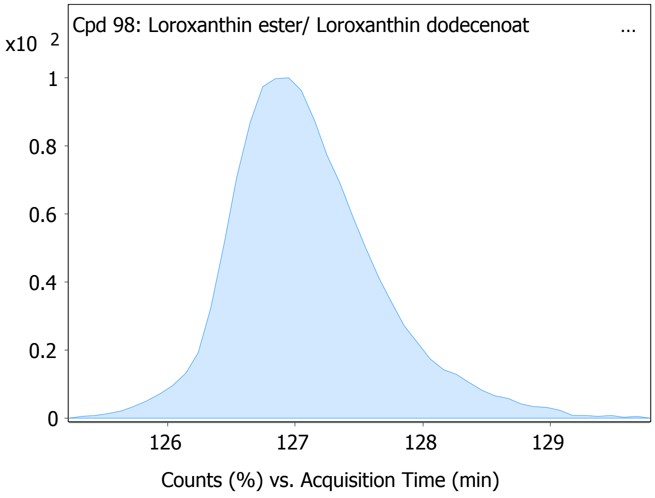

MFE MS Spectrum

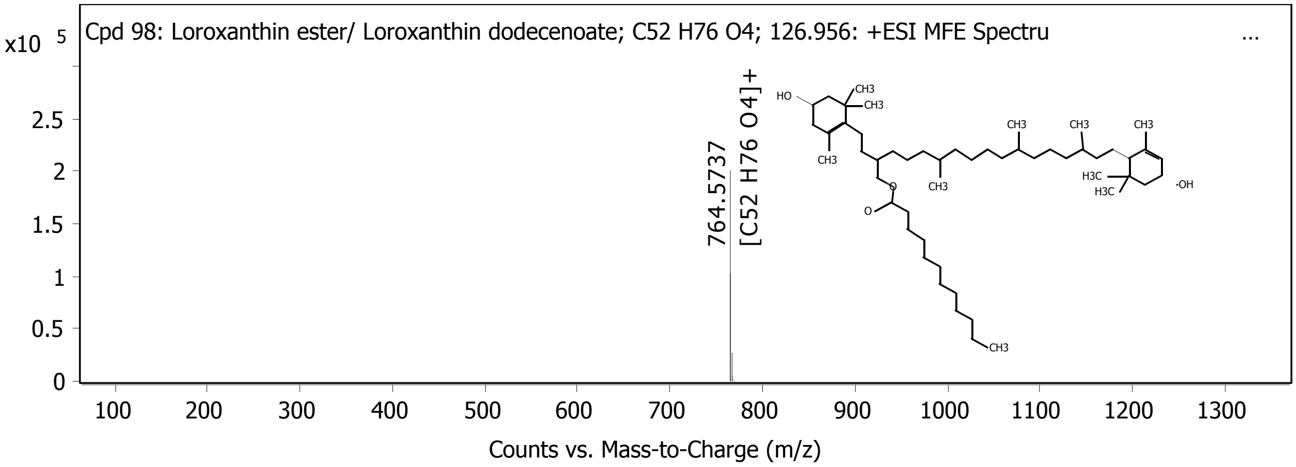

MFE MS Zoomed Spectrum

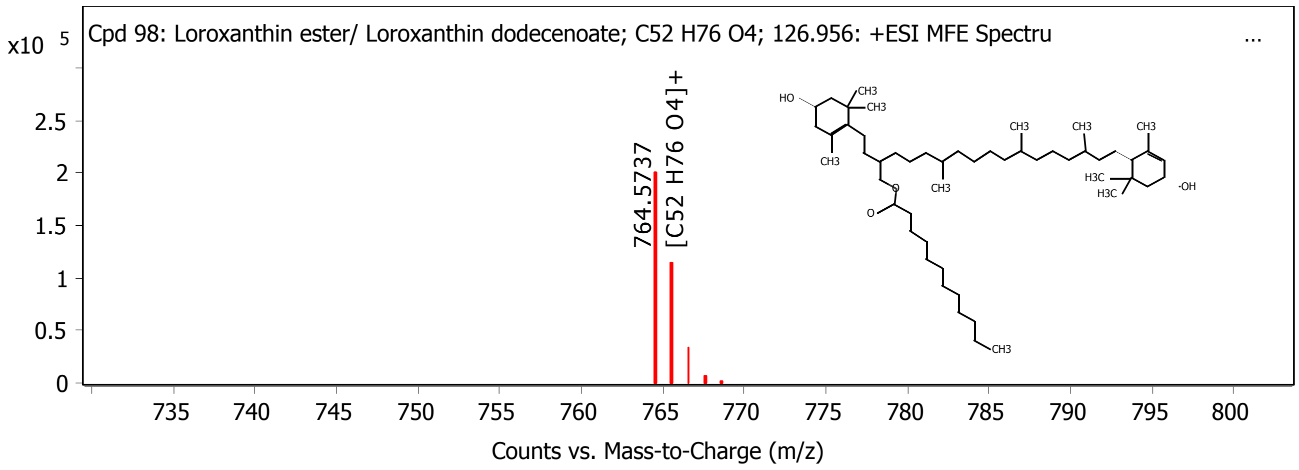

MS Spectrum

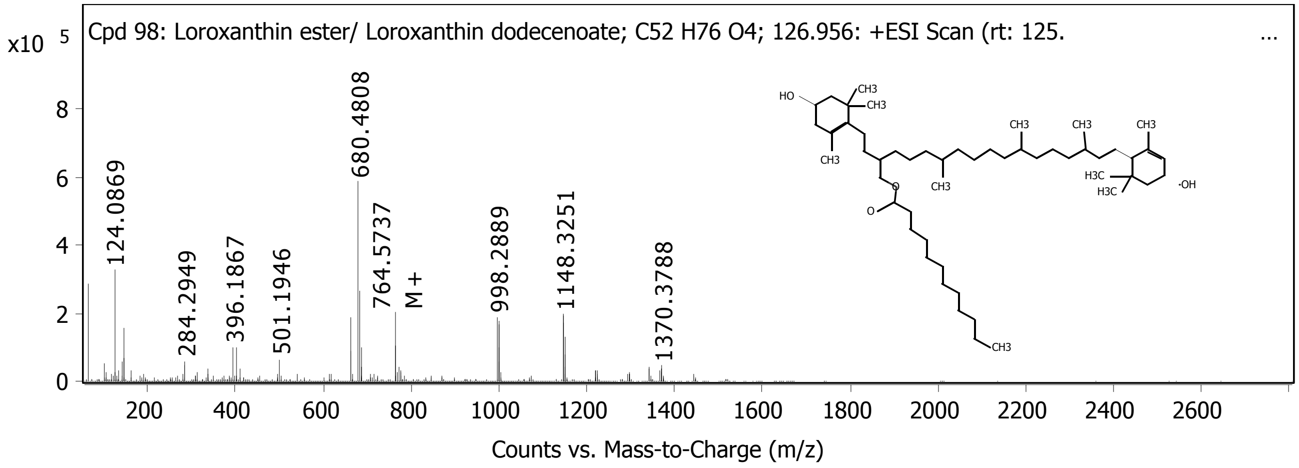

MS Zoomed Spectrum

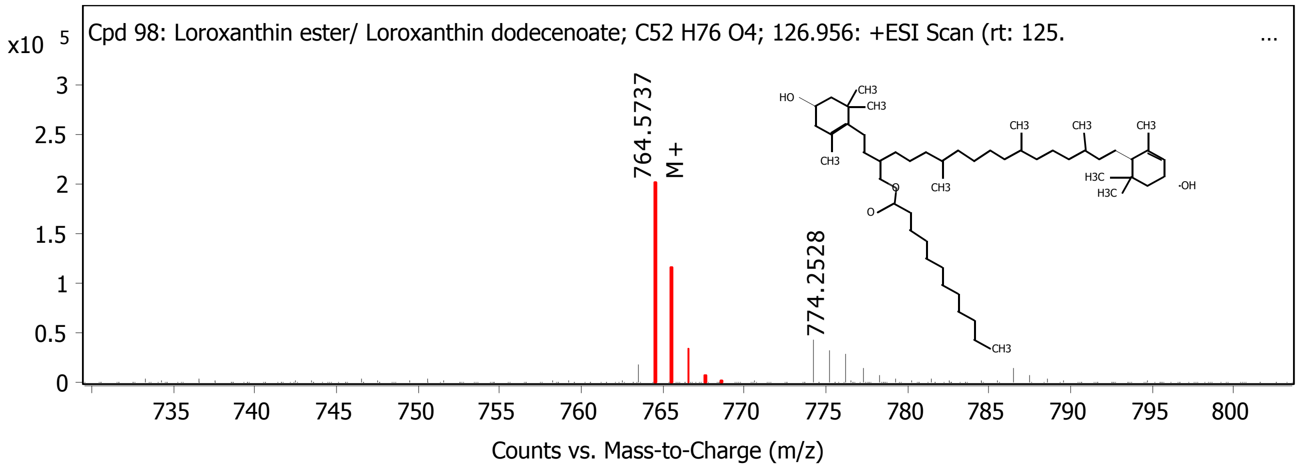

MSMS Spectrum

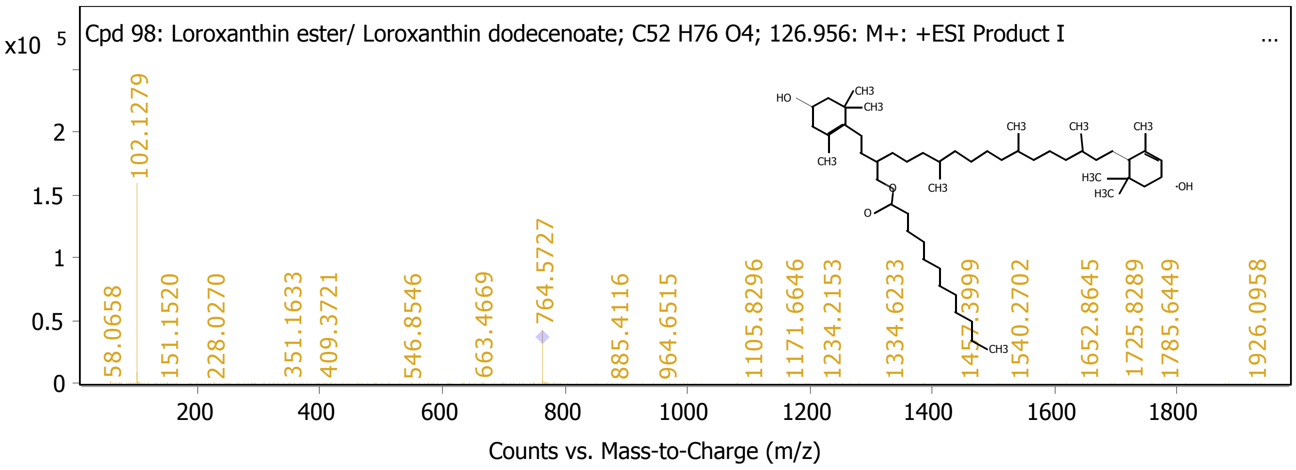

Identification Hit Table

Best Hit	Compound Name	RT	Formula	Notes	Match Score	Mass	Difference	Ion Species
✓	Loroxanthin ester/ Loroxanthin dodecenoate	126.956	C52 H76 O4		97.69	764.5743	0.08	M+
	PA(22:2(13Z,16Z)/20:1(11Z))	126.956	C45 H83 O8 P		94.04	782.5849	-2.31	M+[-H2O]
	PA(20:1(11Z)/22:2(13Z,16Z))	126.956	C45 H83 O8 P		94.04	782.5849	-2.31	M+[-H2O]
	PA(20:2(11Z,14Z)/22:1(11Z))	126.956	C45 H83 O8 P		94.04	782.5849	-2.31	M+[-H2O]
	PA(20:3(8Z,11Z,14Z)/22:0)	126.956	C45 H83 O8 P		94.04	782.5849	-2.31	M+[-H2O]
	PA(22:0/20:3(8Z,11Z,14Z))	126.956	C45 H83 O8 P		94.04	782.5849	-2.31	M+[-H2O]
	PA(22:1(11Z)/20:2(11Z,14Z))	126.956	C45 H83 O8 P		94.04	782.5849	-2.31	M+[-H2O]
	PA(P-20:0/22:4(7Z,10Z,13Z,16Z))	126.956	C45 H81 O7 P		93.86	764.5743	-2.31	M+
	3-dodecanoyl-NBD Cholesterol	126.956	C45 H70 N4 O5		74.41	746.54	-5.41	(M+NH4)+
	MGDG(18:1(9Z)/18:1(9Z))	126.956	C45 H82 O10		72.29	782.5849	5.93	M+[-H2O]

Identification Hit Table

Best Hit	Compound Name	RT	Formula	Notes	Match Score	Mass	Difference	Ion Species
✓	Loroxanthin ester/ Loroxanthin dodecenoate	126.956	C52 H76 O4		97.69	764.5743	0.08	M+
	PA(20:1(11Z)/22:2(13Z,16Z))	126.956	C45 H83 O8 P		94.04	782.5849	-2.31	M+[-H2O]
	PA(22:2(13Z,16Z)/20:1(11Z))	126.956	C45 H83 O8 P		94.04	782.5849	-2.31	M+[-H2O]
	PA(22:1(11Z)/20:2(11Z,14Z))	126.956	C45 H83 O8 P		94.04	782.5849	-2.31	M+[-H2O]
	PA(20:3(8Z,11Z,14Z)/22:0)	126.956	C45 H83 O8 P		94.04	782.5849	-2.31	M+[-H2O]
	PA(20:2(11Z,14Z)/22:1(11Z))	126.956	C45 H83 O8 P		94.04	782.5849	-2.31	M+[-H2O]
	PA(22:0/20:3(8Z,11Z,14Z))	126.956	C45 H83 O8 P		94.04	782.5849	-2.31	M+[-H2O]
	PA(P-20:0/22:4(7Z,10Z,13Z,16Z))	126.956	C45 H81 O7 P		93.86	764.5743	-2.31	M+
	MGDG(18:1(9Z)/18:1(9Z))	126.956	C45 H82 O10		72.29	782.5849	5.93	M+[-H2O]
	MGDG(18:0(9Z)/18:2(9Z,12Z))	126.956	C45 H82 O10		72.29	782.5849	5.93	M+[-H2O]

Identification Hit Table

Best Hit	Compound Name	RT	Formula	Notes	Match Score	Mass	Difference	Ion Species

Compound Label	Name	*m/z*	RT	Algorithm	Mass
Cpd 99: (S)-3-Hydroxytetradecanoyl-CoA; C35 H62 N7 O18 P3 S; 128.936	**(S)-3-Hydroxytetradecanoyl-CoA**	998.2887	128.936	Find by Molecular Feature	993.3061

Compound Chromatograms

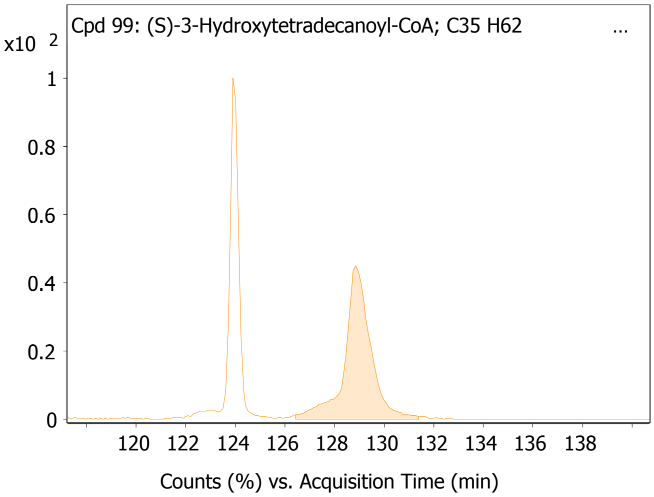

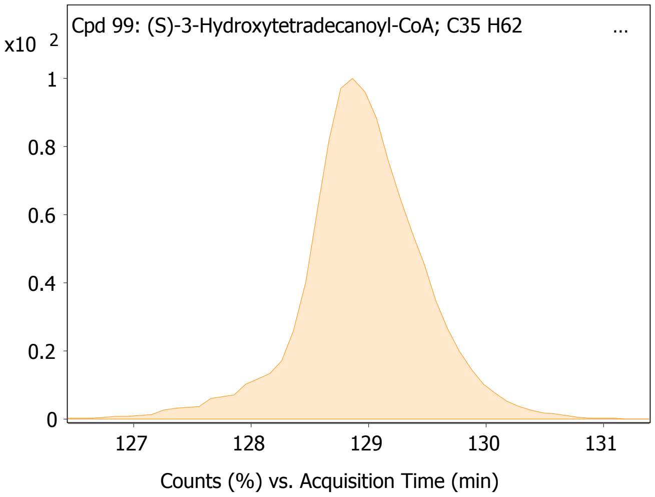

Qualitative Compound Identification Report

MFE MS Spectrum

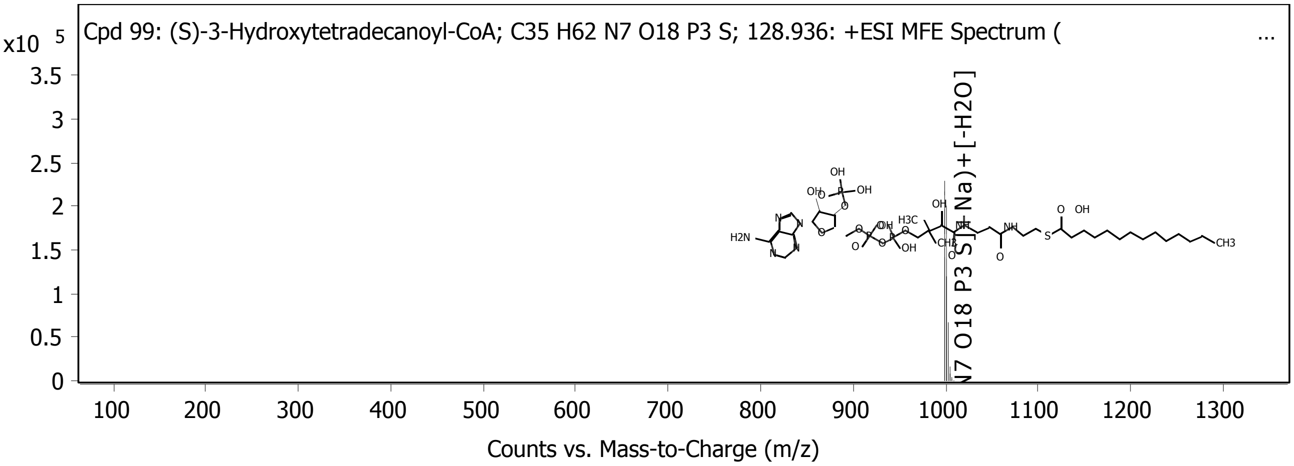

MFE MS Zoomed Spectrum

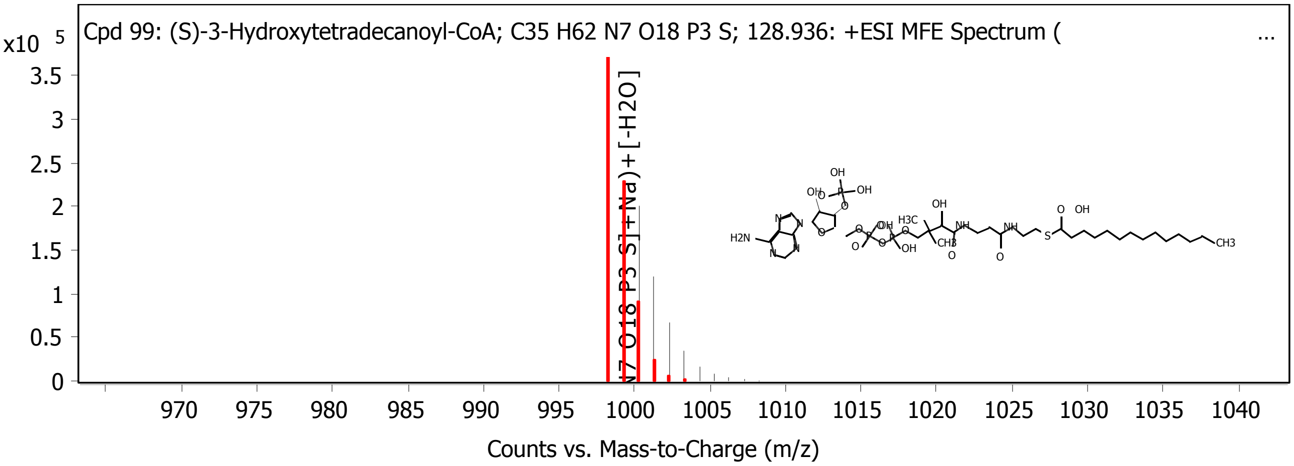

MS Spectrum

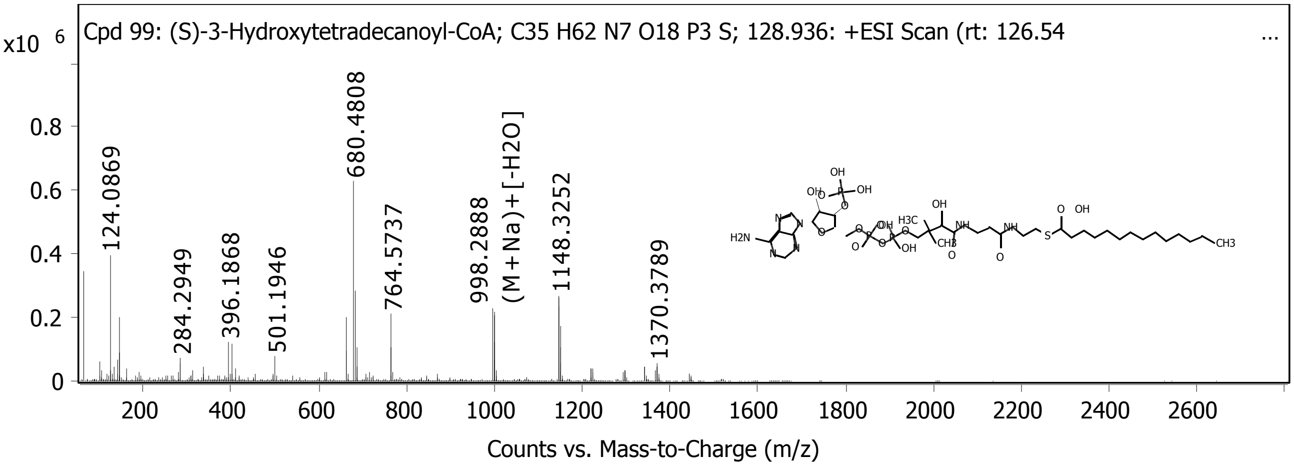

MS Zoomed Spectrum

Agilent Technologies

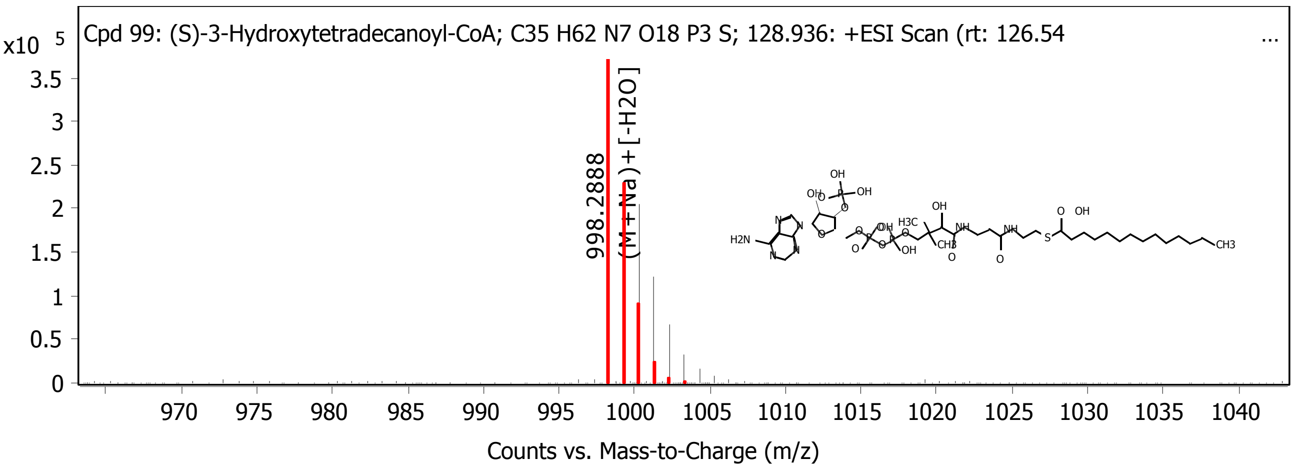

MSMS Spectrum

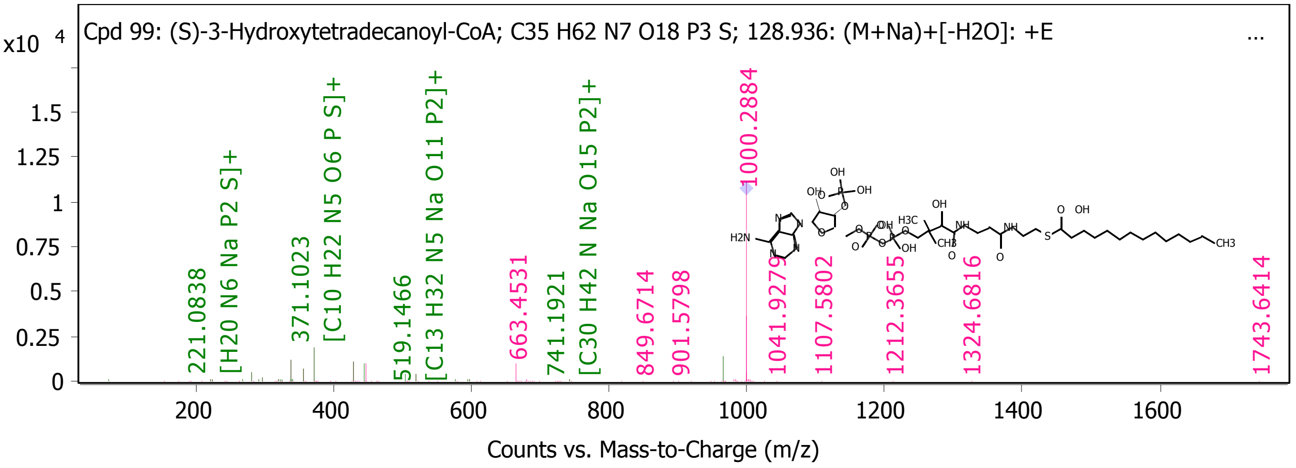

Identification Hit Table

Best Hit	Compound Name	RT	Formula	Notes	Match Score	Mass	Difference	Ion Species
✓	(S)-3-Hydroxytetradecanoyl-CoA	128.936	C35 H62 N7 O18 P3 S		63.88	993.3061	2.35	(M+Na)+[-H2O]
	3-hydroxymyristoyl-CoA	128.936	C35 H62 N7 O18 P3 S		63.88	993.3061	2.35	(M+Na)+[-H2O]
	11Z-tetradecenoyl-CoA	128.936	C35 H60 N7 O17 P3 S		63.76	975.2956	2.35	(M+Na)+
	11E-tetradecenoyl-CoA	128.936	C35 H60 N7 O17 P3 S		63.76	975.2956	2.35	(M+Na)+
	2E-tetradecenoyl-CoA	128.936	C35 H60 N7 O17 P3 S		63.76	975.2956	2.35	(M+Na)+
	OPC6-CoA	128.936	C37 H60 N7 O18 P3 S		52.47	1015.288	4.85	(M+H)+[-H2O]

Identification Hit Table

Best Hit	Compound Name	RT	Formula	Notes	Match Score	Mass	Difference	Ion Species
✓	3-hydroxymyristoyl-CoA	128.936	C35 H62 N7 O18 P3 S		63.88	993.3061	2.35	(M+Na)+[-H2O]
	2E-tetradecenoyl-CoA	128.936	C35 H60 N7 O17 P3 S		63.76	975.2956	2.35	(M+Na)+
	11E-tetradecenoyl-CoA	128.936	C35 H60 N7 O17 P3 S		63.76	975.2956	2.35	(M+Na)+
	11Z-tetradecenoyl-CoA	128.936	C35 H60 N7 O17 P3 S		63.76	975.2956	2.35	(M+Na)+

Identification Hit Table

Best Hit	Compound Name	RT	Formula	Notes	Match Score	Mass	Difference	Ion Species

Compound Label	Name	*m/z*	RT	Algorithm	Mass
Cpd 100: Retinoyl CoA; C41 H62 N7 O17 P3 S; 136.205	**Retinoyl CoA**	1072.307	136.205	Find by Molecular Feature	1049.3137

Compound Chromatograms

Qualitative Compound Identification Report

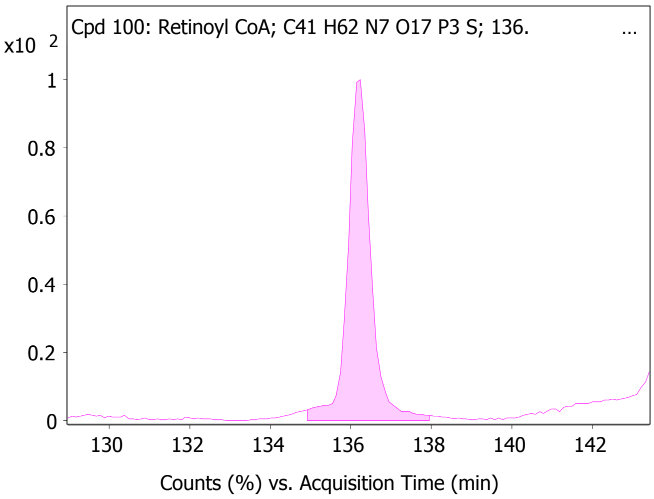

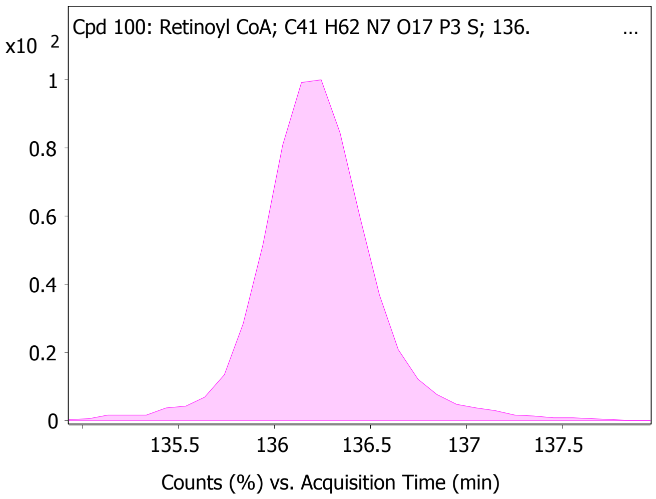

MFE MS Spectrum

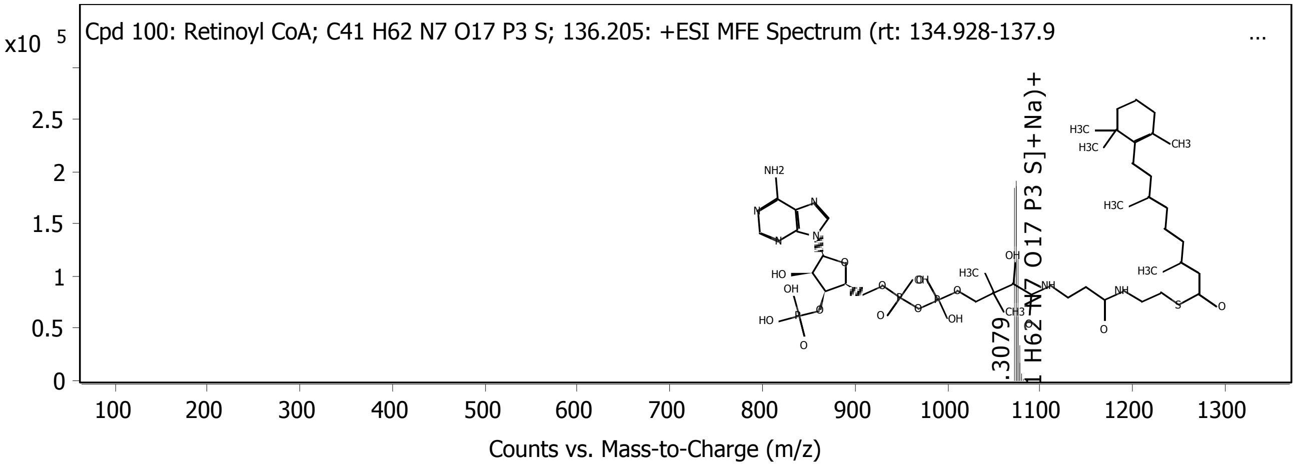

MFE MS Zoomed Spectrum

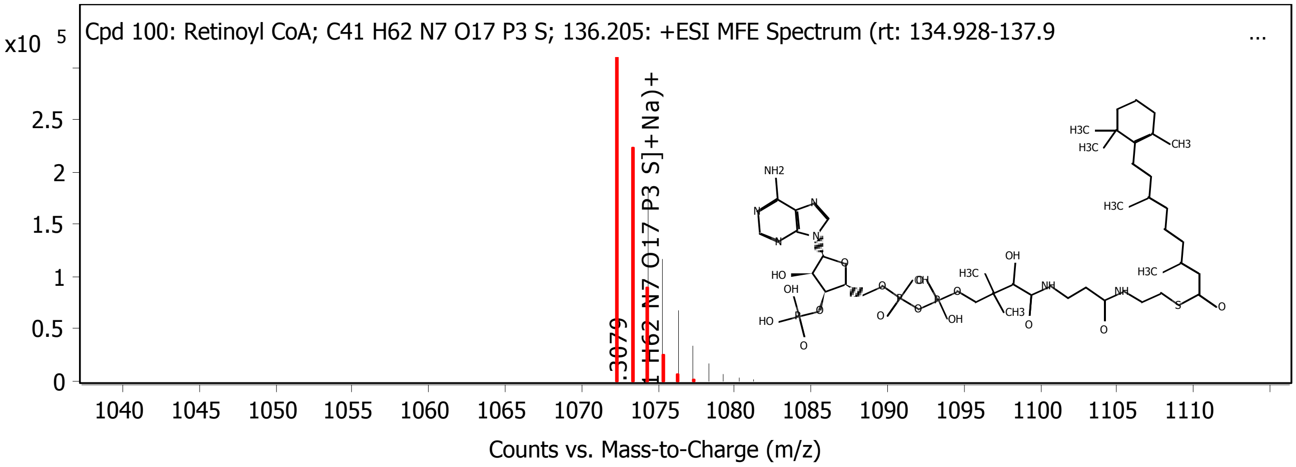

MS Spectrum

Qualitative Compound Identification Report

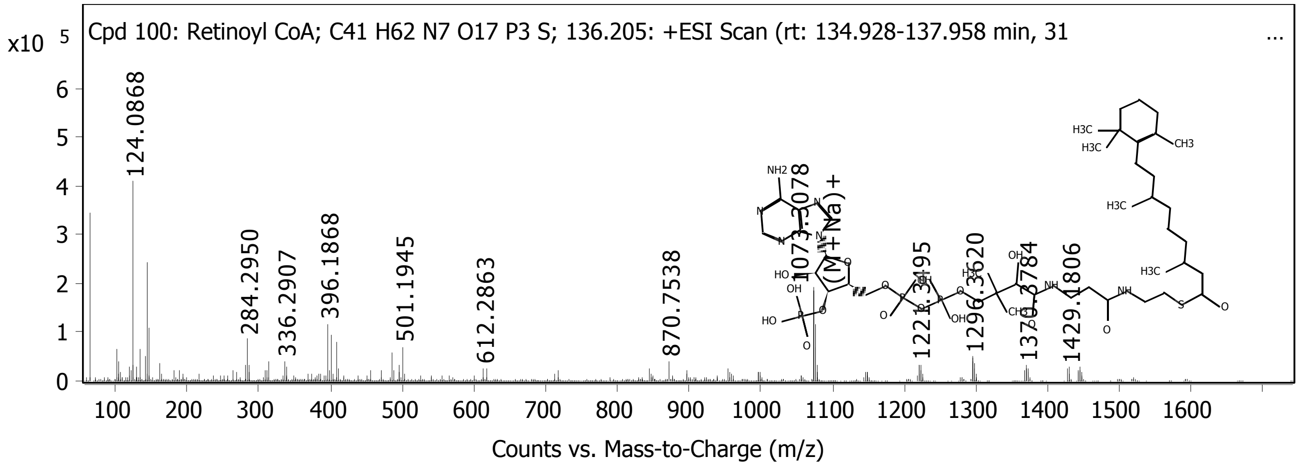

MS Zoomed Spectrum

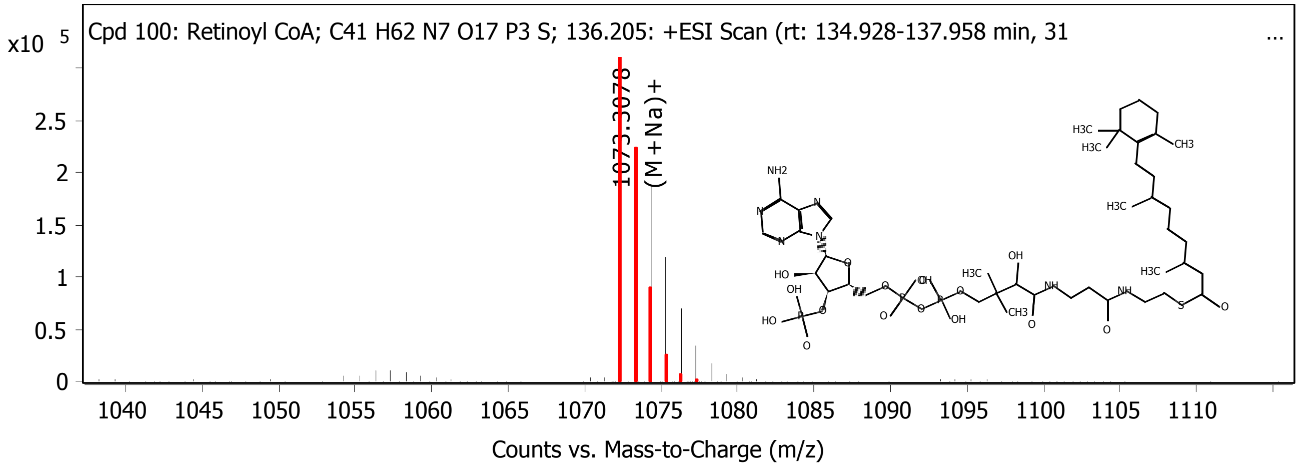

Identification Hit Table

Best Hit	Compound Name	RT	Formula	Notes	Match Score	Mass	Difference	Ion Species
✓	Retinoyl CoA	136.205	C41 H62 N7 O17 P3 S		68.45	1049.3137	-0.13	(M+Na)+

Identification Hit Table

Best Hit	Compound Name	RT	Formula	Notes	Match Score	Mass	Difference	Ion Species

Identification Hit Table

Best Hit	Compound Name	RT	Formula	Notes	Match Score	Mass	Difference	Ion Species

--- End Of Report ---
